# Regioselective alkylation of a versatile indazole: Electrophile scope and mechanistic insights from density functional theory calculations

**DOI:** 10.3762/bjoc.20.170

**Published:** 2024-08-09

**Authors:** Pengcheng Lu, Luis Juarez, Paul A Wiget, Weihe Zhang, Krishnan Raman, Pravin L Kotian

**Affiliations:** 1 Department of Discovery Chemistry, BioCryst Pharmaceuticals Inc., Discovery Center of Excellence, 2100 Riverchase Center Building 200, Suite 200 Birmingham, AL, 35244, USA; 2 Department of Computational Chemistry and Structural Biology, BioCryst Pharmaceuticals Inc., Discovery Center of Excellence, 2100 Riverchase Center Building 200, Suite 200 Birmingham, AL, 35244, USA

**Keywords:** DFT, indazole, indazole substitution, mechanism, *N*^1^-substituted indazole, *N*^2^-substituted indazole, regioselectivity

## Abstract

Herein, we report a pair of regioselective *N*^1^- and *N*^2^*-*alkylations of a versatile indazole, methyl 5-bromo-1*H*-indazole-3-carboxylate (**6**) and the use of density functional theory (DFT) to evaluate their mechanisms. Over thirty *N*^1^- and *N*^2^-alkylated products were isolated in over 90% yield regardless of the conditions. DFT calculations suggest a chelation mechanism produces the *N*^1^-substituted products when cesium is present and other non-covalent interactions (NCIs) drive the *N*^2^-product formation. Methyl 1*H*-indazole-7-carboxylate (**18**) and 1*H*-indazole-3-carbonitrile (**21**) were also subjected to the reaction conditions and their mechanisms were evaluated. The *N*^1^- and *N*^2^-partial charges and Fukui indices were calculated for compounds **6**, **18**, and **21** via natural bond orbital (NBO) analyses which further support the suggested reaction pathways.

## Introduction

**Figure 1:**
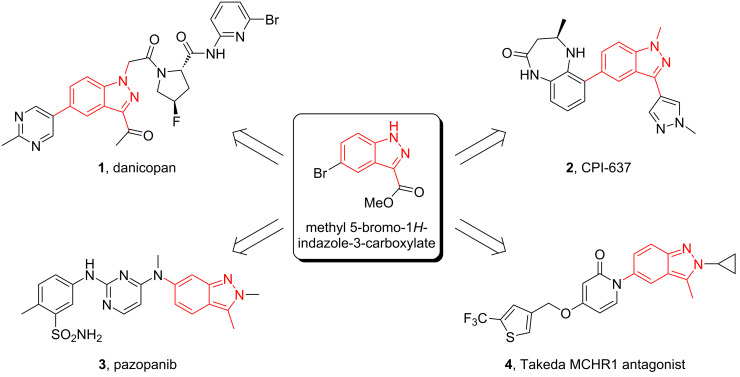
Indazole-containing bioactive molecules*.*

Indazoles constitute an important class of heterocycles with interesting biological and medicinal properties. Indazole, also called benzpyrazole, is a heterocyclic organic compound commonly found as a structural motif in natural products, pharmaceuticals, agrochemicals, and bioactive compounds [1–6]. Indazole-containing compounds possess a wide range of pharmacological activities, such as anti-inflammatory, anti-arrhythmic, antitumor, antifungal, antibacterial, and anti-HIV activities [7–13]. For example, two *N*^1^-substituted bioactive indazoles are found in Figure 1, danicopan (**1**), a complement factor D inhibitor for the treatment of paroxysmal nocturnal hemoglobinuria, and CPI-637 (**2**), an inhibitor of both cyclic-AMP response element binding protein (CBP) and adenoviral E1A binding protein [14–16]. The *N*^2^-substituted indazole analogs pazopanib (**3**), an FDA-approved tyrosine kinase inhibitor used for the treatment of renal cell carcinoma, and Takeda’s MCHR1 antagonist **4** further exemplify indazole’s biological importance. Generally, direct alkylation of 1*H*-indazoles leads to a mixture of *N*^1^- and *N*^2^-substituted products [17–20]. Procedures that selectively produce either *N*^1^- or *N*^2^-substituted indazoles would provide greater synthetic utility for this valuable heterocycle. These examples suggest a common intermediate such as methyl 5-bromo-1*H*-indazole-3-carboxylate (**6**) could be used to generate such compounds. An alkylation strategy that uses the vast array of commercially available alcohols as precursors of alkylating reagents presents an opportunity to find new ways to diversify indazole reactivity by simple modifications of reaction conditions. Mechanistic understanding would allow for further diversification in related systems.

**Figure 2:**
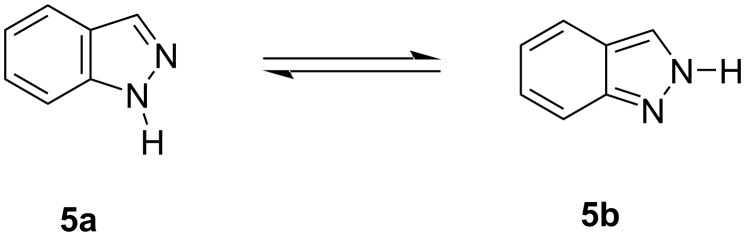
Tautomerism of indazole.

The indazole ring presents annular tautomerism regarding the position of the NH hydrogen atom: 1*H*-indazole (**5a**, benzenoid 1*H*-indazole tautomer) and 2*H*-indazole (**5b**, quinonoid 2*H*-indazole tautomer) (Figure 2) [21–23]. Since 1*H*-indazole is thermodynamically more stable than 2*H*-indazole, **5a** is the predominant tautomer [24–26]. Conventionally, indazoles are employed as nucleophiles in chemical transformations, and a mixture of both *N*^1^- and *N^2^*-alkylated products is formed, depending on the reaction conditions, with little selectivity in regards to substituent effects [27–33].

Considering the importance of indazoles as a widely used pharmacophore in medicinal chemistry and the challenges in obtaining either *N*^1^- or *N*^2^-alkylated indazoles as the dominant regioisomer [30,34–39], we were interested in exploring the regioselectivity with methyl 5-bromo-1*H*-indazole-3-carboxylate (**6**, Figure 1) as a multifunctional model compound for *N*^1^*/N*^2^ discrimination studies. The existing approaches for generating *N*-substituted indazoles of compound **6** often lead to a mixture of *N*^1^- and *N*^2^-alkylated indazoles with either low selectivity or moderate yields depending on the conditions used. For example, Takahashi et al. obtained *N*^1^- and *N*^2^-substituted indazole analogs in 44% and 40% yields, respectively, by treating compound **6** with methyl iodide and potassium carbonate in dimethylformamide (DMF) at room temperature for 17 h [40]. Other works have shown poor selectivity when **6** and other isomers similar to **6** were reacted with isopropyl iodide and potassium carbonate, isopropyl bromide and cesium carbonate, and bromocyclohexane with potassium carbonate, and only afforded yields not higher than 52% in various solvents [41–43].

Recently, Alam and Keeting [37] explored the regioselectivity in the alkylation of variously substituted indazoles similar to **6**. They observed high *N*^1^-selectivity using NaH in THF with pentyl bromide and electron-deficient indazoles, postulating a coordination of the indazole *N*^2^-atom and an electron-rich oxygen atom in a C-3 substituent with the Na^+^ cation from NaH. Under anhydrous conditions the yields ranged from 44% at room temperature to 89% when warmed to 50 °C. No information was provided to justify any *N*^2^-selectivity or the lack thereof. Should an *N*^2^–Cs^+^–O ion pair exist, this could reasonably account for all the reported results presented herein (vide infra). Additionally, using Cs_2_CO_3_ in dioxane provided no products at room temperature (see Table 1) presumably due to the low solubility of Cs_2_CO_3_ in dioxane. They also provided a single example of a Mitsunobu reaction utilizing *n*-pentanol, dibutyl azodicarboxylate (DBAD), and PPh_3_.

**Table 1:** Representative reactions from reference [37].

Electrophile	Solvent	Reagents	Temp (°C)	Time (h)	Major product isolated yield	*N*^1^:*N*^2^

*n-*C_5_H_11_Br	1,4-dioxane	Cs_2_CO_3_	rt	16	0%	n.a.
*n-*C_5_H_11_Br	THF	NaH	0 → 50	24	89%	>99:1
*n-*C_5_H_11_OH	THF	DBAD, PPh_3_	0 → rt	2	58%	1:2.9

Therefore, there is still a great need to develop an operationally simple and mild method to selectively generate *N*^1^- or *N*^2^-substituted indazole analogs when the substituents appear to favor one over the other. Ideally, it would be greatly beneficial if the desired high regioselectivity on *N^1^* or *N^2^* could be achieved when commercially available chemicals, such as alcohols, react with **6** under different reaction conditions. In this paper, we report a concise and efficient approach to prepare *N*^1^- and *N*^2^-substituted analogs with high selectivity and excellent yields (>84%) from the same substrates: alcohols and 5-bromo-1*H*-indazole-3-carboxylate (**6**).

## Results and Discussion

**Scheme 1:**
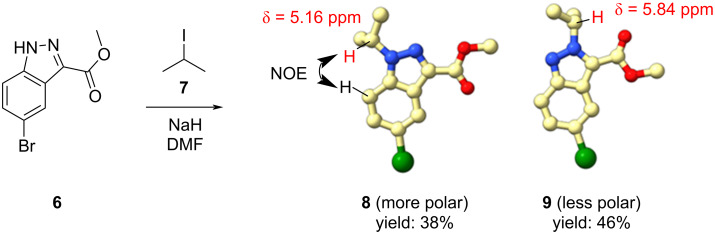
NMR, NOE, and yield data of compounds **8** and **9**.

We commenced our studies by investigating yields of *N*^1^*-* and *N*^2^*-*substituted products of conventional indazole alkylation reactions using our model substrate methyl 5-bromo-1*H*-indazole-3-carboxylate (**6**) and confirmed structures of the corresponding *N*^1^-substituted and *N*^2^-substituted products. In Scheme 1 compound **6** was treated with isopropyl iodide (**7**) in DMF in the presence of sodium hydride to provide products **8** and **9** in 38% and 46% yields, respectively. The structures of both compounds were unambiguously assigned using X-ray crystallography and ^1^H and nuclear Overhauser effect (NOE) NMR spectroscopy (see Supporting Information File 1).

**Scheme 2:**
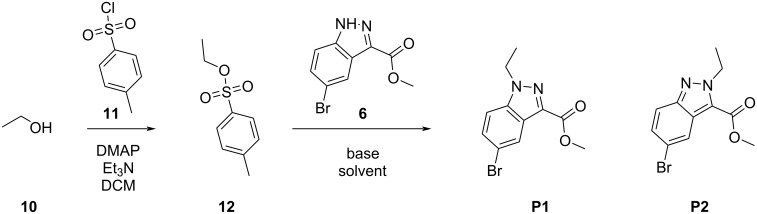
Synthesis of compounds **P1** and **P2**.

From the yield of products **8** and **9**, we decided to explore new reaction conditions to improve the yields of the *N*^1^-substituted indazole analogs. As shown in Scheme 2, compound **12** was prepared by treating ethanol (**10**) with tosyl chloride (**11**) in the presence of 4-dimethylaminopyridine (DMAP) and triethylamine. Used as a model system, the subsequent reaction of sulfonate **12** with compound **6** under varied conditions afforded products **P1** and **P2**. We investigated effects of reagent stoichiometry, bases, reaction time, and temperature on the yields of product **P1**, as summarized in Table S1 in Supporting Information File 1. Stoichiometric manipulation of **12** to **6** in DMF at 90 °C provided the *N*^1^-substituted product **P1** in 52–60% yields. The yields of **P1** formation were largely unaffected in DMF with temperatures ranging from room temperature to 110 °C. Varying the equivalents of Cs_2_CO_3_ showed little effect (averaging 52% yield), however, using NaH and lowering the temperature to rt lowered the **P1** yield, averaging 32%. Finally, the effect of other solvents at 90 °C was investigated and the results are summarized in Table 2. Entry 1 shows the best conditions for the above reaction in DMF. The use of chlorobenzene slightly improved the yield to 66%. The reaction in dioxane at 90 °C, entry 6, had a 96% yield. This was a surprising outcome as Keeting observed no reaction in dioxane at rt, suggesting the concentration of Cs_2_CO_3_ is significantly increased at this temperature.

**Table 2:** Effect of solvent on indazole *N*^1^ yield.^a^

Entry	Solvent	**P1** (isolated yield, %)

1	DMF	60
2	DMSO	54
3	NMP	42
4	chlorobenzene	66
5	toluene	56
6	dioxane	96

^a^DMF: dimethylformamide; DMSO: dimethyl sulfoxide; NMP: *N*-methyl-2-pyrrolidone. Reaction conditions: 1.5 equiv **12**, 1.0 equiv **6**, 2.0 equiv Cs_2_CO_3_, 90 °C, 2 h.

With the promising yield results of **P1**, we next explored the scope of this transformation using a variety of alcohols (**13a**–**q**, Table 3) and report their regioselectivity as determined by crude LC–MS. Sulfonates **14a–q** were prepared as described above or purchased (see Supporting Information File 1). The subsequent reactions with compound **6** afforded the *N*^1^-substituted indazole analogs **15a–q** with excellent yields (>90%), except for **15m**, which failed to form after multiple attempts likely due to an instability of the electrophile **14m** under optimized conditions (conditions A: 1.5 equiv tosylate, 1.0 equiv **6**, 2.0 equiv Cs_2_CO_3_, 90 °C, 2 h). Compounds containing linear or branched alkyl substitutions (**15a–g**), varied sizes of cycloalkane or saturated heterocycles (**15h–p**), including *S*- and *R*-tetrahydrofuran substitutions (**15j**, **15k**) were isolated in excellent yields (>90%). Azetane **14m** was unreactive towards alkylation in the presence of Cs_2_CO_3_.

**Table 3:** Scope of transformation and regioselectivity.

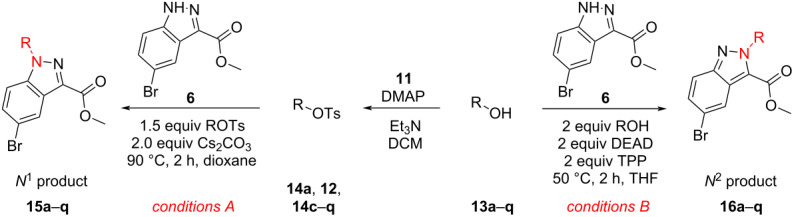						

R	Conditions A, major product	Isolated yield **15a**–**q** (%)	*N*^1^/*N*^2^ (LC–MS)	Conditions B, major product	Isolated yield **16a**–**q** (%)	*N*^2^/*N*^1^ (LC–MS)

CH_3_	**15a**	90	7.5	**16a**	92	4.0

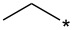	**15b**	96	12.5	**16b**	93	2.0

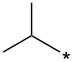	**15c**	90	7.5	**16c**	91	8.6

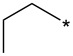	**15d**	94	13.1	**16d**	92	3.9

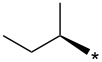	**15e**	95	9.5	**16e**	97	8.2

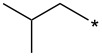	**15f**	96	13.8	**16f**	93	5.1

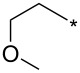	**15g**	96	12.6	**16g**	95	3.4

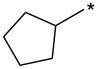	**15h**	94	7.7	**16h**	90	9.6

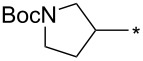	**15i**	96	13.4	**16i**	95	12.1

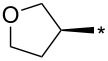	**15j**	94	14.8	**16j**	91	8.0

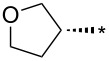	**15k**	96	12.4	**16k**	97	3.9

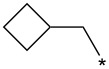	**15l**	91	13.9	**16l**	90	4.0

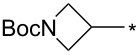	**15m**	n.r.	–	**16m**	93	–

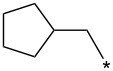	**15n**	94	>99	**16n**	96	8.7

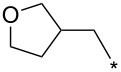	**15o**	94	10.2	**16o**	98	5.6

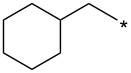	**15p**	95	15.8	**16p**	95	4.9

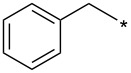	**15q**	96	20.2	**16q**	91	2.2

To explore the possibility of *N*^2^-selectivity, we hypothesized that the phosphine intermediate of a Mitsunobu reaction could provide chelation control, directing alkylation to the indazole *N*^2^-atom while using identical alcohols as described above. Thus, we subjected **6** to simple and mild Mitsunobu conditions for the preparation of *N*^2^-substituted indazole analogs **16a**–**q**. By directly reacting compound **6** with alcohols **13a**–**q** (2 equiv), diethyl azodicarboxylate (DEAD, 2 equiv), and triphenylphosphine (TPP, 2 equiv) in THF at 50 °C (conditions B), the corresponding *N*^2^-substituted products were isolated in excellent yields (>90%) and high regiocontrol.

Crude product ratios as determined by LC–MS (averaging integrations at 254 nm and 260 nm) had an average error (standard deviation) of 3.2% (2.76%) and 13.7% (7.54%) for conditions A and B, respectively. The *N*^1^-isomer overlapped with OPPh_3_ contributing to the increased error and standard deviation. Compound **6** was completely consumed and not detected (see Supporting Information File 1).

## Mechanistic considerations

**Figure 3:**
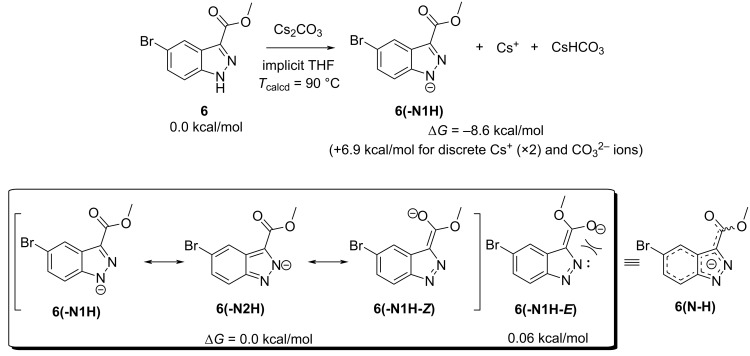
DFT-calculated deprotonation of **6** with Cs_2_CO_3_ in implicit THF with the temperature of the calculation set to 90 °C to simulate the dioxane conditions (top) and energy differences of four enolate resonance structures of **6** calculated as discrete structures. The hybrid is identified as **6(N-H)** (bottom).

**Figure 4:**
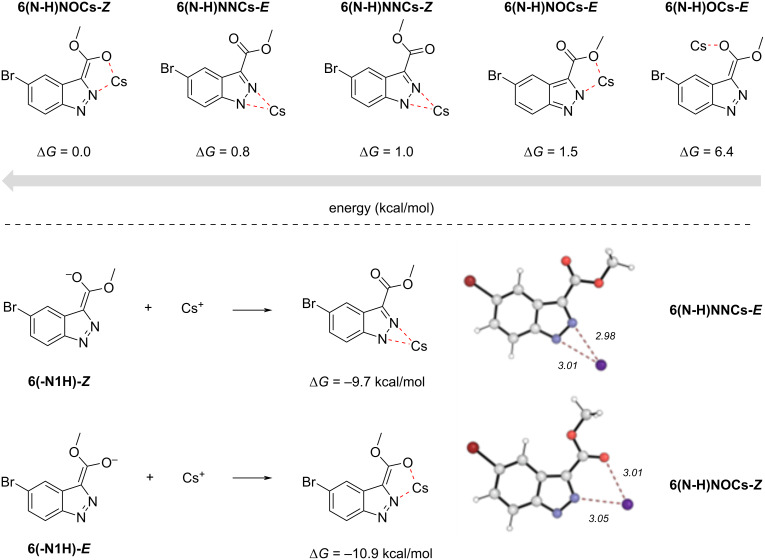
DFT-calculated Cs^+^-coordinated complexes with different enolate forms of **6(N-H)** calculated as isolated compounds (top) and calculated intermediates of the reactions of **6(-N1H-Z)** and **6(-N1H-E)** with Cs^+^ (bottom).

Alam and Keeting proposed a deprotonated intermediate that utilized the indazole *N*^2^ and C=O from an ester substituent at C-3 as a bidentate ligand to the Na^+^ cation from NaH. The tight ion pair would direct alkylation under conditions A to *N*^1^. As this and other postulations exist, we explored the possible mechanisms of each reaction conditions computationally. All calculations were performed in implicit THF at the reaction condition temperature using Gaussian 16: SMD(THF)-PBE0/def2-TZVP // SMD(THF)-PBE0/def2-SVP, def2-TZVP(Cs) at 50 °C (w/MeOPPh_3_^+^) or 90 °C (w/Cs^+^), utilizing Goodvibes to calculate thermochemistry. The energy of the *N*^1^- and *N*^2^-tautomers of **6** differ by 3.1 kcal/mol at 50 °C, favoring the *N*^1^-tautomer, implying a 7:1 distribution of isomers in solution. Concerning conditions A, compound **6** + Cs_2_CO_3_ was found to favor the deprotonated indazole with a free Cs^+^ ion by 8.6 kcal/mol (see Figure 3), however, when all ions are discrete (2 × Cs^+^ and CO_3_^2−^) the reaction becomes endergonic by 6.9 kcal/mol, presumably due to entropic penalties. Three of four computed resonance forms were all found to be of approximately equal energy. Only the *E*-enolate form **6** (-**N1H-*E***) was slightly higher in energy by 0.06 kcal/mol likely due to electrostatic destabilization of the oxyanion with *N*^2^, however, this difference is negligible. These data suggest that deprotonation occurs prior to alkylation and that deprotonation of either indazole tautomer leads to anions of identical or highly similar energy. Furthermore, as seen in Figure 4, a total, five coordinated complexes were found to be at least 4.5 kcal/mol more stable than the uncoordinated anion when calculated as isolated structures. When the calculation is performed as a reaction of *E* and *Z*-enolates with Cs^+^ ion, two coordinated complexes **6(N-H)NNCs-*E*** and**-6(N-H)NOCs-*Z*** are exergonically formed by 9.7 and 10.9 kcal/mol, respectively.

**Figure 5:**
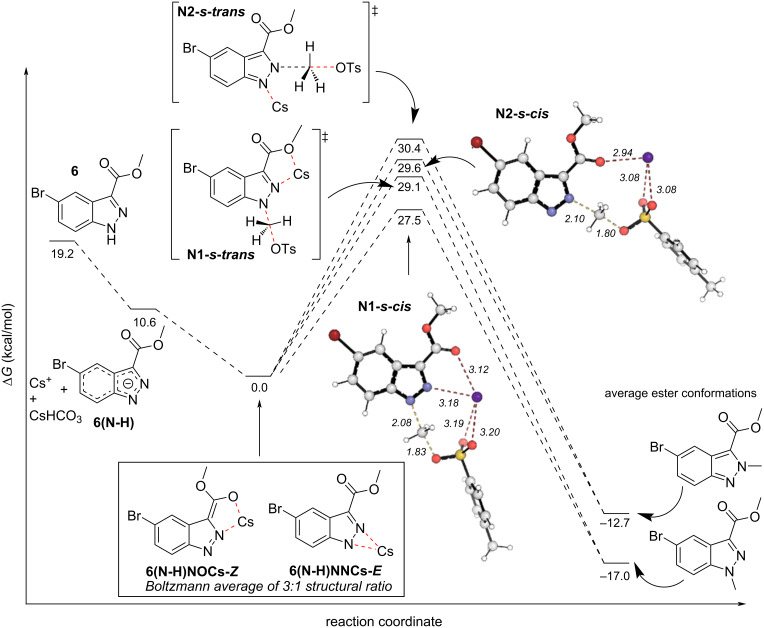
DFT-calculated reaction coordinate diagram for the reaction of **6** under conditions A. Concerning conditions B, we began our calculations under the assumption that MeOPPh_3_^+^ was already present in THF at 50 °C. The deprotonation by the dimethyl azodicarboxylate (DMAD) anion (DMAD^−^) to form **6(N-H)** and **17** was found favorable by 14.7 kcal/mol (Figure 6).

We then searched for transition state (TS) structures that would produce both the *N*^1^- and *N*^2^-products from CH_3_OTs as a model system. When the Boltzmann average of the cesium-coordinated intermediates is calculated, a 3:1 ratio of **6(N-H)NOCs-*Z***:**6(N-H)NNCs-*E*** is found. This average is 10.6 kcal/mol more stable than **6(N-H)**, and was subsequently set to 0 kcal/mol leading to the energy diagram in Figure 5. Two TSs leading to each product were found, all four of which utilized a coordinating Cs^+^ cation. The **N1-*s*-cis** and **N1-*s*-trans** TSs were the lowest in energy (27.5 kcal/mol and 29.1 kcal/mol, respectively), leading to two conformations of the *N*^1^-product with highly similar energy (averaging −16.8 kcal/mol). The **N2-*s*-cis** and **N2-*s*-trans** TSs leading to the *N*^2^-product were higher in energy and led to the higher energy *N*^2^ products. The critical difference between **N1-*s*-cis** and **N2-*s*-cis** is the presence of the *N*^2^–Cs^+^–O non-covalent interaction (NCI) in **N1-*s*-cis**, which accounts for the 2.1 kcal/mol difference in energy. Calculations showed that the sulfonate oxygens also chelate the cesium ion in both TSs. Thus, nitrogen NCIs with cesium, or lack thereof, seem to drive *N*^1^-product formation, which is both kinetically and thermodynamically favorable under conditions A.

**Figure 6:**
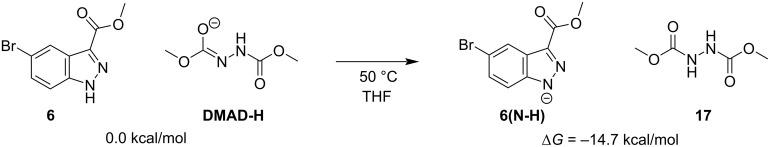
DFT-calculated energy for the deprotonation of **6** by the DMAD anion.

**Figure 7:**
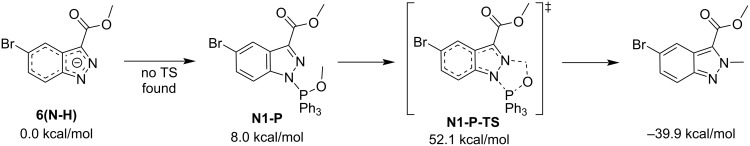
DFT-calculations concerning a coordinated Mitsunobu reaction pathway.

**Figure 8:**
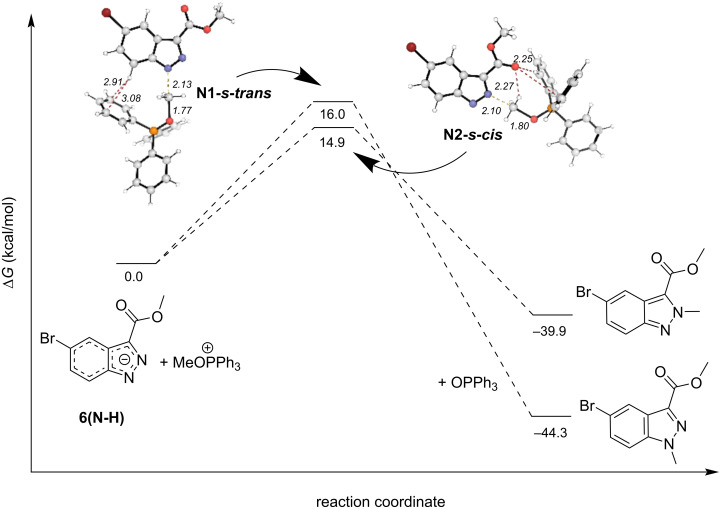
Reaction coordinate diagram of **6(N-H)** reacting under conditions B. All calculated energies in kcal/mol. Ball-and-stick transition-state structures are provided for the lowest energy *N*^1^- and *N*^2^-transition states with favorable NCIs shown as red dashed lines.

Postulating that O or N-dative interactions with phosphorus were responsible for the high *N*^2^-selectivity in an analogous fashion to conditions A, we searched for intermediates and TSs that included this possibility. No O–P or *N*^2^–P-coordinated intermediates were found. The coordinated intermediate **N1-P** was found considerably endergonic with a Δ*G* of +8.0 kcal/mol compared to **6(N-H)** (see Figure 7). A synchronous TS (**N1-P-TS**) leading to the *N*^2^-product was found starting from **N1-P**; however, the reaction barrier was 52.1 kcal/mol and thus a highly unlikely pathway. We again searched for TSs that led to both the *N*^1^*-* and *N*^2^*-*products but lacked any dative preorganization. However, under these reaction conditions, we found that the TS leading to the *N*^2^-product, **N2-*s-cis,*** was lower in energy than its *N*^1^-analog, **N1-*s-trans****,* by 1.1 kcal/mol (see Figure 8). This energy difference appears to be driven by stabilizing non-covalent interactions. Specifically, the carbonyl O in **N2-s-*cis*** shows NCIs with one of the benzene rings of PPh_3_ as well as a hydrogen bond-like NCI with a H-atom of the electrophilic methyl. Thus, the partitioning between transition states favor the *N*^2^-pathway over the *N*^1^-pathway by a product ratio of 4.5:1, which supports a pathway producing the observed experimental *N*^2^-product ratios with greater than 80% yield. The *N*^1^-product was again found to be lower in energy by 4.4 kcal/mol than the *N*^2^-product.

**Figure 9:**
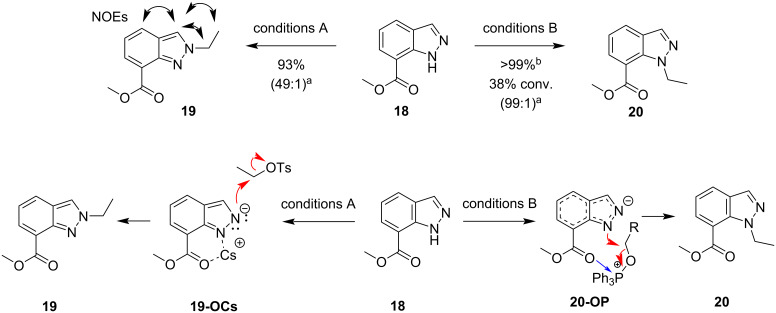
Reaction of **18** under conditions A and B (top), and proposed chelation/coordination pathways to account for regioselectivity (bottom); black two-headed curved arrows indicate the observed NOEs of the major product; ^a^determined by LC–MS; ^b^based on recovered starting material.

To further explore whether the reaction mechanisms followed the chelation pathway proposed in Figure 5, we hypothesized that **18** (Figure 9) would provide a model for exploring the mechanism further. If chelation between an electron-rich oxygen atom from a substituent and a Lewis acid (such as Cs^+^ or P^+^) were taking place, we would expect regioselectivity for this substrate to be reversed (**19-OCs, 20-OP)**, such that conditions A would produce the *N*^2^-product and conditions B would produce the *N*^1^-product. This was found to be the case as can be observed in Figure 9. The *N*^2^-product **19** was isolated in 93% yield under conditions A, and the *N*^1^-product **20** was isolated in >99% yield under conditions B albeit with low conversion. Both conditions provided >98:2 regioselectivity for their respective major products as determined by LC–MS.

**Figure 10:**
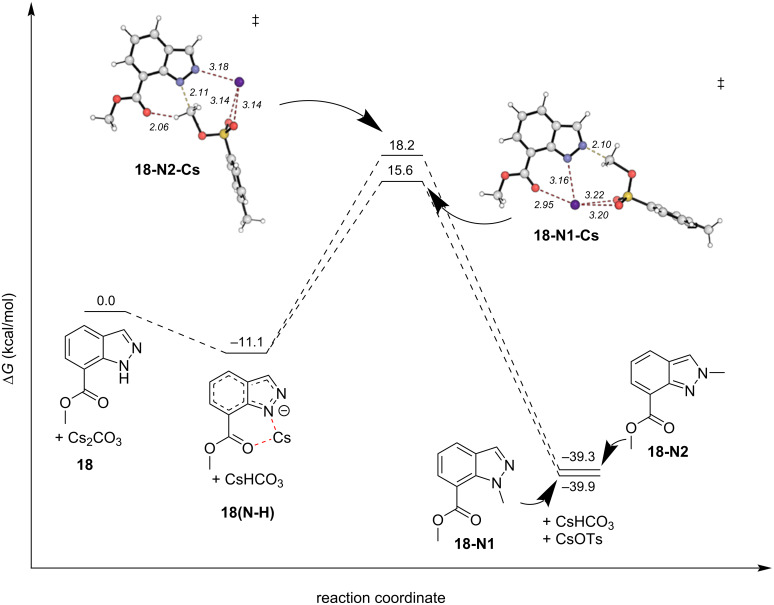
DFT-calculated reaction coordinate diagram for the reaction of **18** under conditions A.

**Figure 11:**
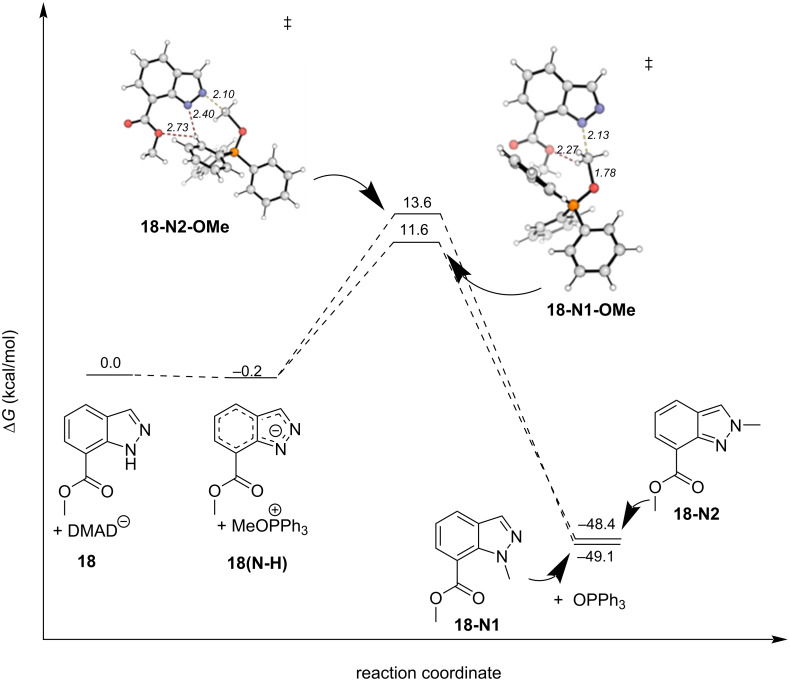
DFT-calculated reaction coordinate diagram for the reaction of **18** under conditions B. Ball-and-stick transition-state structures are provided for the lowest energy *N*^1^ and *N*^2^ transition states with favorable NCIs shown as red dashed lines.

We again turned to DFT calculations to explore the mechanisms of these reactions and found very similar results to the parent system (Figure 10). Under conditions A, deprotonation and cesium coordination were heavily favored by 11.1 kcal/mol (**18(N-H)**). Transition states **18-N2-Cs** and **18-N1-Cs** were found leading to *N*^1^- and *N*^2^-products, respectively. NCIs between the cesium cation with the ester and sulfonate oxygens, and critically *N*^1^, position the electrophilic methyl group 2.1 Å from *N*^2^, lowering the TS energy by ΔΔ*G*^‡^ = 2.6 kcal/mol in **18-N1-Cs**. Interestingly, the difference in product energies is quite small, only favoring the *N*^1^-product, **18-N1**, by 0.6 kcal/mol. Concerning conditions B*,* the difference between the neutral indazole and the deprotonated indazole was only −0.2 kcal/mol (Figure 11). Again, no preorganized intermediates were found. The NCIs were consistent with the parent system. The hydrogen bond between the H on the electrophilic methyl group and an ester oxygen was found in the transition state leading to the *N*^1^ product, was conserved (**18-N1-OMe**, Figure 11), in a total of 4 NCIs. The only relevant NCIs in **18-N2-OMe** were between an aryl hydrogen, *N*^1^ and an ester oxygen, the result of which is ΔΔ*G*^‡^ = 2.0 kcal/mol favoring the *N*^1^ transition state **18-N1-OMe** among the NCIs found.

**Scheme 3:**
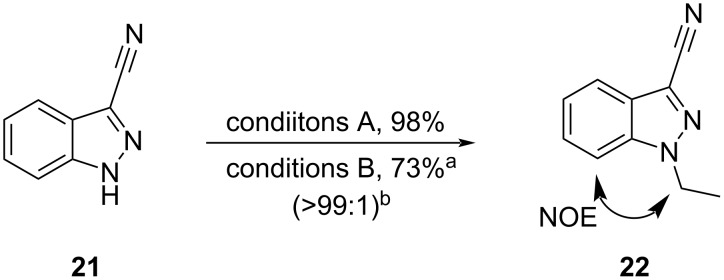
Reaction of **21** under conditions A and B; ^a^multiple purifications; ^b^determined by LC–MS.

To further probe whether the dominant discriminating factor was chelation or other NCIs, compound **21** was also subjected to the same reaction conditions (Scheme 3). As this cyano compound is not capable of forming an *N*^2^–Cs^+^–*N*_CN_ ion pair or dative bond, we were curious to observe product ratios. Compound **21** produced the *N*^1^-product **22** as the major regioisomer regardless of which conditions were employed. Though separation of products was challenging, resulting in a lower isolated yield under conditions B, the regioselectivity was greater than 99:1 under both conditions.

**Figure 12:**
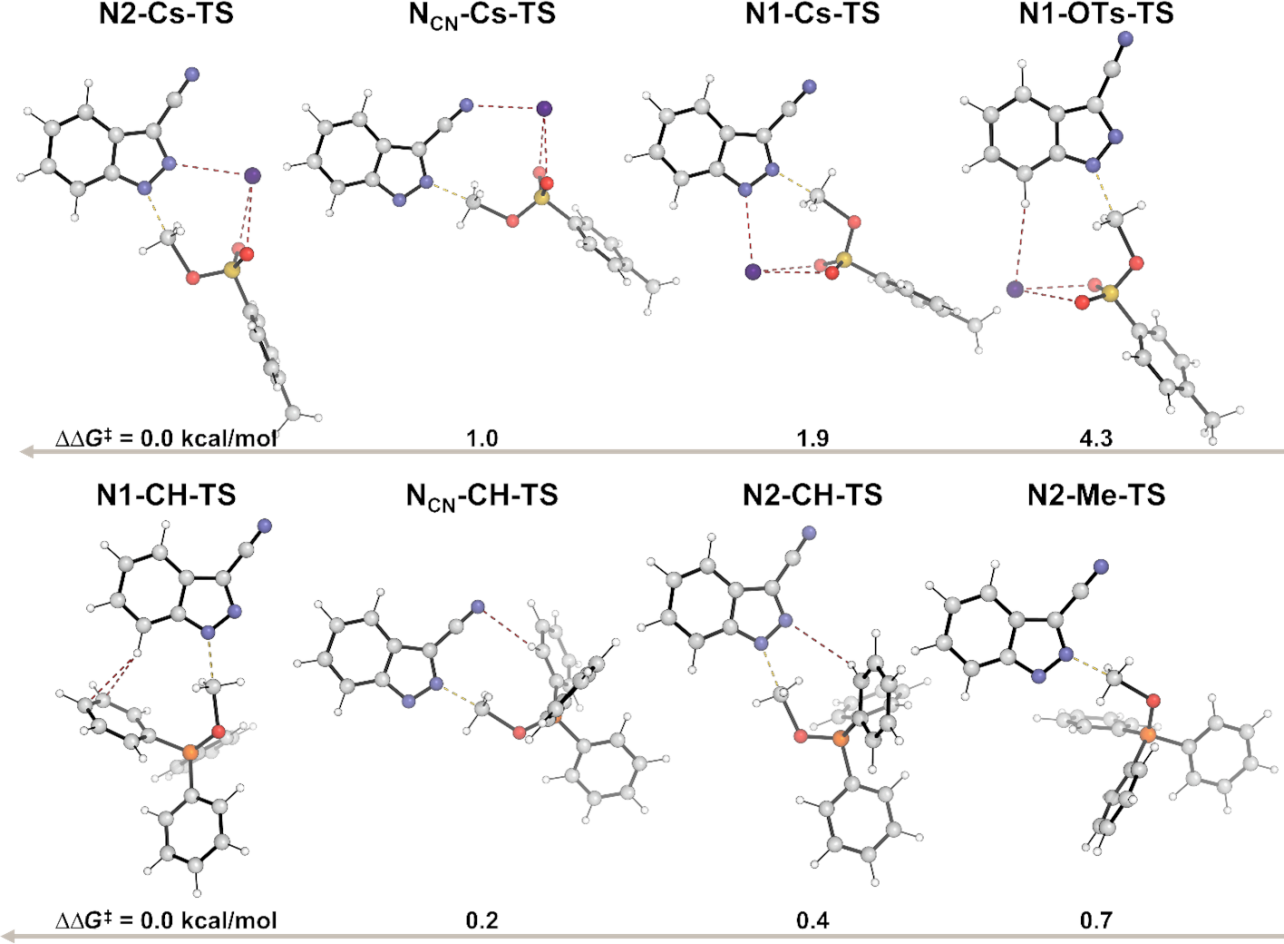
DFT-calculated transition-state structures and energies of **21** under conditions A (top) and conditions B (bottom).

DFT calculations again showed the deprotonated analog of **21** to be heavily favored in complex with Cs^+^ by 20.6 kcal/mol under conditions A, and 13.6 kcal/mol under conditions B. Transition states were found for the reaction of **21** under both conditions (see Supporting Information File 1). However, in this case, neither chelation nor other NCIs resulted in an energy difference between the lowest energy *N*^1^- and *N*^2^-transition states greater than 1 kcal/mol (Figure 12). Thus, we employed natural bond orbital (NBO) analysis to estimate the partial charge of *N*^1^ and *N*^2^ in both the neutral and deprotonated states using Gaussian 16: NBO7, *SMD(THF)-PBE0/def2-SVP*. Additionally, Parr and Yang developed a now widely used index of nucleophilicity from robust electron population methods such as NBO analyses, referred to as the Fukui index (*f*-) [44]. The larger the Fukui index, the greater the nucleophilicity, and is thus inversely proportional to the partial charge. Our calculations showed that *N*^1^ was more electronegative and had a larger Fukui index in both neutral and deprotonated states, not only in **21**, but in **18** and **6** also (Table 4). These data suggest that in the absence of an electron-withdrawing group responsible for either cation chelation or favorable NCI stabilization, nucleophilicity would dictate regioselectivity outcomes. This also implies that the favorable NCIs and chelation are stronger driving forces towards transition-state energy partitioning than nucleophilicity alone.

**Table 4:** DFT-calculated electronegativities and Fukui indices.

Compound	Partial charge		Fukui index (*f-*)	
	*N* ^1^	*N* ^2^	*N* ^1^	*N* ^2^

**6**	−0.33525	−0.24443	0.61445	0.52779
**6(N-H)**	−0.38846	−0.29832	0.65078	0.56750
**18**	−0.36252	−0.30508	0.15932	0.03122
**18(N-H)**	−0.42226	−0.36313	0.24944	0.00431
**21**	−0.33075	−0.23976	0.06065	0.16123
**21(N-H)**	−0.37927	−0.30008	0.20158	0.01133

These results suggest chelation is a highly plausible driving force for regioselectivity in the alkylation of methyl indazole-3- or -7-carboxylates. When the ester substituent is placed at the 3- or 7-position, the chelation of Cs^+^ or NCIs with ROPPh_3_^+^ and the associated nitrogens will drive regioselectivity to or away from that nitrogen, leading to excellent selectivity. These data support the claim made by Alam and Keeting that a tight ion pair drives *N*^1^-selectivity when electron-withdrawing groups that can coordinate the cation are present at the 3-position. When 3-cyanoindazole is employed and no bidentate coordination is possible with *N*^2^, the nucleophilicity of *N*^1^ drives the regioselectivity. Additionally, these data show the importance of NCIs in understanding mechanisms where regioselectivity outcomes are unexpected. Lastly, it should be noted that these reactions are likely irreversible due to the ≈50–60 kcal/mol barriers of the reverse reactions and near-absent nucleophilic character of TsO^−^ and triphenylphosphine oxide, precluding any thermodynamic versus kinetic arguments for regioselectivity.

## Conclusion

We have established highly regioselective *N*^1^*-* and *N*^2^-alkylations of methyl 5-bromo-1*H*-indazole-3-carboxylate from diverse commercially available alcohols with excellent yields (>84%). The unique quality of this work is the production of working mechanisms that support the observed regioisomeric ratios. This work presents the first comprehensive DFT mechanistic study on these systems which differentiate formation of *either N*^1^- *or N*^2^-substituted indazoles in excellent yields *from the same carbon sources* through reagent control.

## Experimental

### General methods

All materials were obtained from commercial suppliers and used without further purification unless otherwise noted. Anhydrous solvents were obtained from Sigma-Aldrich and used directly. Reactions involving air- or moisture-sensitive reagents were performed under a nitrogen or argon atmosphere. Silica gel chromatography was performed using prepacked silica gel columns (RediSep^®^ Rf, Teledyne ISCO). An aluminum block atop a hotplate with a thermocouple was used to heat reactions to the specified temperatures. NMR spectra were acquired on Bruker 300 MHz spectrometers equipped with 5 mm BBFO probes. HRMS data were acquired using an Agilent 6530 LC/Q-TOF using a Dual AJS/ESI ion source, and the isotope 79 was used for HRMS analysis for any bromine-containing compounds.

### Synthesis

All procedures and spectra can be found in Supporting Information File 1.

### General procedure for the *N*^1^-alkylation using alkyltosylates

#### Preparation of methyl 5-bromo-1-methy-1*H*-indazole-3-carboxylate (**15a**)

To a solution of methyl 5-bromo-1*H*-indazole-3-carboxylate (300 mg, 1.176 mmol) in dioxane (10 mL) at room temperature was added cesium carbonate (766 mg, 2.352 mmol) followed by the necessary tosylate (1.5 equiv). The resulting mixture was stirred for 2 hours at 90 °C. The mixture was poured into EtOAc (500 mL) and washed with water (100 mL) and brine. The organic layer was dried and concentrated, the obtained residue was purified by chromatography (silica [24 g], eluting with EtOAc in hexane from 0–70%) to give methyl 5-bromo-1-alkyl-1*H*-indazole-3-carboxylate, in 90–98%. For **15a**, 284.5 mg, 90%, as a white solid. Mp 141.3 °C; ^1^H NMR (300 MHz, DMSO-*d*_6_) δ 8.19 (dd, *J* = 1.9, 0.7 Hz, 1H), 7.82 (dd, *J* = 9.0, 0.7 Hz, 1H), 7.65 (dd, *J* = 8.9, 1.9 Hz, 1H), 4.17 (s, 3H), 3.92 (s, 3H); ^13^C{1H} NMR (75 MHz, DMSO-*d*_6_) δ 161.8, 139.4, 132.7, 129.4, 124.1, 123.0, 116.0, 113.0, 51.8, 36.6; IR (KBr disk): 1722, 1466, 1433, 1395, 1354, 1289, 1200, 1183, 1153 cm^−1^; HRESIMS (*m*/*z*): [M + H]^+^ calcd for C_10_H_10_BrN_2_O_2_^+^, 268.9921; found, 268.9902.

### General procedure for the *N*^2^-alkylation using Mitsunobu conditions

#### Preparation of methyl 5-bromo-2-methyl-2*H*-indazole-3-carboxylate (**16a**)

To a solution of methyl 5-bromo-1*H*-indazole-3-carboxylate (1.384 g, 5.43 mmol) in THF (15 mL) was added triphenylphosphine (2.85 g, 10.85 mmol) and methanol (0.5 mL, 12.4 mmol) at 0 °C, followed by adding DEAD (1.718 mL, 10.85 mmol). The resulting mixture was stirred for 10 min at 0 °C, warmed to 50 °C, and stirred for 2 h. After TLC showed completion, the solvent was removed, and the residue was purified by chromatography (silica [24 g], eluting with ethyl acetate in hexane from 0–60%) to give methyl 5-bromo-2-alkyl-2*H*-indazole-3-carboxylate in 90–97% yield. For **16a**, 291 mg, 92%, as a light pink solid. Mp 110.7 °C; ^1^H NMR (300 MHz, DMSO-*d*_6_) δ 8.14 (d, *J* = 2.0 Hz, 1H), 7.77 (dd, *J* = 9.1, 0.7 Hz, 1H), 7.49 (dd, *J* = 9.0, 1.9 Hz, 1H), 4.42 (s, 3H), 3.98 (s, 3H); ^13^C{1H} NMR (75 MHz, DMSO-*d*_6_) δ 159.4, 144.7, 129.3, 123.6, 123.2, 122.8, 120.0, 118.0, 64.2, 52.2, 41.4, 14.4, 13.9; IR (KBr disk): 1708, 1459, 1442, 1392, 1326, 1252, 1196 cm^−1^; HRESIMS (*m*/*z*): [M + H]^+^ calcd for C_10_H_10_BrN_2_O_2_^+^, 268.9921; found, 268.9918.

## Supporting Information

File 1 Characterization of all compounds (^1^H NMR, ^13^C NMR, LC–MS, IR), and crystallographic methods and data for products **P1** and **P2**.

File 2 DFT methods, relative energy comparisons, TS imaginary frequencies, and XYZ coordinates.

File 3 GoodVibes outputs.

## Data Availability

All data that supports the findings of this study is available in the published article and/or the supporting information to this article.
