# Supplementary material for: Regioselective alkylation of a versatile indazole: Electrophile scope and mechanistic insights from density functional theory calculations
Source: Beilstein J Org Chem. 2024 Aug 9;20:1940–54. doi: 10.3762/bjoc.20.170 (PMC11318628; doi:10.3762/bjoc.20.170)
Supplement: File 1 — Characterization of all compounds (1H NMR, 13C NMR, LC–MS, IR), and crystallographic methods and data for products P1 and P2. [file Beilstein_J_Org_Chem-20-1940-s001.pdf]

BEILSTEIN JOURNAL OF ORGANIC CHEMISTRY

Supporting Information

for

Regioselective alkylation of a versatile indazole: Electrophile scope and mechanistic insights from density functional theory calculations

Pengcheng Lu, Luis Juarez, Paul A. Wiget, Weihe Zhang, Krishnan Raman and Pravin L. Kotian

Beilstein J. Org. Chem. **2024,** *20,* 1940–1954. doi:10.3762/bjoc.20.170

Characterization of all compounds (^{1}H NMR, ^{13}C NMR, LC–MS, IR), and crystallographic methods and data for products P1 and P2

License and Terms: This is a supporting information file under the terms of the Creative Commons Attribution License (https://creativecommons.org/licenses/by/4.0). Please note that the reuse, redistribution and reproduction in particular requires that the author(s) and source are credited and that individual graphics may be subject to special legal provisions.
The license is subject to the *Beilstein Journal of Organic Chemistry* terms and conditions: (https://www.beilstein-journals.org/bjoc/terms)

Table of contents

Scheme S1 Procedure S3
Table S1. Conditions screening. S3
Figure S1. 1D NOE of **8**. S5
Figure S2. NMR comparison of **8** and **9**. S6
Synthesis of compounds **12**, **14a**, **c–l**. S6
Preparation of ethyl 4-methylbenzenesulfonate (**12**). S6
Preparation of propyl 4-methylbenzenesulfonate (**14d**). S7
Preparation of (*R*)-sec-butyl 4-methylbenzenesulfonate (**14e**). S8
Preparation of isobutyl 4-methylbenzenesulfonate (**14f**). S8
Preparation of 2-methoxyethyl 4-methylbenzenesulfonate (**14g**). S9
Preparation of cyclopentyl 4-methylbenzenesulfonate (**14h**). S10
Preparation of *tert*-butyl 3-(tosyloxy)pyrrolidine-1-carboxylate (**14i**). S10
Preparation of (*R*)-tetrahydrofuran-3-yl 4-methylbenzenesulfonate (**14j**). 11
Preparation of (*S*)-tetrahydrofuran-3-yl 4-methylbenzenesulfonate (**14k**). S12
Preparation of (*S*)-tetrahydrofuran-3-yl 4-methylbenzenesulfonate (**14l**). S12
Preparation of *tert*-butyl 3-(tosyloxy)azetidine-1-carboxylate (**14m**). S13
Preparation of cyclopentylmethyl 4-methylbenzenesulfonate (**14n**). S14
Preparation of (tetrahydrofuran-3-yl)methyl 4-methylbenzenesulfonate (**14o**). S14
Preparation of cyclohexylmethyl 4-methylbenzenesulfonate (**14p**). S15
Figure S3. 2D NOE of **6**. Circled in-phase correlation is between CH3 and NH. S16
Preparation of methyl 5-bromo-1-methy-1*H*-indazole-3-carboxylate (**15a**) S17
Preparation of methyl 5-bromo-1-ethyl-1*H*-indazole-3-carboxylate (**15b**). S17
Preparation of methyl 5-bromo-1-propyl-1*H*-indazole-3-carboxylate (**15c**). S18
Preparation of methyl 5-bromo-1-propyl-1*H*-indazole-3-carboxylate (**15d**) S18
Preparation of methyl (*S*)-5-bromo-1-(sec-butyl)-1*H*-indazole-3-carboxylate (**15e**). S19
Preparation of methyl 5-bromo-1-isobutyl-1*H*-indazole-3-carboxylate (**15f**) S20
Preparation of methyl 5-bromo-1-(2-methoxyethyl)-1*H*-indazole-3-carboxylate (**15g**) S20
Preparation of methyl 5-bromo-1-cyclopentyl-1*H*-indazole-3-carboxylate (**15h**). S21
Preparation of methyl 5-bromo-1-(1-(*tert*-butoxycarbonyl)pyrrolidin-3-yl)-1*H*-indazole-3-carboxylate (**15i**). S22
Preparation of methyl (*S*)-5-bromo-1-(tetrahydrofuran-3-yl)-1*H*-indazole-3-carboxylate (**15j**). S22
Preparation of methyl (*R*)-5-bromo-1-(tetrahydrofuran-3-yl)-1*H*-indazole-3-carboxylate (**15k**). S23
Preparation of methyl 5-bromo-1-(cyclobutylmethyl)-1*H*-indazole-3-carboxylate (**15l**). S24
Preparation of methyl 5-bromo-1-((1-(*tert*-butoxycarbonyl)azetidin-3-yl)methyl)-1*H*-indazole-3-carboxylate (**15m**). No reaction. S24
Preparation of methyl 5-bromo-1-(cyclopentylmethyl)-1*H*-indazole-3-carboxylate (**15n**). S25
Preparation of methyl 5-bromo-1-((tetrahydrofuran-2-yl)methyl)-1*H*-indazole-3-carboxylate (**15o**). S25
Preparation of methyl 5-bromo-1-(cyclohexylmethyl)-1*H*-indazole-3-carboxylate (**15p**). S26
Preparation of methyl 1-benzyl-5-bromo-1*H*-indazole-3-carboxylate (**15q**). S27
Preparation of methyl 5-bromo-2-methyl-2*H*-indazole-3-carboxylate (**16a**). S27
Preparation of methyl 5-bromo-2-ethyl-2*H*-indazole-3-carboxylate (**16b**). S28
Preparation of methyl 5-bromo-2-isopropyl-2*H*-indazole-3-carboxylate (**16c**). S29
Preparation of methyl 5-bromo-2-propyl-2*H*-indazole-3-carboxylate (**16d**). S29
Preparation of methyl (*S*)-5-bromo-2-(sec-butyl)-2*H*-indazole-3-carboxylate (**16e**). S30
Preparation of methyl 5-bromo-2-isobutyl-2*H*-indazole-3-carboxylate (**16f**). S30
Preparation of methyl 5-bromo-2-(2-methoxyethyl)-2*H*-indazole-3-carboxylate (**16g**). S31

Preparation of methyl 5-bromo-2-cyclopentyl-2*H*-indazole-3-carboxylate (**16h**).. S32
Preparation of methyl 5-bromo-2-(1-(*tert*-butoxycarbonyl)pyrrolidin-3-yl)-2*H*-indazole-3-carboxylate (**16i**)............... S32
Preparation of methyl (*S*)-5-bromo-2-(tetrahydrofuran-3-yl)-2*H*-indazole-3-carboxylate (**16j**)..................................... S33
Preparation of methyl (*R*)-5-bromo-2-(tetrahydrofuran-3-yl)-2*H*-indazole-3-carboxylate (**16k**). S34
Preparation of methyl 5-bromo-2-(cyclobutylmethyl)-2*H*-indazole-3-carboxylate (**16l**). S34
Preparation of methyl 5-bromo-2-(1-(*tert*-butoxycarbonyl)azetidin-3-yl)-2*H*-indazole-3-carboxylate (**16m**)................ S35
Preparation of methyl 5-bromo-2-(cyclopentylmethyl)-2*H*-indazole-3-carboxylate (**16n**). S35
Preparation of methyl 5-bromo-2-((tetrahydrofuran-3-yl)methyl)-2*H*-indazole-3-carboxylate (**16o**)............................. S36
Preparation of methyl 5-bromo-2-(cyclohexylmethyl)-2*H*-indazole-3-carboxylate (**16p**).. S37
Preparation of methyl 2-benzyl-5-bromo-2*H*-indazole-3-carboxylate (**16q**). S37
Preparation of methyl 2-ethyl-2*H*-indazole-7-carboxylate (**19**). S38
Preparation of methyl 1-ethyl-1*H*-indazole-7-carboxylate (**20**). S38
Preparation of 1-ethyl-1*H*-indazole-3-carbonitrile (**22**), method A. S39
Preparation of 1-ethyl-1*H*-indazole-3-carbonitrile (**22**), method B. S39
Crude LCMS data. Condition A (top), Condition B (bottom). N^1 Product has shorter Rt for a-q. S41
NMR Spectra of synthesized compounds. S75
Compound **8** crystal data. S173
Compound **9** crystal data. S175
Compound **16m** crystal data. S177
IR Spectra of all compounds S179

Scheme S1 Procedure.

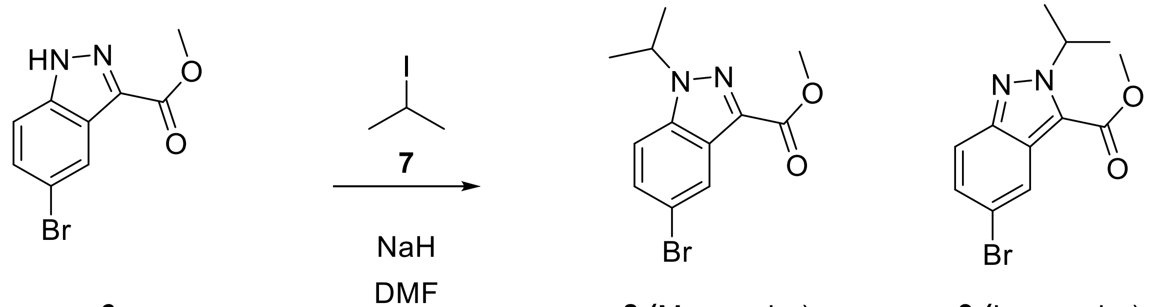

6 8 (More polar) 9 (Less polar)

To a solution of methyl 5-bromo-1*H*-indazole-3-carboxylate (**6,** 1 g, 3.92 mmol) in DMF (Volume: 10 mL) at 0 °C was treated with sodium hydride (0.172 g, 60% dispersion, 4.31 mmol). After 15 min, 2-iodopropane (1.187 mL, 11.76 mmol) was added at 0 °C and the reaction was warmed slowly in the ice bath to RT and stirred for 15 h. The mixture was poured into EtOAc and washed with water and brine. The organic layer was dried and concentrated, and the obtained residue was purified by chromatography [silica gel (40 g), eluting with EtOAc in hexane from 0–50%] to give methyl 5-bromo-1-isopropyl-1*H*-indazole-3-carboxylate (**8**, 0.45 g, 1.514 mmol, 38.6% yield) as a white solid and methyl 5-bromo-2-isopropyl-2*H*-indazole-3-carboxylate (**9**, 0.54 g, 1.817 mmol, 46.4% yield) as a white solid. Characterization is consistent with **15c** and **16c** (vide infra).

Table S1. Conditions screening.

To a solution of methyl 5-bromo-1*H*-indazole-3-carboxylate (0.500 g, 1.960 mmol) in [solvent] (20 mL) at RT was added [base, equiv] followed by ethyl 4-methylbenzenesulfonate (**12,** equiv). The resulting mixture was stirred for [#] hours at [#] °C. The mixture was poured into EtOAc 500 mL and washed with water 100 mL and brine. The organic layer was dried and concentrated, the obtained residue was purified by chromatography [silica gel (24 g), eluting with EtOAc in hexane from 0–70%] to give ## P1 and ## P2 as white solids. Characterization is consistent with **15b** and **16b** (vide infra).

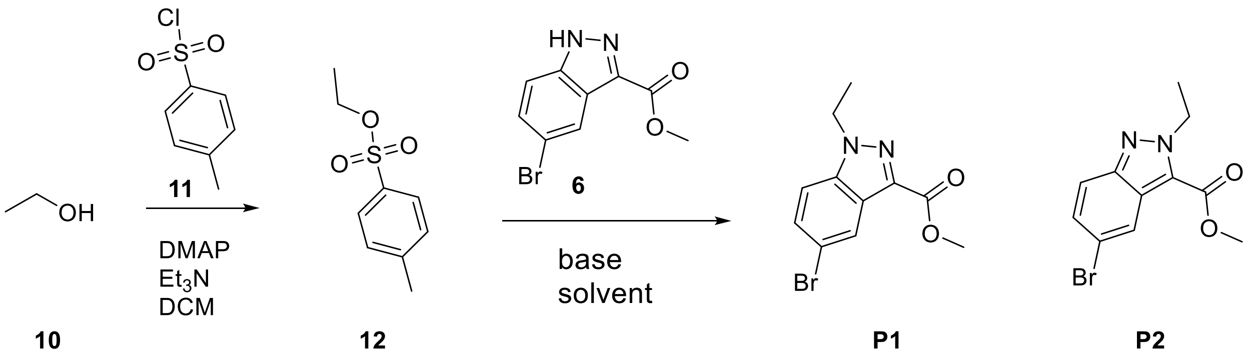

Entry	Eq. of 12	Eq. of 6	Base (eq.)	Solvent	Temperature (°C)	Time (h)	P1 % (Isolated)
1	1.0	1.0	Cs_2CO_3 (2.0)	DMF	90	2	52
2	1.1	1.0	Cs_2CO_3 (2.0)	DMF	90	2	54
3	1.2	1.0	Cs_2CO_3 (2.0)	DMF	90	2	54
4	1.3	1.0	Cs_2CO_3 (2.0)	DMF	90	2	54
5	1.4	1.0	Cs_2CO_3 (2.0)	DMF	90	2	55
6	1.5	1.0	Cs_2CO_3 (2.0)	DMF	90	2	60
7	2.0	1.0	Cs_2CO_3 (2.0)	DMF	90	2	54
8	2.5	1.0	Cs_2CO_3 (2.0)	DMF	90	2	53
9	3.0	1.0	Cs_2CO_3 (2.0)	DMF	90	2	54
10	1.5	1.0	Cs_2CO_3 (2.0)	DMF	110	2	48
11	1.5	1.0	Cs_2CO_3 (2.0)	DMF	100	2	51
12	1.5	1.0	Cs_2CO_3 (2.0)	DMF	80	2	51
13	1.5	1.0	Cs_2CO_3 (2.0)	DMF	70	2	54
14	1.5	1.0	Cs_2CO_3 (2.0)	DMF	60	2	49
15	1.5	1.0	Cs_2CO_3 (2.0)	DMF	50	2	52
16	1.5	1.0	Cs_2CO_3 (2.0)	DMF	40	2	45
17	1.5	1.0	Cs_2CO_3 (1.0)	DMF	RT	24	49
18	1.5	1.0	Cs_2CO_3 (1.0)	DMF	90	2	54

19	1.5	1.0	Cs_2CO_3 (1.5)	DMF	90	2	54
20	1.5	1.0	Cs_2CO_3 (2.5)	DMF	90	2	53
21	1.5	1.0	Cs_2CO_3 (3.0)	DMF	90	2	51
22	1.5	1.0	Cs_2CO_3 (4.0)	DMF	90	2	53
23	1.5	1.0	NaH (1.0)	DMF	25	24	31
24	1.5	1.0	NaH (2.0)	DMF	25	24	35
25	1.5	1.0	NaH (3.0)	DMF	25	24	35

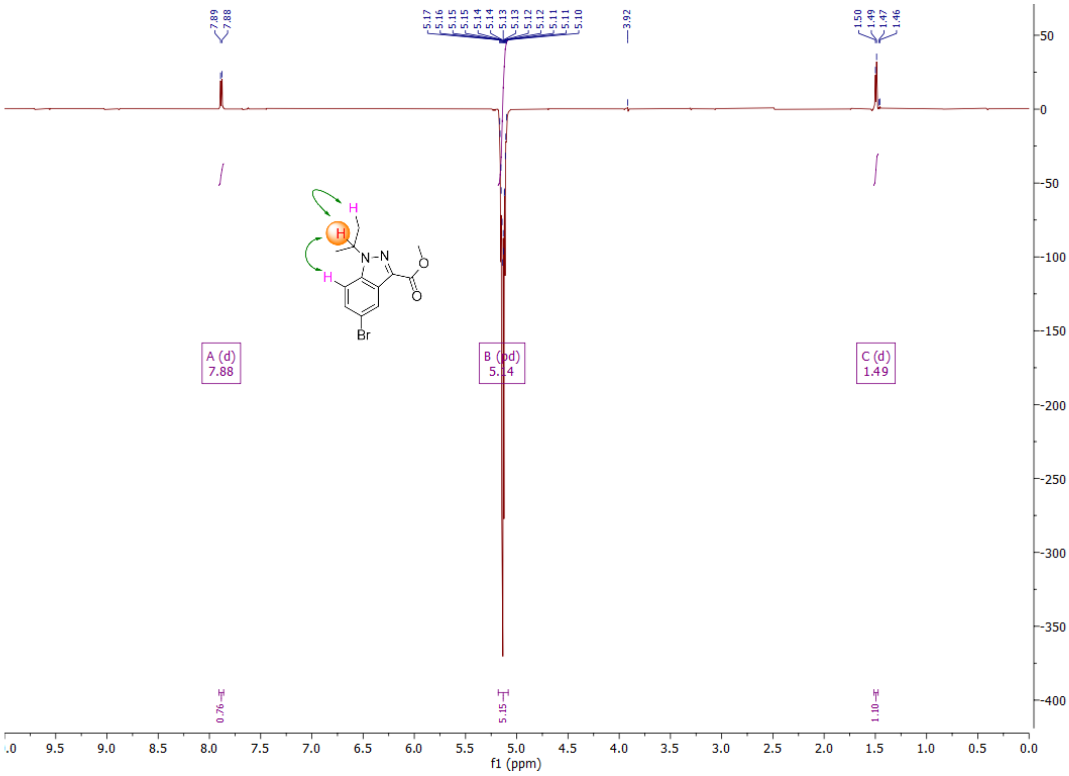

Figure S1. 1D NOE of 8.

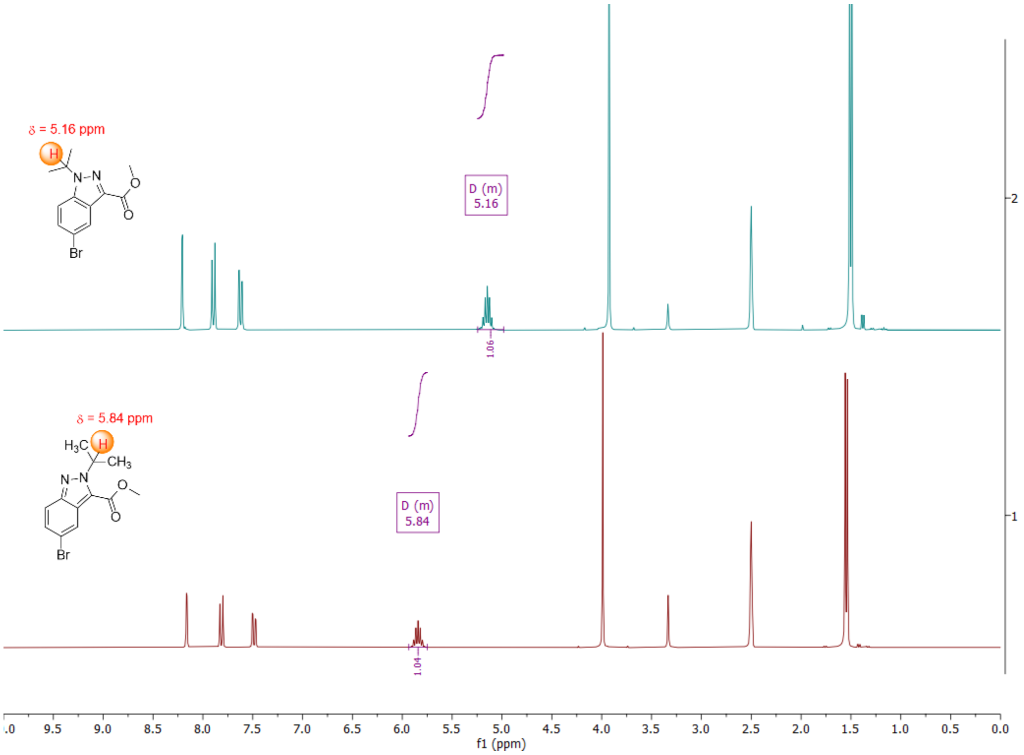

Figure S2. NMR comparison of 8 and 9.

Synthesis of compounds 12, 14a, c–l.

Alkyl sulfonates **12** and **14d–p** were prepared according to the procedure described by Marco, J. L. ***J. Heterocyclic Chem*., 23,** 1059 **(1986)**. Our specific procedures and characterization are provided below. Alkyl sulfonates **14a**, **c**, and **q** were purchased from Sigma-Aldrich.

Preparation of ethyl 4-methylbenzenesulfonate (12).

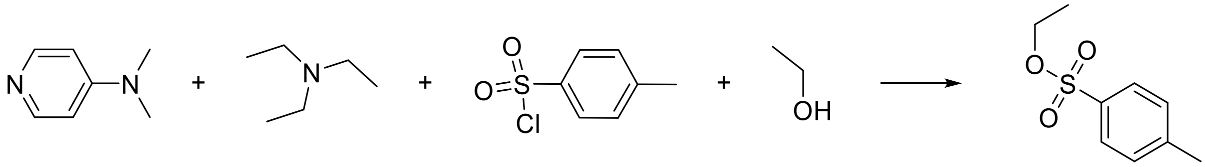

To a mixture of ethanol (10 mL, 171 mmol), DMAP (1.046 g, 8.56 mmol) and TEA (35.8 mL, 257 mmol) in anhydrous DCM (450 mL) was added *p*-toluenesulfonyl chloride (35.9 g, 188 mmol) at 0 °C. The mixture was stirred briskly at 20 °C for 16 h and later diluted with brine (20 mL) and water (20 mL). The organic layer was separated, washed with brine (20 mL), dried over $MgSO_4$ and evaporated on a rotary evaporator. The residue obtained was purified by flash column chromatography [silica gel (80 g), eluting with EtOAc in hexane from 0–

100%] to give ethyl 4-methylbenzenesulfonate (23.4 g, 117 mmol, 68.2% yield) as a clear oil; ^{1}H NMR (300 MHz, DMSO-*d*$_6$) δ 7.91 – 7.81 (m, 2H), 7.61 – 7.51 (m, 2H), 4.14 (q, *J* = 7.1 Hz, 2H), 2.50 (s, 3H), 1.26 (t, *J* = 7.1 Hz, 3H); ^{13}C{H} NMR (75 MHz, DMSO-*d*$_6$) δ 144.8, 132.7, 130.2, 127.5, 67.3, 21.1, 14.5; IR (KBr disk) 3025, 1354, 1189, 1176, 1004, 915, 8616, 804, 778, 663, 555 cm^{-1}; HRMS (ESI) m/z: [M + H_2O]$^+$ calculated for $C_9H_{14}O_4S$ 218.0613, found 218.0808.

Preparation of propyl 4-methylbenzenesulfonate (14d).

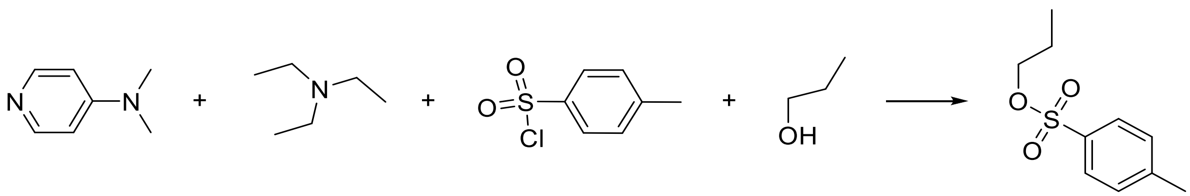

To a mixture of 4-methylbenzenesulfonyl chloride (3.49 g, 18.30 mmol), *N*,*N*-dimethylpyridin-4-amine (0.102 g, 0.832 mmol) and propan-1-ol (1 g, 16.64 mmol) in anhydrous DCM (10 mL) was added 4-methylbenzenesulfonyl chloride (3.49 g, 18.30 mmol) at 0 °C. The mixture was stirred briskly at 20 °C for 16 h and later diluted with brine (50 mL) and water (50 mL). The organic layer was separated, washed with brine (50 mL), dried over $MgSO_4$ and evaporated on a rotary evaporator. The residue obtained was purified by flash column chromatography [silica gel (40 g), eluting with EtOAc in hexane from 0–100%] to give propyl 4-methylbenzenesulfonate (3.2 g, 14.93 mmol, 90% yield) as a clear oil; ^{1}H NMR (300 MHz, DMSO-*d*$_6$) δ 7. 7.78 (d, J = 8.4 Hz, 2H), 7.48 (d, J = 8.0 Hz, 2H), 3.97 (t, J = 6.4 Hz, 2H), 2.42 (s, 3H), 1.56 (h, J = 7.4 Hz, 2H), 0.80 (t, J = 7.4 Hz, 3H); ^{13}C{H} NMR (75 MHz, DMSO-*d*$_6$) δ 144.8, 132.6, 130.1, 127.5, 72.4, 21.8, 21.1, 9.7; HRMS (ESI+) m/z: calculated for $C_{10}H_{14}NaO_3S^+$ 237.0561, ion source failed; LCMS mass found: 237.10.

Preparation of (*R*)-sec-butyl 4-methylbenzenesulfonate (14e).

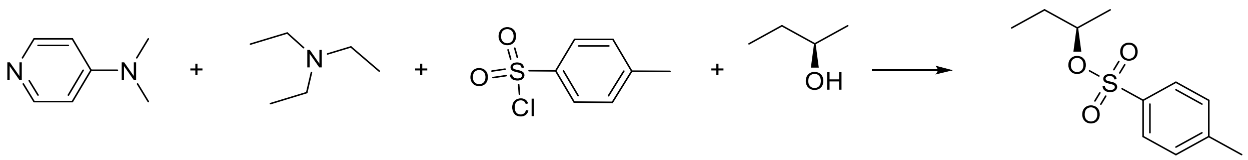

To a mixture of (*R*)-butan-2-ol (1 g, 13.49 mmol), *N*,*N*-dimethylpyridin-4-amine (0.082 g, 0.675 mmol) and triethylamine (2.82 mL, 20.24 mmol) in anhydrous DCM (20 mL) was added 4-methylbenzenesulfonyl chloride (2.83 g, 14.84 mmol)at 0 °C. The mixture was stirred briskly at 20 °C for 16 h and later diluted with brine (50 mL) and water (50 mL). The organic layer was separated, washed with brine (50 mL), dried over $MgSO_4$ and evaporated on a rotary evaporator. The residue obtained was purified by flash column chromatography [silica gel (40 g), eluting with EtOAc in hexane from 0–100%] to give (*R*)-sec-butyl 4-methylbenzenesulfonate (2.98 g, 13.05 mmol, 97% yield) as a clear oil; ^{1}H NMR (300 MHz, DMSO-d_6) δ 7.83 (d, J = 8.4 Hz, 2H), 7.48 (d, J = 8.3 Hz, 2H), 4.93 (s, 1H), 4.74 – 4.46 (m, 1H), 1.71 – 1.47 (m, 2H), 1.25 (dd, J = 6.2, 2.2 Hz, 3H), 0.83 (t, J = 7.4 Hz, 3H); ^{13}C{H} NMR (75 MHz, DMSO-d_6) δ 144.6, 133.8, 130.1, 127.4, 81.8, 28.7, 21.1, 20.0, 9.0; IR (KBr disk) 2979, 2939, 1599, 1463, 1382, 1362, 1306, 1189, 1176, 1110, 1094, 1020 cm^{-1}; HRMS (ESI) m/z: [M + Na]$^+$ calculated for $C_{11}H_{20}NO_3S^+$ 246.1158 found 246.1162.

Preparation of isobutyl 4-methylbenzenesulfonate (14f).

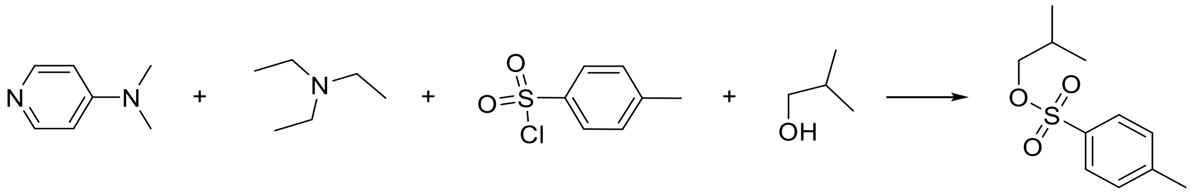

To a mixture of 2-methylpropan-1-ol (1 g, 13.49 mmol), *N*,*N*-dimethylpyridin-4-amine (0.082 g, 0.675 mmol) and triethylamine (2.82 mL, 20.24 mmol) in anhydrous DCM (10 mL) was added 4-methylbenzenesulfonyl chloride (2.83 g, 14.84 mmol) at 0 °C. The mixture was stirred briskly at 20 °C for 16 h and later diluted with brine (50 mL) and water (50 mL). The organic layer was separated, washed with brine (50 mL), dried over $MgSO_4$

and evaporated on a rotary evaporator. The residue obtained was purified by flash column chromatography [silica gel (40 g), eluting with EtOAc in hexane from 0–100%] to give isobutyl 4-methylbenzenesulfonate (2.66 g, 11.65 mmol, 86% yield) as a clear oil; ^{1}H NMR (300 MHz, DMSO-d_6) δ 6.97 (d, J = 8.3 Hz, 2H), 6.64 (d, J = 8.3 Hz, 2H), 2.98 (d, J = 6.3 Hz, 2H), 1.65 (s, 3H), 1.07 (hept, J = 13.2, 6.6 Hz, 1H), 0.06 (d, J = 6.8 Hz, 6H); ^{13}C{H} NMR (75 MHz, DMSO-d_6) δ 144.8, 132.5, 130.1, 127.5, 76.2, 27.4, 21.0, 18.2; IR (KBr disk) 2967, 1470, 1395, 1360, 1189, 1175, 1097 cm^{-1}; HRMS (ESI) m/z: $[M + H_2O]^+$ calculated for $C_{11}H_{18}O_4S^+$ 246.0926, found 246.1139.

Preparation of 2-methoxyethyl 4-methylbenzenesulfonate (14g).

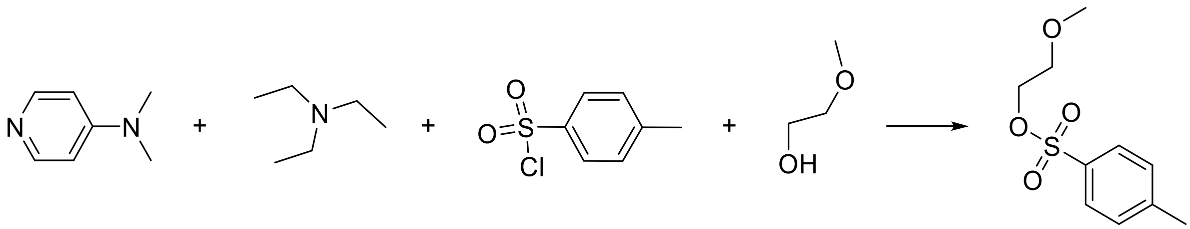

To a mixture of 2-methoxyethan-1-ol (1 g, 13.14 mmol), *N*,*N*-dimethylpyridin-4-amine (0.080 g, 0.657 mmol) and triethylamine (2.75 mL, 19.71 mmol) in anhydrous DCM (20 mL) was added 4-methylbenzenesulfonyl chloride (2.76 g, 14.46 mmol) at 0 °C. The mixture was stirred briskly at 20 °C for 16 h and later diluted with brine (50 mL) and water (50 mL). The organic layer was separated, washed with brine (50 mL), dried over $MgSO_4$ and evaporated on a rotary evaporator. The residue obtained was purified by flash column chromatography [silica gel (40 g), eluting with EtOAc in hexane from 0–100%] to give 2-methoxyethyl 4-methylbenzenesulfonate (2.62 g, 11.38 mmol, 87% yield) as a clear oil. ^{1}H NMR (300 MHz, DMSO-d_6) δ 7.83 (d, J = 8.5 Hz, 2H), 7.49 (d, J = 8.3 Hz, 2H), 4.22 – 4.11 (m, 2H), 3.58 (dd, J = 5.1, 3.8 Hz, 2H), 3.35 (s, 3H), 3.31 (s, 3H); ^{13}C{H} NMR (75 MHz, DMSO-d_6) δ 144.9, 132.4, 130.1, 127.6, 69.7, 69.3, 57.9, 21.1, 21.1; IR (KBr disk) 1598, 1451, 1356, 1189, 1176, 1131, 1097, 1019 cm^{-1}; HRMS (ESI) m/z: $[M + H_2O]^+$ calculated for $C_{10}H_{16}O_5S^+$ 248.0718, found 248.0940.

Preparation of cyclopentyl 4-methylbenzenesulfonate (14h).

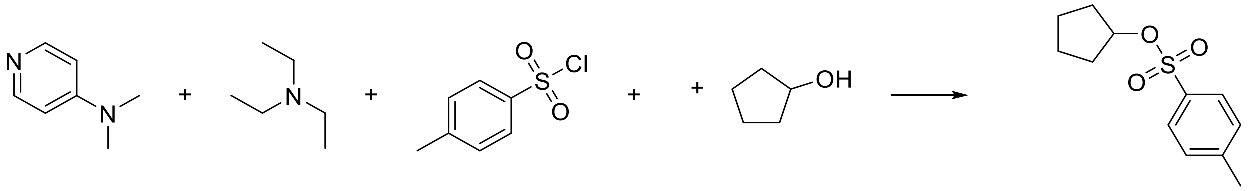

To a mixture of 4-methylbenzenesulfonyl chloride (2.435 g, 12.77 mmol), *N*,*N*-dimethylpyridin-4-amine (0.071 g, 0.580 mmol) and triethylamine (2.427 mL, 17.41 mmol) in anhydrous DCM (20 mL) was 4-methylbenzenesulfonyl chloride (2.435 g, 12.77 mmol) at 0 °C. The mixture was stirred briskly at 20 °C for 16 h and later diluted with brine (50 mL) and water (50 mL). The organic layer was separated, washed with brine (50 mL), dried over MgSO4 and evaporated on a rotary evaporator. The residue obtained was purified by flash column chromatography [silica gel (40 g), eluting with EtOAc in hexane from 0–100%] to give cyclopentyl 4-methylbenzenesulfonate (1.54 g, 6.41 mmol, 55.2% yield) as a clear oil; ^{1}H NMR (300 MHz, DMSO-d_6) δ 7.82 (dq, J = 8.5, 1.8 Hz, 2H), 7.48 (d, J = 7.9 Hz, 2H), 4.98 (q, J = 3.8 Hz, 1H), 4.94 (d, J = 3.3 Hz, 2H), 1.85 – 1.67 (m, 7H), 1.62 (s, 2H); ^{13}C{H} NMR (75 MHz, DMSO-d_6) δ 144.7, 133.8, 130.1, 127.4, 85.6, 32.5, 22.6, 21.1; IR (KBr disk) ~3000, ~1300, 1163 cm^{-1}; HRMS (ESI) m/z: $[M + NH_4]^+$ calculated for $C_{12}H_{20}NO_3S^+$ 258.1158, found 258.1164.

Preparation of *tert*-butyl 3-(tosyloxy)pyrrolidine-1-carboxylate (14i).

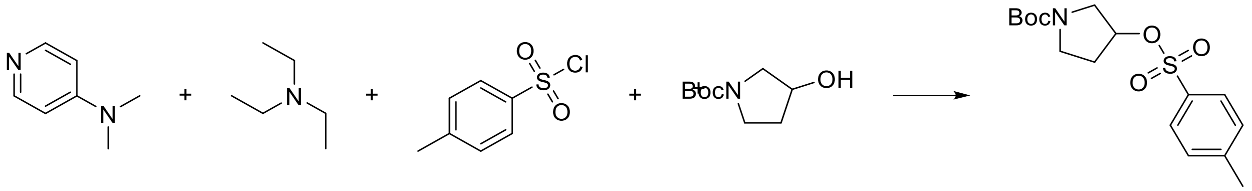

To a mixture of *tert*-butyl 3-hydroxypyrrolidine-1-carboxylate (1 g, 5.34 mmol), *N*,*N*-dimethylpyridin-4-amine (0.033 g, 0.267 mmol) and triethylamine (1.117 mL, 8.01 mmol) in anhydrous DCM (20 mL) was added 4-

methylbenzenesulfonyl chloride (1.120 g, 5.87 mmol) at 0 °C. The mixture was stirred briskly at 20 °C for 16 h and later diluted with brine (50 mL) and water (50 mL). The organic layer was separated, washed with brine (50 mL), dried over $MgSO_4$ and evaporated on a rotary evaporator. The residue obtained was purified by flash column chromatography [silica gel (40 g), eluting with EtOAc in hexane from 0–100%] to give *tert*-butyl 3-(tosyloxy)pyrrolidine-1-carboxylate as a mixture of rotomers (1.76 g, 5.15 mmol, 97% yield) as a clear oil; ^1H NMR (300 MHz, DMSO-d_6) δ 7.85 (d, J = 8.3 Hz, 2H), 7.50 (d, J = 8.2 Hz, 2H), 5.14 – 5.05 (m, 1H), 4.92 (s, 2H), 3.56 – 3.26 (m, 7H), 2.06 (d, J = 9.7 Hz, 2H), 1.47 (d, J = 7.2 Hz, 8H); $^{13}C\{H\}$ NMR (75 MHz, DMSO-d_6) δ 153.3, 153.2, 145.1, 133.2, 133.1, 130.3, 127.5, 81.7, 80.8, 78.7, 51.4, 51.4, 43.4, 43.1, 31.4, 30.6, 28.0, 21.1; IR (KBr disk) 2972, 1683, 1412, 1356, 1198, 1189, 1167, 1119 cm^{-1}; HRMS (ESI) m/z: $[M + Na]^+$ calculated for $C_{16}H_{23}NNaO_5S^+$ 364.1195, found 364.1221.

Preparation of (*R*)-tetrahydrofuran-3-yl 4-methylbenzenesulfonate (14j).

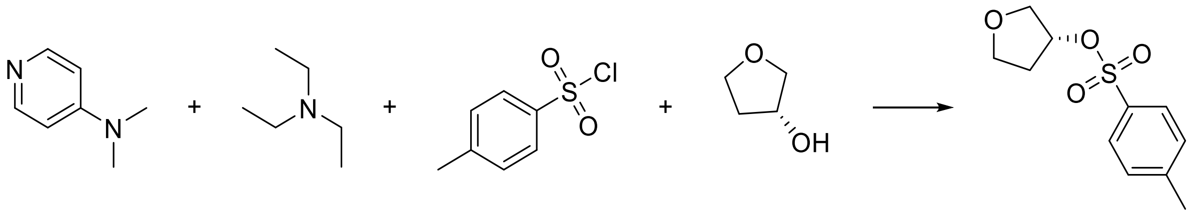

To a mixture of (*R*)-tetrahydrofuran-3-ol (1 g, 11.35 mmol), *N*,*N*-dimethylpyridin-4-amine (0.069 g, 0.567 mmol) and triethylamine (2.373 mL, 17.02 mmol) in anhydrous DCM (20 mL) was added 4-methylbenzenesulfonyl chloride (2.380 g, 12.48 mmol) at 0 °C. The mixture was stirred briskly at 20 °C for 16 h and later diluted with brine (50 mL) and water (50 mL). The organic layer was separated, washed with brine (50 mL), dried over $MgSO_4$ and evaporated on a rotary evaporator. The residue obtained was purified by flash column chromatography [silica gel (40 g), eluting with EtOAc in hexane from 0–100%] to give (*R*)-tetrahydrofuran-3-yl 4-methylbenzenesulfonate (2.175 g, 8.98 mmol, 79% yield) as a clear oil; ^1H NMR (300 MHz, DMSO-d_6) δ 7.87 – 7.78 (m, 2H), 7.49 (d, J = 8.1 Hz, 2H), 5.16 (ddt, J = 6.0, 3.9, 1.8 Hz, 1H), 4.92 (s, 2H), 3.95 – 3.70 (m, 4H), 2.22 – 2.09 (m, 1H), 2.04 (td, J = 11.4, 4.6 Hz, 1H); $^{13}C\{H\}$ NMR (75 MHz, DMSO-d_6) δ 145.1, 133.2, 130.3, 127.5,

82.4, 72.1, 66.0, 32.5, 21.1; IR (KBr disk) 2984, 2874, 1598, 1364, 1189, 1175, 1093, 1079 cm^{-1}; HRMS (ESI) m/z: $[M + H_2O]^+$ calculated for $C_{11}H_{16}O_5S^+$ 260.0718, found 260.0946.

Preparation of (*S*)-tetrahydrofuran-3-yl 4-methylbenzenesulfonate (14k).

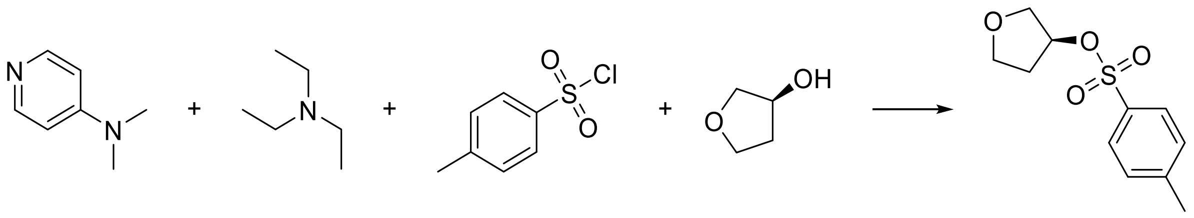

To a mixture of (*S*)-tetrahydrofuran-3-ol (1 g, 11.35 mmol), *N*,*N*-dimethylpyridin-4-amine (0.069 g, 0.567 mmol) and triethylamine (2.373 mL, 17.02 mmol) in anhydrous DCM (20 mL) was added 4-methylbenzenesulfonyl chloride (2.380 g, 12.48 mmol) at 0 °C. The mixture was stirred briskly at 20 °C for 16 h and later diluted with brine (50 mL) and water (50 mL). The organic layer was separated, washed with brine (50 mL), dried over $MgSO_4$ and evaporated on a rotary evaporator. The residue obtained was purified by flash column chromatography [silica gel (40 g), eluting with EtOAc in hexane from 0–100%] to give (*S*)-tetrahydrofuran-3-yl 4-methylbenzenesulfonate (2.45 g, 10.11 mmol, 89% yield) as a clear oil; ^{1}H NMR (300 MHz, DMSO-d_6) δ 7.89 – 7.78 (m, 2H), 7.49 (dt, J = 8.5, 2.7 Hz, 2H), 5.16 (ddt, J = 6.0, 4.1, 2.0 Hz, 1H), 4.92 (d, J = 2.5 Hz, 1H), 3.97 – 3.71 (m, 5H), 2.27 – 1.89 (m, 2H); ^{13}C{H} NMR (75 MHz, DMSO-d_6) δ 145.1, 133.2, 130.3, 127.5, 82.4, 72.1, 66.0, 32.5, 21.1; IR (KBr disk) 2984, 2956, 2874, 1598, 1360, 1189, 1176, 1093 cm^{-1}; HRMS (ESI) m/z: $[M + H_2O]^+$ calculated for $C_{11}H_{16}O_5S^+$ 260.0718, found 260.0948.

Preparation of (*S*)-tetrahydrofuran-3-yl 4-methylbenzenesulfonate (14l).

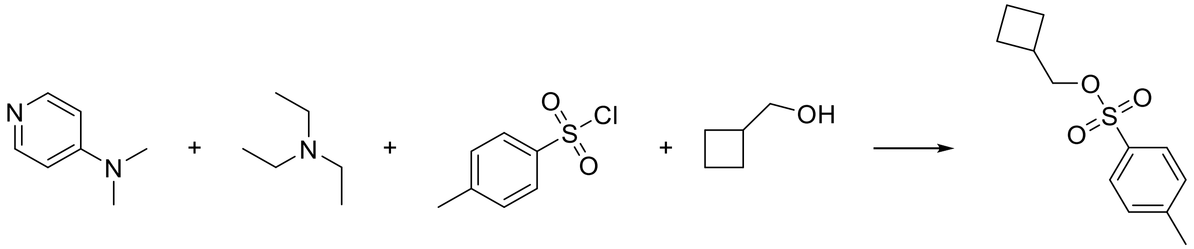

To a mixture of cyclobutylmethanol (1 g, 11.61 mmol), *N*,*N*-dimethylpyridin-4-amine (0.071 g, 0.580 mmol) and triethylamine (2.427 mL, 17.41 mmol) in anhydrous DCM (20 mL) was added 4-methylbenzenesulfonyl chloride (2.435 g, 12.77 mmol)at 0 °C. The mixture was stirred briskly at 20 °C for 16 h and later diluted with brine (50 mL) and water (50 mL). The organic layer was separated, washed with brine (50 mL), dried over MgSO4 and evaporated on a rotary evaporator. The residue obtained was purified by flash column chromatography [silica gel (40 g), eluting with EtOAc in hexane from 0–100%] to give cyclobutylmethyl 4-methylbenzenesulfonate (2.45 g, 10.19 mmol, 88% yield) as a clear oil; ^{1}H NMR (300 MHz, DMSO-d_6) δ 7.94 – 7.65 (m, 2H), 7.49 (d, J = 7.9 Hz, 2H), 4.93 (s, 2H), 4.01 (d, J = 6.6 Hz, 2H), 2.63 (p, J = 7.3 Hz, 1H), 2.04 (ddd, J = 10.9, 6.1, 2.5 Hz, 2H), 1.94 (d, J = 8.7 Hz, 1H), 1.87 (dd, J = 10.2, 5.2 Hz, 1H), 1.76 (q, J = 8.3 Hz, 2H); ^{13}C{H} NMR (75 MHz, DMSO-d_6) δ 144.7, 132.6, 130.1, 127.5, 74.0, 33.3, 23.6, 21.0, 17.6; IR (KBr disk) 2980, 2945, 2891, 2868, 1623, 1616, 1599, 1576, 1559, 1360, 1189, 1176, 1097 cm^{-1}; HRMS (ESI) m/z: $[M + NH_4]^+$ calculated for $C_{12}H_{20}NO_3S^+$ 258.1158, found 258.1160.

Preparation of *tert*-butyl 3-(tosyloxy)azetidine-1-carboxylate (14m).

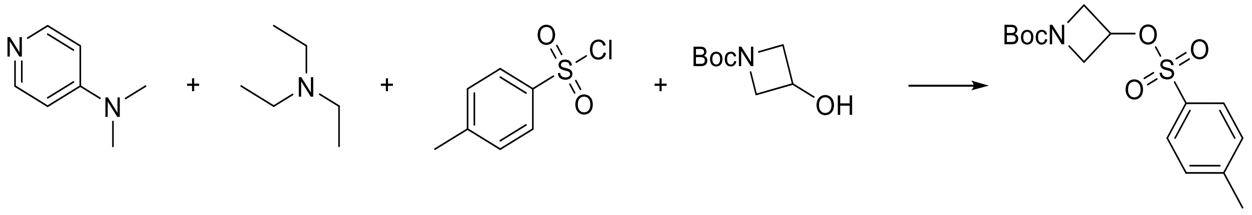

To a mixture of *tert*-butyl 3-hydroxyazetidine-1-carboxylate (1 g, 5.77 mmol), *N*,*N*-dimethylpyridin-4-amine (0.035 g, 0.289 mmol) and triethylamine (1.207 mL, 8.66 mmol) in anhydrous DCM (20 mL) was added 4-methylbenzenesulfonyl chloride (1.211 g, 6.35 mmol) at 0 °C. The mixture was stirred briskly at 20 °C for 16 h and later diluted with brine (50 mL) and water (50 mL). The organic layer was separated, washed with brine (50 mL), dried over MgSO4 and evaporated on a rotary evaporator. The residue obtained was purified by flash column chromatography [silica gel (40 g), eluting with EtOAc in hexane from 0–100%] to give *tert*-butyl 3-(tosyloxy)azetidine-1-carboxylate (1.6 g, 85% yield) as a clear oil. ^{1}H NMR (300 MHz, DMSO-d_6) δ 7.81 (d, J = 8.5 Hz, 2H), 7.51 (d, J = 7.9 Hz, 2H), 5.08 (tt, J = 6.6, 3.9 Hz, 1H), 4.17 – 3.86 (m, 2H), 3.73 (s, 2H), 2.43 (s,

3H), 1.34 (s, 9H); ^{13}C{H} NMR (75 MHz, DMSO-d_6) δ 155.3, 145.5, 132.2, 130.3, 127.7, 79.2, 68.6, 55.9, 27.9, 21.1; IR (KBr disk) 2978, 2933, 2886, 1705, 1598, 1495, 1479, 1456, 1401, 1367, 1299, 1256, 1210, 1192, 1179, 1091, 1022 cm^{-1}; HRMS (ESI) m/z: [M + Na]$^+$ calculated for $C_{15}H_{21}NNaO_5S^+$ 350.1033, found 350.1043.

Preparation of cyclopentylmethyl 4-methylbenzenesulfonate (14n).

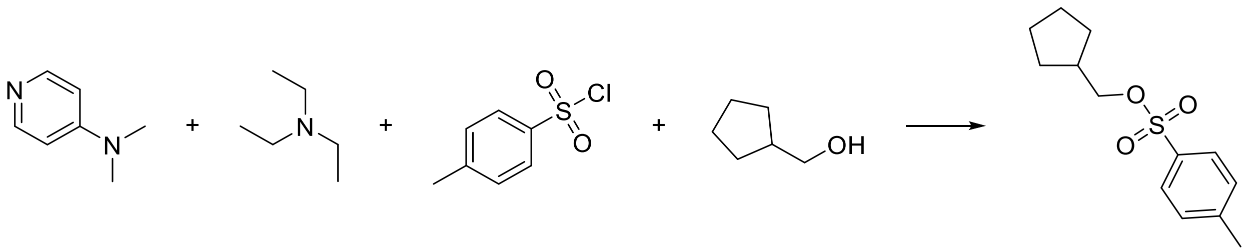

To a mixture of cyclopentylmethanol (1 g, 9.98 mmol), *N*,*N*-dimethylpyridin-4-amine (0.061 g, 0.499 mmol) and triethylamine (2.087 mL, 14.98 mmol) in anhydrous DCM (20 mL) was added 4-methylbenzenesulfonyl chloride (2.094 g, 10.98 mmol) at 0 °C. The mixture was stirred briskly at 20 °C for 16 h and later diluted with brine (50 mL) and water (50 mL). The organic layer was separated, washed with brine (50 mL), dried over MgSO4 and evaporated on a rotary evaporator. The residue obtained was purified by flash column chromatography [silica gel (40 g), eluting with EtOAc in hexane from 0–100%] to give cyclopentylmethyl 4-methylbenzenesulfonate (2.2 g, 8.65 mmol, 87% yield) as a clear oil; ^{1}H NMR (300 MHz, DMSO-d_6) δ 7.82 (dt, J = 8.8, 2.4 Hz, 2H), 7.53 – 7.43 (m, 2H), 4.93 (d, J = 2.6 Hz, 3H), 3.99 – 3.84 (m, 2H), 2.29 – 2.13 (m, 1H), 1.75 (d, J = 11.4 Hz, 2H), 1.65 – 1.51 (m, 4H), 1.34 – 1.14 (m, 2H); ^{13}C{H} NMR (75 MHz, DMSO-d_6) δ 144.7, 132.6, 130.0, 127.5, 74.0, 38.0, 28.3, 24.7, 21.0; IR (KBr disk) 2956, 2870, 1599, 1495, 1454, 1362, 1190, 1097, 1020 cm^{-1}; HRMS (ESI) m/z: [M + NH$_4$]$^+$ calculated for $C_{13}H_{22}NO_3S^+$ 272.1315, found 272.1324.

Preparation of (tetrahydrofuran-3-yl)methyl 4-methylbenzenesulfonate (14o).

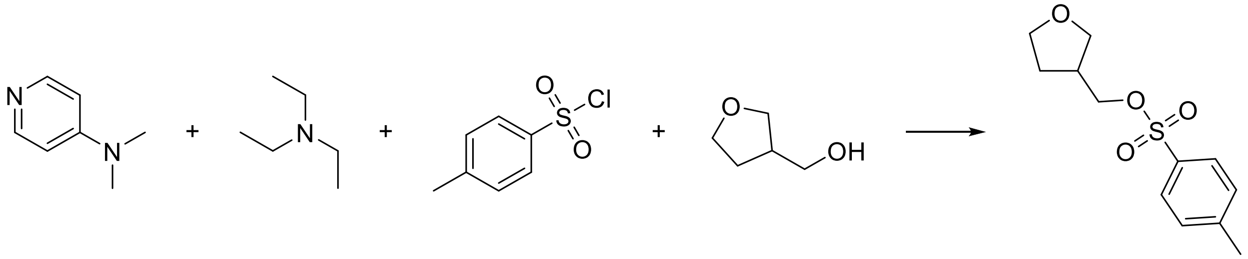

To a mixture of (tetrahydrofuran-3-yl)methanol (1 g, 9.79 mmol), *N*,*N*-dimethylpyridin-4-amine (0.060 g, 0.490 mmol) and triethylamine (2.047 mL, 14.69 mmol) in anhydrous DCM (20 mL) was added 4-methylbenzenesulfonyl chloride (2.053 g, 10.77 mmol)at 0 °C. The mixture was stirred briskly at 20 °C for 16 h and later diluted with brine (50 mL) and water (50 mL). The organic layer was separated, washed with brine (50 mL), dried over MgSO4 and evaporated on a rotary evaporator. The residue obtained was purified by flash column chromatography [silica gel (40 g), eluting with EtOAc in hexane from 0–100%] to give (tetrahydrofuran-3-yl)methyl 4-methylbenzenesulfonate (2.215 g, 8.64 mmol, 88% yield) as a clear oil; ^{1}H NMR (300 MHz, DMSO-*d*$_6$) δ 7.90 – 7.75 (m, 2H), 7.49 (dd, *J* = 8.4, 2.4 Hz, 2H), 4.09 – 3.89 (m, 2H), 3.87 – 3.62 (m, 3H), 3.50 (dd, *J* = 9.0, 5.2 Hz, 1H), 2.61 (q, *J* = 7.1 Hz, 1H), 2.50 (d, *J* = 2.7 Hz, 3H), 2.03 (dtd, *J* = 13.1, 8.0, 5.5 Hz, 1H), 1.69 – 1.51 (m, 1H); ^{13}C{H} NMR (75 MHz, DMSO-*d*$_6$) δ 145.1, 132.4, 130.3, 127.7, 72.0, 69.1, 66.9, 37.9, 28.0, 21.1; IR (KBr disk) 2974, 2871, 1779, 1599, 1495, 1360, 1190, 1097 cm^{-1}; HRMS (ESI) m/z: $[M + H]^+$ calculated for $C_{12}H_{17}O_4S^+$ 257.0842, found 257.0854.

Preparation of cyclohexylmethyl 4-methylbenzenesulfonate (14p).

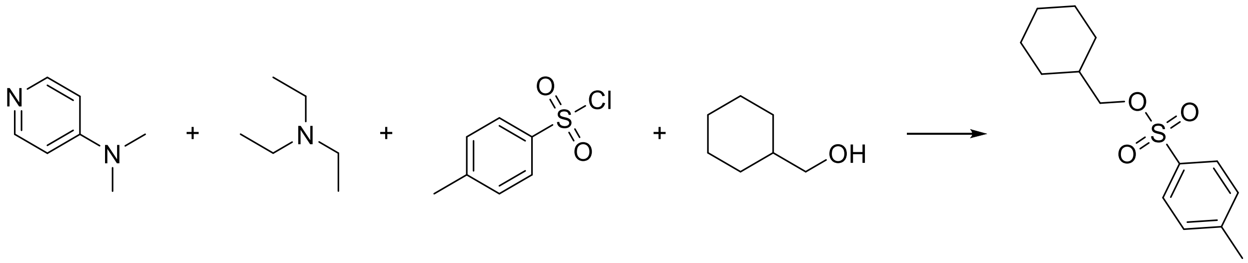

To a mixture of cyclohexylmethanol (1 g, 8.76 mmol), *N*,*N*-dimethylpyridin-4-amine (0.053 g, 0.438 mmol) and triethylamine (1.831 mL, 13.14 mmol) in anhydrous DCM (20 mL) was added 4-methylbenzenesulfonyl chloride (1.836 g, 9.63 mmol) at 0 °C. The mixture was stirred briskly at 20 °C for 16 h and later diluted with brine (50 mL) and water (50 mL). The organic layer was separated, washed with brine (50 mL), dried over $MgSO_4$ and evaporated on a rotary evaporator. The residue obtained was purified by flash column chromatography [silica gel (40 g), eluting with EtOAc in hexane from 0–100%] to give cyclohexylmethyl 4-methylbenzenesulfonate (850mg, 3.17 mmol, 36.2% yield) as a clear oil; ^{1}H NMR (300 MHz, DMSO-*d*$_6$) δ 7.81 (dt, J = 8.4, 2.0 Hz, 2H), 7.49 (d,

J = 7.9 Hz, 2H), 4.93 (d, J = 1.6 Hz, 4H), 3.85 (dt, J = 6.1, 2.0 Hz, 2H), 1.69 (d, J = 13.1 Hz, 6H), 1.34 – 1.13 (m, 3H), 0.97 (t, J = 11.6 Hz, 2H); $^{13}C\{H\}$ NMR (75 MHz, DMSO-d_6) δ 144.8, 132.5, 130.1, 127.5, 75.2, 36.5, 28.4, 25.6, 24.9, 21.1; IR (KBr disk) 2950, 2875, 1599, 1494, 1450, 1360, 1306, 1291, 1189, 1176, 1097, 1019 cm^{-1}; HRMS (ESI) m/z: $[M + H_2O]^+$ calculated for $C_{14}H_{22}O_4S^+$ 286.1239, found 286.1476.

Figure S3. 2D NOE of 6. Circled in-phase correlation is between CH3 and NH.

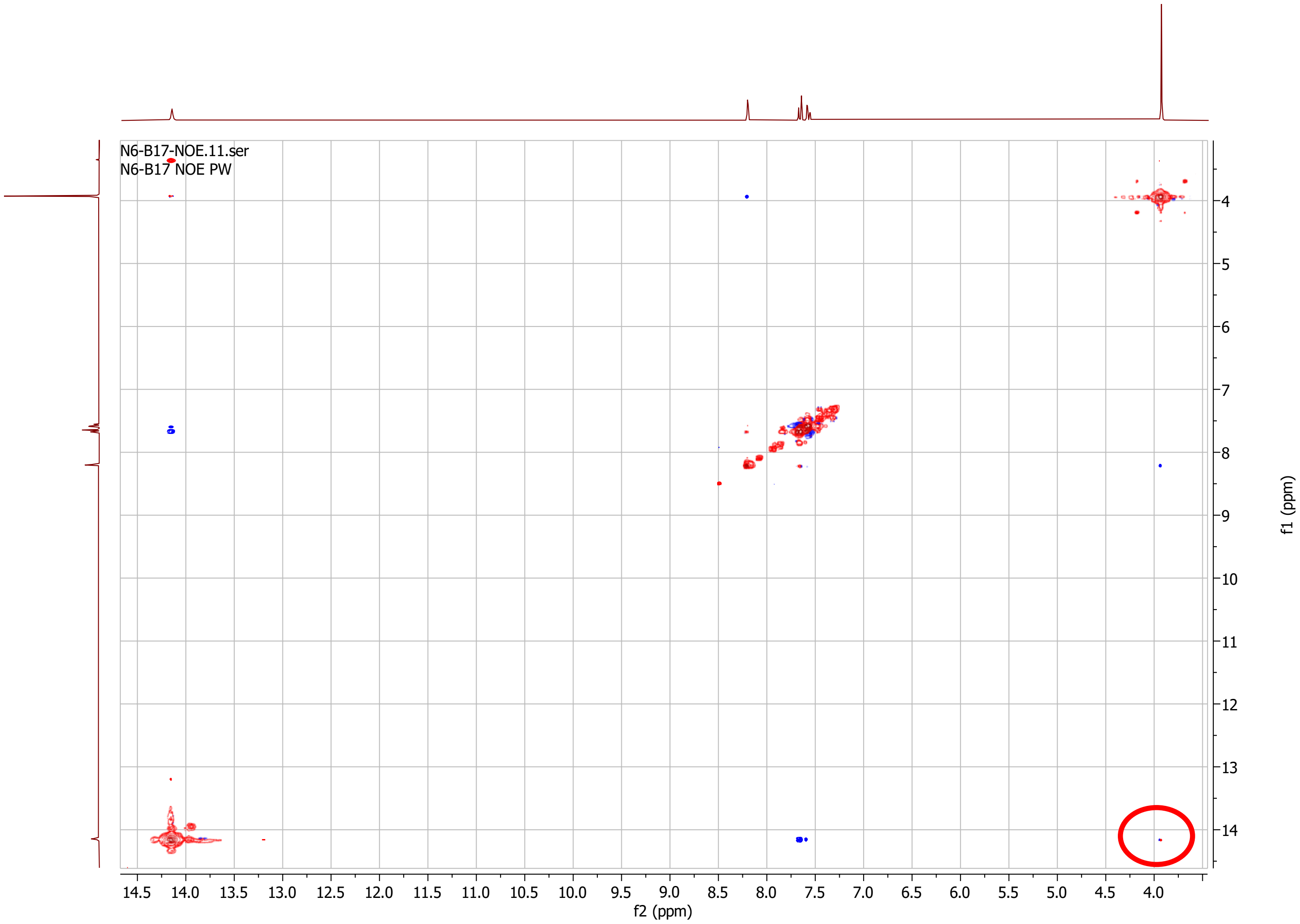

Preparation of methyl 5-bromo-1-methy-1*H*-indazole-3-carboxylate (15a)

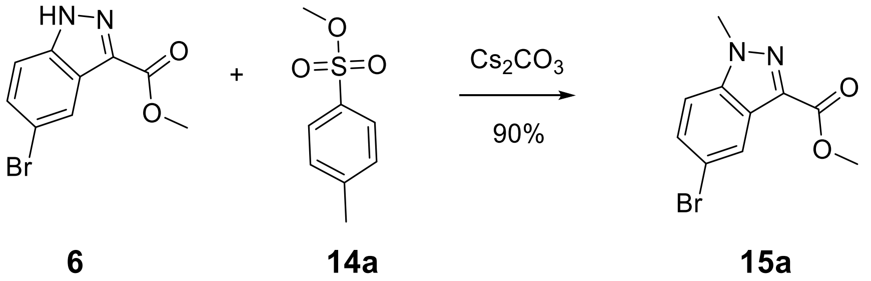

To a solution of methyl 5-bromo-1*H*-indazole-3-carboxylate (**6**, 300 mg, 1.176 mmol) in dioxane (10 mL) at RT was added cesium carbonate (766 mg, 2.352 mmol) followed by tosylate **14a**. The resulting mixture was stirred for 2 h at 90 °C. The mixture was poured into EtOAc (500 mL) and washed with water (100 mL) and brine. The organic layer was dried and concentrated, the obtained residue was purified by chromatography [silica gel (24 g), eluting with EtOAc in hexane from 0–70%] to give methyl 5-bromo-1-methyl-1*H*-indazole-3-carboxylate, **15a**, (285 mg, 1.059 mmol, 90% yield) as a white solid; melting point = 141.3 °C. 1H NMR (300 MHz, DMSO-d_6) δ 8.19 (dd, J = 1.9, 0.7 Hz, 1H), 7.82 (dd, J = 9.0, 0.7 Hz, 1H), 7.65 (dd, J = 8.9, 1.9 Hz, 1H), 4.17 (s, 3H), 3.92 (s, 3H); ^{13}C{1H} NMR (75 MHz, DMSO-d_6) δ 161.8, 139.4, 132.7, 129.4, 124.1, 123.0, 116.0, 113.0, 51.8, 36.6; IR (KBr disk) 1708, 1459, 1442, 1392, 1326, 1252, 1196 cm^{-1}; HRMS (ESI) m/z: $[M + H]^+$ calculated for $C_{10}H_{10}BrN_2O_2^+$ 268.9921, found 268.9902. **16a** isolated yield: n.d.

Preparation of methyl 5-bromo-1-ethyl-1*H*-indazole-3-carboxylate (15b)

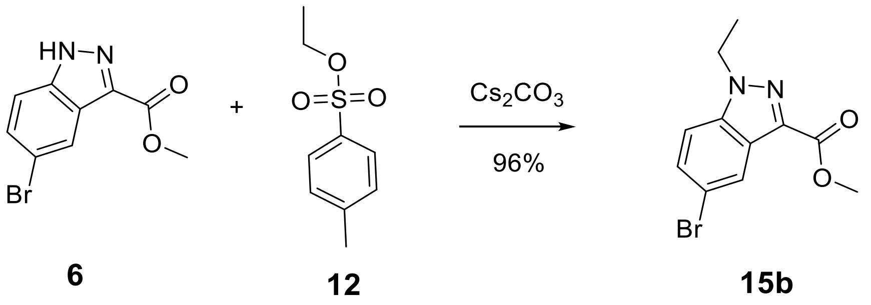

The compound was prepared from **6** (500 mg, 1.96 mmol) using similar procedure as compound **15a** with 96% yield (532 mg, 1.879 mmol) as a white solid as a white solid: melting point = 78.4 °C; ^{1}H NMR (300 MHz, DMSO-d_6) δ 8.21 (d, J = 1.8 Hz, 1H), 7.87 (d, J = 8.9 Hz, 1H), 7.64 (dd, J = 8.9, 1.9 Hz, 1H), 4.56 (q, J = 7.2 Hz,

2H), 3.93 (s, 3H), 1.43 (t, *J* = 7.2 Hz, 3H); $^{13}C\{1H\}$ NMR (75 MHz, DMSO-d_6) δ 162.0, 138.3, 132.9, 129.3, 124.3, 123.2, 116.0, 112.9, 51.8, 50.9, 21.9; IR (KBr disk) 1705, 1466, 1196, 1183, 1086, 1067, 1041 cm^{-1}; HRMS (ESI) m/z: $[M + H]^+$ calculated for $C_{11}H_{11}BrN_2O_2^+$ 282.0077, found 282.0092. **16b** isolated yield, 25 mg, 5%.

Preparation of methyl 5-bromo-1-propyl-1*H*-indazole-3-carboxylate (15c)

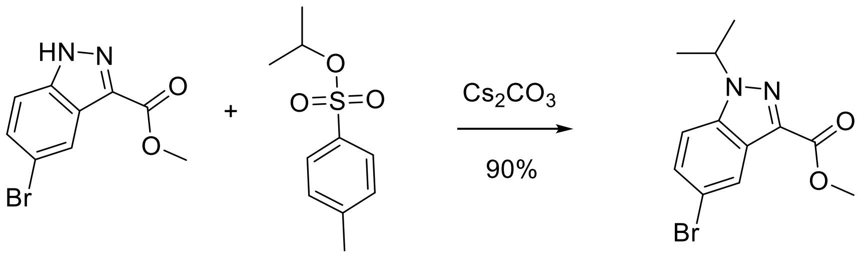

6 **14c** **15c**

The compound was prepared from **6** (0.238 g, 0.933 mmol) using similar procedure as compound **15a** with 90% yield (249 mg, 0.838 mmol) as a white solid; melting point = 106.9 °C; ^1H NMR (300 MHz, DMSO-d_6) δ 8.21 (d, *J* = 1.9, 0.7 Hz, 1H), 7.90 (d, *J* = 9.1, 0.7 Hz, 1H), 7.63 (dd, *J* = 9.0, 1.9 Hz, 1H), 5.15 (hept, *J* = 6.6 Hz, 1H), 3.93 (s, 3H), 1.50 (d, *J* = 6.6 Hz, 6H); $^{13}C\{1H\}$ NMR (75 MHz, DMSO-d_6) δ 162.0, 138.3, 132.9, 129.3, 124.3, 123.2, 116.0, 112.9, 51.8, 50.9, 21.9; IR (KBr disk) 1485, 1462, 1436, 1392, 1332, 1196 cm^{-1}; HRMS (ESI) m/z: $[M + H]^+$ calculated for $C_{12}H_{13}BrN_2O_2^+$ 296.0233, found 296.0254. **16c** isolated yield: n.d.

Preparation of methyl 5-bromo-1-propyl-1*H*-indazole-3-carboxylate (15d)

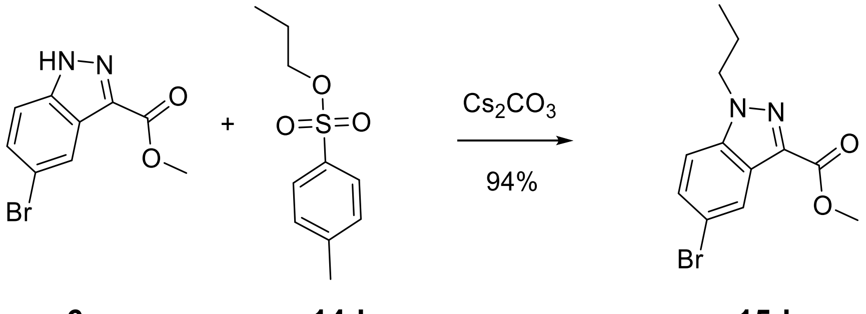

6 **14d** **15d**

The compound was prepared from **6** (238 mg, 0.933 mmol) using similar procedure as compound **15a** with 94% yield (261 mg, 0.878 mmol) as a white solid; melting point = 63.8 °C; ^{1}H NMR (300 MHz, DMSO-*d*$_6$) δ 8.21 (d, *J* = 1.9, 0.7 Hz, 1H), 7.88 (d, *J* = 9.0, 0.7 Hz, 1H), 7.63 (d, *J* = 9.0, 1.9 Hz, 1H), 4.49 (t, *J* = 6.9 Hz, 2H), 3.93 (s, 3H), 1.87 (h, *J* = 7.3 Hz, 2H), 0.82 (t, *J* = 7.4 Hz, 3H). ^{13}C{1H} NMR (75 MHz, DMSO-*d*$_6$) δ 161.9, 139.2, 133.0, 129.4, 124.1, 123.2, 116.0, 112.9, 51.8, 50.7, 22.7, 10.9; IR (KBr disk) 1725, 1464, 1190, 1175, 1149, 1118 cm^{-1}; HRMS (ESI) m/z: [M + H]$^+$ calculated for $C_{12}H_{13}BrN_2O_2^+$ 296.0233, found 296.0245. **16d** isolated yield: 10 mg, 0.034 mmol, 3.61%.

Preparation of methyl (*S*)-5-bromo-1-(sec-butyl)-1*H*-indazole-3-carboxylate (15e)

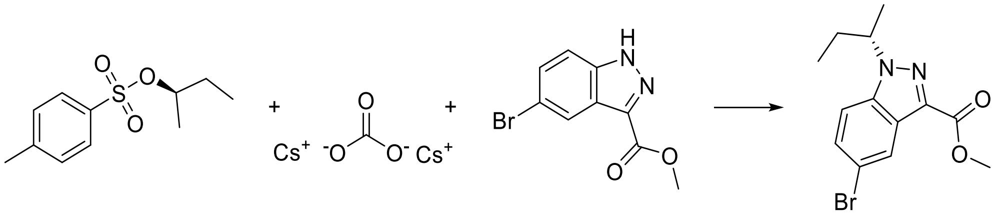

This compound was prepared from **6** (0.223 g, 0.876 mmol) using similar procedure as compound **15a** in 95% yield (259 mg, 0.832 mmol) as a white solid; m.p. = 68.4 °C; ^{1}H NMR (300 MHz, DMSO-*d6*) δ 8.22 (d, *J* = 1.8 Hz, 1H), 7.92 (d, *J* = 9.0 Hz, 1H), 7.62 (dd, *J* = 9.0, 1.9 Hz, 1H), 4.98 – 4.85 (m, 1H), 3.93 (s, 3H), 2.05 – 1.76 (m, 2H), 1.50 (d, *J* = 6.6 Hz, 3H), 0.66 (t, *J* = 7.4 Hz, 3H); ^{13}C{H} NMR (75 MHz, DMSO-*d*$_6$) δ 162.0, 139.3, 133.2, 129.4, 124.0, 123.3, 116.0, 112.9, 56.5, 51.8, 29.2, 20.2, 10.5; IR (KBr disk) 2965, 2937, 2875, 1701, 1451, 1377, 1244, 1205 cm^{-1}; HRMS (ESI) m/z: [M$^+$] calculated for $C_{13}H_{15}BrN_2O_2^+$ 310.0390, found 310.0399.

16e isolated yield: 6 mg, 0.019 mmol, 2.201% yield.

Preparation of methyl 5-bromo-1-isobutyl-1*H*-indazole-3-carboxylate (15f)

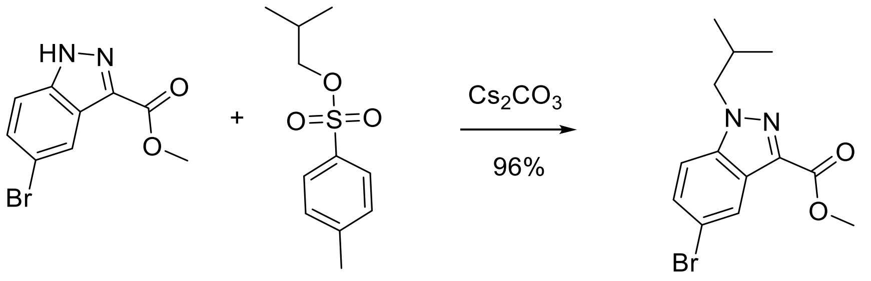

6 **14e** **15e**

The compound was prepared from **6** (0.223 g, 0.876 mmol) using a similar procedure as compound **15a** with 96% yield (261 mg, 0.839 mmol) as a white solid; melting point = 98.4 °C; ^{1}H NMR (300 MHz, DMSO-d_6) δ 8.20 (d, *J* = 1.9, 0.7 Hz, 1H), 7.89 (d, *J* = 9.0, 0.7 Hz, 1H), 7.63 (dd, *J* = 9.0, 1.9 Hz, 1H), 4.35 (d, *J* = 7.3 Hz, 2H), 3.93 (s, 3H), 2.31 – 2.13 (m, 1H), 0.85 (d, *J* = 6.7 Hz, 6H). ^{13}C{1H} NMR (75 MHz, DMSO-d_6) δ 162.0, 139.8, 133.1, 129.6, 124.0, 123.2, 116.0, 113.3, 55.0, 51.8, 29.9, 25.8, 25.1; IR (KBr disk) 2961, 1733, 1717, 1482, 1466, 1229, 1194, 1122 cm^{-1}; HRMS (ESI) m/z: [M + H]$^{+}$ calculated for $C_{13}H_{15}BrN_2O_2^{+}$ 310.0399, found 310.0394. **16f** isolated yield: 3 mg, 9.64 µmol, 1.101%.

Preparation of methyl 5-bromo-1-(2-methoxyethyl)-1*H*-indazole-3-carboxylate (15g)

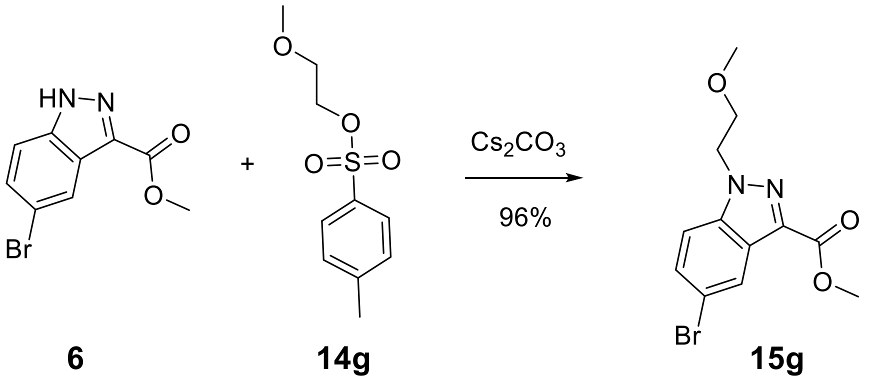

6 **14g** **15g**

The compound was prepared from **6** (0.222 g, 0.869 mmol) using a similar procedure as compound **15a** with 96% yield (260 mg, 0.830 mmol) as a white solid; melting point = 111.5 °C; ^{1}H NMR (300 MHz, DMSO-d_6) δ 8.21 (d, *J* = 1.9, 0.7 Hz, 1H), 7.85 (d, *J* = 9.0, 0.7 Hz, 1H), 7.64 (dd, *J* = 9.0, 1.9 Hz, 1H), 4.71 (t, *J* = 5.1 Hz, 2H), 3.94

(s, 3H), 3.79 (t, J = 5.1 Hz, 2H), 3.18 (s, 3H); ^{13}C{1H} NMR (75 MHz, DMSO-d_6) δ 161.9, 139.9, 133.3, 129.5, 124.1, 123.1, 116.0, 113.4, 70.4, 58.1, 51.8, 49.4; IR (KBr disk) 1718, 1483, 1461, 1395, 1228, 1198, 1164, 1112, 1011 cm^{-1}; HRMS (ESI) m/z: $[M + H]^+$ calculated for $C_{12}H_{13}BrN_2O_3^+$ 312.0182, found 312.0189. **16f** isolated yield: 5 mg, 0.016 mmol, 1.838%.

Preparation of methyl 5-bromo-1-cyclopentyl-1*H*-indazole-3-carboxylate (15h)

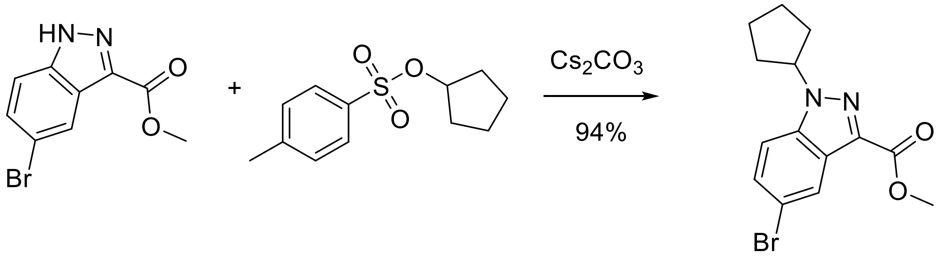

6 **14h** **15h**

The compound was prepared from **6** (0.212 g, 0.832 mmol) using a similar procedure as compound **15a** with 94% yield (252 mg, 0.780 mmol) as a white solid; melting point = 84.6 °C; ^{1}H NMR (300 MHz, DMSO-d_6) δ 8.21 (d, J = 1.9, 0.7 Hz, 1H), 7.89 (d, J = 9.0, 0.7 Hz, 1H), 7.63 (dd, J = 9.0, 1.9 Hz, 1H), 5.39 – 5.22 (m, 1H), 3.93 (s, 3H), 2.25 – 2.12 (m, 2H), 2.07 – 1.94 (m, 2H), 1.95 – 1.81 (m, 2H), 1.79 – 1.66 (m, 2H). ^{13}C{1H} NMR (75 MHz, DMSO-d_6) δ 162.0, 138.9, 132.8, 129.4, 124.4, 123.2, 123.2, 116.0, 113.0, 59.6, 51.8, 32.0, 24.2; IR (KBr disk) 2950, 2871, 1712, 1477, 1399, 1231, 1194, 1155, 1131, 1097 cm^{-1}; HRMS (ESI) m/z: $[M + H]^+$ calculated for $C_{14}H_{15}BrN_2O_2^+$ 322.0390, found 322.0404. **16g** isolated yield: 12 mg, 0.037 mmol, 4.46%.

Preparation of methyl 5-bromo-1-(1-(*tert*-butoxycarbonyl)pyrrolidin-3-yl)-1*H*-indazole-3-carboxylate (15i).

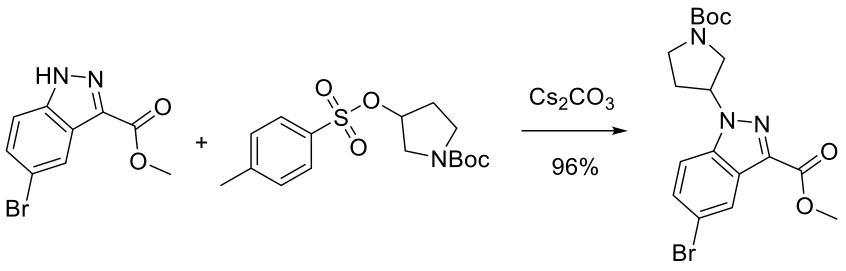

6 **14i** **15i**

The compound was prepared from **6** (0.149 g, 0.586 mmol) as a mixture of rotomers using a similar procedure as compound **15a** with 96% yield (239 mg, 0.563 mmol) as a white solid; melting point = 106.8 °C; ^{1}H NMR (300 MHz, DMSO-d_6) δ 8.22 (d, *J* = 1.8 Hz, 1H), 7.92 (d, *J* = 9.0 Hz, 1H), 7.67 (dd, *J* = 9.0, 1.9 Hz, 1H), 5.59 (d, *J* = 6.4 Hz, 1H), 3.93 (s, 3H), 3.88 – 3.78 (m, 1H), 3.65 – 3.54 (m, 2H), 3.46 (d, *J* = 7.1 Hz, 1H), 2.39 (s, 2H), 1.40 (d, *J* = 8.8 Hz, 9H); ^{13}C{1H} NMR (75 MHz, DMSO-d_6) δ 161.8, 153.5, 153.4, 139.1, 133.5, 129.8, 124.3, 123.3, 116.3, 113.0, 78.7, 57.5, 56.8, 56.8, 51.9, 50.4, 50.4, 44.7, 44.4, 31.0, 30.2, 28.1; IR (KBr disk) 2980, 2955, 2933, 2886, 1718, 1684, 1481, 1412, 1219, 1173, 1116, 1100 cm^{-1}; HRMS (ESI) m/z: [M + Na]$^+$ calculated for $C_{18}H_{22}BrN_3O_4^+$ 423.0866, found 446.0707. **16i** isolated yield: 3 mg, 7.07 µmol, 1.207%.

Preparation of methyl (*S*)-5-bromo-1-(tetrahydrofuran-3-yl)-1*H*-indazole-3-carboxylate (15j).

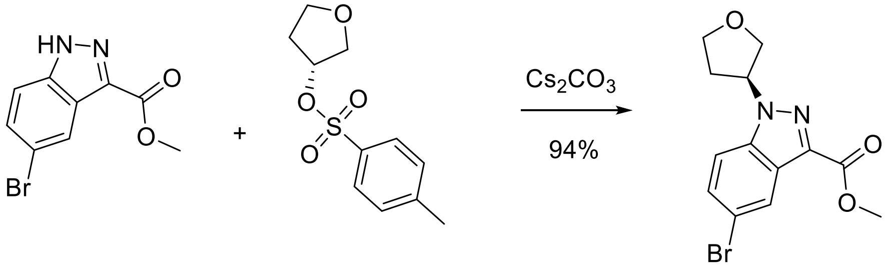

6 **14j** **15j**

The compound was prepared from **6** (0.211 g, 0.825 mmol) using a similar procedure as compound **15a** with 94% yield (253 mg, 0.778 mmol) as a white solid; melting point = 108.8 °C; ^{1}H NMR (300 MHz, DMSO-d_6) δ 8.23

(d, J = 1.9, 0.7 Hz, 1H), 7.91 (d, J = 9.0, 0.7 Hz, 1H), 7.66 (dd, J = 9.0, 1.9 Hz, 1H), 5.68 – 5.59 (m, 1H), 4.14 – 4.04 (m, 2H), 3.98 (d, J = 3.8 Hz, 1H), 3.93 (s, 3H), 3.91 – 3.82 (m, 1H), 2.49 – 2.44 (m, 1H), 2.40 – 2.28 (m, 1H); ^{13}C{1H} NMR (75 MHz, DMSO-d_6) δ 161.8, 138.9, 133.2, 129.6, 124.5, 123.3, 116.2, 113.0, 71.6, 67.4, 59.0, 51.9, 32.1; HRMS (ESI) m/z: $[M + H]^+$ calculated for $C_{13}H_{13}BrN_2O_3^+$ 324.0182, found 324.0187. **16j** isolated yield: 5 mg, 0.015 mmol, 1.863%.

Preparation of methyl (*R*)-5-bromo-1-(tetrahydrofuran-3-yl)-1*H*-indazole-3-carboxylate (15k).

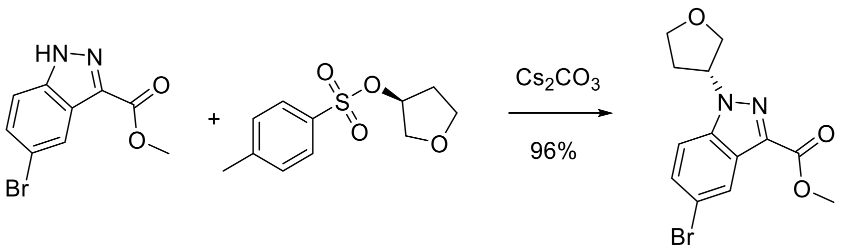

6 **14k** **15k**

The compound was prepared from **6** (0.211 g, 0.825 mmol) using a similar procedure as compound **15a** with 96% yield (257 mg, 0.790 mmol) as a white solid; melting point = 89.9 °C; ^{1}H NMR (300 MHz, DMSO-d_6) δ 8.23 (d, J = 1.8 Hz, 1H), 7.92 (d, 1H), 7.67 (dd, J = 9.0, 1.9 Hz, 1H), 5.70 – 5.59 (m, 1H), 4.15 – 4.05 (m, 2H), 3.99 (d, J = 3.8 Hz, 1H), 3.94 (s, 3H), 3.88 (td, J = 8.1, 5.9 Hz, 1H), 2.56 (d, J = 7.6 Hz, 1H), 2.41 – 2.25 (m, 1H); ^{13}C{1H} NMR (75 MHz, DMSO-d_6) δ 161.9, 138.9, 133.2, 129.6, 124.5, 123.3, 116.2, 113.0, 71.6, 67.4, 59.0, 51.9, 32.1; IR (KBr disk) 1701, 1459, 1435, 1399, 1230, 1209, 1168 cm^{-1}; HRMS (ESI) m/z: $[M + H]^+$ calculated for $C_{13}H_{13}BrN_2O_3^+$ 324.0182, found 324.0196. **16k** isolated yield: 4 mg, 0.012 mmol, 1.49%.

Preparation of methyl 5-bromo-1-(cyclobutylmethyl)-1*H*-indazole-3-carboxylate (15l).

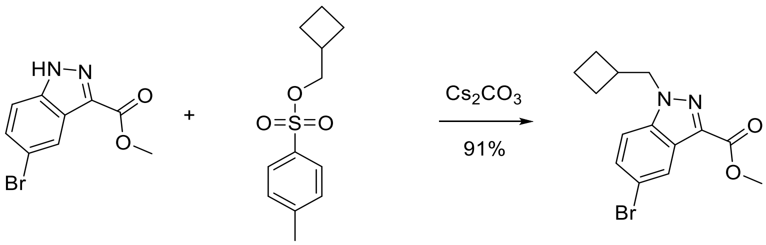

6 **14l** **15l**

The compound was prepared from **6** (0.212 g, 0.832 mmol using a similar procedure as compound **15a** in 91% yield (245 mg, 0.758 mmol) as a white solid; m.p. = 88.9 °C; ^{1}H NMR (300 MHz, DMSO-d_6) δ 8.20 (d, *J* = 1.9, 0.7 Hz, 1H), 7.90 (d, *J* = 9.0, 0.7 Hz, 1H), 7.63 (dd, *J* = 9.0, 1.9 Hz, 1H), 4.56 (d, *J* = 7.2 Hz, 2H), 3.93 (s, 3H), 2.85 (p, *J* = 7.4 Hz, 1H), 1.99 – 1.87 (m, 2H), 1.87 – 1.73 (m, 4H); ^{13}C{H} NMR (75 MHz, DMSO-d_6) δ 162.0, 139.4, 133.1, 129.5, 124.1, 123.2, 116.0, 113.1, 53.8, 51.8, 35.2, 25.3, 17.7; IR (KBr disk) 2953, 2933, 1718, 1466, 1449, 1433, 1407, 1317, 1244, 1205, 1170 cm^{-1}; HRMS (ESI) m/z: [M$^+$] calculated for $C_{14}H_{15}BrN_2O_2^+$ 322.0390, found 322.0393.**16l** isolated yield: 4 mg, 0.012 mmol, 1.487% yield.

Preparation of methyl 5-bromo-1-((1-(*tert*-butoxycarbonyl)azetidin-3-yl)methyl)-1*H*-indazole-3-carboxylate (15m). No reaction.

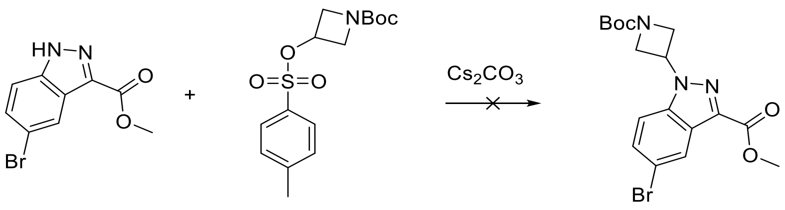

6 **14m** **15m**

Preparation of methyl 5-bromo-1-(cyclopentylmethyl)-1*H*-indazole-3-carboxylate (15n).

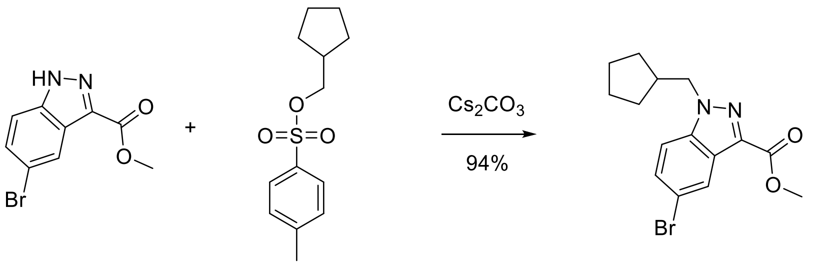

6 **14n** **15n**

The compound was prepared from **6** (0.201 g, 0.786 mmol) in 94% yield (248 mg, 0.735 mmol) as a white solid; m.p. = 111.4 °C; ^{1}H NMR (300 MHz, DMSO-*d*$_6$) δ 8.21 (d, *J* = 1.8 Hz, 1H), 7.90 (d, *J* = 8.7 Hz, 1H), 7.63 (dd, *J* = 9.0, 1.9 Hz, 1H), 4.46 (d, *J* = 7.5 Hz, 2H), 3.93 (s, 3H), 2.48 – 2.44 (m, 1H), 1.68 – 1.43 (m, 6H), 1.35 – 1.20 (m, 2H); ^{13}C{1H} NMR (75 MHz, DMSO-*d*$_6$) δ 162.0, 139.3, 133.0, 129.5, 124.1, 123.2, 116.0, 113.1, 53.6, 51.8, 29.6, 24.4; IR (KBr disk) 2950, 2864, 1722, 1457, 1435, 1405, 1358, 1321, 1231, 1194, 1157, 1123 cm^{-1}; HRMS (ESI) m/z: [M$^+$] calculated for $C_{15}H_{17}BrN_2O_2^+$ 336.0546, found 336.0567. **16n** isolated yield: 5 mg, 0.015 mmol, 1.886% yield.

Preparation of methyl 5-bromo-1-((tetrahydrofuran-2-yl)methyl)-1*H*-indazole-3-carboxylate (15o).

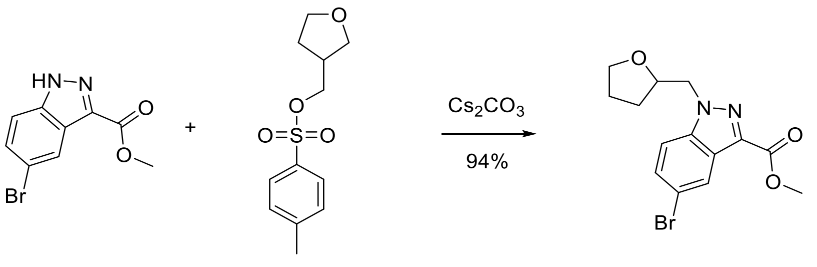

6 **14o** **15o**

The compound was prepared from **6** (0.199 g, 0.780 mmol) in 94% yield (248 mg, 0.735 mmol) as a white solid; m.p. = 74.5 °C; ^{1}H NMR (300 MHz, DMSO-*d*$_6$) δ 8.23 (d, 1H), 7.93 (d, *J* = 9.0, 0.7 Hz, 1H), 7.66 (dd, *J* = 9.0, 1.9 Hz, 1H), 4.54 (d, *J* = 7.5 Hz, 2H), 3.94 (s, 3H), 3.88 – 3.74 (m, 1H), 3.71 – 3.57 (m, 2H), 3.48 (dd, *J* = 8.7, 5.6 Hz, 1H), 2.83 (p, *J* = 6.6 Hz, 1H), 1.98 – 1.81 (m, 1H), 1.72 – 1.55 (m, 1H); ^{13}C{H} NMR (75 MHz,

DMSO-d_6) δ 162.3, 139.8, 133.7, 130.1, 124.6, 123.7, 116.6, 113.4, 70.4, 67.2, 52.3, 51.9, 29.8; IR (KBr disk) 2950, 2866, 1732, 1713, 1483, 1224, 1166, 1123 cm^{-1}; HRMS (ESI) m/z: [M^+] calculated for $C_{14}H_{15}BrN_2O_3^+$ 338.0339, found 338.0355. **16o** isolated yield: 5 mg, 0.015 mmol, 2% yield.

Preparation of methyl 5-bromo-1-(cyclohexylmethyl)-1*H*-indazole-3-carboxylate (15p).

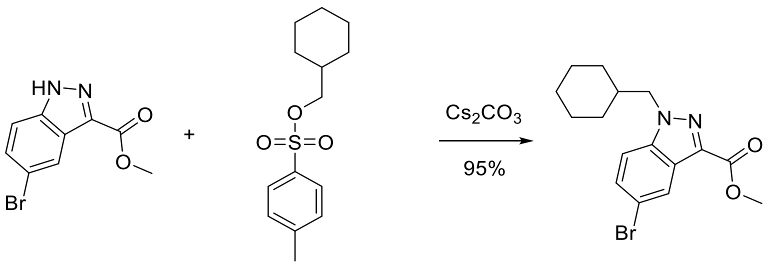

6 **14p** **15p**

The compound was prepared from **6** (0.190 g, 0.745 mmol) using a similar procedure as compound **15a** with 95% yield (249 mg, 0.709 mmol) as a white solid; melting point = 136.5 °C; ^{1}H NMR (300 MHz, DMSO-d_6) δ 8.21 (d, *J* = 1.9, 0.6 Hz, 1H), 7.89 (d, *J* = 9.0, 0.7 Hz, 1H), 7.63 (dd, *J* = 9.0, 1.9 Hz, 1H), 4.38 (d, *J* = 7.2 Hz, 2H), 3.94 (s, 3H), 2.00 – 1.83 (m, 1H), 1.63 (s, 3H), 1.46 (d, *J* = 12.1 Hz, 2H), 1.14 (t, *J* = 9.9 Hz, 3H), 1.02 (d, *J* = 11.2 Hz, 2H). ^{13}C{1H} NMR (75 MHz, DMSO-d_6) δ 162.0, 139.8, 133.1, 129.6, 124.0, 123.2, 116.0, 113.3, 55.0, 51.8, 29.9, 25.8, 25.1; IR (KBr disk) 2927, 2849, 1727, 1477, 1462, 1447, 1435, 1323, 1219, 1166, 1152, 1125 cm^{-1}; HRMS (ESI) m/z: $[M + H]^+$ calculated for $C_{16}H_{19}BrN_2O_2^+$ 350.0703, found 350.0709. **16p** isolated yield: 7 mg, 0.020 mmol, 2.67%.

Preparation of methyl 1-benzyl-5-bromo-1*H*-indazole-3-carboxylate (15q).

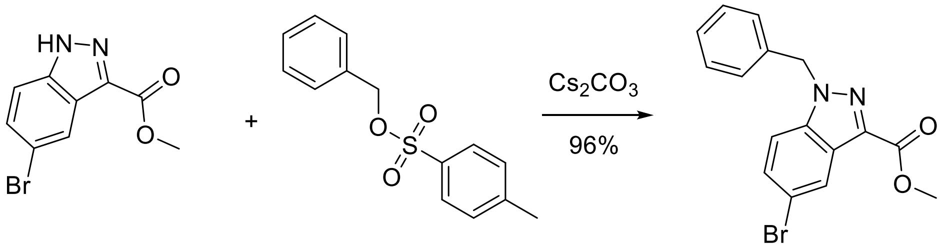

6 **14q** **15q**

The compound was prepared from **6** (0.194 g, 0.762 mmol) using a similar procedure as compound **15a** with 96% yield (253 mg, 0.733 mmol) as a white solid; melting point = 122 °C; ^{1}H NMR (300 MHz, DMSO-*d*$_6$) δ 8.22 (d, *J* = 1.8 Hz, 1H), 7.90 (d, *J* = 9.0 Hz, 1H), 7.65 (dd, *J* = 9.0, 1.9 Hz, 1H), 7.39 – 7.21 (m, 5H), 5.80 (s, 2H), 3.93 (s, 3H). ^{13}C{1H} NMR (75 MHz, DMSO-*d*$_6$) δ 161.9, 139.2, 136.2, 133.6, 129.9, 128.7, 127.9, 127.5, 124.4, 123.3, 116.2, 113.1, 52.9, 51.9; IR (KBr disk) 1735, 1455, 1325, 1298, 1220, 1153 cm^{-1}; HRMS (ESI) m/z: [M + H]$^+$ calculated for $C_{16}H_{13}BrN_2O_2^+$ 344.0233, found 344.0249. **16q** isolated yield: n.d.

Preparation of methyl 5-bromo-2-methyl-2*H*-indazole-3-carboxylate (16a).

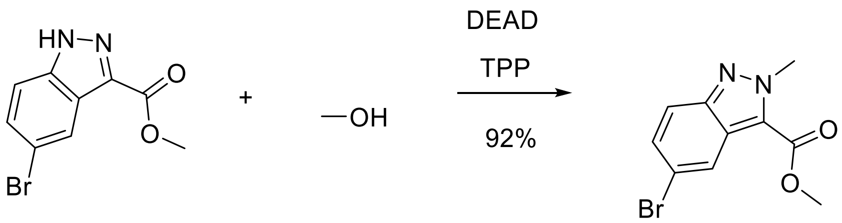

6 **13a** **16a**

To a solution of methanol (631 µL, 15.60 mmol) in THF (15 mL) was added triphenylphosphine (8186 mg, 31.2 mmol) and methyl 5-bromo-1*H*-indazole-3-carboxylate (**6**, 3980 mg, 15.60 mmol) at 0 °C, followed by adding DEAD (4941 µL, 31.2 mmol). The resulting mixture was stirred for 10 min at 0 °C and warmed to 50 °C and stirred for 2 h. After TLC showed completion, solvent was removed, the residue was purified by chromatography [silica gel (24 g), eluting with EA in hexane from 0–60%] to give methyl 5-bromo-2-methyl-2*H*-indazole-3-

carboxylate, **16a**, (3.850 g, 14.31 mmol, 92% yield) as a light pink solid; melting point = 110.7 °C; ^{1}H NMR (300 MHz, DMSO-d_6) δ 8.14 (d, *J* = 2.0 Hz, 1H), 7.77 (dd, *J* = 9.1, 0.7 Hz, 1H), 7.49 (dd, *J* = 9.0, 1.9 Hz, 1H), 4.42 (s, 3H), 3.98 (s, 3H); ^{13}C{1H} NMR (75 MHz, DMSO-d_6) δ 159.4, 144.7, 129.3, 123.6, 123.2, 122.8, 120.0, 118.0, 52.2, 41.4; IR (KBr disk) 1708, 1459, 1442, 1392, 1326, 1252, 1196 cm^{-1}; HRMS (ESI) m/z: [M + H]$^{+}$ calculated for $C_{10}H_{10}BrN_2O_2^{+}$ 268.9921, found 268.9918.

Preparation of methyl 5-bromo-2-ethyl-2*H*-indazole-3-carboxylate (16b).

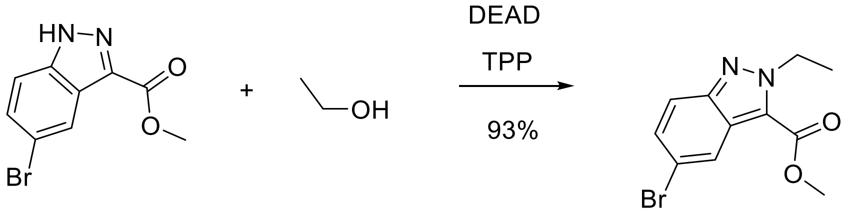

6 **13b** **16b**

The compound was prepared from **6** (1.384 g, 5.43 mmol) using a similar procedure as compound **16a** with 93% yield (1.422 g, 5.02 mmol) as a white solid; melting point = 98.8 °C; ^{1}H NMR (300 MHz, DMSO-d_6) δ 8.16 (dd, *J* = 1.9, 0.8 Hz, 1H), 7.80 (dd, *J* = 9.1, 0.8 Hz, 1H), 7.50 (dd, *J* = 9.1, 1.9 Hz, 1H), 4.86 (q, *J* = 7.2 Hz, 2H), 4.00 (s, 3H), 1.48 (t, *J* = 7.2 Hz, 3H). ^{13}C{1H} NMR (75 MHz, DMSO-d_6) δ 8.16 (dd, J = 1.9, 0.8 Hz, 1H), 7.80 (dd, J = 9.1, 0.8 Hz, 1H), 7.50 (dd, J = 9.1, 1.9 Hz, 1H), 4.86 (q, J = 7.2 Hz, 2H), 4.00 (s, 3H), 1.48 (t, J = 7.2 Hz, 3H); IR (KBr disk) 1708, 1459, 1436, 1325, 1200, 1120, 1069, 1030 cm^{-1}; HRMS (ESI) m/z: [M + H]$^{+}$ calculated for $C_{11}H_{11}BrN_2O_2^{+}$ 282.0077, found 282.0093.

Preparation of methyl 5-bromo-2-isopropyl-2*H*-indazole-3-carboxylate (16c).

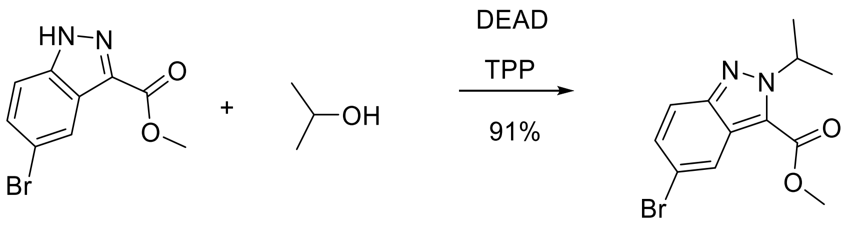

The compound was prepared from **6** (1061 mg, 4.16 mmol) using a similar procedure as compound **16a** with 91% yield (1.141 g, 3.84 mmol) as a white solid; melting point = 103.3 °C; ^{1}H NMR (300 MHz, DMSO-d_6) δ 8.17 (d, *J* = 1.9 Hz, 1H), 7.82 (d, *J* = 9.0 Hz, 1H), 7.49 (dd, *J* = 9.1, 1.9 Hz, 1H), 5.84 (hept, *J* = 6.6 Hz, 1H), 3.99 (s, 3H), 1.55 (d, *J* = 6.6 Hz, 6H). ^{13}C{1H} NMR (75 MHz, DMSO-d_6) δ 160.0, 145.4, 129.8, 124.3, 123.7, 122.9, 120.9, 118.5, 54.2, 52.7, 23.1; IR (KBr disk) 1703, 1455, 1433, 1382, 1248, 1200, 1168 cm^{-1}; HRMS (ESI) m/z: [M + H]$^+$ calculated for $C_{12}H_{13}BrN_2O_2^+$ 296.0233, found 296.0232.

Preparation of methyl 5-bromo-2-propyl-2*H*-indazole-3-carboxylate (16d).

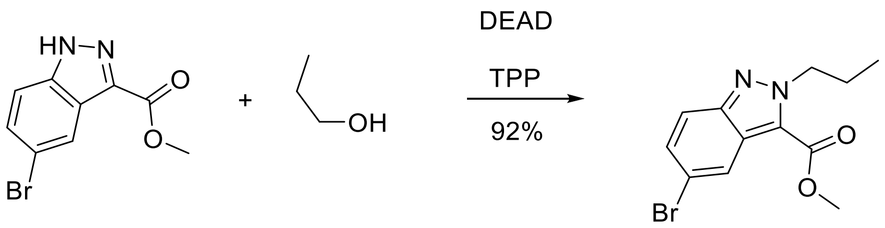

The compound was prepared from **6** (1,061 mg, 4.16 mmol) using a similar procedure as compound **16a** with 92% yield (1.141 g, 3.84 mmol) as a white solid; melting point = 77.7 °C; ^{1}H NMR (300 MHz, DMSO-d_6) δ 8.17 (dd, *J* = 1.9, 0.8 Hz, 1H), 7.80 (dd, *J* = 9.1, 0.8 Hz, 1H), 7.50 (dd, *J* = 9.1, 1.9 Hz, 1H), 4.80 (t, *J* = 7.2 Hz, 2H), 1.92 (h, *J* = 7.4 Hz, 2H), 0.87 (t, *J* = 7.4 Hz, 3H); ^{13}C{1H} NMR (75 MHz, DMSO-d_6) δ 159.4, 144.9, 129.5, 123.8,

123.1, 122.9, 120.3, 118.1, 54.6, 52.3, 23.5, 10.8; IR (KBr disk) 1701, 1462, 1325, 1218, 1202, 1185 cm^{-1}; HRMS (ESI) m/z: $[M + H]^+$ calculated for $C_{12}H_{13}BrN_2O_2^+$ 296.0233, found 296.0245.

Preparation of methyl (*S*)-5-bromo-2-(sec-butyl)-2*H*-indazole-3-carboxylate (16e).

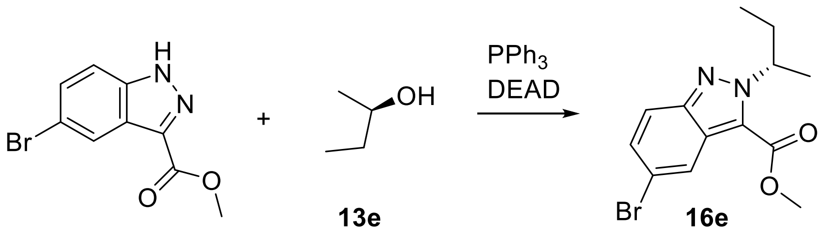

The compound was prepared from **6** (860 mg, 3.37 mmol) using a similar procedure as compound **16a** 97% yield as a white solid (1.020 g, 97% yield); m.p. = 70.3 °C; ^{1}H NMR (300 MHz, DMSO-d_6) δ 8.17 (d, J = 1.9, 0.8 Hz, 1H), 7.82 (d, J = 9.1, 0.8 Hz, 1H), 7.49 (dd, J = 9.1, 1.9 Hz, 1H), 5.78 – 5.61 (m, 1H), 3.99 (s, 3H), 2.13 – 1.79 (m, 2H), 1.54 (d, J = 6.6 Hz, 3H), 0.70 (t, J = 7.4 Hz, 3H); ^{13}C{H} NMR (75 MHz, DMSO-d_6) δ 159.7, 145.2, 129.4, 123.6, 123.3, 120.4, 118.1, 58.9, 52.3, 29.6, 20.9, 10.3; IR (KBr disk) 2981, 2965, 2937, 2875, 1708, 1490, 1462, 1454, 1380, 1255, 1247, 1209, 1195 cm^{-1}; HRMS (ESI) m/z: $[M^+]$ calculated for $C_{13}H_{15}BrN_2O_2^+$ 310.0390, found 310.0399.

Preparation of methyl 5-bromo-2-isobutyl-2*H*-indazole-3-carboxylate (16f).

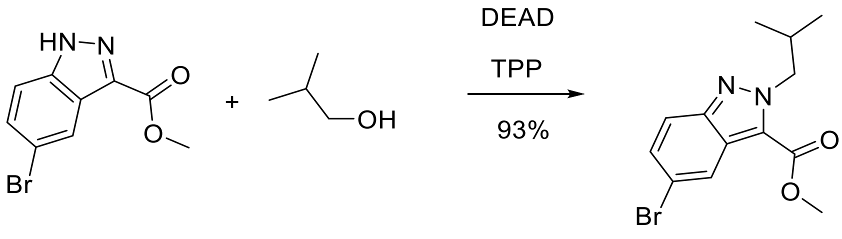

6 **13f** **16f**

The compound was prepared from **6** (860 mg, 3.37 mmol) using a similar procedure as compound **16a** with 93% yield (981 mg, 3.15 mmol) as a white solid; melting point = 100.6 °C; ^{1}H NMR (300 MHz, DMSO-d_6) δ 8.17 (d,

J = 1.9, 0.8 Hz, 1H), 7.80 (d, *J* = 9.1, 0.7 Hz, 1H), 7.50 (dd, *J* = 9.1, 1.9 Hz, 1H), 4.67 (d, *J* = 7.3 Hz, 2H), 3.99 (s, 3H), 2.35 – 2.18 (m, 1H), 0.87 (d, *J* = 6.7 Hz, 6H). ^{13}C{1H} NMR (75 MHz, DMSO-d_6) δ 159.5, 144.9, 129.5, 123.8, 123.2, 123.1, 120.3, 118.2, 59.7, 52.3, 29.6, 19.6; IR (KBr disk) 2953, 2909, 2871, 1705, 1459, 1323, 1254, 1205, 1192, 1082 cm^{-1}; HRMS (ESI) m/z: [M + H]$^+$ calculated for $C_{13}H_{15}BrN_2O_2^+$ 310.0399, found 310.0396.

Preparation of methyl 5-bromo-2-(2-methoxyethyl)-2*H*-indazole-3-carboxylate (16g).

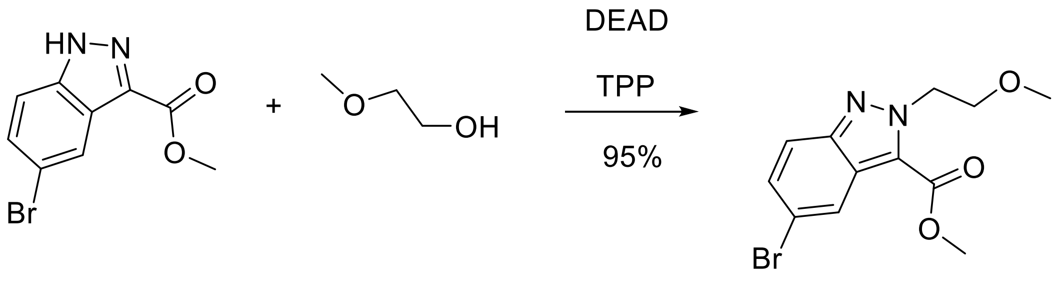

6 **13g** **16g**

The compound was prepared from **6** (838 mg, 3.29 mmol) using similar procedure as compound **16a** with 95% yield (981 mg, 3.13 mmol) as a white solid; melting point = 74 °C; ^{1}H NMR (300 MHz, DMSO-d_6) δ 8.17 (dd, *J* = 1.9, 0.8 Hz, 1H), 7.80 (dd, *J* = 9.1, 0.8 Hz, 1H), 7.51 (dd, *J* = 9.1, 1.9 Hz, 1H), 5.04 (t, *J* = 5.5 Hz, 2H), 4.00 (s, 3H), 3.85 (t, *J* = 5.5 Hz, 2H), 3.21 (s, 3H). ^{13}C{1H} NMR (75 MHz, DMSO-d_6) δ 159.5, 145.1, 129.6, 123.8, 123.5, 123.1, 120.3, 118.2, 70.5, 58.0, 52.3, 52.3; IR (KBr disk) 1705, 1487, 1459, 1435, 1407, 1326, 1304, 1256, 1220, 1205 cm^{-1}; HRMS (ESI) m/z: [M + H]$^+$ calculated for $C_{12}H_{13}BrN_2O_3^+$ 312.0182, found 312.0181.

Preparation of methyl 5-bromo-2-cyclopentyl-2*H*-indazole-3-carboxylate (16h).

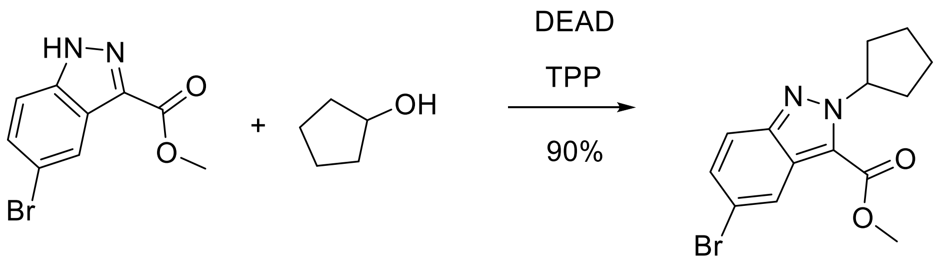

6 **13h** **16h**

The compound was prepared from **6** (740 mg, 2.90 mmol) using a similar procedure as compound **16a** with 90% yield (845 mg, 2.61 mmol) as a white solid; melting point = 101.8 °C; ^{1}H NMR (300 MHz, DMSO-d_6) δ 8.17 (d, J = 1.9, 0.8 Hz, 1H), 7.81 (dd, J = 9.1, 0.8 Hz, 1H), 7.49 (dd, J = 9.1, 1.9 Hz, 1H), 6.04 – 5.88 (m, 1H), 4.00 (s, 3H), 2.29 – 2.17 (m, 2H), 2.16 – 2.04 (m, 2H), 1.98 – 1.84 (m, 2H), 1.79 – 1.63 (m, 2H). ^{13}C{1H} NMR (75 MHz, DMSO-d_6) δ 159.6, 144.8, 129.4, 124.0, 123.1, 120.5, 118.0, 62.6, 52.3, 33.2, 24.5; IR (KBr disk) 2952, 2866, 1712, 1490, 1466, 1457, 1434, 1376, 1324, 1308, 1257, 1213, 1098 cm^{-1}; HRMS (ESI) m/z: $[M + H]^+$ calculated for $C_{14}H_{15}BrN_2O_2^+$ 322.0390, found 322.0408.

Preparation of methyl 5-bromo-2-(1-(*tert*-butoxycarbonyl)pyrrolidin-3-yl)-2*H*-indazole-3-carboxylate (16i).

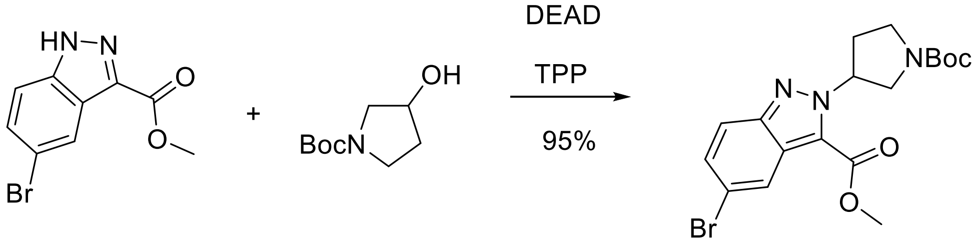

6 **13i** **16i**

The compound was prepared from **6** (341 mg, 1.335 mmol) as a mixture of rotamers using a similar procedure as compound **16a** with 95% yield (541 mg, 1.275 mmol) as a white solid; melting point = 148.8 °C; ^{1}H NMR (300 MHz, DMSO-d_6) δ 8.19 (d, J = 1.9, 0.7 Hz, 1H), 7.81 (d, J = 9.1, 0.8 Hz, 1H), 7.51 (dd, J = 9.1, 1.9 Hz, 1H), 6.12

(s, 1H), 4.01 (s, 3H), 3.90 – 3.79 (m, 1H), 3.72 (dd, J = 12.1, 3.2 Hz, 1H), 3.57 (s, 1H), 3.49 (s, 1H), 2.45 (d, J = 8.3 Hz, 2H), 1.41 (dd, J = 6.9, 2.6 Hz, 9H). ^{13}C{1H} NMR (75 MHz, DMSO-d_6) δ 159.6, 153.5, 153.4, 144.9, 129.7, 123.9, 123.4, 123.1, 120.5, 118.4, 78.6, 61.2, 60.5, 52.4, 51.8, 44.9, 44.7, 31.7, 30.9, 28.1; IR (KBr disk) 1708, 1679, 1403, 1375, 1243, 1205, 1189, 1162 cm^{-1}; HRMS (ESI) m/z: [M + Na]$^+$ calculated for $C_{18}H_{22}BrN_3O_4Na^+$ 446.0691, found 446.0705.

Preparation of methyl (*S*)-5-bromo-2-(tetrahydrofuran-3-yl)-2*H*-indazole-3-carboxylate (16j).

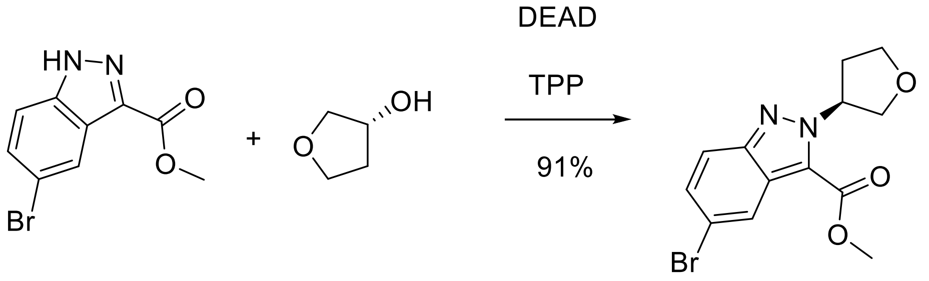

6 **13j** **16j**

The compound was prepared from **6** (724 mg, 2.84 mmol) using a similar procedure as compound **16a** with 91% yield (841 mg, 2.59 mmol) as a white solid; melting point = 141.2 °C; ^{1}H NMR (300 MHz, DMSO-d_6) δ 8.17 (d, J = 1.9, 0.8 Hz, 1H), 7.82 (d, J = 9.1, 0.7 Hz, 1H), 7.50 (dd, J = 9.1, 1.9 Hz, 1H), 6.22 – 6.11 (m, 1H), 4.18 – 4.05 (m, 2H), 4.02 (d, J = 3.2 Hz, 1H), 3.99 (s, 3H), 3.95 – 3.85 (m, 1H), 2.59 – 2.52 (m, 1H), 2.47 (d, J = 2.4 Hz, 1H). ^{13}C{1H} NMR (75 MHz, DMSO-d_6) δ 159.7, 144.8, 129.6, 124.1, 123.4, 123.1, 120.6, 118.3, 73.1, 67.5, 62.2, 52.4, 32.4; IR (KBr disk) 1705, 1455, 1257, 1215, 1092, 1067 cm^{-1}; HRMS (ESI) m/z: [M + H]$^+$ calculated for $C_{13}H_{13}BrN_2O_3^+$ 324.0182, found 324.0182.

Preparation of methyl (*R*)-5-bromo-2-(tetrahydrofuran-3-yl)-2*H*-indazole-3-carboxylate (16k).

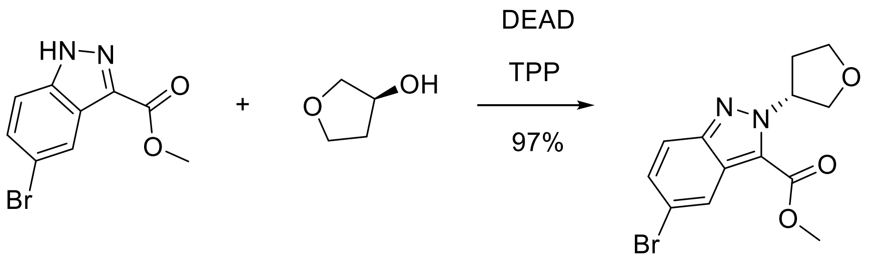

6 **13k** **16k**

The compound was prepared from **6** (724 mg, 2.84 mmol) using a similar procedure as compound **16a** with 97% yield (898 mg, 2.76 mmol) as a white solid; melting point = 126.2 °C; ^{1}H NMR (300 MHz, DMSO-d_6) δ 8.18 (d, *J* = 1.9, 0.7 Hz, 1H), 7.83 (d, *J* = 9.1, 0.8 Hz, 1H), 7.51 (dd, *J* = 9.1, 1.9 Hz, 1H), 6.22 – 6.12 (m, 1H), 4.21 – 4.06 (m, 2H), 4.03 (d, *J* = 3.0 Hz, 1H), 4.00 (s, 3H), 3.96 – 3.86 (m, 1H), 2.59 – 2.54 (m, 1H), 2.49 – 2.41 (m, 1H); ^{13}C{1H} NMR (75 MHz, DMSO-d_6) δ 159.6, 144.8, 129.6, 124.0, 123.3, 123.1, 120.5, 118.3, 73.1, 67.5, 62.2, 52.3, 32.4; IR (KBr disk) 1701, 1455, 1256, 1213, 1090, 1067, 1036 cm^{-1}; HRMS (ESI) m/z: $[M + H]^+$ calculated for $C_{13}H_{13}BrN_2O_3^+$ 324.0182, found 324.0182.

Preparation of methyl 5-bromo-2-(cyclobutylmethyl)-2*H*-indazole-3-carboxylate (16l).

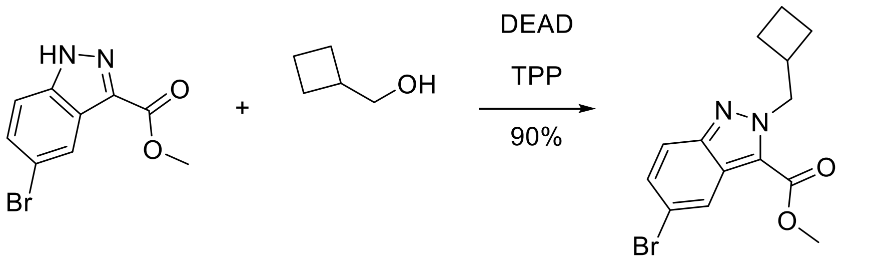

6 **13l** **16l**

The compound was prepared from **6** (740 mg, 2.90 mmol) using a similar procedure as compound **16a** with 90% yield (844 mg, 2.61 mmol) as a white solid; m.p. = °87.5; ^{1}H NMR (300 MHz, DMSO-d_6) δ 8.16 (d, *J* = 1.9, 0.8 Hz, 1H), 7.79 (d, *J* = 9.0, 0.7 Hz, 1H), 7.49 (dd, *J* = 9.1, 1.9 Hz, 1H), 4.88 (d, *J* = 7.3 Hz, 2H), 3.99 (s, 3H), 2.90 (p, *J* = 7.4 Hz, 1H), 1.98 – 1.90 (m, 2H), 1.88 – 1.78 (m, 4H); ^{13}C{H} NMR (75 MHz, DMSO-d_6) δ 159.5, 144.9,

129.4, 123.7, 123.0, 122.7, 120.2, 118.1, 57.3, 52.2, 35.6, 25.1, 17.7; IR (KBr disk) 2942, 1705, 1462, 1321, 1252, 1205 cm^{-1}; HRMS (ESI) m/z: $[M^+]$ calculated for $C_{14}H_{15}BrN_2O_2^+$ 322.0390, found 322.0398.

Preparation of methyl 5-bromo-2-(1-(*tert*-butoxycarbonyl)azetidin-3-yl)-2*H*-indazole-3-carboxylate (16m).

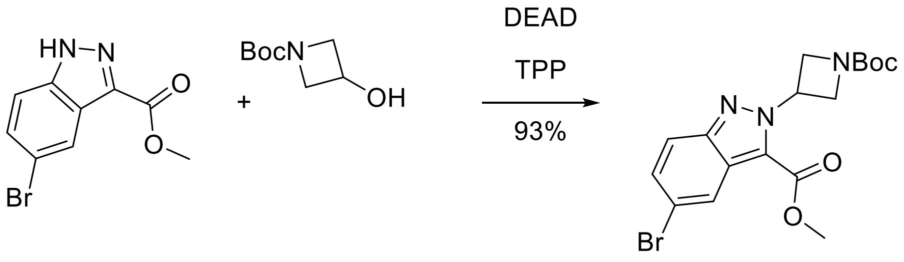

6 **13m** **16m**

The compound was prepared as a mixture of rotamers from **6** (368 mg, 1.443 mmol) using a similar procedure as compound **16a** with 93% yield (551 mg, 1.343 mmol) as a white solid; ^{1}H NMR (300 MHz, DMSO-d_6) δ 8.17 (s, 1H), 7.88 (d, J = 8.8 Hz, 1H), 7.54 (d, J = 9.1 Hz, 1H), 6.22 – 6.08 (m, 1H), 4.41 (q, J = 7.7 Hz, 4H), 4.13 (q, J = 7.0 Hz, 1H), 3.98 (s, 3H), 1.41 (d, J = 1.2 Hz, 9H); ^{13}C{H} NMR (75 MHz, DMSO-d_6) δ 159.3, 155.5, 155.4, 153.5, 145.0, 130.0, 124.0, 123.9, 123.0, 120.6, 118.5, 79.0, 79.0, 65.9, 64.0, 52.4, 50.9, 28.02, 27.96, 14.0; IR (KBr disk) 3060, 2976, 2966, 2930, 2892, 1748, 1709, 1494, 01468, 1439, 1394, 1368, 1332, 1307, 1259, 1248, 1215, 1171, 1157, 1140, 1101, 1084, 1035, 1003 cm^{-1}; HRMS (ESI) m/z: $[M\text{-}C_4H_8{}^1 + H]^+$ calculated for $C_{13}H_{13}BrN_3O_4^+$ 354.0084, found 354.0094.

Preparation of methyl 5-bromo-2-(cyclopentylmethyl)-2*H*-indazole-3-carboxylate (16n).

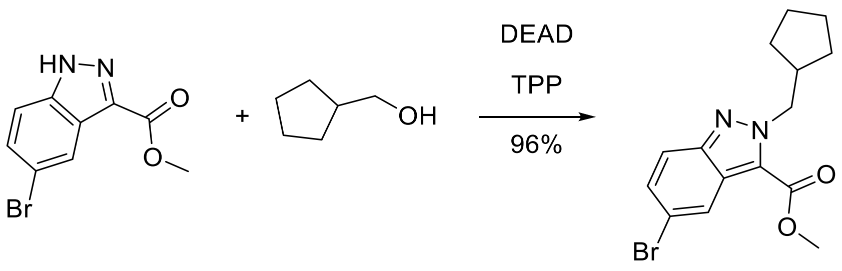

6 **13n** **16n**

The compound was prepared from **6** (637 mg, 2.496 mmol) using a similar procedure as compound **16a** with 96% yield (810 mg, 2.42 mmol) as a white solid; m.p. = 101.7 °C; ^{1}H NMR (300 MHz, DMSO-d_6) δ 8.17 (d, J = 1.9 Hz, 1H), 7.80 (d, J = 9.1 Hz, 1H), 7.50 (dd, J = 9.1, 1.9 Hz, 1H), 4.78 (d, J = 7.6 Hz, 2H), 3.99 (s, 3H), 2.58 – 2.52 (m, 1H), 1.67 – 1.45 (m, 6H), 1.39 – 1.24 (m, 2H); ^{13}C{1H} NMR (75 MHz, DMSO-d_6) δ 159.6, 144.9, 129.5, 123.9, 123.2, 122.9, 120.4, 118.2, 57.1, 52.3, 29.4, 24.4; IR (KBr disk) 2946, 2868, 1705, 1485, 1459, 1420, 1325, 1302, 1254, 1203 cm^{-1}; HRMS (ESI) m/z: [M^{+}] calculated for $C_{15}H_{17}BrN_2O_2^{+}$ 336.0546, found 336.0560.

Preparation of methyl 5-bromo-2-((tetrahydrofuran-3-yl)methyl)-2*H*-indazole-3-carboxylate (16o).

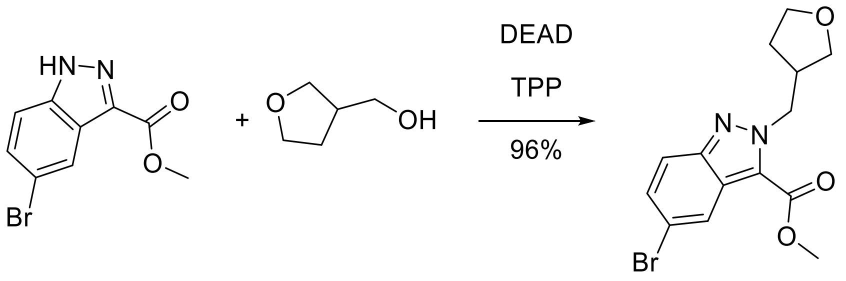

6 **13o** **16o**

The compound was prepared from **6** (624 mg, 2.448 mmol) using a similar procedure as compound **16a** with 98% yield (810 mg, 2.388 mmol) as a white solid; m.p. = 69.1 °C; ^{1}H NMR (300 MHz, DMSO-d_6) δ 8.18 (d, 1H), 7.82 (d, J = 9.1, 0.8 Hz, 1H), 7.51 (dd, J = 9.1, 1.9 Hz, 1H), 4.93 – 4.73 (m, 2H), 4.00 (s, 3H), 3.81 (td, J = 8.1, 5.6 Hz, 1H), 3.69 – 3.60 (m, 2H), 3.60 – 3.52 (m, 1H), 2.91 (h, J = 13.2, 6.8, 6.3 Hz, 1H), 1.99 – 1.85 (m, 1H), 1.76 – 1.58 (m, 1H); ^{13}C{H} NMR (75 MHz, DMSO-d_6) δ 159.5, 145.0, 129.7, 123.8, 123.2, 120.4, 118.3, 69.9, 66.7, 55.2, 52.4, 29.1; IR (KBr disk) 2953, 2853, 1708, 1492, 1459, 1254, 1205 cm^{-1}; HRMS (ESI) m/z: [M + H^{+}] calculated for $C_{14}H_{16}BrN_2O_3^{+}$ 339.0339, found 339.0367.

Preparation of methyl 5-bromo-2-(cyclohexylmethyl)-2*H*-indazole-3-carboxylate (16p).

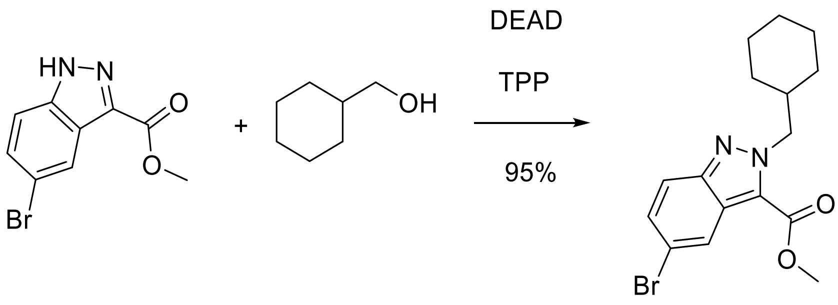

6 **13p** **16p**

The compound was prepared from **6** (558 mg, 2.189 mmol) using a similar procedure as compound **16a** with 95% yield (730 mg, 2.078 mmol) as a white solid; melting point = 98.8 °C; ^{1}H NMR (300 MHz, DMSO-d_6) δ 8.17 (d, *J* = 1.9, 0.8 Hz, 1H), 7.79 (d, *J* = 9.1, 0.8 Hz, 1H), 7.49 (dd, *J* = 9.1, 1.9 Hz, 1H), 4.70 (d, *J* = 7.2 Hz, 2H), 3.99 (s, 3H), 2.03 – 1.89 (m, 1H), 1.61 (d, *J* = 17.2 Hz, 3H), 1.47 (d, *J* = 11.8 Hz, 2H), 1.20 – 1.08 (m, 3H), 1.04 (d, *J* = 11.5 Hz, 2H); ^{13}C{1H} NMR (75 MHz, DMSO-d_6) δ 159.6, 159.5, 144.9, 133.4, 129.6, 123.8, 123.2, 120.3, 118.2, 58.5, 52.3, 29.9, 25.8, 25.1; IR (KBr disk) 2916, 2847, 1701, 1466, 1446, 1323, 1209 cm^{-1}; HRMS (ESI) m/z: $[M + H]^+$ calculated for $C_{16}H_{19}BrN_2O_2^+$ 350.0703, found 350.0716.

Preparation of methyl 2-benzyl-5-bromo-2*H*-indazole-3-carboxylate (16q).

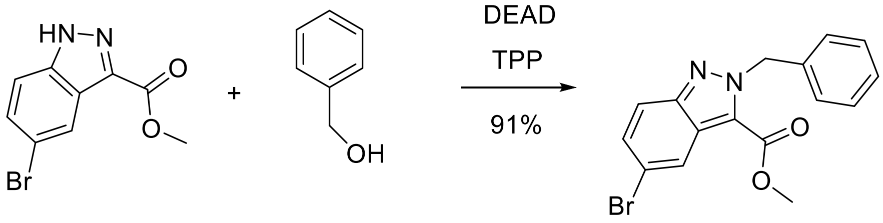

6 **13q** **16q**

The compound was prepared from **6** (500 mg, 4.62 mmol) using a similar procedure as compound **16a** in 91% yield (723 mg, 2.094 mmol) as a white solid; melting point = 122.6 °C; ^{1}H NMR (300 MHz, DMSO-d_6) δ 8.18 (d, *J* = 1.9, 0.8 Hz, 1H), 7.82 (d, *J* = 9.1, 0.8 Hz, 1H), 7.52 (dd, *J* = 9.1, 1.9 Hz, 1H), 7.39 – 7.20 (m, 5H), 6.07 (s, 2H), 3.98 (s, 3H); ^{13}C{1H} NMR (75 MHz, DMSO-d_6) δ 159.5, 145.4, 136.3, 129.9, 128.6, 127.9, 127.5, 123.9,

123.3, 123.1, 120.5, 118.5, 56.1, 52.4; IR (KBr disk) 1708, 1470, 1300, 1256, 1216, 1093 cm^{-1}; HRMS (ESI) m/z: $[M + H]^+$ calculated for $C_{16}H_{13}BrN_2O_2^+$ 344.0233, found 344.0249.

Preparation of methyl 2-ethyl-2*H*-indazole-7-carboxylate (19).

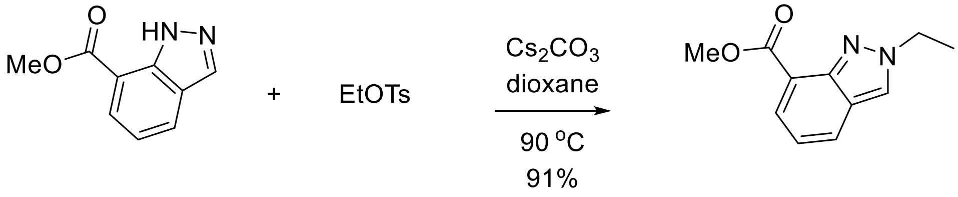

18 **12** **19**

This compounds was prepared from methyl 1*H*-indazole-7-carboxylate (**18**, 176 mg, 1.0 mmol) using a similar procedure as **15a** in 93% yield (190 mg, 0.93 mmol) as a viscous oil; ^{1}H NMR (300 MHz, DMSO-d_6) δ 8.57 (s, 1H), 8.02 (d, *J* = 8.3 Hz, 1H), 7.92 (d, *J* = 7.1 Hz, 1H), 7.14 (t, *J* = 7.7 Hz, 1H), 4.51 (q, *J* = 7.3 Hz, 2H), 3.88 (s, 3H), 1.52 (t, *J* = 7.3 Hz, 3H); ^{13}C{1H} NMR (75 MHz, DMSO-d_6) δ 166.1, 144.8, 129.7, 126.6, 124.2, 123.2, 119.9, 118.5, 51.8, 48.0, 15.7; IR (KBr disk) 1729, 1606, 1575, 1505, 1455, 1435, 1375, 1304, 1271, 1235, 1202, 1176, 1138, 1116, 1065, 1032 cm^{-1}; HRMS (ESI) m/z: $[M + H]^+$ calculated for $C_{11}H_{13}N_2O_2^+$ 205.0977, found 205.0923.

Preparation of methyl 1-ethyl-1*H*-indazole-7-carboxylate (20).

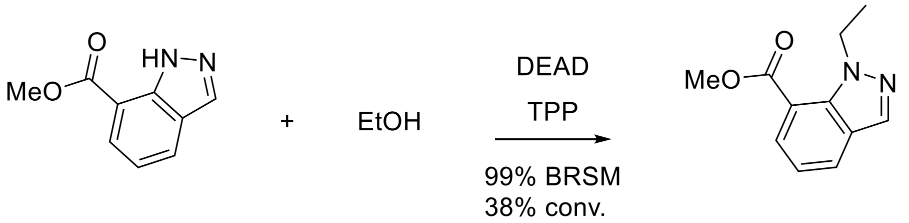

18 **20**

This compound was prepared from methyl 1*H*-indazole-7-carboxylate (**18**, 1.912 g, 10.85 mmol) using a similar procedure as **16a** in >99% (852 mg, 4.17 mmol) yield as a viscous oil based on recovered starting material **18** (1.190 g, 6.7 mmol); ^{1}H NMR (300 MHz, DMSO-d_6) δ 8.25 (s, 1H), 8.05 (d, *J* = 8.0 Hz, 1H), 7.87 (d, *J* = 7.3 Hz, 1H), 7.23 (t, *J* = 7.6 Hz, 1H), 4.59 (q, *J* = 7.1 Hz, 2H), 3.94 (s, 3H), 1.30 (t, *J* = 7.2 Hz, 3H); ^{13}C{1H} NMR (75 MHz, DMSO-d_6) δ 166.4, 135.2, 133.9, 129.4, 126.3, 126.1, 119.7, 115.0, 52.4, 46.6, 15.1; IR (KBr disk) 1710,

1617, 1555, 1522, 1436, 1380, 1318, 1280, 1203, 1140, 1039, 1005 cm^{-1}; HRMS (ESI) m/z: $[M + H]^+$ calculated for $C_{11}H_{13}N_2O_2^+$ 205.0977, found 205.0993.

Preparation of 1-ethyl-1*H*-indazole-3-carbonitrile (22), method A.

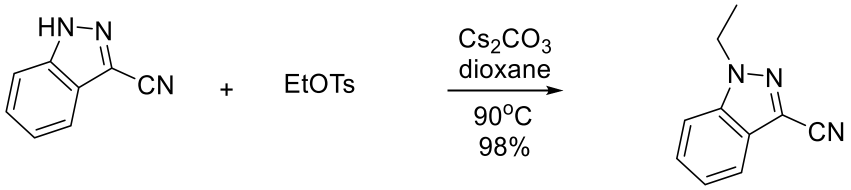

21 **12** **22**

This compound was prepared from 1*H*-indazole-3-carbonitrile (**21**, 143 mg, 1 mmol) using a similar procedure to **15a** in 98% yield (167 mg, 0.98 mmol) as a viscous oil; 1H NMR (300 MHz, DMSO-d_6) δ 7.92 (dd, *J* = 20.4, 8.5 Hz, 2H), 7.59 (t, *J* = 7.7 Hz, 1H), 7.42 (t, *J* = 8.0 Hz, 1H), 4.60 (q, *J* = 6.9 Hz, 2H), 1.45 (t, *J* = 7.2 Hz, 3H); $^{13}C\{1H\}$ NMR (75 MHz, DMSO-d_6) δ 138.9, 127.7, 124.5, 123.9, 118.8, 115.8, 113.8, 111.3, 44.6, 14.5; IR (KBr disk) 2230, 1466, 1352, 1235, 1223 cm^{-1}; HRMS (ESI) m/z: $[M + H]^+$ calculated for $C_{10}H_{10}N_3^+$ 172.0875, found 172.0775.

Preparation of 1-ethyl-1*H*-indazole-3-carbonitrile (22), method B.

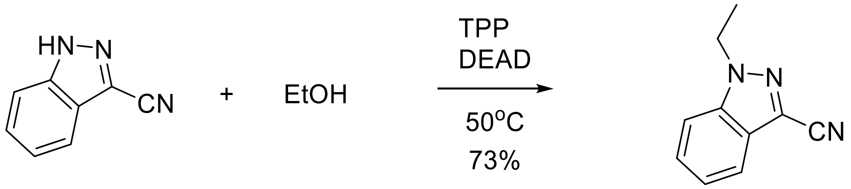

21 **22**

This compound was prepared from 1*H*-indazole-3-carbonitrile (**21**, 777 mg, 5.43 mmol) using a similar procedure to **16a** in 73% yield (679 mg, 3.97 mmol) as a viscous oil; ^1H NMR (300 MHz, DMSO-d_6) δ 7.92 (dd, *J* = 20.4, 8.5 Hz, 2H), 7.59 (t, *J* = 7.7 Hz, 1H), 7.42 (t, *J* = 8.0 Hz, 1H), 4.60 (q, *J* = 6.9 Hz, 2H), 1.45 (t, *J* = 7.2 Hz, 3H); $^{13}C\{1H\}$ NMR (75 MHz, DMSO-d_6) δ 138.9, 127.7, 124.5, 123.9, 118.8, 115.8, 113.8, 111.3, 44.6, 14.5; IR (KBr

disk) 2230, 1466, 1352, 1235, 1223 cm^{-1}; HRMS (ESI) m/z: $[M + H]^+$ calculated for $C_{10}H_{10}N_3^+$ 172.0875, found 172.0779.

Crude LCMS data. Condition A (top), Condition B (bottom). *N*[1] Product has shorter Rt for a-q.

a (zoom)

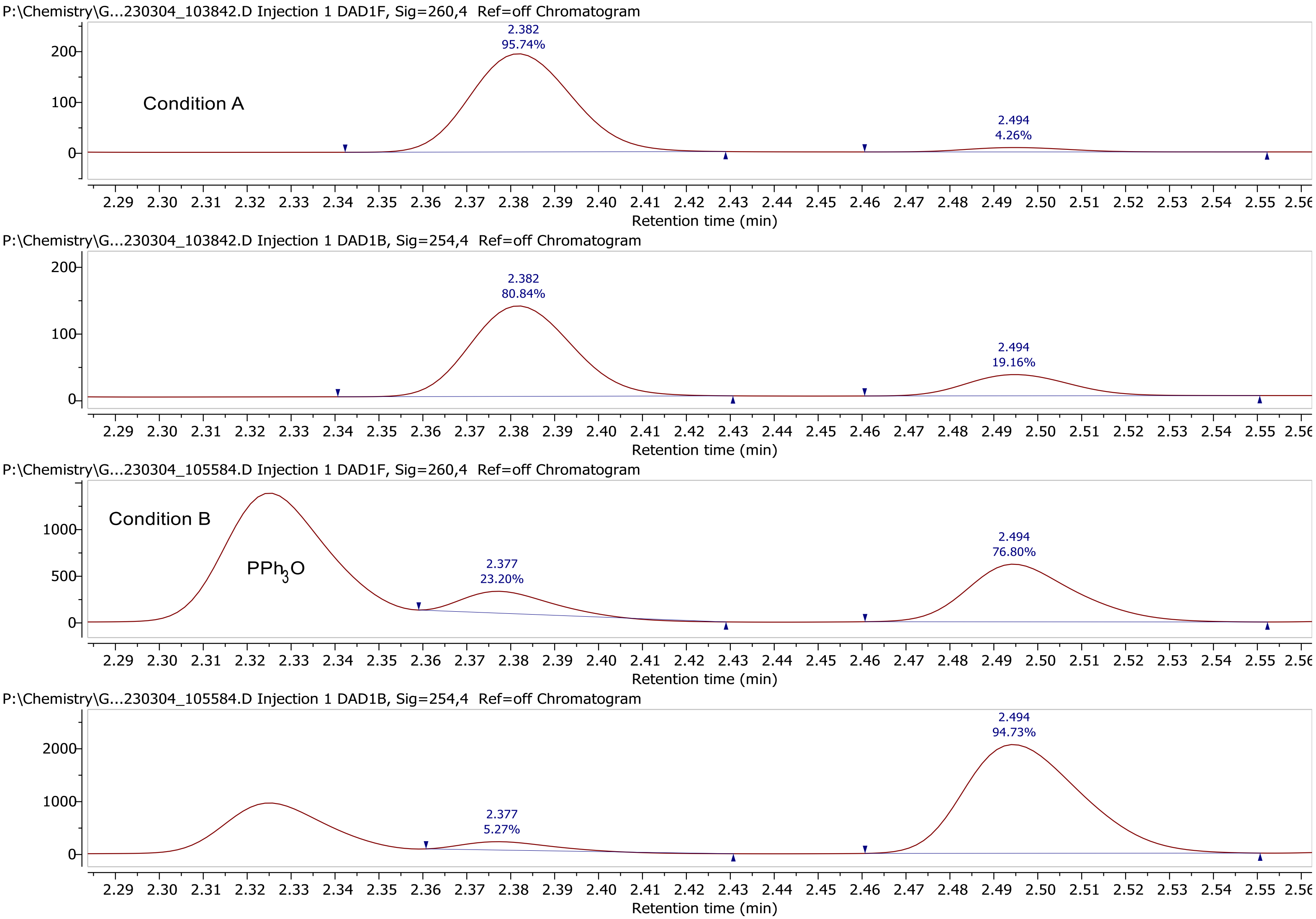

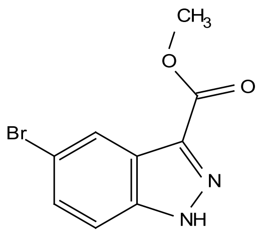

6

Condition A
R=CH_3

Condition B
R=CH_3

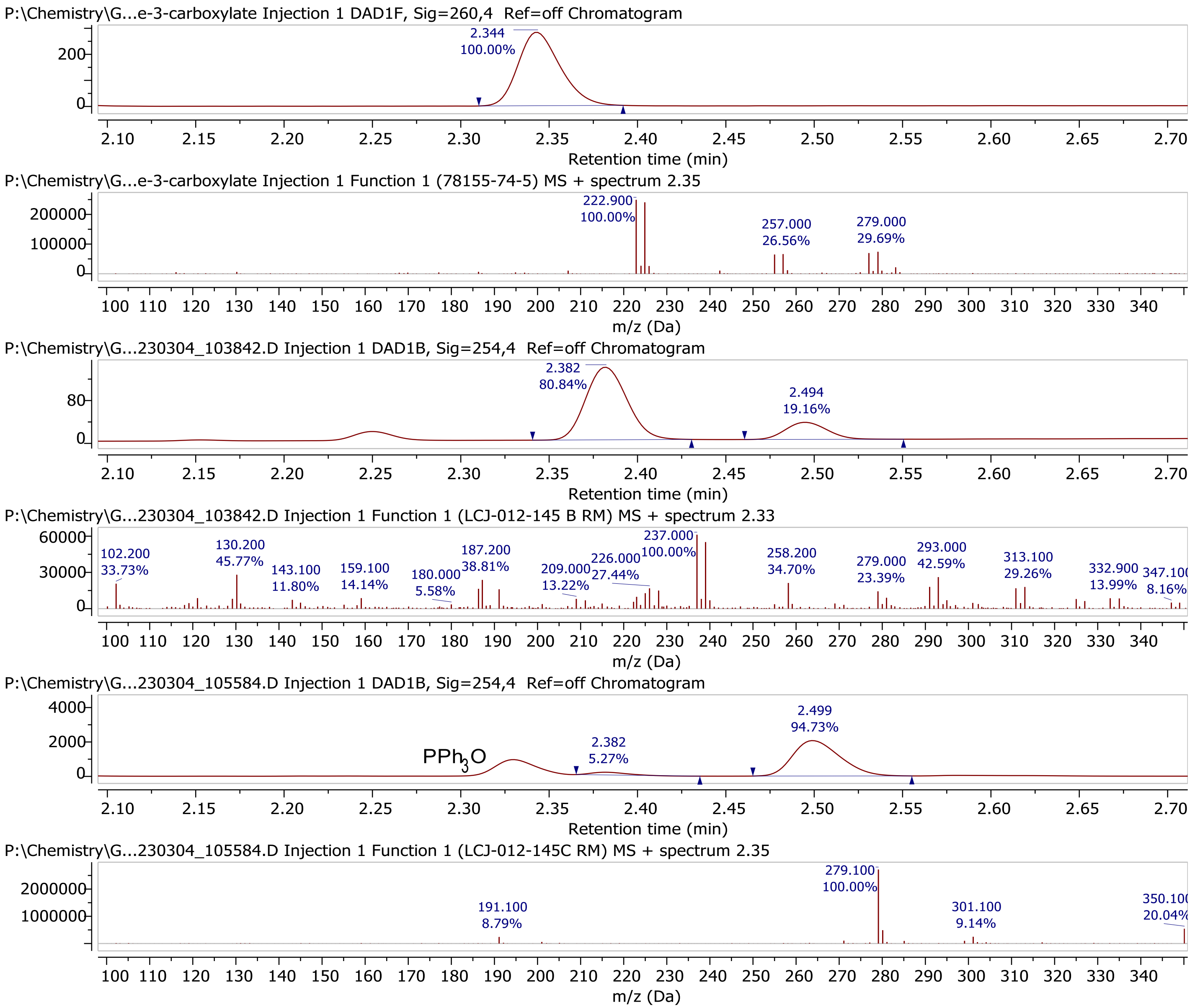

a: R = CH_3, 6 versus reaction mixtures. Mass spectra for reaction mixtures at 2.344 minutes for each condition.

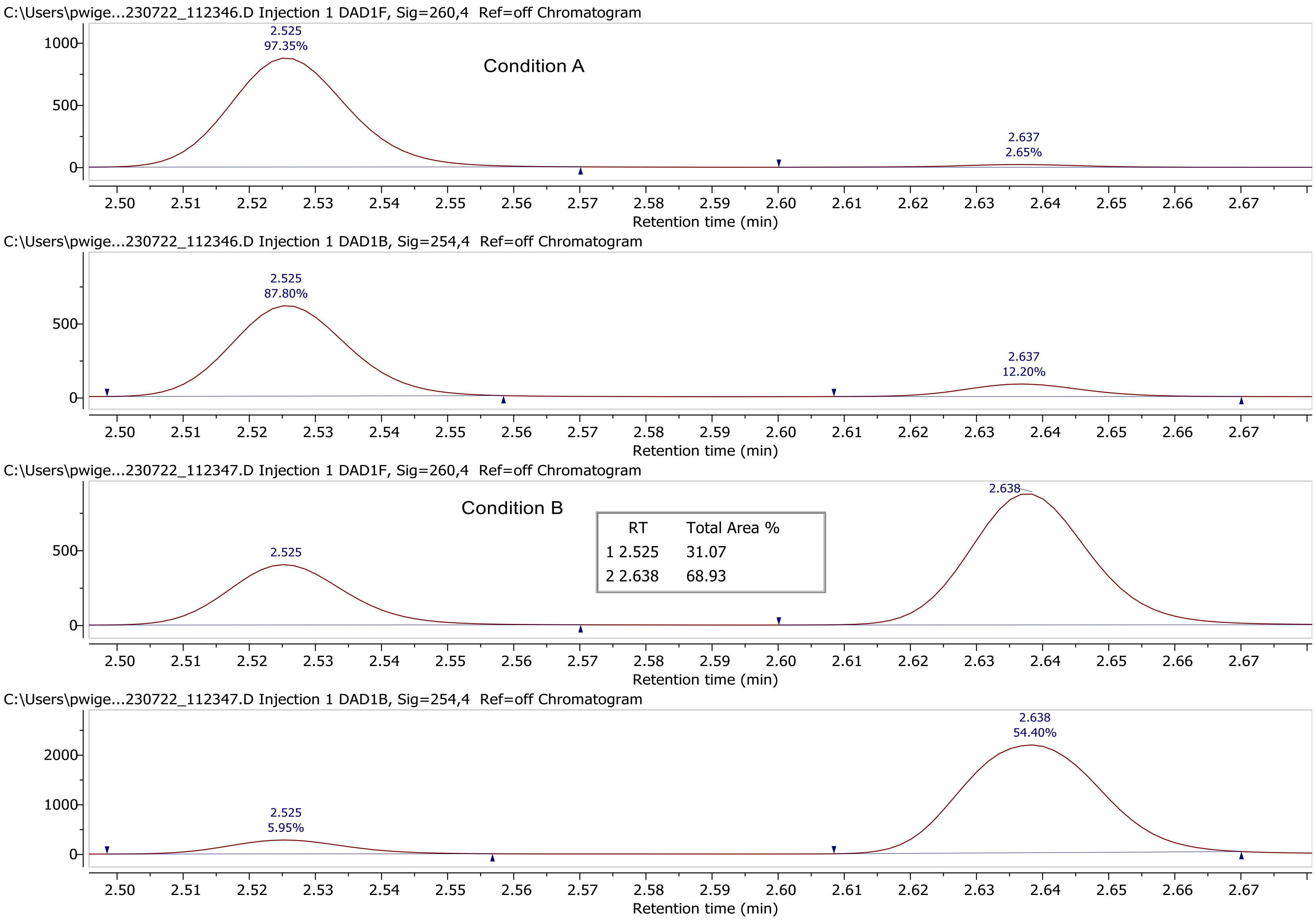
C:\Users\pwige...230722_112346.D Injection 1 DAD1F, Sig=260,4 Ref=off Chromatogram
Condition A
2.525
97.35%
2.637
2.65%
C:\Users\pwige...230722_112346.D Injection 1 DAD1B, Sig=254,4 Ref=off Chromatogram
2.525
87.80%
2.637
12.20%
C:\Users\pwige...230722_112347.D Injection 1 DAD1F, Sig=260,4 Ref=off Chromatogram
Condition B
RT Total Area %
1 2.525 31.07
2 2.638 68.93
2.525
2.638
C:\Users\pwige...230722_112347.D Injection 1 DAD1B, Sig=254,4 Ref=off Chromatogram
2.525
5.95%
2.638
54.40%
Retention time (min)

b (zoom)

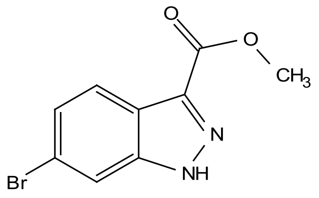

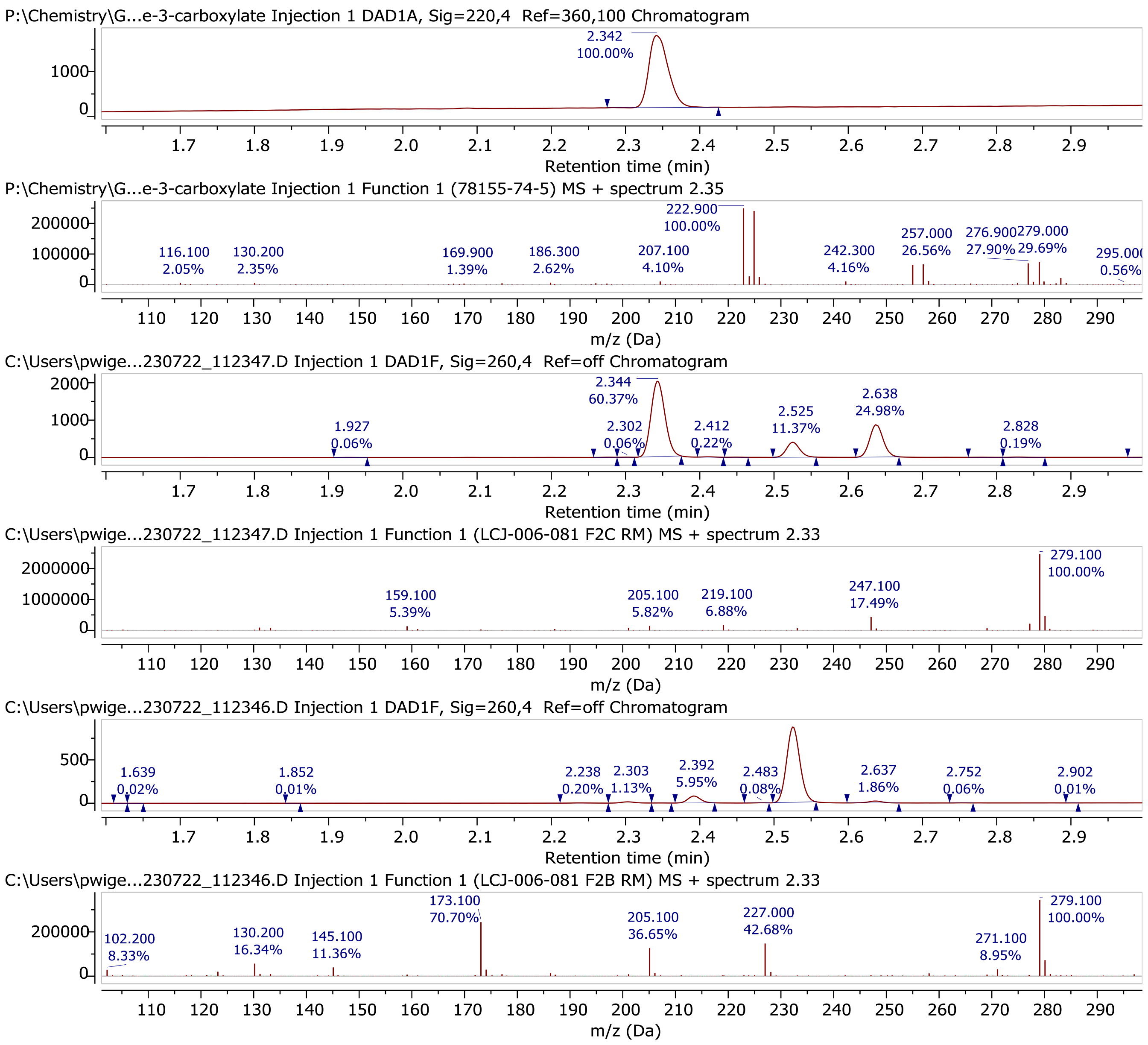

Condition A

Condition B

b: R = Et, 6 versus reaction mixtures. Mass spectra for reaction mixtures at 2.344 minutes for each condition.

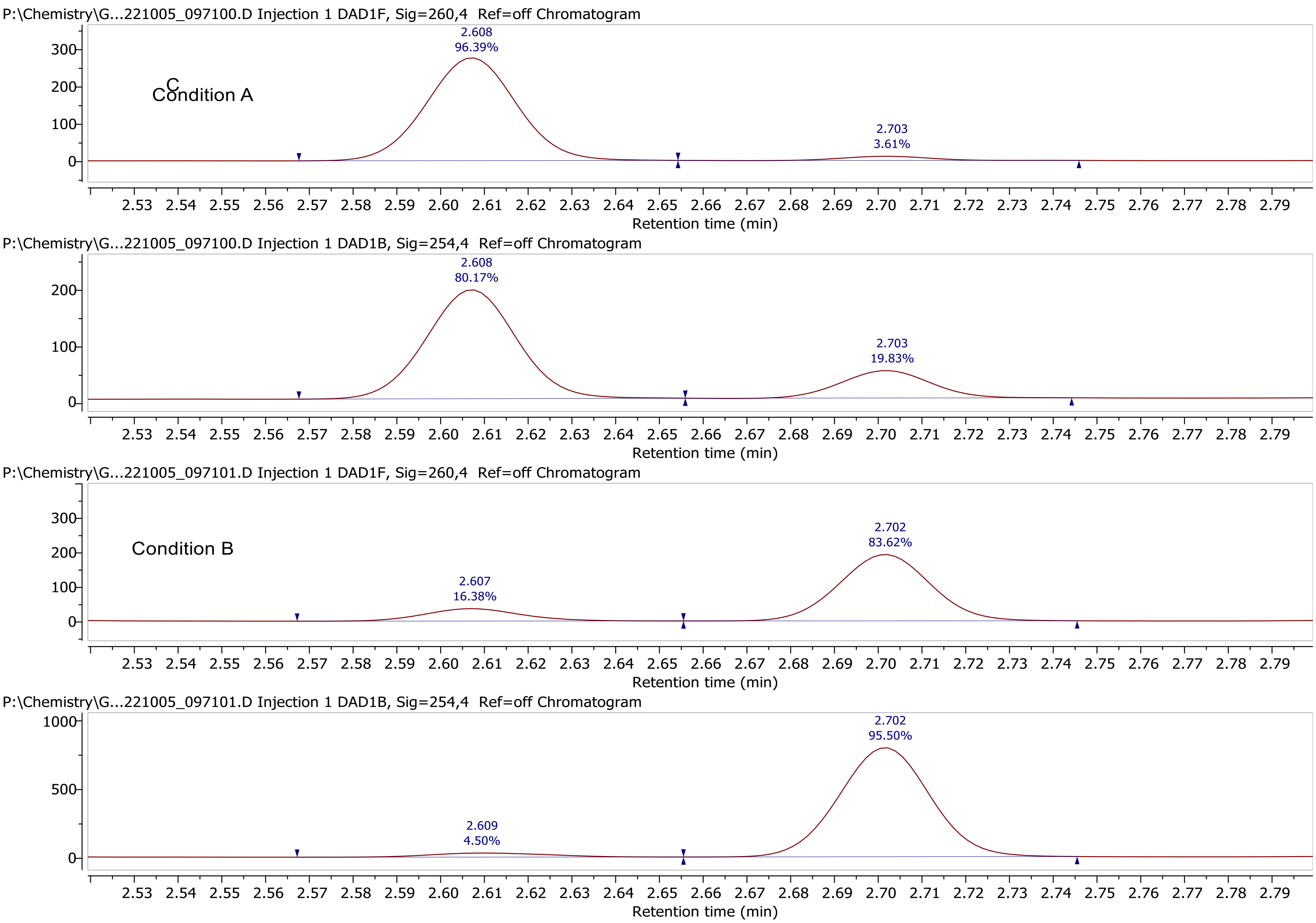
P:\Chemistry\G...221005_097100.D Injection 1 DAD1F, Sig=260,4 Ref=off Chromatogram
Condition A
2.608
96.39%
2.703
3.61%
Retention time (min)
P:\Chemistry\G...221005_097100.D Injection 1 DAD1B, Sig=254,4 Ref=off Chromatogram
2.608
80.17%
2.703
19.83%
Retention time (min)
P:\Chemistry\G...221005_097101.D Injection 1 DAD1F, Sig=260,4 Ref=off Chromatogram
Condition B
2.607
16.38%
2.702
83.62%
Retention time (min)
P:\Chemistry\G...221005_097101.D Injection 1 DAD1B, Sig=254,4 Ref=off Chromatogram
2.609
4.50%
2.702
95.50%
Retention time (min)

c

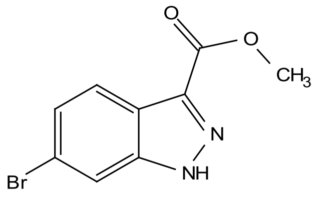

Condition A

Condition B

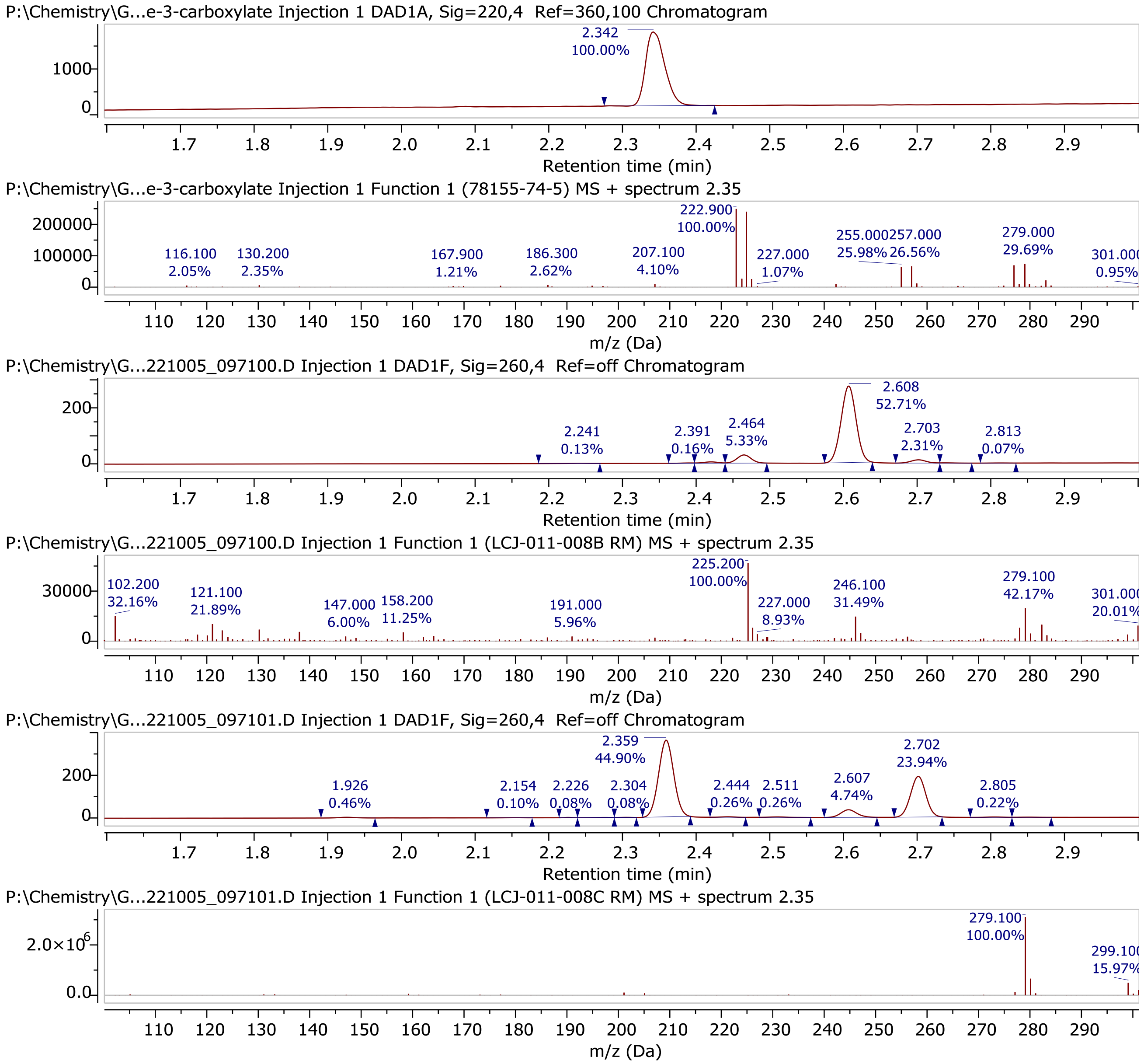

c: R = iPr, 6 versus reaction mixtures. Mass spectra for reaction mixtures at 2.344 minutes for each condition.

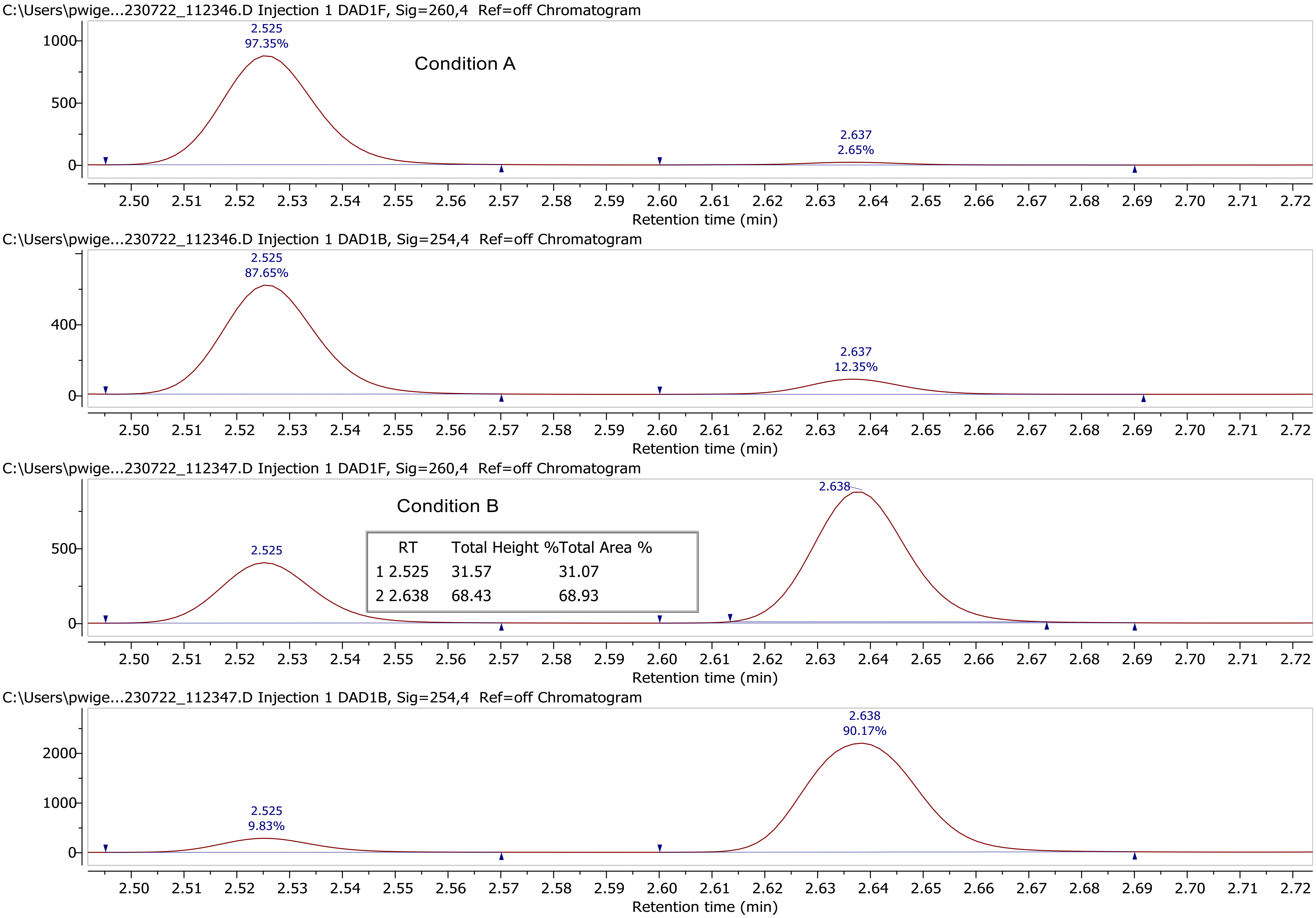
C:\Users\pwige...230722_112346.D Injection 1 DAD1F, Sig=260,4 Ref=off Chromatogram
Condition A
2.525
97.35%
2.637
2.65%
C:\Users\pwige...230722_112346.D Injection 1 DAD1B, Sig=254,4 Ref=off Chromatogram
2.525
87.65%
2.637
12.35%
C:\Users\pwige...230722_112347.D Injection 1 DAD1F, Sig=260,4 Ref=off Chromatogram
Condition B
2.525
2.638
RT Total Height %Total Area %
1 2.525 31.57 31.07
2 2.638 68.43 68.93
C:\Users\pwige...230722_112347.D Injection 1 DAD1B, Sig=254,4 Ref=off Chromatogram
2.525
9.83%
2.638
90.17%
Retention time (min)

d

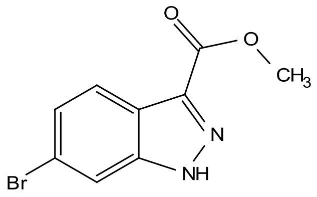

Condition A

Condition B

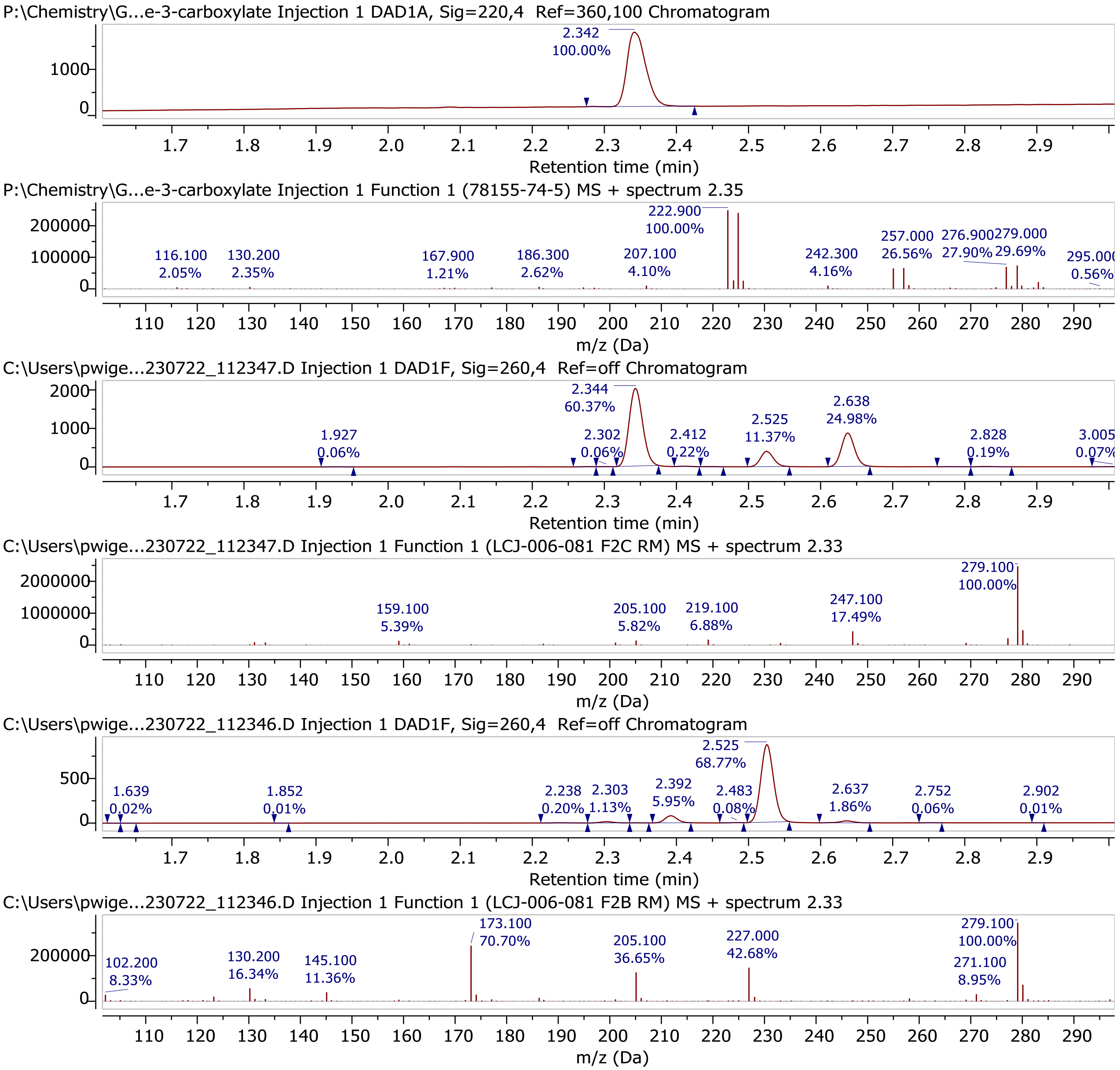

d: R = Pr, 6 versus reaction mixtures. Mass spectra for reaction mixtures at 2.344 minutes for each condition.

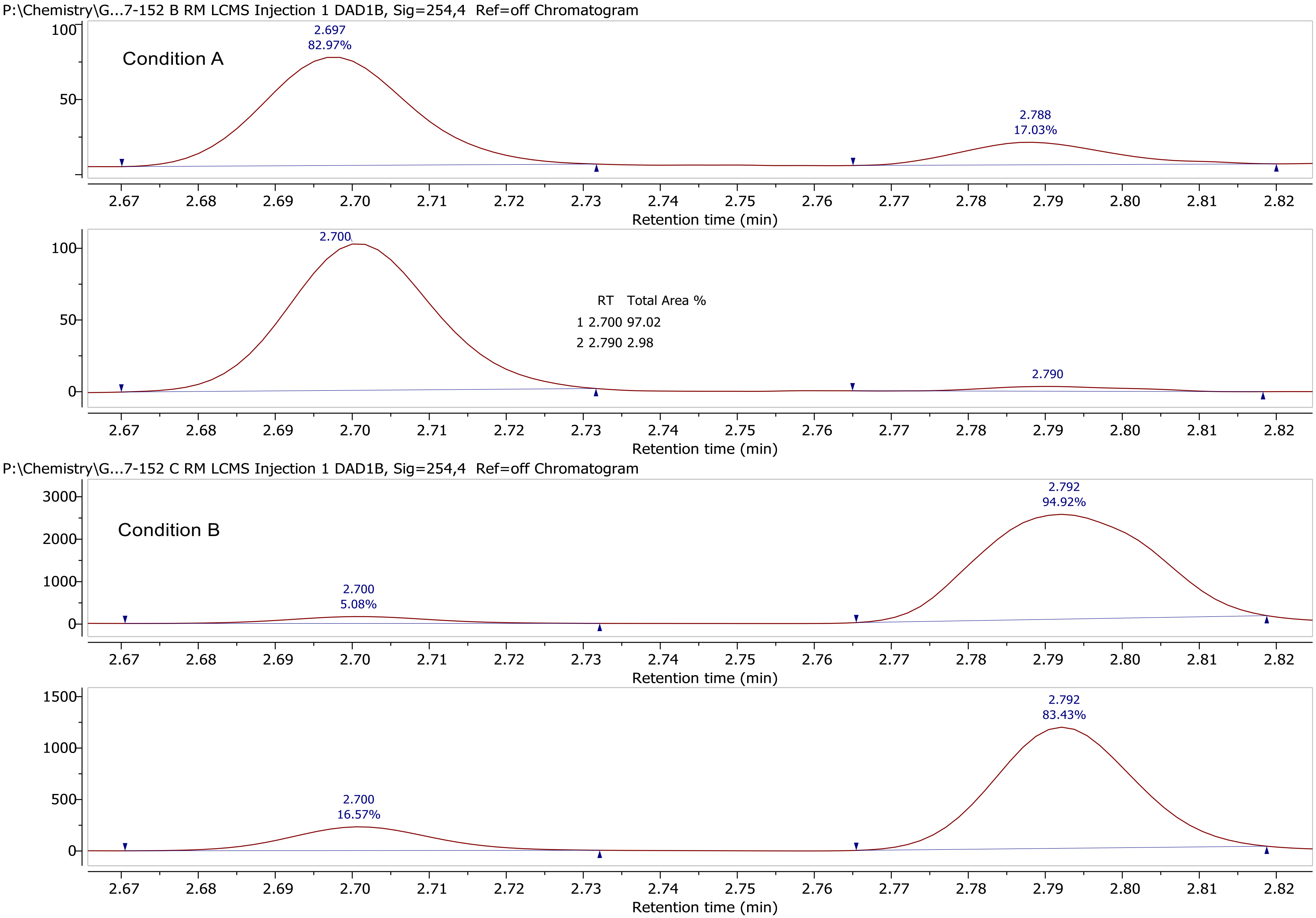
P:\Chemistry\G...7-152 B RM LCMS Injection 1 DAD1B, Sig=254,4 Ref=off Chromatogram
Condition A
2.697
82.97%
2.788
17.03%
Retention time (min)
2.700
RT Total Area %
1 2.700 97.02
2 2.790 2.98
2.790
Retention time (min)
P:\Chemistry\G...7-152 C RM LCMS Injection 1 DAD1B, Sig=254,4 Ref=off Chromatogram
Condition B
2.792
94.92%
2.700
5.08%
Retention time (min)
2.792
83.43%
2.700
16.57%
Retention time (min)

e

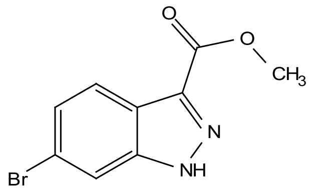

Condition A

Condition B

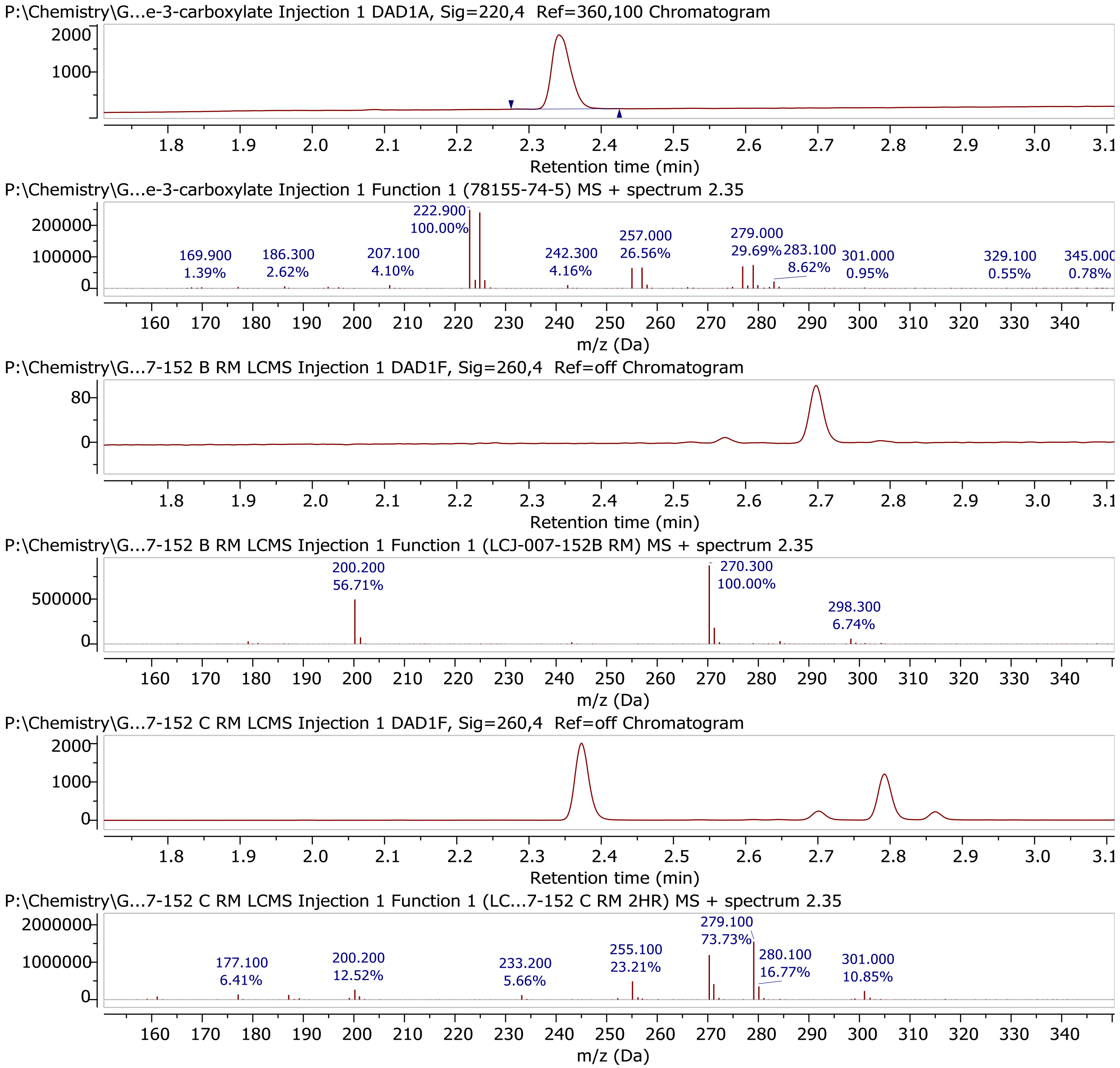

e: R = (R)- *sec*-butyl, 6 versus reaction mixtures. Mass spectra for reaction mixtures at 2.35 minutes for each condition

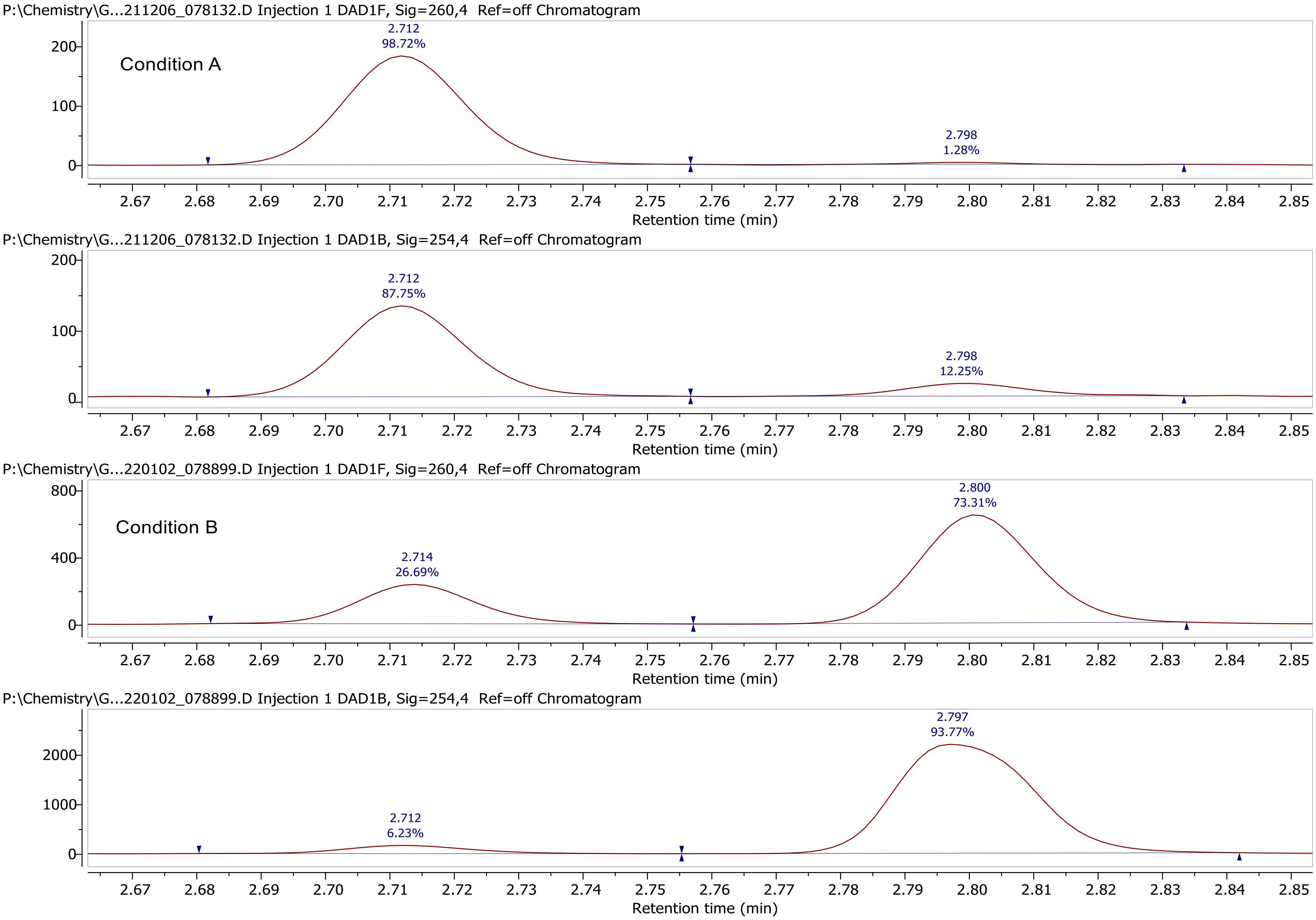
P:\Chemistry\G...211206_078132.D Injection 1 DAD1F, Sig=260,4 Ref=off Chromatogram
Condition A
2.712
98.72%
2.798
1.28%
Retention time (min)
P:\Chemistry\G...211206_078132.D Injection 1 DAD1B, Sig=254,4 Ref=off Chromatogram
2.712
87.75%
2.798
12.25%
Retention time (min)
P:\Chemistry\G...220102_078899.D Injection 1 DAD1F, Sig=260,4 Ref=off Chromatogram
Condition B
2.714
26.69%
2.800
73.31%
Retention time (min)
P:\Chemistry\G...220102_078899.D Injection 1 DAD1B, Sig=254,4 Ref=off Chromatogram
2.712
6.23%
2.797
93.77%
Retention time (min)

f

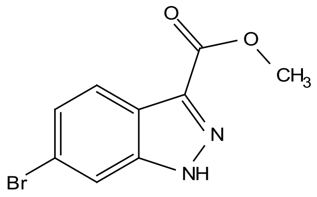

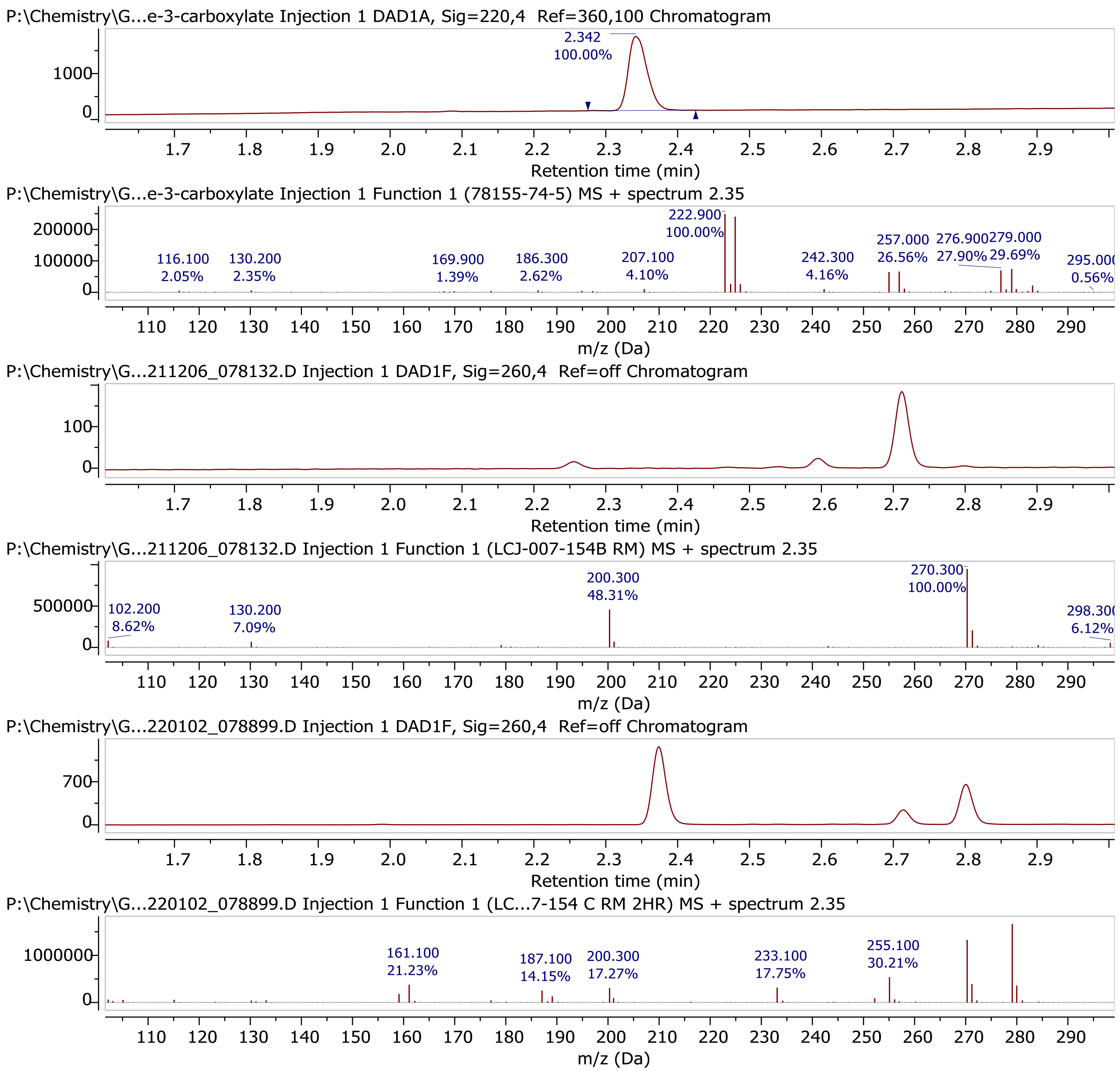

Condition A

Condition B

f: R = iBu, 6 versus reaction mixtures. Mass spectra for reaction mixtures at 2.35 minutes for each condition.

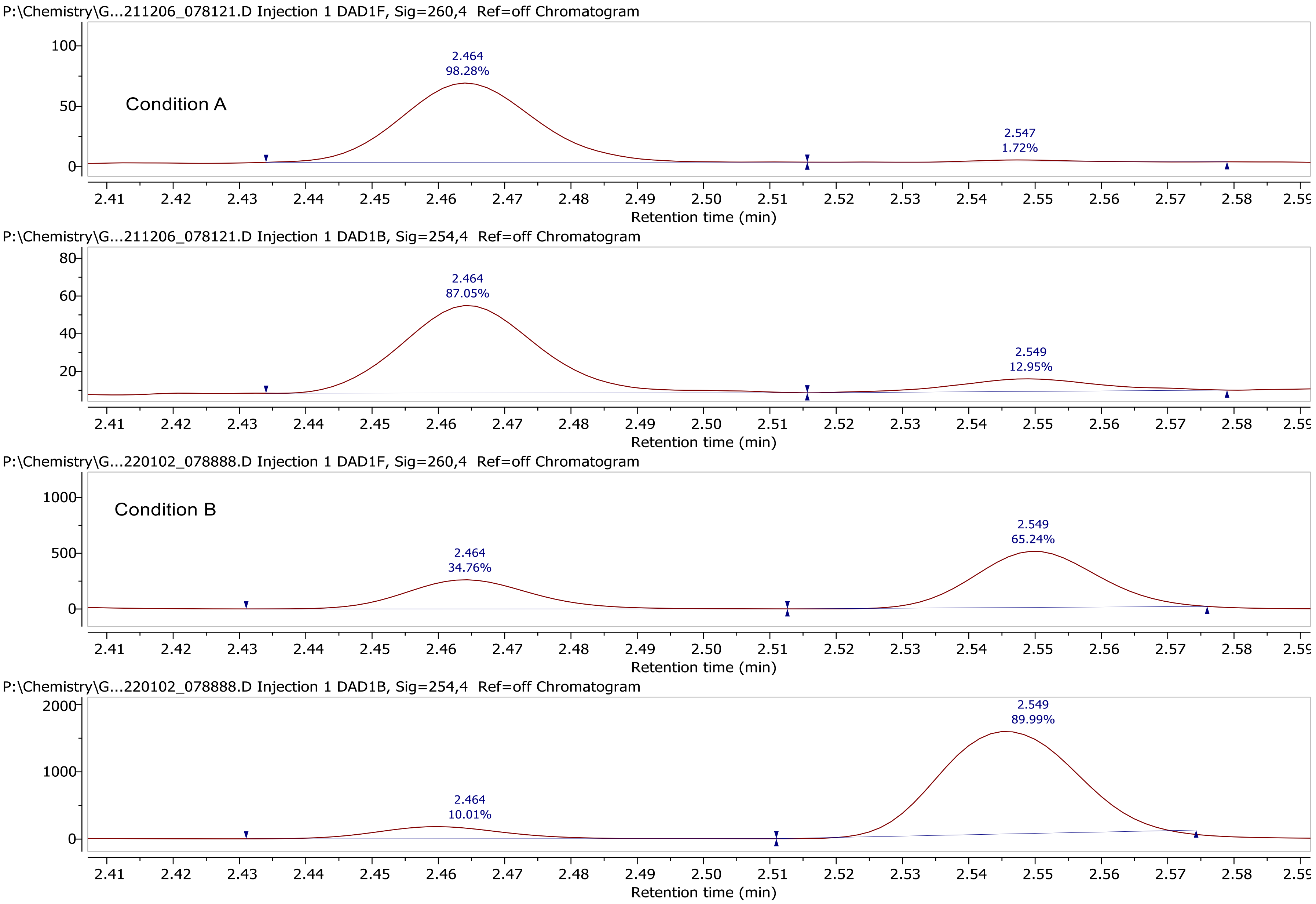

P:\Chemistry\G...211206_078121.D Injection 1 DAD1F, Sig=260,4 Ref=off Chromatogram
Condition A
2.464
98.28%
2.547
1.72%
Retention time (min)
P:\Chemistry\G...211206_078121.D Injection 1 DAD1B, Sig=254,4 Ref=off Chromatogram
2.464
87.05%
2.549
12.95%
Retention time (min)
P:\Chemistry\G...220102_078888.D Injection 1 DAD1F, Sig=260,4 Ref=off Chromatogram
Condition B
2.464
34.76%
2.549
65.24%
Retention time (min)
P:\Chemistry\G...220102_078888.D Injection 1 DAD1B, Sig=254,4 Ref=off Chromatogram
2.464
10.01%
2.549
89.99%
Retention time (min)

g

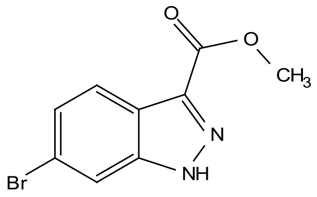

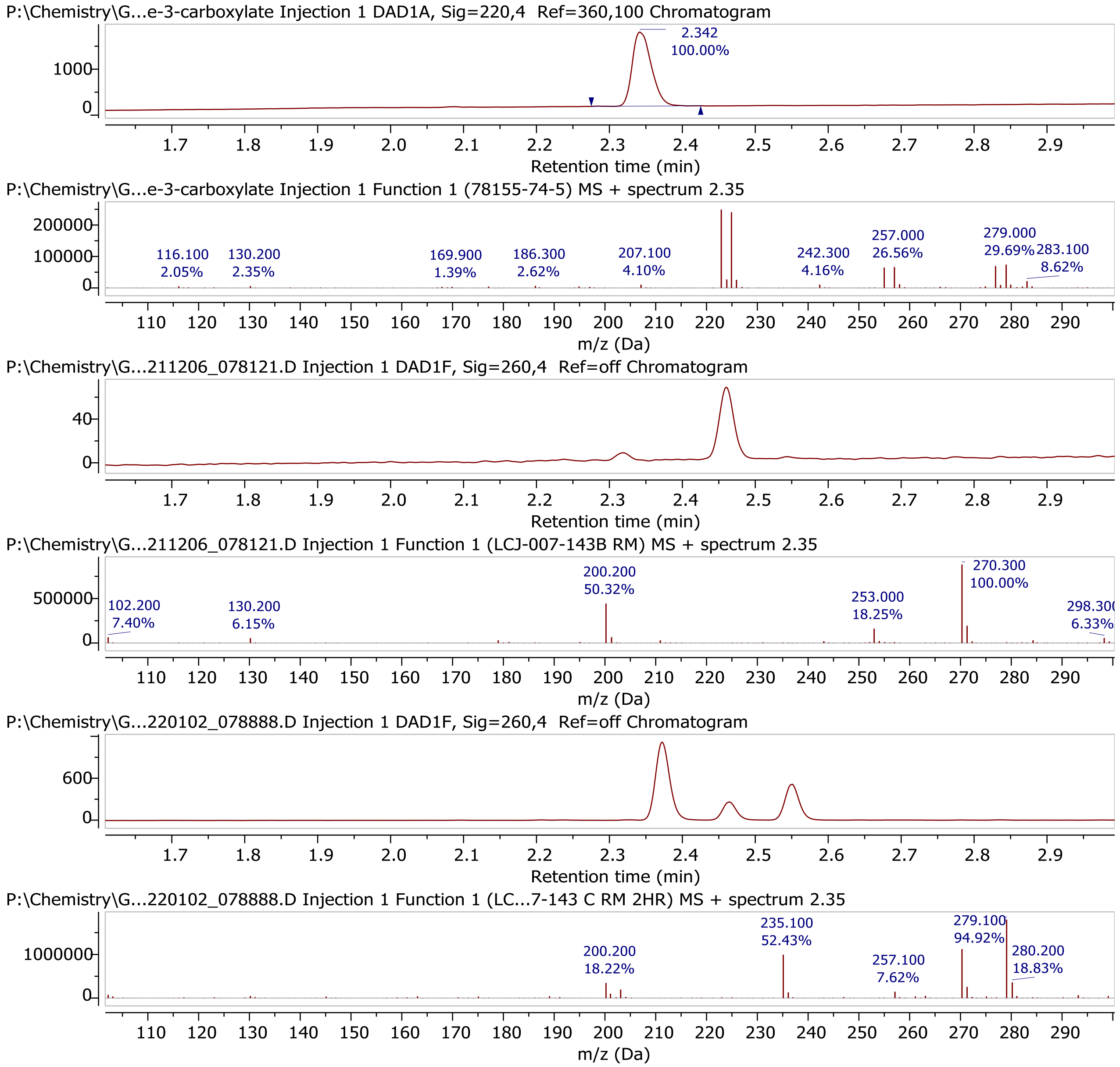

Condition A

Condition B

g: R = 2-OMeEt, 6 versus reaction mixtures. Mass spectra for reaction mixtures at 2.35 minutes for each condition.

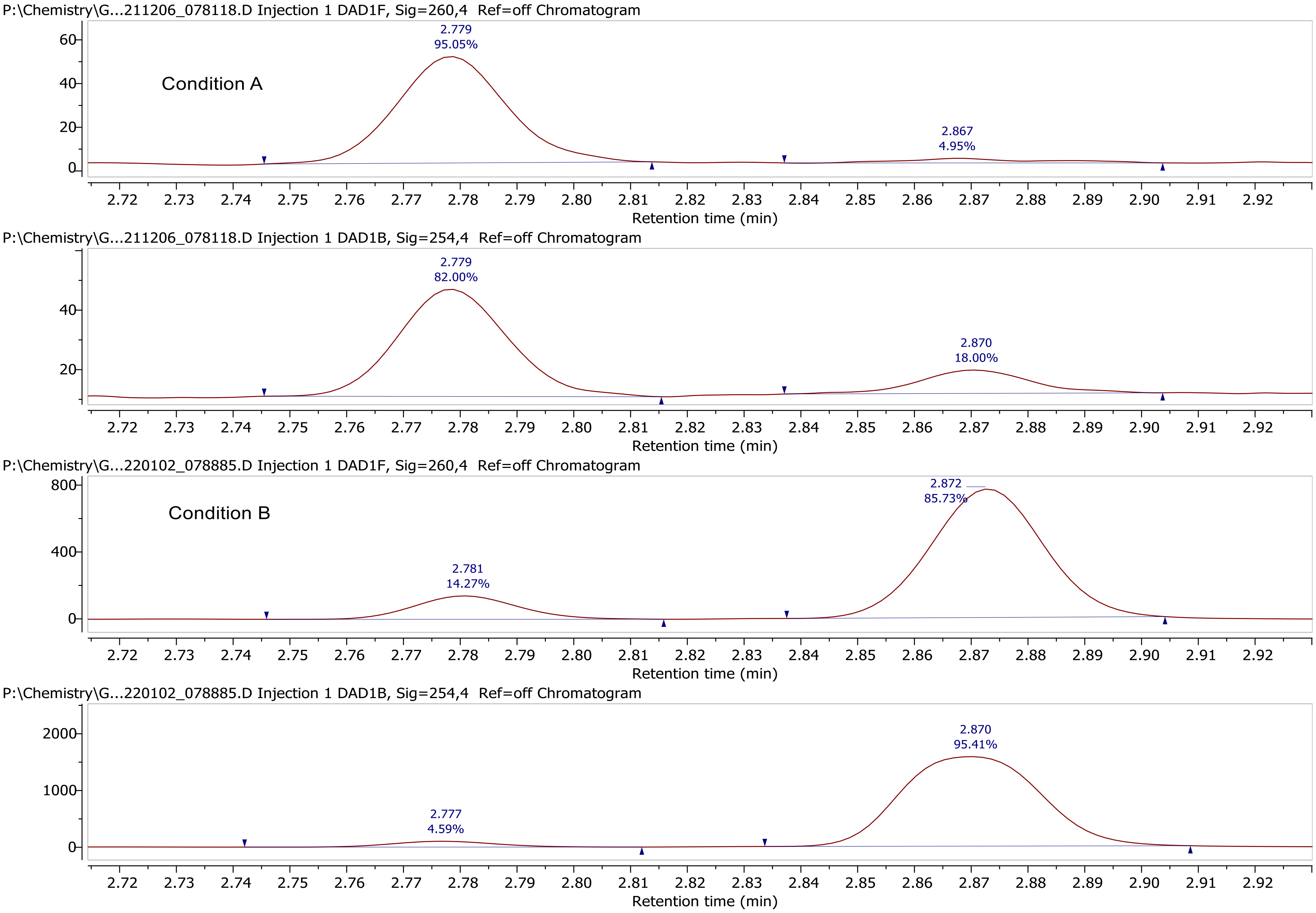
P:\Chemistry\G...211206_078118.D Injection 1 DAD1F, Sig=260,4 Ref=off Chromatogram
Condition A
2.779
95.05%
2.867
4.95%
Retention time (min)
P:\Chemistry\G...211206_078118.D Injection 1 DAD1B, Sig=254,4 Ref=off Chromatogram
2.779
82.00%
2.870
18.00%
Retention time (min)
P:\Chemistry\G...220102_078885.D Injection 1 DAD1F, Sig=260,4 Ref=off Chromatogram
Condition B
2.781
14.27%
2.872
85.73%
Retention time (min)
P:\Chemistry\G...220102_078885.D Injection 1 DAD1B, Sig=254,4 Ref=off Chromatogram
2.777
4.59%
2.870
95.41%
Retention time (min)

h

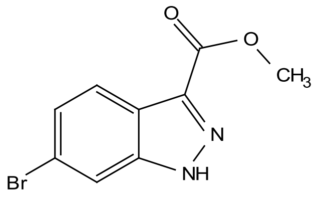

Condition A

Condition B

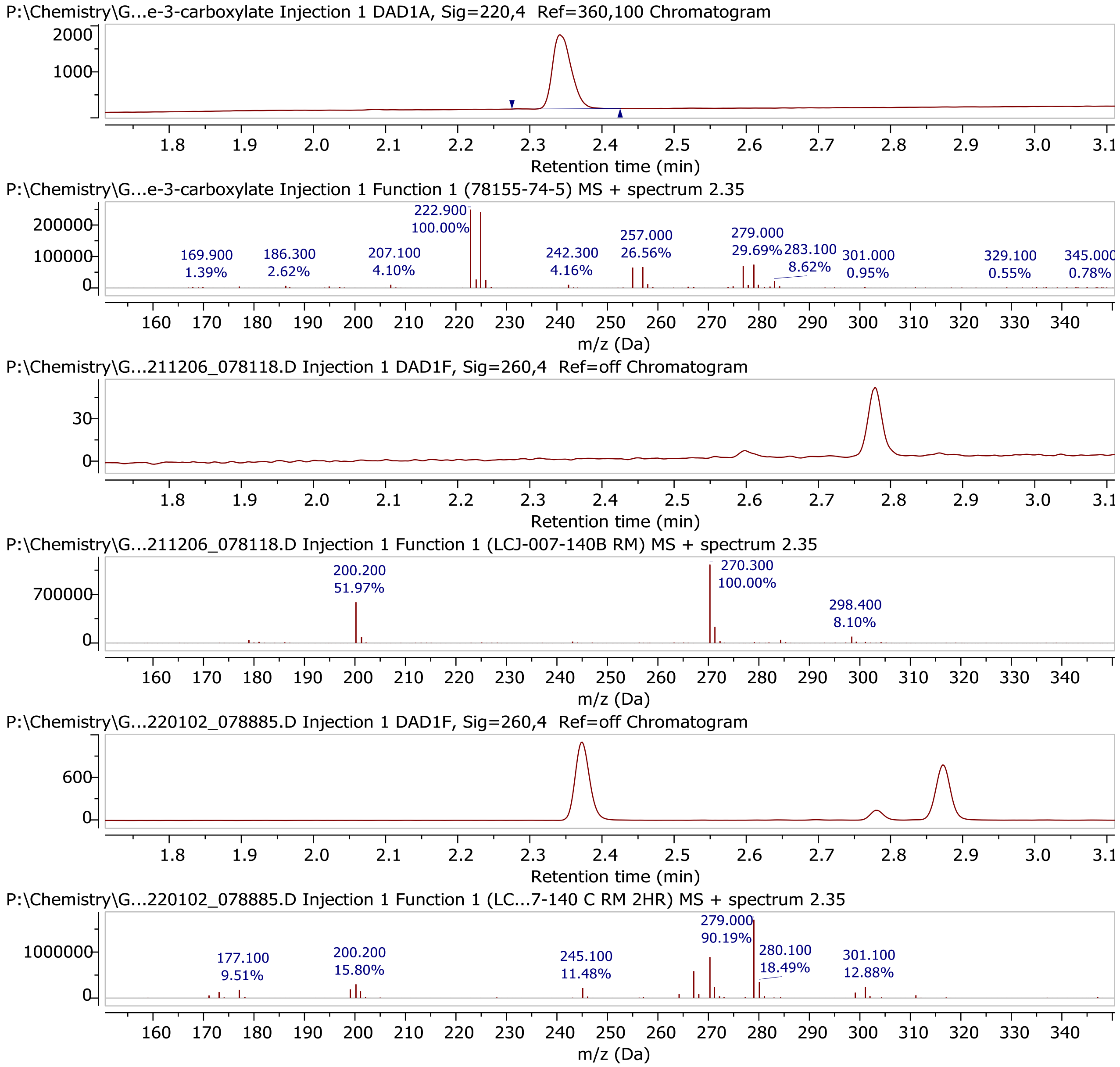

h: R = cC5H9, 6 versus reaction mixtures. Mass spectra for reaction mixtures at 2.35 minutes for each condition.

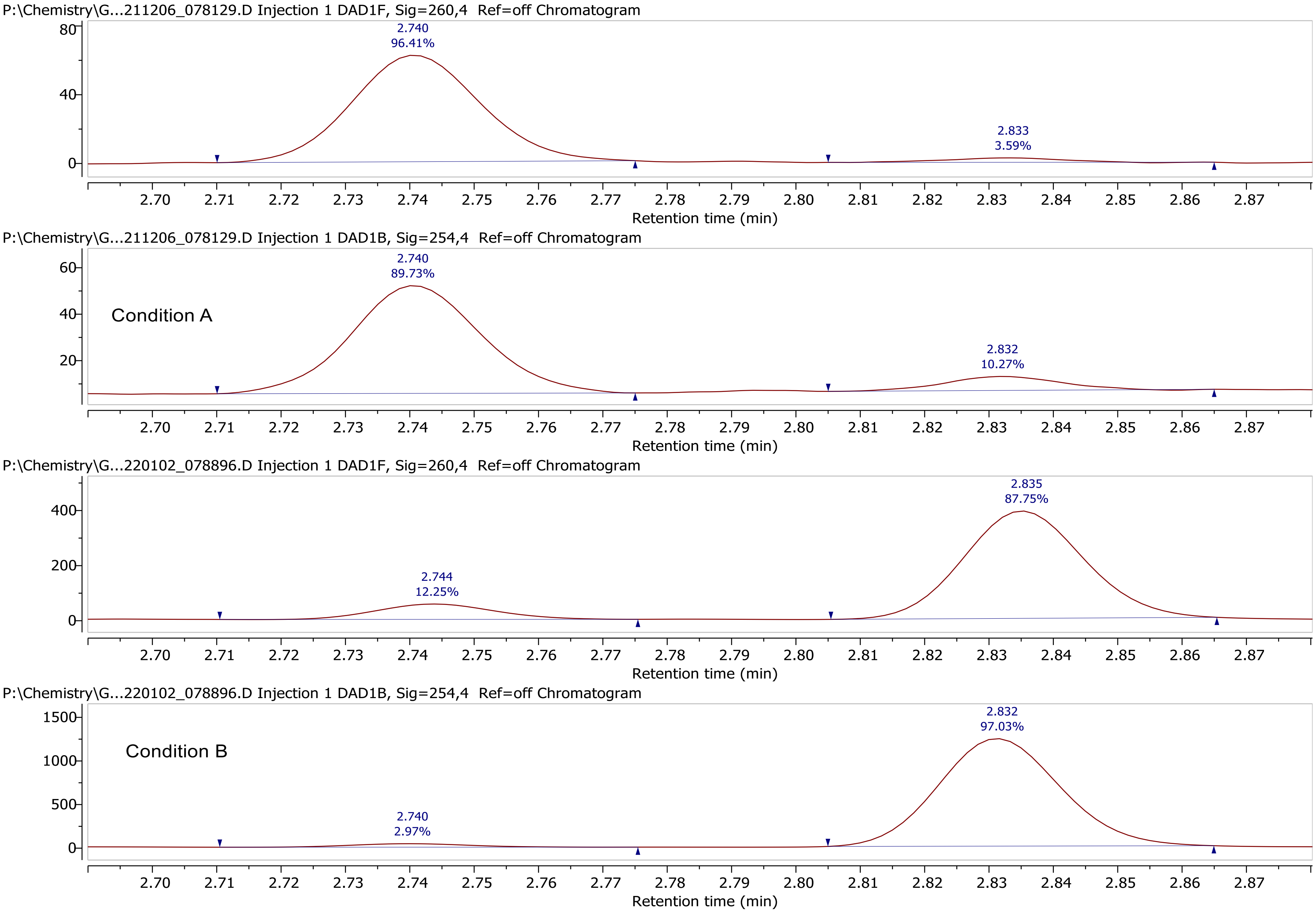
P:\Chemistry\G...211206_078129.D Injection 1 DAD1F, Sig=260,4 Ref=off Chromatogram
2.740
96.41%
2.833
3.59%
Retention time (min)
P:\Chemistry\G...211206_078129.D Injection 1 DAD1B, Sig=254,4 Ref=off Chromatogram
Condition A
2.740
89.73%
2.832
10.27%
Retention time (min)
P:\Chemistry\G...220102_078896.D Injection 1 DAD1F, Sig=260,4 Ref=off Chromatogram
2.744
12.25%
2.835
87.75%
Retention time (min)
P:\Chemistry\G...220102_078896.D Injection 1 DAD1B, Sig=254,4 Ref=off Chromatogram
Condition B
2.740
2.97%
2.832
97.03%
Retention time (min)

i

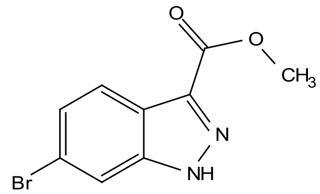

Condition A

Condition B

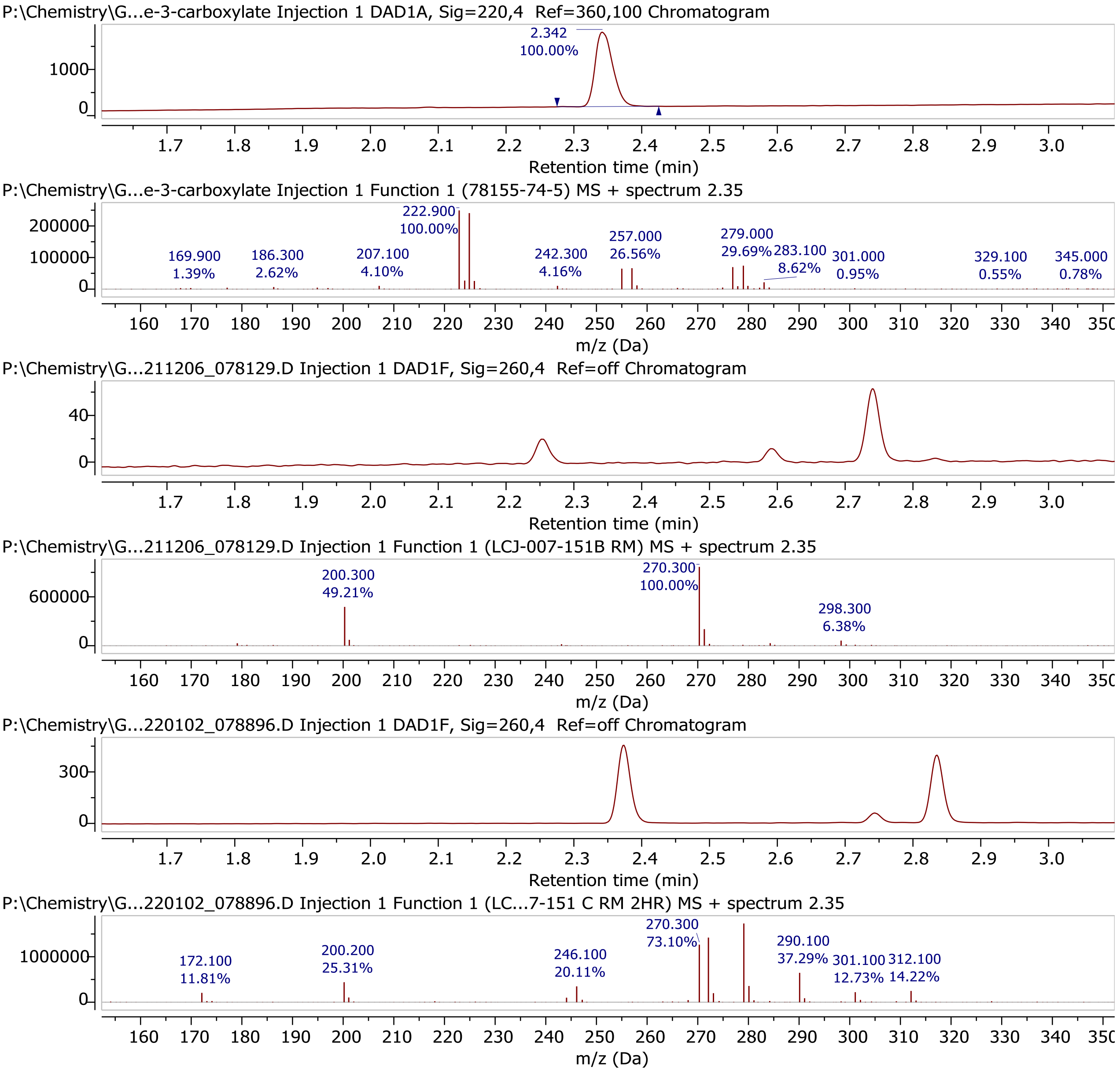

i: R = *N*-Boc-3-pyrrolidine, 6 versus reaction mixtures. Mass spectra for reaction mixtures at 2.35 minutes for each condition.

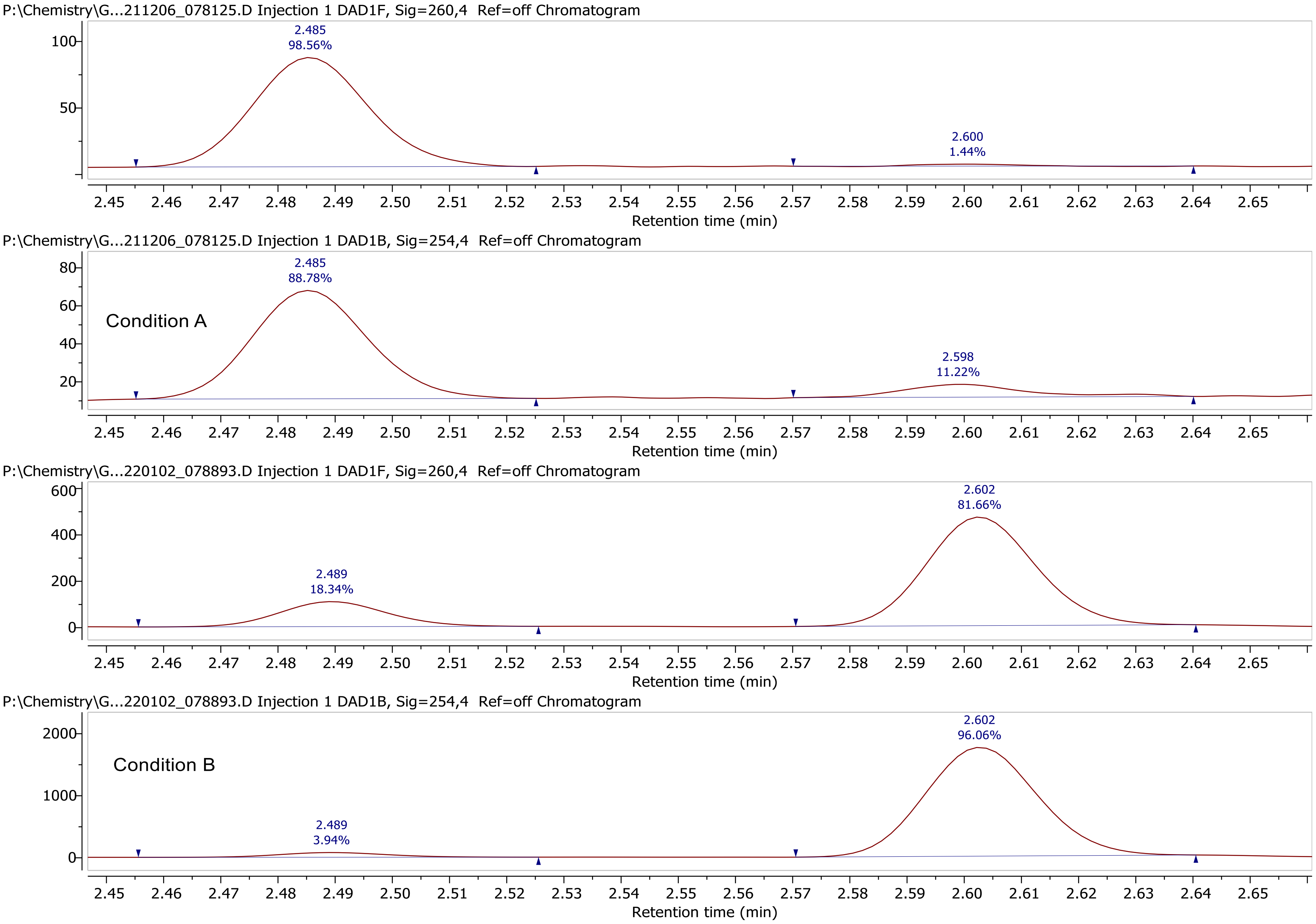
P:\Chemistry\G...211206_078125.D Injection 1 DAD1F, Sig=260,4 Ref=off Chromatogram
2.485
98.56%
2.600
1.44%
Retention time (min)
P:\Chemistry\G...211206_078125.D Injection 1 DAD1B, Sig=254,4 Ref=off Chromatogram
Condition A
2.485
88.78%
2.598
11.22%
Retention time (min)
P:\Chemistry\G...220102_078893.D Injection 1 DAD1F, Sig=260,4 Ref=off Chromatogram
2.489
18.34%
2.602
81.66%
Retention time (min)
P:\Chemistry\G...220102_078893.D Injection 1 DAD1B, Sig=254,4 Ref=off Chromatogram
Condition B
2.489
3.94%
2.602
96.06%
Retention time (min)

j

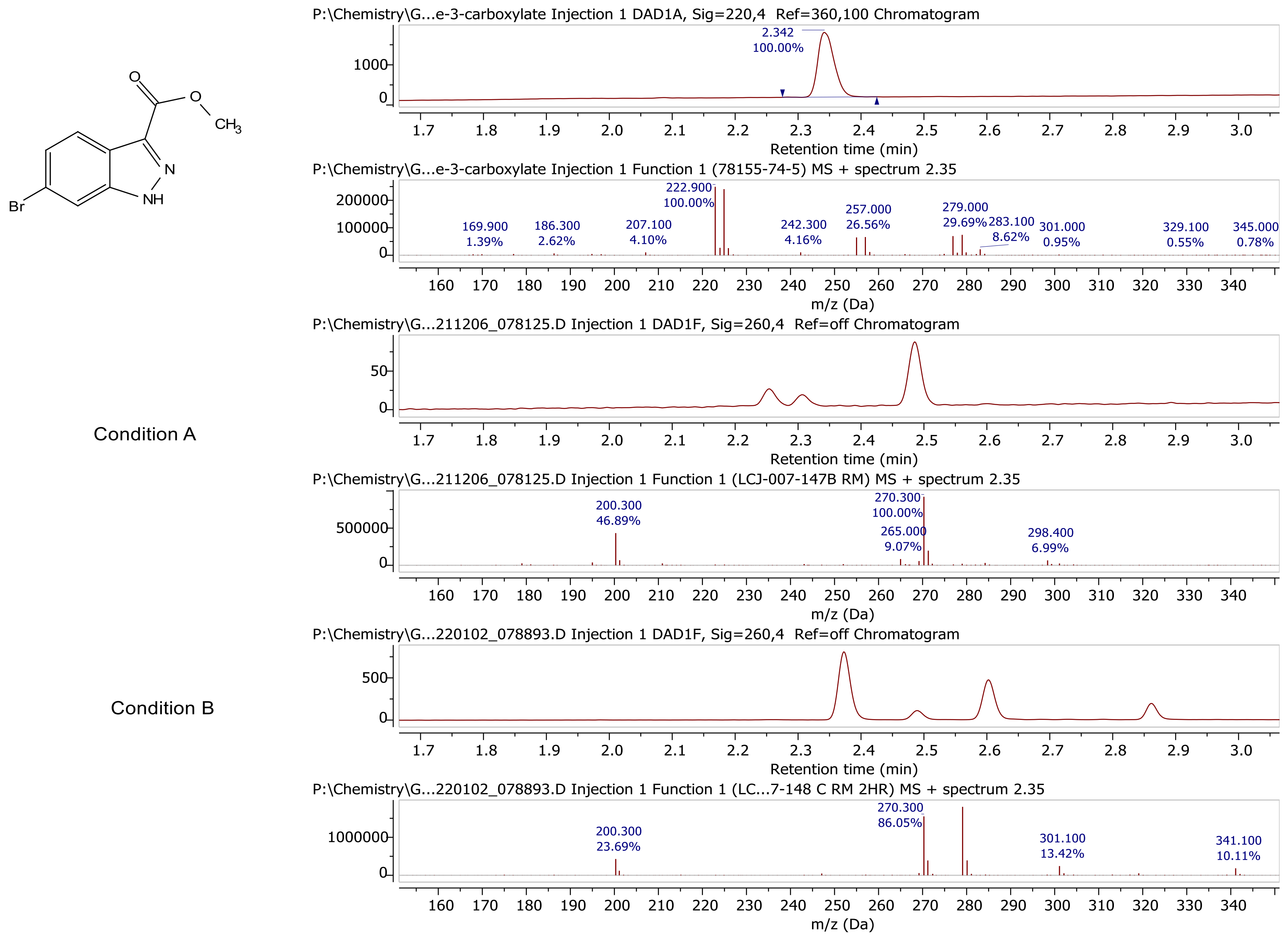

j: R = (*S*)-3-THF, 6 versus reaction mixtures. Mass spectra for reaction mixtures at 2.35 minutes for each condition.

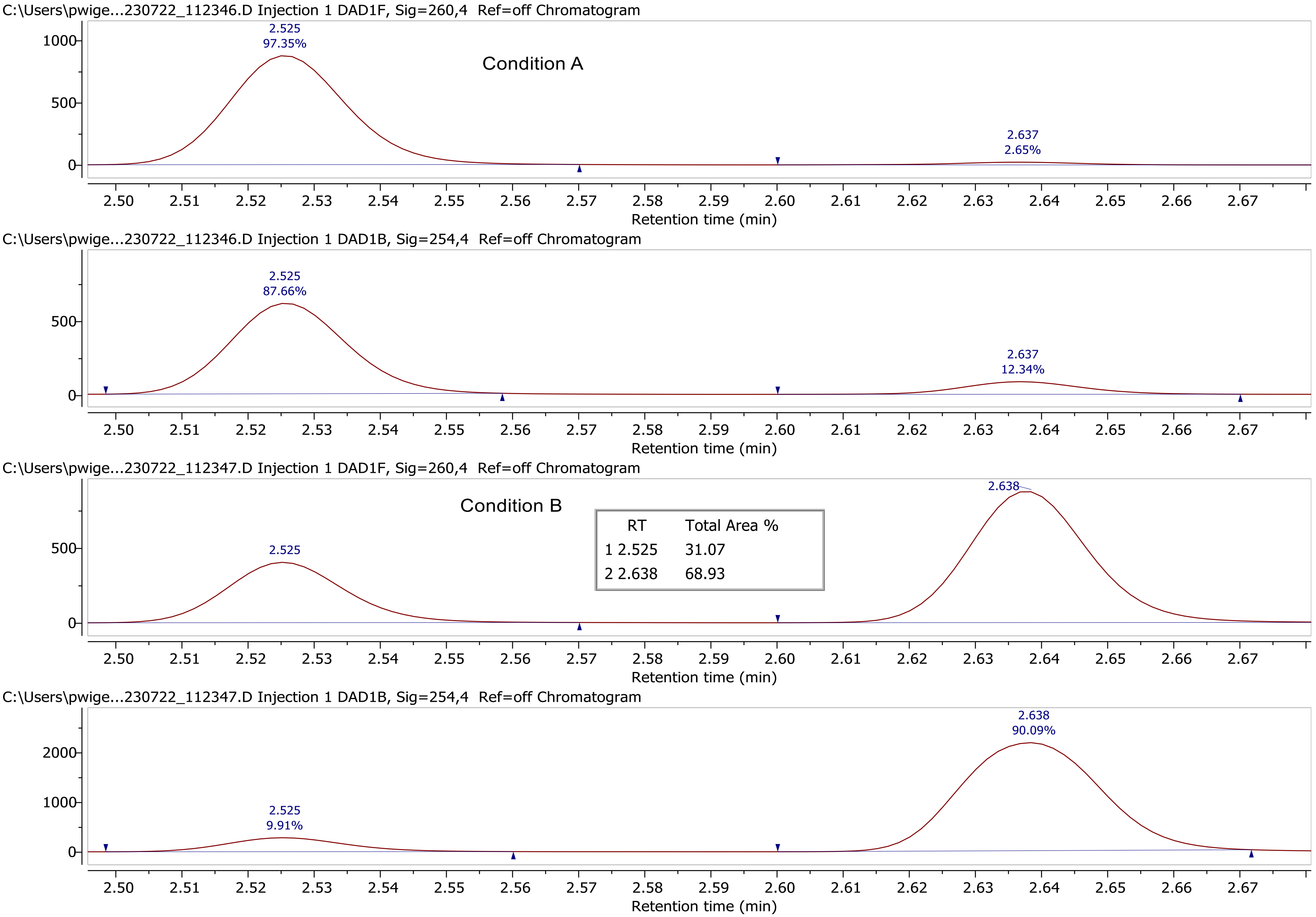
C:\Users\pwige...230722_112346.D Injection 1 DAD1F, Sig=260,4 Ref=off Chromatogram
Condition A
2.525
97.35%
2.637
2.65%
Retention time (min)
C:\Users\pwige...230722_112346.D Injection 1 DAD1B, Sig=254,4 Ref=off Chromatogram
2.525
87.66%
2.637
12.34%
Retention time (min)
C:\Users\pwige...230722_112347.D Injection 1 DAD1F, Sig=260,4 Ref=off Chromatogram
Condition B
RT Total Area %
1 2.525 31.07
2 2.638 68.93
2.525
2.638
Retention time (min)
C:\Users\pwige...230722_112347.D Injection 1 DAD1B, Sig=254,4 Ref=off Chromatogram
2.525
9.91%
2.638
90.09%
Retention time (min)

k

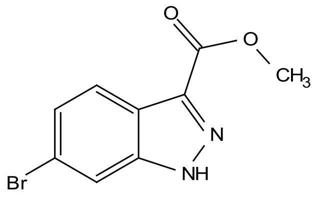

Condition A

Condition B

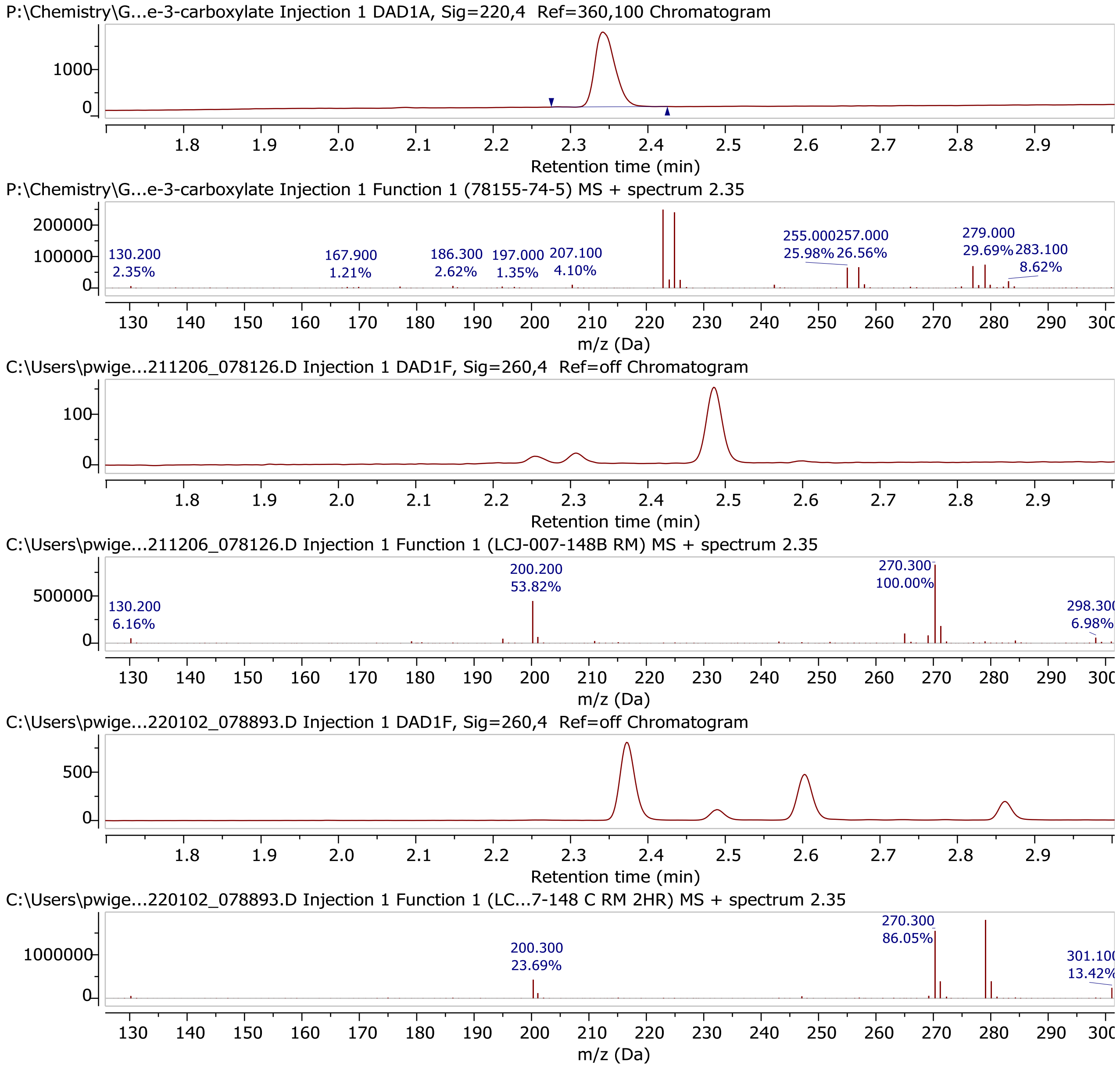

k: R = (*R*)-3-THF, 6 versus reaction mixtures. Mass spectra for reaction mixtures at 2.35 minutes for each condition.

P:\Chemistry\G...7-142 B RM LCMS Injection 1 DAD1B, Sig=254,4 Ref=off Chromatogram

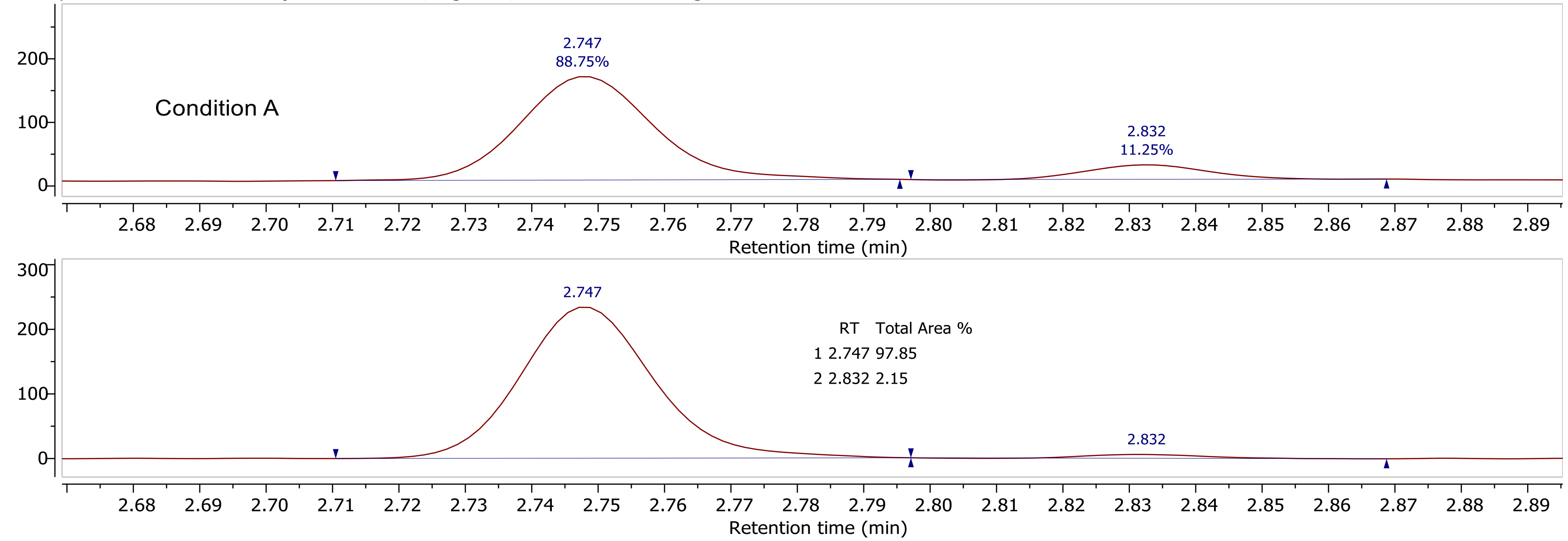

P:\Chemistry\G...7-142 C RM LCMS Injection 1 DAD1B, Sig=254,4 Ref=off Chromatogram

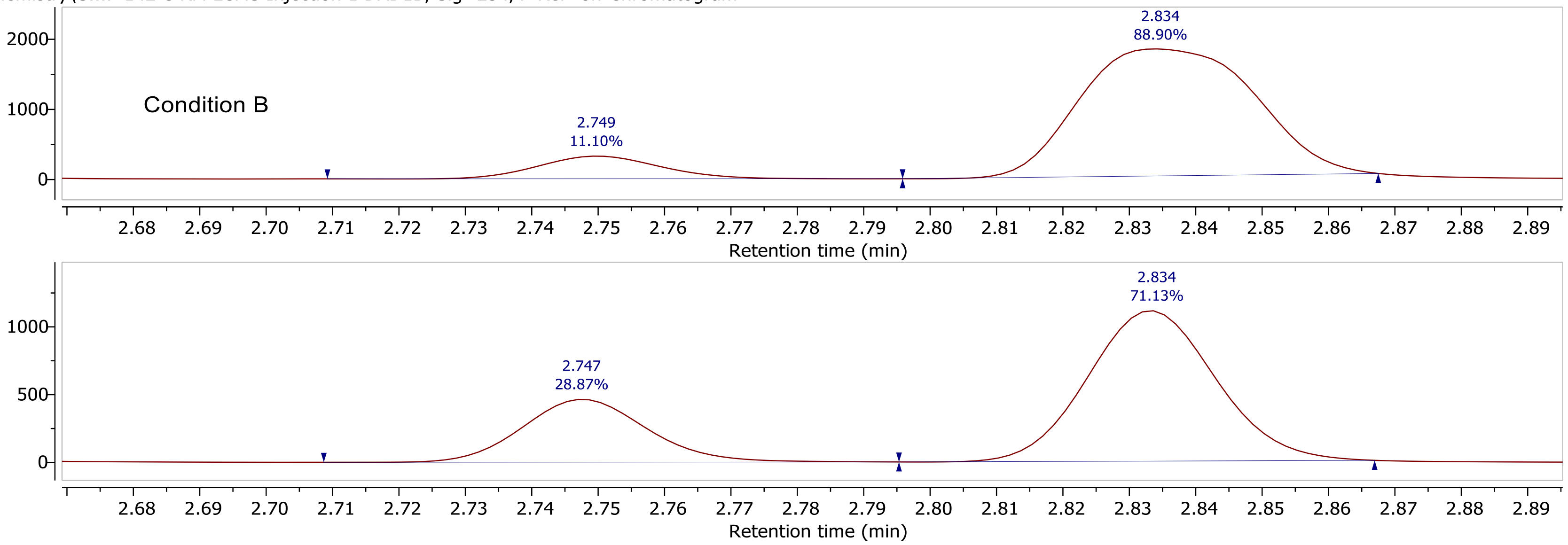

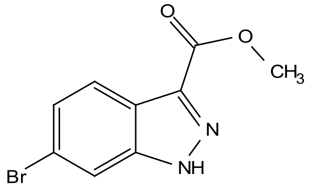

Condition A

Condition B

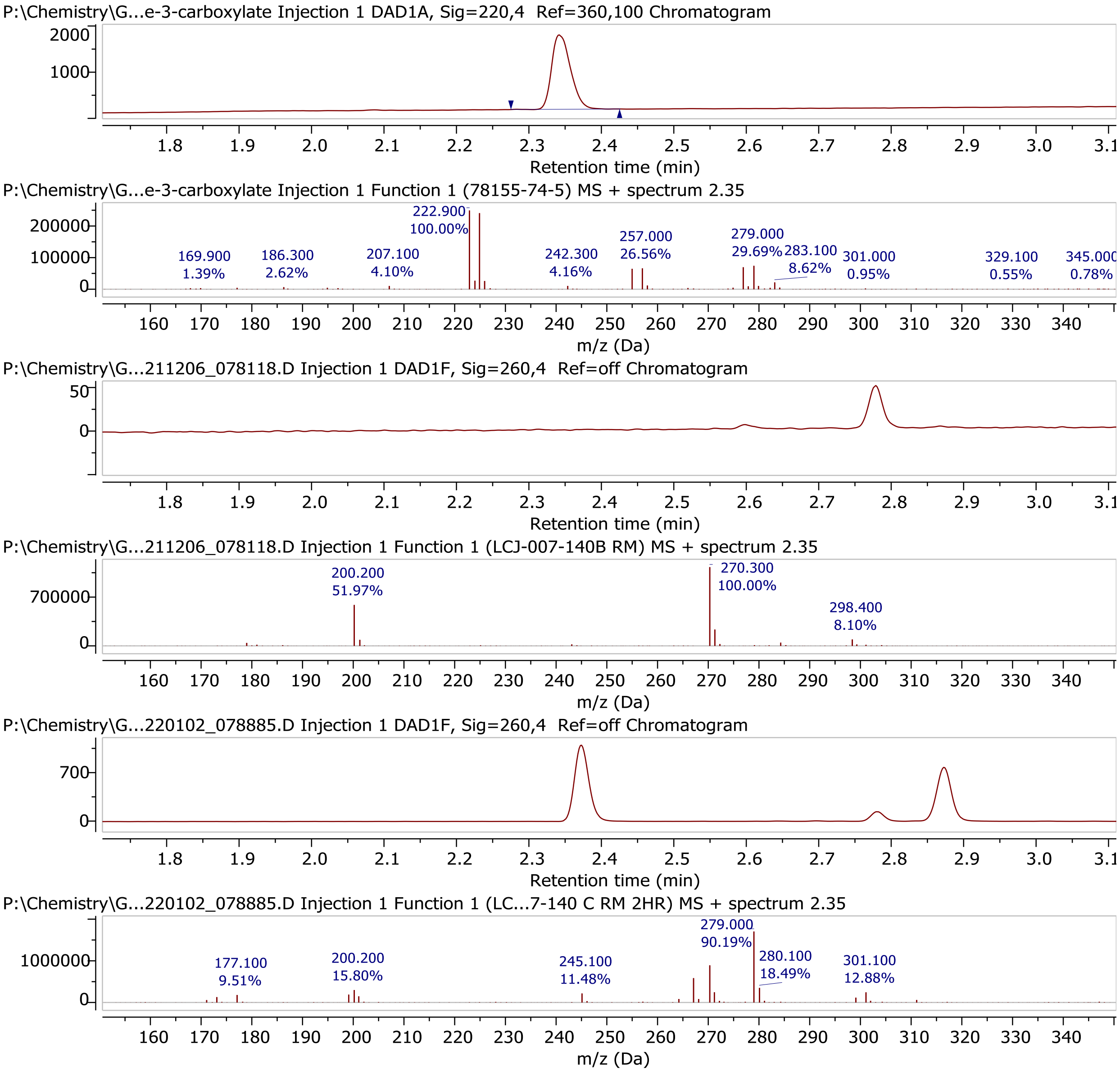

l: R = $CH_2\mathit{c}C_4H_7$, 6 versus reaction mixtures. Mass spectra for reaction mixtures at 2.35 minutes for each condition.

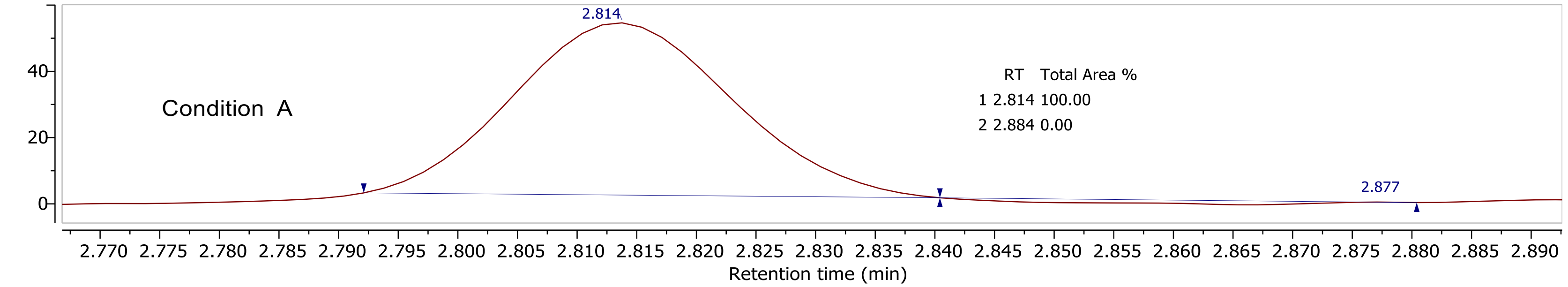

P:\Chemistry\G...7-141 B RM LCMS Injection 1 DAD1B, Sig=254,4 Ref=off Chromatogram

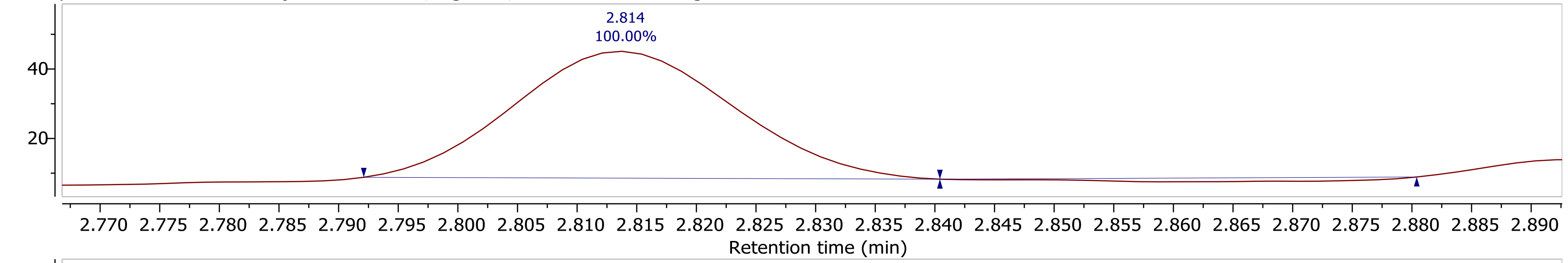

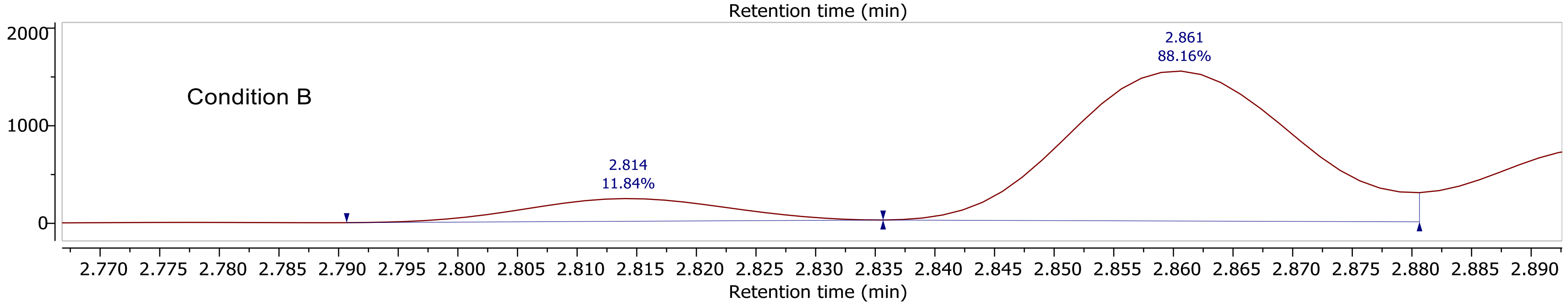

P:\Chemistry\G...7-141 C RM LCMS Injection 1 DAD1B, Sig=254,4 Ref=off Chromatogram

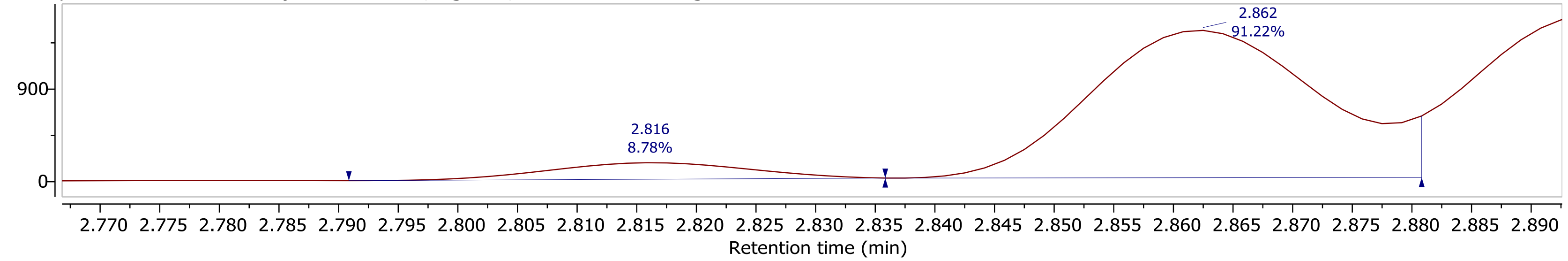

n

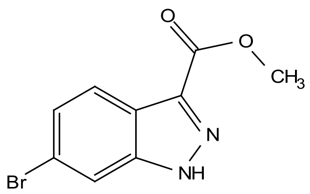

Condition A

Condition B

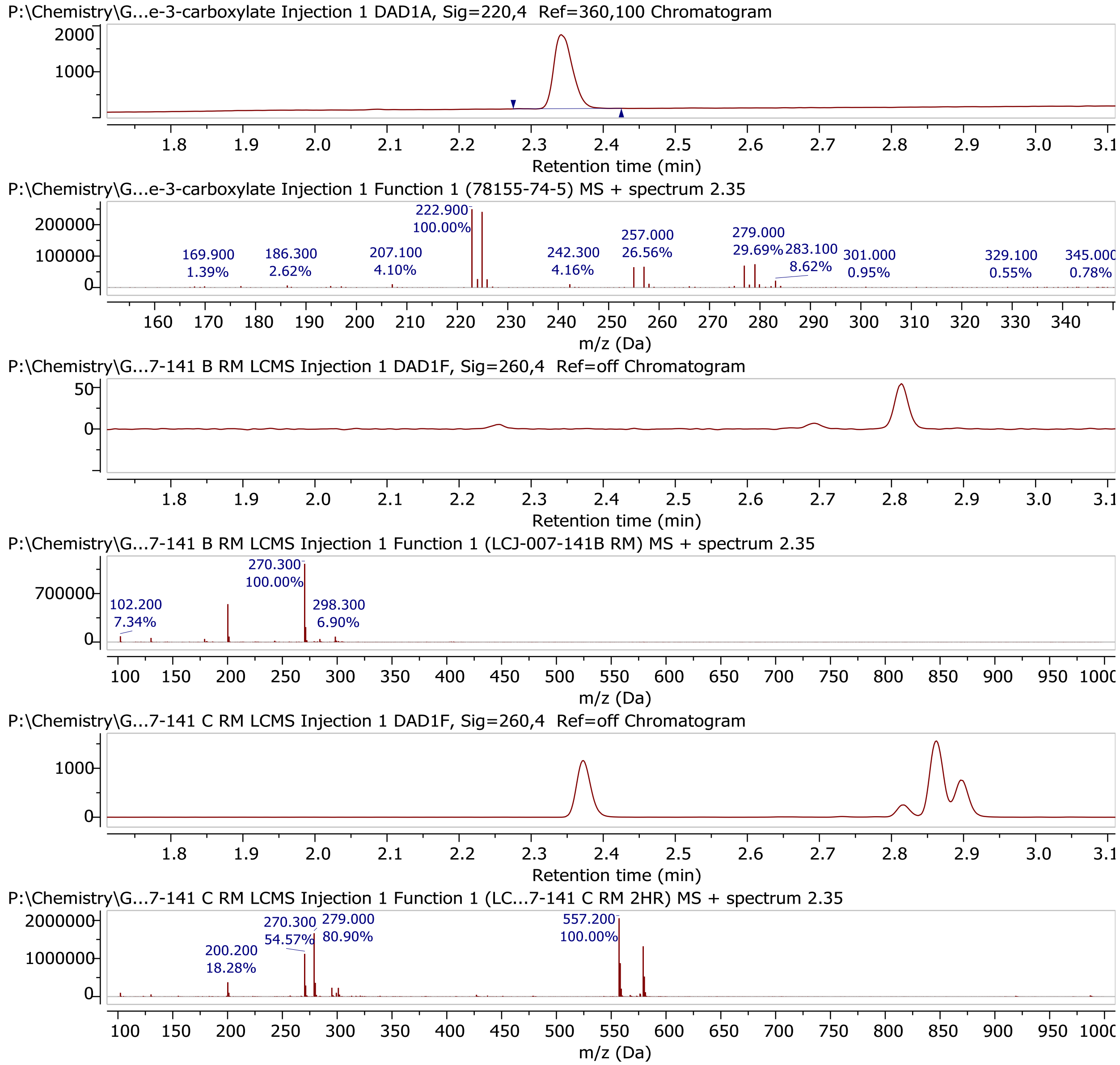

n: R = $CH_2cC_5H_9$, 6 versus reaction mixtures. Mass spectra for reaction mixtures at 2.35 minutes for each condition.

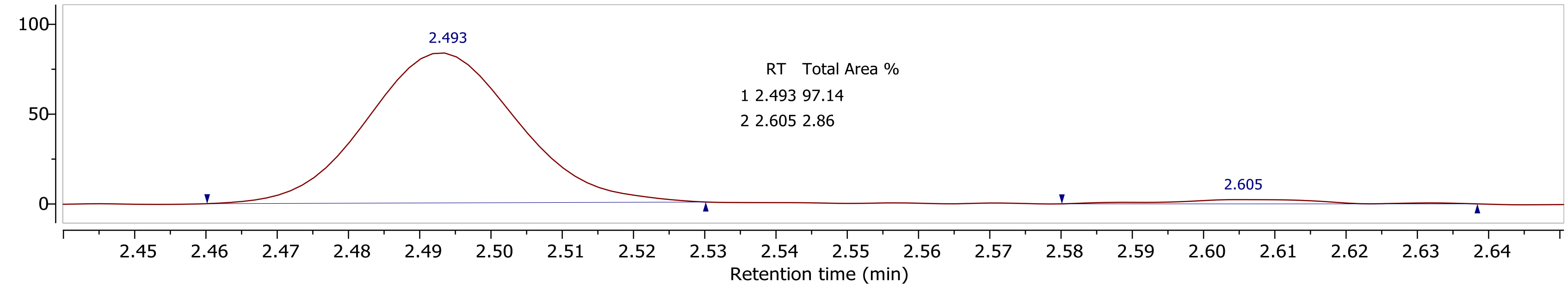

P:\Chemistry\G...7-146 B RM LCMS Injection 1 DAD1B, Sig=254,4 Ref=off Chromatogram

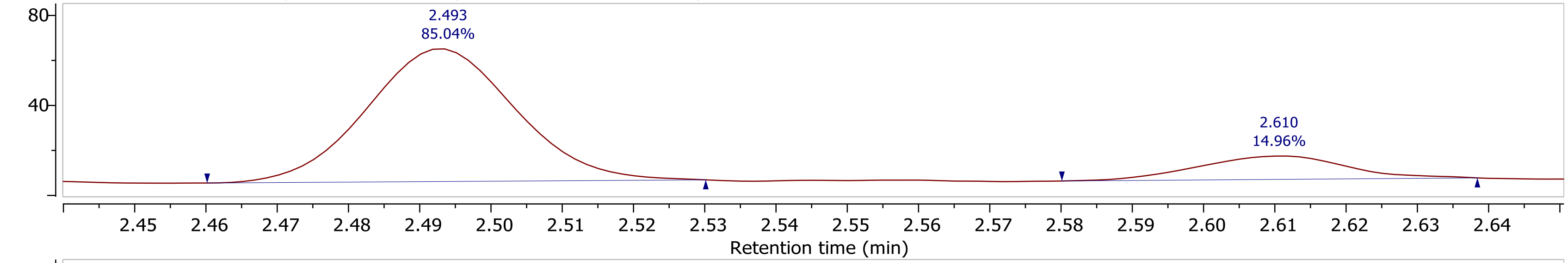

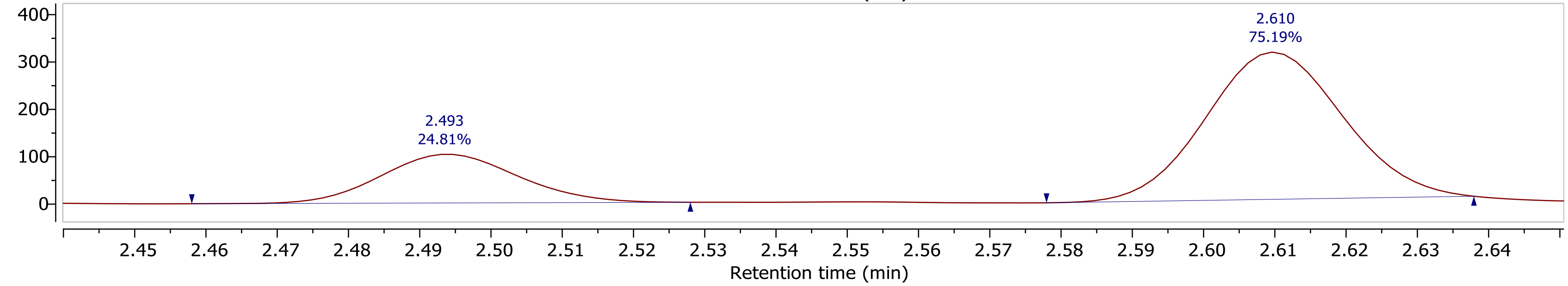

P:\Chemistry\G...7-146 C RM LCMS Injection 1 DAD1B, Sig=254,4 Ref=off Chromatogram

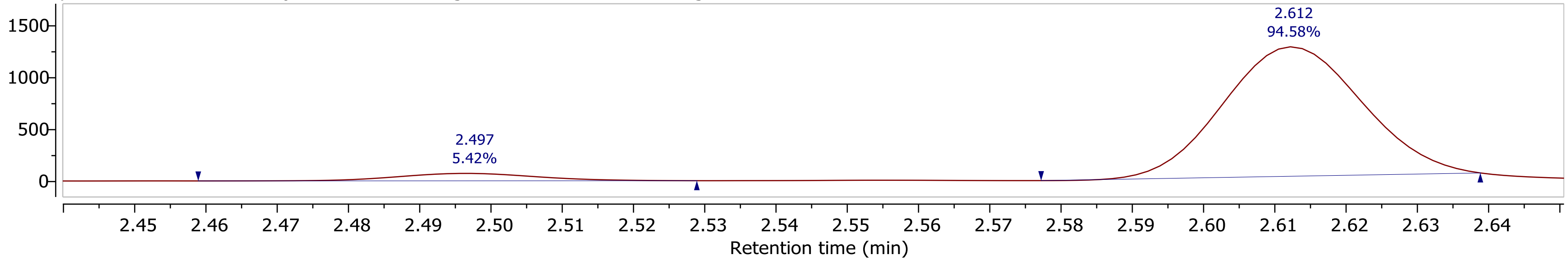

o

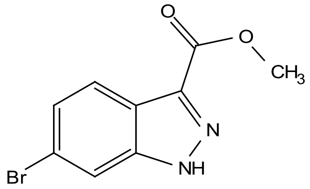

Condition A

Condition B

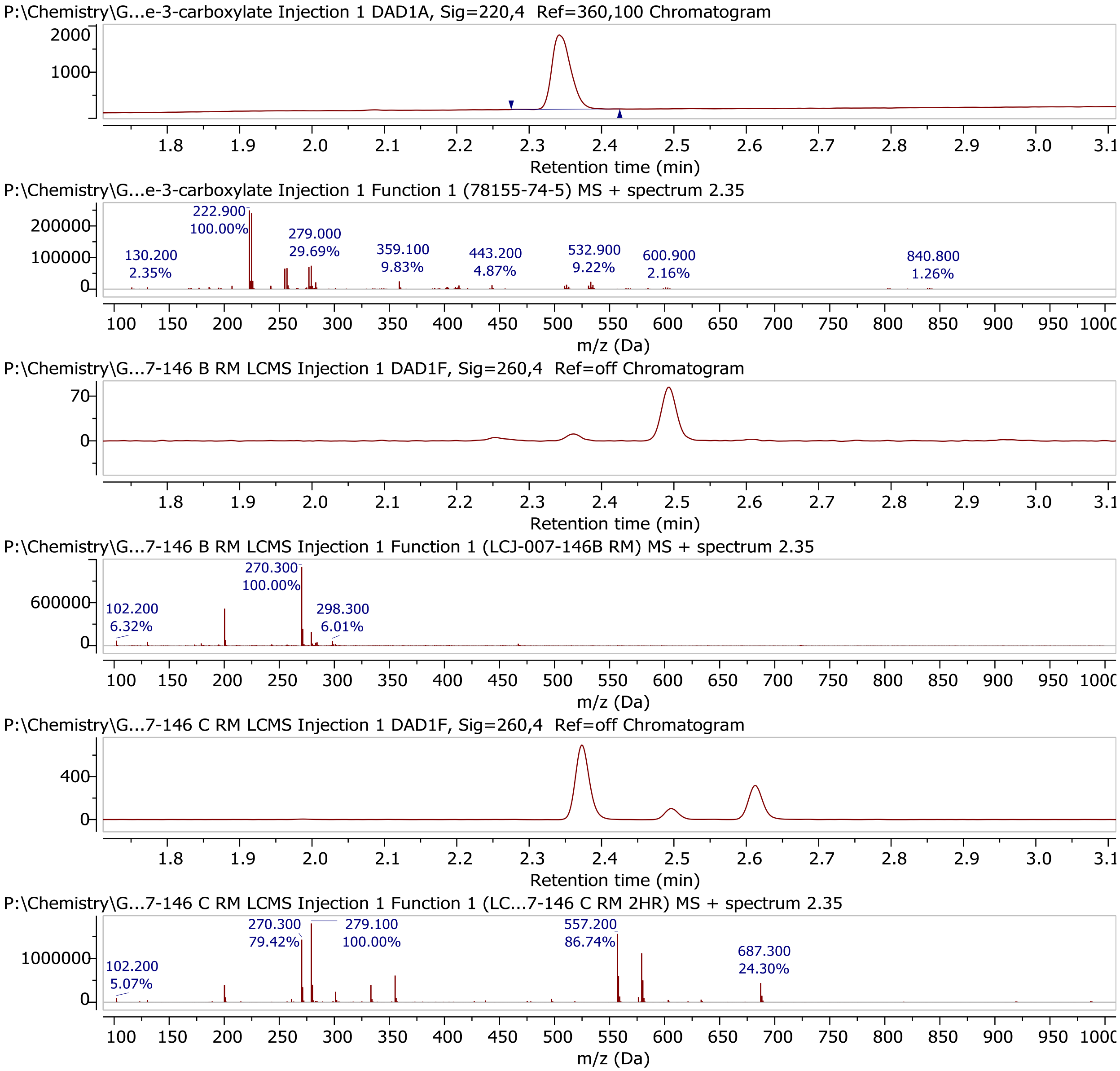

o: R = $CH_2cC_4H_7O$, 6 versus reaction mixtures. Mass spectra for reaction mixtures at 2.35 minutes for each condition.

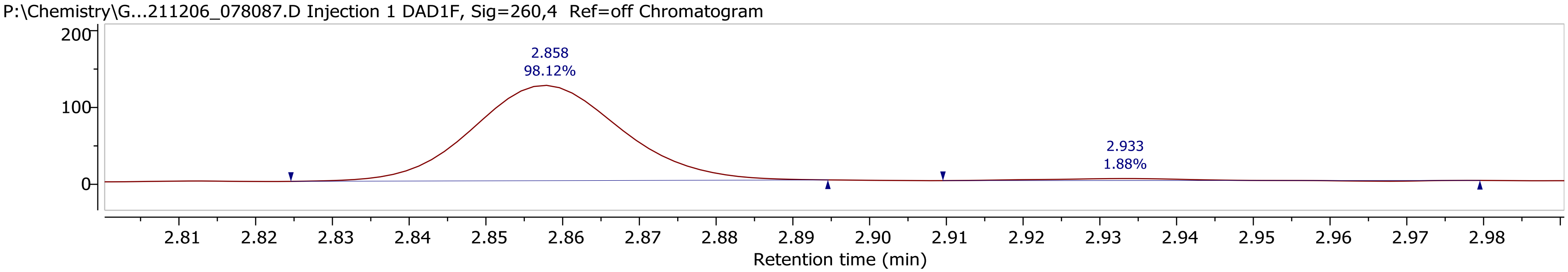
P:\Chemistry\G...211206_078087.D Injection 1 DAD1F, Sig=260,4 Ref=off Chromatogram
2.858
98.12%
2.933
1.88%
Retention time (min)

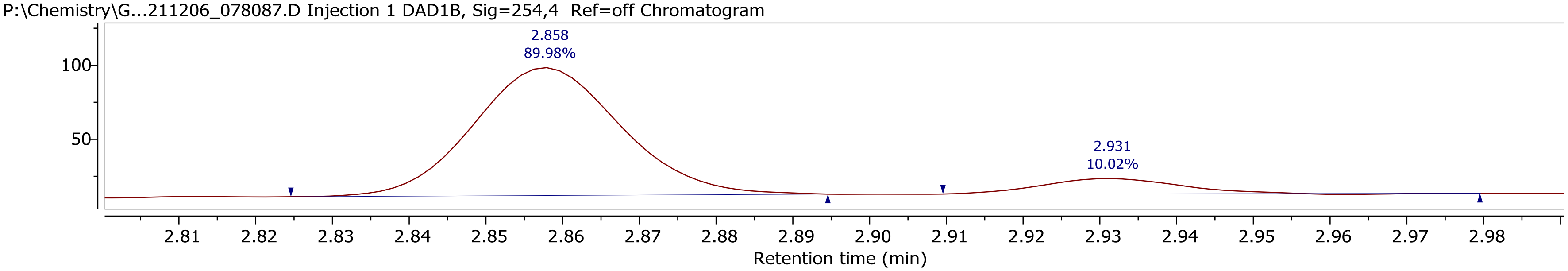
P:\Chemistry\G...211206_078087.D Injection 1 DAD1B, Sig=254,4 Ref=off Chromatogram
2.858
89.98%
2.931
10.02%
Retention time (min)

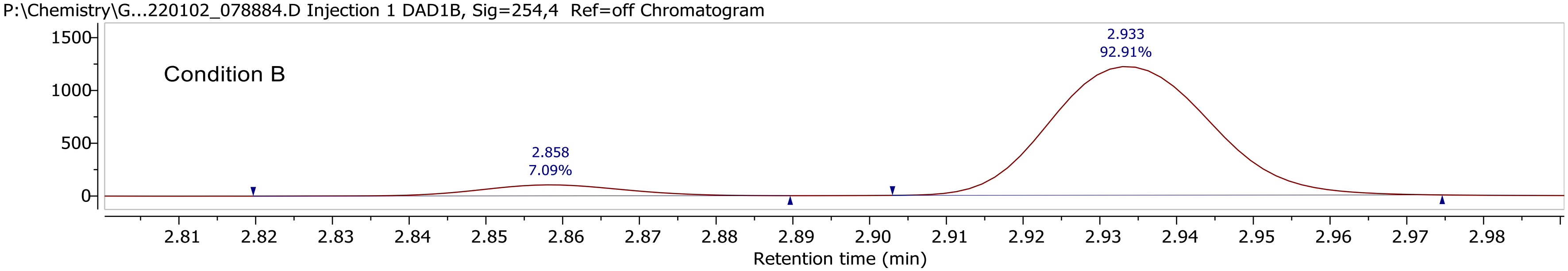
P:\Chemistry\G...220102_078884.D Injection 1 DAD1B, Sig=254,4 Ref=off Chromatogram
Condition B
2.858
7.09%
2.933
92.91%
Retention time (min)

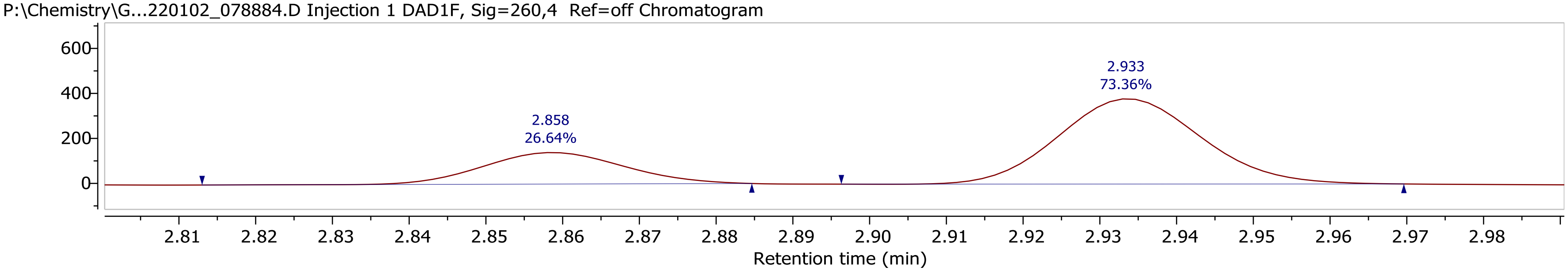
P:\Chemistry\G...220102_078884.D Injection 1 DAD1F, Sig=260,4 Ref=off Chromatogram
2.858
26.64%
2.933
73.36%
Retention time (min)

p

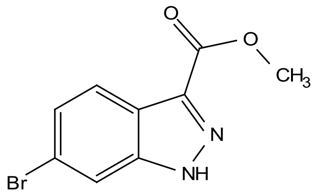

Condition A

Condition B

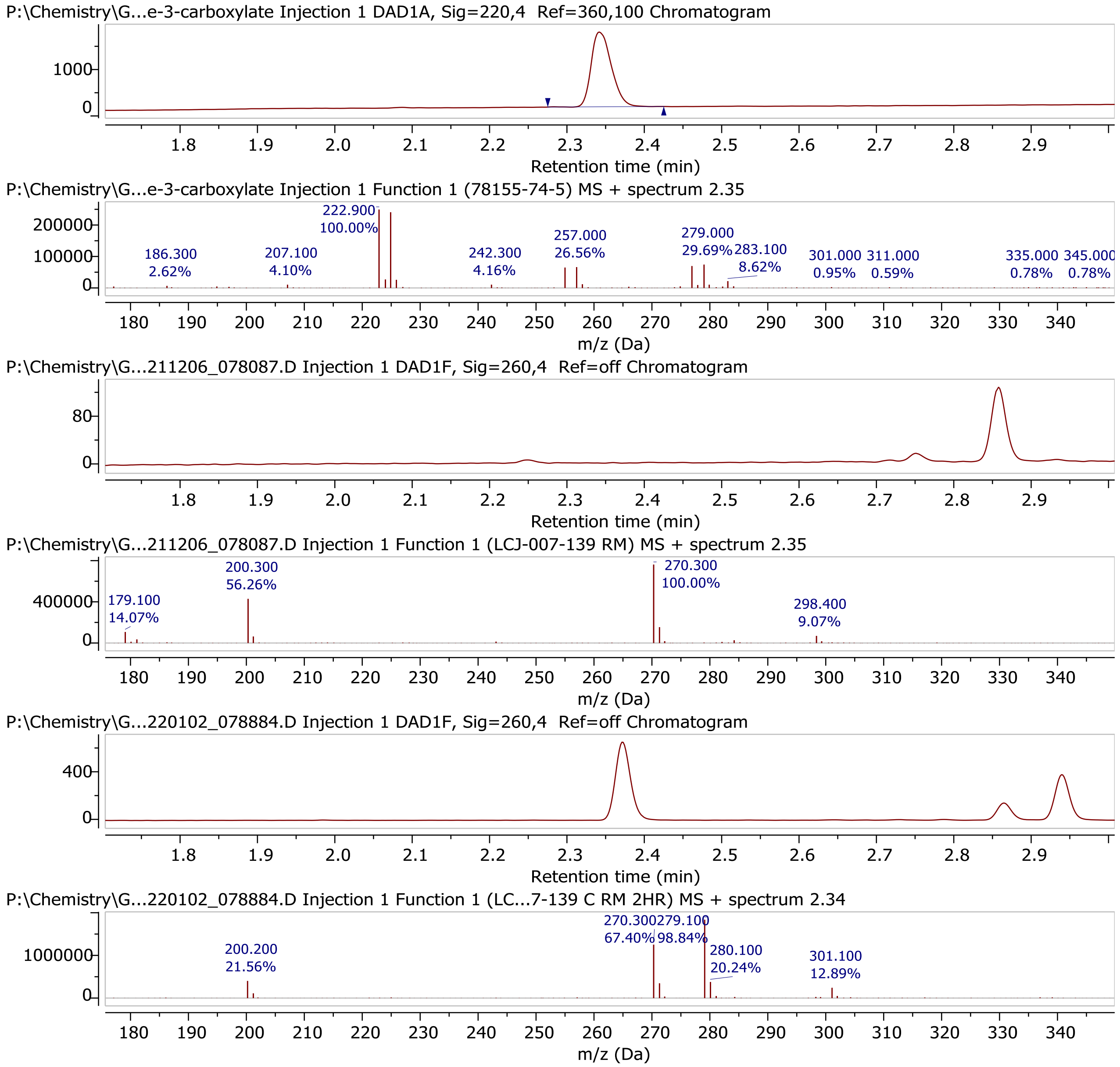

p: R = $CH_2\textit{c}C_6H_{11}$, 6 versus reaction mixtures. Mass spectra for reaction mixtures at 2.35 minutes for each condition.

P:\Chemistry\G...220802_092723.D Injection 1 DAD1F, Sig=260,4 Ref=off Chromatogram

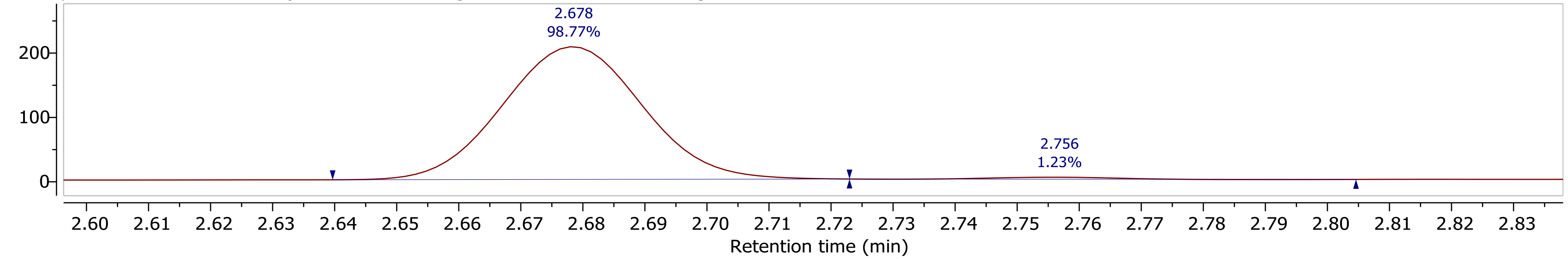

P:\Chemistry\G...220802_092723.D Injection 1 DAD1B, Sig=254,4 Ref=off Chromatogram

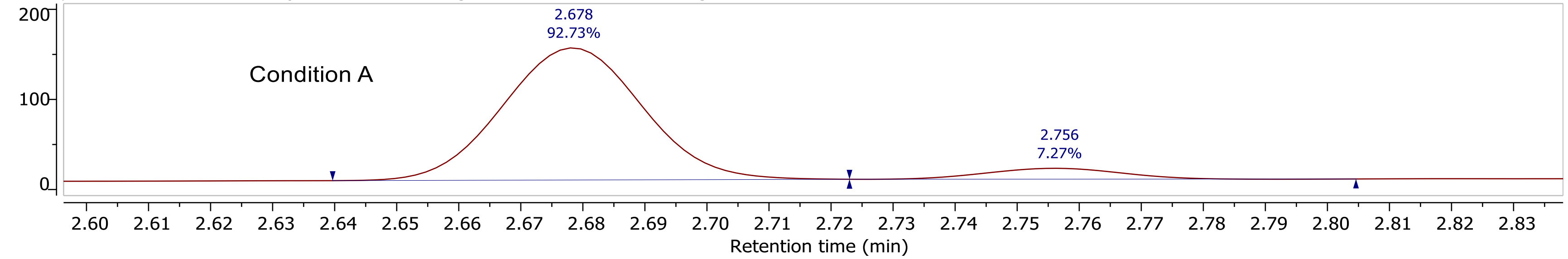

P:\Chemistry\G...220802_092724.D Injection 1 DAD1F, Sig=260,4 Ref=off Chromatogram

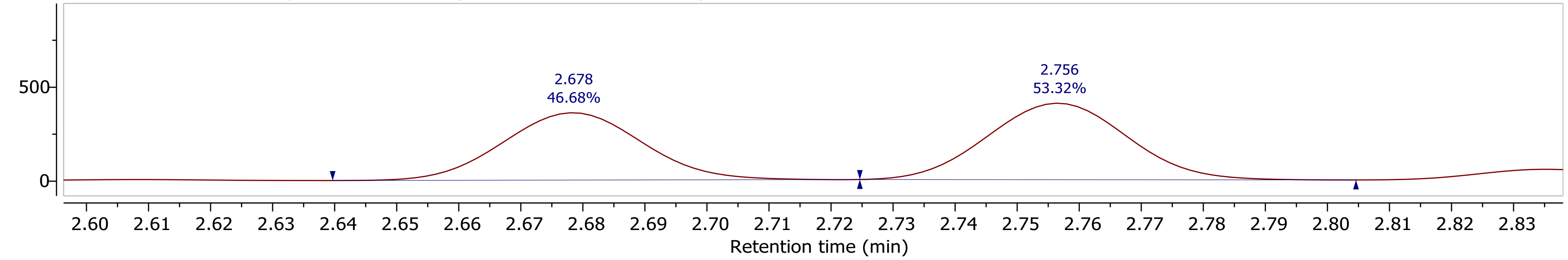

P:\Chemistry\G...220802_092724.D Injection 1 DAD1B, Sig=254,4 Ref=off Chromatogram

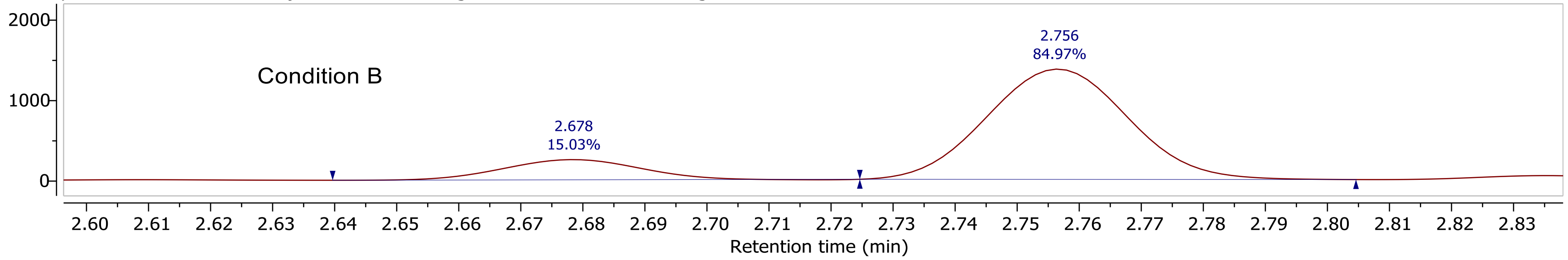

q

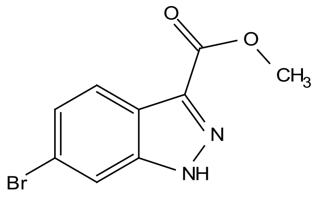

Condition A

Condition B

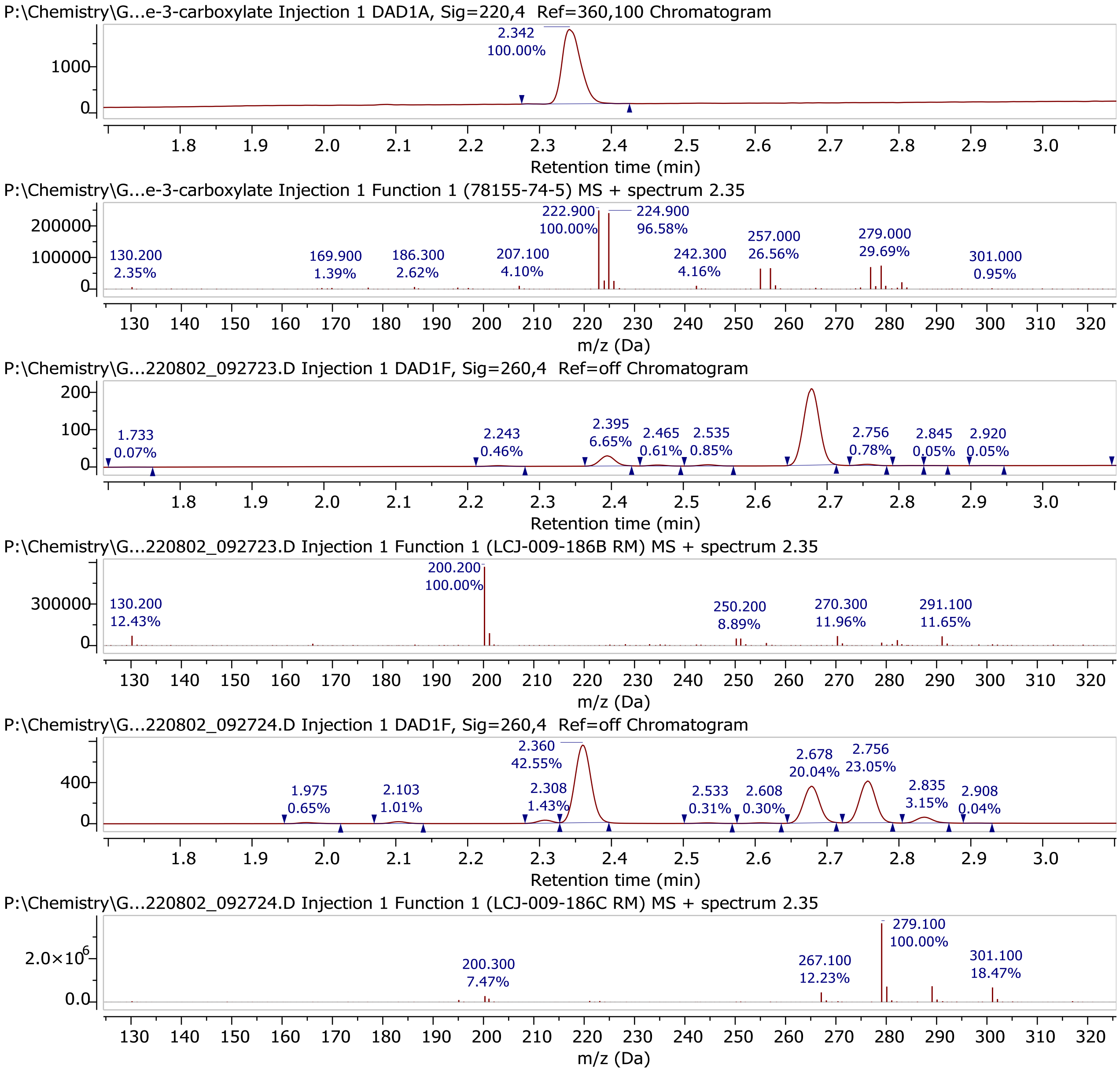

q: R = Bn, 6 versus reaction mixtures. Mass spectra for reaction mixtures at 2.35 minutes for each condition

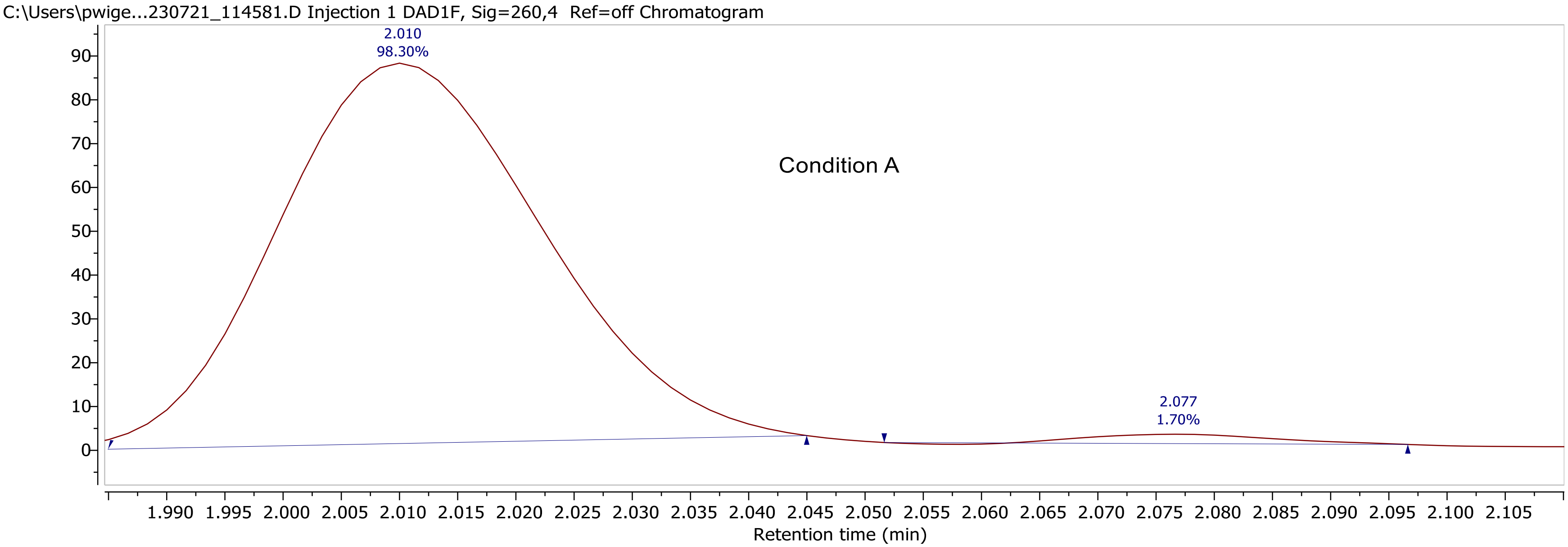
C:\Users\pwige...230721_114581.D Injection 1 DAD1F, Sig=260,4 Ref=off Chromatogram
2.010
98.30%
Condition A
2.077
1.70%
Retention time (min)

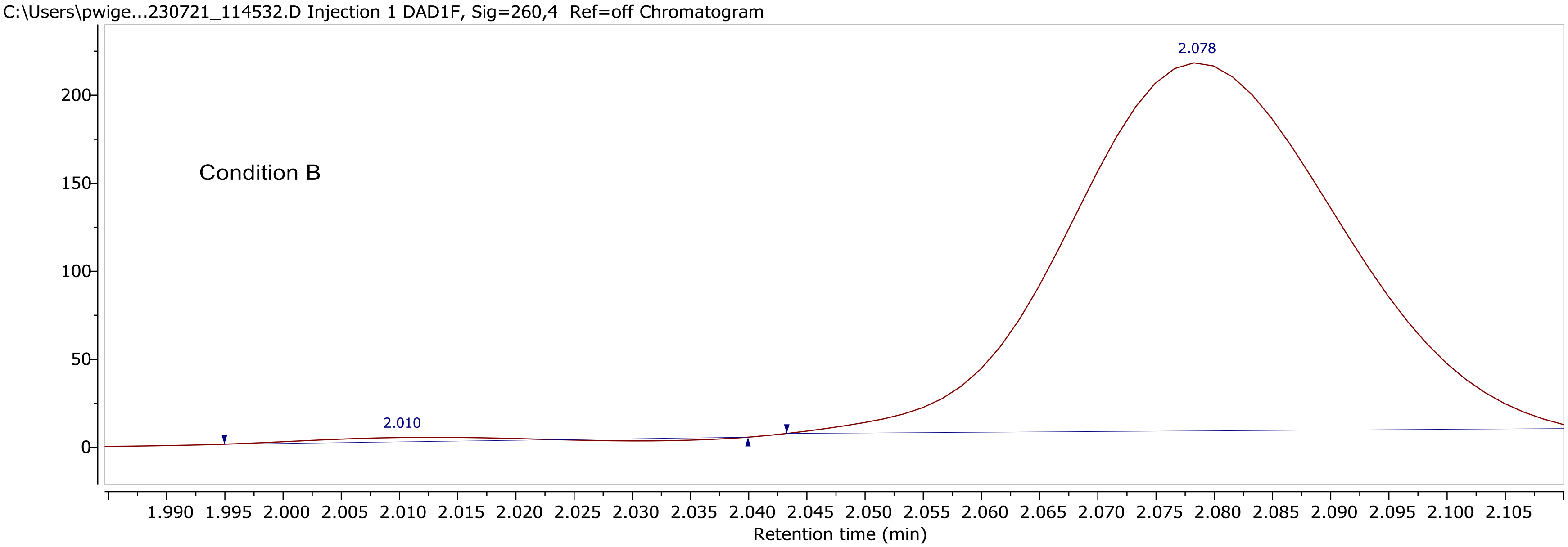
C:\Users\pwige...230721_114532.D Injection 1 DAD1F, Sig=260,4 Ref=off Chromatogram
2.078
Condition B
2.010
Retention time (min)

C:\Users\pwige...230716_111844.D Injection 1 DAD1F, Sig=260,4 Ref=off Chromatogram

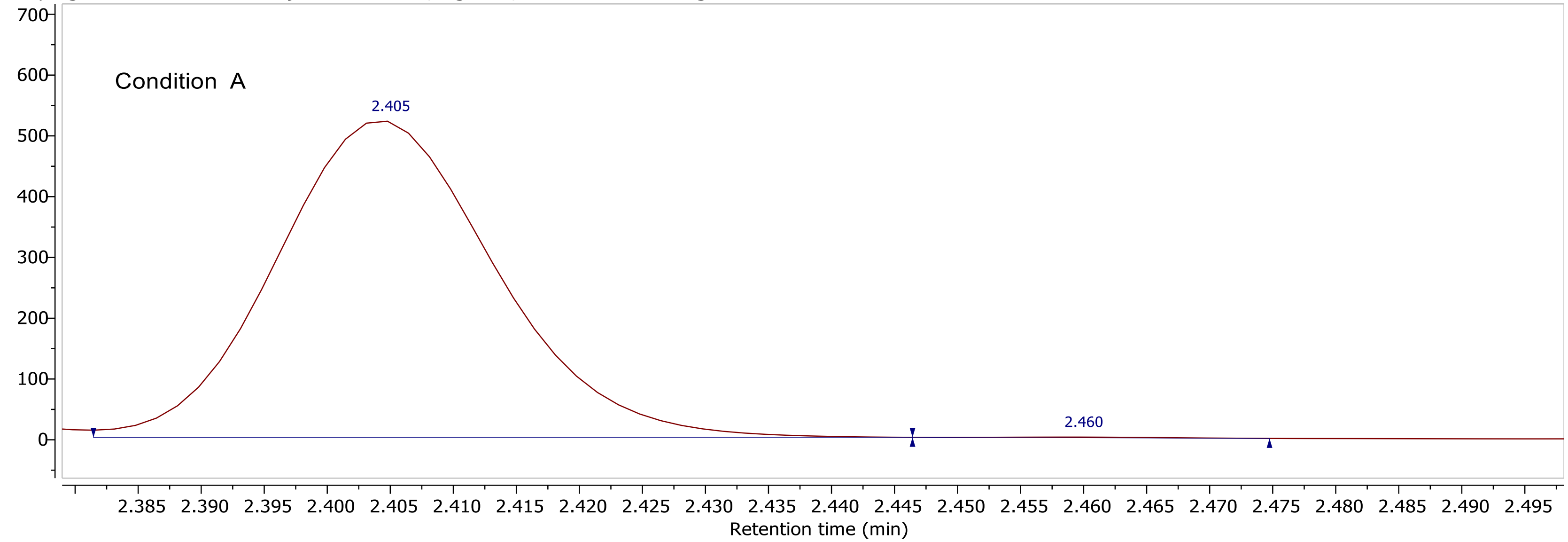

C:\Users\pwige...230716_111847.D Injection 1 DAD1F, Sig=260,4 Ref=off Chromatogram

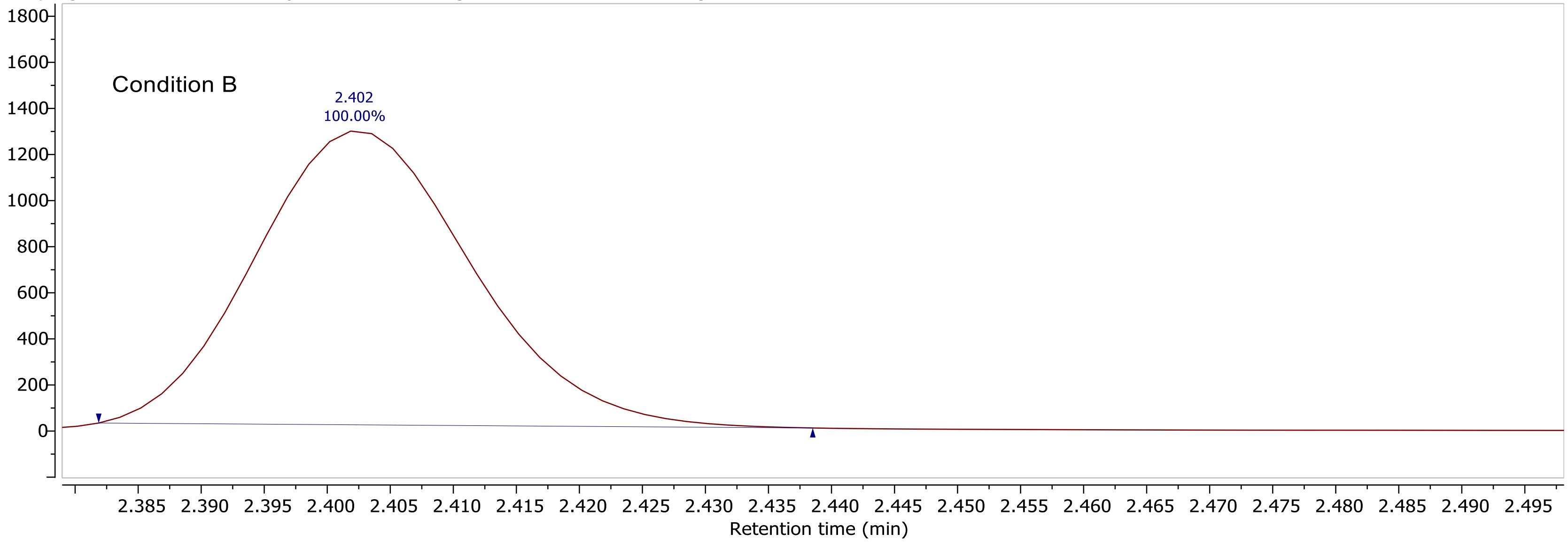

22

NMR Spectra of synthesized compounds.

14b-^{1}H

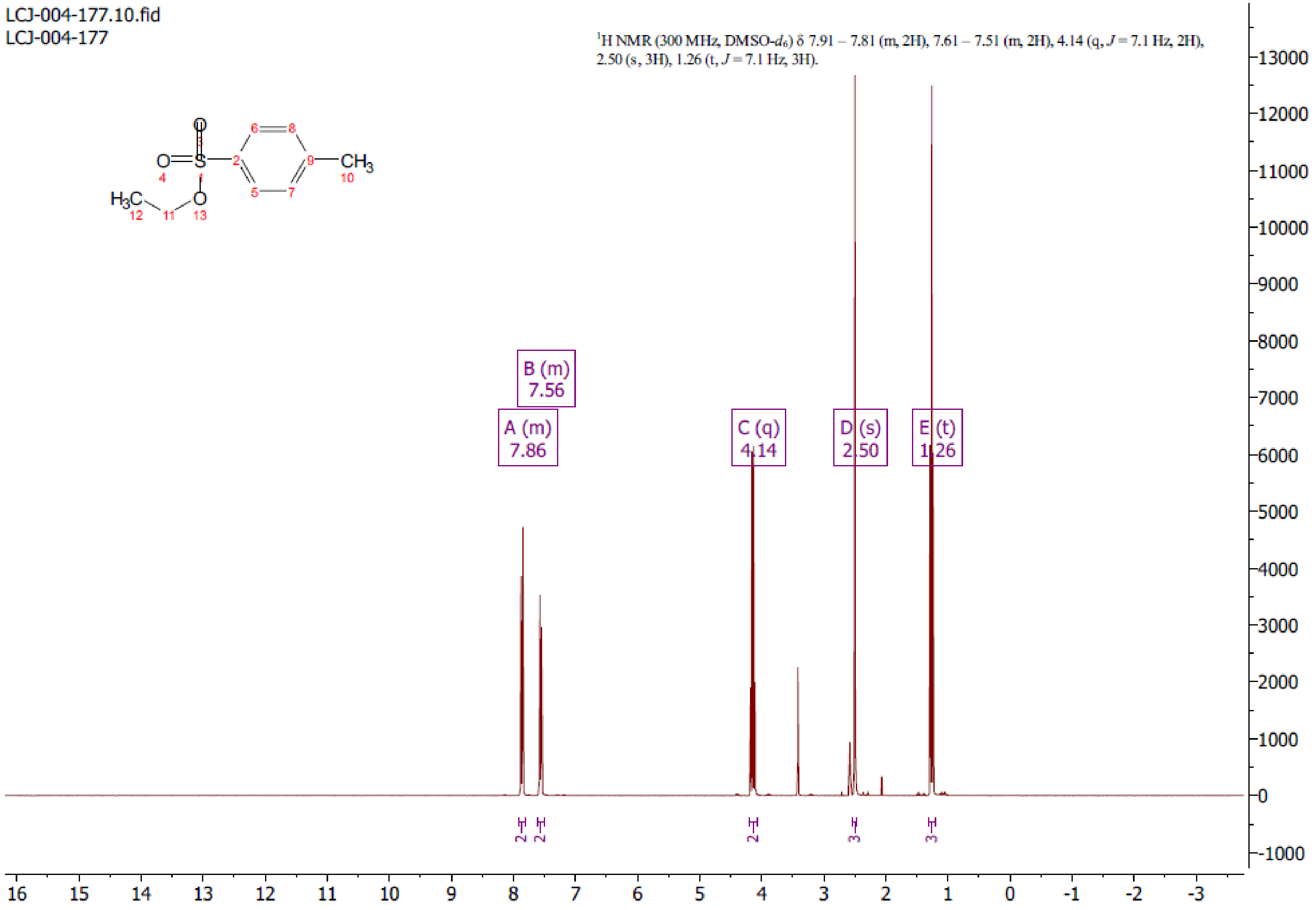

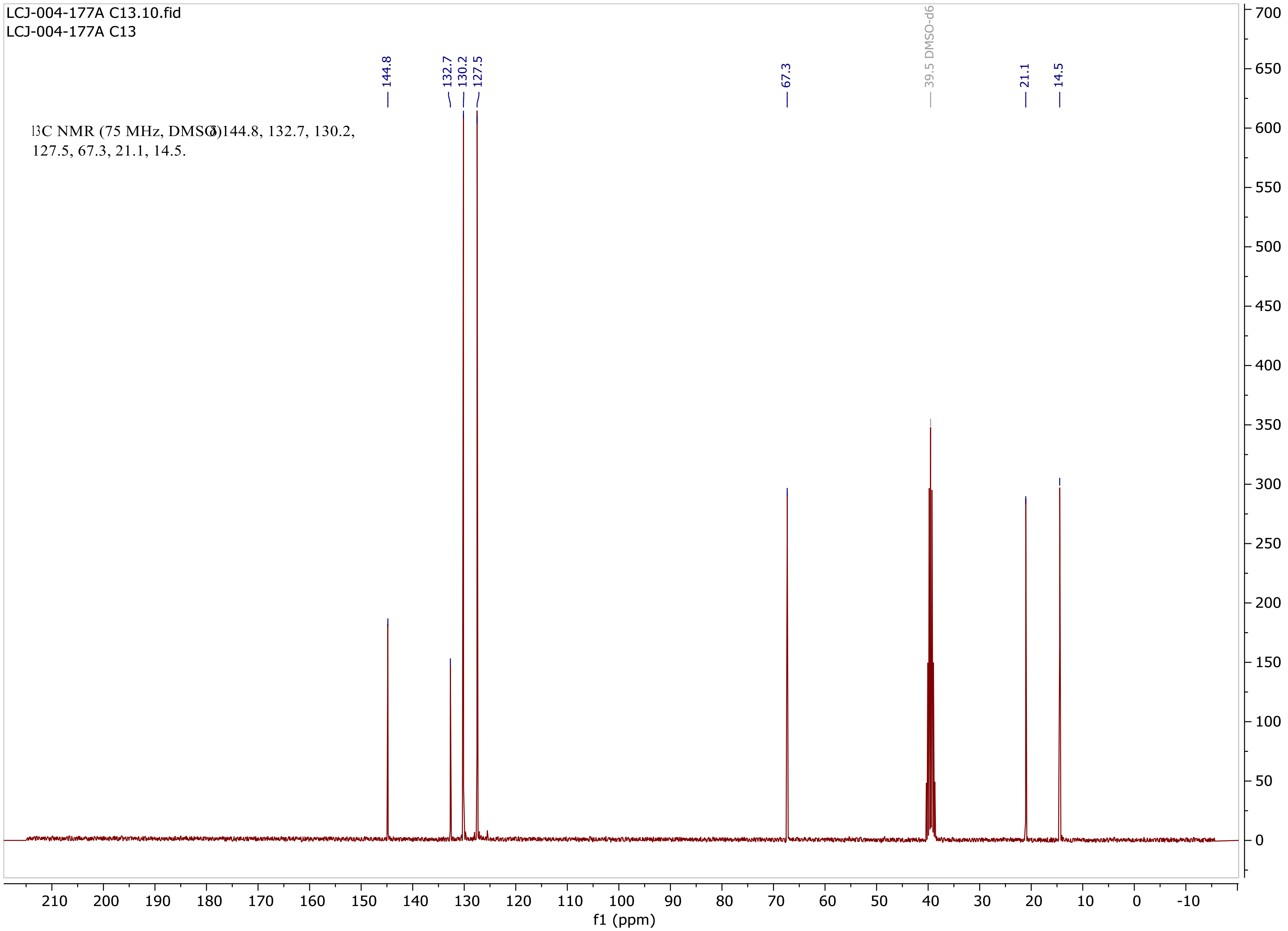
LCJ-004-177A C13.10.fid
LCJ-004-177A C13
144.8
132.7
130.2
127.5
67.3
39.5 DMSO-d6
21.1
14.5
13C NMR (75 MHz, DMSO δ) 144.8, 132.7, 130.2, 127.5, 67.3, 21.1, 14.5.
700
650
600
550
500
450
400
350
300
250
200
150
100
50
0
210
200
190
180
170
160
150
140
130
120
110
100
90
80
70
60
50
40
30
20
10
0
-10
f1 (ppm)

14b-^{13}C

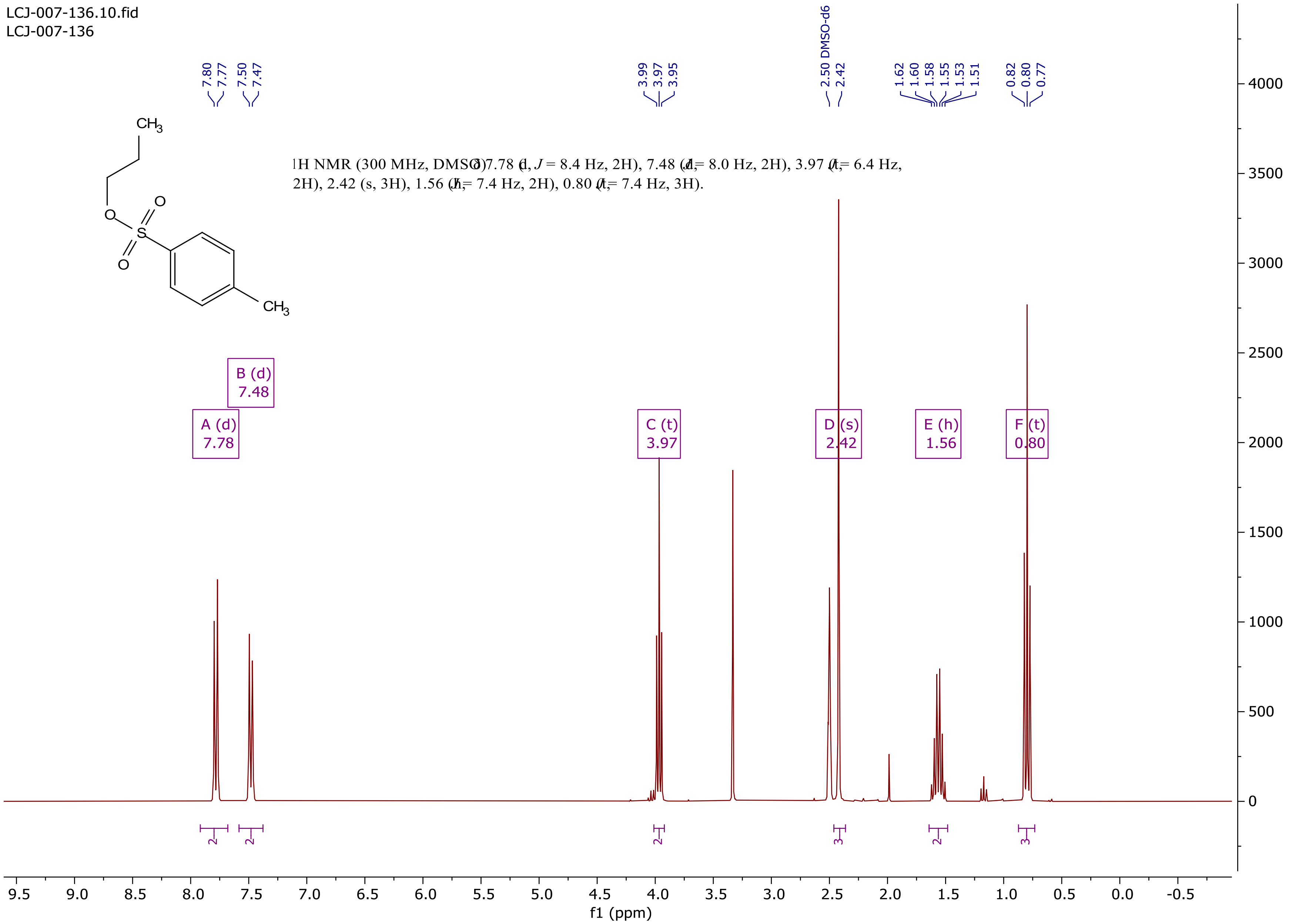
LCJ-007-136.10.fid
LCJ-007-136
7.80
7.77
7.50
7.47
3.99
3.97
3.95
2.50 DMSO-d6
2.42
1.62
1.60
1.58
1.55
1.53
1.51
0.82
0.80
0.77
CH3
O
S
O
O
CH3
1H NMR (300 MHz, DMSO) δ 7.78 (d, J = 8.4 Hz, 2H), 7.48 (d, J = 8.0 Hz, 2H), 3.97 (t, J = 6.4 Hz, 2H), 2.42 (s, 3H), 1.56 (h, J = 7.4 Hz, 2H), 0.80 (t, J = 7.4 Hz, 3H).
B (d) 7.48
A (d) 7.78
C (t) 3.97
D (s) 2.42
E (h) 1.56
F (t) 0.80
2
2
2
3
2
3
f1 (ppm)

14d-^{1}H

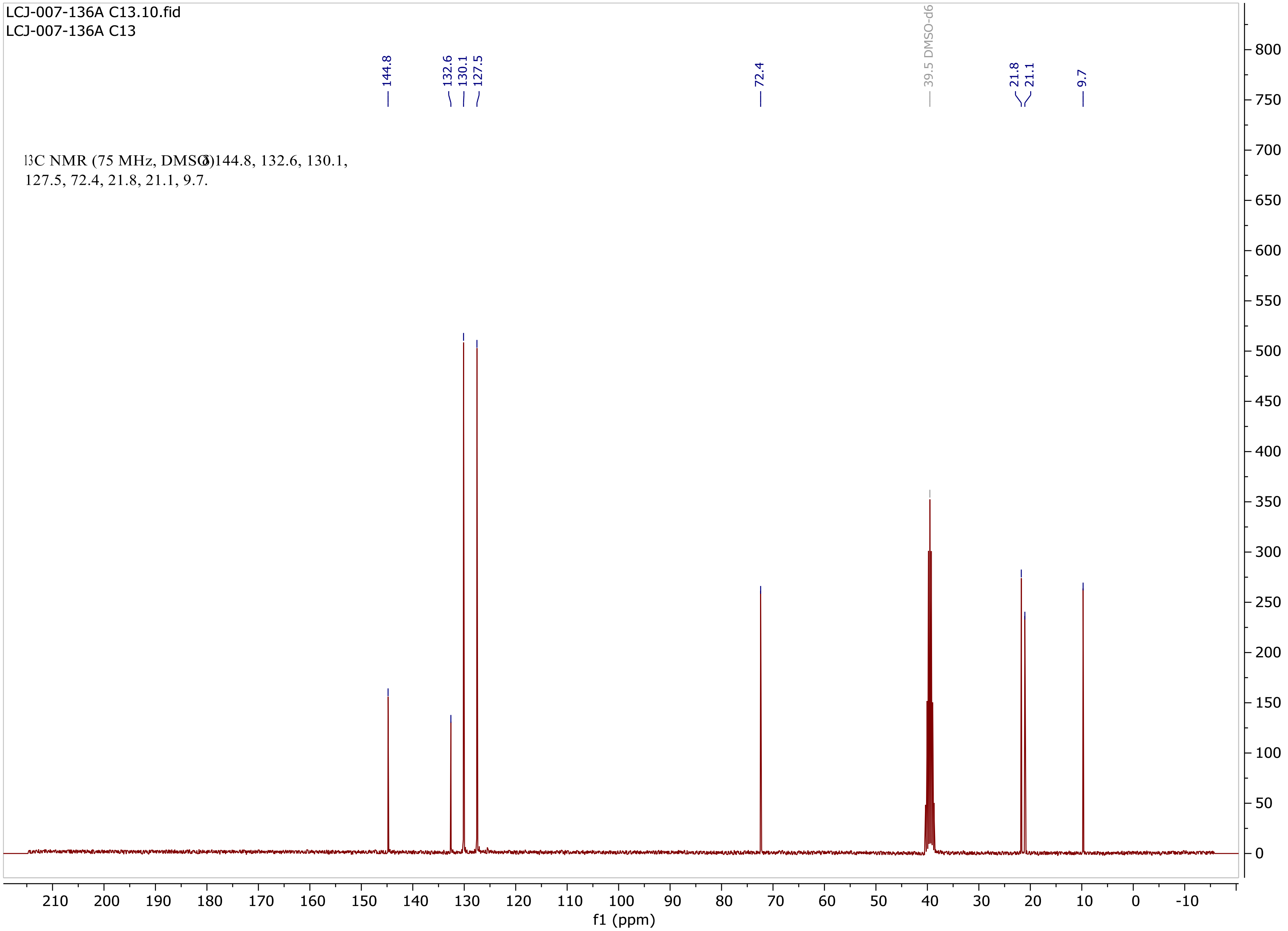

LCJ-007-136A C13.10.fid
LCJ-007-136A C13
144.8
132.6
130.1
127.5
72.4
39.5 DMSO-d6
21.8
21.1
9.7
13C NMR (75 MHz, DMSO) δ 144.8, 132.6, 130.1,
127.5, 72.4, 21.8, 21.1, 9.7.
f1 (ppm)

14d-^{13}C

14e-^{1}H

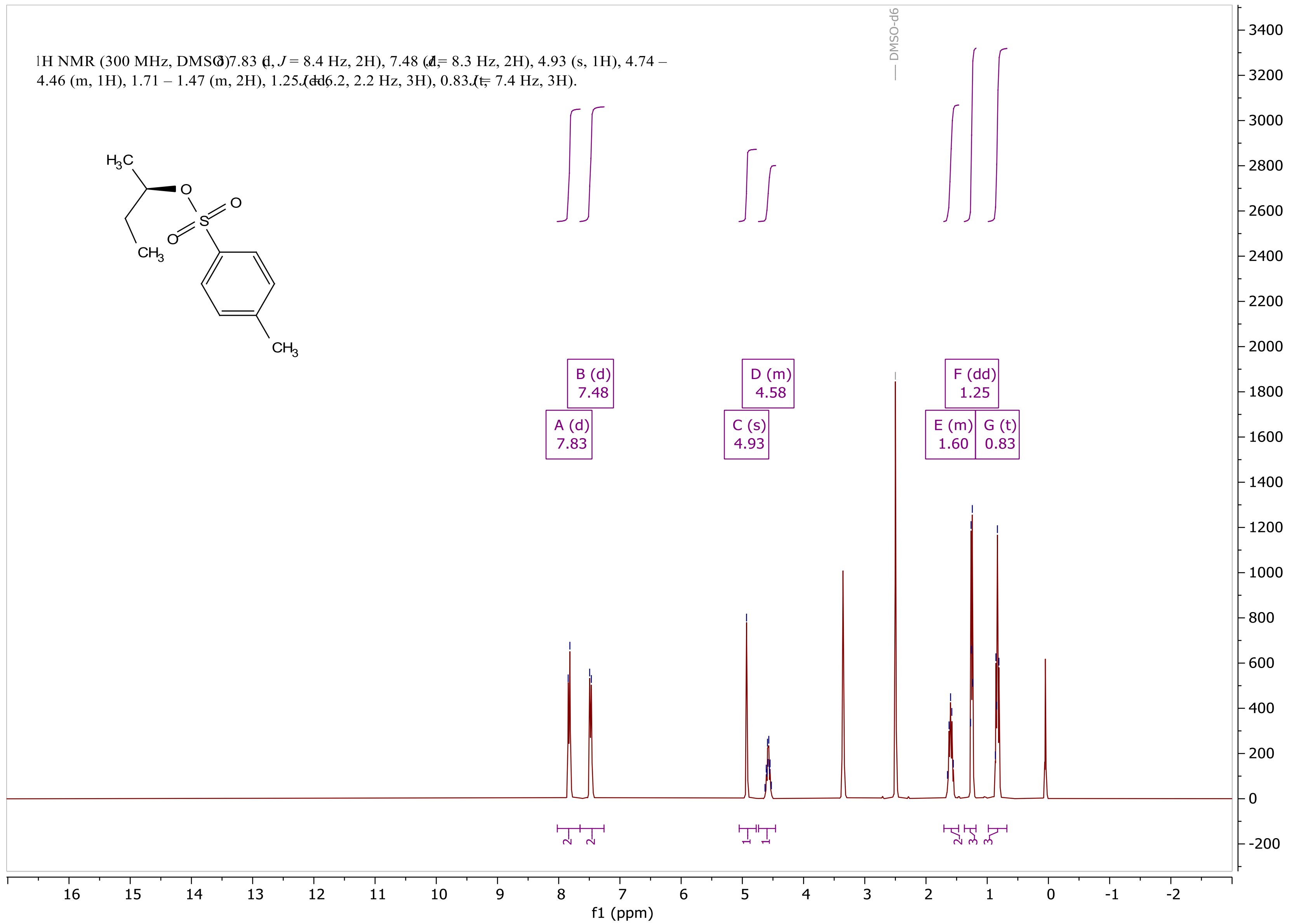

1H NMR (300 MHz, DMSO-d6) δ 7.83 (d, J = 8.4 Hz, 2H), 7.48 (d, J = 8.3 Hz, 2H), 4.93 (s, 1H), 4.74 – 4.46 (m, 1H), 1.71 – 1.47 (m, 2H), 1.25 (dd, J = 6.2, 2.2 Hz, 3H), 0.83 (t, J = 7.4 Hz, 3H).
DMSO-d6
B (d) 7.48
A (d) 7.83
D (m) 4.58
C (s) 4.93
F (dd) 1.25
E (m) 1.60
G (t) 0.83
f1 (ppm)

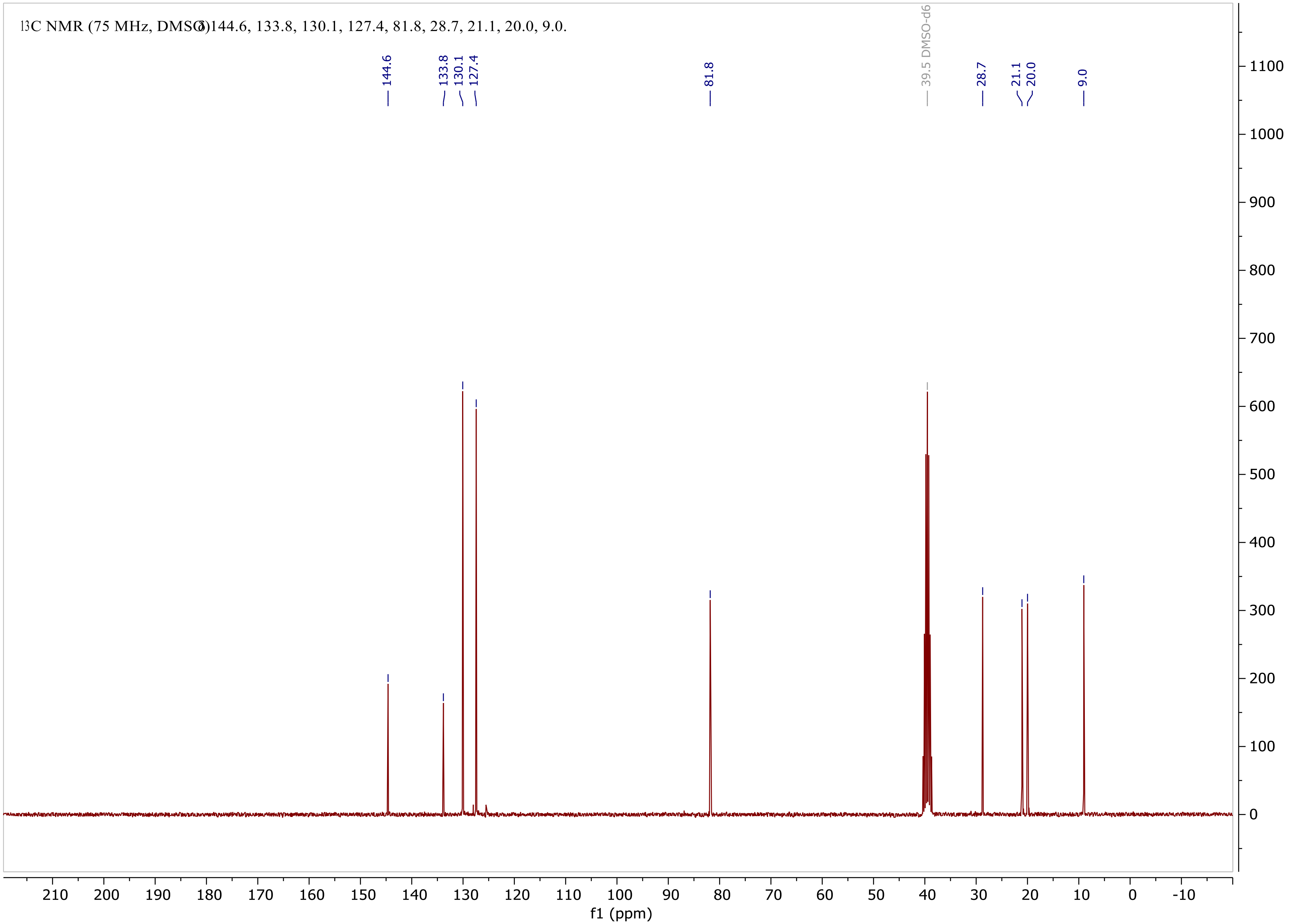
13C NMR (75 MHz, DMSO) δ 144.6, 133.8, 130.1, 127.4, 81.8, 28.7, 21.1, 20.0, 9.0.
144.6
133.8
130.1
127.4
81.8
39.5 DMSO-d6
28.7
21.1
20.0
9.0
f1 (ppm)

14e-^{13}C

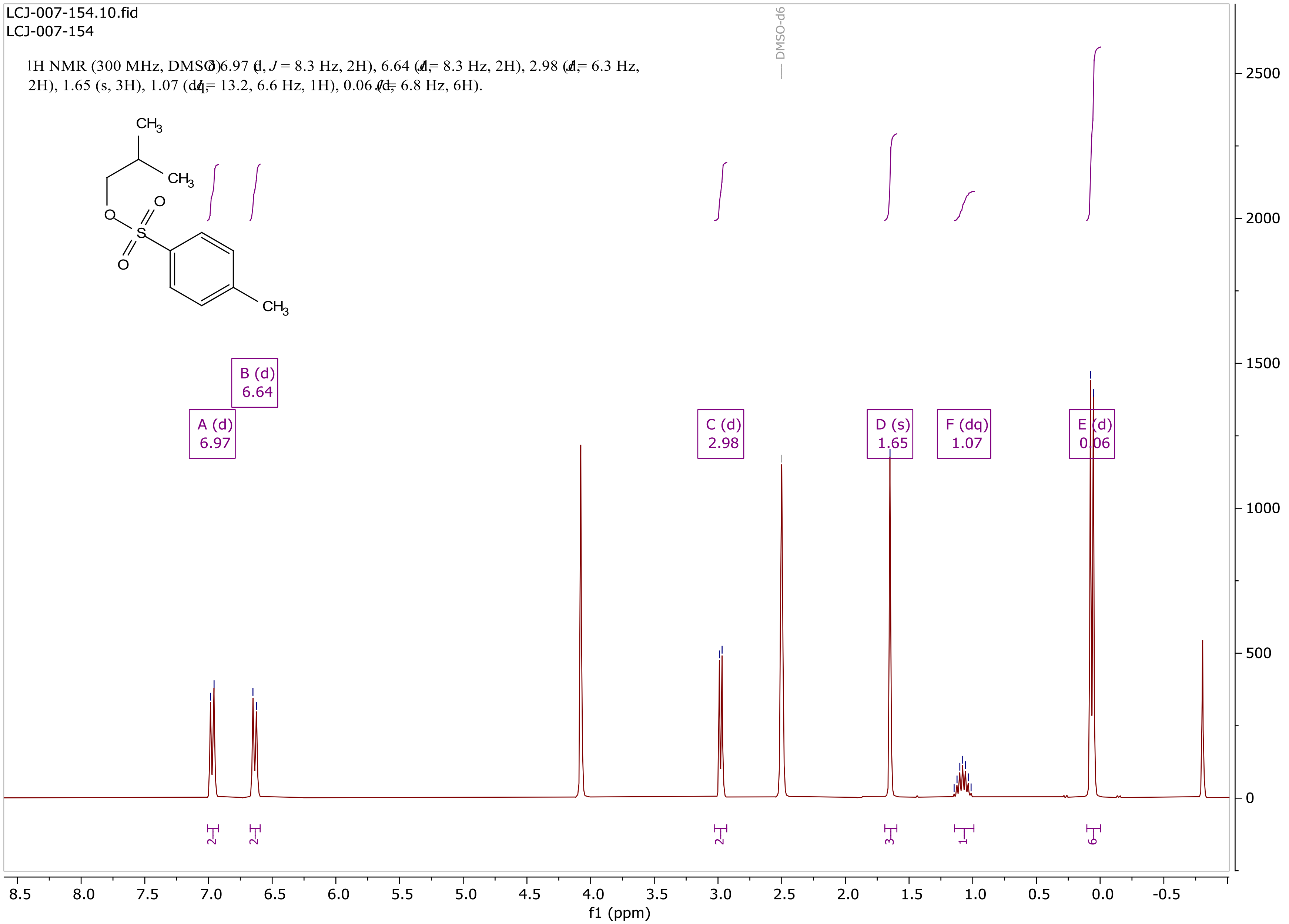
LCJ-007-154.10.fid
LCJ-007-154
1H NMR (300 MHz, DMSO) δ 6.97 (d, J = 8.3 Hz, 2H), 6.64 (d, J = 8.3 Hz, 2H), 2.98 (d, J = 6.3 Hz, 2H), 1.65 (s, 3H), 1.07 (dd, J = 13.2, 6.6 Hz, 1H), 0.06 (d, J = 6.8 Hz, 6H).
DMSO-d6
A (d) 6.97
B (d) 6.64
C (d) 2.98
D (s) 1.65
F (dq) 1.07
E (d) 0.06
f1 (ppm)

14f-^{1}H

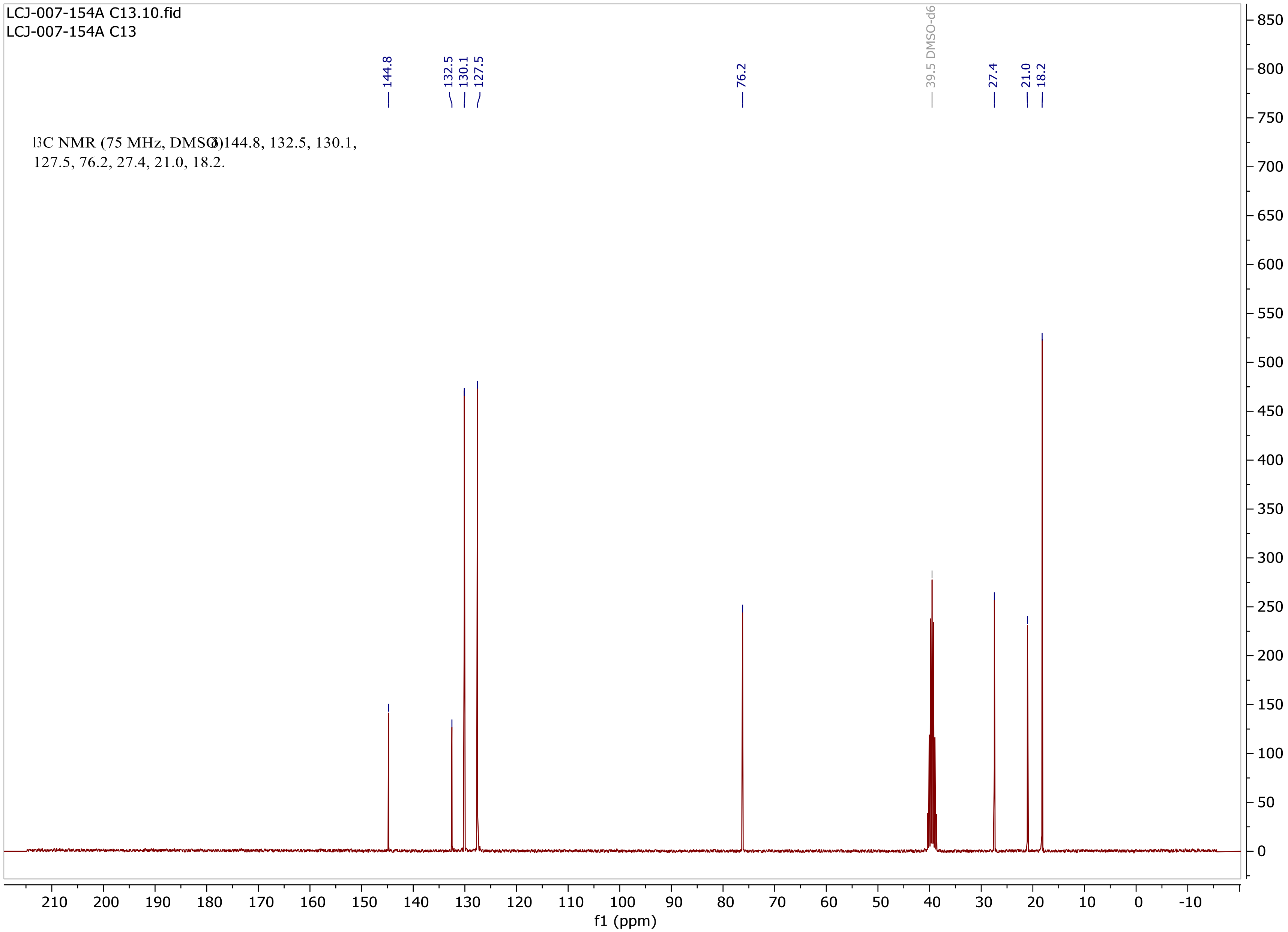

LCJ-007-154A C13.10.fid
LCJ-007-154A C13
144.8
132.5
130.1
127.5
76.2
39.5 DMSO-d6
27.4
21.0
18.2
13C NMR (75 MHz, DMSO) δ 144.8, 132.5, 130.1, 127.5, 76.2, 27.4, 21.0, 18.2.
f1 (ppm)

14f-^{13}C

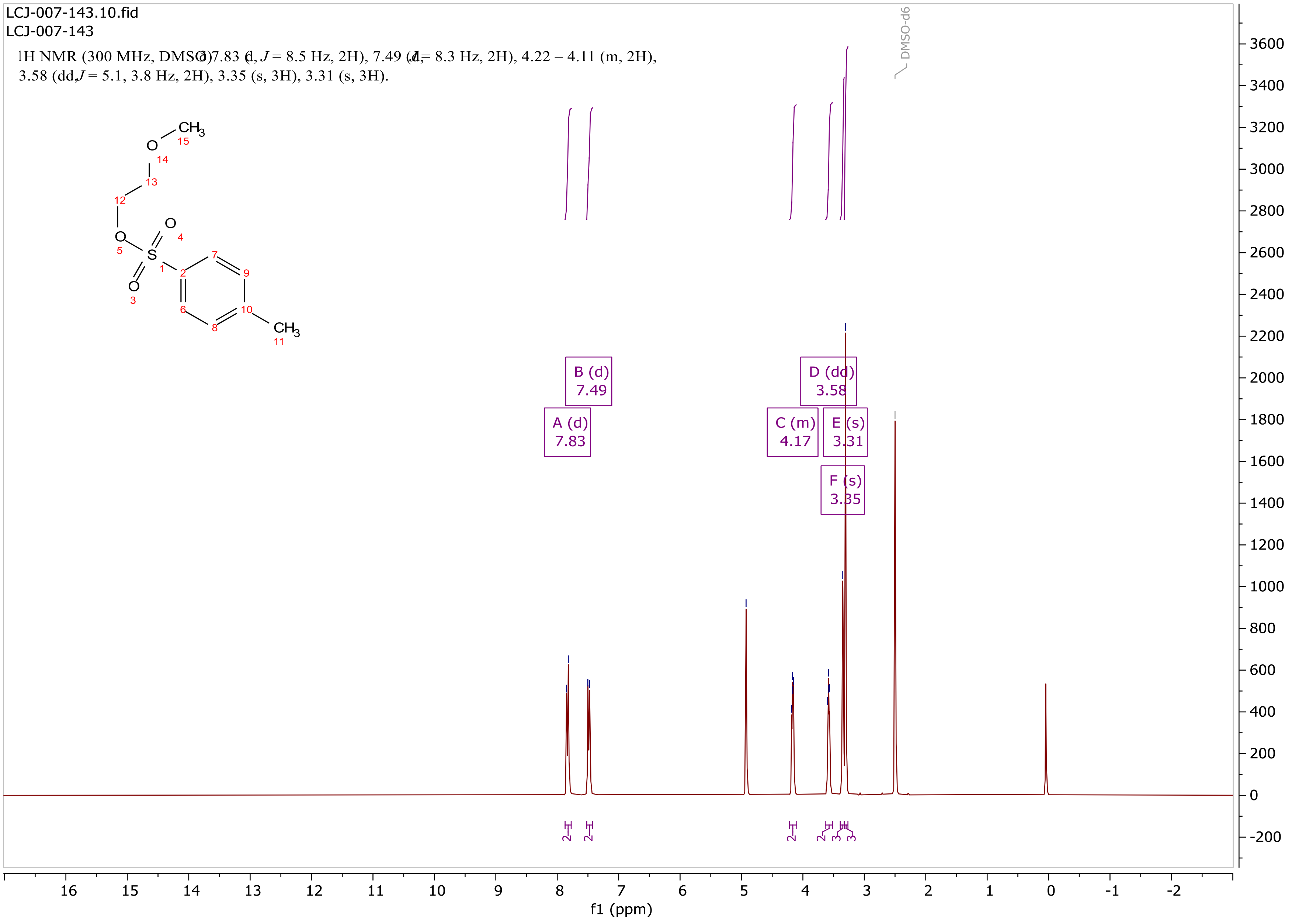
LCJ-007-143.10.fid
LCJ-007-143
1H NMR (300 MHz, DMSO-d6) δ 7.83 (d, J = 8.5 Hz, 2H), 7.49 (d, J = 8.3 Hz, 2H), 4.22 – 4.11 (m, 2H), 3.58 (dd, J = 5.1, 3.8 Hz, 2H), 3.35 (s, 3H), 3.31 (s, 3H).
DMSO-d6
A (d) 7.83
B (d) 7.49
C (m) 4.17
D (dd) 3.58
E (s) 3.31
F (s) 3.35
f1 (ppm)

14g-^{1}H

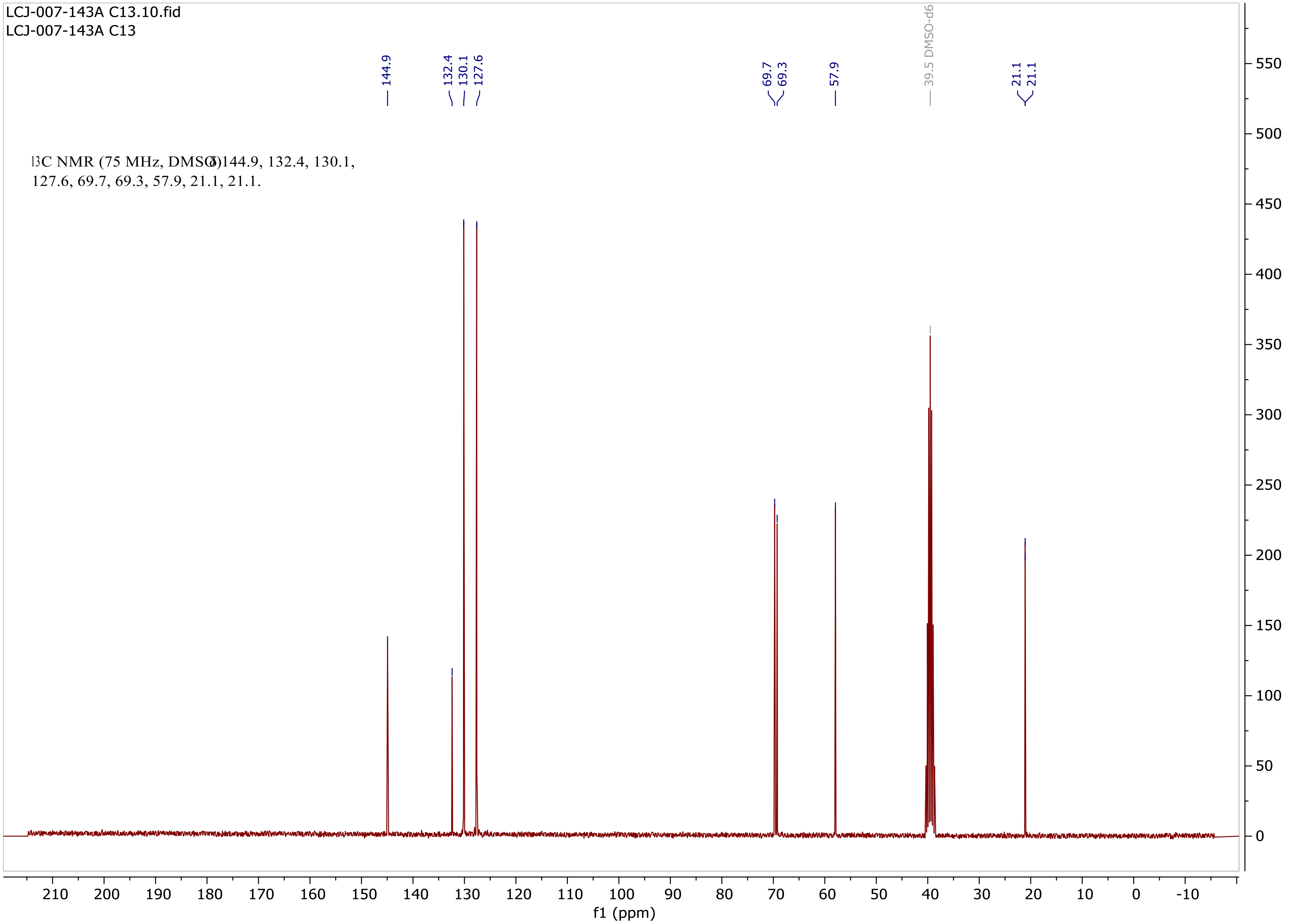
LCJ-007-143A C13.10.fid
LCJ-007-143A C13
144.9
132.4
130.1
127.6
69.7
69.3
57.9
39.5 DMSO-d6
21.1
21.1
13C NMR (75 MHz, DMSO) δ 144.9, 132.4, 130.1, 127.6, 69.7, 69.3, 57.9, 21.1, 21.1.
f1 (ppm)

14g-^{13}C

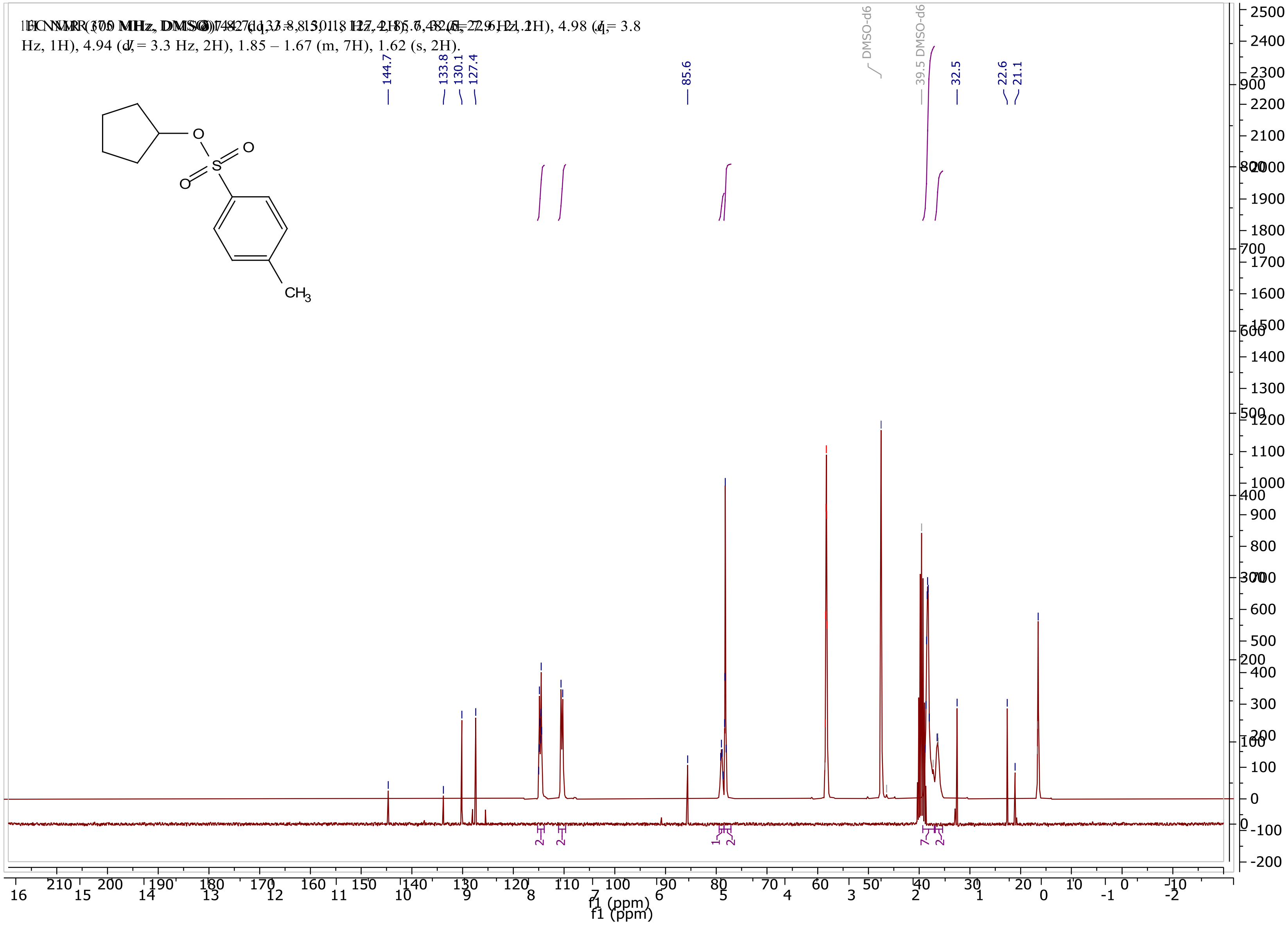

14h-^{1}H

14h-^{13}C

LCJ-007-151.10.fid
LCJ-007-151

1H NMR (300 MHz, DMSO) δ 7.85 (d, J = 8.3 Hz, 2H), 7.50 (d, J = 8.2 Hz, 2H), 5.14 – 5.05 (m, 1H), 4.92 (s, 2H), 3.56 – 3.26 (m, 7H), 2.06 (d, J = 9.7 Hz, 2H), 1.47 (d, J = 7.2 Hz, 8H).

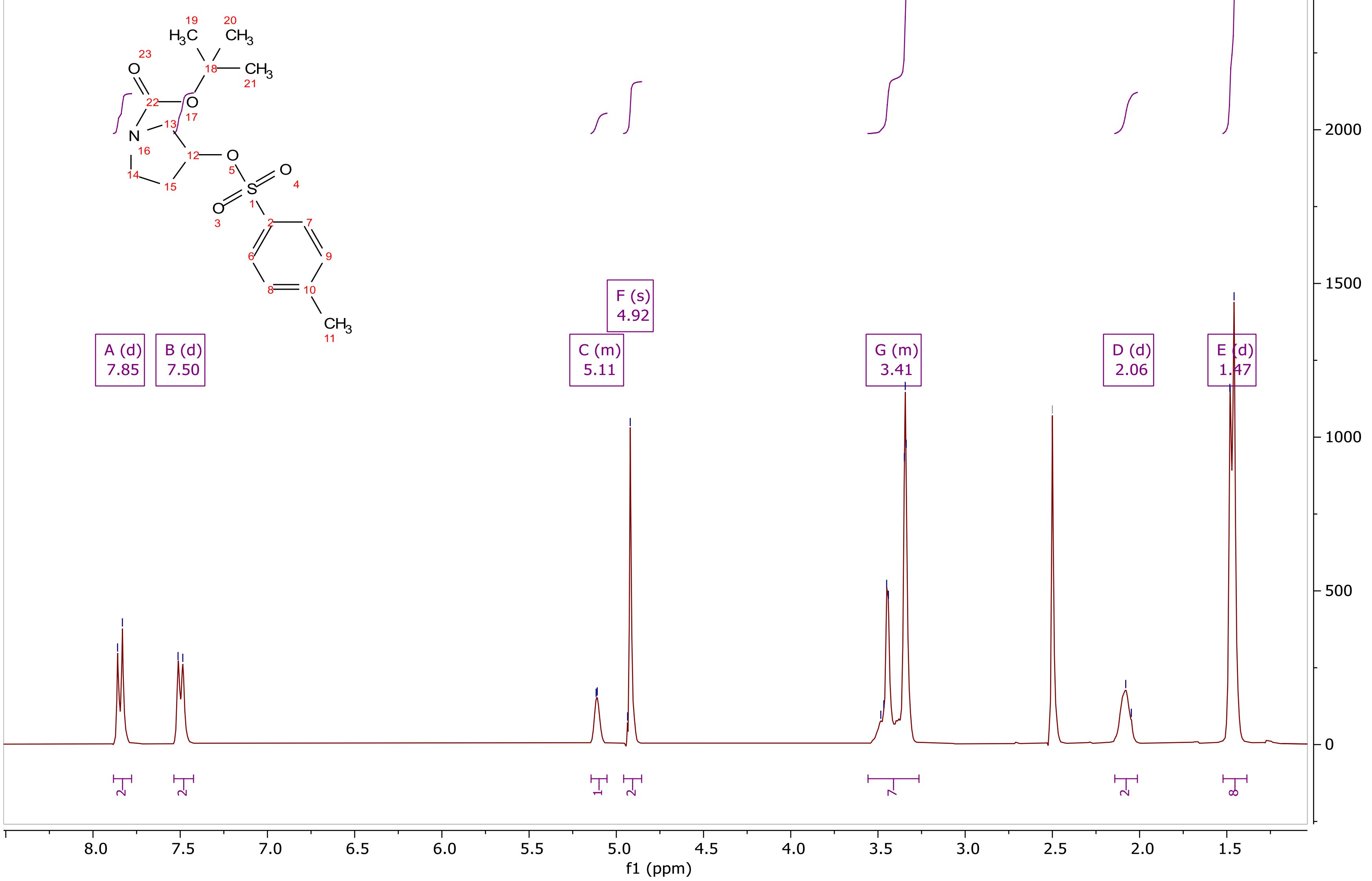

14i-^{1}H

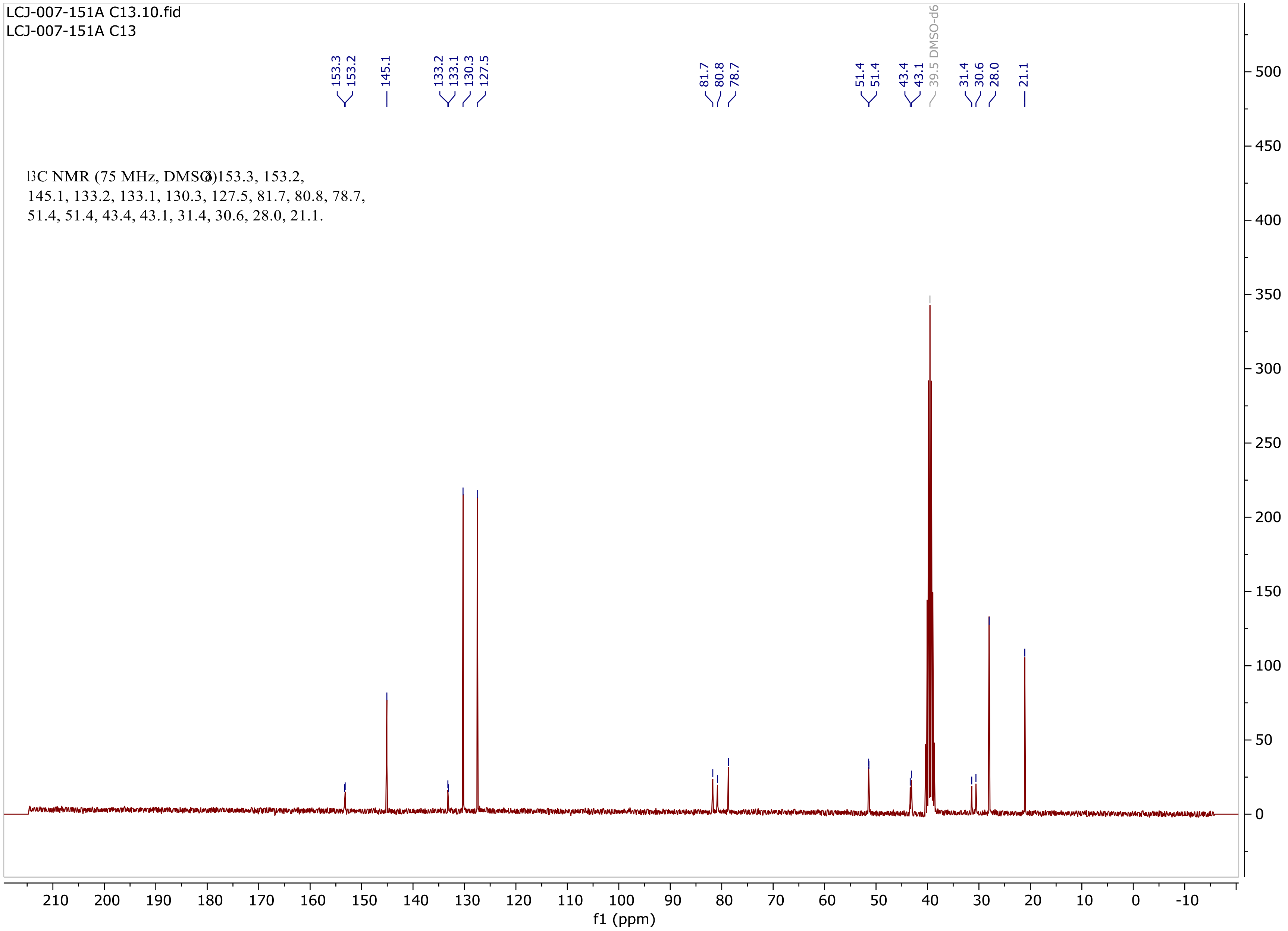

LCJ-007-151A C13.10.fid
LCJ-007-151A C13
153.3
153.2
145.1
133.2
133.1
130.3
127.5
81.7
80.8
78.7
51.4
51.4
43.4
43.1
39.5 DMSO-d6
31.4
30.6
28.0
21.1
13C NMR (75 MHz, DMSO) δ 153.3, 153.2,
145.1, 133.2, 133.1, 130.3, 127.5, 81.7, 80.8, 78.7,
51.4, 51.4, 43.4, 43.1, 31.4, 30.6, 28.0, 21.1.
f1 (ppm)

14i-^{13}C

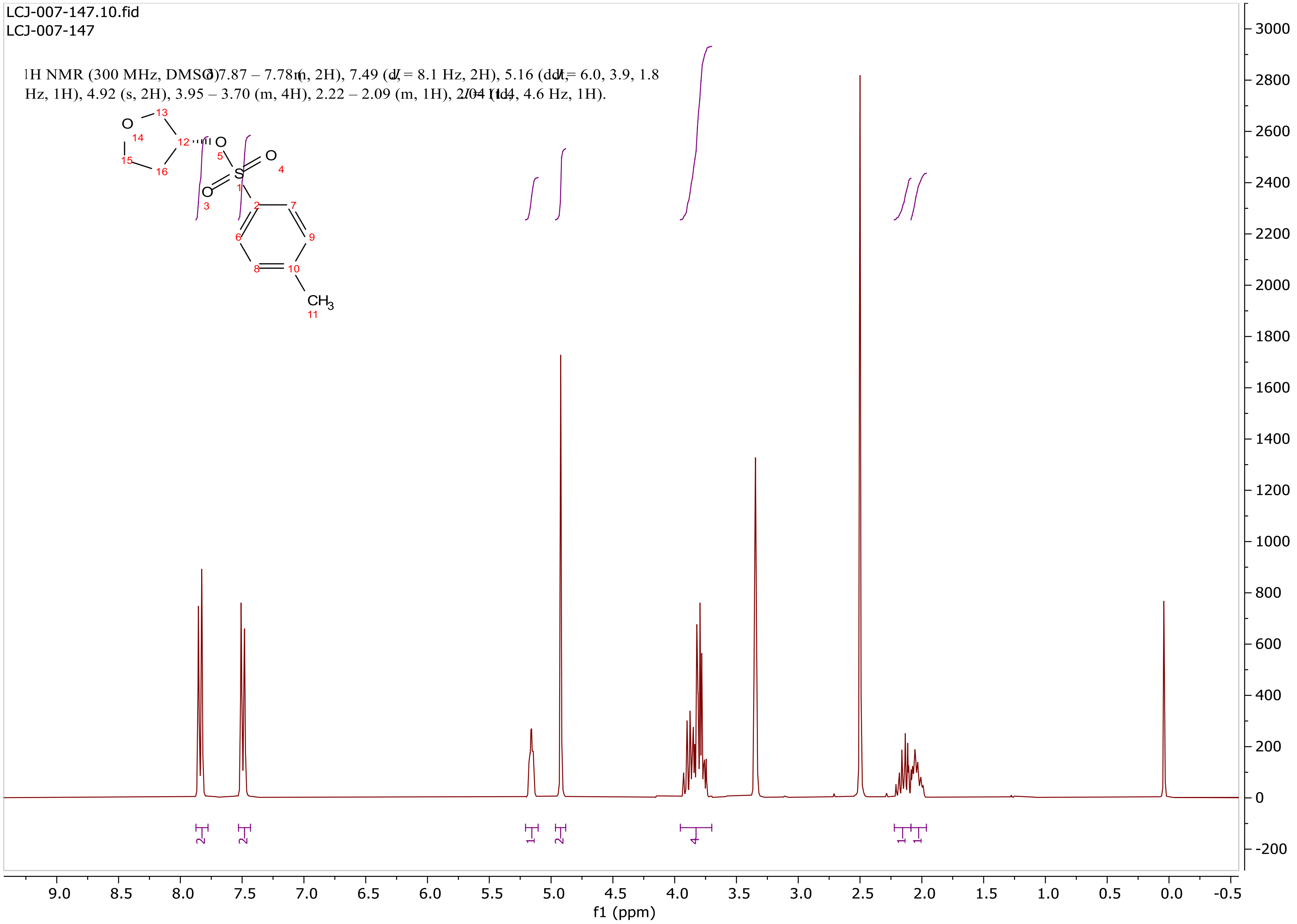
LCJ-007-147.10.fid
LCJ-007-147
1H NMR (300 MHz, DMSO) δ 7.87 – 7.78 (m, 2H), 7.49 (d, J = 8.1 Hz, 2H), 5.16 (ddt, J = 6.0, 3.9, 1.8 Hz, 1H), 4.92 (s, 2H), 3.95 – 3.70 (m, 4H), 2.22 – 2.09 (m, 1H), 2.04 (td, J = 11.4, 4.6 Hz, 1H).
CH3
f1 (ppm)

14j-^{1}H

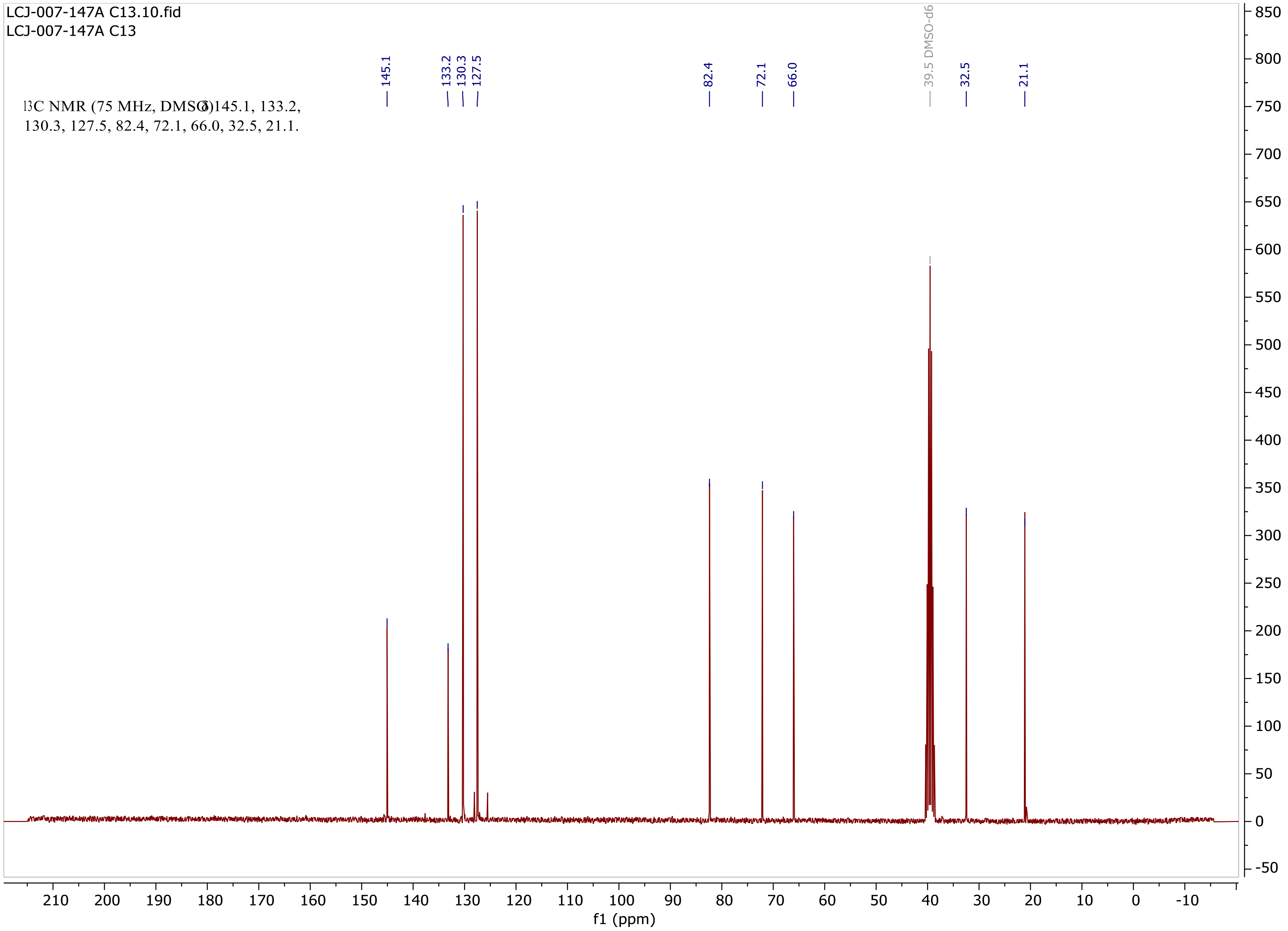
LCJ-007-147A C13.10.fid
LCJ-007-147A C13
13C NMR (75 MHz, DMSO) δ 145.1, 133.2, 130.3, 127.5, 82.4, 72.1, 66.0, 32.5, 21.1.
145.1
133.2
130.3
127.5
82.4
72.1
66.0
39.5 DMSO-d6
32.5
21.1
f1 (ppm)

14j-^{13}C

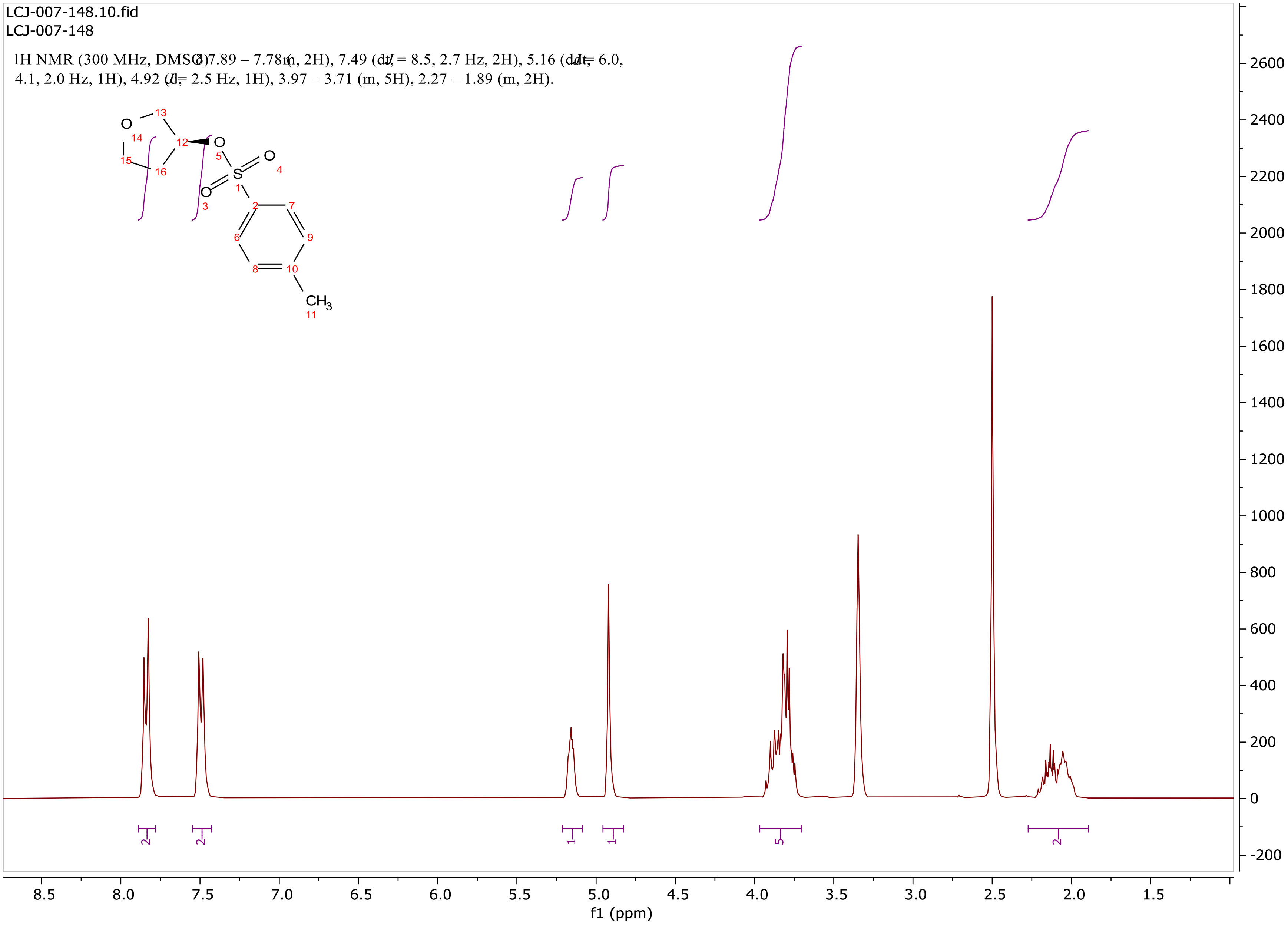
LCJ-007-148.10.fid
LCJ-007-148
1H NMR (300 MHz, DMSO) δ 7.89 – 7.78 (m, 2H), 7.49 (dt, J = 8.5, 2.7 Hz, 2H), 5.16 (ddt, J = 6.0, 4.1, 2.0 Hz, 1H), 4.92 (d, J = 2.5 Hz, 1H), 3.97 – 3.71 (m, 5H), 2.27 – 1.89 (m, 2H).
CH3
f1 (ppm)

14k-^{1}H

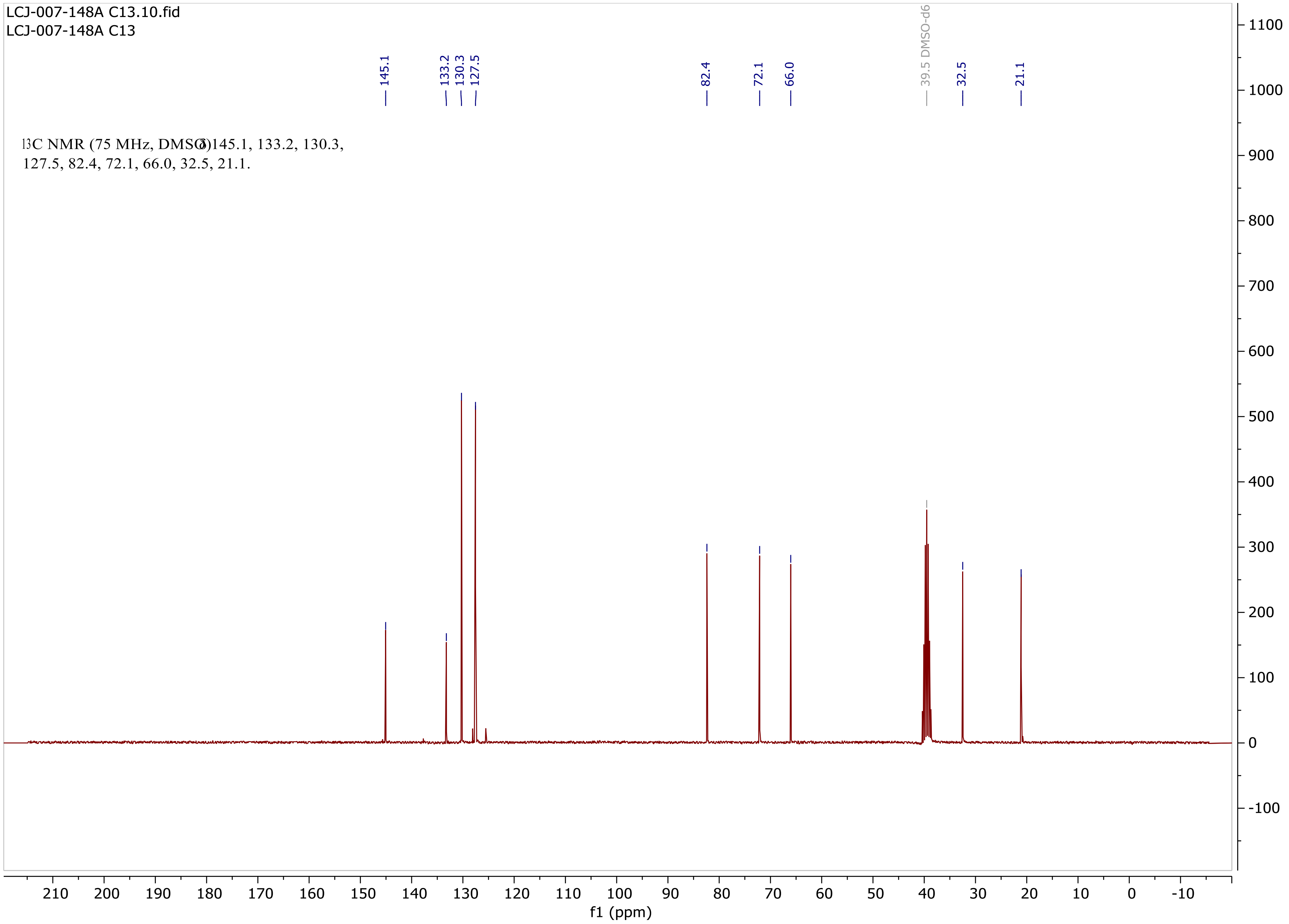
LCJ-007-148A C13.10.fid
LCJ-007-148A C13
145.1
133.2
130.3
127.5
82.4
72.1
66.0
39.5 DMSO-d6
32.5
21.1
13C NMR (75 MHz, DMSO) δ 145.1, 133.2, 130.3, 127.5, 82.4, 72.1, 66.0, 32.5, 21.1.
f1 (ppm)

14k-^{13}C

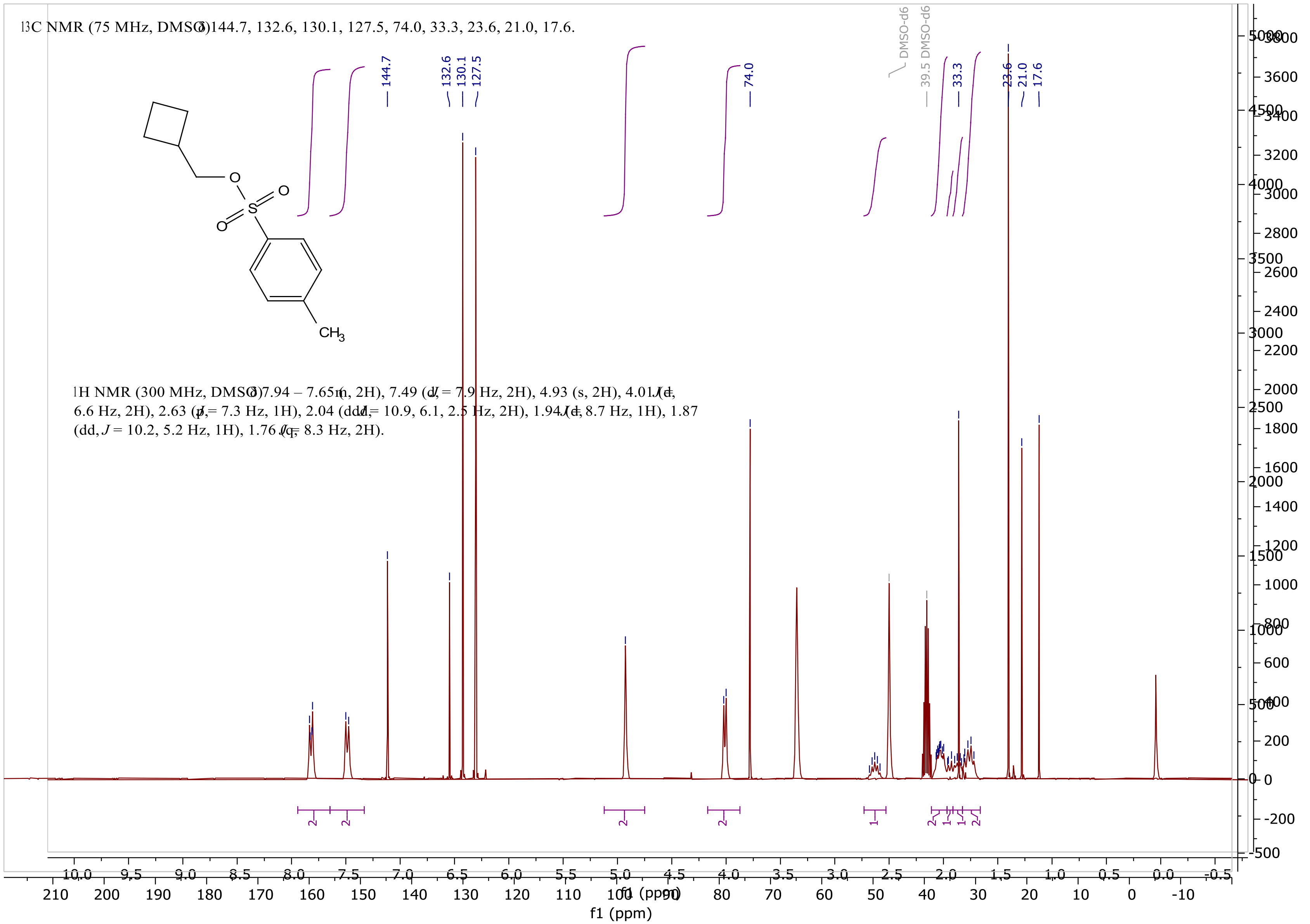

14l-^{1}H

14l-^{13}C

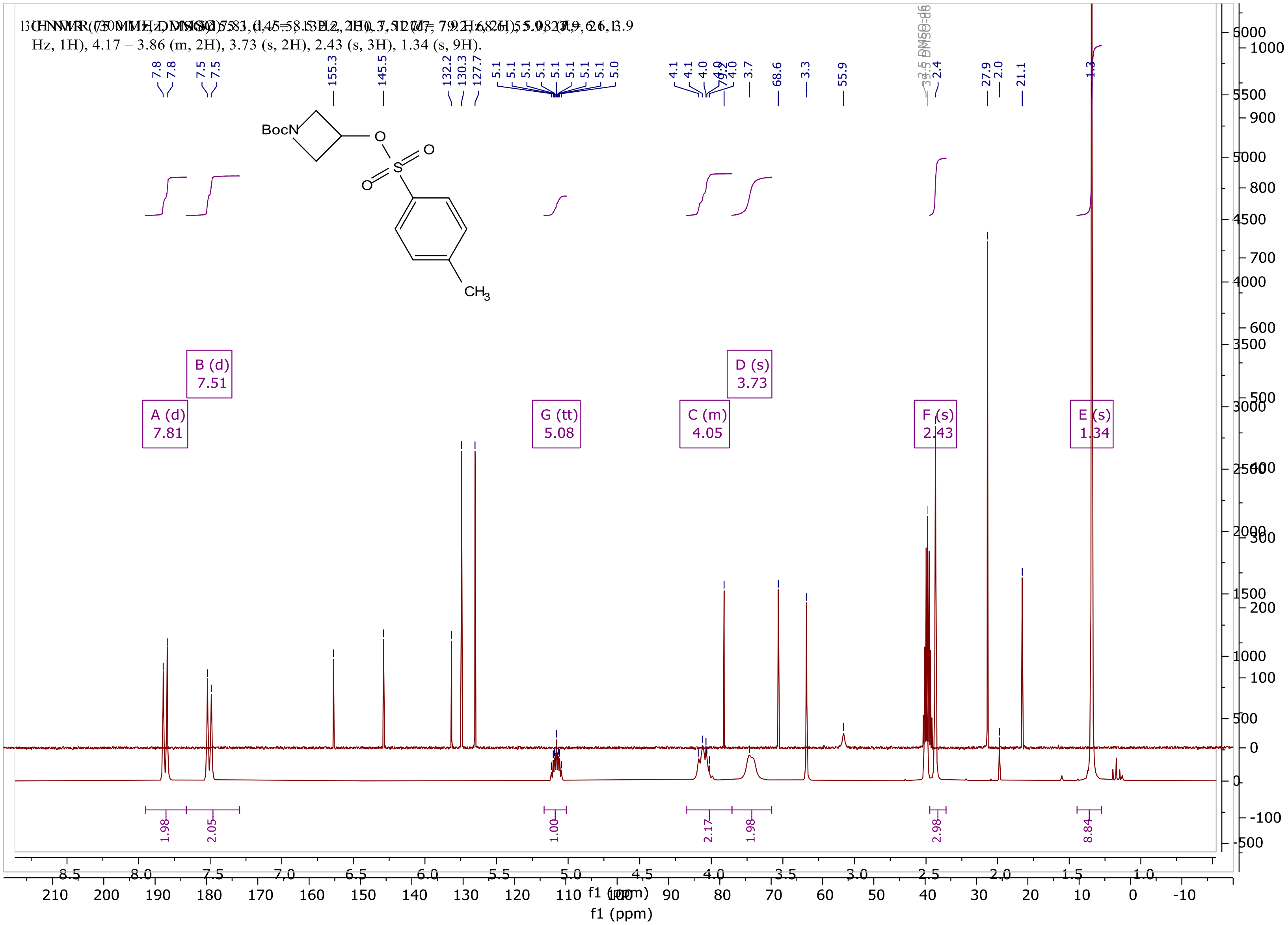

14m-^{1}H

14m-^{13}C

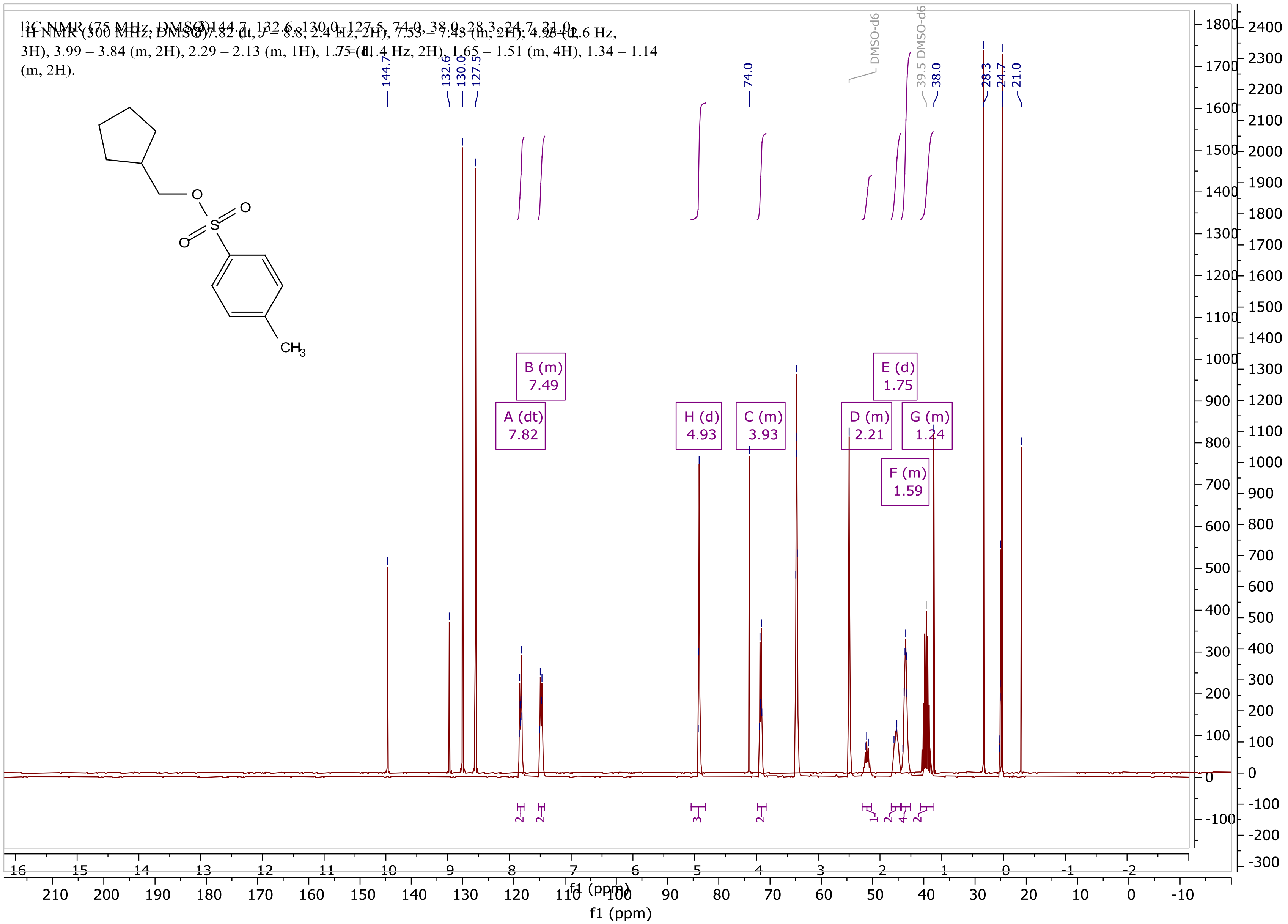

15n-^{1}H

15n-^{13}C

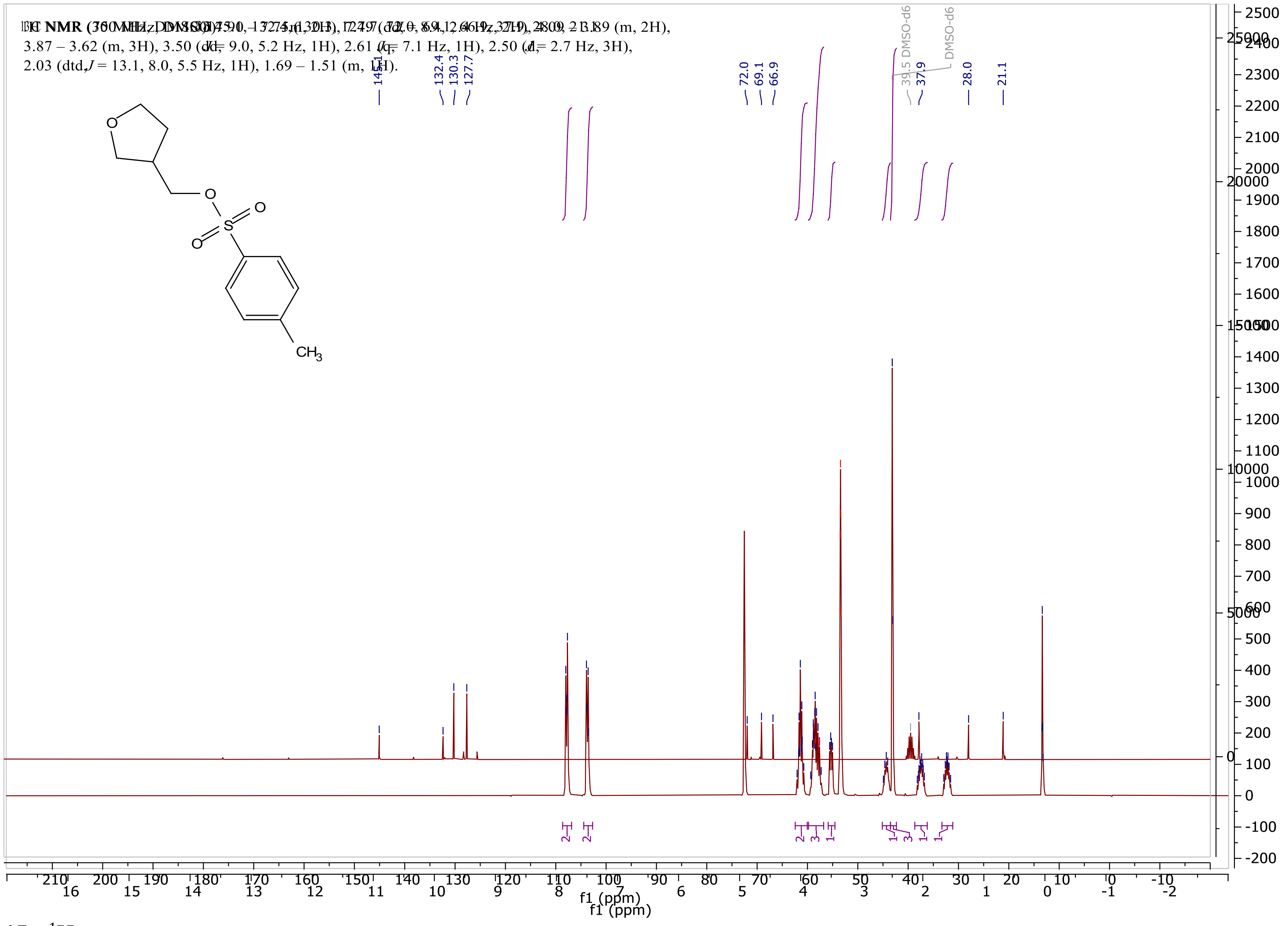

15o-^{1}H

15o-^{13}C

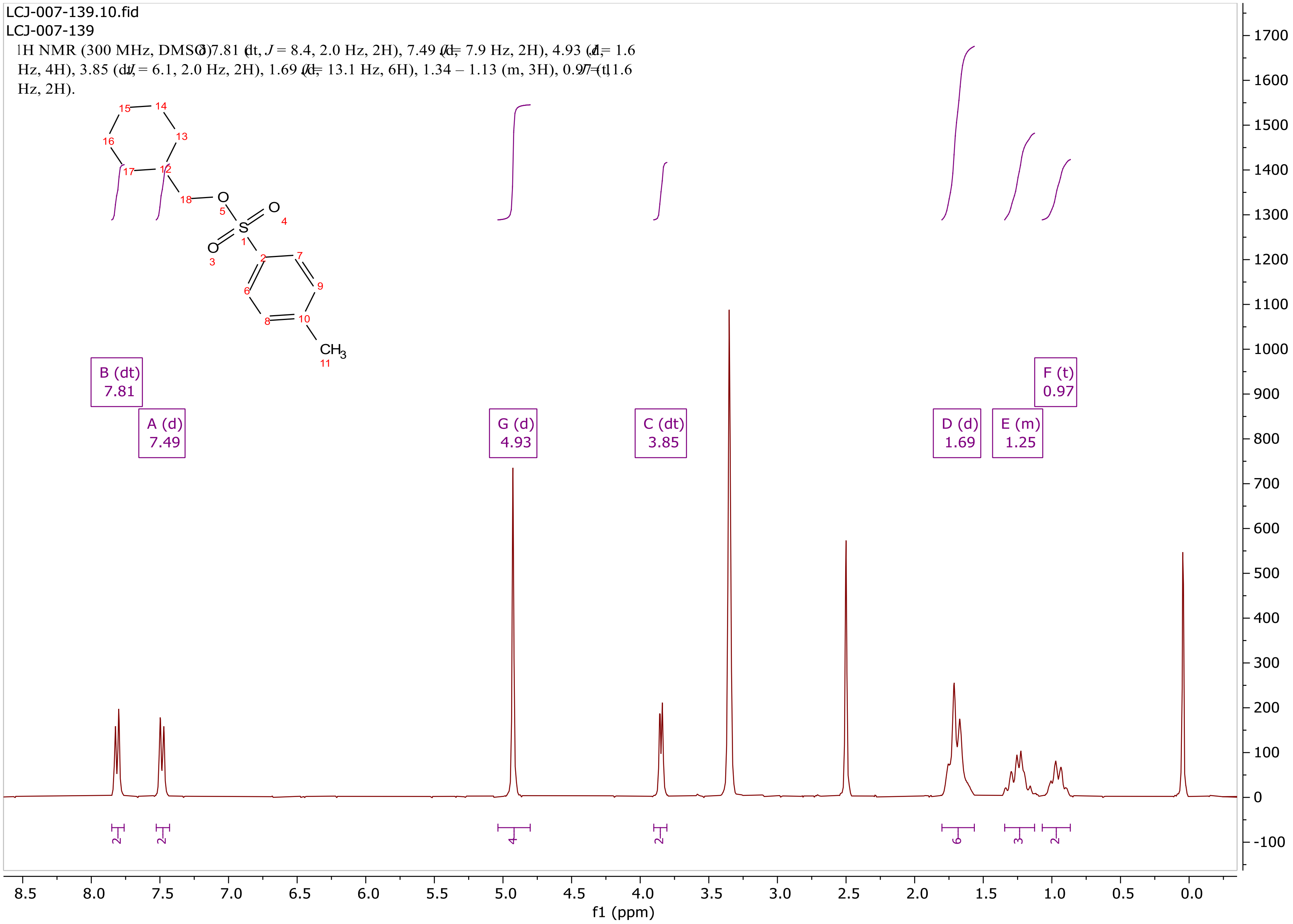
LCJ-007-139.10.fid
LCJ-007-139
1H NMR (300 MHz, DMSO) δ 7.81 (dt, J = 8.4, 2.0 Hz, 2H), 7.49 (d, J = 7.9 Hz, 2H), 4.93 (d, J = 1.6 Hz, 4H), 3.85 (dt, J = 6.1, 2.0 Hz, 2H), 1.69 (d, J = 13.1 Hz, 6H), 1.34 – 1.13 (m, 3H), 0.97 (t, J = 1.6 Hz, 2H).
CH3
B (dt)
7.81
A (d)
7.49
G (d)
4.93
C (dt)
3.85
D (d)
1.69
E (m)
1.25
F (t)
0.97
f1 (ppm)

14p-^{1}H

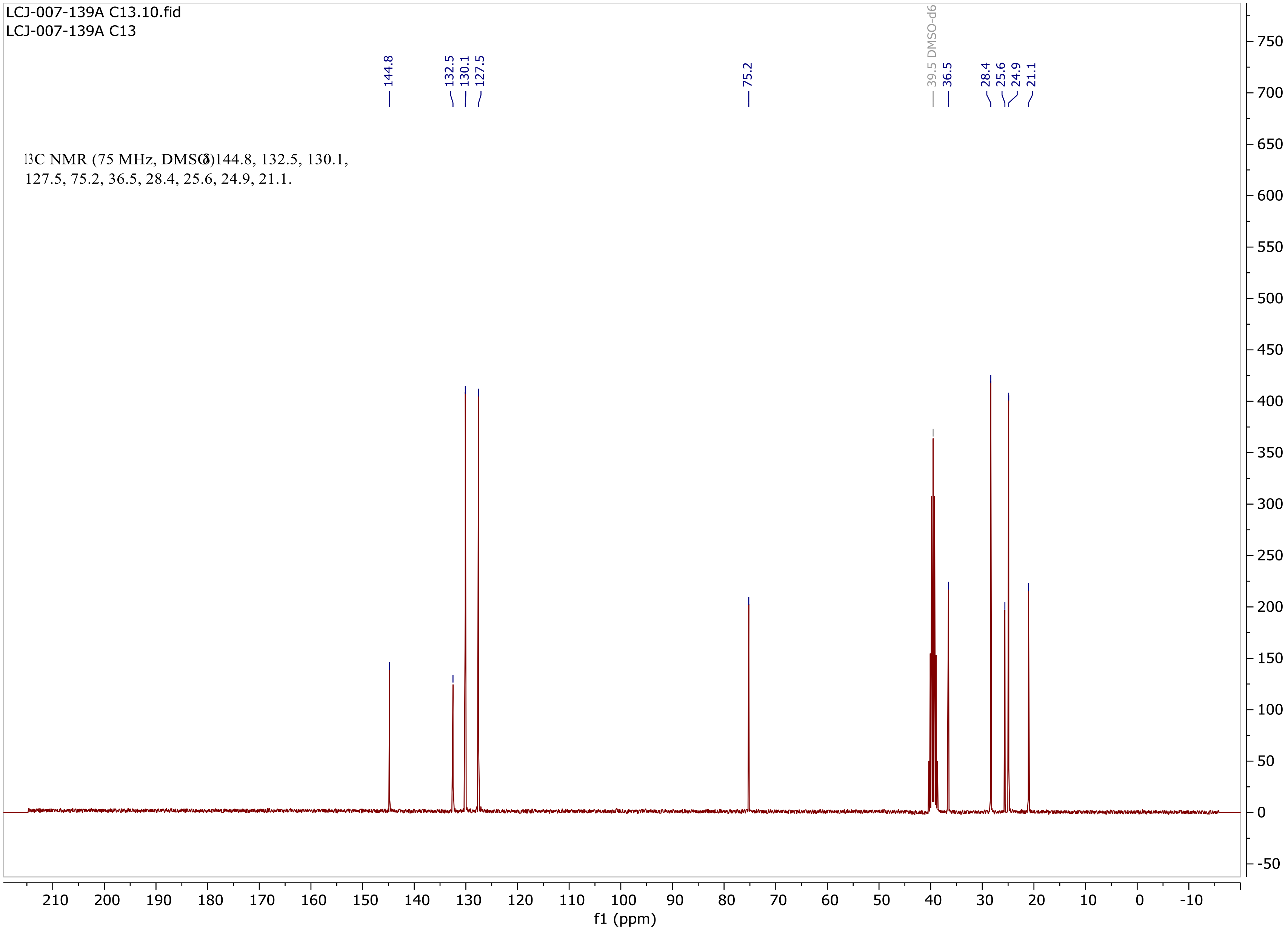
LCJ-007-139A C13.10.fid
LCJ-007-139A C13
144.8
132.5
130.1
127.5
75.2
39.5 DMSO-d6
36.5
28.4
25.6
24.9
21.1
13C NMR (75 MHz, DMSO) δ 144.8, 132.5, 130.1,
127.5, 75.2, 36.5, 28.4, 25.6, 24.9, 21.1.
f1 (ppm)

14p-^{13}C

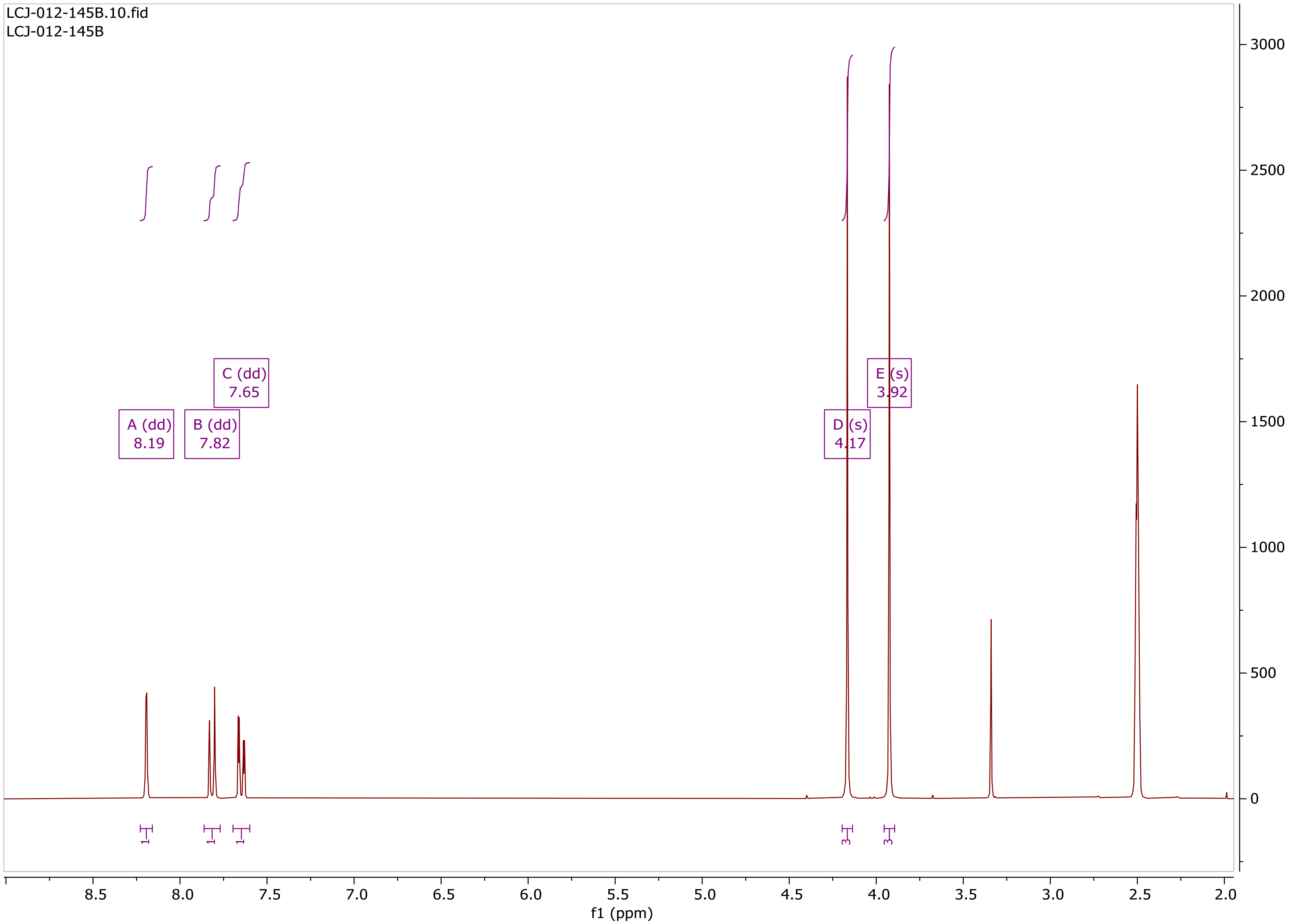

LCJ-012-145B.10.fid
LCJ-012-145B
A (dd) 8.19
B (dd) 7.82
C (dd) 7.65
D (s) 4.17
E (s) 3.92
1
1
1
3
3
3000
2500
2000
1500
1000
500
0
8.5
8.0
7.5
7.0
6.5
6.0
5.5
5.0
4.5
4.0
3.5
3.0
2.5
2.0
f1 (ppm)

15a-^{1}H

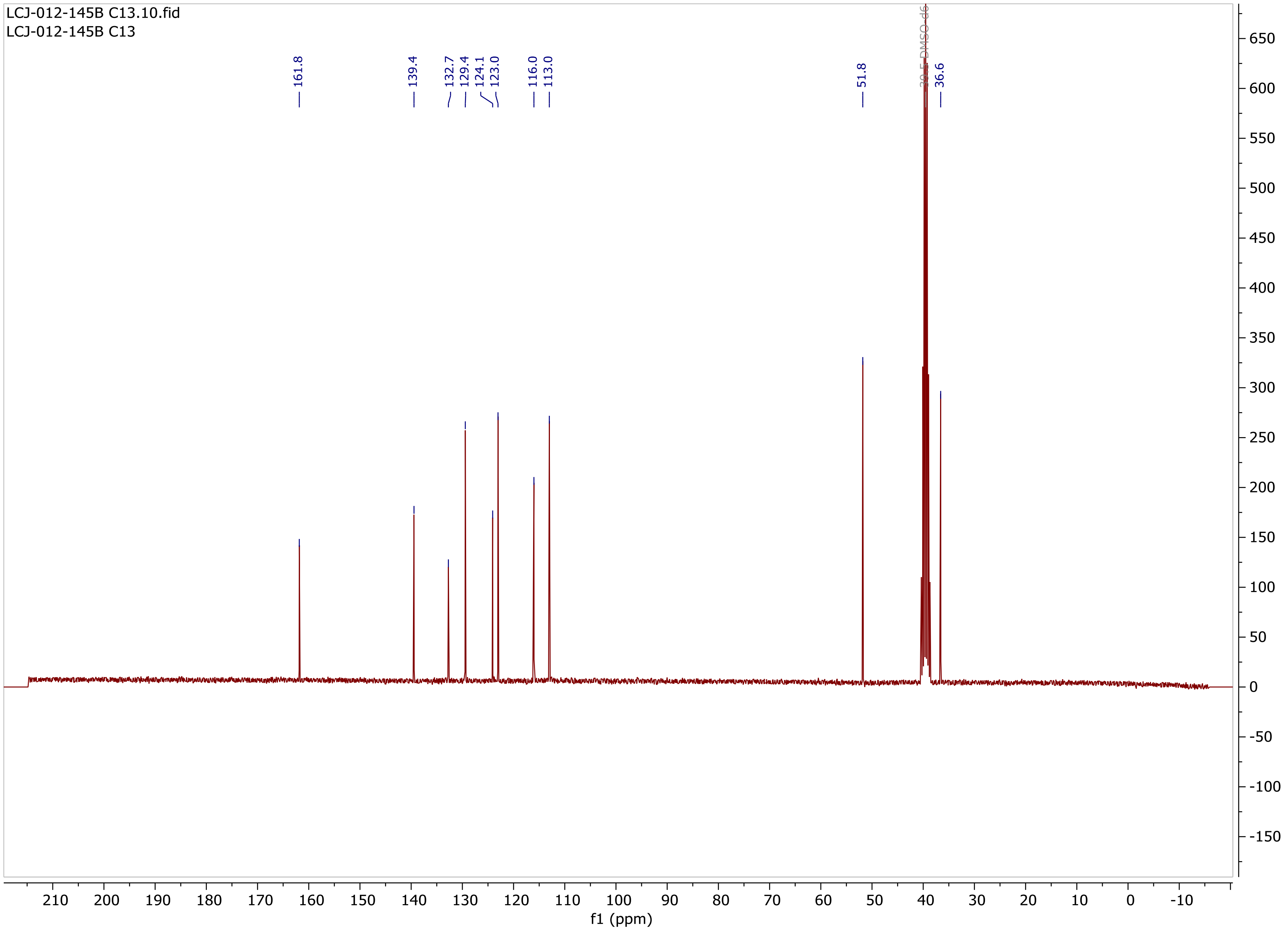

LCJ-012-145B C13.10.fid
LCJ-012-145B C13
161.8
139.4
132.7
129.4
124.1
123.0
116.0
113.0
51.8
39.5 DMSO-d6
36.6
f1 (ppm)

15a-^{13}C

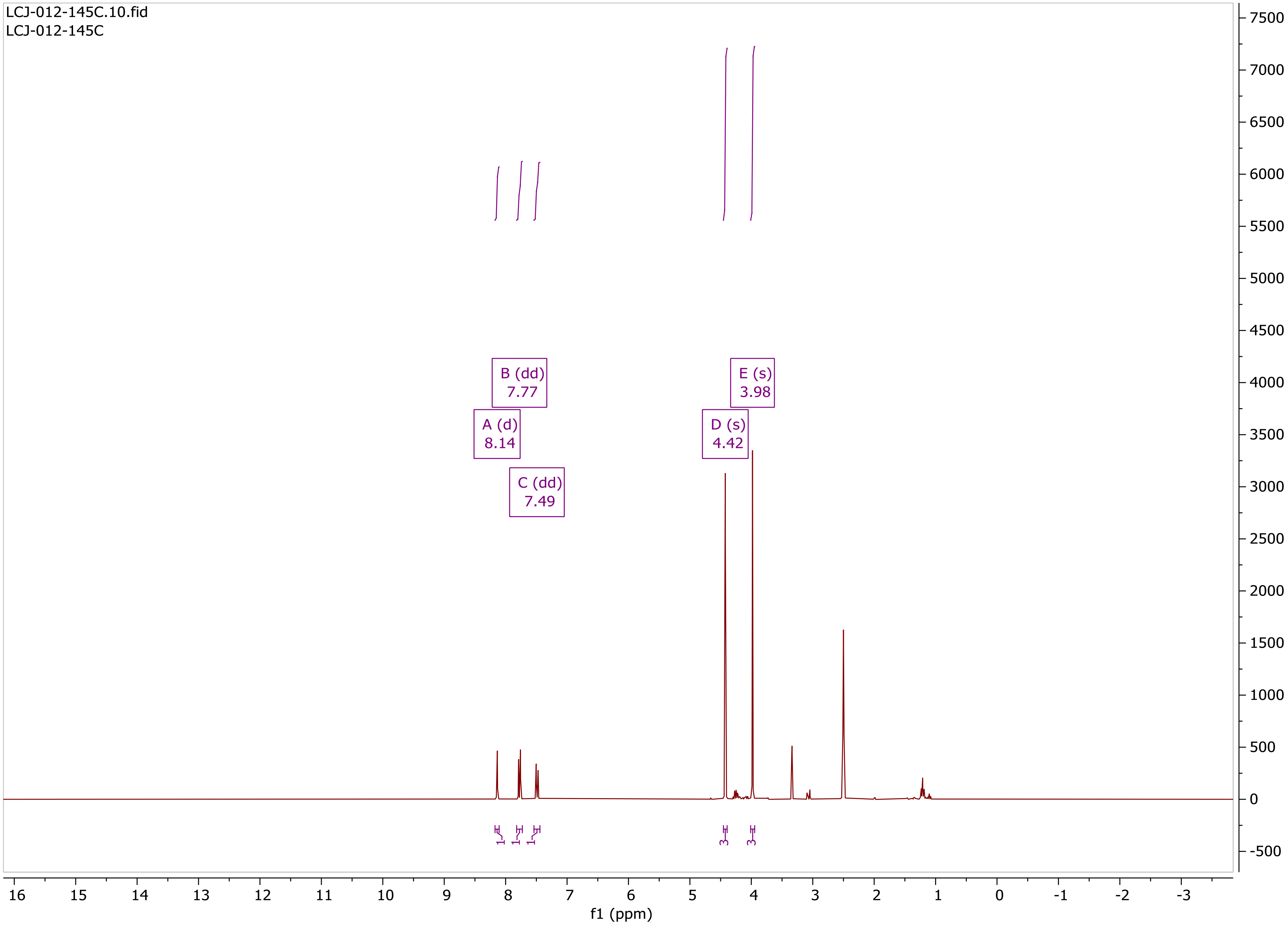
LCJ-012-145C.10.fid
LCJ-012-145C
B (dd) 7.77
E (s) 3.98
A (d) 8.14
D (s) 4.42
C (dd) 7.49
f1 (ppm)

16a-^{1}H

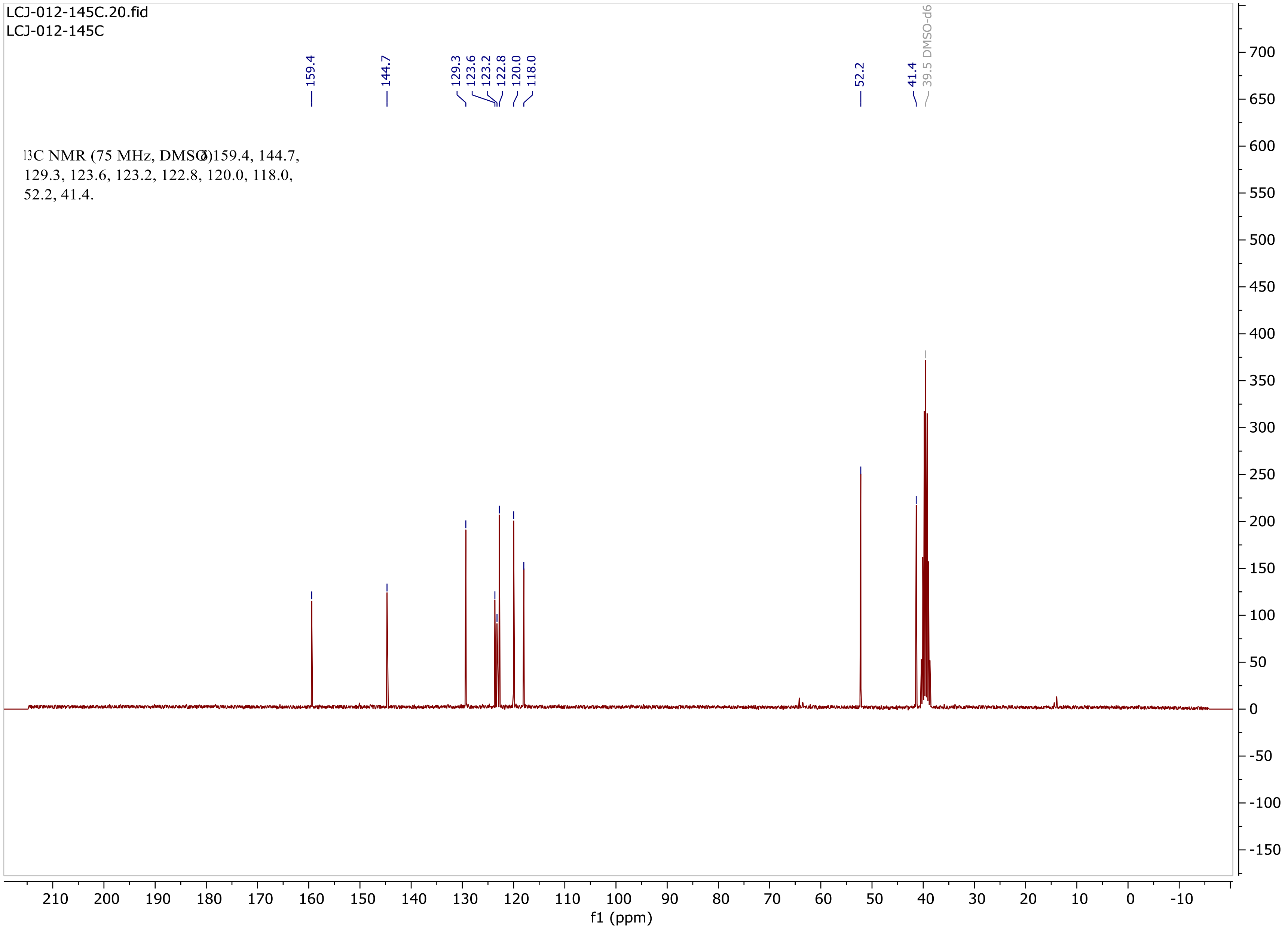
LCJ-012-145C.20.fid
LCJ-012-145C
13C NMR (75 MHz, DMSO) δ 159.4, 144.7, 129.3, 123.6, 123.2, 122.8, 120.0, 118.0, 52.2, 41.4.
159.4
144.7
129.3
123.6
123.2
122.8
120.0
118.0
52.2
41.4
39.5 DMSO-d6
f1 (ppm)

16a-^{13}C

LCJ-006-025 A2 (N1).10.fid
LCJ-006-025 A2 (N1)

1H NMR (300 MHz, DMSO-d_6) δ 8.21 (d, J = 1.8 Hz, 1H), 7.87 (d, J = 8.9 Hz, 1H), 7.64 (dd, J = 8.9, 1.9 Hz, 1H), 4.56 (q, J = 7.2 Hz, 2H), 3.93 (s, 3H), 1.43 (t, J = 7.2 Hz, 3H).

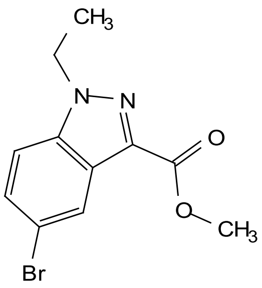

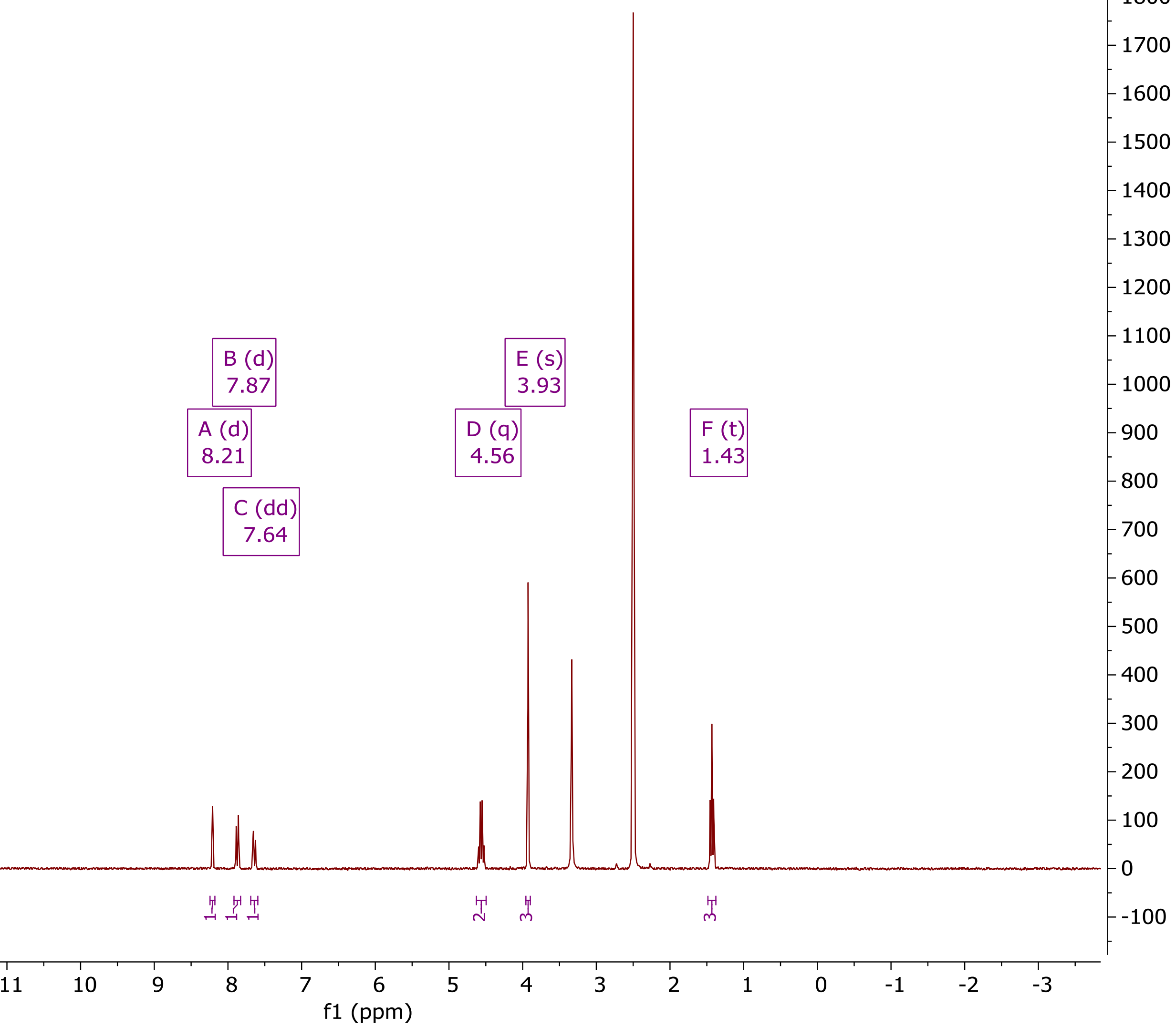

15b-^{1}H

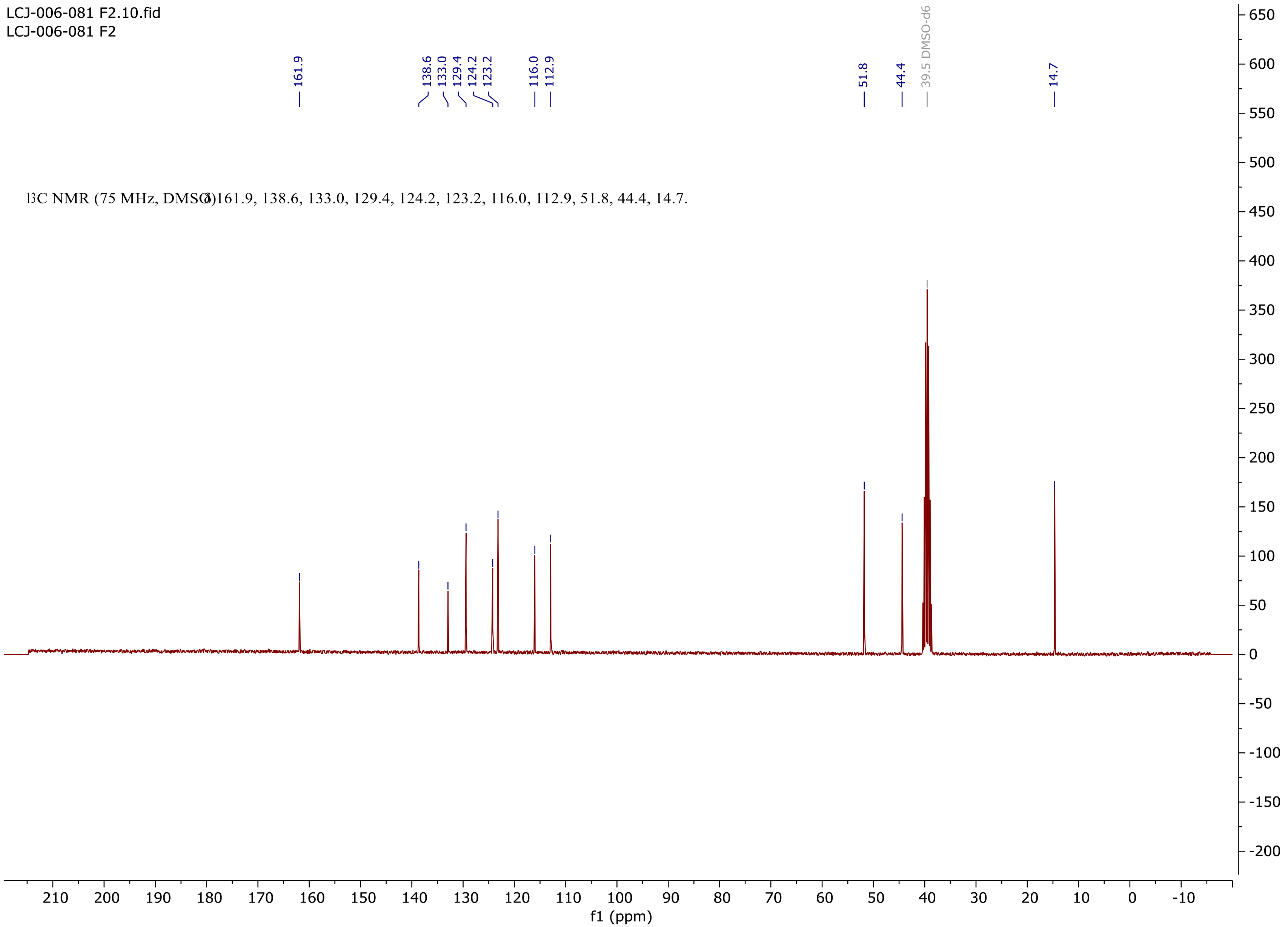

13C NMR (75 MHz, DMSO) δ 161.9, 138.6, 133.0, 129.4, 124.2, 123.2, 116.0, 112.9, 51.8, 44.4, 14.7.

15b-^{13}C

LCJ-006-081 F2C.10.fid
LCJ-006-081 F2C

1H NMR (300 MHz, DMSO-d_6) δ 8.16 (dd, J = 1.9, 0.8 Hz, 1H), 7.80 (dd, J = 9.1, 0.8 Hz, 1H), 7.50 (dd, J = 9.1, 1.9 Hz, 1H), 4.86 (q, J = 7.2 Hz, 2H), 4.00 (s, 3H), 1.48 (t, J = 7.2 Hz, 3H).

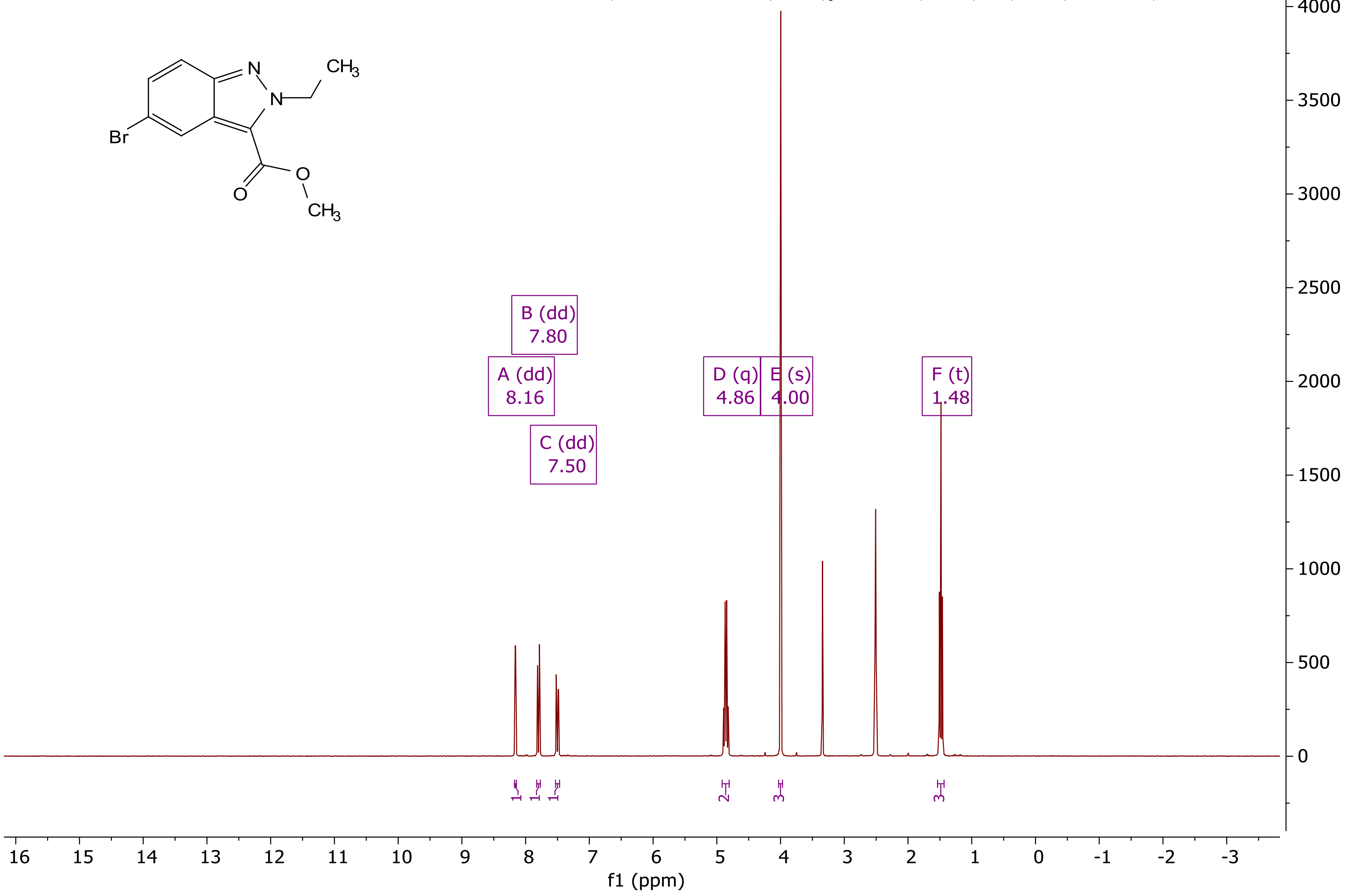

16b-^{1}H

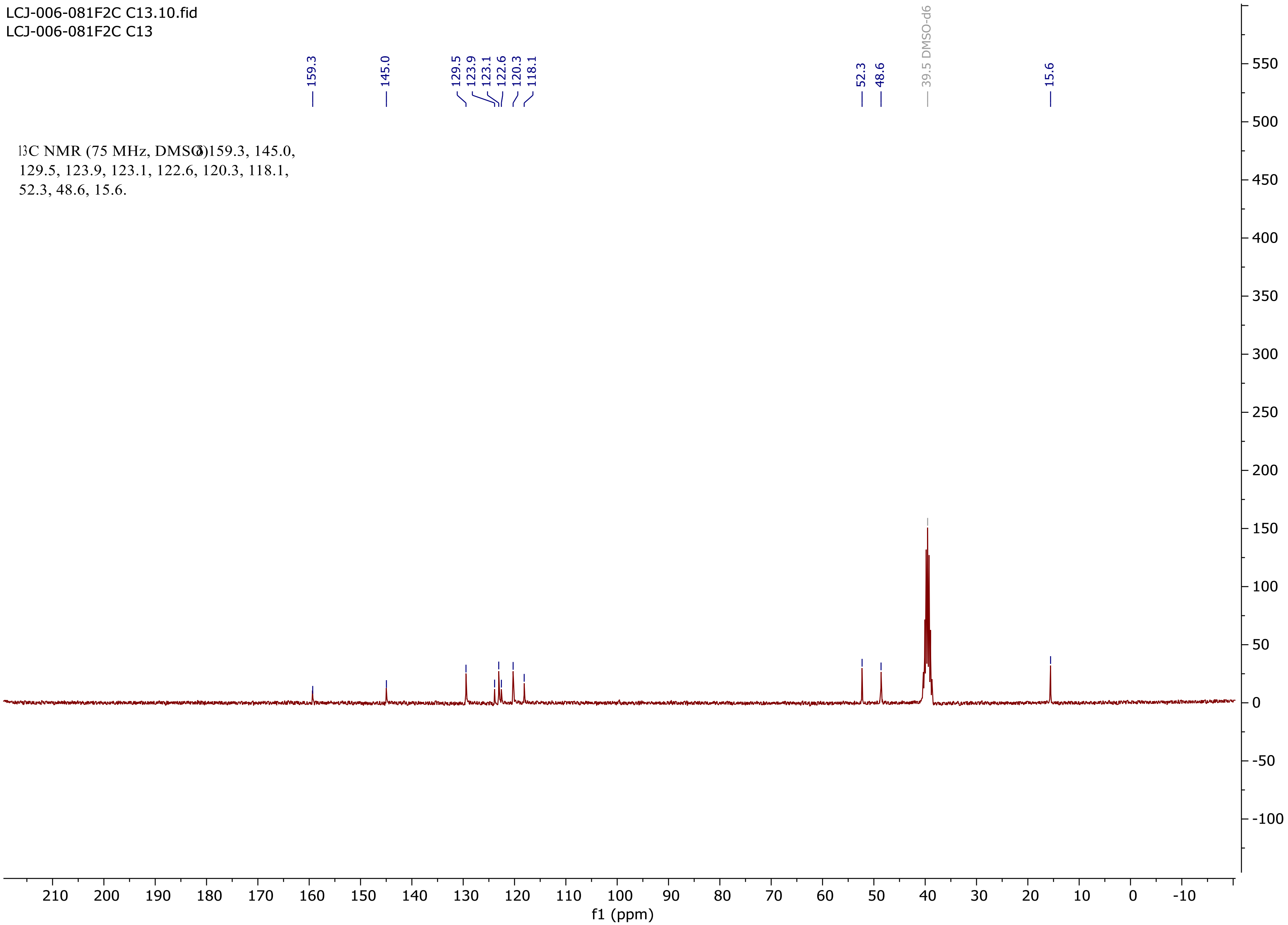

^{13}C NMR (75 MHz, DMSO[illegible]) 159.3, 145.0, 129.5, 123.9, 123.1, 122.6, 120.3, 118.1, 52.3, 48.6, 15.6.

16b-^{13}C

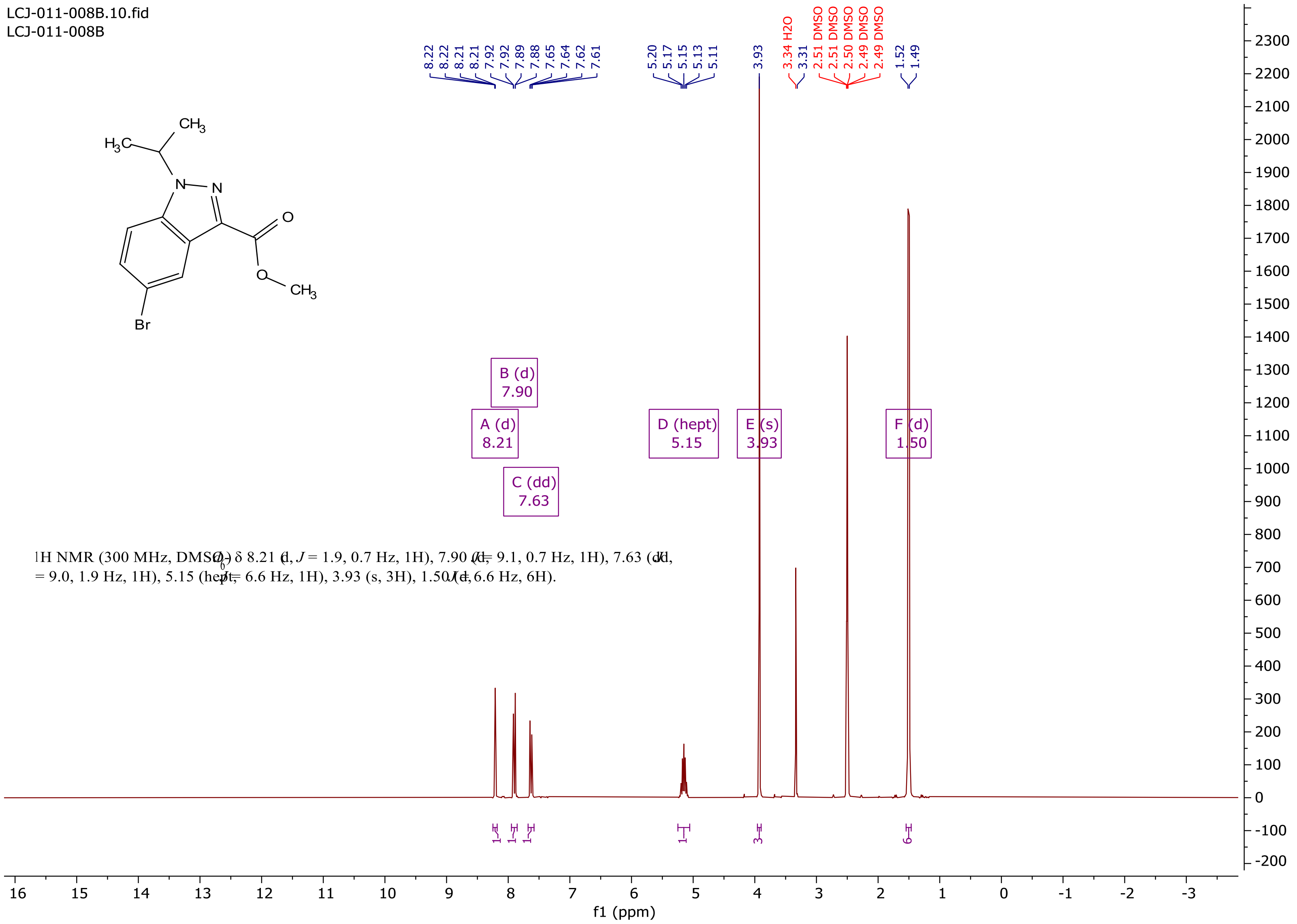

1H NMR (300 MHz, DMSO-d_6) δ 8.21 (d, J = 1.9, 0.7 Hz, 1H), 7.90 (d, J = 9.1, 0.7 Hz, 1H), 7.63 (dd, J = 9.0, 1.9 Hz, 1H), 5.15 (hept, J = 6.6 Hz, 1H), 3.93 (s, 3H), 1.50 (d, J = 6.6 Hz, 6H).

15c-^{1}H

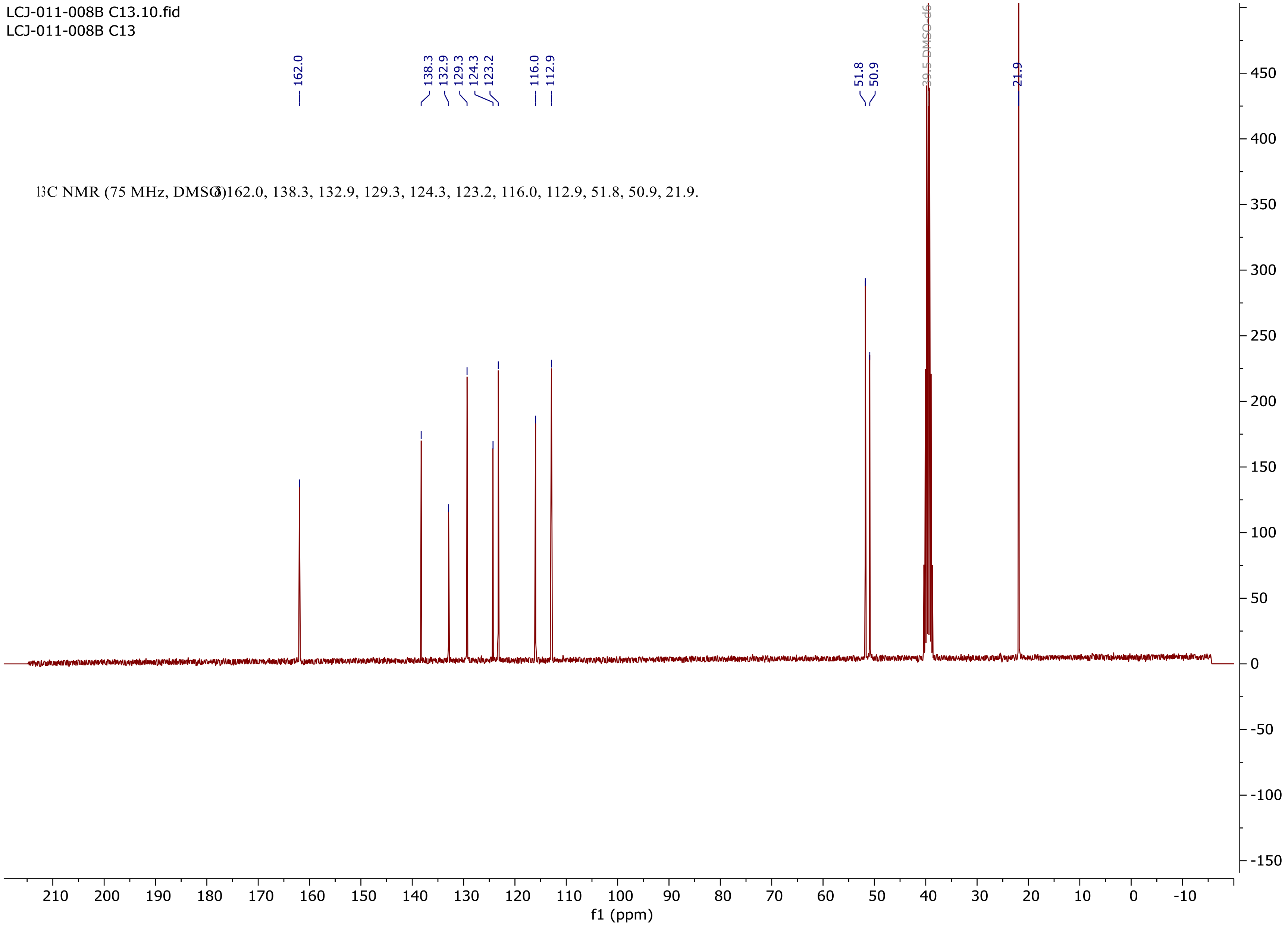

^{13}C NMR (75 MHz, DMSO) δ 162.0, 138.3, 132.9, 129.3, 124.3, 123.2, 116.0, 112.9, 51.8, 50.9, 21.9.

15c-^{13}C

LCJ-011-008C.10.fid
LCJ-011-008C

1H NMR (300 MHz, DMSO-d_6) δ 8.17 (d, *J* = 1.9 Hz, 1H), 7.82 (d, *J* = 9.0 Hz, 1H), 7.49 (dd, *J* = 9.1, 1.9 Hz, 1H), 5.84 (hept, *J* = 6.6 Hz, 1H), 3.99 (s, 3H), 1.55 (d, *J* = 6.6 Hz, 6H).

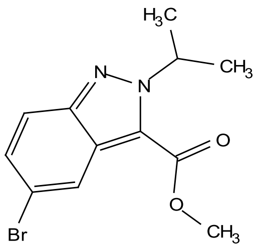

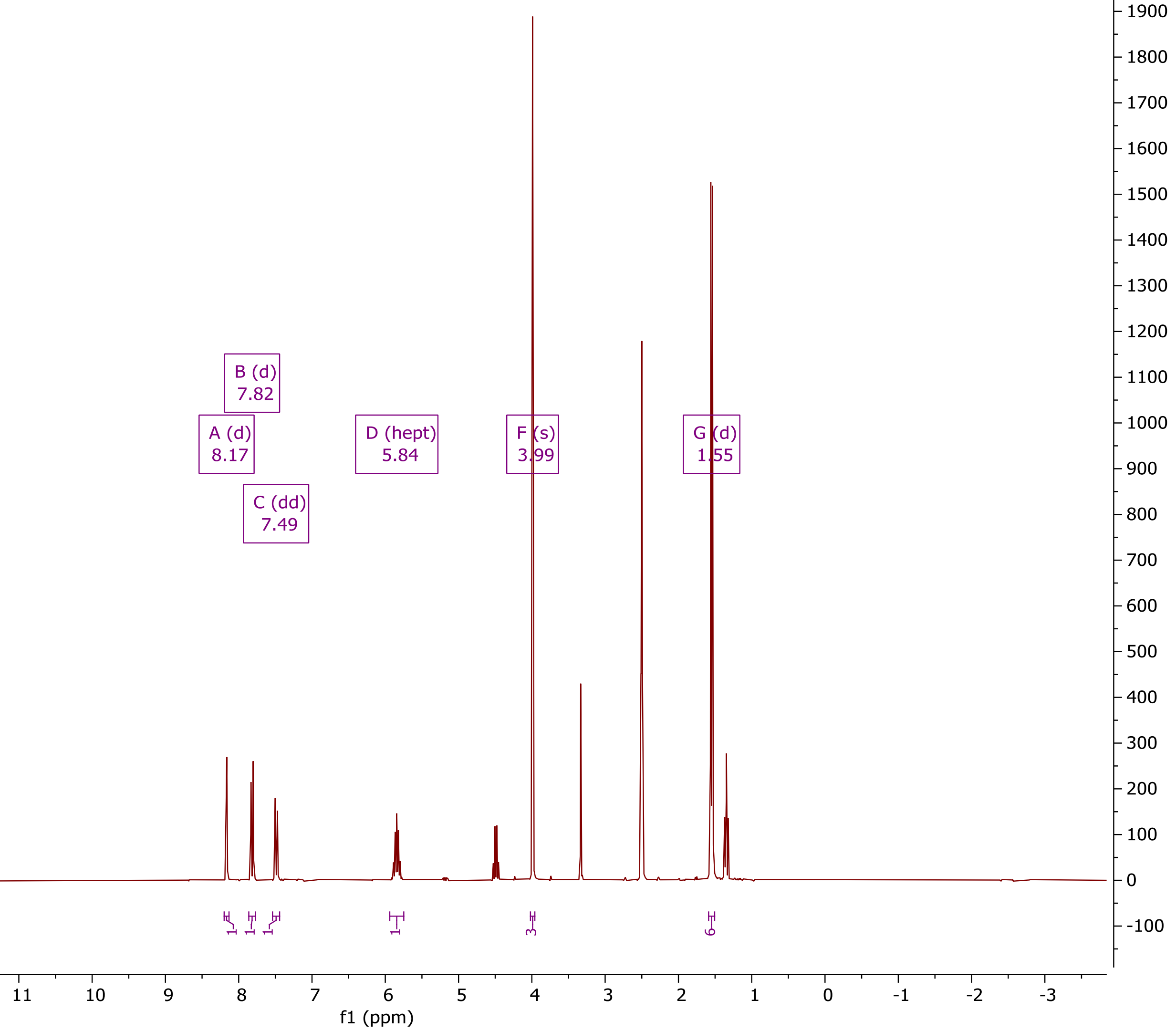

16c-^{1}H

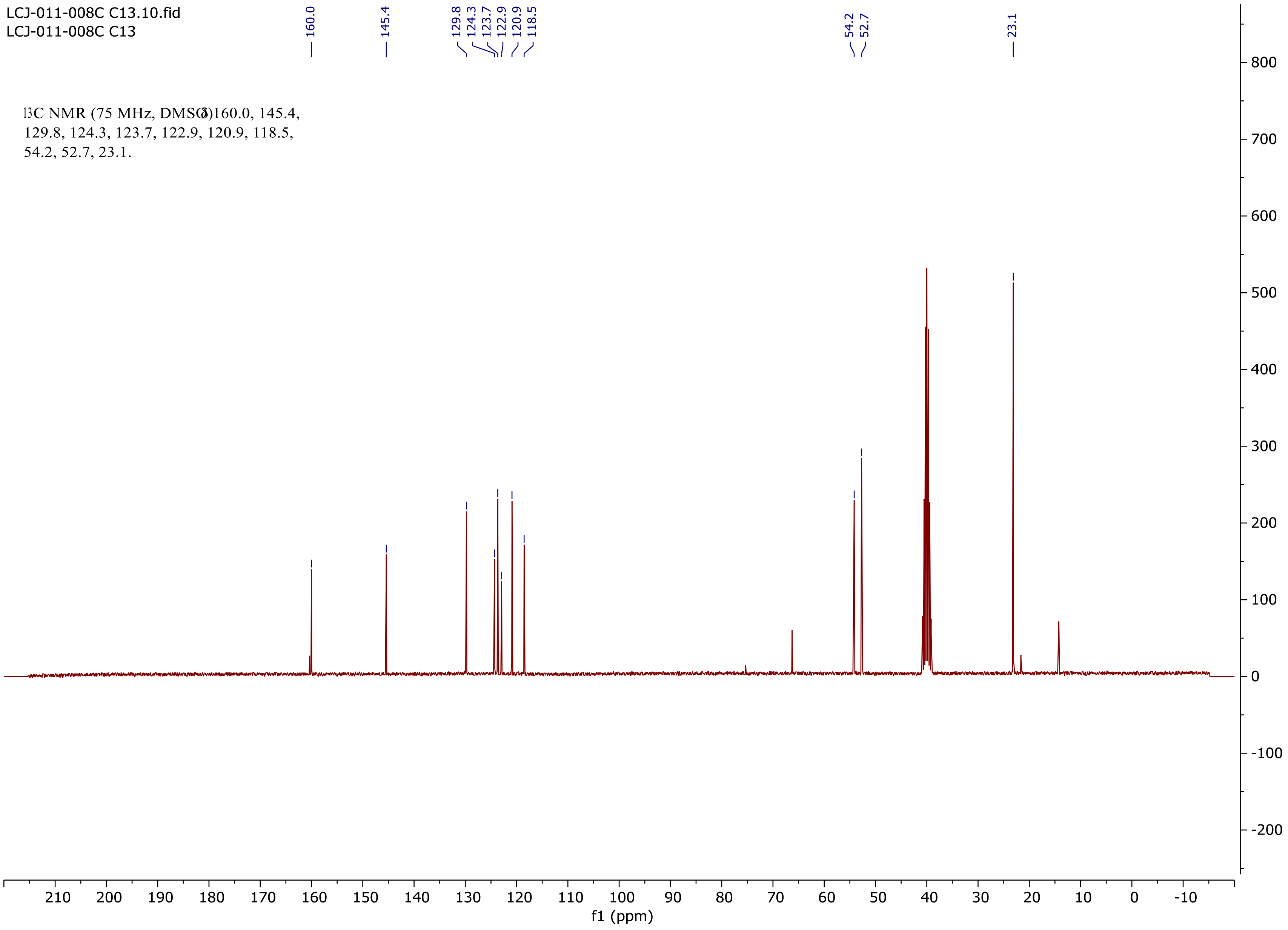
LCJ-011-008C C13.10.fid
LCJ-011-008C C13
160.0
145.4
129.8
124.3
123.7
122.9
120.9
118.5
54.2
52.7
23.1
13C NMR (75 MHz, DMSO) δ 160.0, 145.4, 129.8, 124.3, 123.7, 122.9, 120.9, 118.5, 54.2, 52.7, 23.1.
f1 (ppm)

16c-^{13}C

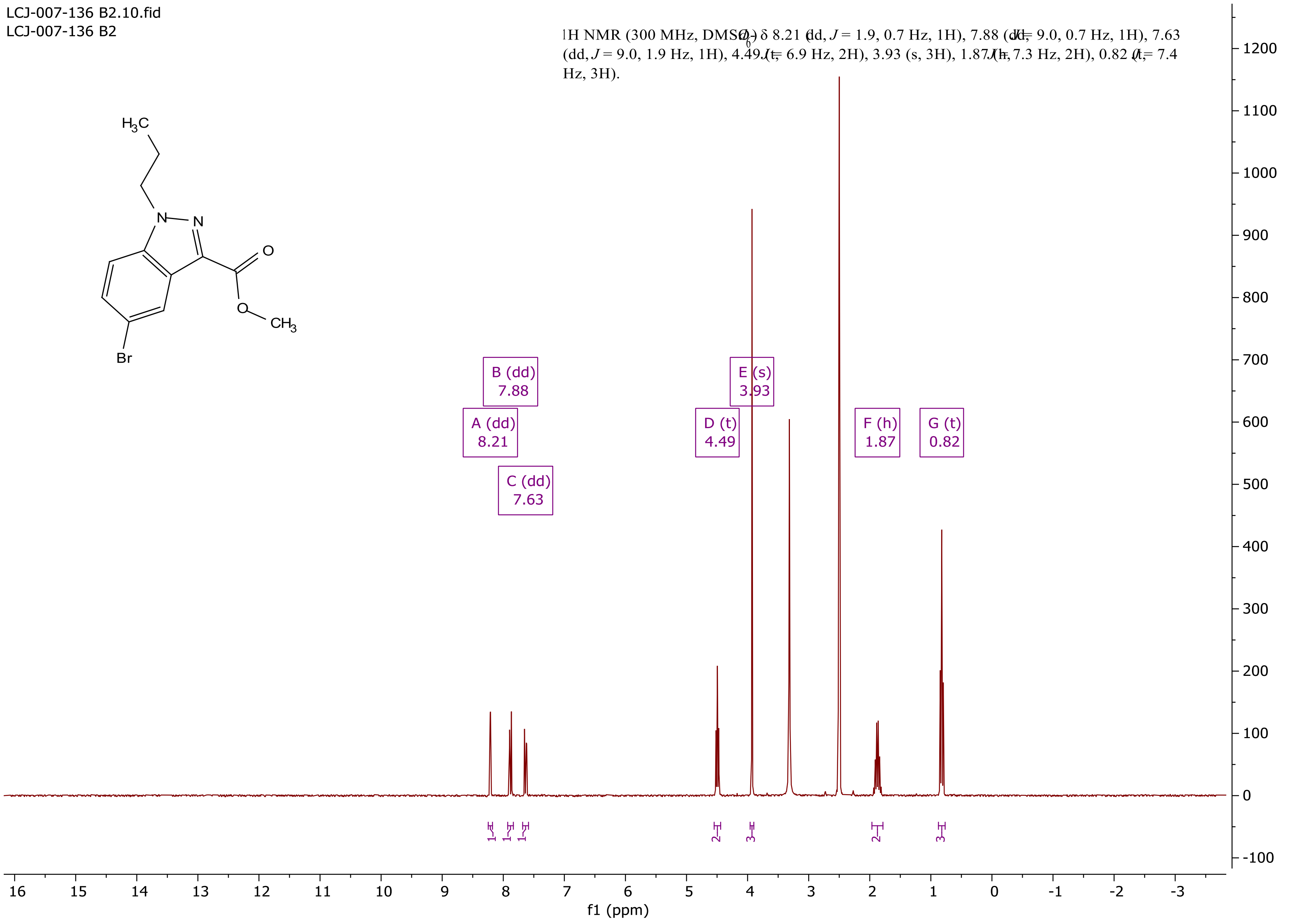
LCJ-007-136 B2.10.fid
LCJ-007-136 B2
1H NMR (300 MHz, DMSO-d6) δ 8.21 (dd, J = 1.9, 0.7 Hz, 1H), 7.88 (dd, J = 9.0, 0.7 Hz, 1H), 7.63 (dd, J = 9.0, 1.9 Hz, 1H), 4.49 (t, J = 6.9 Hz, 2H), 3.93 (s, 3H), 1.87 (h, J = 7.3 Hz, 2H), 0.82 (t, J = 7.4 Hz, 3H).
H3C
N
N
O
O
CH3
Br
B (dd) 7.88
A (dd) 8.21
C (dd) 7.63
D (t) 4.49
E (s) 3.93
F (h) 1.87
G (t) 0.82
f1 (ppm)

15d-^{1}H

LCJ-007-136B C13.10.fid
LCJ-007-136B C13

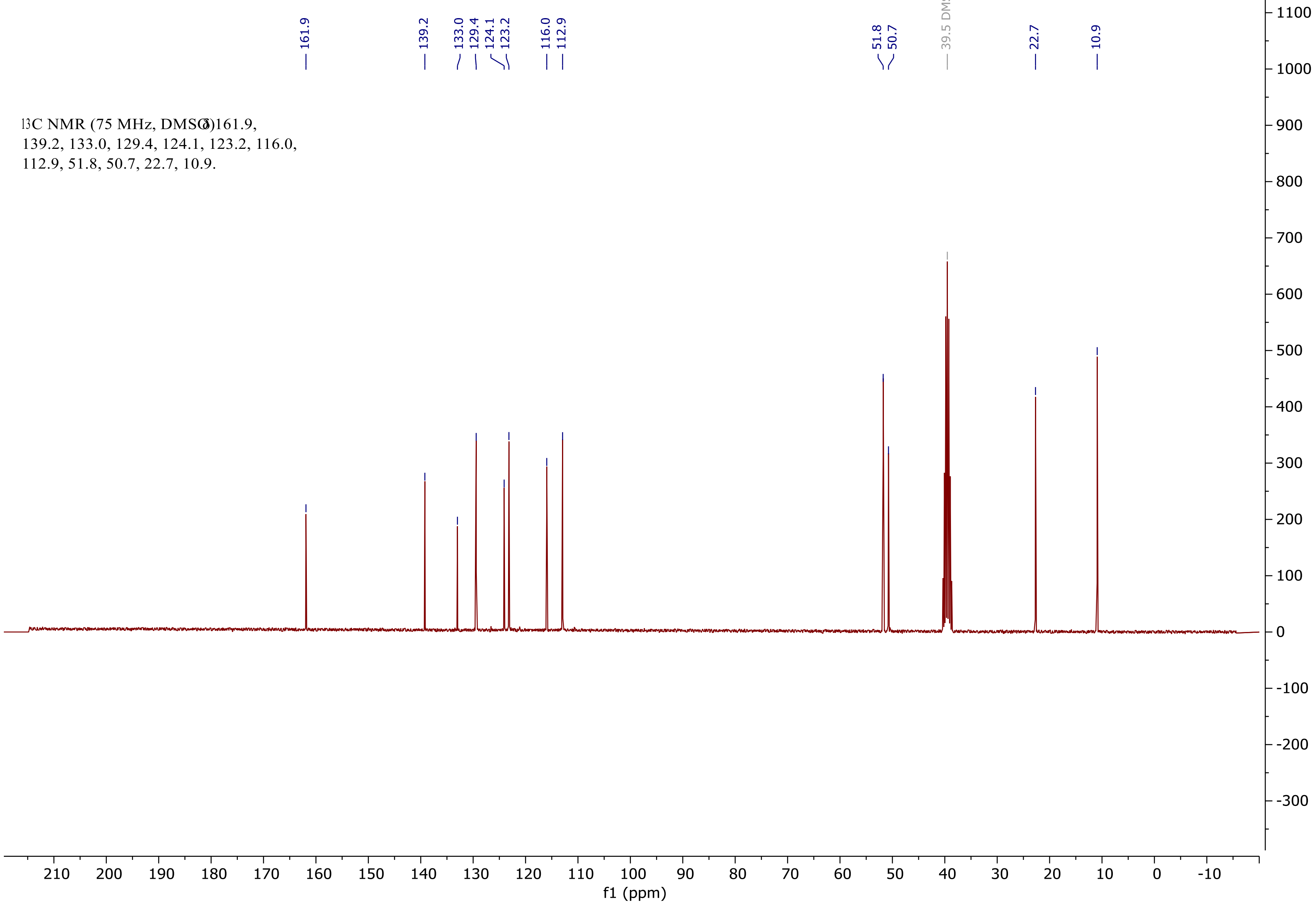

^{13}C NMR (75 MHz, DMSO δ) 161.9, 139.2, 133.0, 129.4, 124.1, 123.2, 116.0, 112.9, 51.8, 50.7, 22.7, 10.9.

15d-^{13}C

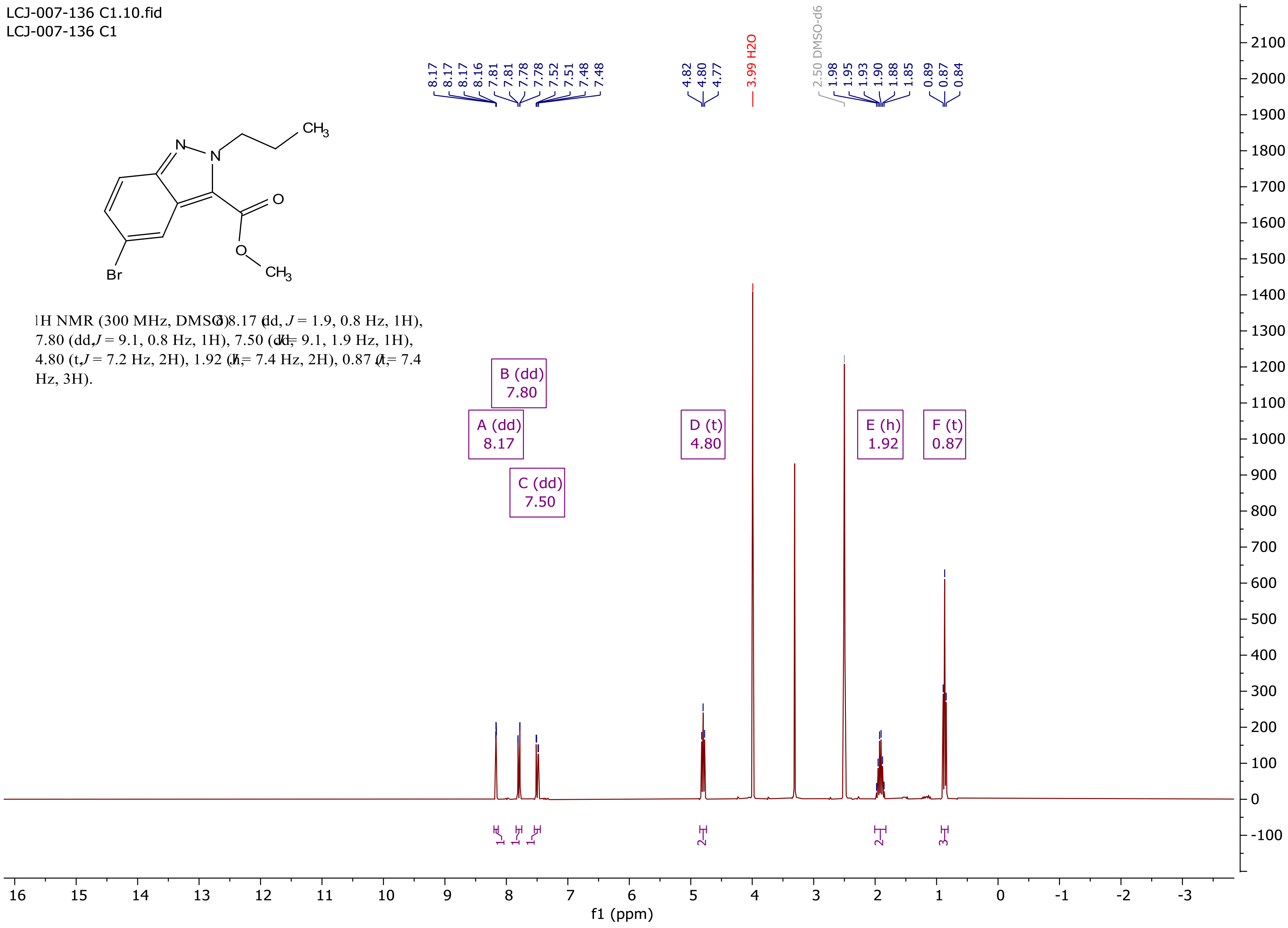

1H NMR (300 MHz, DMSO) δ 8.17 (dd, J = 1.9, 0.8 Hz, 1H), 7.80 (dd, J = 9.1, 0.8 Hz, 1H), 7.50 (dd, J = 9.1, 1.9 Hz, 1H), 4.80 (t, J = 7.2 Hz, 2H), 1.92 (h, J = 7.4 Hz, 2H), 0.87 (t, J = 7.4 Hz, 3H).

16d-^{1}H

LCJ-007-136C C13.10.fid
LCJ-007-136C C13

13C NMR (75 MHz, DMSO) δ 159.4, 144.9, 129.5, 123.8, 123.1, 122.9, 120.3, 118.1, 54.6, 52.3, 23.5, 10.8.

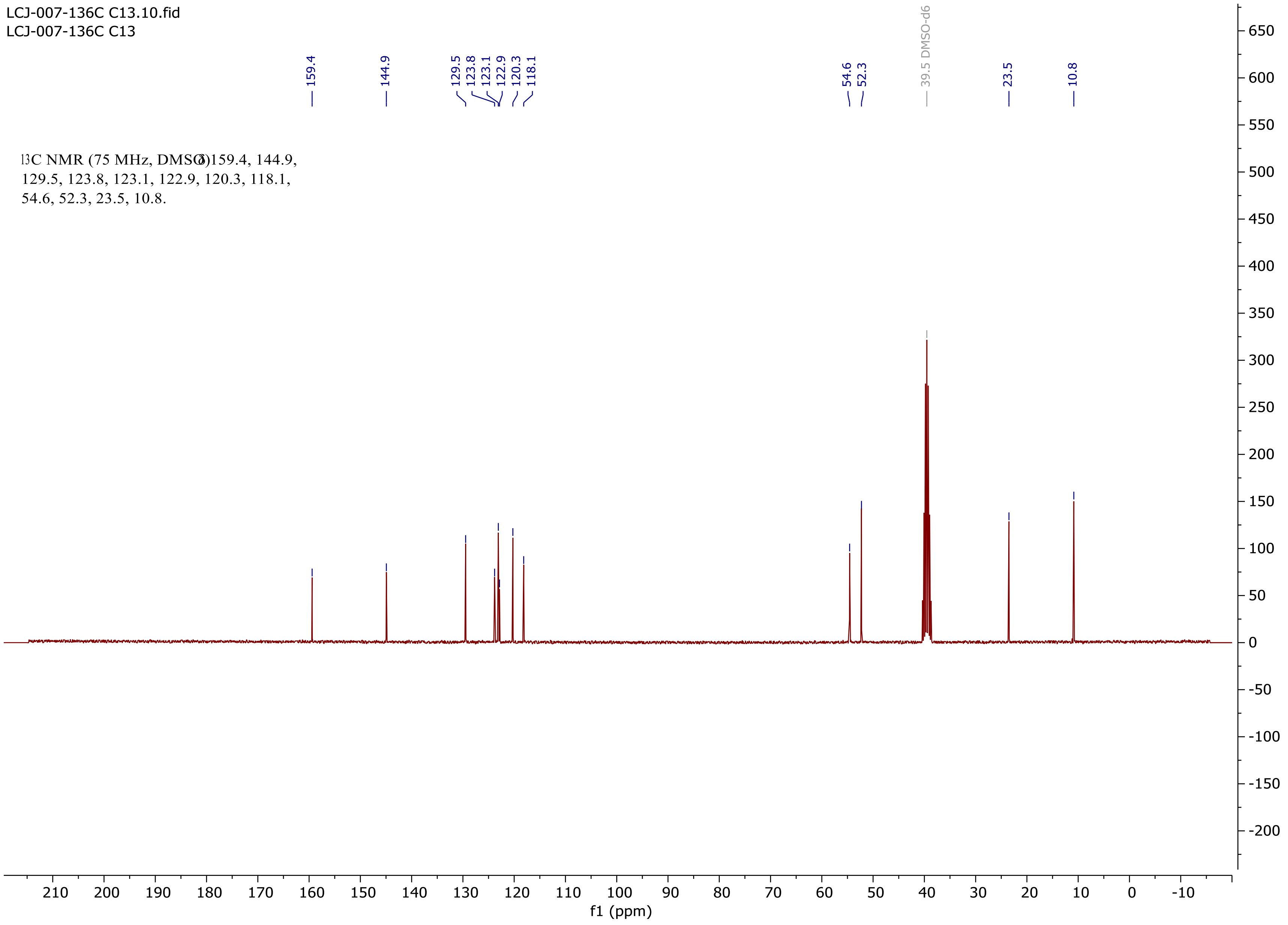

16d-^{13}C

LCJ-007-152B.10.fid
LCJ-007-152B

1H NMR (300 MHz, DMSO-d_6) δ 8.22 (d, J = 1.8 Hz, 1H), 7.92 (d, J = 9.0 Hz, 1H), 7.62 (dd, J = 9.0, 1.9 Hz, 1H), 4.98 – 4.85 (m, 1H), 3.93 (s, 3H), 2.05 – 1.76 (m, 2H), 1.50 (d, J = 6.6 Hz, 3H), 0.66 (t, J = 7.4 Hz, 3H).

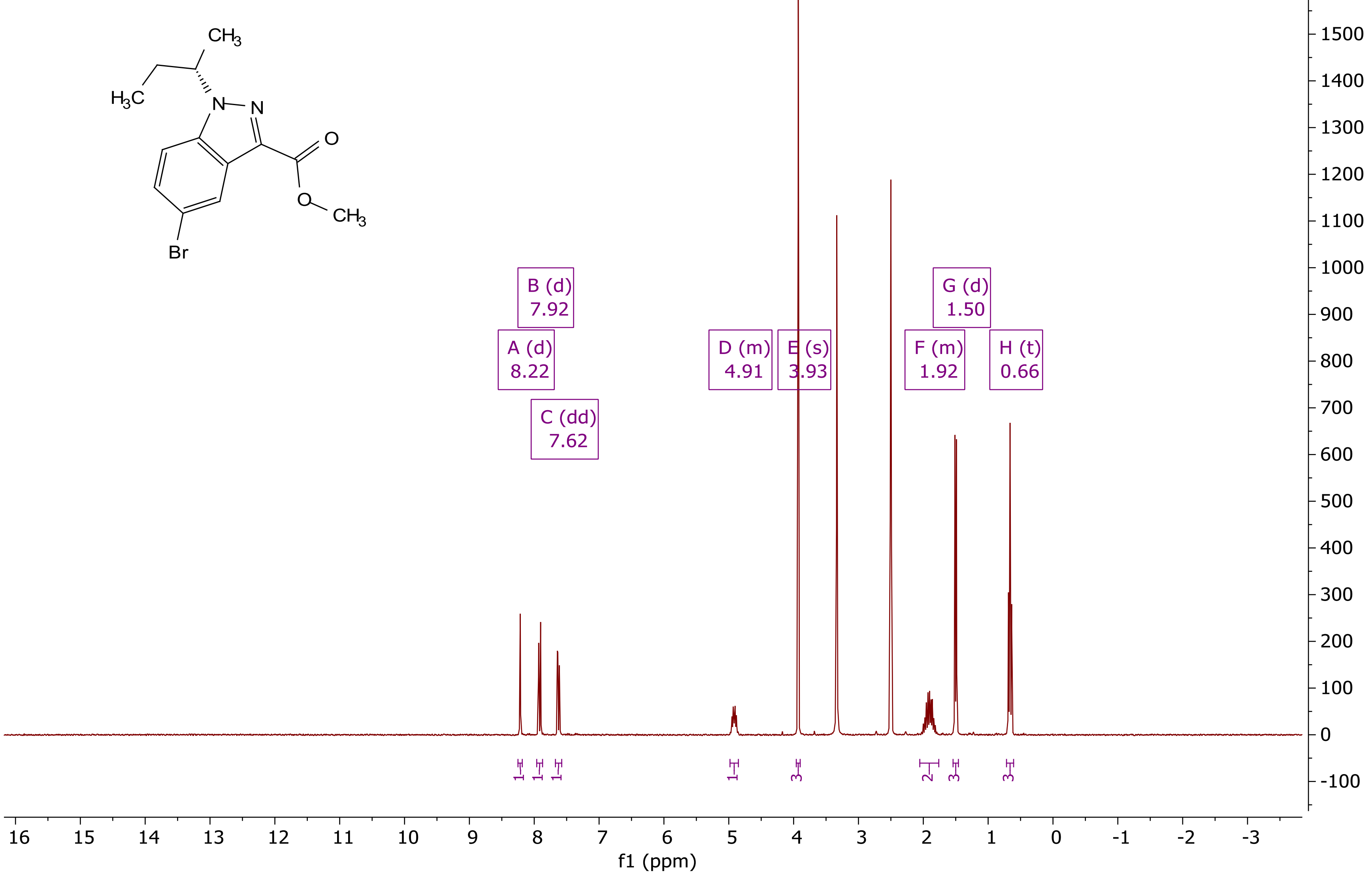

15e-^{1}H

13C NMR (75 MHz, DMSO) δ 162.0, 139.3, 133.2, 129.4, 124.0, 123.3, 116.0, 112.9, 56.5, 51.8, 29.2, 20.2, 10.5.

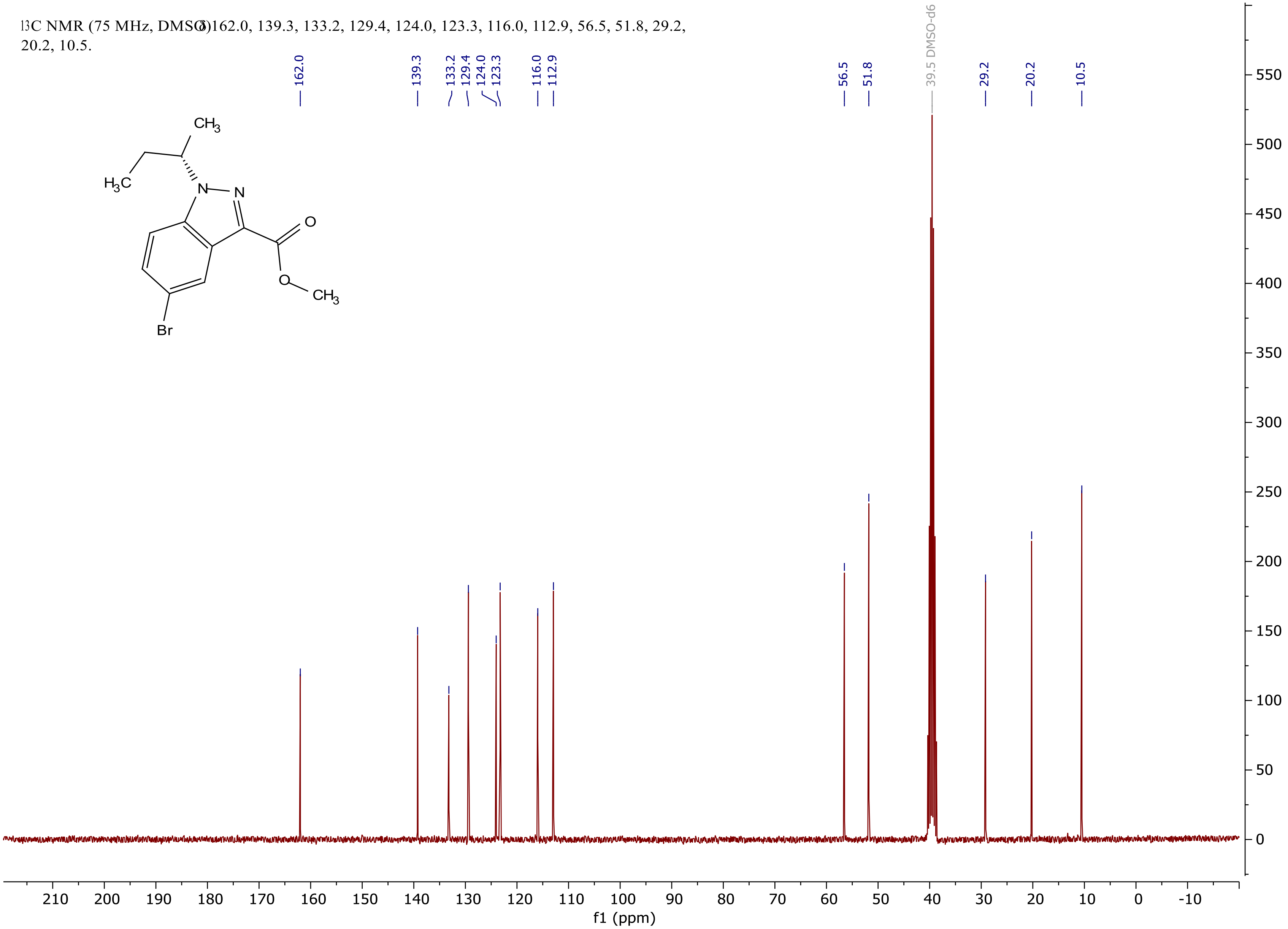

15e-^{13}C

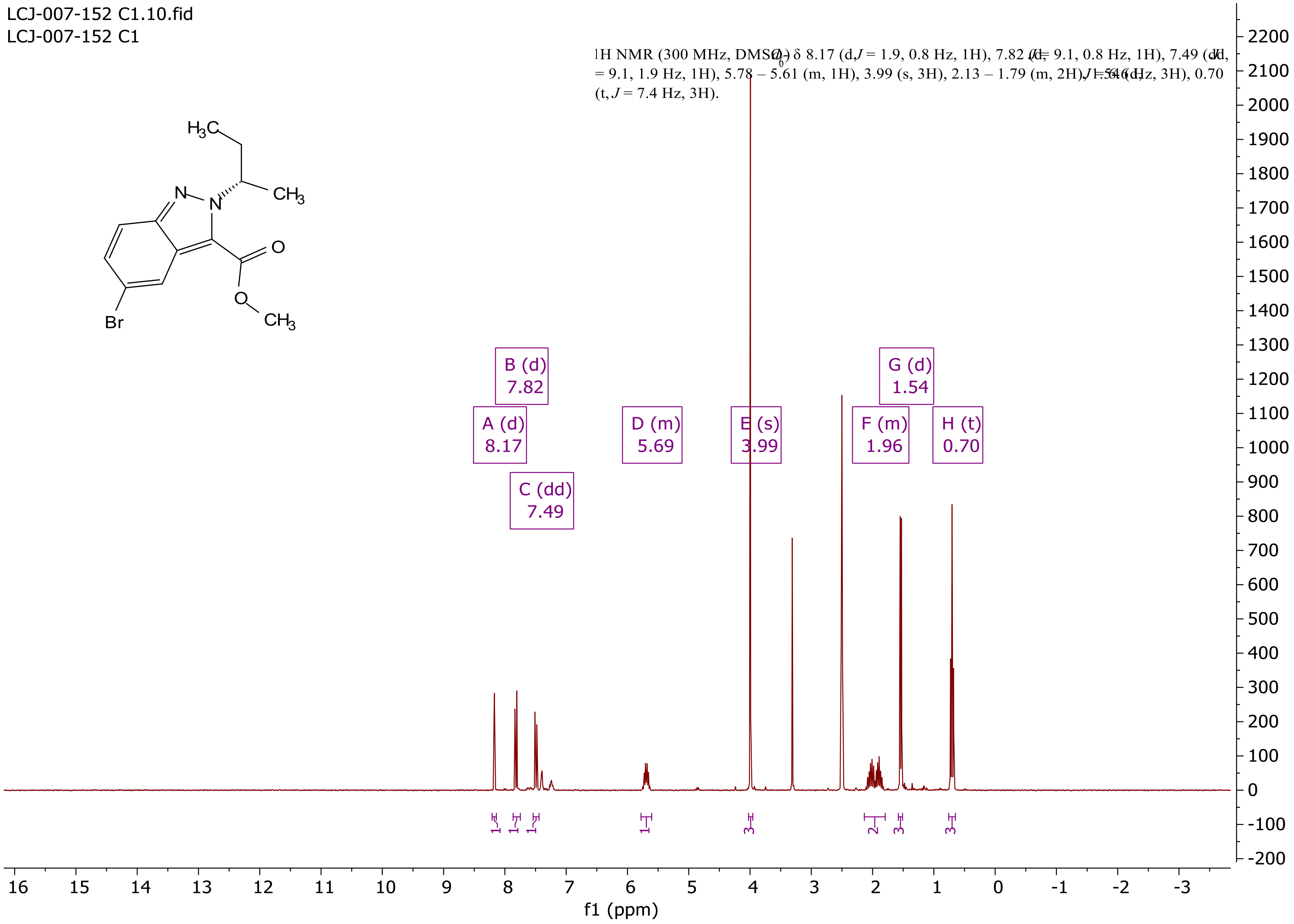
LCJ-007-152 C1.10.fid
LCJ-007-152 C1
1H NMR (300 MHz, DMSO-d_6) δ 8.17 (d, J = 1.9, 0.8 Hz, 1H), 7.82 (d, J = 9.1, 0.8 Hz, 1H), 7.49 (dd, J = 9.1, 1.9 Hz, 1H), 5.78 – 5.61 (m, 1H), 3.99 (s, 3H), 2.13 – 1.79 (m, 2H), 1.54 (d, J = 6.6 Hz, 3H), 0.70 (t, J = 7.4 Hz, 3H).
H3C
N
N
CH3
O
O
CH3
Br
B (d) 7.82
A (d) 8.17
C (dd) 7.49
D (m) 5.69
E (s) 3.99
F (m) 1.96
G (d) 1.54
H (t) 0.70
f1 (ppm)

16e-^{1}H

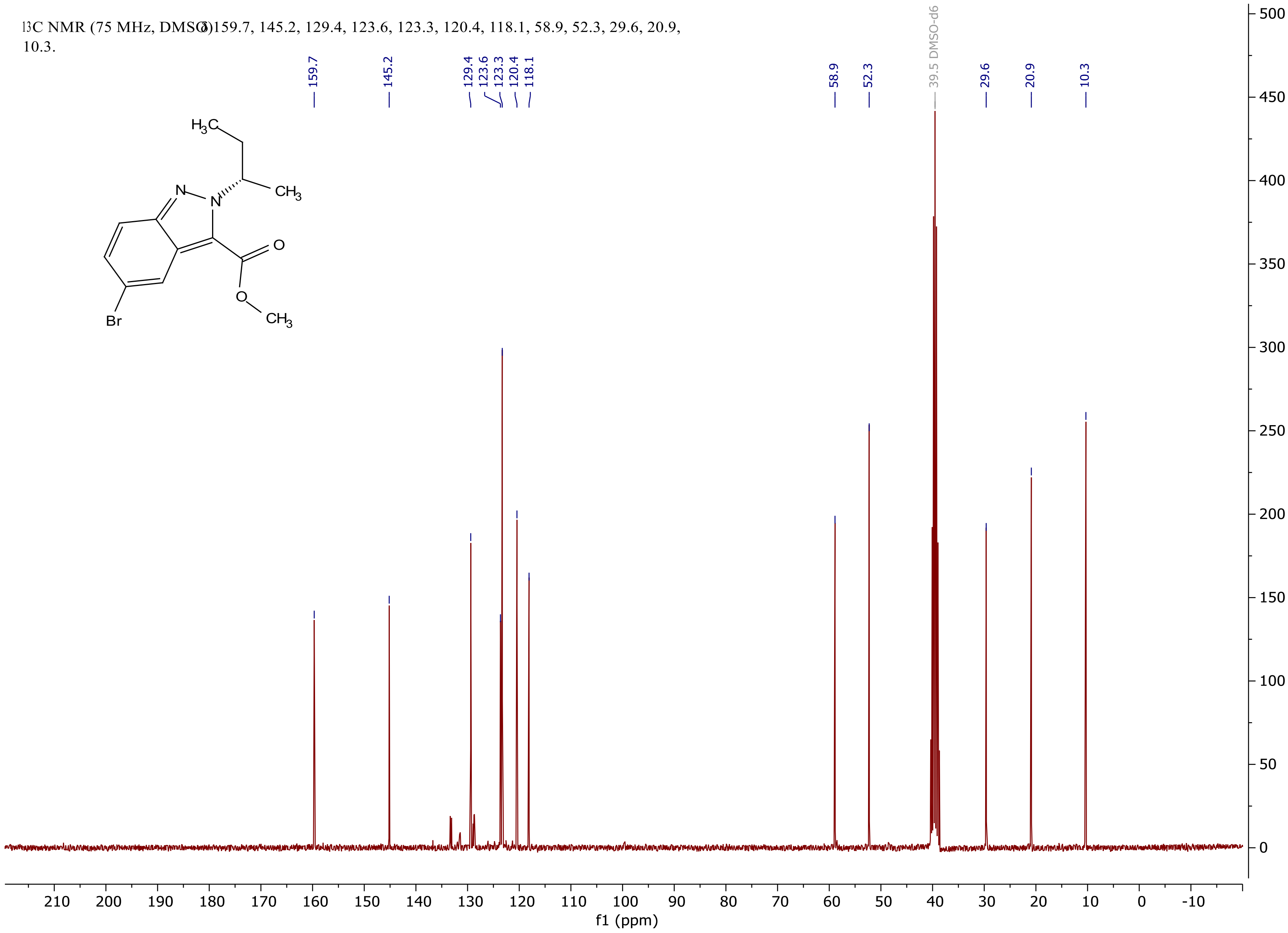
13C NMR (75 MHz, DMSO δ) 159.7, 145.2, 129.4, 123.6, 123.3, 120.4, 118.1, 58.9, 52.3, 29.6, 20.9, 10.3.
159.7
145.2
129.4
123.6
123.3
120.4
118.1
58.9
52.3
39.5 DMSO-d6
29.6
20.9
10.3
H3C
N
N
CH3
O
O
CH3
Br
f1 (ppm)

16e-^{13}C

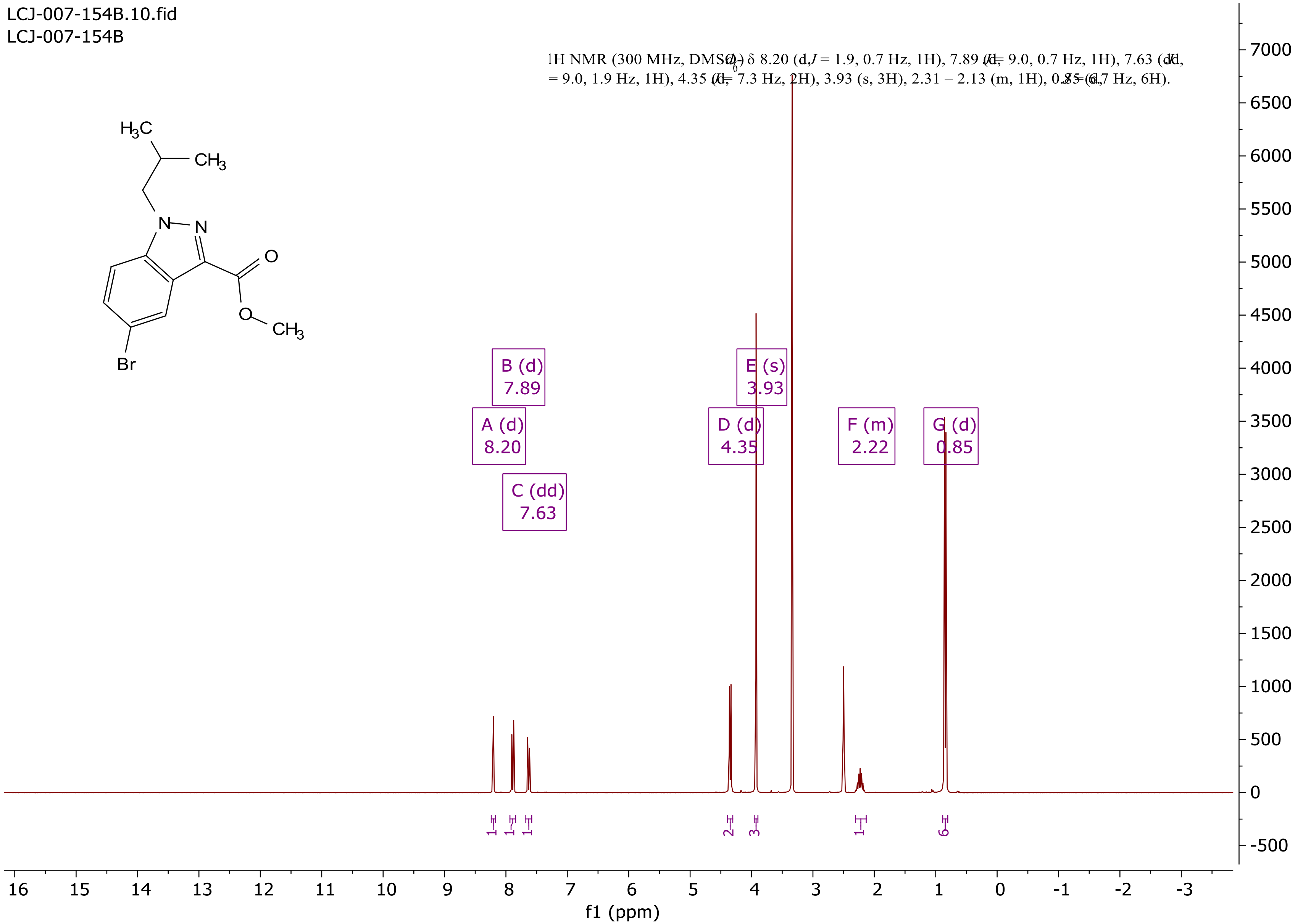

LCJ-007-154B.10.fid
LCJ-007-154B
1H NMR (300 MHz, DMSO-d6) δ 8.20 (d, J = 1.9, 0.7 Hz, 1H), 7.89 (d, J = 9.0, 0.7 Hz, 1H), 7.63 (dd, J = 9.0, 1.9 Hz, 1H), 4.35 (d, J = 7.3 Hz, 2H), 3.93 (s, 3H), 2.31 – 2.13 (m, 1H), 0.85 (d, J = 6.7 Hz, 6H).
H3C
CH3
N
N
O
O
CH3
Br
B (d) 7.89
A (d) 8.20
C (dd) 7.63
E (s) 3.93
D (d) 4.35
F (m) 2.22
G (d) 0.85
f1 (ppm)

15f-^{1}H

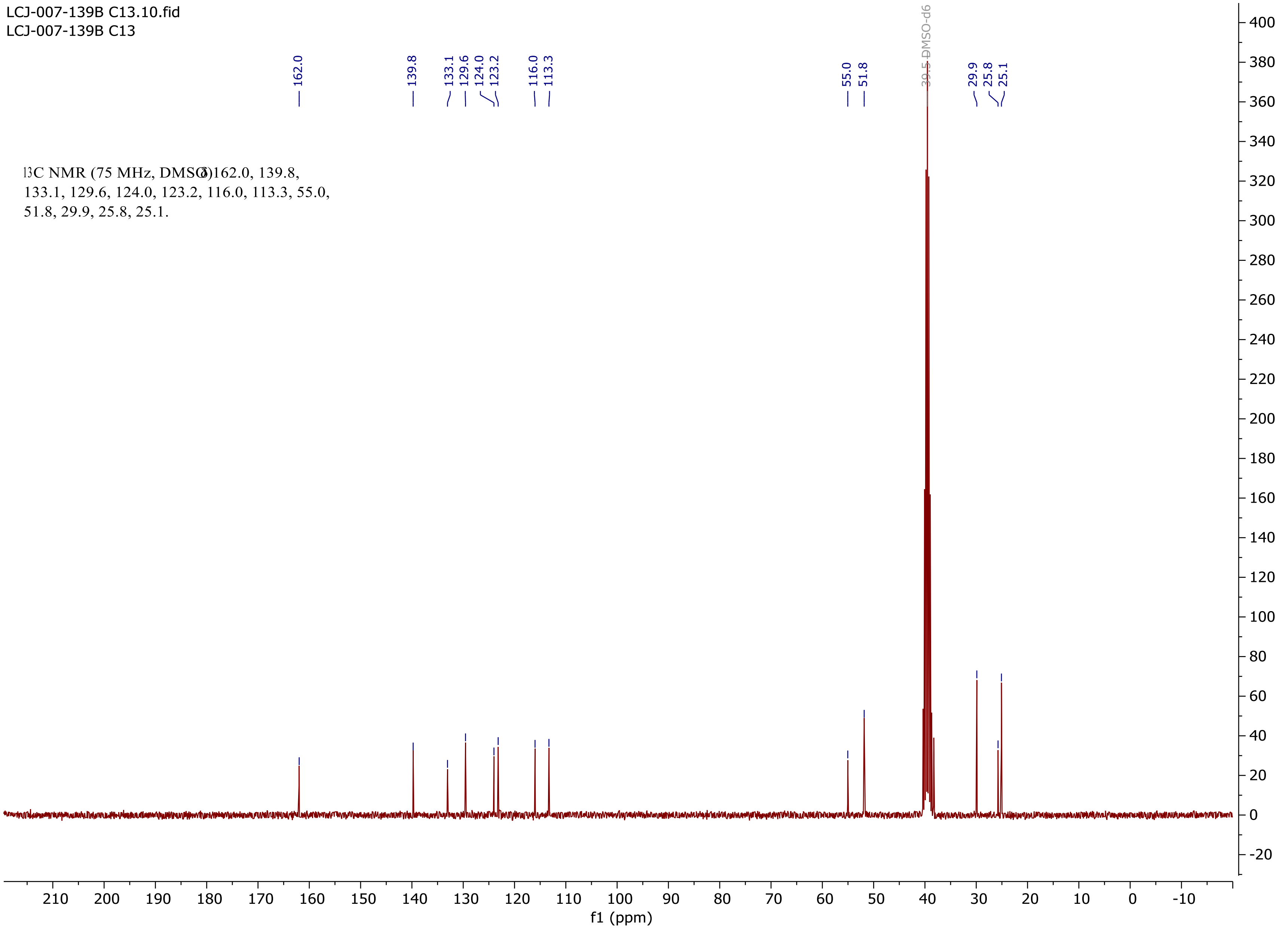
LCJ-007-139B C13.10.fid
LCJ-007-139B C13
162.0
139.8
133.1
129.6
124.0
123.2
116.0
113.3
55.0
51.8
39.5 DMSO-d6
29.9
25.8
25.1
13C NMR (75 MHz, DMSO) δ 162.0, 139.8, 133.1, 129.6, 124.0, 123.2, 116.0, 113.3, 55.0, 51.8, 29.9, 25.8, 25.1.
f1 (ppm)

15f-^{13}C

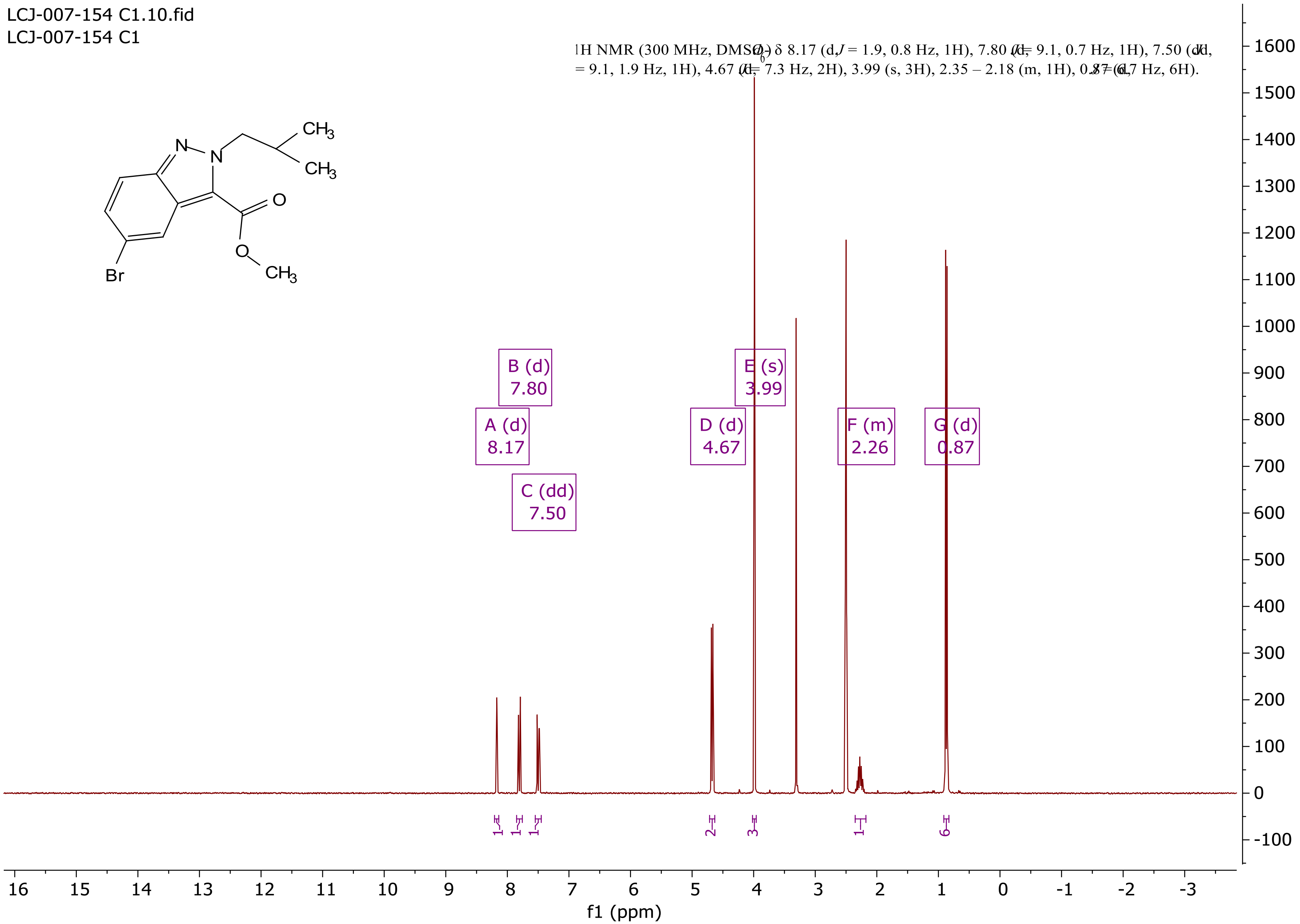
LCJ-007-154 C1.10.fid
LCJ-007-154 C1
1H NMR (300 MHz, DMSO-d6) δ 8.17 (d, J = 1.9, 0.8 Hz, 1H), 7.80 (d, J = 9.1, 0.7 Hz, 1H), 7.50 (dd, J = 9.1, 1.9 Hz, 1H), 4.67 (d, J = 7.3 Hz, 2H), 3.99 (s, 3H), 2.35 – 2.18 (m, 1H), 0.87 (d, J = 6.7 Hz, 6H).
N
N
CH3
CH3
O
O
CH3
Br
B (d) 7.80
A (d) 8.17
C (dd) 7.50
D (d) 4.67
E (s) 3.99
F (m) 2.26
G (d) 0.87
f1 (ppm)

16f-1H

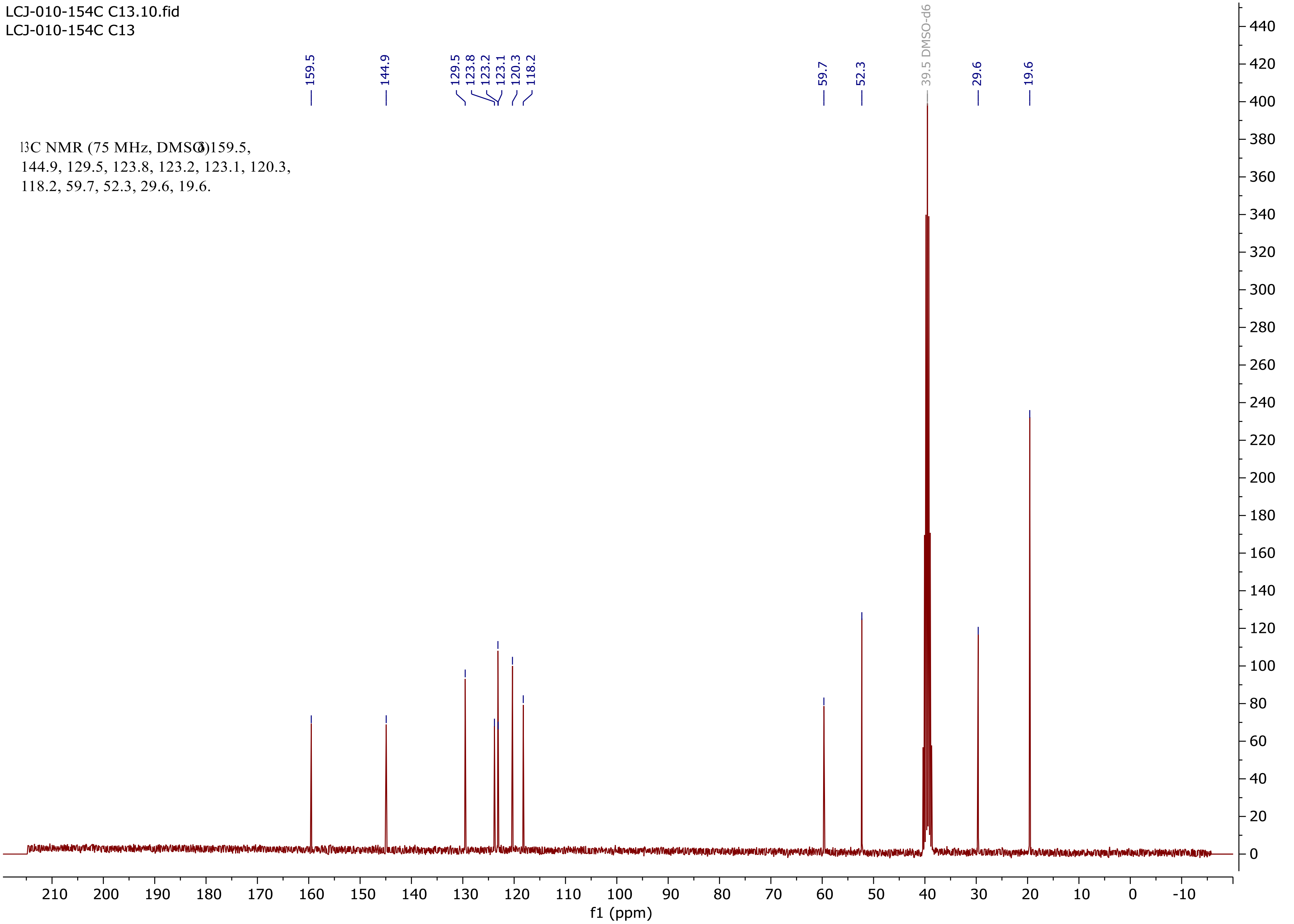

^{13}C NMR (75 MHz, DMSO[illegible]) 159.5, 144.9, 129.5, 123.8, 123.2, 123.1, 120.3, 118.2, 59.7, 52.3, 29.6, 19.6.

16f-^{13}C

LCJ-007-143 B2.10.fid
LCJ-007-143 B2

1H NMR (300 MHz, DMSO-d_6) δ 8.21 (d, J = 1.9, 0.7 Hz, 1H), 7.85 (d, J = 9.0, 0.7 Hz, 1H), 7.64 (dd, J = 9.0, 1.9 Hz, 1H), 4.71 (t, J = 5.1 Hz, 2H), 3.94 (s, 3H), 3.79 (t, J = 5.1 Hz, 2H), 3.18 (s, 3H).

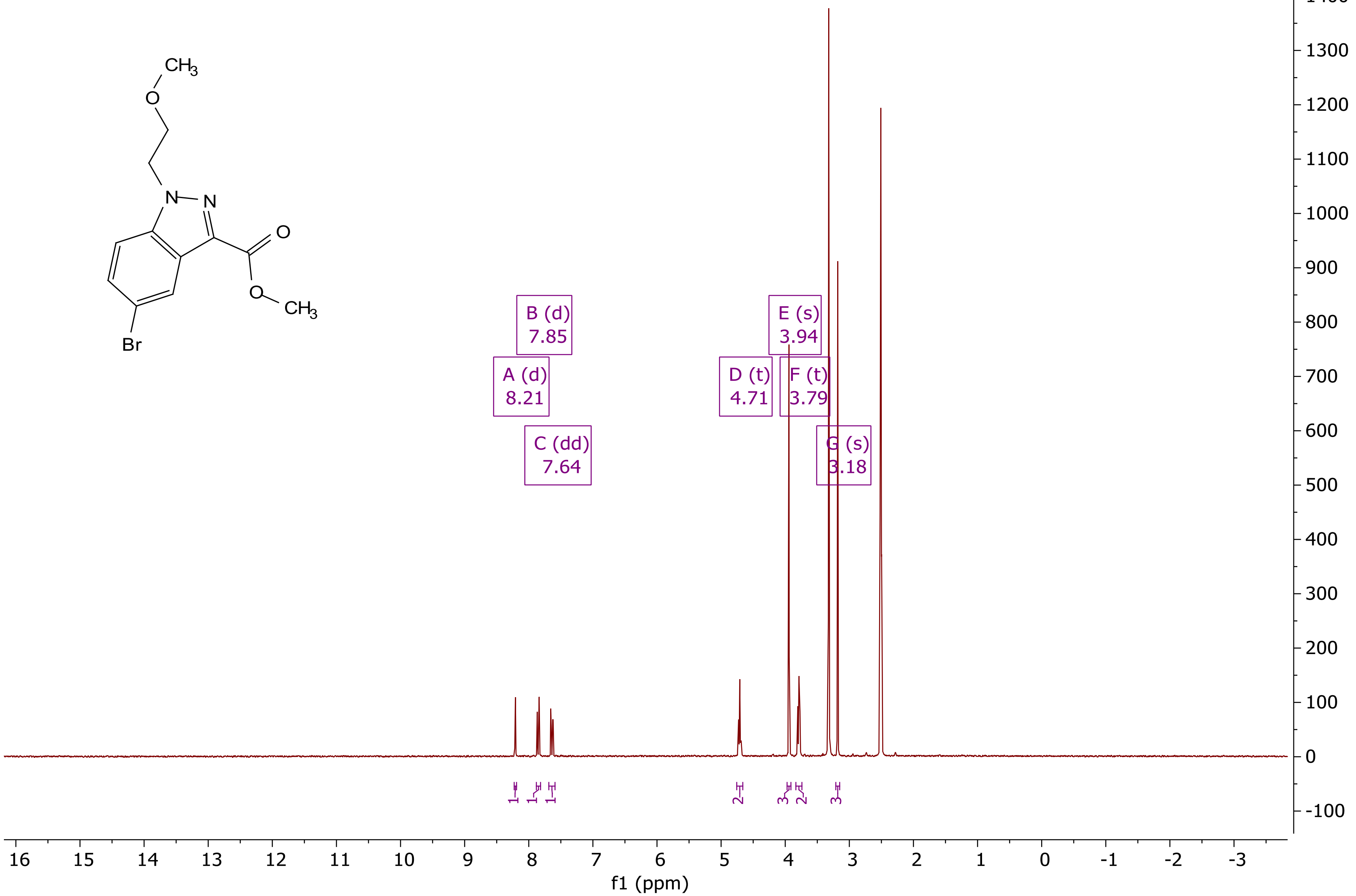

15g-^{1}H

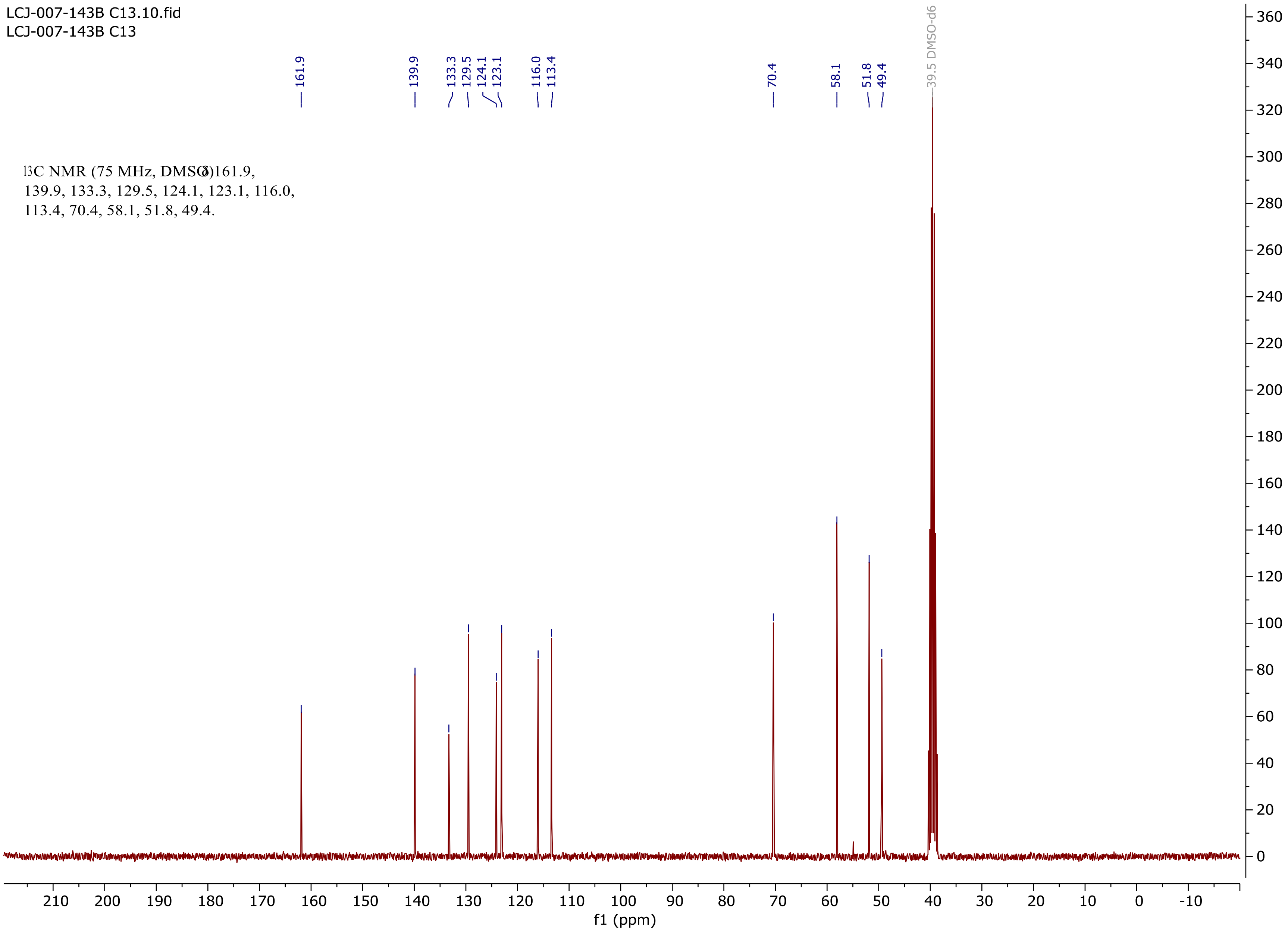
LCJ-007-143B C13.10.fid
LCJ-007-143B C13
161.9
139.9
133.3
129.5
124.1
123.1
116.0
113.4
70.4
58.1
51.8
49.4
39.5 DMSO-d6
13C NMR (75 MHz, DMSO) δ 161.9, 139.9, 133.3, 129.5, 124.1, 123.1, 116.0, 113.4, 70.4, 58.1, 51.8, 49.4.
f1 (ppm)

15g-^{13}C

LCJ-007-143 C1.10.fid
LCJ-007-143 C1

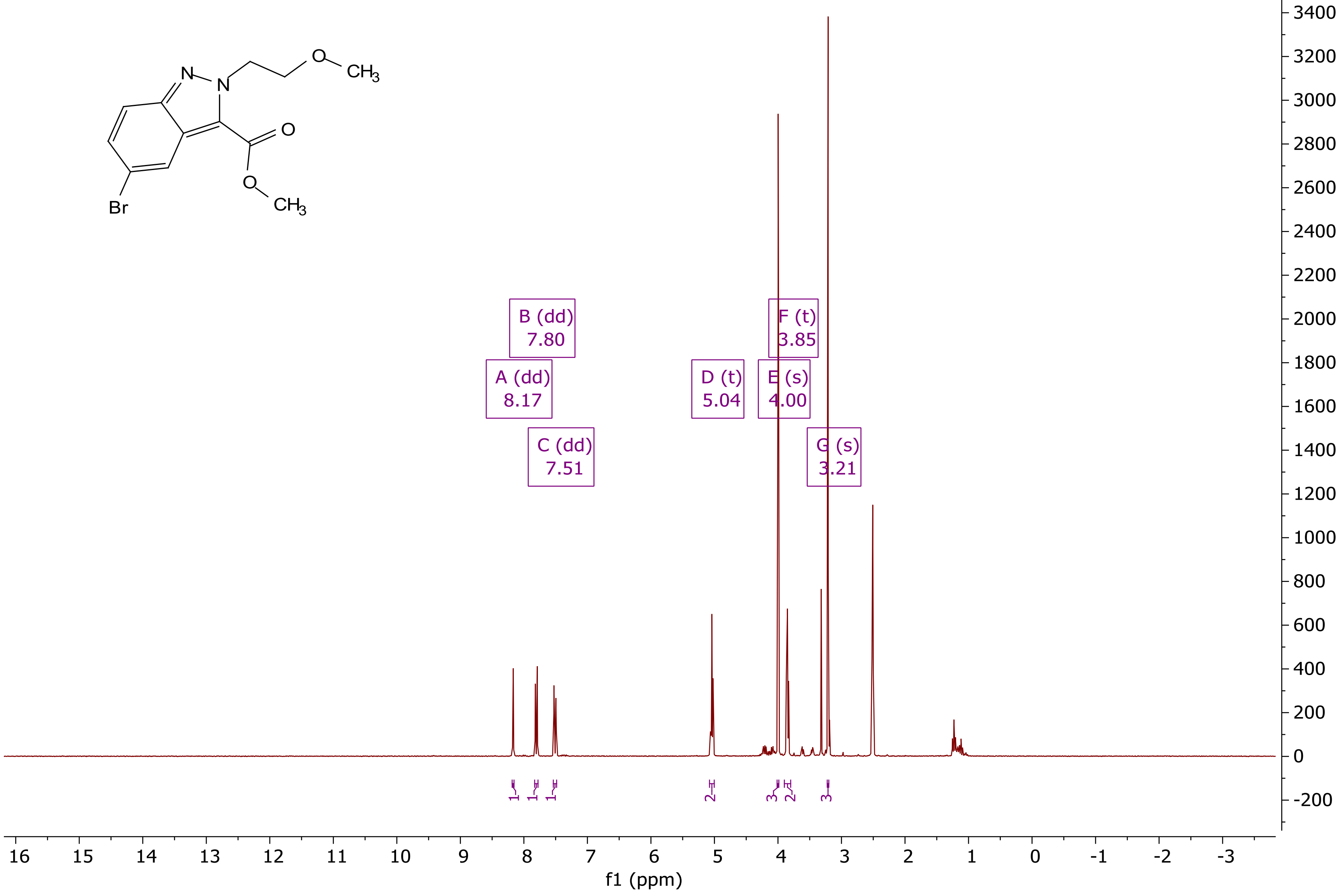

1H NMR (300 MHz, DMSO-d_6) δ 8.17 (dd, J = 1.9, 0.8 Hz, 1H), 7.80 (dd, J = 9.1, 0.8 Hz, 1H), 7.51 (dd, J = 9.1, 1.9 Hz, 1H), 5.04 (t, J = 5.5 Hz, 2H), 4.00 (s, 3H), 3.85 (t, J = 5.5 Hz, 2H), 3.21 (s, 3H).

16g-1H

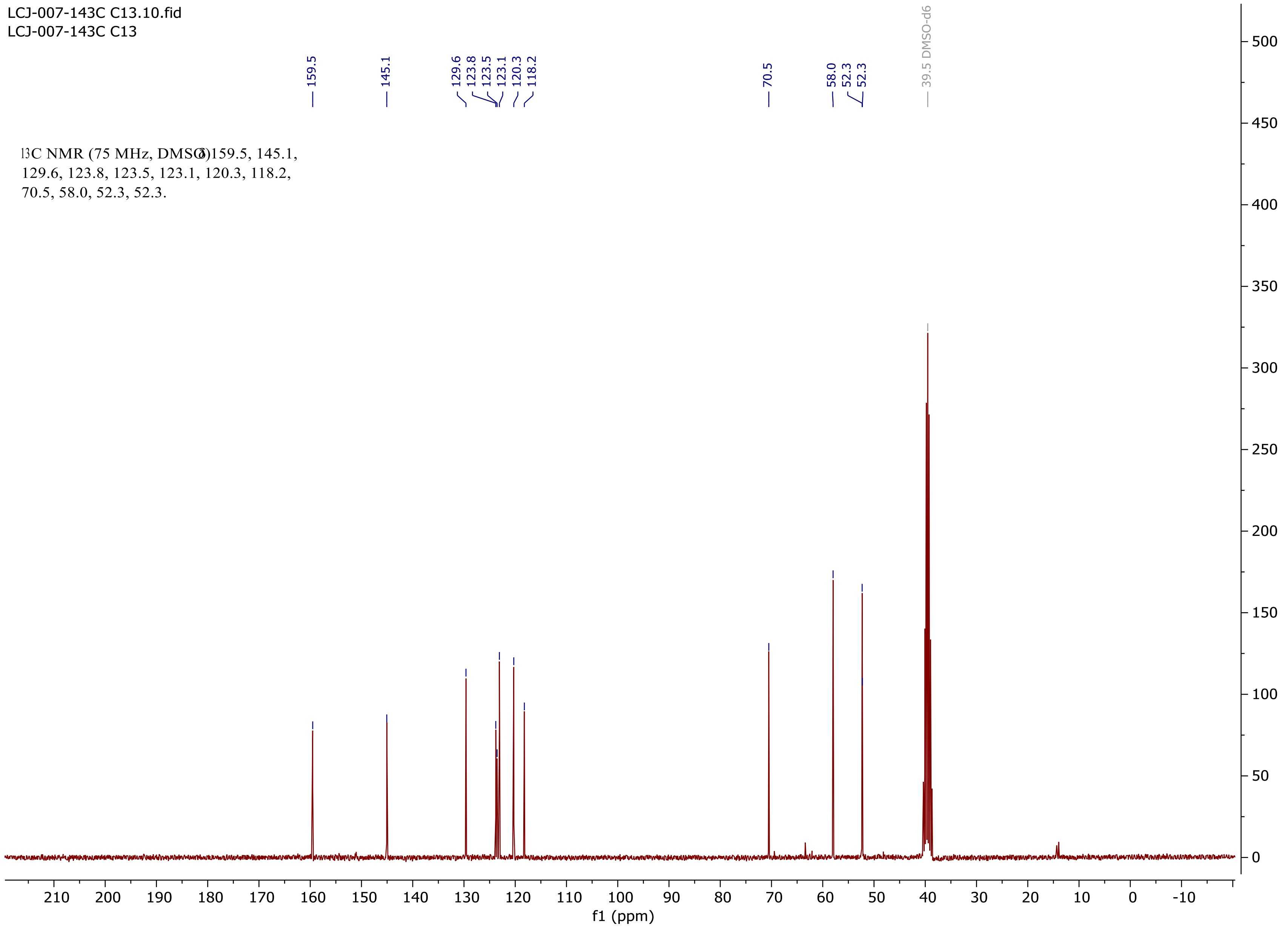
LCJ-007-143C C13.10.fid
LCJ-007-143C C13
159.5
145.1
129.6
123.8
123.5
123.1
120.3
118.2
70.5
58.0
52.3
52.3
39.5 DMSO-d6
13C NMR (75 MHz, DMSO) δ 159.5, 145.1, 129.6, 123.8, 123.5, 123.1, 120.3, 118.2, 70.5, 58.0, 52.3, 52.3.
f1 (ppm)

16g-^{13}C

LCJ-007-140 B2.10.fid
LCJ-007-140 B2

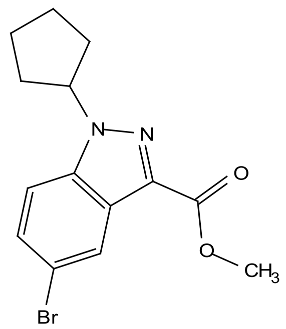

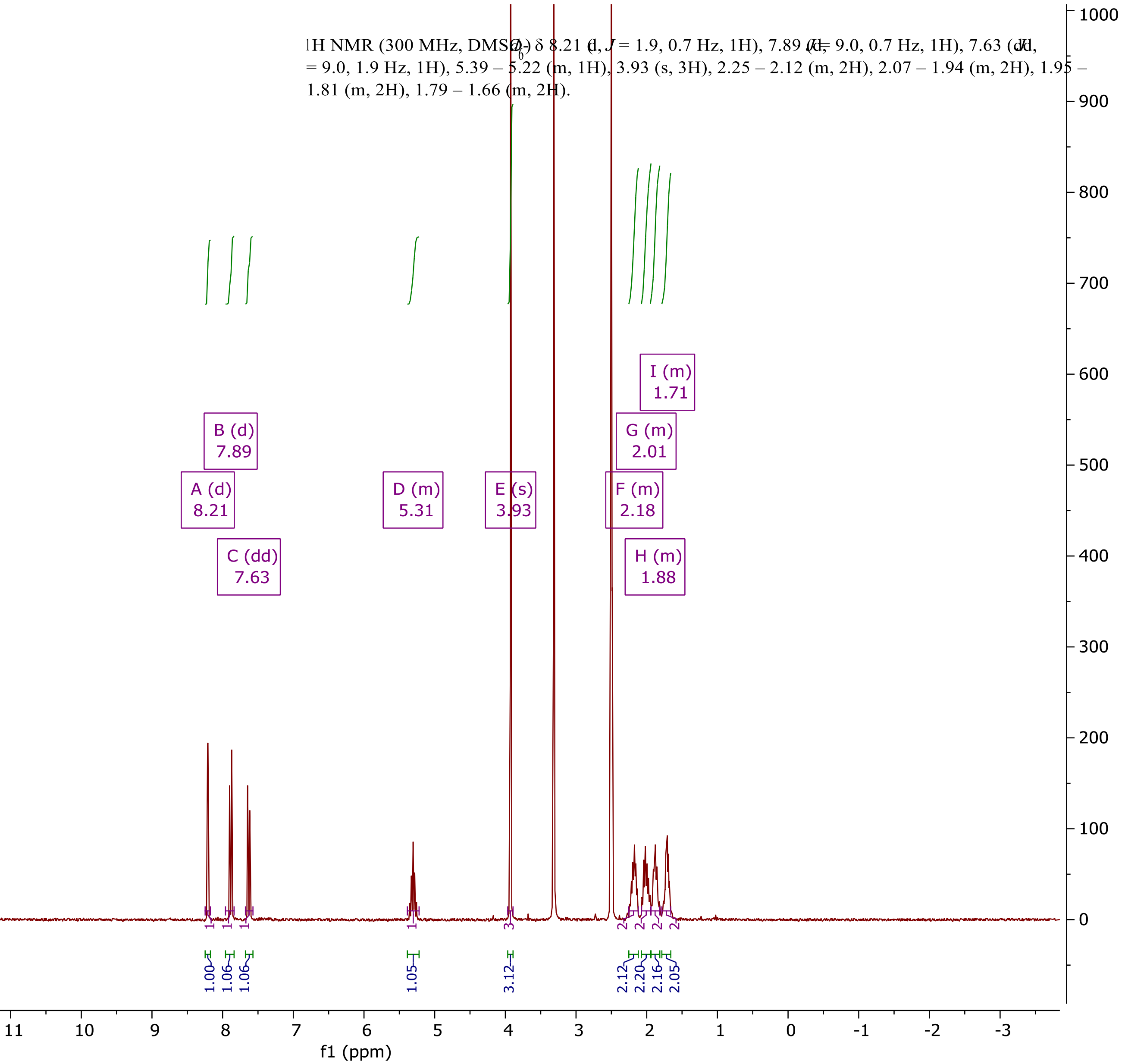

15h-^{1}H

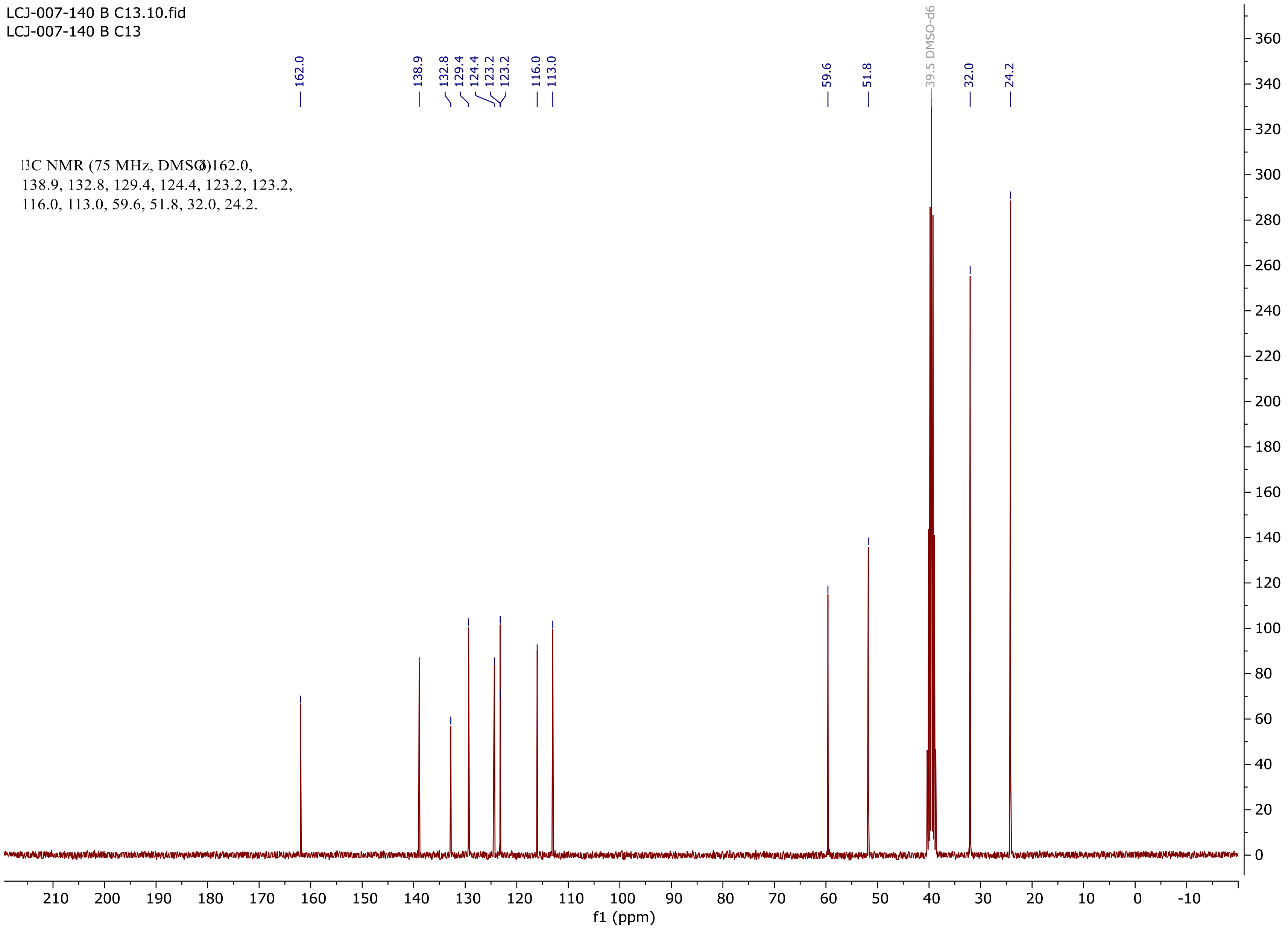
LCJ-007-140 B C13.10.fid
LCJ-007-140 B C13
13C NMR (75 MHz, DMSO) δ 162.0,
138.9, 132.8, 129.4, 124.4, 123.2, 123.2,
116.0, 113.0, 59.6, 51.8, 32.0, 24.2.
162.0
138.9
132.8
129.4
124.4
123.2
123.2
116.0
113.0
59.6
51.8
39.5 DMSO-d6
32.0
24.2
f1 (ppm)

15h-^{13}C

LCJ-007-140 C1.10.fid
LCJ-007-140 C1

1H NMR (300 MHz, DMSO-d_6) δ 8.17 (d, J = 1.9, 0.8 Hz, 1H), 7.81 (dd, J = 9.1, 0.8 Hz, 1H), 7.49 (dd, J = 9.1, 1.9 Hz, 1H), 6.04 – 5.88 (m, 1H), 4.00 (s, 3H), 2.29 – 2.17 (m, 2H), 2.16 – 2.04 (m, 2H), 1.98 – 1.84 (m, 2H), 1.79 – 1.63 (m, 2H).

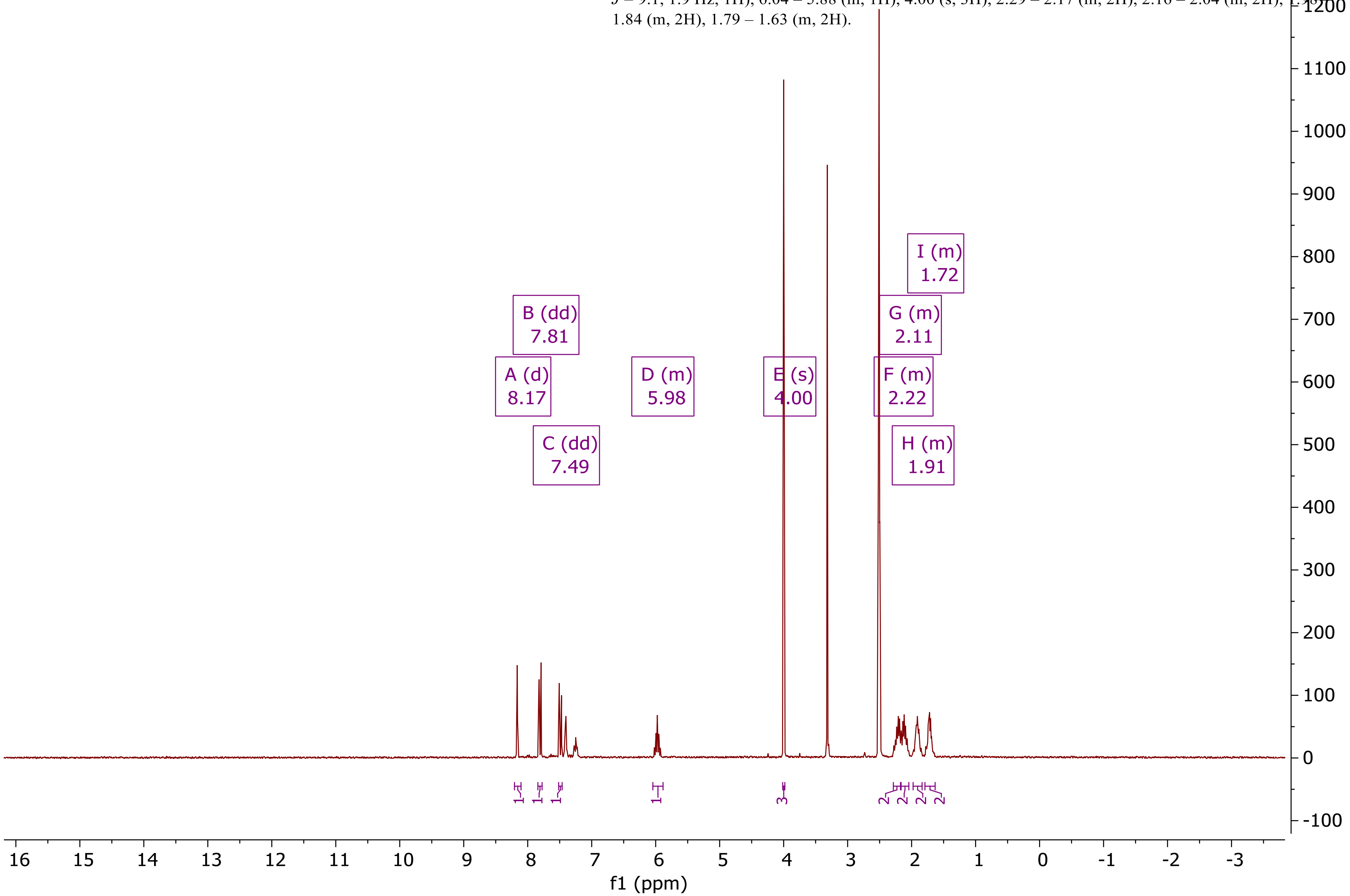

16h-^{1}H

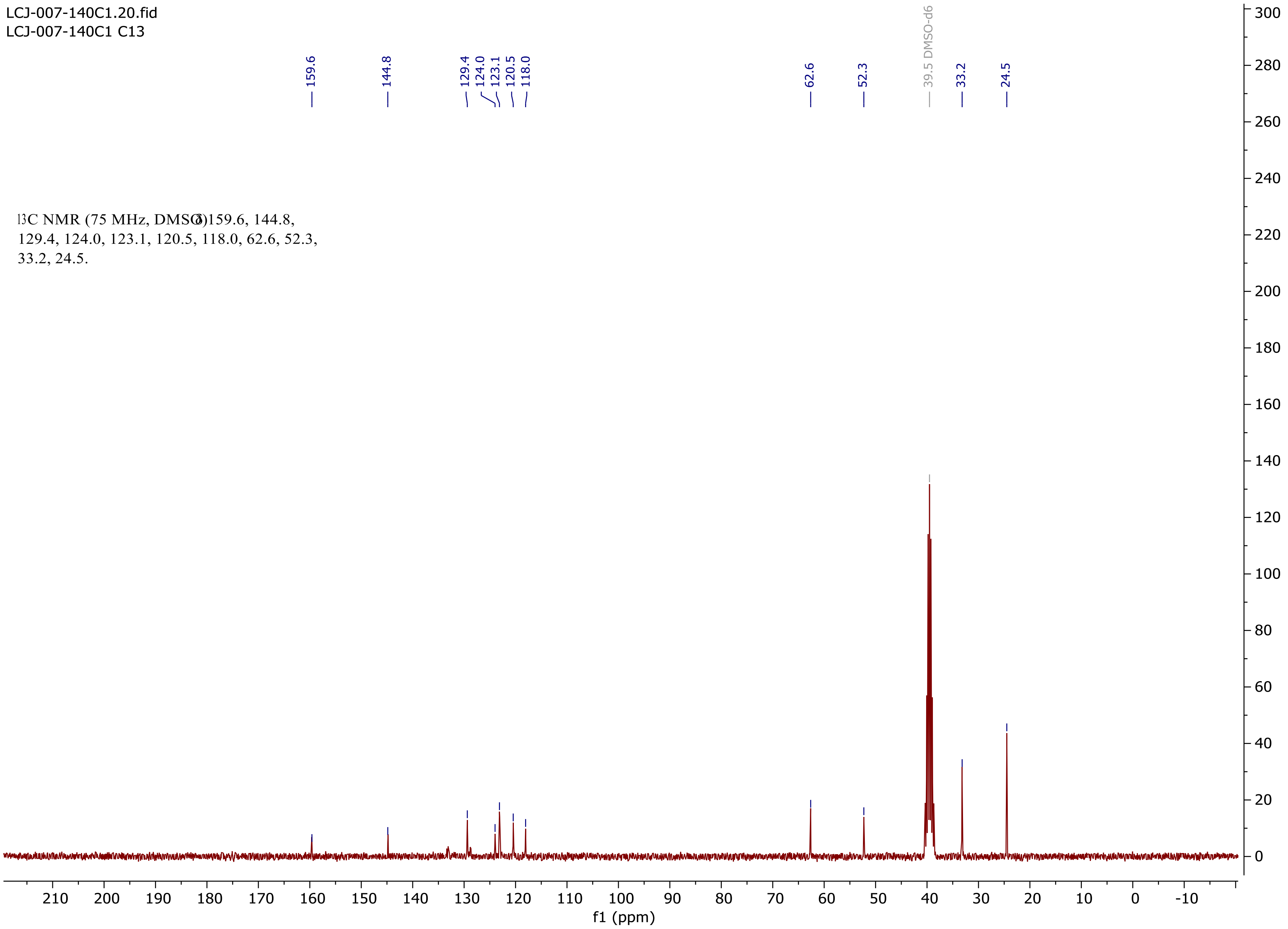
LCJ-007-140C1.20.fid
LCJ-007-140C1 C13
159.6
144.8
129.4
124.0
123.1
120.5
118.0
62.6
52.3
39.5 DMSO-d6
33.2
24.5
13C NMR (75 MHz, DMSO) δ 159.6, 144.8, 129.4, 124.0, 123.1, 120.5, 118.0, 62.6, 52.3, 33.2, 24.5.
f1 (ppm)

16h-^{13}C

1H NMR (300 MHz, DMSO-d_6) δ 8.22 (d, J = 1.8 Hz, 1H), 7.92 (d, J = 9.0 Hz, 1H), 7.67 (dd, J = 9.0, 1.9 Hz, 1H), 5.59 (d, J = 6.4 Hz, 1H), 3.93 (s, 3H), 3.88 – 3.78 (m, 1H), 3.65 – 3.54 (m, 2H), 3.46 (d, J = 7.1 Hz, 1H), 2.39 (s, 2H), 1.40 (d, J = 8.8 Hz, 9H).

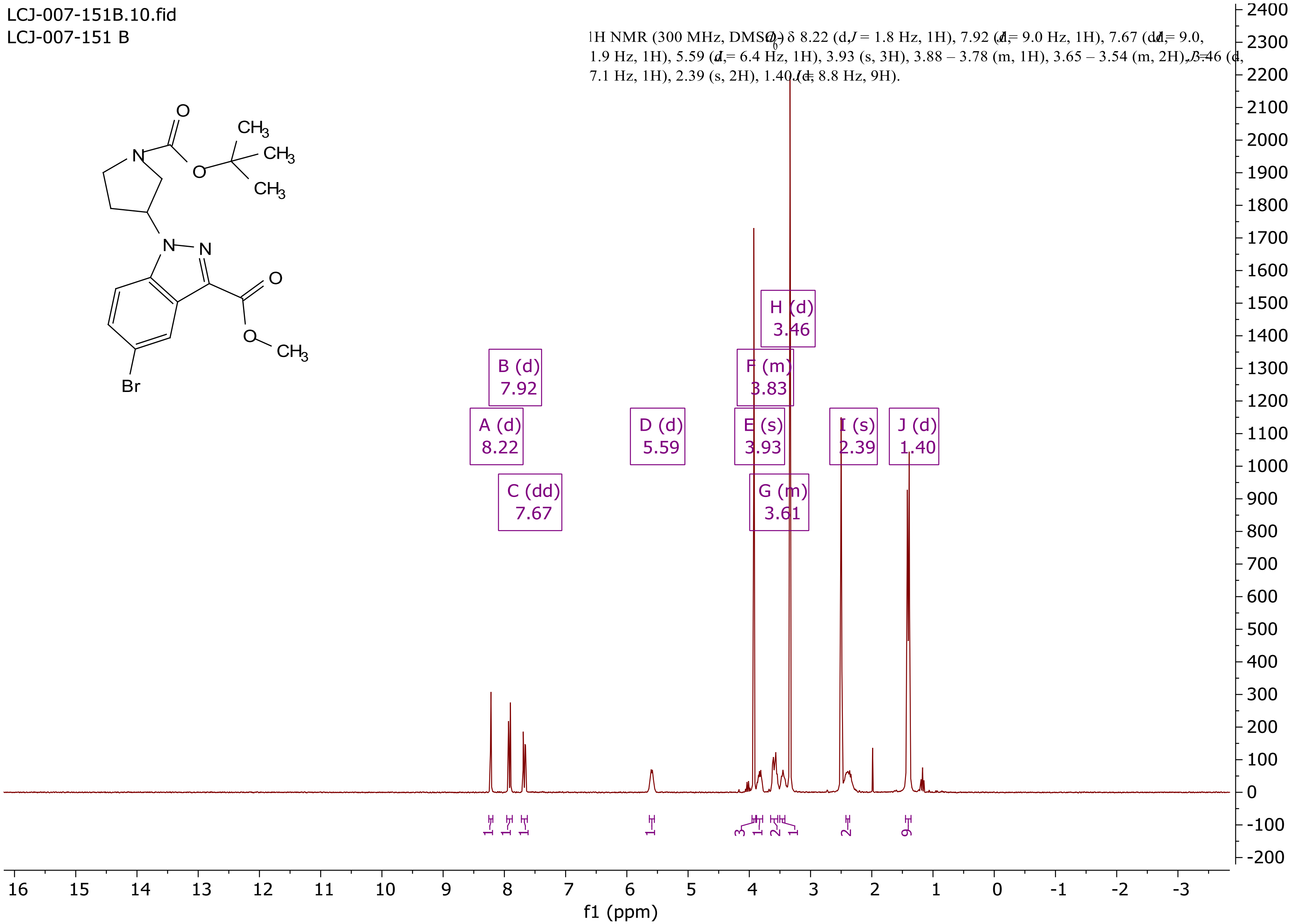

15i-¹H

LCJ-007-151B C13.10.fid
LCJ-007-151B C13

13C NMR (75 MHz, DMSO) δ 161.8, 153.5, 153.4, 139.1, 133.5, 129.8, 124.3, 123.3, 116.3, 113.0, 78.7, 57.5, 56.8, 56.8, 51.9, 50.4, 50.4, 44.7, 44.4, 31.0, 30.2, 28.1.

mixture of rotomers

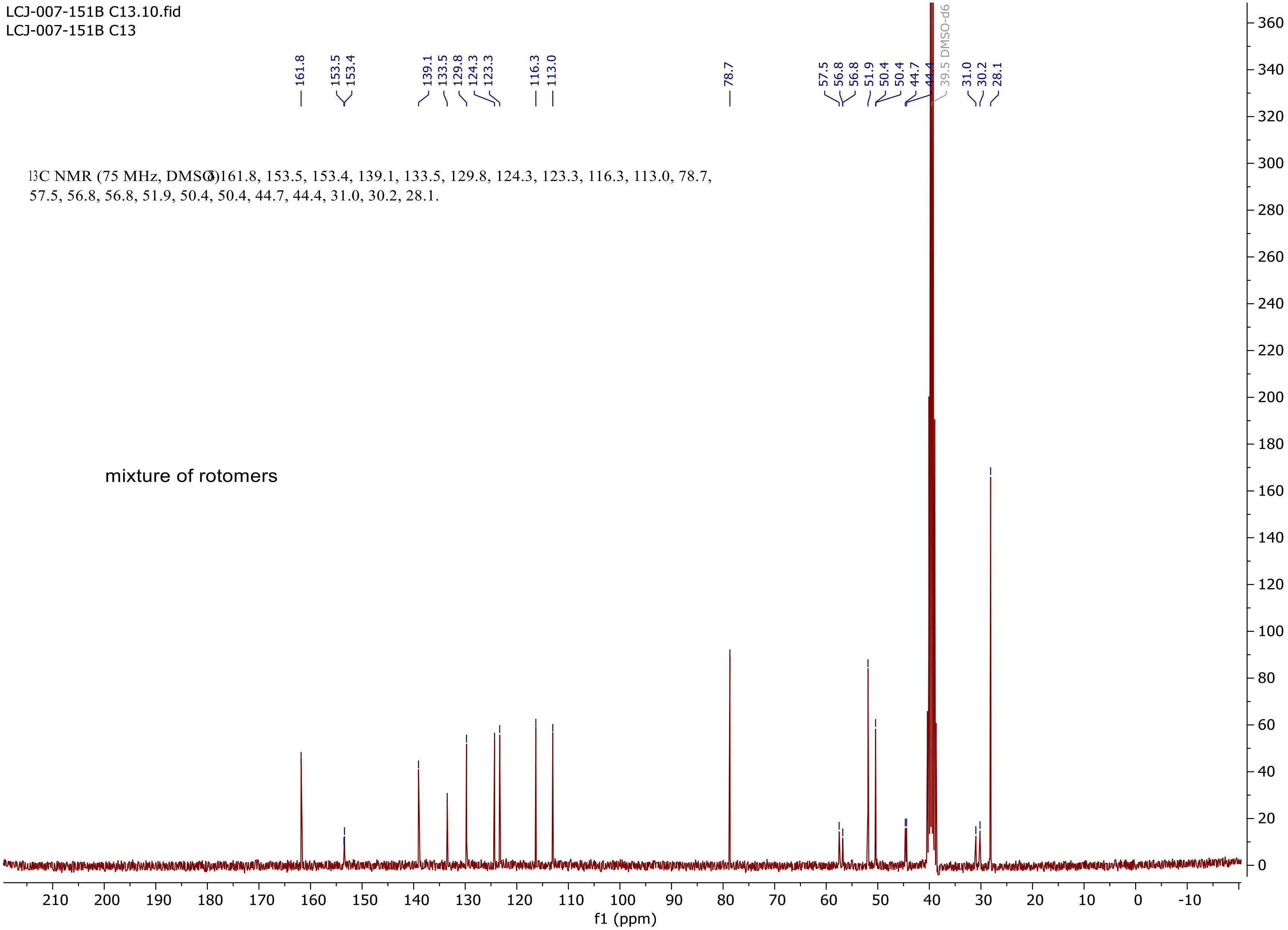

15i-^{13}C

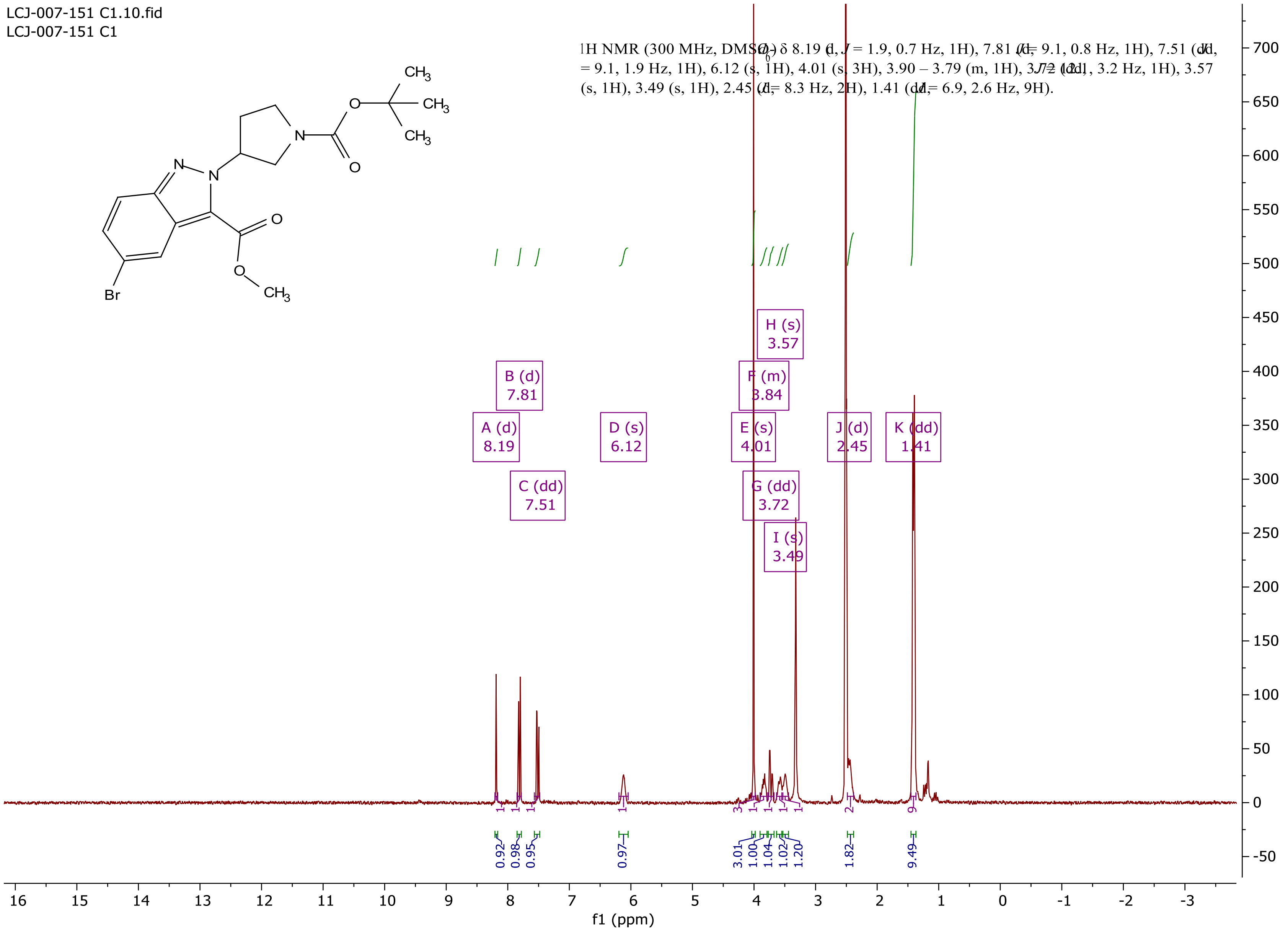
LCJ-007-151 C1.10.fid
LCJ-007-151 C1
1H NMR (300 MHz, DMSO-d6) δ 8.19 (d, J = 1.9, 0.7 Hz, 1H), 7.81 (d, J = 9.1, 0.8 Hz, 1H), 7.51 (dd, J = 9.1, 1.9 Hz, 1H), 6.12 (s, 1H), 4.01 (s, 3H), 3.90 – 3.79 (m, 1H), 3.72 (dd, J = 12.1, 3.2 Hz, 1H), 3.57 (s, 1H), 3.49 (s, 1H), 2.45 (d, J = 8.3 Hz, 2H), 1.41 (dd, J = 6.9, 2.6 Hz, 9H).
A (d) 8.19
B (d) 7.81
C (dd) 7.51
D (s) 6.12
E (s) 4.01
F (m) 3.84
G (dd) 3.72
H (s) 3.57
I (s) 3.49
J (d) 2.45
K (dd) 1.41
0.92
0.98
0.95
0.97
3.01
1.00
1.04
1.02
1.20
1.82
9.49
f1 (ppm)

16i-^{1}H

LCJ-007-151C C13.10.fid
LCJ-007-151C C13

^{13}C NMR (75 MHz, DMSO δ) 159.6, 153.5, 153.4, 144.9, 129.7, 123.9, 123.4, 123.1, 120.5, 118.4, 78.6, 61.2, 60.5, 52.4, 51.8, 44.9, 44.7, 31.7, 30.9, 28.1.

mixture of rotomers

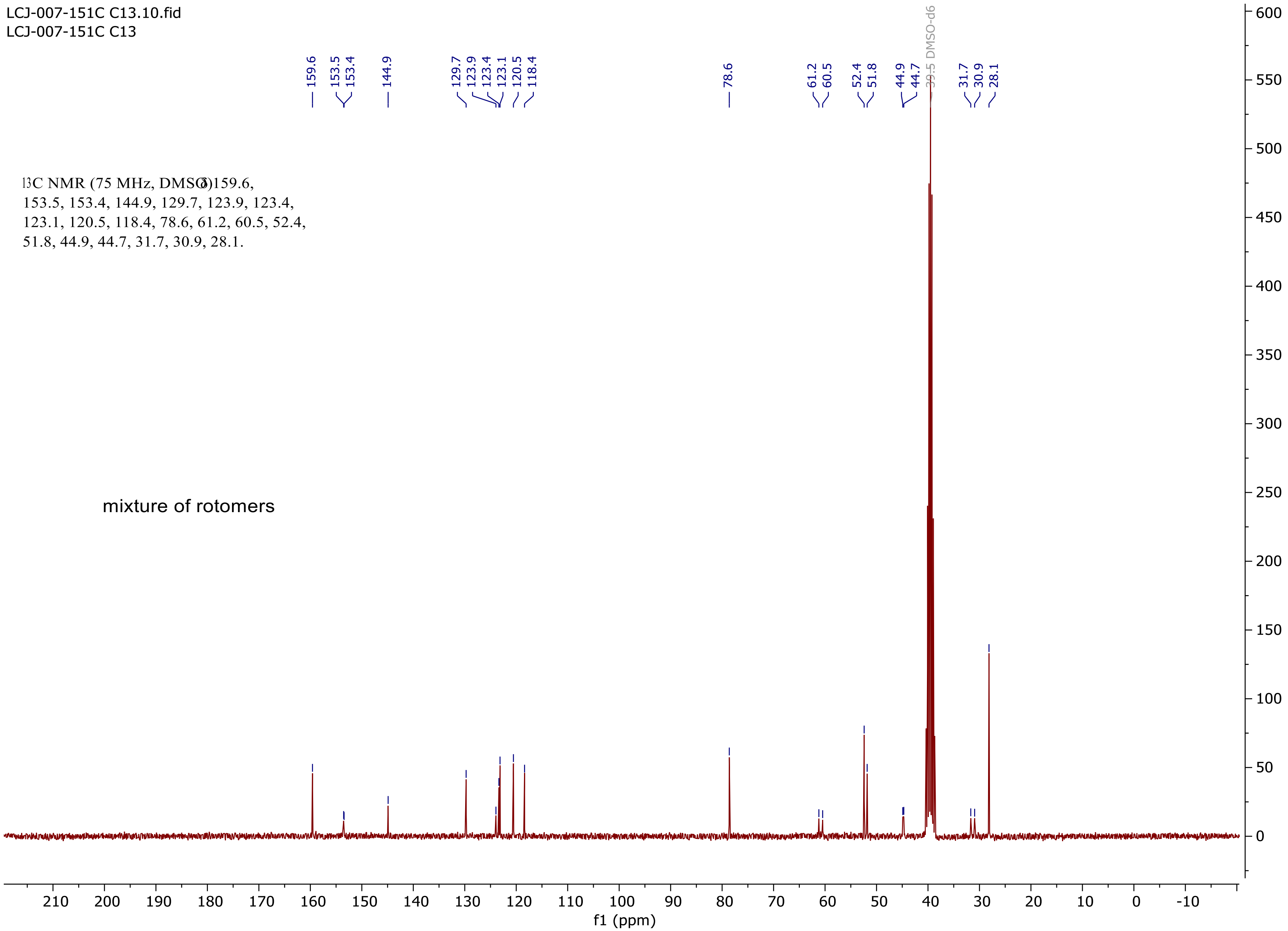

16i-^{13}C

LCJ-007-147 B2.10.fid
LCJ-007-147 B2

1H NMR (300 MHz, DMSO-d_6) δ 8.23 (d, J = 1.9, 0.7 Hz, 1H), 7.91 (d, J = 9.0, 0.7 Hz, 1H), 7.66 (dd, J = 9.0, 1.9 Hz, 1H), 5.68 – 5.59 (m, 1H), 4.14 – 4.04 (m, 2H), 3.98 (d, J = [illegible] Hz, 1H), 3.93 (s, 3H), 3.91 – 3.82 (m, 1H), 2.49 – 2.44 (m, 1H), 2.40 – 2.28 (m, 1H).

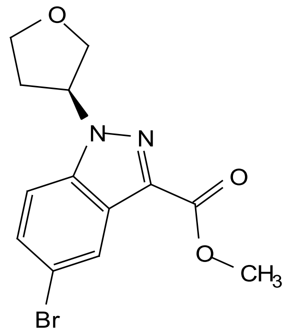

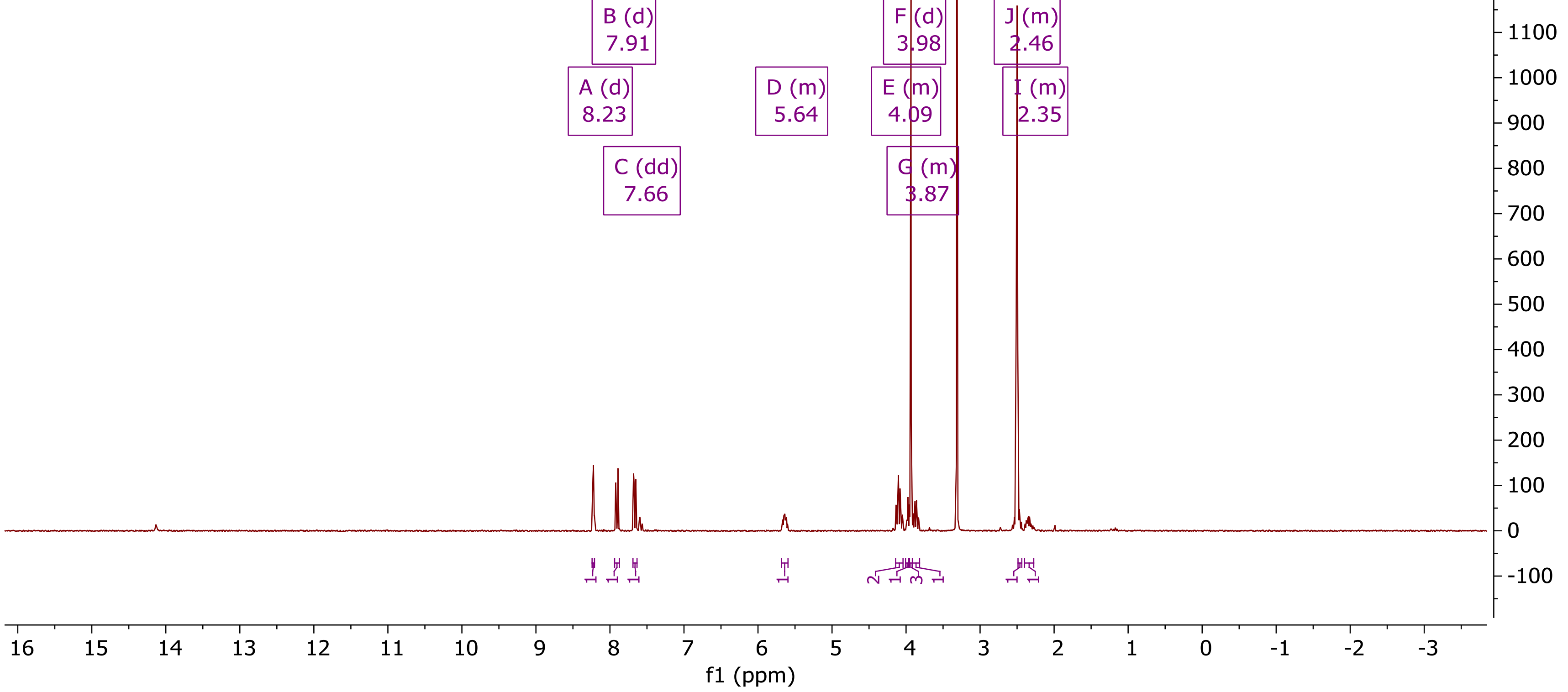

15j-^{1}H

LCJ-007-147B C13.10.fid
LCJ-007-147B C13

^{13}C NMR (75 MHz, DMSO δ) 161.8, 138.9, 133.2, 129.6, 124.5, 123.3, 116.2, 113.0, 71.6, 67.4, 59.0, 51.9, 32.1.

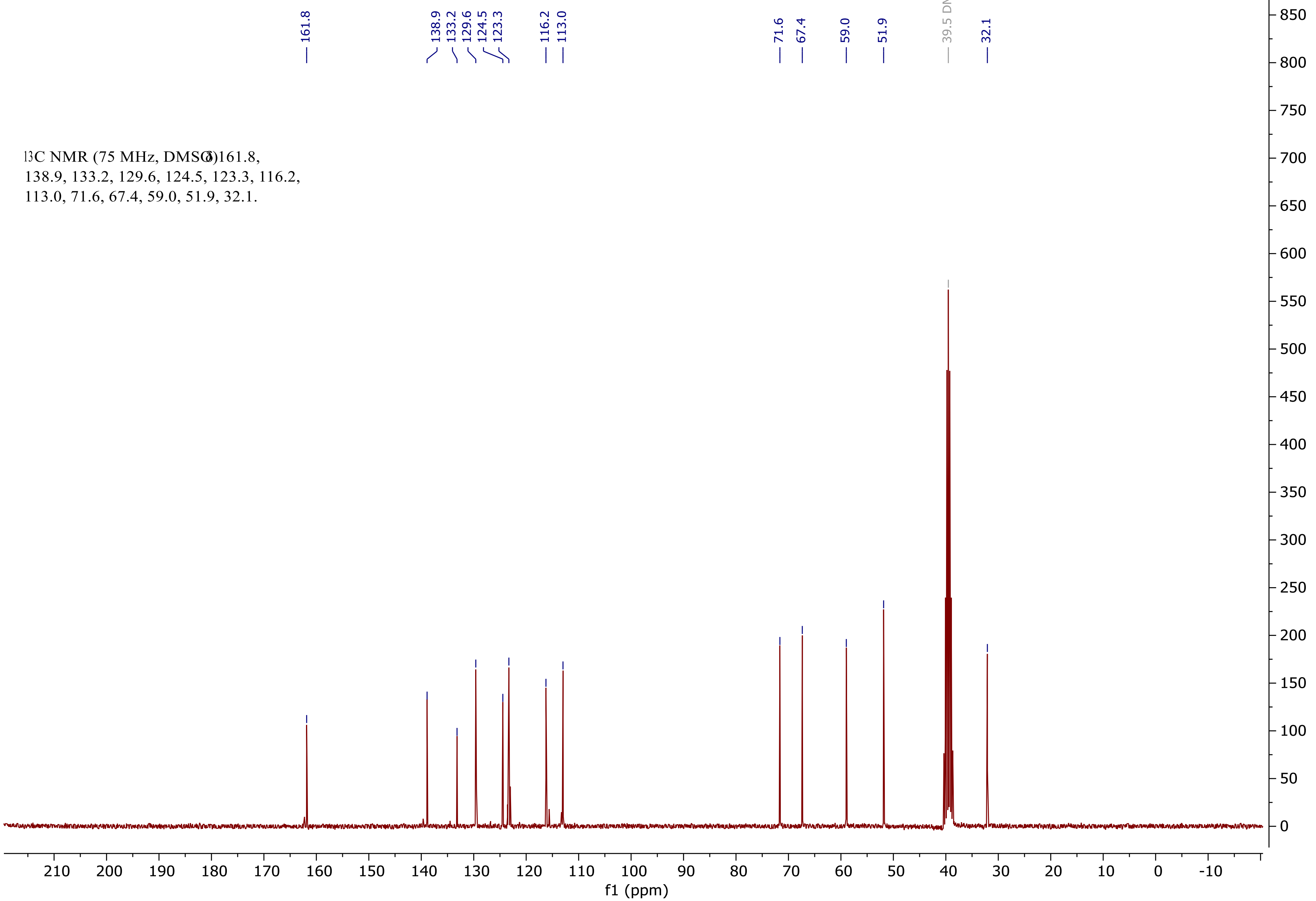

15j-^{13}C

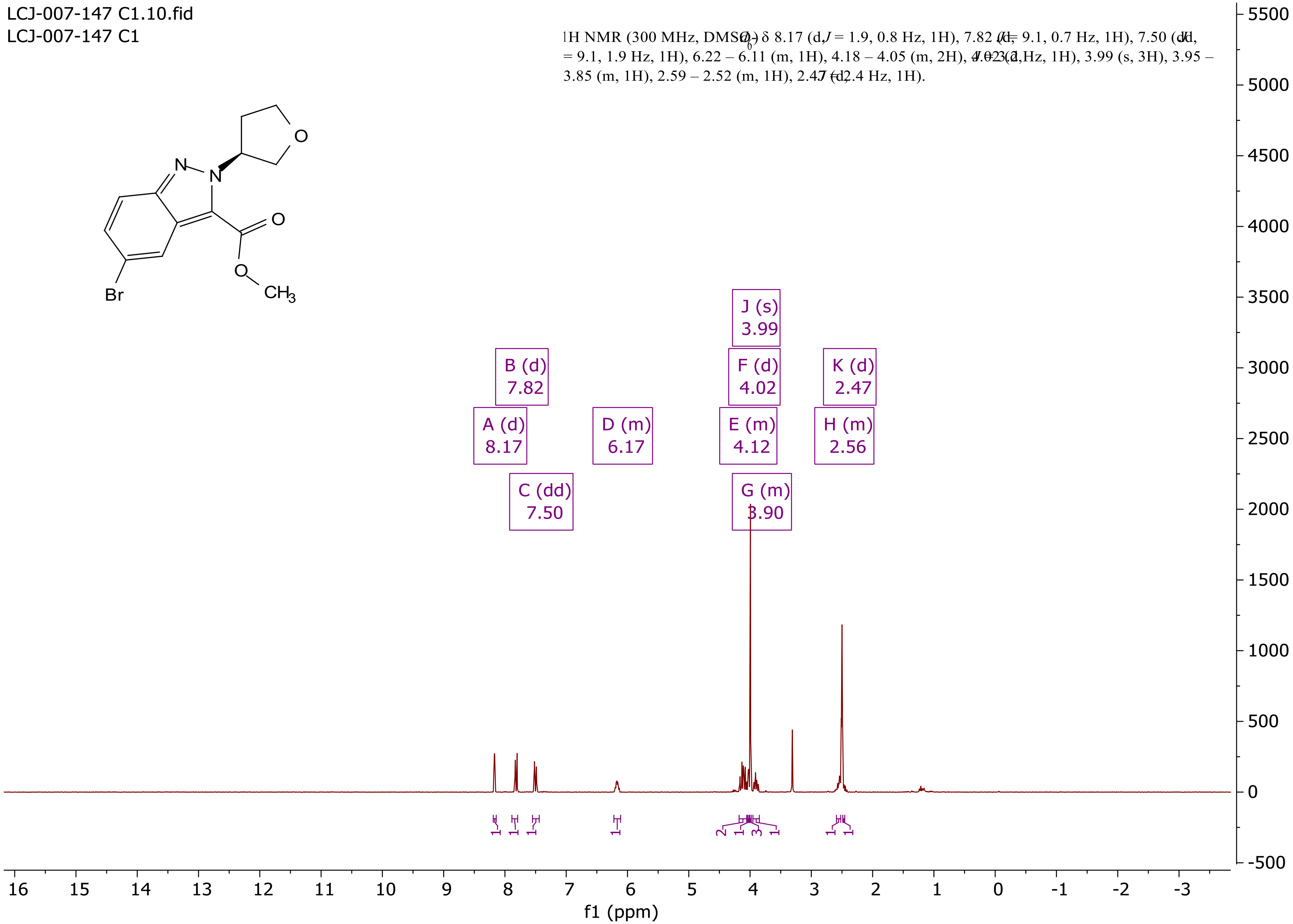
LCJ-007-147 C1.10.fid
LCJ-007-147 C1
1H NMR (300 MHz, DMSO-d_6) δ 8.17 (d, J = 1.9, 0.8 Hz, 1H), 7.82 (d, J = 9.1, 0.7 Hz, 1H), 7.50 (dd, J = 9.1, 1.9 Hz, 1H), 6.22 – 6.11 (m, 1H), 4.18 – 4.05 (m, 2H), 4.02 (d, J = 3.1 Hz, 1H), 3.99 (s, 3H), 3.95 – 3.85 (m, 1H), 2.59 – 2.52 (m, 1H), 2.47 (d, J = 2.4 Hz, 1H).
J (s) 3.99
B (d) 7.82
F (d) 4.02
K (d) 2.47
A (d) 8.17
D (m) 6.17
E (m) 4.12
H (m) 2.56
C (dd) 7.50
G (m) 3.90
f1 (ppm)

16j-^{1}H

LCJ-007-147C C13.10.fid
LCJ-007-147C C13

13C NMR (75 MHz, DMSO) δ 159.7, 144.8, 129.6, 124.1, 123.4, 123.1, 120.6, 118.3, 73.1, 67.5, 62.2, 52.4, 32.4.

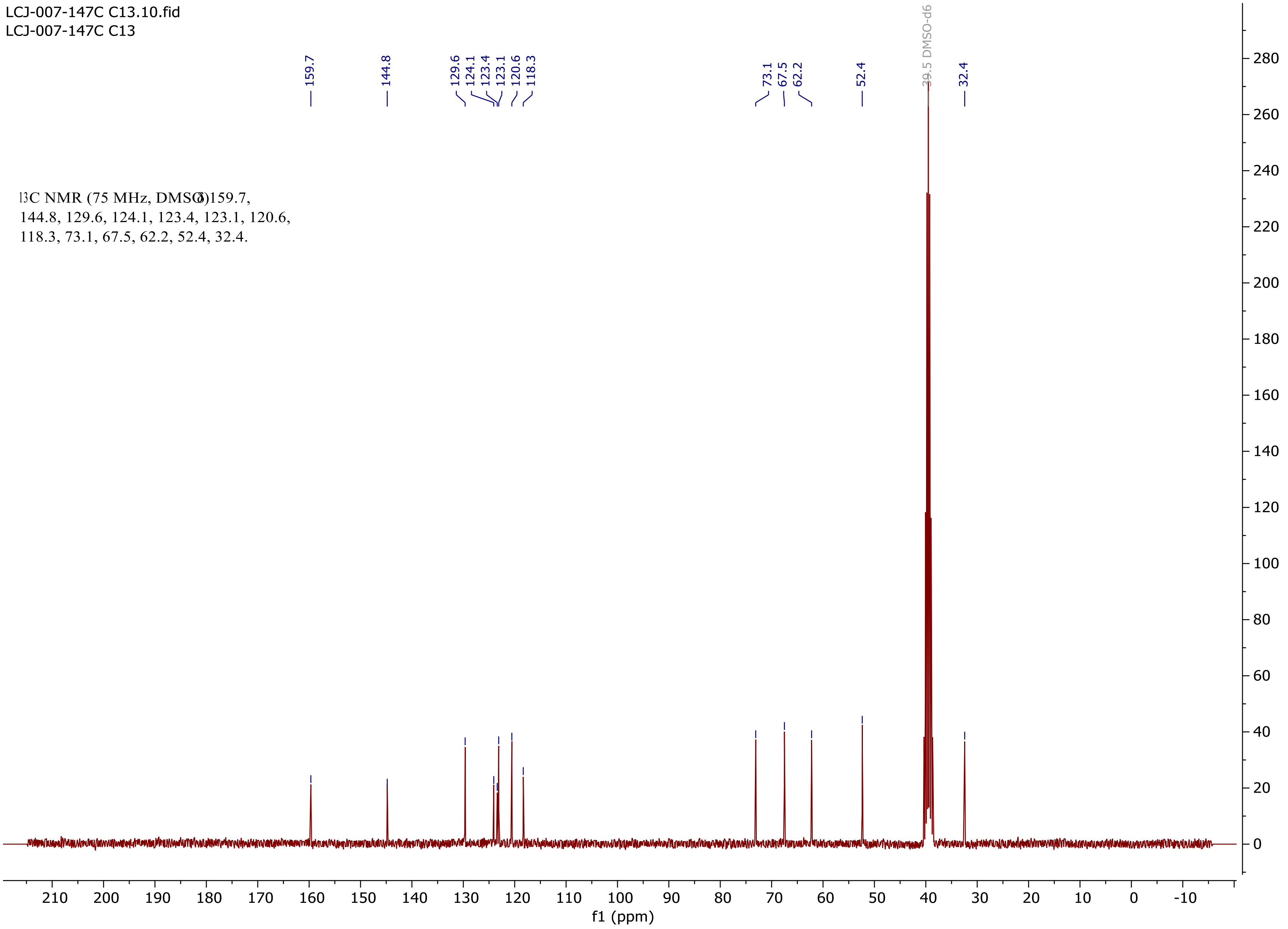

16j-^{13}C

LCJ-007-148 B2.10.fid
LCJ-007-148 B2

1H NMR (300 MHz, DMSO-d_6) δ 8.23 (d, J = 1.8 Hz, 1H), 7.92 (d, 1H), 7.67 (dd, J = 9.0, 1.9 Hz, 1H), 5.70 – 5.59 (m, 1H), 4.15 – 4.05 (m, 2H), 3.99 (d, J = 3.8 Hz, 1H), 3.94 (s, 3H), 3.88 (td, J = 8.1, 5.9 Hz, 1H), 2.56 (d, J = 7.6 Hz, 1H), 2.41 – 2.25 (m, 1H).

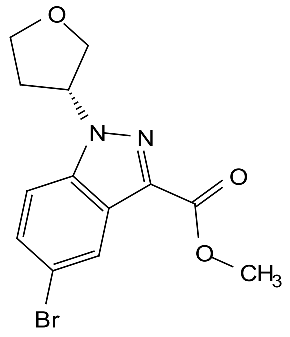

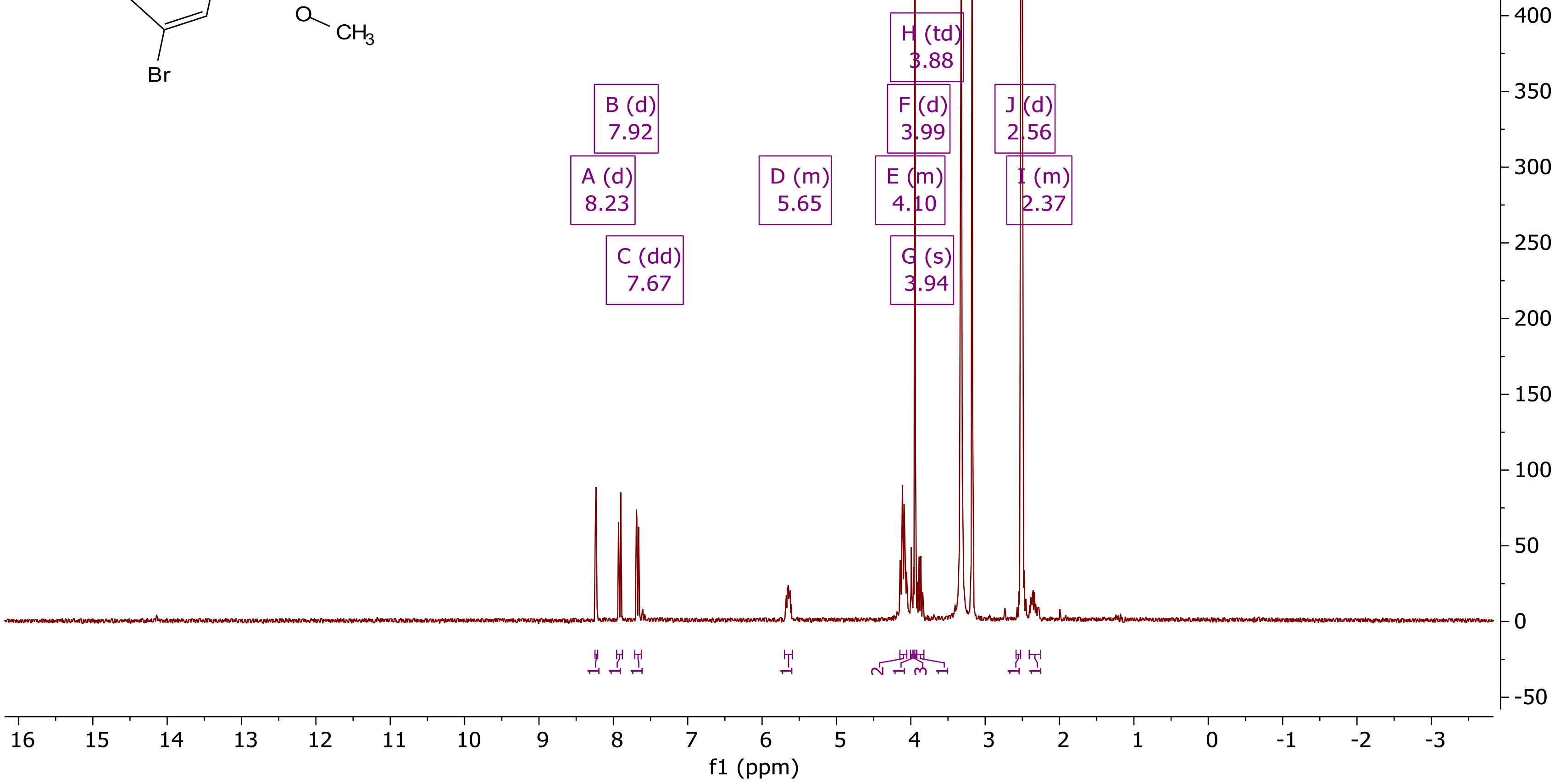

15k-^{1}H

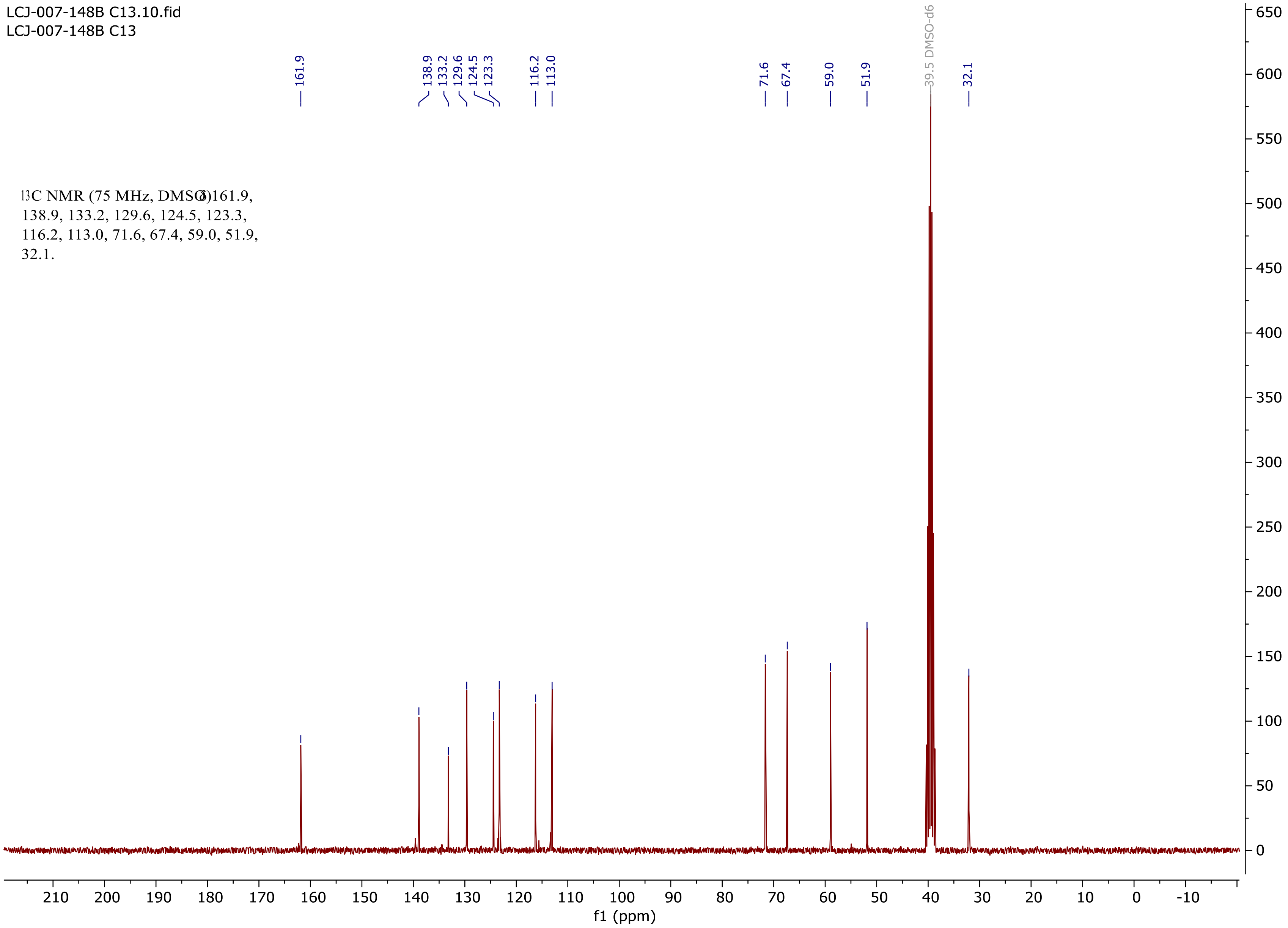

^{13}C NMR (75 MHz, DMSO) δ 161.9, 138.9, 133.2, 129.6, 124.5, 123.3, 116.2, 113.0, 71.6, 67.4, 59.0, 51.9, 32.1.

15k-^{13}C

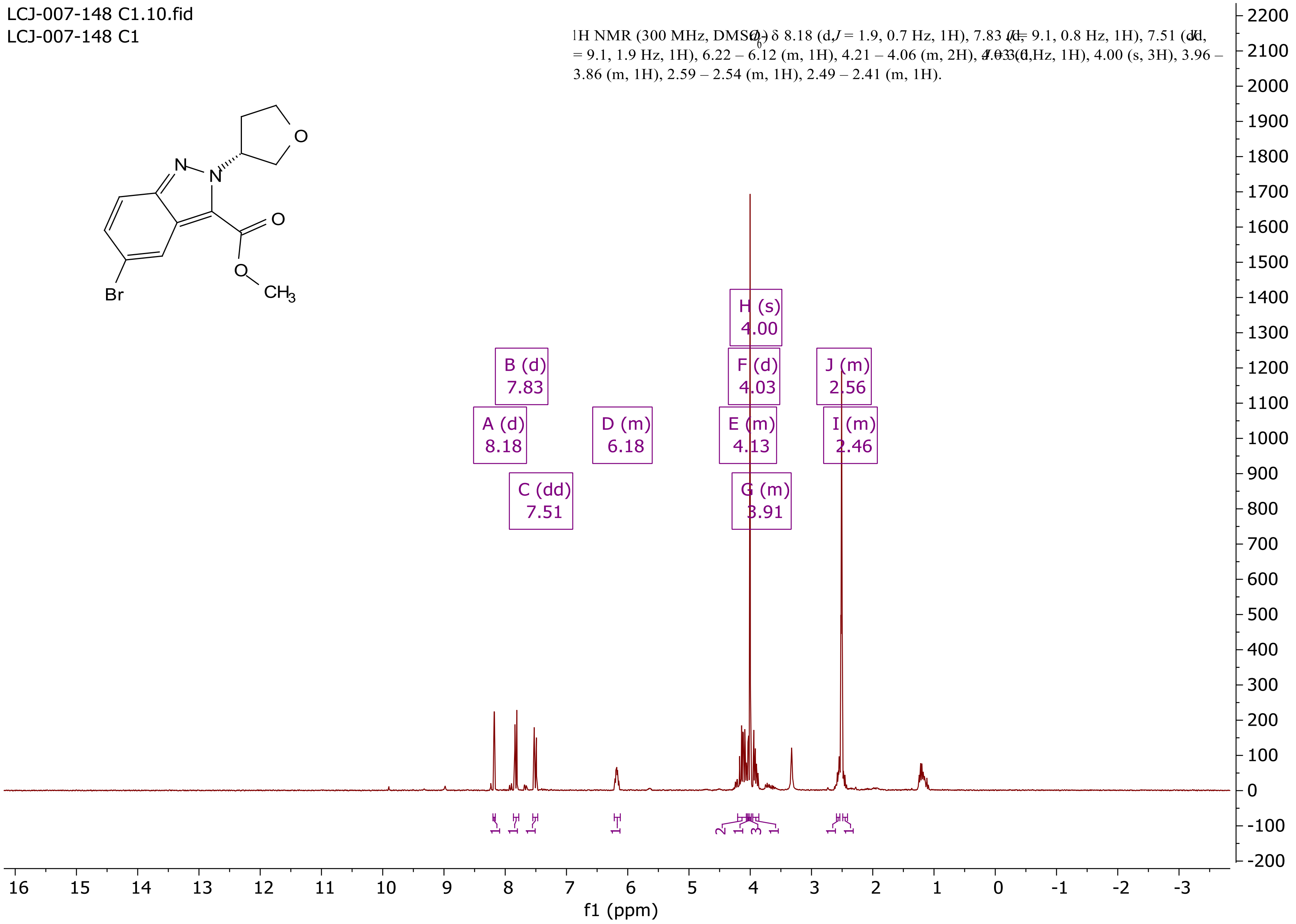

LCJ-007-148 C1.10.fid
LCJ-007-148 C1
1H NMR (300 MHz, DMSO-d6) δ 8.18 (d, J = 1.9, 0.7 Hz, 1H), 7.83 (dd, J = 9.1, 0.8 Hz, 1H), 7.51 (dd, J = 9.1, 1.9 Hz, 1H), 6.22 – 6.12 (m, 1H), 4.21 – 4.06 (m, 2H), 4.03 (d, J = [illegible] Hz, 1H), 4.00 (s, 3H), 3.96 – 3.86 (m, 1H), 2.59 – 2.54 (m, 1H), 2.49 – 2.41 (m, 1H).
Br
N
N
O
O
O
CH3
A (d) 8.18
B (d) 7.83
C (dd) 7.51
D (m) 6.18
E (m) 4.13
F (d) 4.03
G (m) 3.91
H (s) 4.00
I (m) 2.46
J (m) 2.56
f1 (ppm)

16k-^{1}H

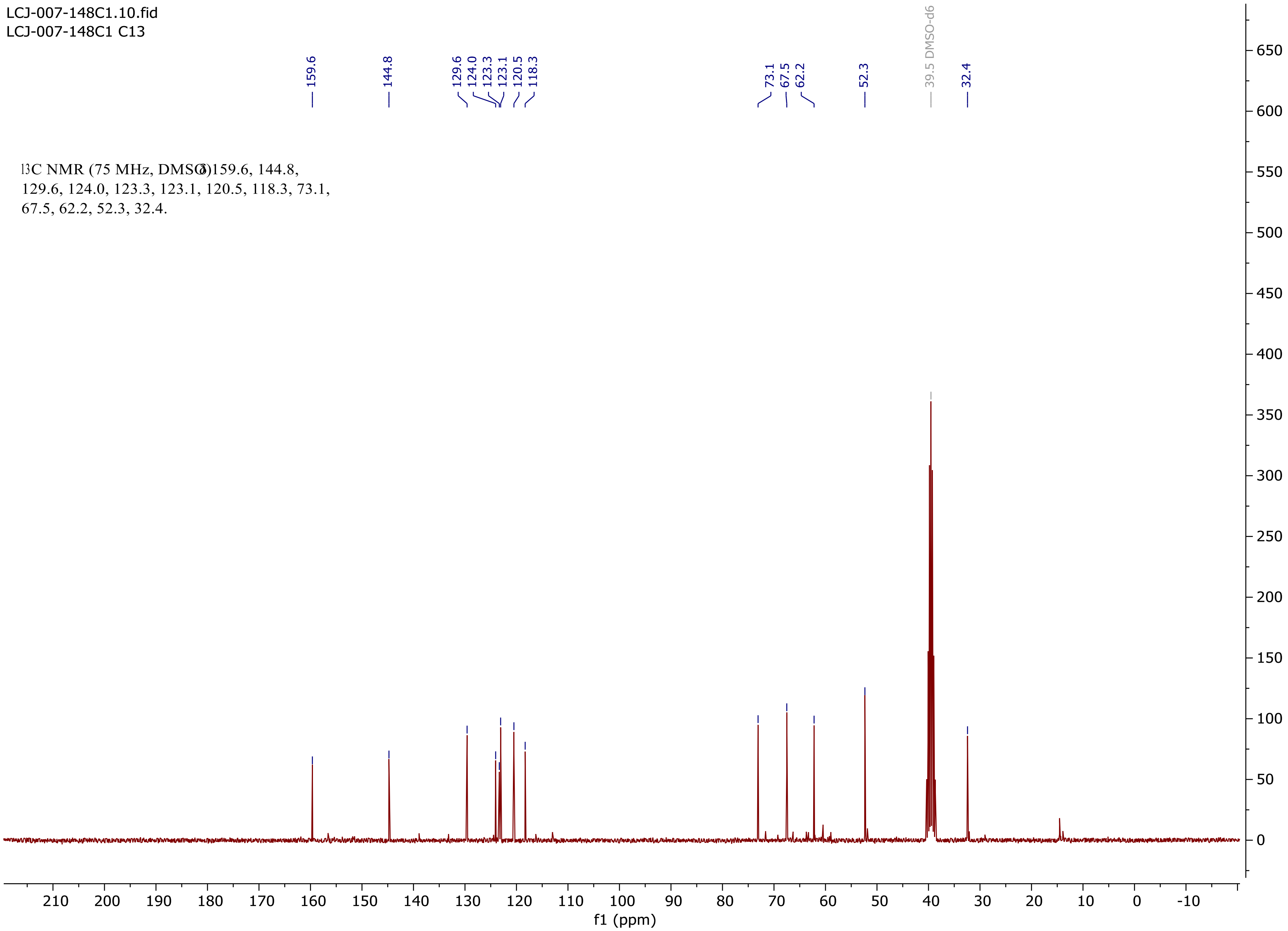
LCJ-007-148C1.10.fid
LCJ-007-148C1 C13
159.6
144.8
129.6
124.0
123.3
123.1
120.5
118.3
73.1
67.5
62.2
52.3
39.5 DMSO-d6
32.4
13C NMR (75 MHz, DMSO δ) 159.6, 144.8,
129.6, 124.0, 123.3, 123.1, 120.5, 118.3, 73.1,
67.5, 62.2, 52.3, 32.4.
f1 (ppm)

16k-^{13}C

LCJ-007-142 B2.10.fid
LCJ-007-142 B2

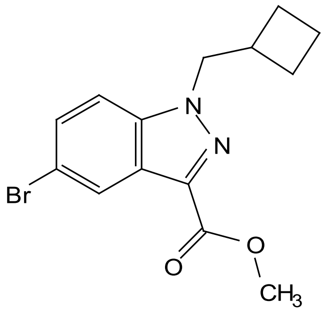

1H NMR (300 MHz, DMSO-d_6) δ 8.20 (d, J = 1.9, 0.7 Hz, 1H), 7.90 (d, J = 9.0, 0.7 Hz, 1H), 7.63 (dd, J = 9.0, 1.9 Hz, 1H), 4.56 (d, J = 7.2 Hz, 2H), 3.93 (s, 3H), 2.85 (p, J = 7.4 Hz, 1H), 1.99 – 1.87 (m, 2H), 1.87 – 1.73 (m, 4H).

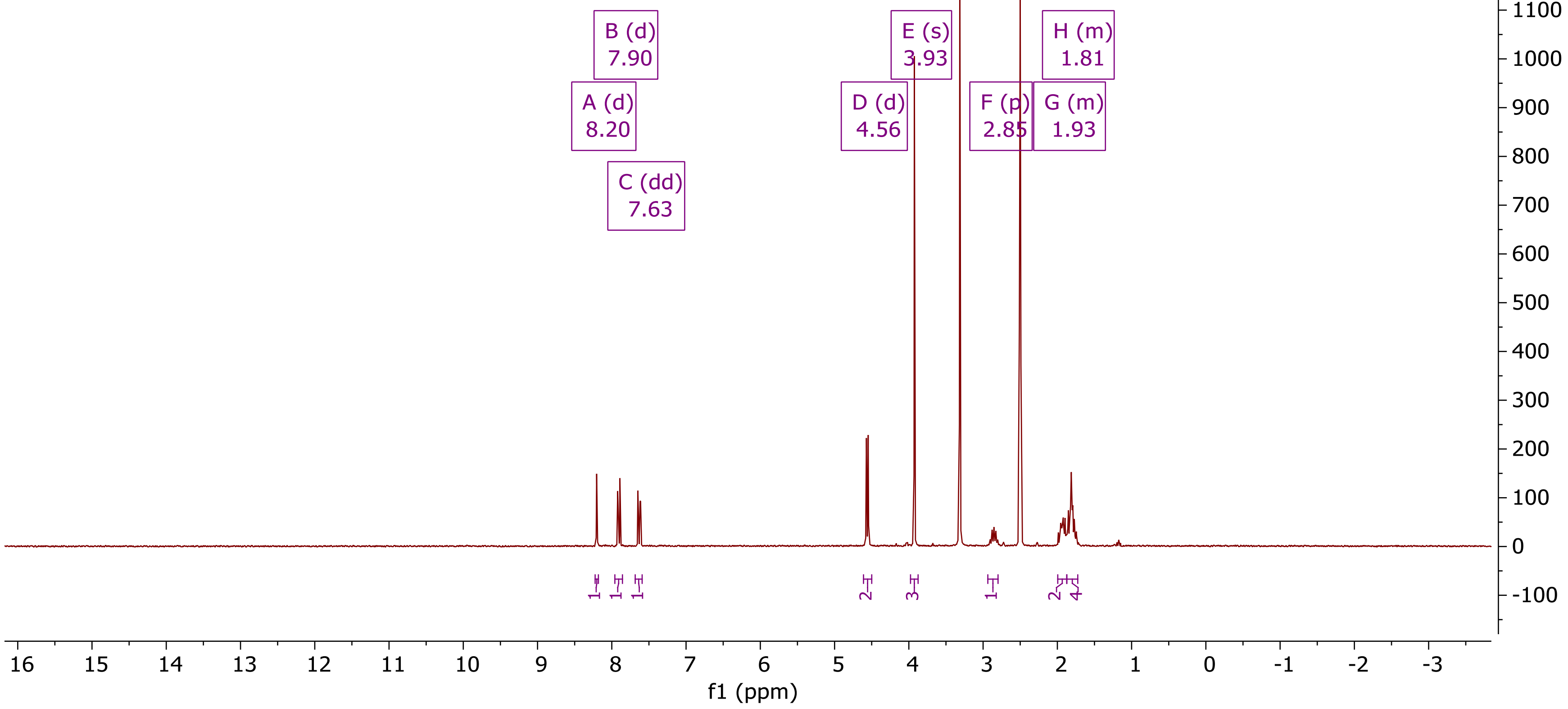

15l-^{1}H

13C NMR (75 MHz, DMSO) δ 162.0, 139.4, 133.1, 129.5, 124.1, 123.2, 116.0, 113.1, 53.8, 51.8, 35.2, 25.3, 17.7.

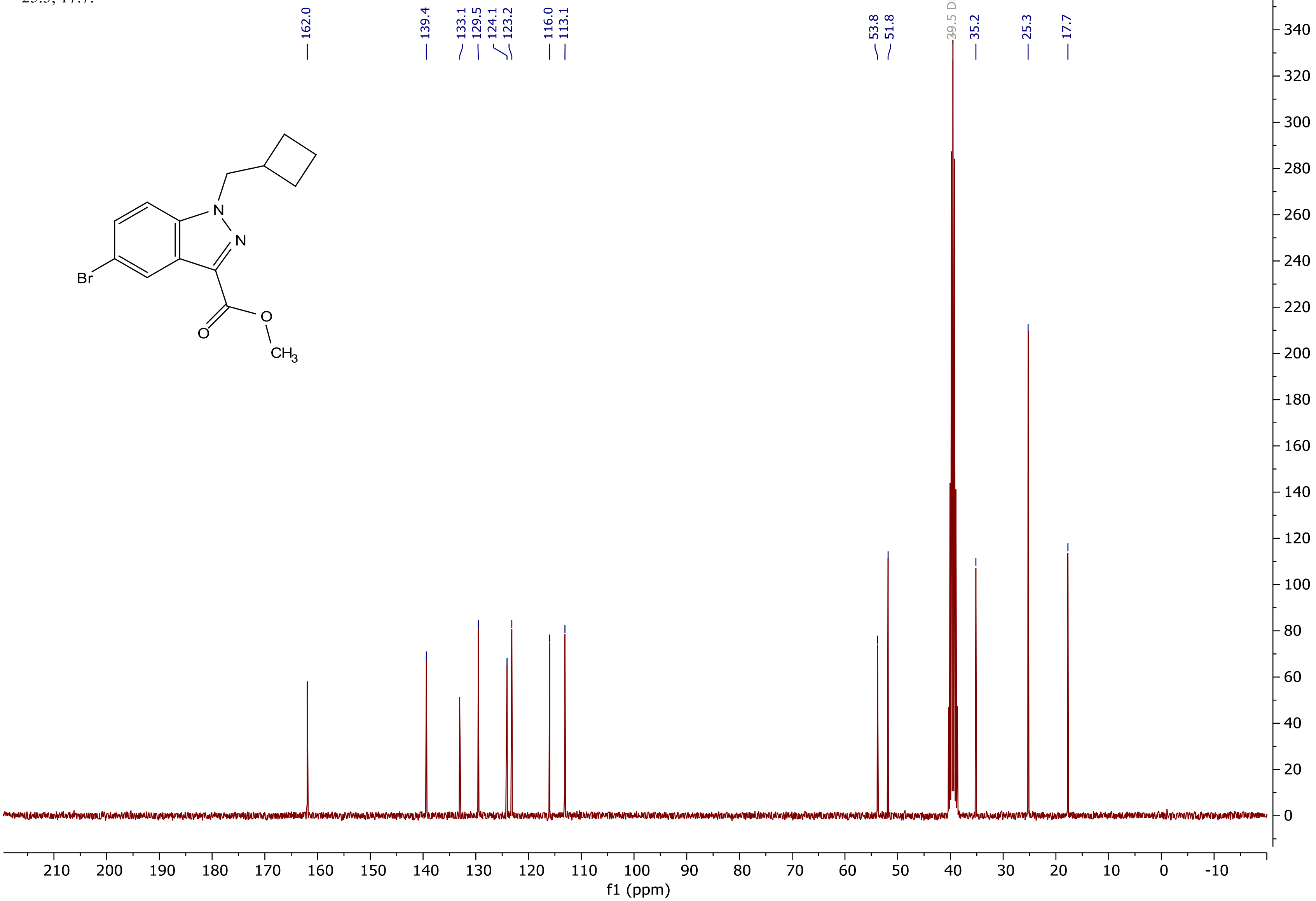

15l-^{13}C

LCJ-007-142 C1.10.fid
LCJ-007-142 C1

1H NMR (300 MHz, DMSO-d_6) δ 8.16 (d, J = 1.9, 0.8 Hz, 1H), 7.79 (d, J = 9.0, 0.7 Hz, 1H), 7.49 (dd, J = 9.1, 1.9 Hz, 1H), 4.88 (d, J = 7.3 Hz, 2H), 3.99 (s, 3H), 2.90 (p, J = 7.4 Hz, 1H), 1.98 – 1.90 (m, 2H), 1.88 – 1.78 (m, 4H).

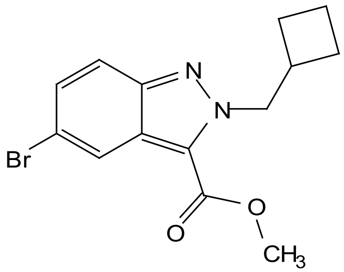

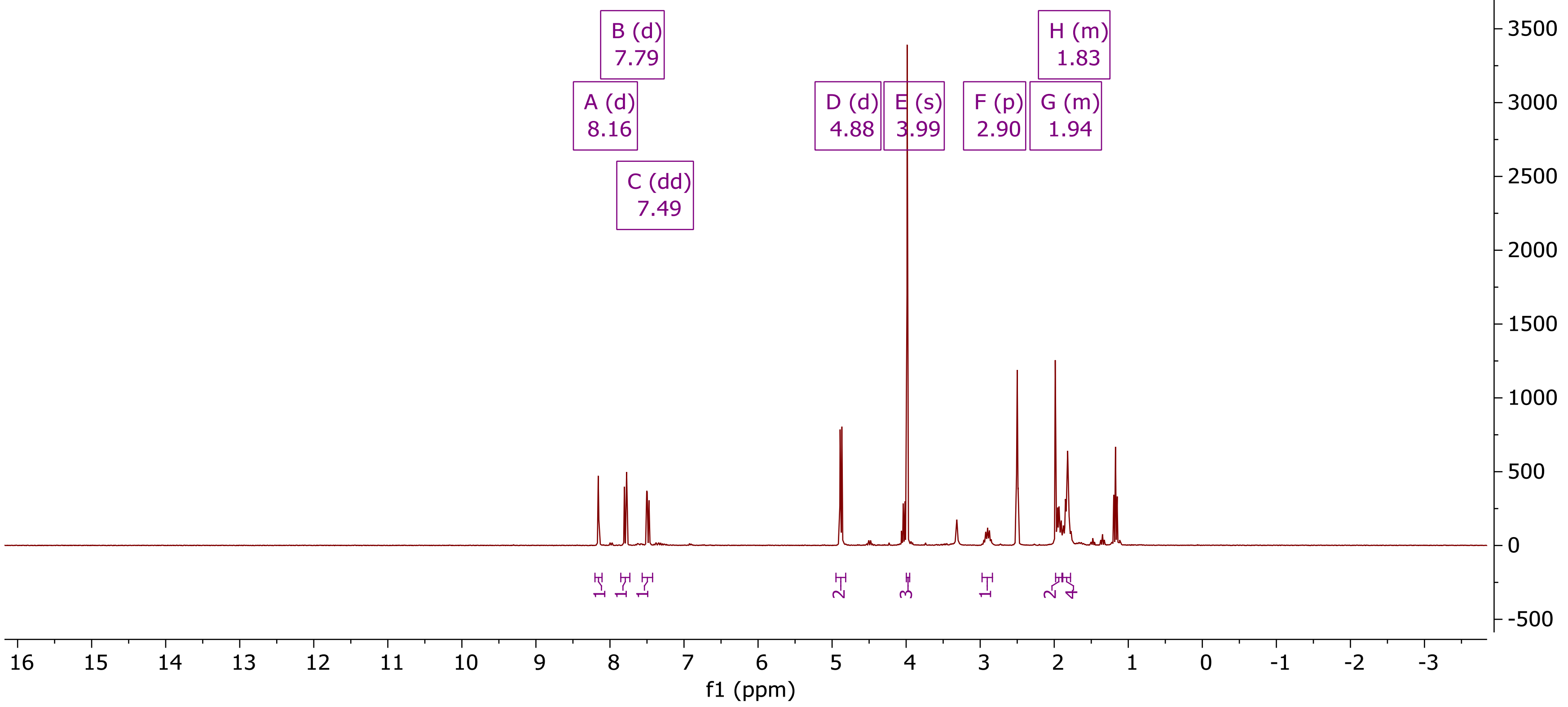

16l-^{1}H

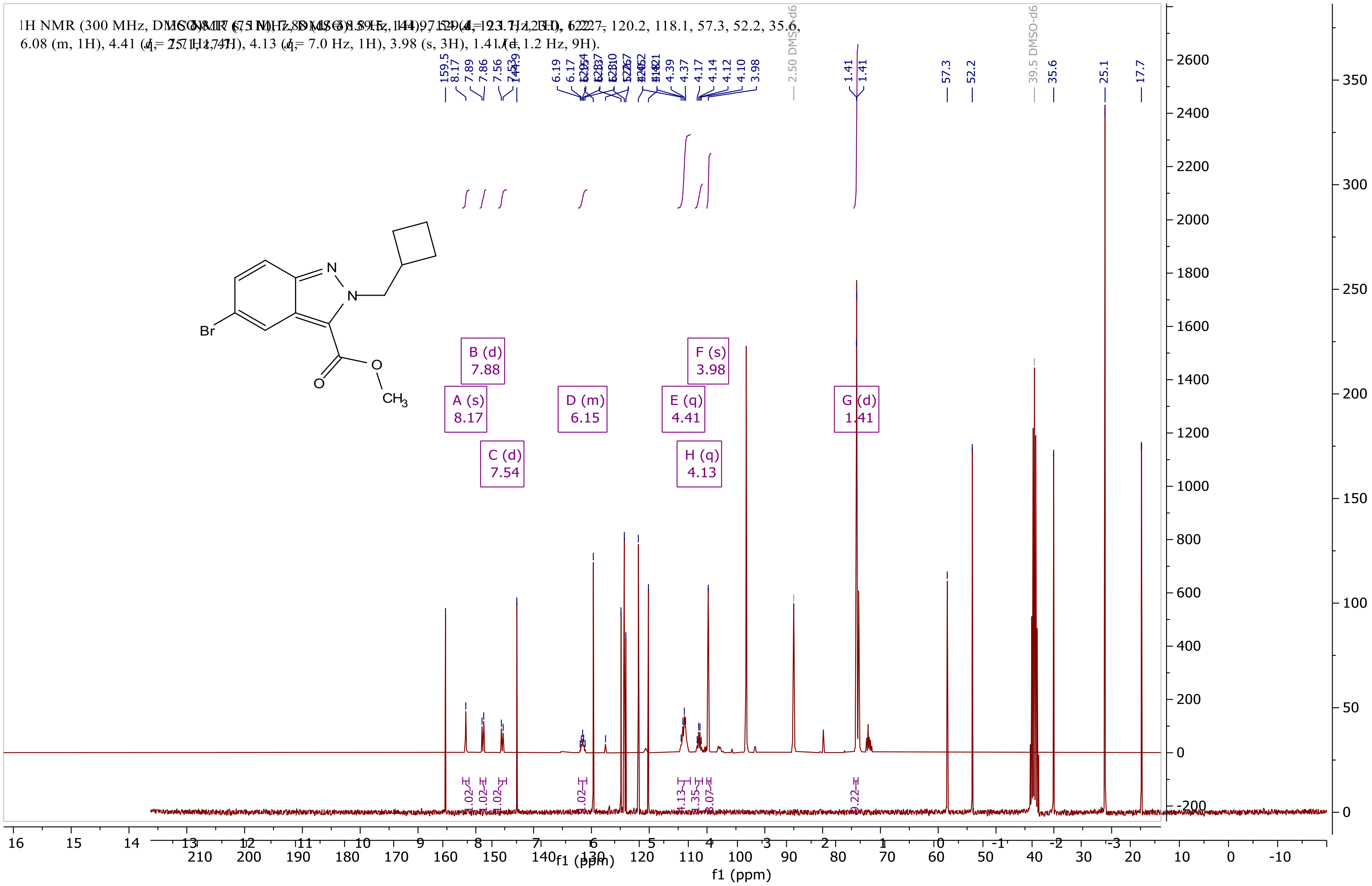

16l-^{13}C

16m-^{1}H

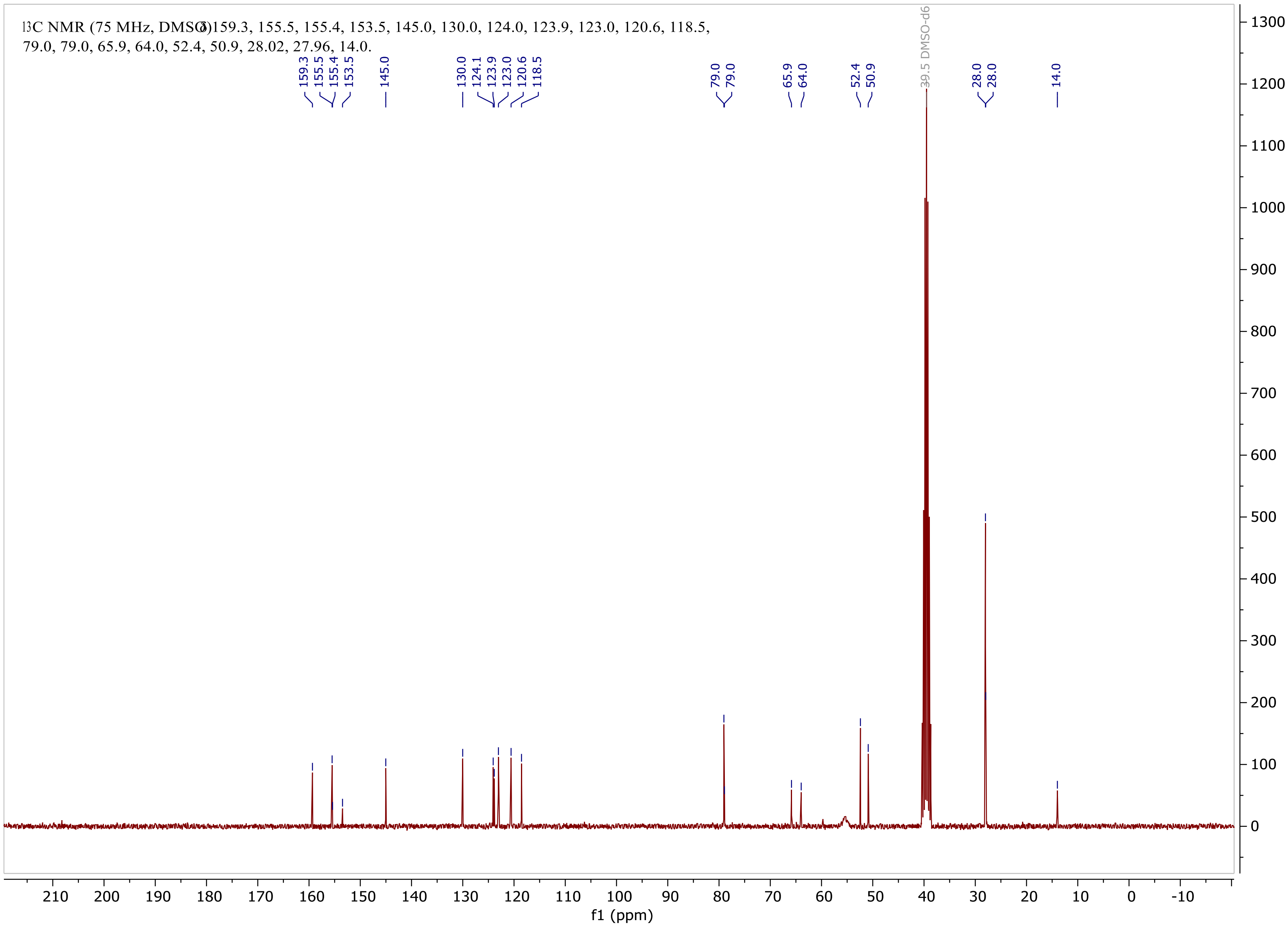
13C NMR (75 MHz, DMSO) δ 159.3, 155.5, 155.4, 153.5, 145.0, 130.0, 124.0, 123.9, 123.0, 120.6, 118.5,
79.0, 79.0, 65.9, 64.0, 52.4, 50.9, 28.02, 27.96, 14.0.
39.5 DMSO-d6
f1 (ppm)

16m-^{13}C

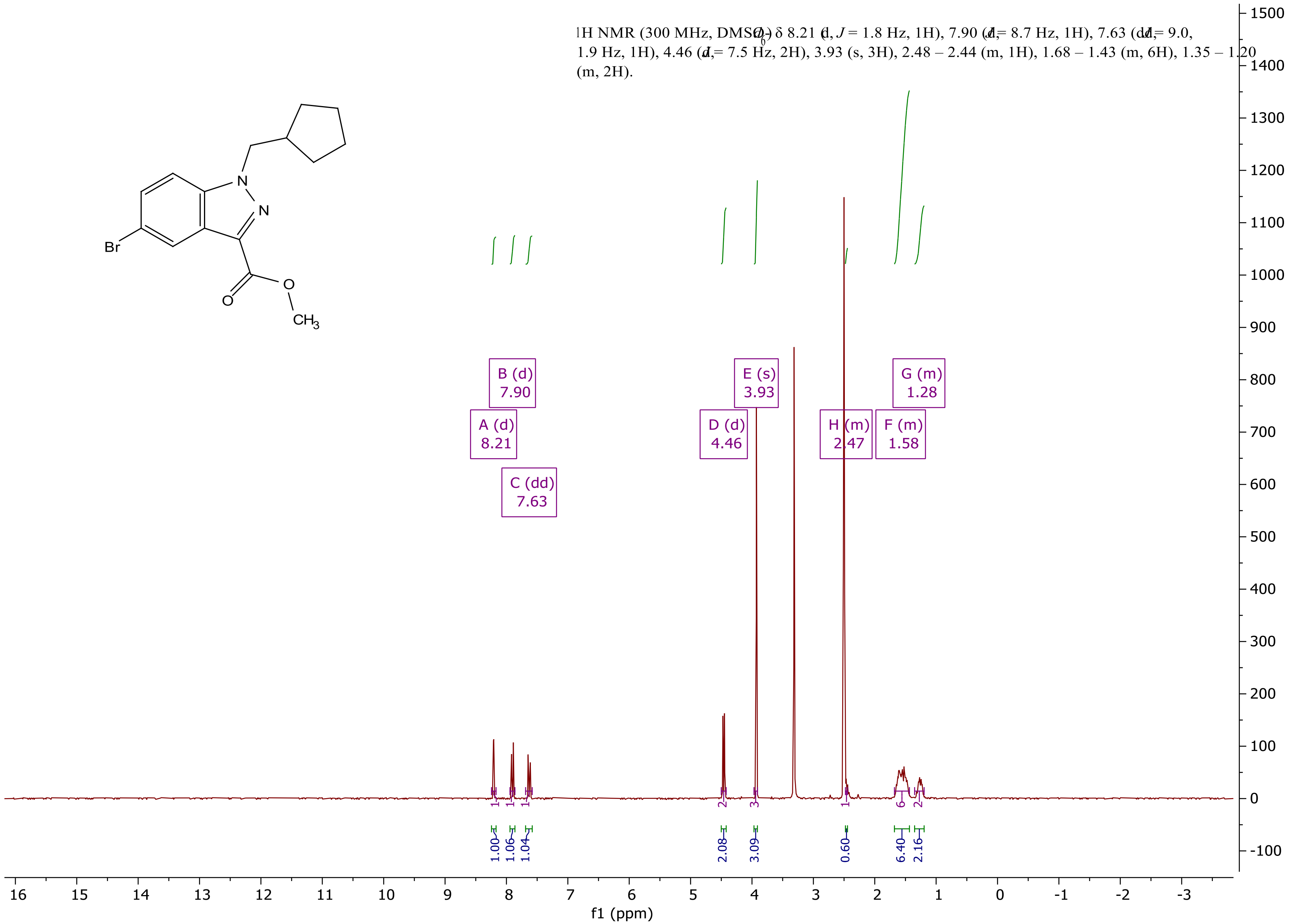
1H NMR (300 MHz, DMSO-d_6) δ 8.21 (d, J = 1.8 Hz, 1H), 7.90 (d, J = 8.7 Hz, 1H), 7.63 (dd, J = 9.0, 1.9 Hz, 1H), 4.46 (d, J = 7.5 Hz, 2H), 3.93 (s, 3H), 2.48 – 2.44 (m, 1H), 1.68 – 1.43 (m, 6H), 1.35 – 1.20 (m, 2H).
Br
N
N
O
O
CH3
A (d) 8.21
B (d) 7.90
C (dd) 7.63
D (d) 4.46
E (s) 3.93
H (m) 2.47
F (m) 1.58
G (m) 1.28
1.00
1.06
1.04
2.08
3.09
0.60
6.40
2.16
f1 (ppm)

15n-1H

^{13}C NMR (75 MHz, DMSO) δ 162.0, 139.3, 133.0, 129.5, 124.1, 123.2, 116.0, 113.1, 53.6, 51.8, 29.6, 24.4.

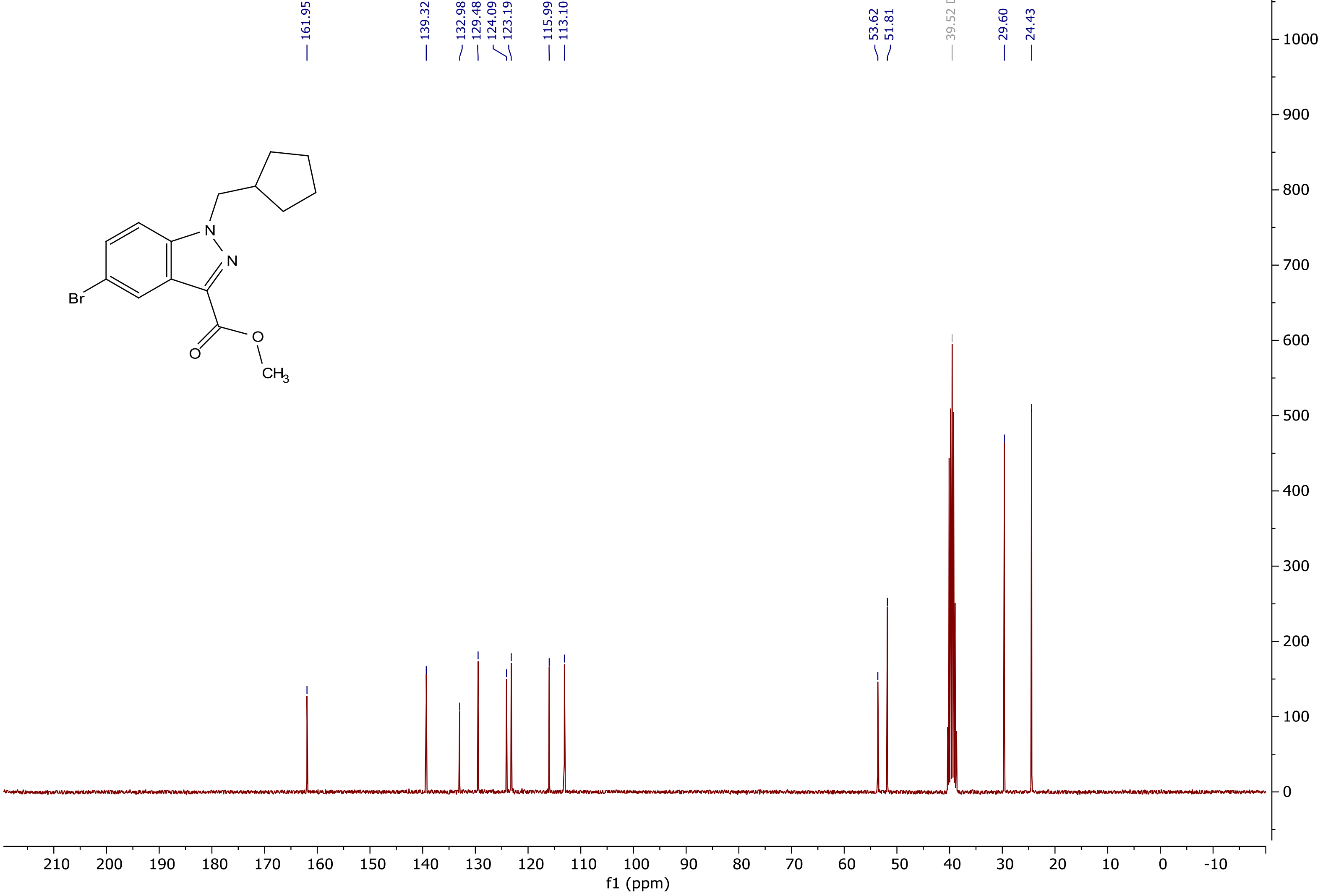

15n-^{13}C

1H NMR (300 MHz, DMSO-d_6) δ 8.17 (d, J = 1.9 Hz, 1H), 7.80 (d, J = 9.1 Hz, 1H), 7.50 (dd, J = 9.1, 1.9 Hz, 1H), 4.78 (d, J = 7.6 Hz, 2H), 3.99 (s, 3H), 2.58 – 2.52 (m, 1H), 1.67 – 1.45 (m, 6H), 1.39 – 1.24 (m, 2H).

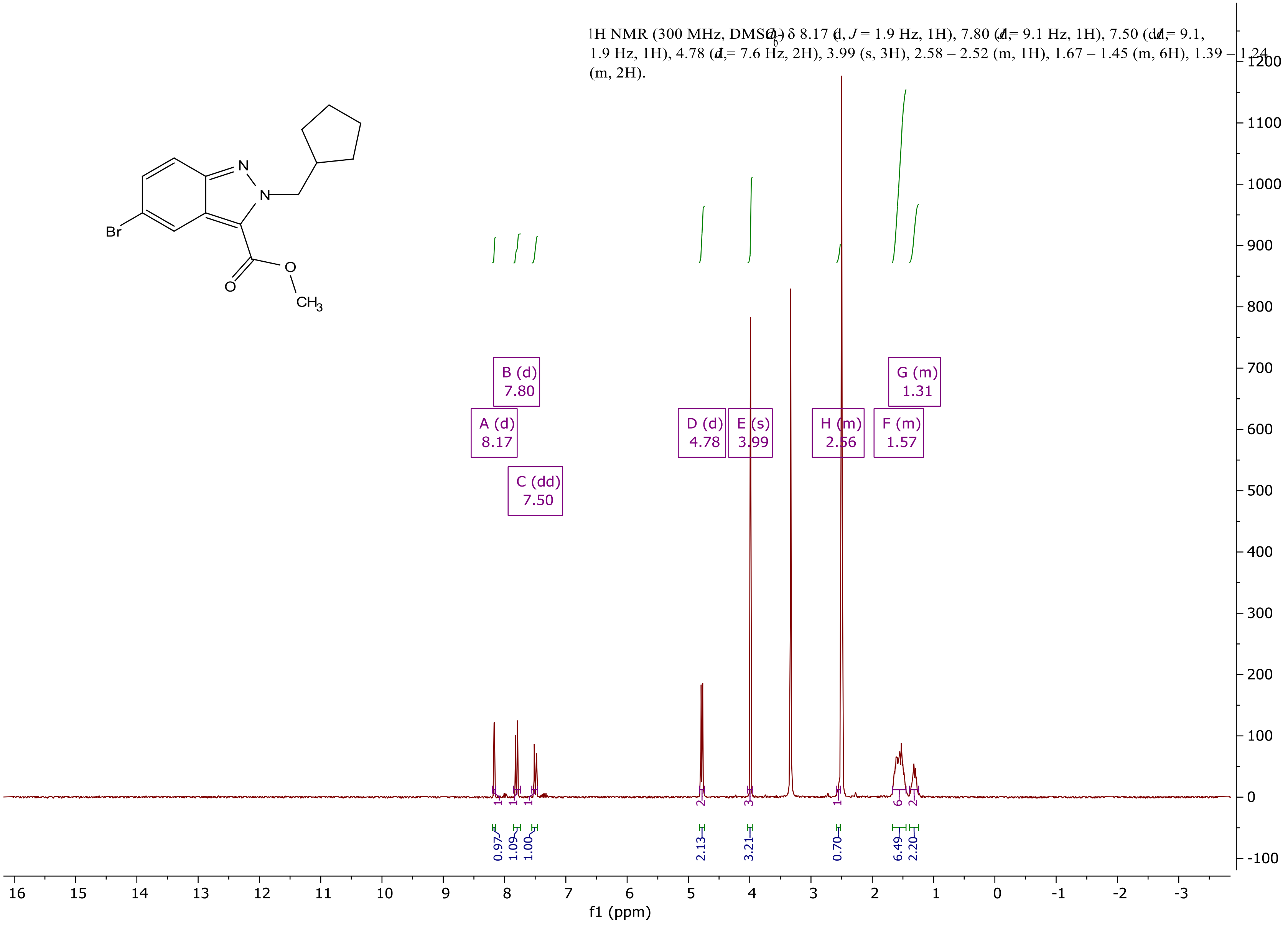

16n-^{1}H

13C NMR (75 MHz, DMSO δ) 159.6, 144.9, 129.5, 123.9, 123.2, 122.9, 120.4, 118.2, 57.1, 52.3, 29.4, 24.4.

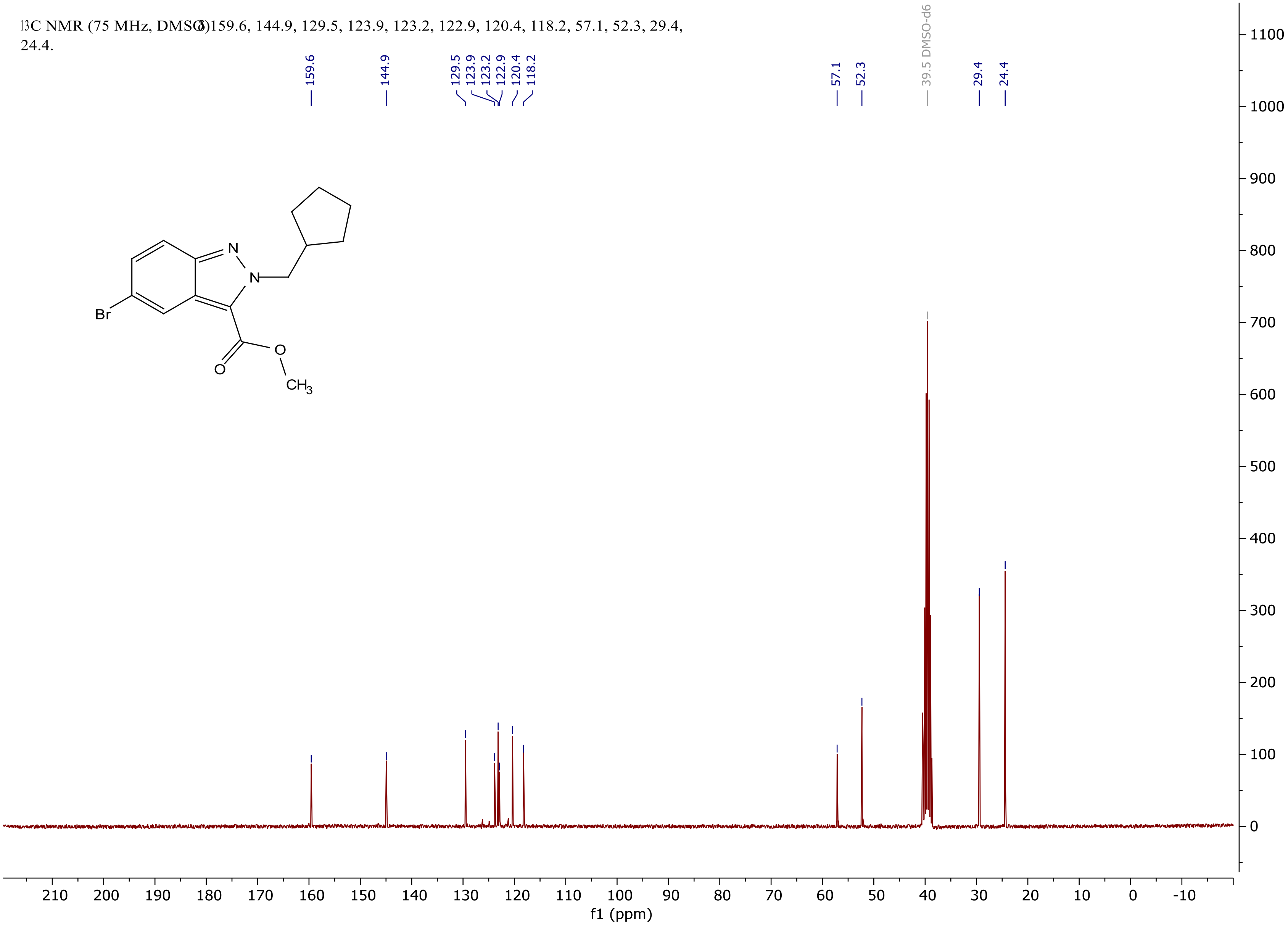

16n-^{13}C

1H NMR (300 MHz, DMSO-d_6) δ 8.23 (d, 1H), 7.93 (d, J = 9.0, 0.7 Hz, 1H), 7.66 (dd, J = 9.0, 1.9 Hz, 1H), 4.54 (d, J = 7.5 Hz, 2H), 3.94 (s, 3H), 3.88 – 3.74 (m, 1H), 3.71 – 3.57 (m, 2H), 3.48 (dd, J = 7.[illegible], 5.6 Hz, 1H), 2.83 (p, J = 6.6 Hz, 1H), 1.98 – 1.81 (m, 1H), 1.72 – 1.55 (m, 1H).

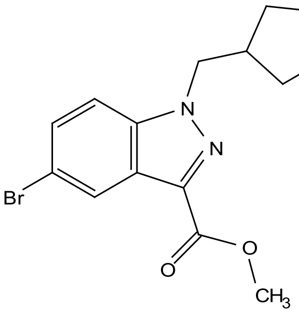

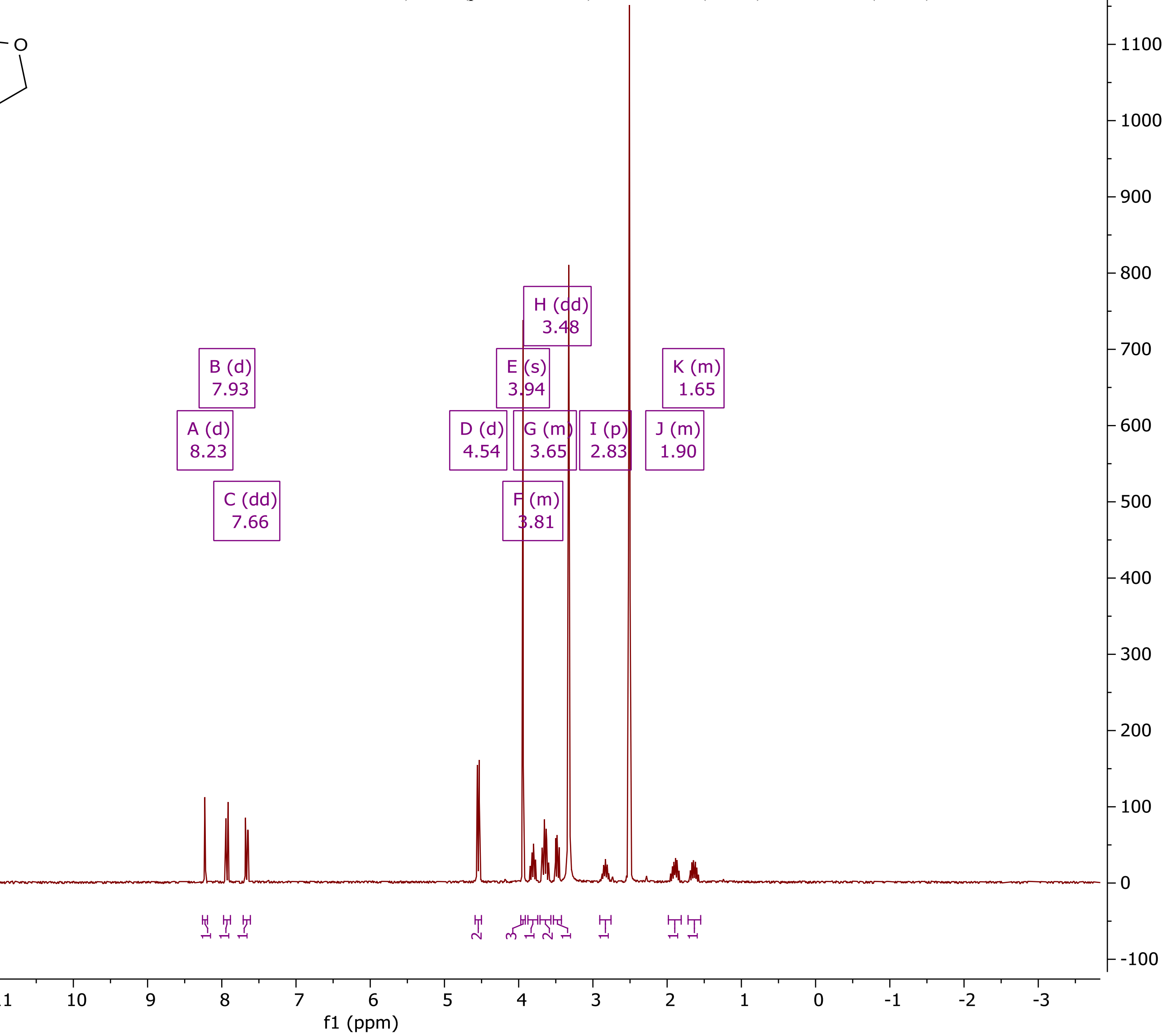

15o-^{1}H

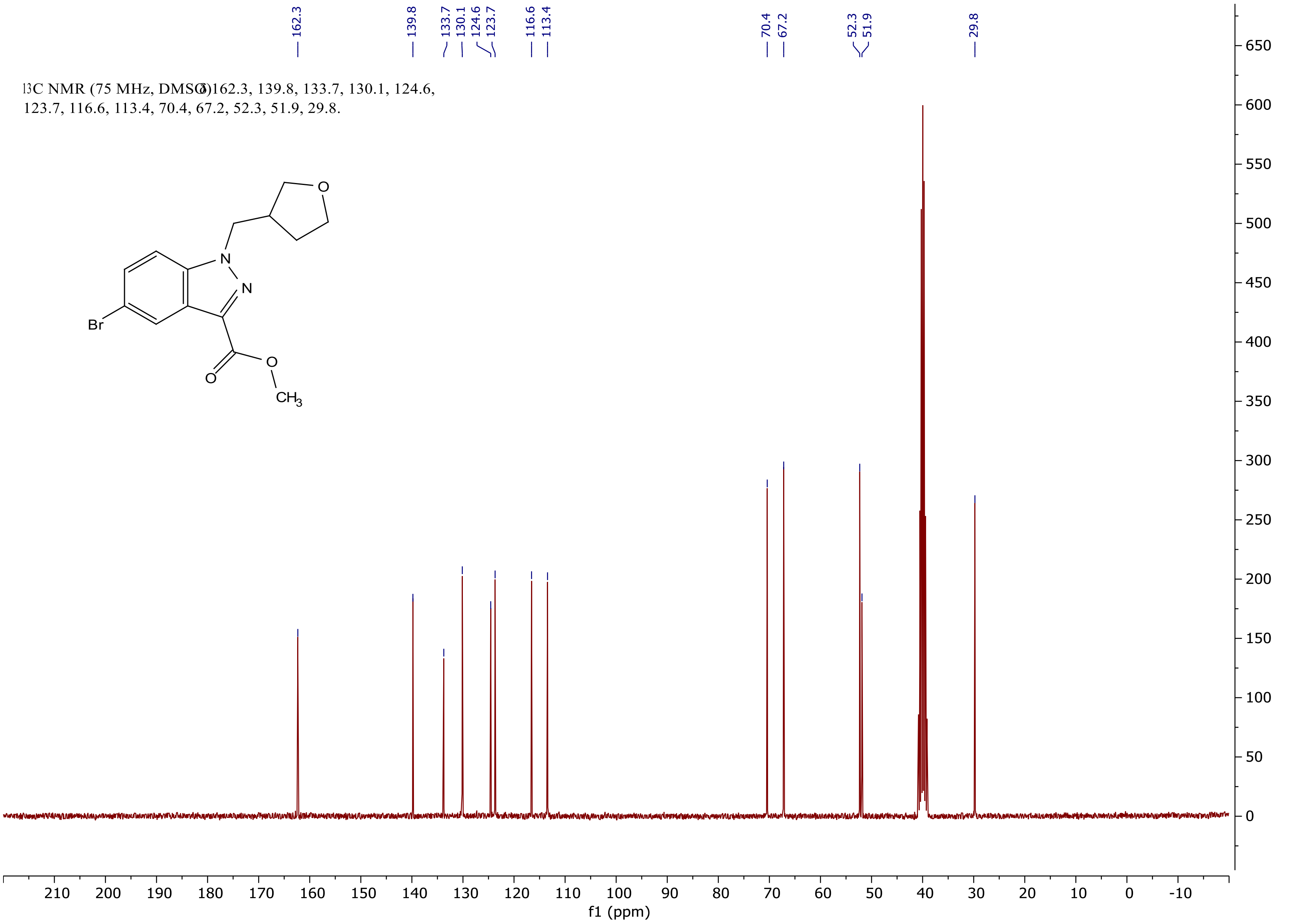

162.3
139.8
133.7
130.1
124.6
123.7
116.6
113.4
70.4
67.2
52.3
51.9
29.8
13C NMR (75 MHz, DMSO) δ 162.3, 139.8, 133.7, 130.1, 124.6,
123.7, 116.6, 113.4, 70.4, 67.2, 52.3, 51.9, 29.8.
O
N
N
Br
O
O
CH3
f1 (ppm)

15o-^{13}C

1H NMR (300 MHz, DMSO-d_6) δ 8.18 (d, 1H), 7.82 (d, J = 9.1, 0.8 Hz, 1H), 7.51 (dd, J = 9.1, 1.9 Hz, 1H), 4.93 – 4.73 (m, 2H), 4.00 (s, 3H), 3.81 (td, J = 8.1, 5.6 Hz, 1H), 3.69 – 3.60 (m, 2H), 3.60 – 3.52 (m, 1H), 2.91 (h, J = 13.2, 6.8, 6.3 Hz, 1H), 1.99 – 1.85 (m, 1H), 1.76 – 1.58 (m, 1H).

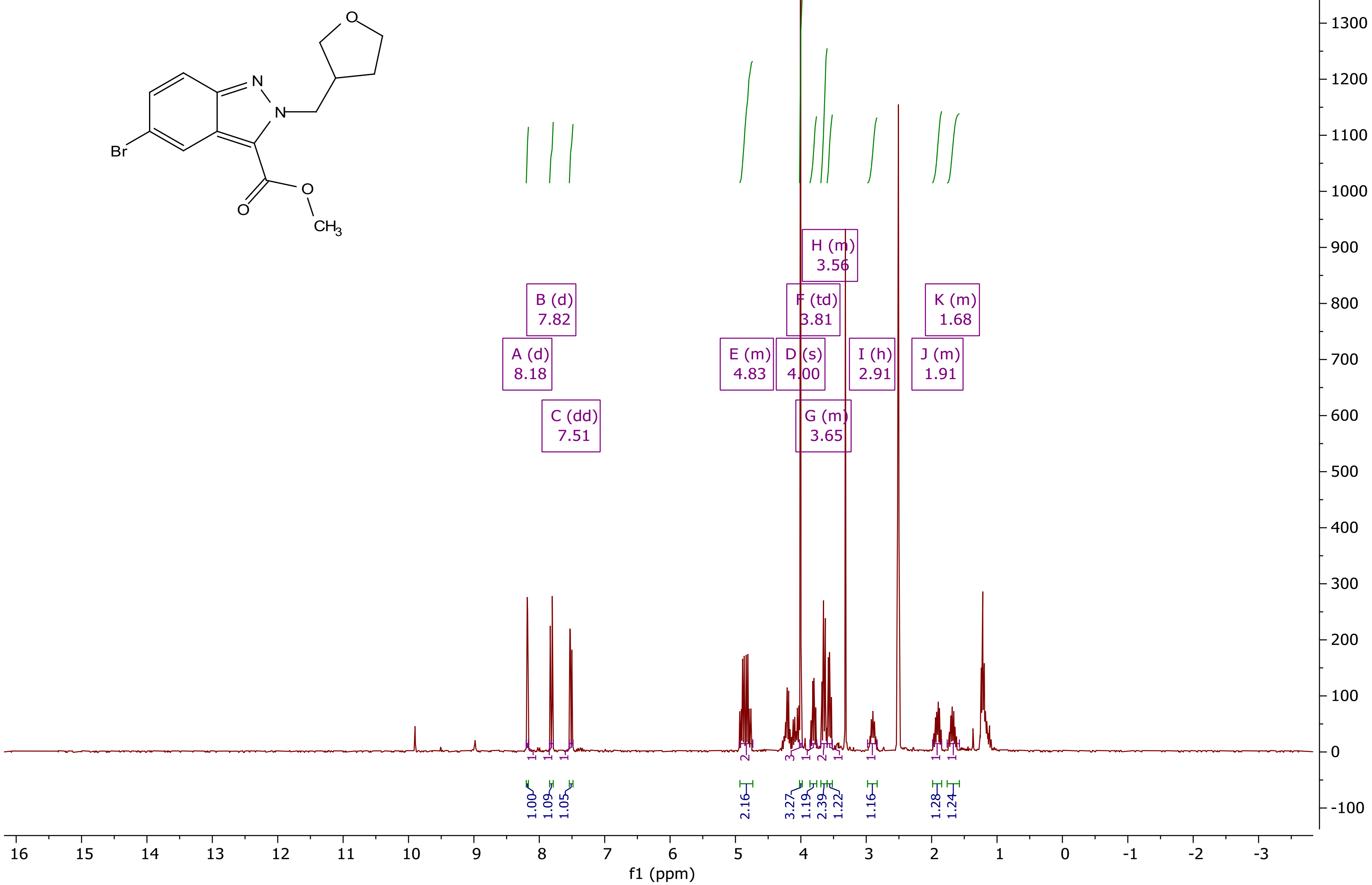

16o-^{1}H

13C NMR (75 MHz, DMSO δ) 159.5, 145.0, 129.7, 123.8, 123.2, 120.4, 118.3, 69.9, 66.7, 55.2, 52.4, 29.1.

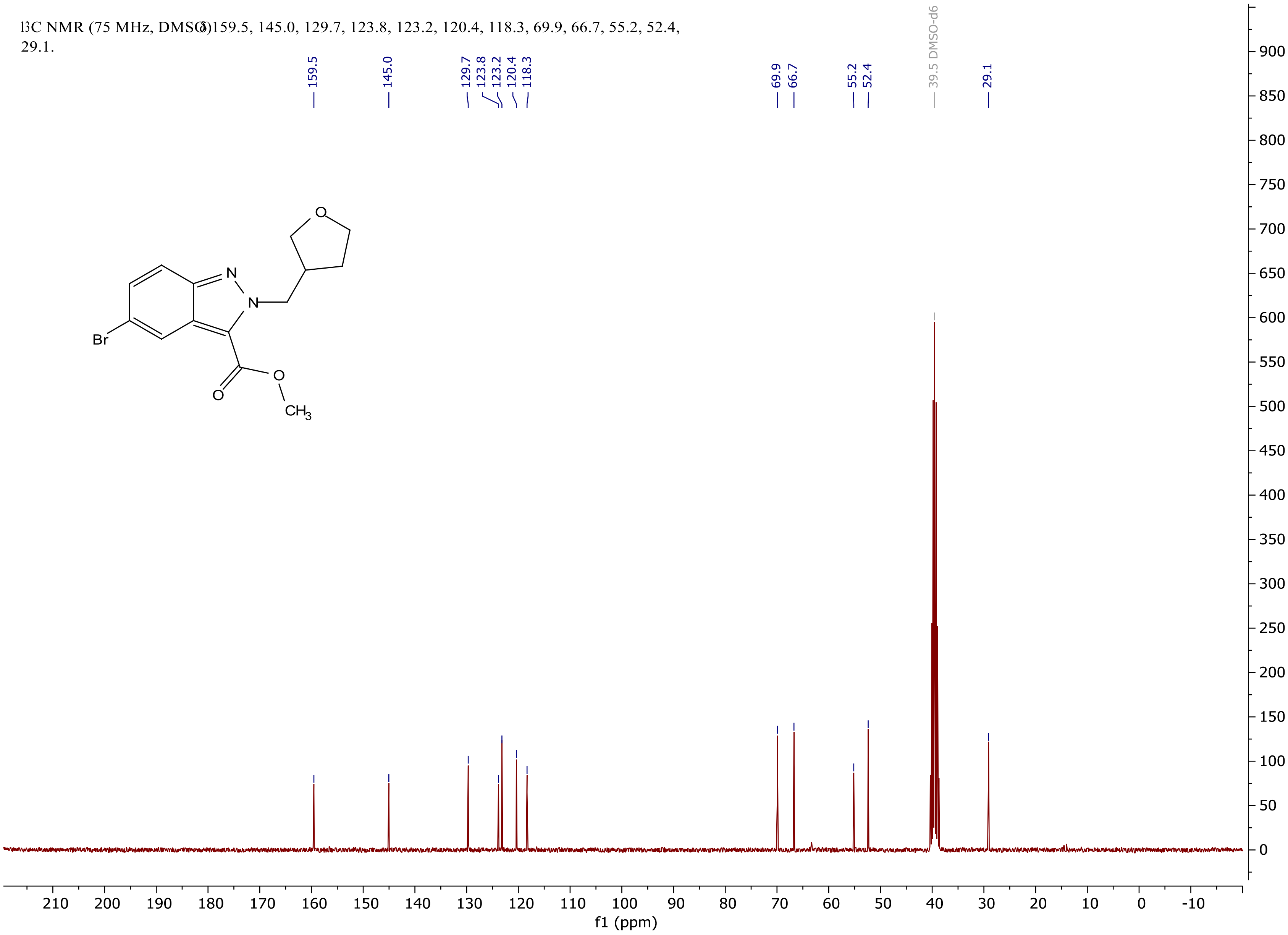

16o-^{13}C

LCJ-007-139 B2.10.fid
LCJ-007-139 B2

1H NMR (300 MHz, DMSO-d_6) δ 8.21 (d, J = 1.9, 0.6 Hz, 1H), 7.89 (d, J = 9.0, 0.7 Hz, 1H), 7.63 (dd, J = 9.0, 1.9 Hz, 1H), 4.38 (d, J = 7.2 Hz, 2H), 3.94 (s, 3H), 2.00 – 1.83 (m, 1H), 1.63 (s, 3H), 1.46 (d, J = 12.1 Hz, 2H), 1.14 (t, J = 9.9 Hz, 3H), 1.02 (d, J = 11.2 Hz, 2H).

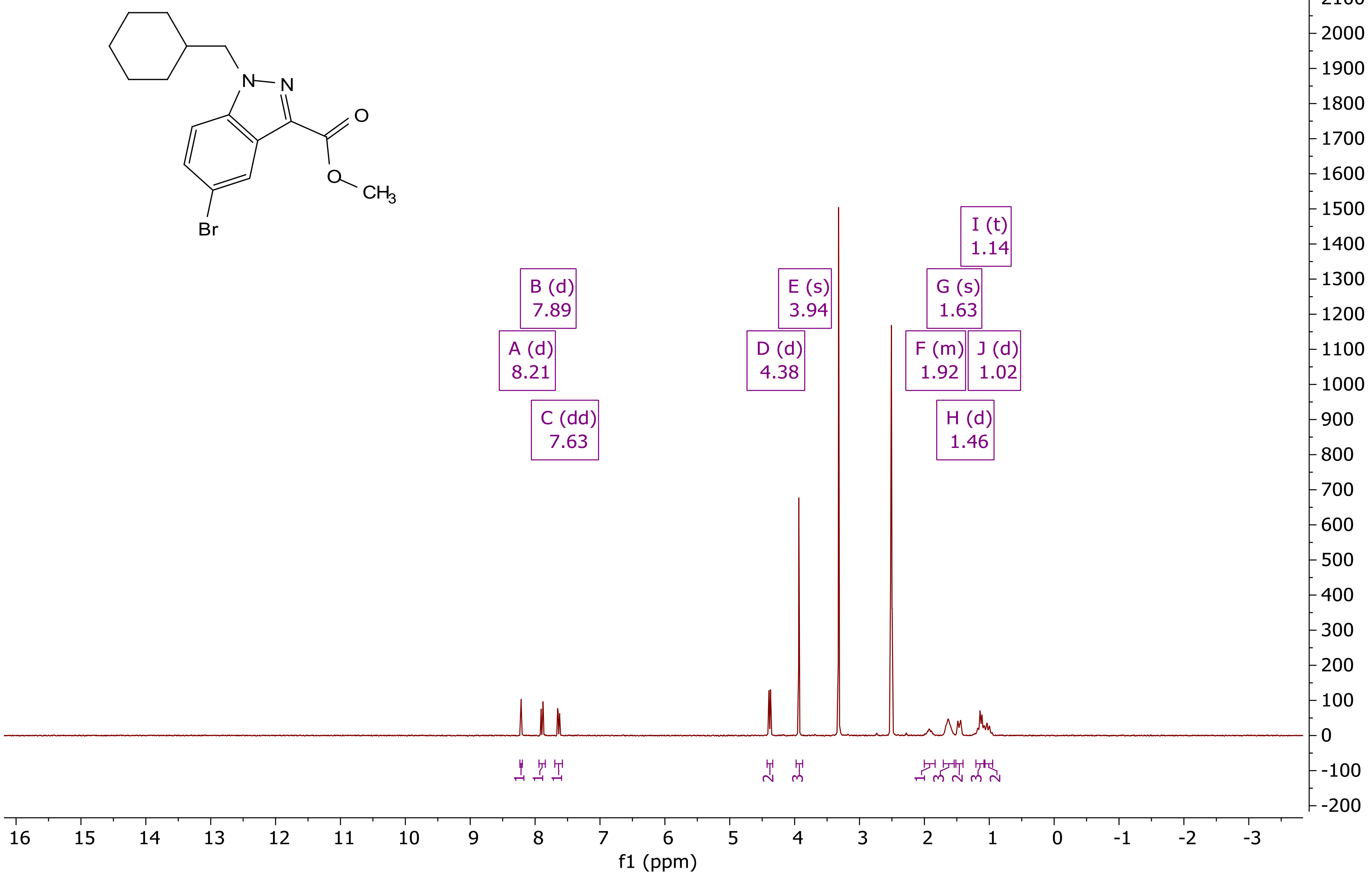

15p-^{1}H

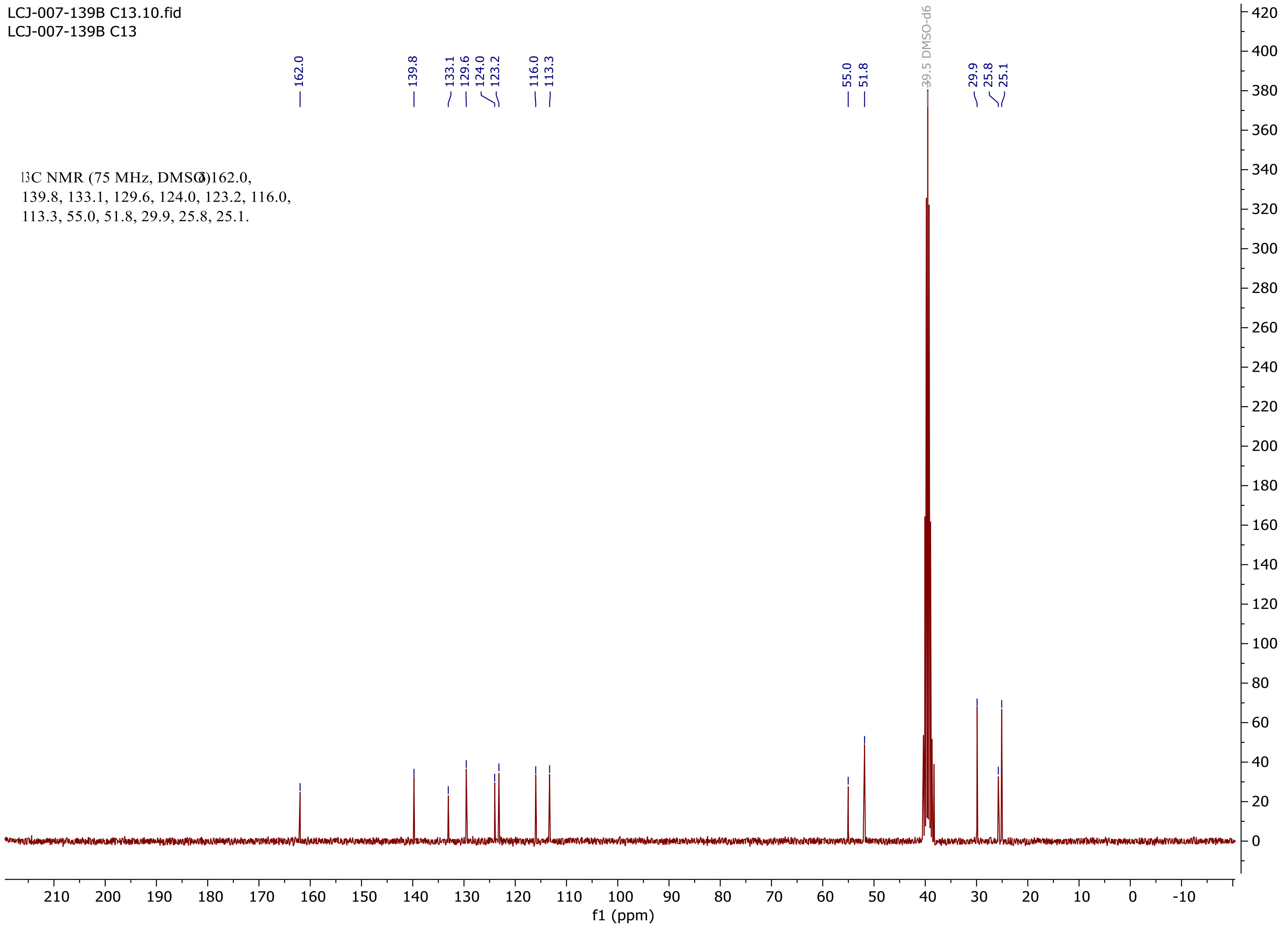

^{13}C NMR (75 MHz, DMSO δ) 162.0, 139.8, 133.1, 129.6, 124.0, 123.2, 116.0, 113.3, 55.0, 51.8, 29.9, 25.8, 25.1.

15p-^{13}C

LCJ-007-139 C1.10.fid
LCJ-007-139 C1

^{1}H NMR (300 MHz, DMSO-d_6) δ 8.17 (d, J = 1.9, 0.8 Hz, 1H), 7.79 (d, J = 9.1, 0.8 Hz, 1H), 7.49 (dd, J = 9.1, 1.9 Hz, 1H), 4.70 (d, J = 7.2 Hz, 2H), 3.99 (s, 3H), 2.03 – 1.89 (m, 1H), 1.61 (d, J = 7.2 Hz, 3H), 1.47 (d, J = 11.8 Hz, 2H), 1.20 – 1.08 (m, 3H), 1.04 (d, J = 11.5 Hz, 2H).

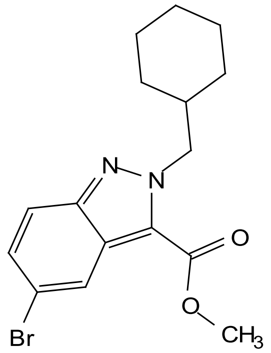

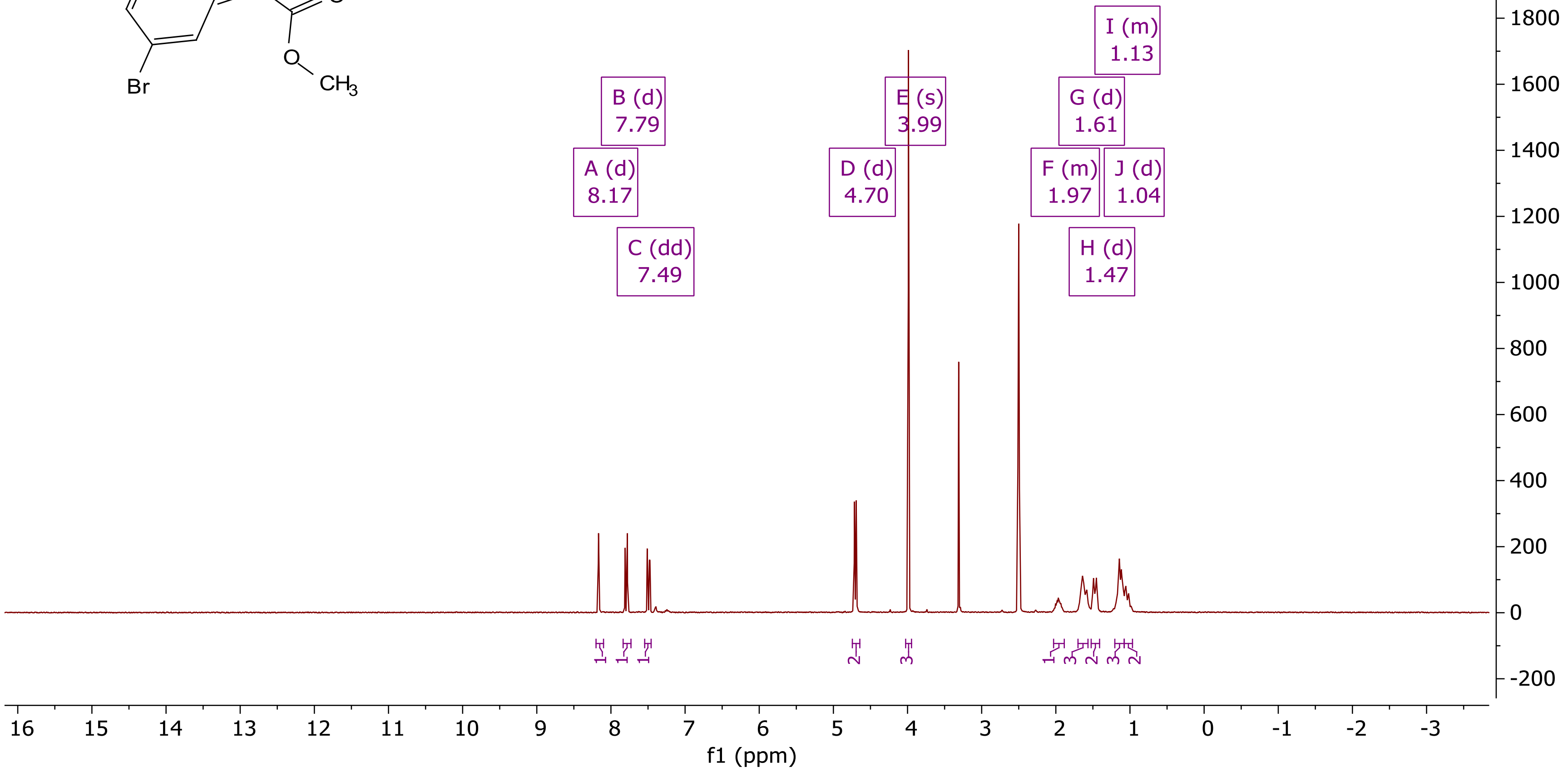

16p-^{1}H

^{13}C NMR (75 MHz, DMSO) δ 159.6, 159.5, 144.9, 133.4, 129.6, 123.8, 123.2, 120.3, 118.2, 58.5, 52.3, 29.9, 25.8, 25.1.

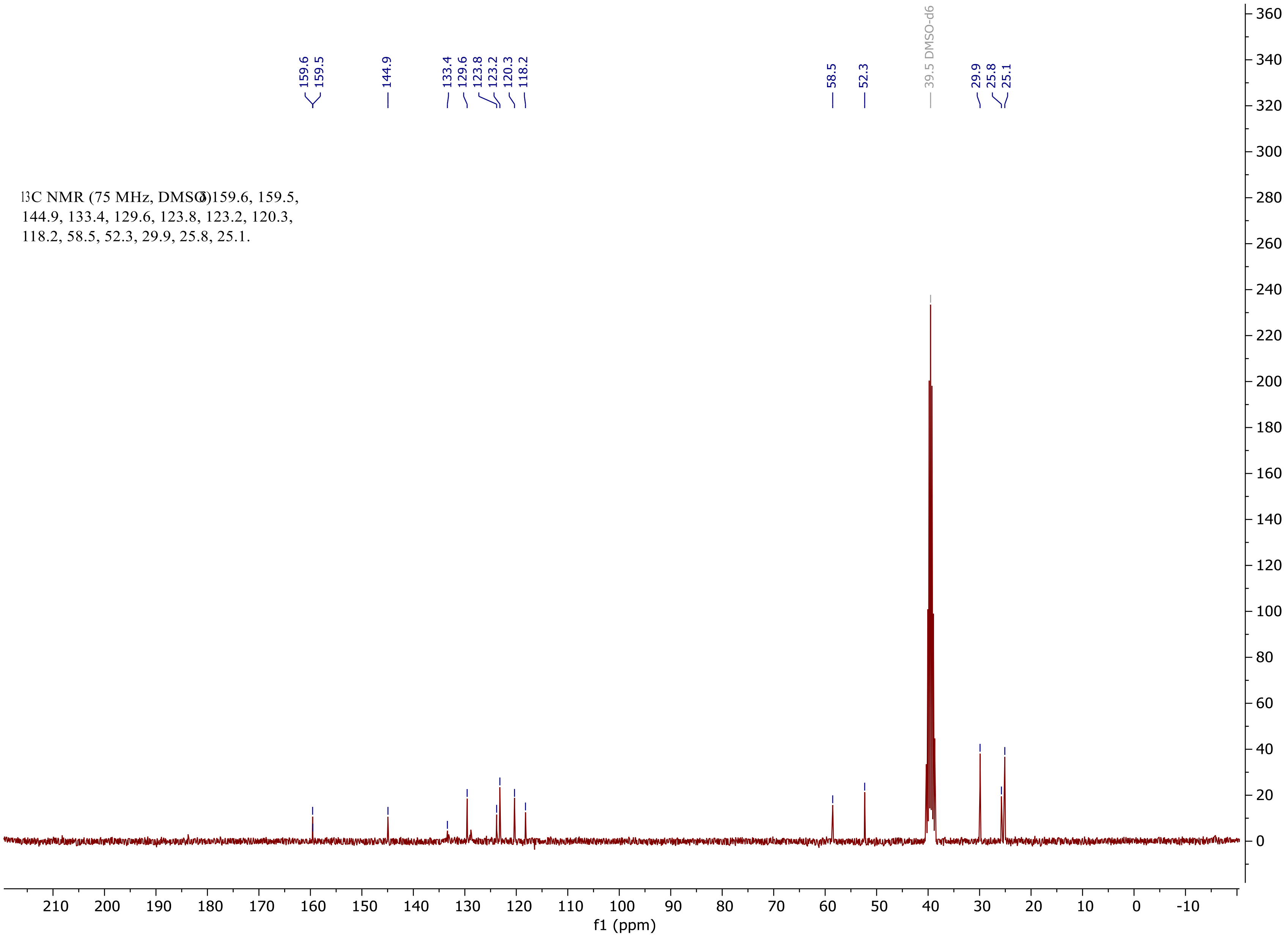

16p-^{13}C

LCJ-009-186B2.10.fid
LCJ-009-186B2

1H NMR (300 MHz, DMSO-d_6) δ 8.22 (d, J = 1.8 Hz, 1H), 7.90 (d, J = 9.0 Hz, 1H), 7.65 (dd, J = 9.0, 1.9 Hz, 1H), 7.39 – 7.21 (m, 5H), 5.80 (s, 2H), 3.93 (s, 3H).

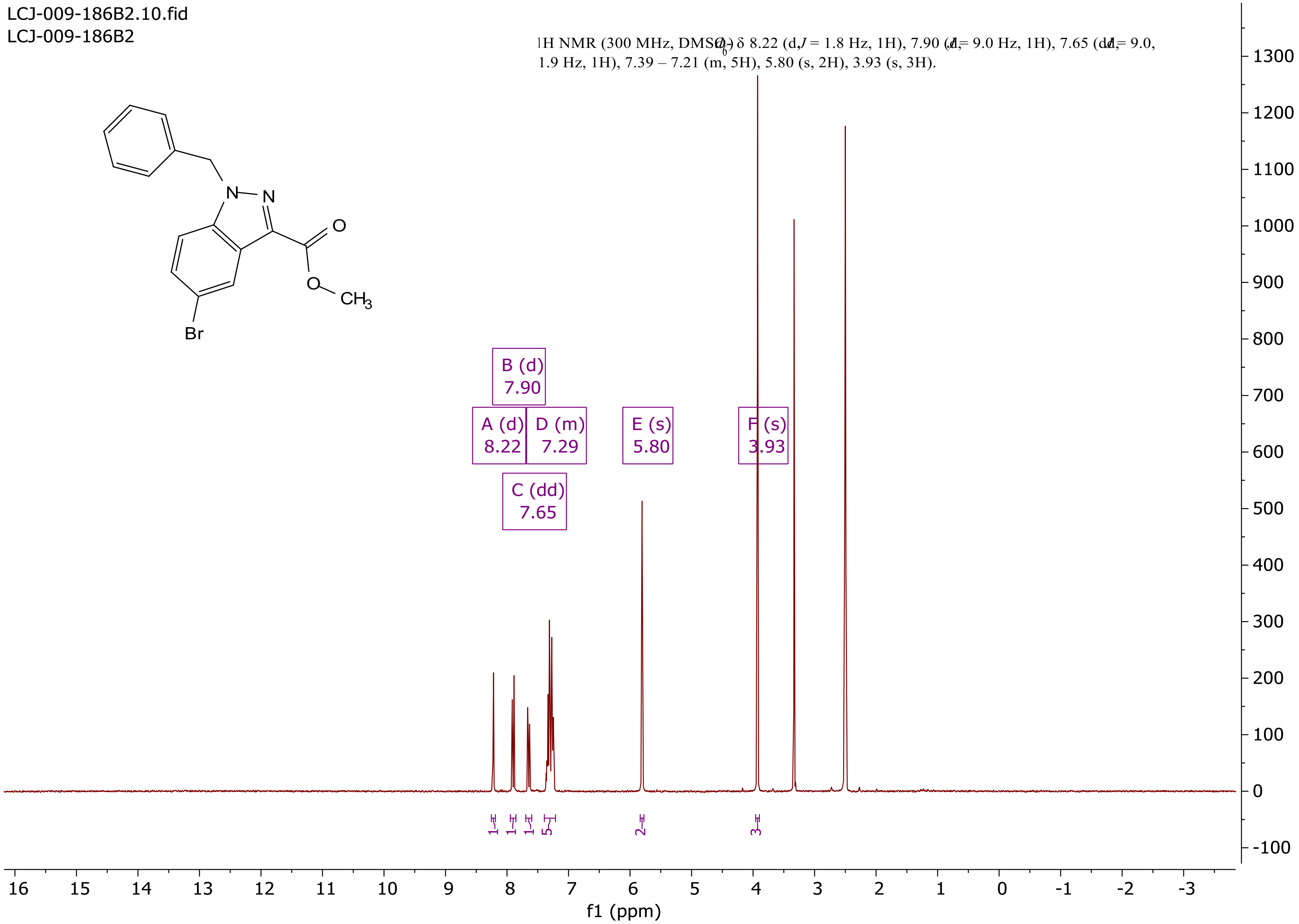

15q-[1]H

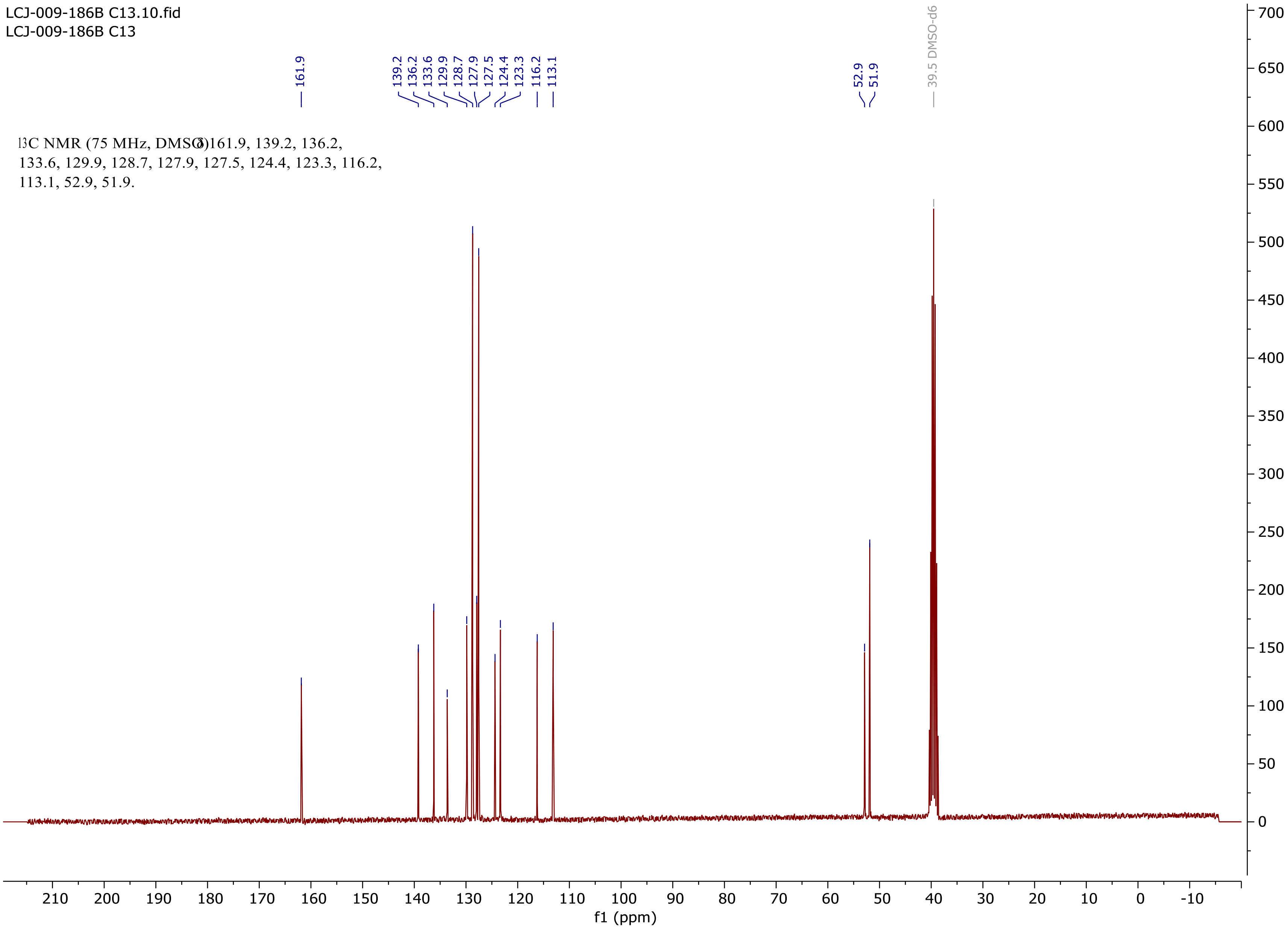

13C NMR (75 MHz, DMSO) δ 161.9, 139.2, 136.2, 133.6, 129.9, 128.7, 127.9, 127.5, 124.4, 123.3, 116.2, 113.1, 52.9, 51.9.

15q-^{13}C

LCJ-009-186C1.10.fid
LCJ-009-186C1

1H NMR (300 MHz, DMSO-d_6) δ 8.18 (d, J = 1.9, 0.8 Hz, 1H), 7.82 (d, J = 9.1, 0.8 Hz, 1H), 7.52 (dd, J = 9.1, 1.9 Hz, 1H), 7.39 – 7.20 (m, 5H), 6.07 (s, 2H), 3.98 (s, 3H).

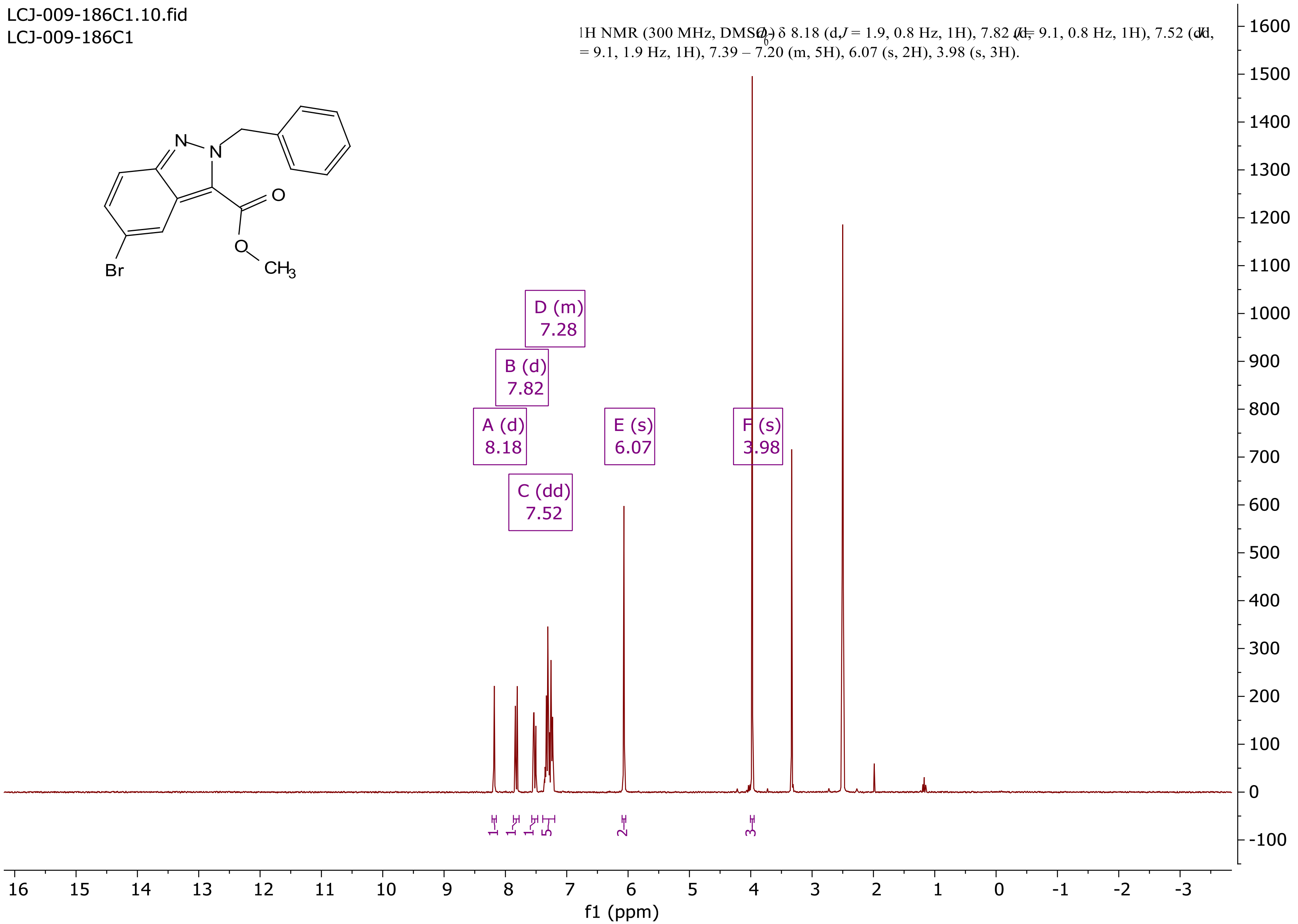

16q-^{1}H

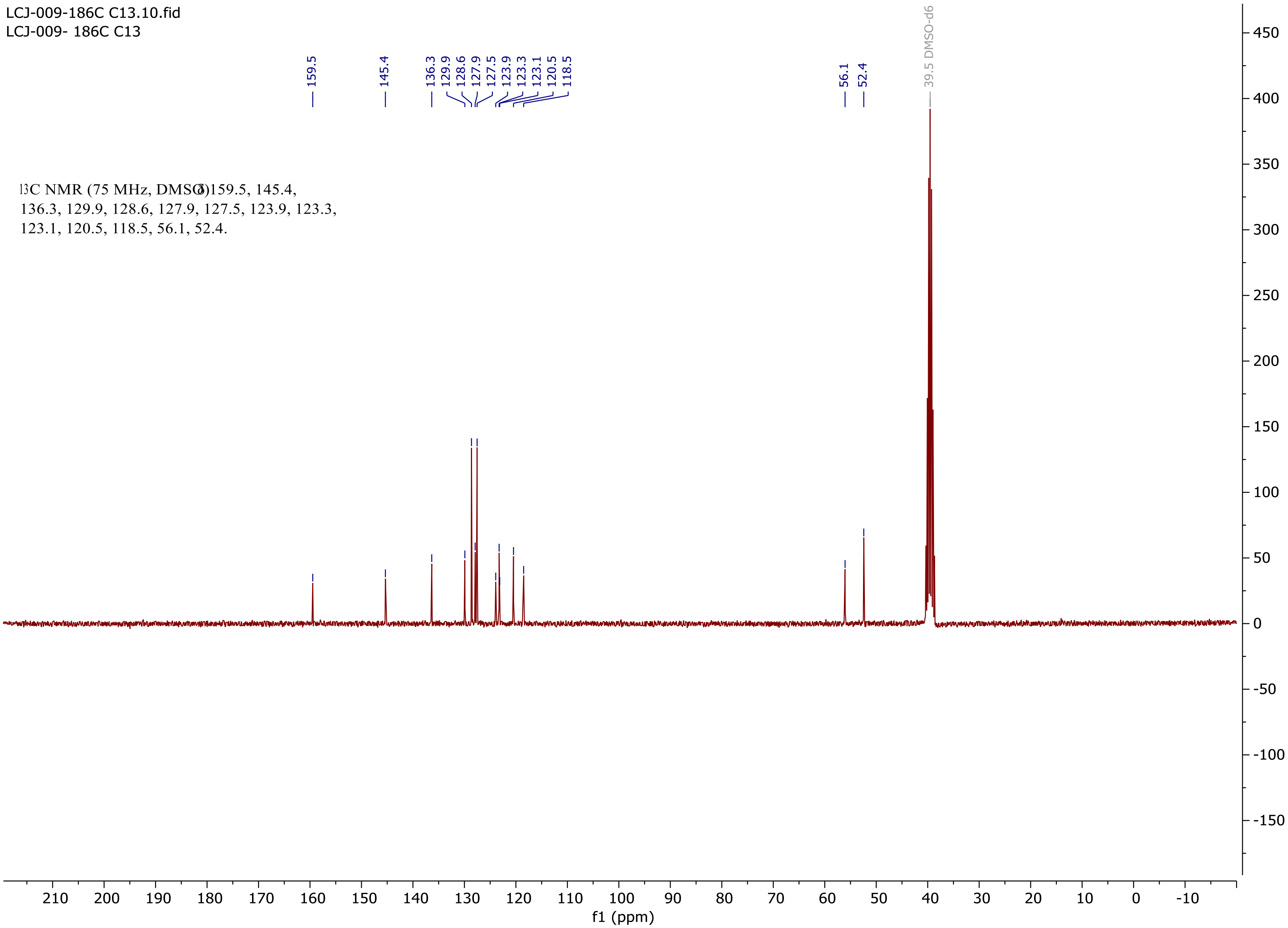
LCJ-009-186C C13.10.fid
LCJ-009- 186C C13
159.5
145.4
136.3
129.9
128.6
127.9
127.5
123.9
123.3
123.1
120.5
118.5
56.1
52.4
39.5 DMSO-d6
13C NMR (75 MHz, DMSO) δ 159.5, 145.4, 136.3, 129.9, 128.6, 127.9, 127.5, 123.9, 123.3, 123.1, 120.5, 118.5, 56.1, 52.4.
f1 (ppm)

16q-^{13}C

Condition A
Major Product

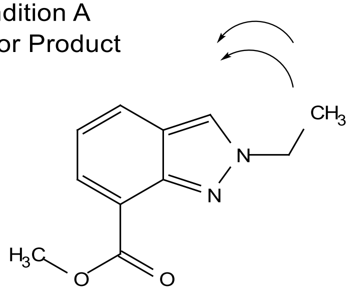

	Parameter	Value
1	Solvent	DMSO
2	Temperature	295.5
3	Pulse Sequence	noesygpphpp
4	Experiment	NOESY
5	Probe	Z104275_0395 (PA BBO 300S1 BBF-H-D-05 Z)
6	Number of Scans	32
7	Receiver Gain	210.0
8	Relaxation Delay	1.9541
9	Pulse Width	14.0000
10	Presaturation Frequency	
11	Acquisition Time	0.3871
12	Acquisition Date	2023-07-17T23:14:13
13	Modification Date	2023-07-18T05:22:11
14	Class	
15	Spectrometer Frequency	(300.13, 300.13)
16	Spectral Width	(2645.5, 2645.5)
17	Lowest Frequency	(188.0, 188.0)
18	Nucleus	(1H, 1H)
19	Acquired Size	(1024, 256)
20	Spectral Size	(1024, 1024)
21	Digital Resolution	(2.58, 2.58)

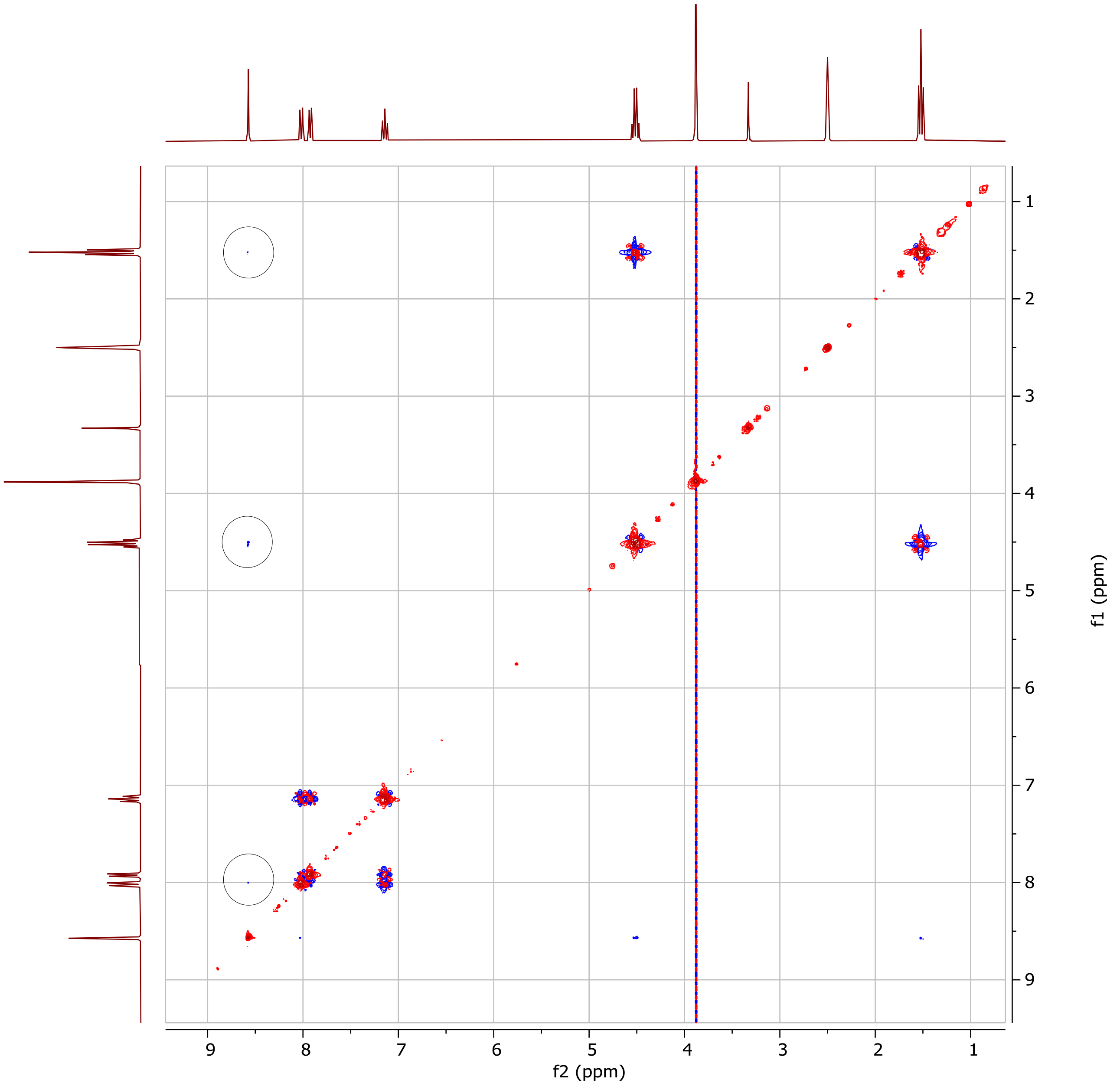

19-NOE

20-¹H

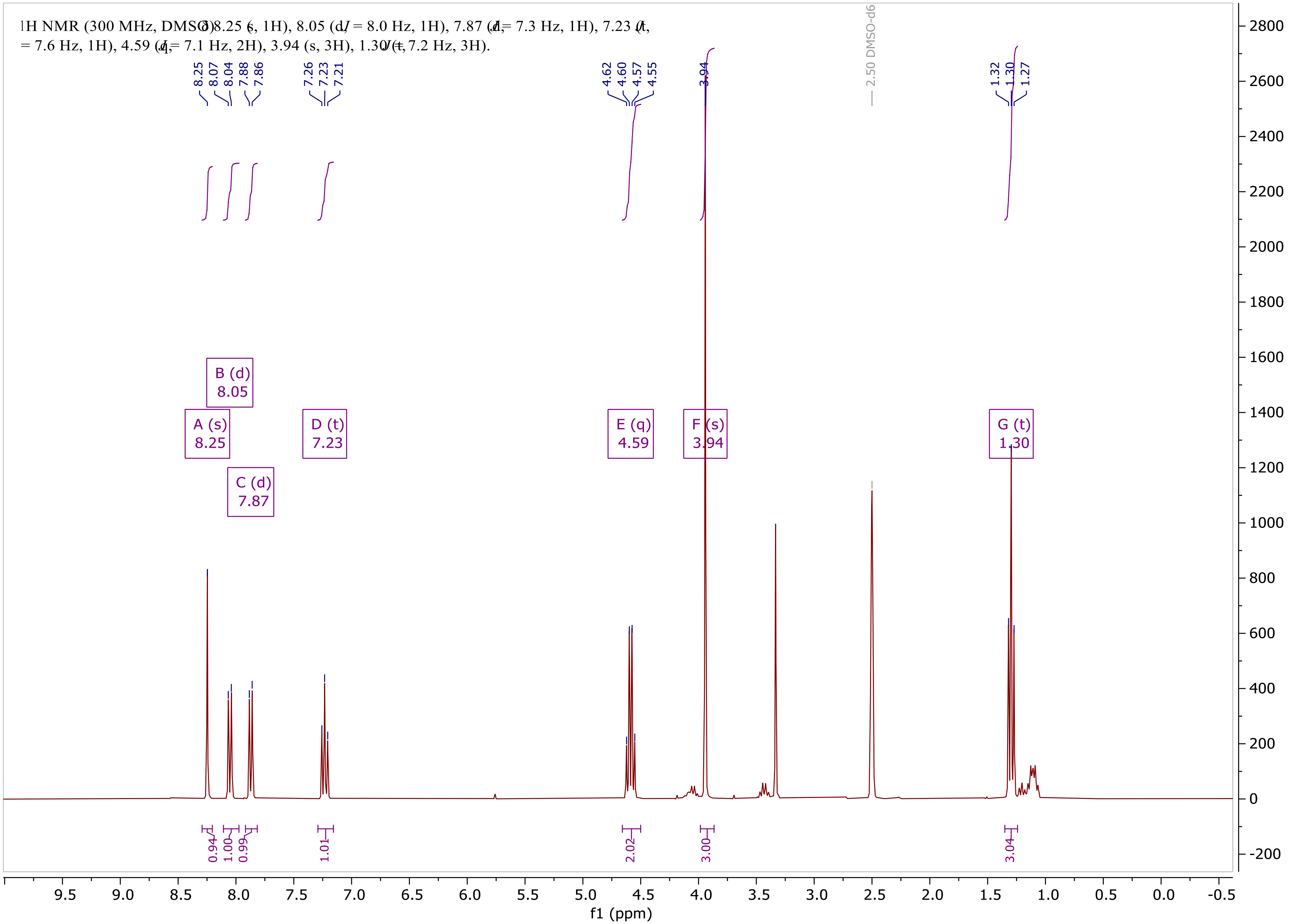
1H NMR (300 MHz, DMSO) δ 8.25 (s, 1H), 8.05 (d, J = 8.0 Hz, 1H), 7.87 (d, J = 7.3 Hz, 1H), 7.23 (t, J = 7.6 Hz, 1H), 4.59 (q, J = 7.1 Hz, 2H), 3.94 (s, 3H), 1.30 (t, J = 7.2 Hz, 3H).
A (s) 8.25
B (d) 8.05
C (d) 7.87
D (t) 7.23
E (q) 4.59
F (s) 3.94
G (t) 1.30
2.50 DMSO-d6
f1 (ppm)

20-^{13}C

13C NMR (75 MHz, DMSO) δ 166.4, 135.2, 133.9, 129.4, 126.3, 126.1, 119.7, 115.0, 52.4, 46.6, 15.1.

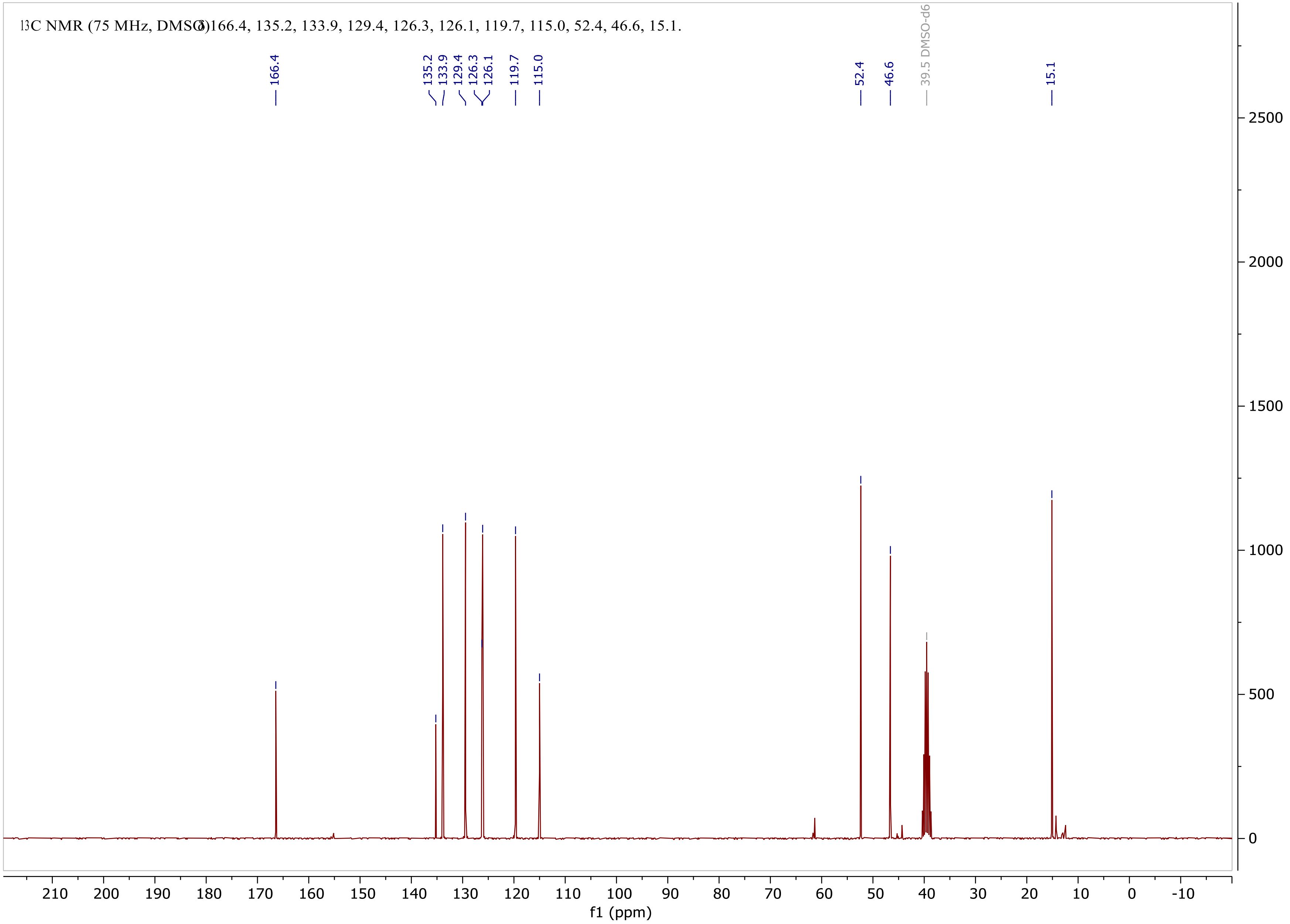

22-^{1}H

Cond. A

1H NMR (300 MHz, DMSO) δ 7.92 (dd, J = 20.4, 8.5 Hz, 2H), 7.59 (t, J = 7.7 Hz, 1H), 7.42 (t, J = 8.0 Hz, 1H), 4.60 (q, J = 6.9 Hz, 2H), 1.45 (t, J = 7.2 Hz, 3H).

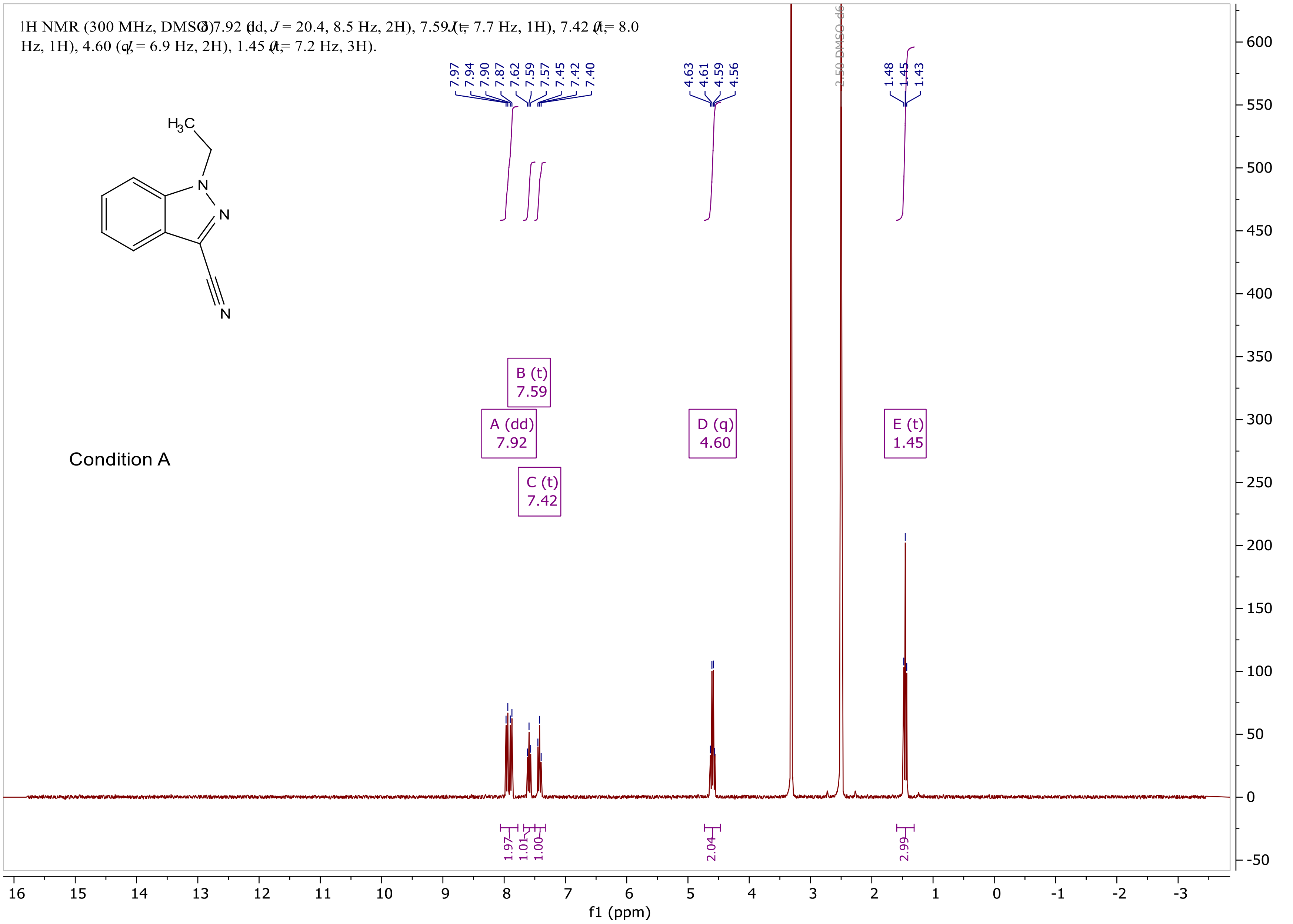

22-^{13}C

Cond. A

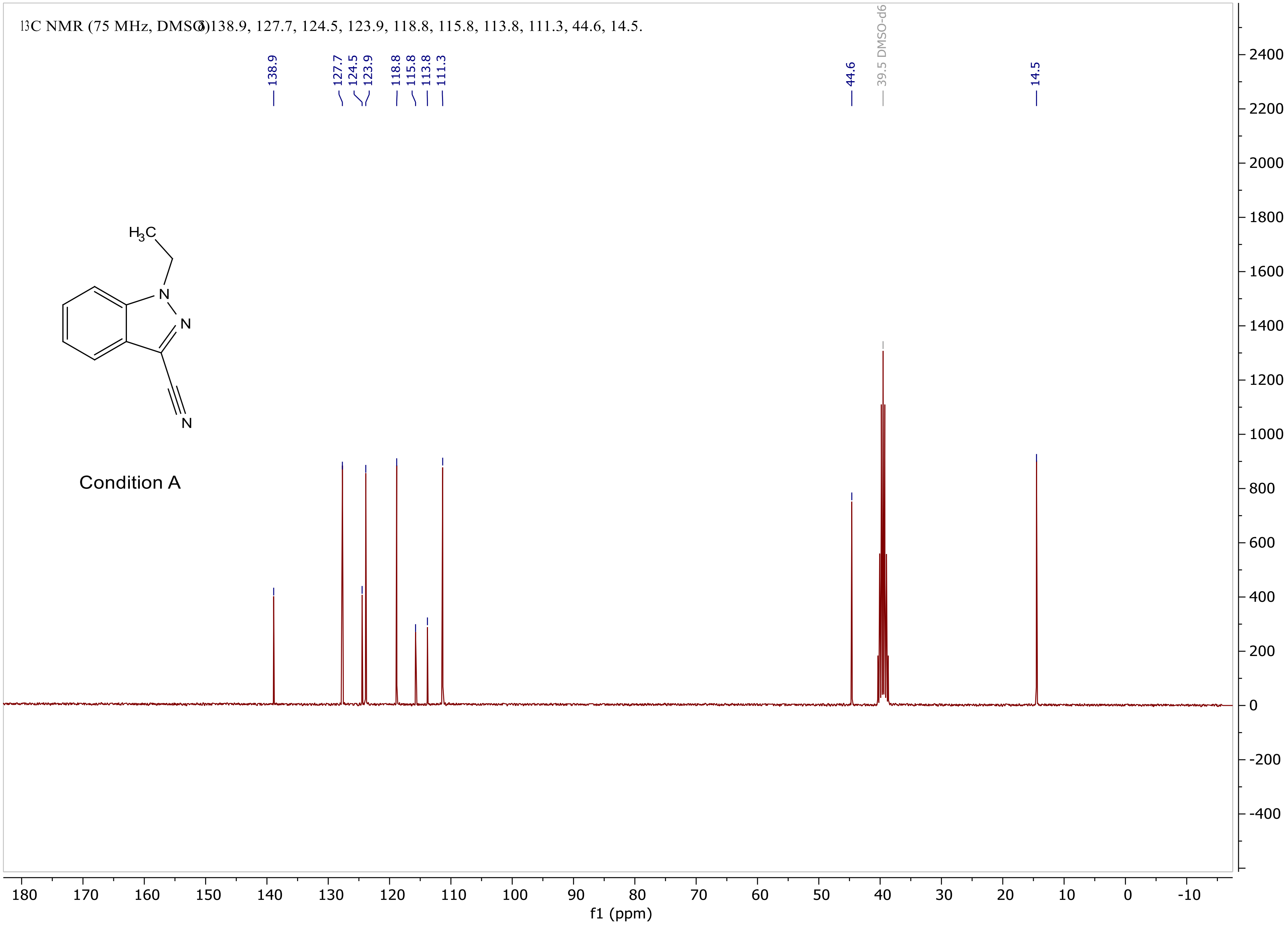
13C NMR (75 MHz, DMSO) δ 138.9, 127.7, 124.5, 123.9, 118.8, 115.8, 113.8, 111.3, 44.6, 14.5.
138.9
127.7
124.5
123.9
118.8
115.8
113.8
111.3
44.6
39.5 DMSO-d6
14.5
H3C
N
N
N
Condition A
f1 (ppm)

22-^{1}H

Cond. B

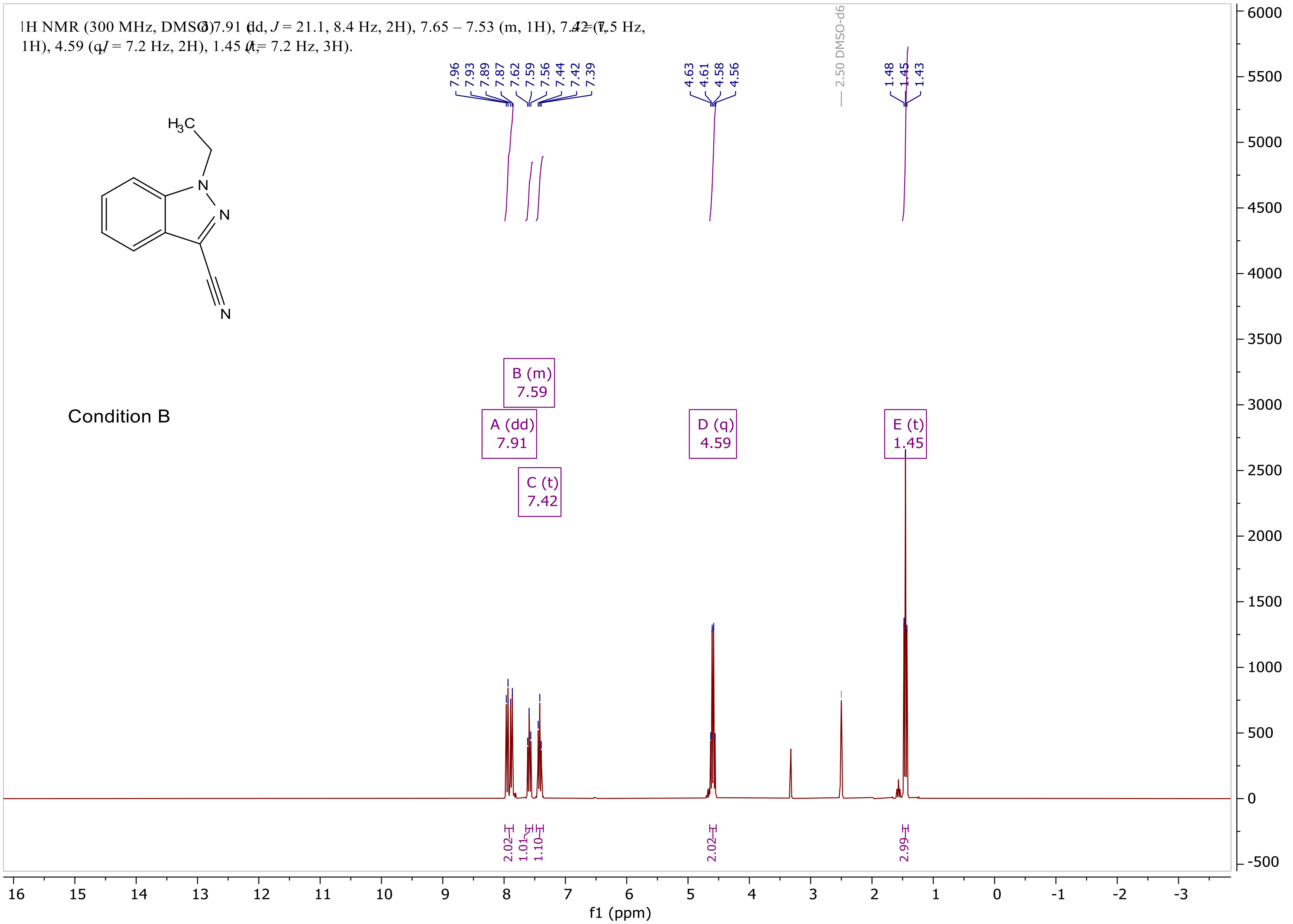

1H NMR (300 MHz, DMSO) δ 7.91 (dd, J = 21.1, 8.4 Hz, 2H), 7.65 – 7.53 (m, 1H), 7.42 (t, J = 7.5 Hz, 1H), 4.59 (q, J = 7.2 Hz, 2H), 1.45 (t, J = 7.2 Hz, 3H).
7.96 7.93 7.89 7.87 7.62 7.59 7.56 7.44 7.42 7.39
4.63 4.61 4.58 4.56
2.50 DMSO-d6
1.48 1.45 1.43
H3C
N
N
N
Condition B
B (m) 7.59
A (dd) 7.91
C (t) 7.42
D (q) 4.59
E (t) 1.45
2.02 1.01 1.10
2.02
2.99
f1 (ppm)

22-^{1}H

Cond. B

13C NMR (75 MHz, DMSO δ) 138.9, 127.7, 124.5, 123.9, 118.8, 115.8, 113.8, 111.3, 44.6, 14.5.

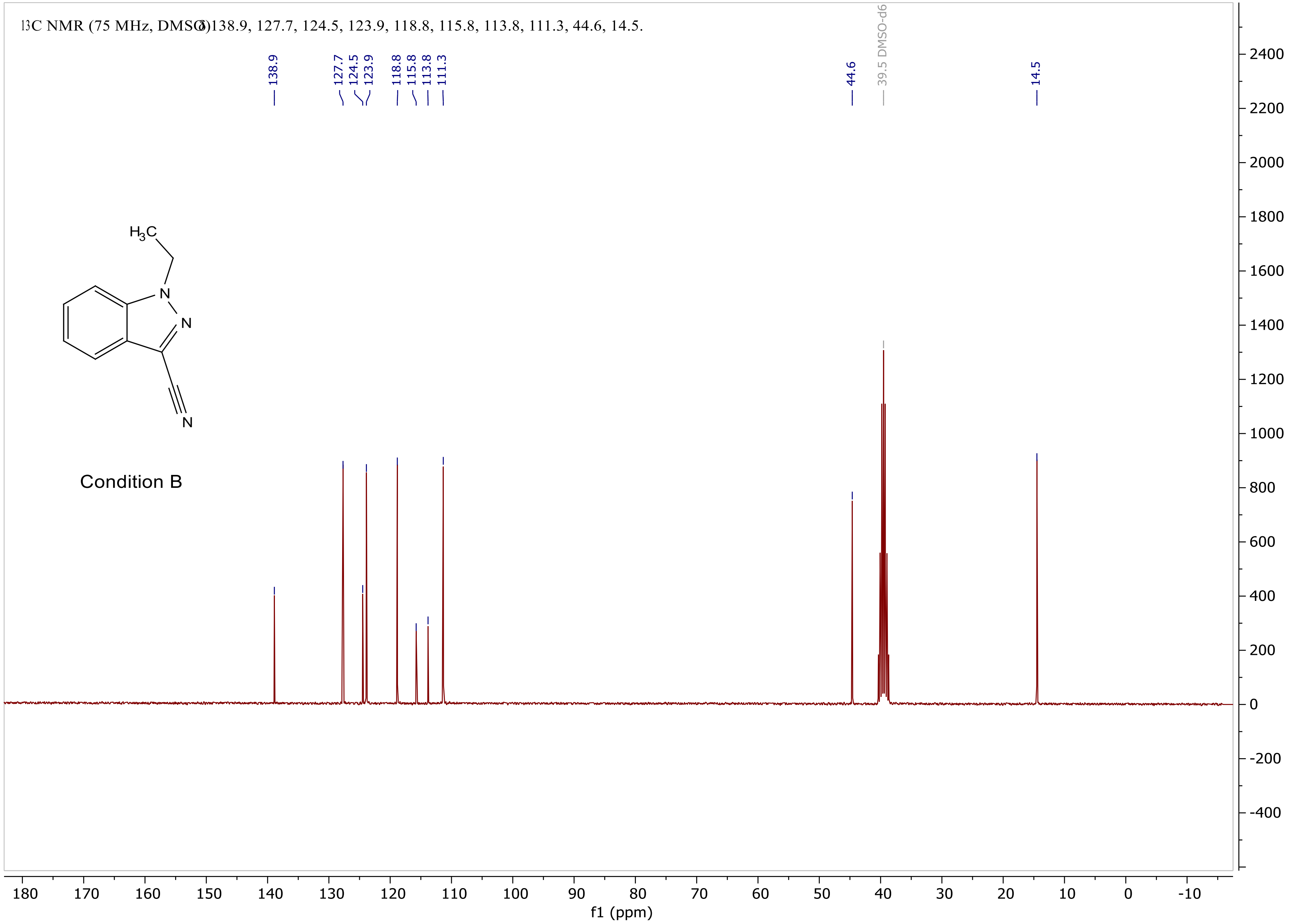

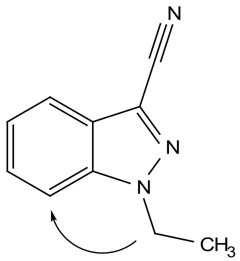

	Parameter	Value
1	Solvent	DMSO
2	Temperature	-4.4
3	Pulse Sequence	noesygpphpp
4	Experiment	NOESY
5	Probe	Z104275_0395 (PA BBO 300S1 BBF-H-D-05 Z)
6	Number of Scans	32
7	Receiver Gain	210.0
8	Relaxation Delay	1.9214
9	Pulse Width	14.0000
10	Presaturation Frequency	
11	Acquisition Time	0.4198
12	Acquisition Date	2023-07-19T21:15:45
13	Modification Date	2023-07-20T03:24:00
14	Class	
15	Spectrometer Frequency	(300.13, 300.13)
16	Spectral Width	(2439.0, 2439.0)
17	Lowest Frequency	(190.9, 190.6)
18	Nucleus	(1H, 1H)
19	Acquired Size	(1024, 256)
20	Spectral Size	(1024, 1024)
21	Digital Resolution	(2.38, 2.38)

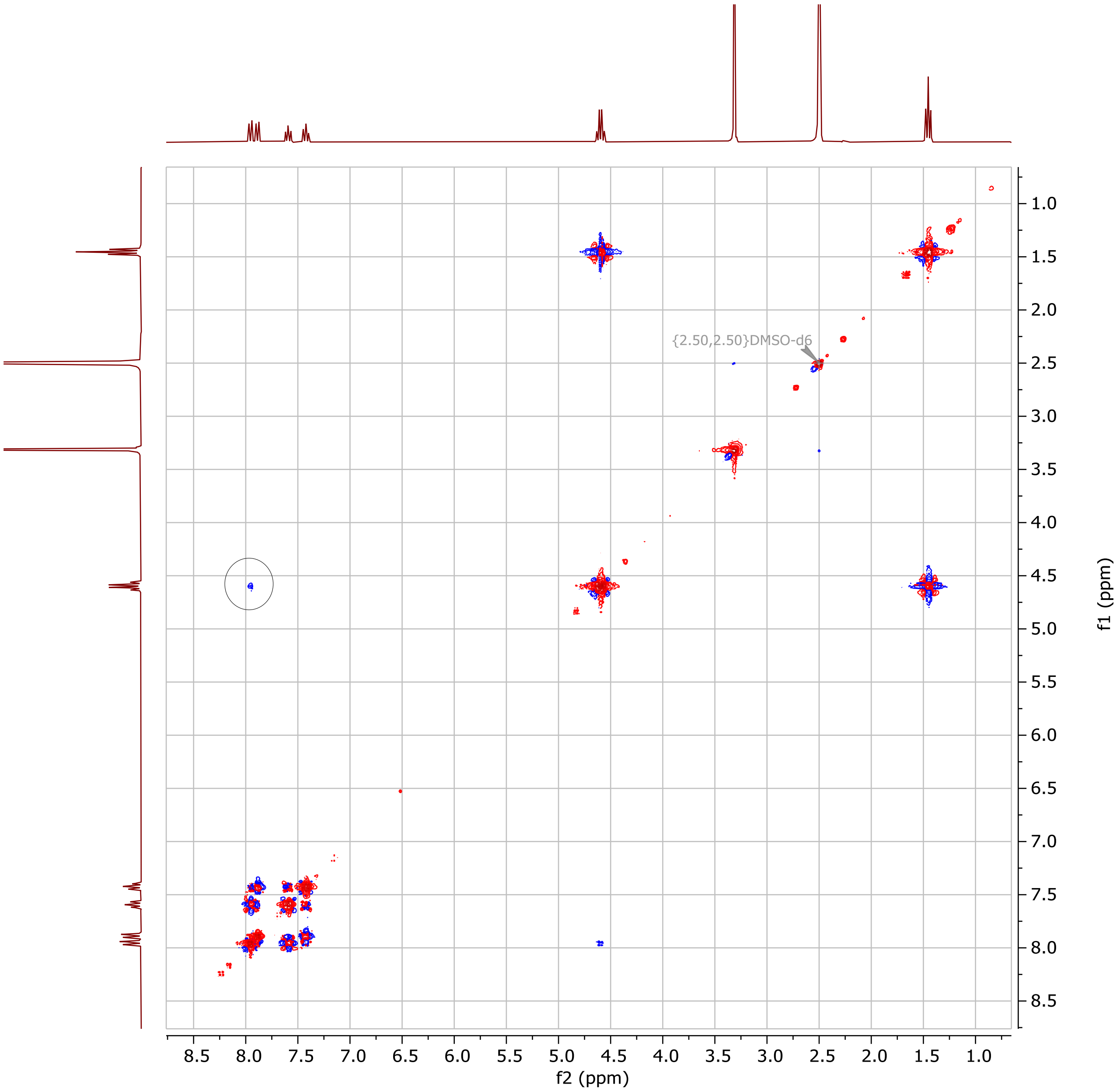

Crystallography data and collection information.

Crystals were grown by liquid-liquid diffusion using acetone and water with a small amount of compound initially dissolved in acetone.

Data collection

A Leica MZ 75 microscope was used to identify a suitable colorless needle with very well-defined faces with dimensions (max, intermediate, and min) 0.471 × 0.141 × 0.085 mm^3 from a representative sample of crystals of the same habit. The crystal mounted on a nylon loop was then placed in a cold nitrogen stream (Oxford) maintained at 110 K.

A BRUKER Quest X-ray (fixed-Chi geometry) diffractometer with a PHOTON II detector was employed for crystal screening, unit cell determination, and data collection. The goniometer was controlled using the APEX3 software suite.[1] The sample was optically centered with the aid of a video camera such that no translations were observed as the crystal was rotated through all positions. The X-ray radiation employed was generated from a Mo-Iμs X-ray tube (K_α = 0.71073Å).

45 data frames were taken at widths of 1°. These reflections were used to determine the unit cell. The unit cell was verified by examination of the *h k l* overlays on several frames of data. No super-cell or erroneous reflections were observed.

After careful examination of the unit cell, an extended data collection procedure (4 sets) was initiated using omega and phi scans.

Data reduction, structure solution, and refinement

Integrated intensity information for each reflection was obtained by reduction of the data frames with the program APEX3.[1] The integration method employed a three-dimensional profiling algorithm, and all data were corrected for Lorentz and polarization factors, as well as for crystal decay effects. Finally, the data were merged and scaled

to produce a suitable data set. The absorption correction program SADABS[2] was employed to correct the data for absorption effects.

Systematic reflection conditions and statistical tests of the data suggested the space group $P2_1/c$. A solution was obtained readily using XT/XS in APEX3.[1,3] Hydrogen atoms were placed in idealized positions and were set riding on the respective parent atoms. All non-hydrogen atoms were refined with anisotropic thermal parameters. Absence of additional symmetry and voids were confirmed using PLATON (ADDSYM). The structure was refined (weighted least squares refinement on F^2) to convergence.[3,4]

Olex2 was employed for the final data presentation and structure plots.[4]

---

[1] APEX3 "Program for Data Collection on Area Detectors" BRUKER AXS Inc., 5465 East Cheryl Parkway, Madison, WI 53711-5373 USA.

[2] SADABS, Sheldrick, G.M. "Program for Absorption Correction of Area Detector Frames", BRUKER AXS Inc., 5465 East Cheryl Parkway, Madison, WI 53711-5373 USA.

[3] Sheldrick, G.M. (2008). Acta Cryst. A64, 112-122. Sheldrick, G. M. (2015), Acta Cryst. A71, 3-8. Sheldrick, G. M. (2015). Acta Cryst. C71, 3-8. XT, XS, BRUKER AXS Inc., 5465 East Cheryl Parkway, Madison, WI 53711-5373 USA.

[4] Dolomanov, O. V, Bourhis, L. J., Gildea, R. J., Howard, J. A. K., and Puschmann, H. "OLEX2: A Complete Structure Solution, Refinement and Analysis Program", *J. Appl. Cryst.* **2009**, *42*, 339-341.

Compound 8 crystal data.

Bond precision: C-C = 0.0030 A Wavelength=0.71073
Cell: a=4.3946(8) b=20.075(3) c=13.610(3)
alpha=90 beta=92.840(7) gamma=90
Temperature: 110 K
Calculated Reported
Volume 1199.2(4) 1199.2(4)
Space group P 21/c P 1 21/c 1
Hall group -P 2ybc -P 2ybc
Moiety formula C12 H13 Br N2 O2 C12 H13 Br N2 O2
Sum formula C12 H13 Br N2 O2 C12 H13 Br N2 O2
Mr 297.14 297.15
Dx,g cm-3 1.646 1.646
Z 4 4
Mu (mm-1) 3.419 3.419
F000 600.0 600.0
F000’ 599.10
h,k,lmax 5,26,17 5,26,17
Nref 2768 2757
Tmin,Tmax 0.659,0.756 0.244,0.431
Tmin’ 0.230
Correction method= # Reported T Limits: Tmin=0.244 Tmax=0.431
AbsCorr = MULTI-SCAN
Data completeness= 0.996 Theta(max)= 27.533
R(reflections)= 0.0263(2422) wR2(reflections)= 0.0594(2757)
S = 1.051 Npar= 157

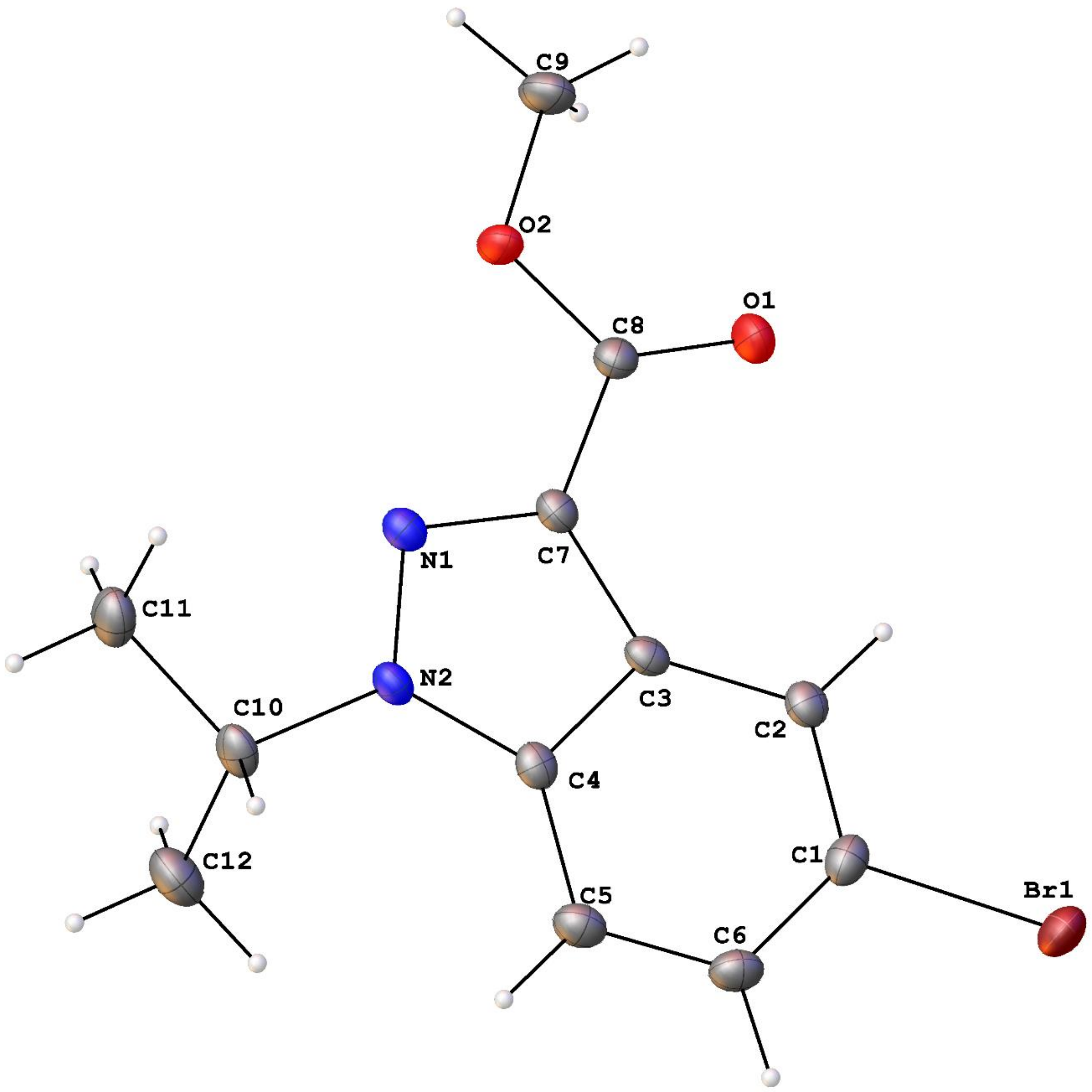

8. Thermal Ellipsoids at 50%

Compound 9 crystal data.

Bond precision: C-C = 0.0030 A Wavelength=0.71073
Cell: a=14.6240(13) b=4.6101(4) c=18.9039(18)
alpha=90 beta=109.153(3) gamma=90
Temperature: 110 K
Calculated Reported
Volume 1203.92(19) 1203.92(19)
Space group P 21/c P 1 21/c 1
Hall group -P 2ybc -P 2ybc
Moiety formula C12 H13 Br N2 O2 C12 H13 Br N2 O2
Sum formula C12 H13 Br N2 O2 C12 H13 Br N2 O2
Mr 297.14 297.15
Dx,g cm-3 1.639 1.639
Z 4 4
Mu (mm-1) 3.406 3.406
F000 600.0 600.0
F000’ 599.10
h,k,lmax 18,5,24 18,5,24
Nref 2743 2741
Tmin,Tmax 0.568,0.749 0.439,0.746
Tmin’ 0.199
Correction method= # Reported T Limits: Tmin=0.439 Tmax=0.746
AbsCorr = MULTI-SCAN
Data completeness= 0.999 Theta(max)= 27.473
R(reflections)= 0.0273(2252) wR2(reflections)= 0.0662(2741)
S = 1.037 Npar= 157

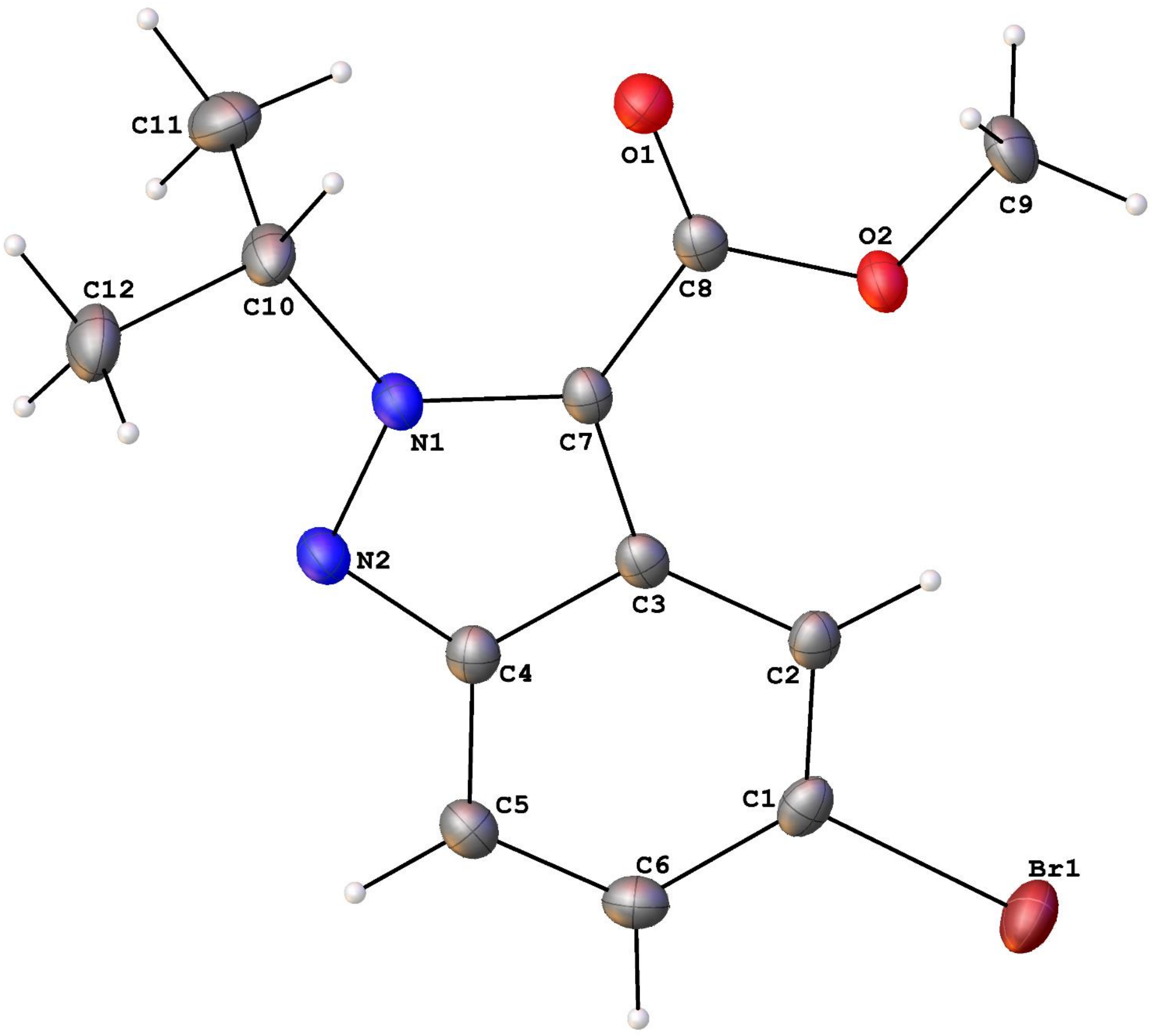

9. Thermal ellipsoids drawn at 50%.

Compound 16m crystal data

Empirical formula C17 H20 Br N3 O4
Formula weight 410.27
Temperature 110.0 K
Wavelength 1.54178 Å
Crystal system Triclinic
Space group P-1
Unit cell dimensions a = 6.7985(4) Å □= 98.920(3)°.
b = 10.3761(5) Å □= 94.802(3)°.
c = 13.0352(5) Å □ = 93.714(3)°.
Volume 902.39(8) Å3
Z 2
Density (calculated) 1.510 Mg/m3
Absorption coefficient 3.341 mm-1
F(000) 420
Crystal size 0.249 x 0.041 x 0.033 mm3
Theta range for data collection 3.448 to 64.998°.
Index ranges -7<=h<=7, -12<=k<=12, -15<=l<=15
Reflections collected 13661
Independent reflections 3034 [R(int) = 0.0290]
Completeness to theta = 64.998° 99.4%
Absorption correction Semi-empirical from equivalents
Max. and min. transmission 0.3810 and 0.2211
Refinement method Full-matrix least-squares on F2
Data / restraints / parameters 3034 / 0 / 230
Goodness-of-fit on F2 1.123
Final R indices [I>2sigma(I)] R1 = 0.0467, wR2 = 0.1412
R indices (all data) R1 = 0.0472, wR2 = 0.1417
Extinction coefficient n/a
Largest diff. peak and hole 2.056 and -0.424 e.Å-3

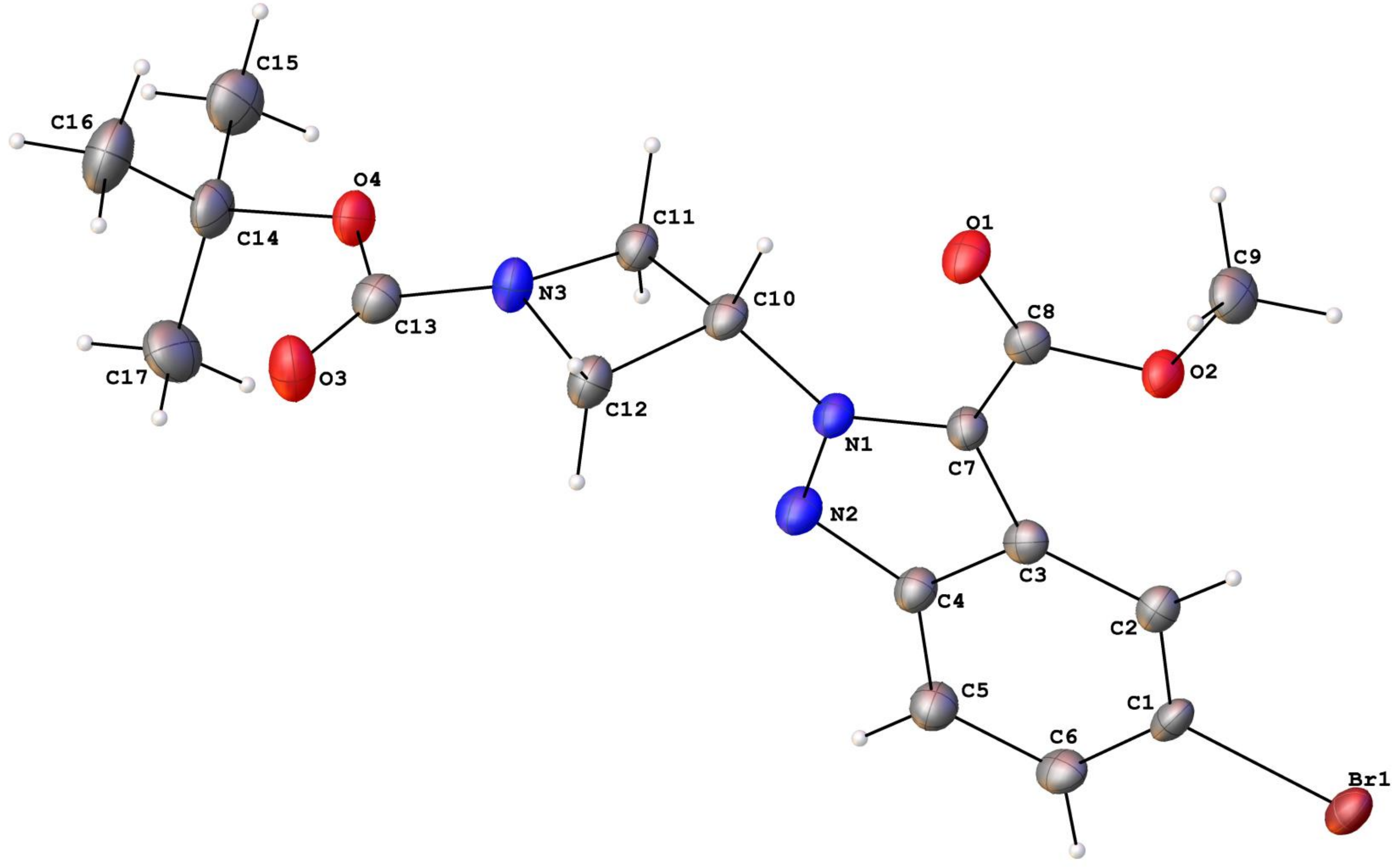

16m. Thermal ellipsoids drawn at 50%.

IR Spectra of all compounds

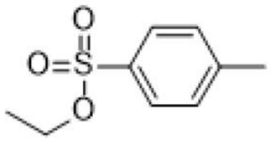

MW 21 July 23

Sample ID:LCJ-004-177A

Sample Scans:32
Background Scans:32
Resolution:2
System Status:Good

Method Name:C:\Users\Public\Documents\Agilent\MicroLab\Methods\400-CM.a2m
User:admin
Date/Time:07/21/2023 8:42:01 AM
Range:4000 - 400
Apodization:Happ-Genzel

File Location:C:\Users\Public\Documents\Agilent\MicroLab\Results\\LCJ-004-177A_2023-07-21T08-42-01.a2r

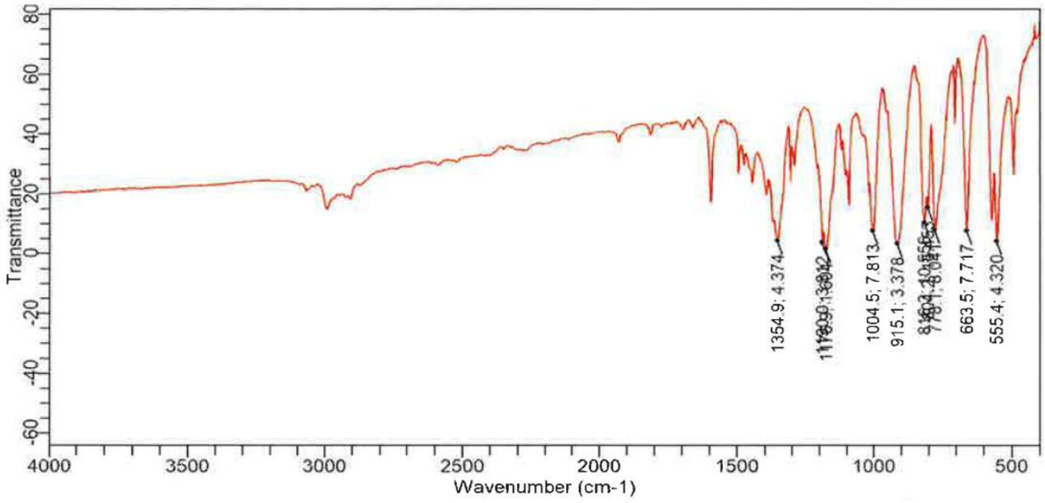

Peak Number	Wavenumber (cm⁻¹)	Intensity
1	555.37334	4.32017
2	663.46614	7.71654
3	778.08177	8.04144
4	804.17314	15.45306
5	816.28699	10.55550

6	915.06144	3.37847
7	1004.51755	7.81263
8	1176.90692	1.60432
9	1189.95260	3.81152
10	1354.88730	4.37407

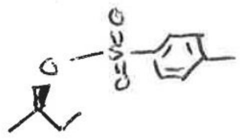

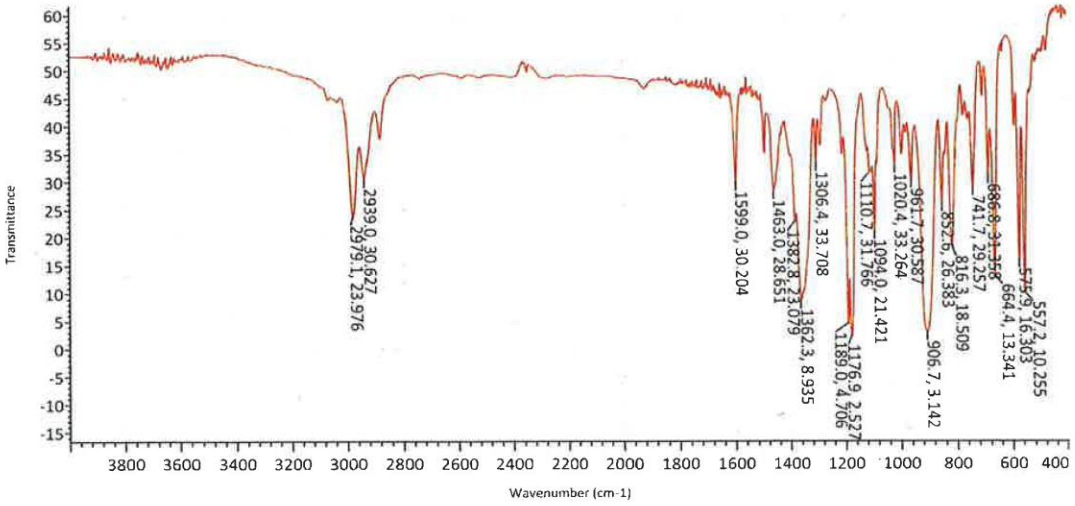

Peak Number	Wavenumber (cm⁻¹)	Intensity
1	557.2	10.255
2	575.9	16.303
3	664.4	13.341
4	686.8	31.358
5	741.7	29.257
6	816.3	18.509
7	852.6	26.383
8	906.7	3.142
9	961.7	30.587
10	1020.4	33.264
11	1094.0	21.421
12	1110.7	31.766

13	1176.9	2.527
14	1189.0	4.706
15	1306.4	33.708
16	1362.3	8.935
17	1382.8	23.079
18	1463.0	28.651
19	1599.0	30.204
20	2939.0	30.627
21	2979.1	23.976

E-SIGNATURES

Signature

EB 21 July 2023

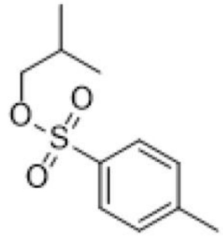

Sample ID:LCJ-7-154A

Sample Scans:32
Background Scans:32
Resolution:2
System Status:Good

Method Name:C:\Users\Public\Documents\A_b\Methods\400-CM.a2m
User:admin
Date/Time:07/21/2023 12:27:22 PM
Range:4000 - 400
Apodization:Happ-Genzel

File Location:C:\Users\Public\Documents\Agilent\MicroLab\Results\\LCJ-7-154A_2023-07-21T12-27-22.a2r

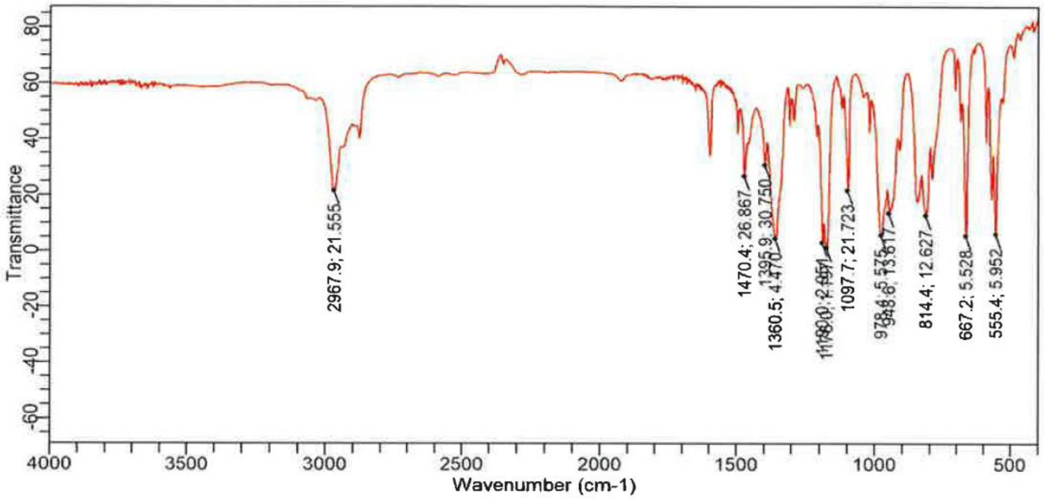

Peak Number	Wavenumber (cm^{-1})	Intensity
1	555.37334	5.95184
2	667.19347	5.52762
3	814.42332	12.62689
4	948.60748	13.61750
5	978.42618	5.57532

6	1097.70099	21.72255
7	1175.97509	1.19730
8	1189.95260	2.95069
9	1360.47831	4.47008
10	1395.88802	30.74959
11	1470.43478	26.86714
12	2967.89275	21.55489

bio cryst

MW 21 July 23

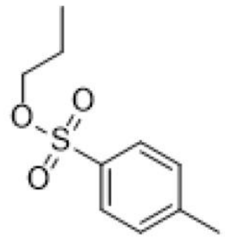

Sample ID:LCJ-007-136A

Sample Scans:32
Background Scans:32
Resolution:2
System Status:Good

Method Name:C:\Users\Public\Documents\Agilent\MicroLab\Methods\400-CM.a2m
User:admin
Date/Time:07/21/2023 9:17:12 AM
Range:4000 - 400
Apodization:Happ-Genzel

File Location:C:\Users\Public\Documents\Agilent\MicroLab\Results\\LCJ-007-136A_2023-07-21T09-17-12.a2r

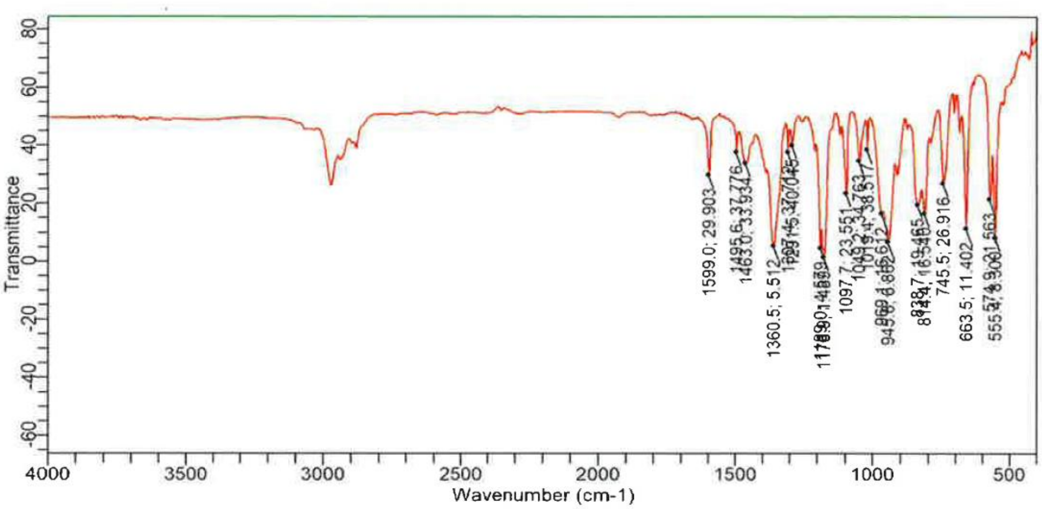

Peak Number	Wavenumber (cm⁻¹)	Intensity
1	555.37334	8.29953
2	574.94186	21.56274
3	663.46614	11.40194
4	745.46757	26.91646
5	814.42332	16.54014

6	838.65101	19.46462
7	945.81198	6.86168
8	969.10784	16.61207
9	1019.42690	38.51664
10	1049.24560	34.76292
11	1097.70099	23.55116
12	1176.90692	1.48874
13	1189.02077	4.57866
14	1291.52256	40.04533
15	1307.36375	37.71186
16	1360.47831	5.51195
17	1462.98010	33.93383
18	1495.59431	37.77560
19	1599.02793	29.90264

bio cryst

MW 21 July 23

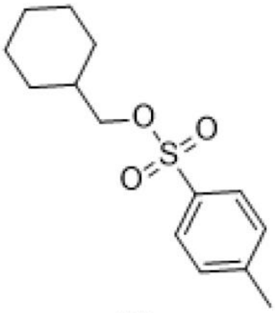

Sample ID:LCJ-007-139A

Sample Scans:32
Background Scans:32
Resolution:2
System Status:Good

Method Name:C:\Users\Public\Documents b\Methods\400-CM.a2m
User:admin
Date/Time:07/21/2023 9:31:21 AM
Range:4000 - 400
Apodization:Happ-Genzel

File Location:C:\Users\Public\Documents\Agilent\MicroLab\Results\\LCJ-007-139A_2023-07-21T09-31-21.a2r

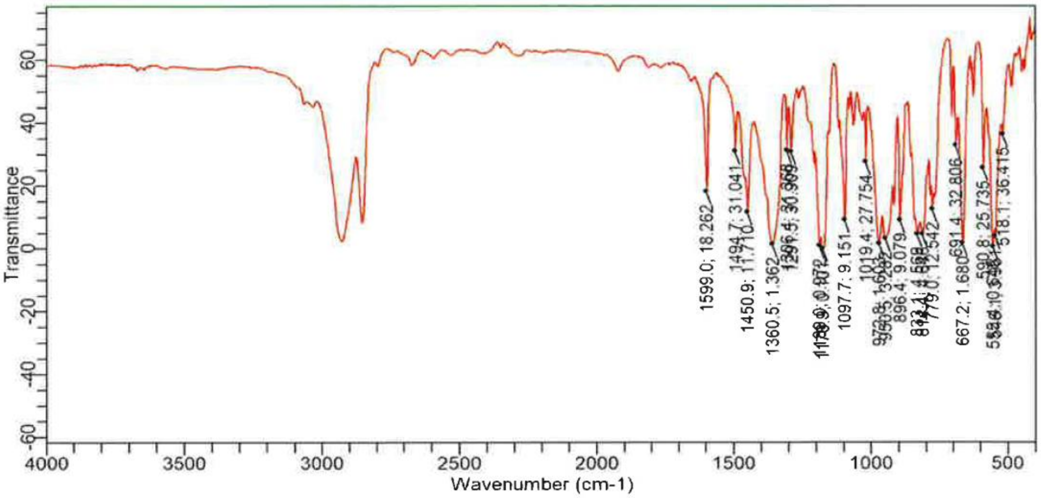

Peak Number	Wavenumber (cm^{-1})	Intensity
1	518.09996	36.41539
2	546.05499	3.96116
3	555.37334	0.64514
4	590.78305	25.73509
5	667.19347	1.67954

6	691.42117	32.80607
7	779.01361	12.54196
8	814.42332	4.64590
9	833.06001	4.56896
10	896.42475	9.07888
11	950.47115	3.28187
12	972.83518	1.60309
13	1019.42690	27.75373
14	1097.70099	9.15143
15	1176.90692	0.10147
16	1189.02077	0.97163
17	1291.52256	30.90919
18	1306.43191	31.36757
19	1360.47831	1.36246
20	1450.86625	11.70978
21	1494.66247	31.04129
22	1599.02793	18.26150

MW 21 July 23

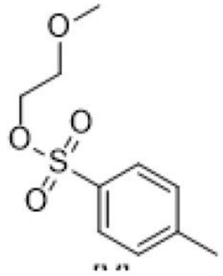

Sample ID:LCJ-007-143A

Sample Scans:32
Background Scans:32
Resolution:2
System Status:Good

Method Name:C:\Users\Public\Documents\Agile b\Methods\400-CM.a2m
User:admin
Date/Time:07/21/2023 11:16:54 AM
Range:4000 - 400
Apodization:Happ-Genzel

File Location:C:\Users\Public\Documents\Agilent\MicroLab\Results\\LCJ-007-143A_2023-07-21T11-16-54.a2r

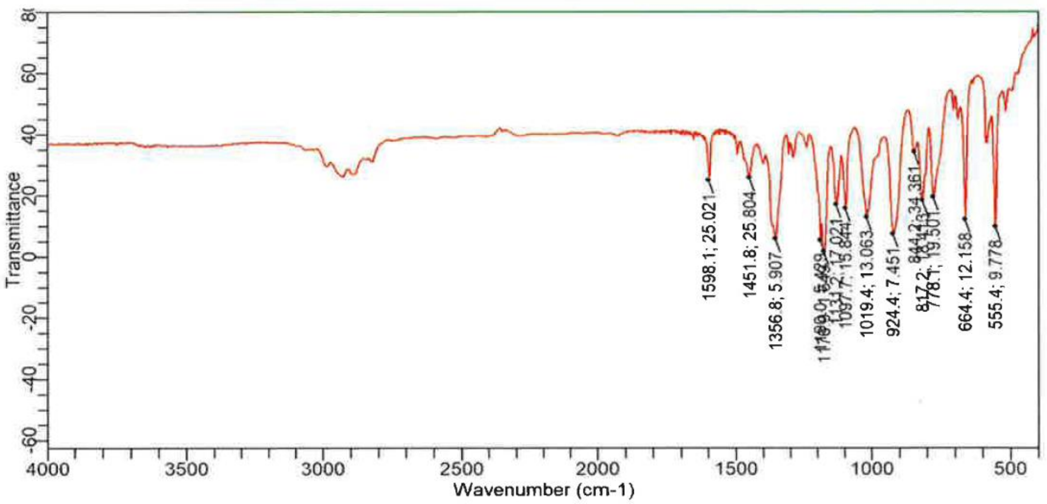

Peak Number	Wavenumber (cm-1)	Intensity
1	555.37334	9.77778
2	664.39797	12.15797
3	778.08177	19.50105
4	817.21882	18.41314
5	844.24202	34.36130

6	924.37978	7.45086
7	1019.42690	13.06254
8	1097.70099	15.84414
9	1131.24703	17.02116
10	1176.90692	1.64900
11	1189.95260	5.42944
12	1356.75097	5.90657
13	1451.79809	25.80354
14	1598.09610	25.02067

File Location:NAPLBBHPC2L810D\SQLEXPRESS|VAIMDB_Public(000)|Public Results\LCJ-007-140A.a2r

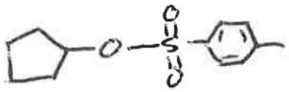

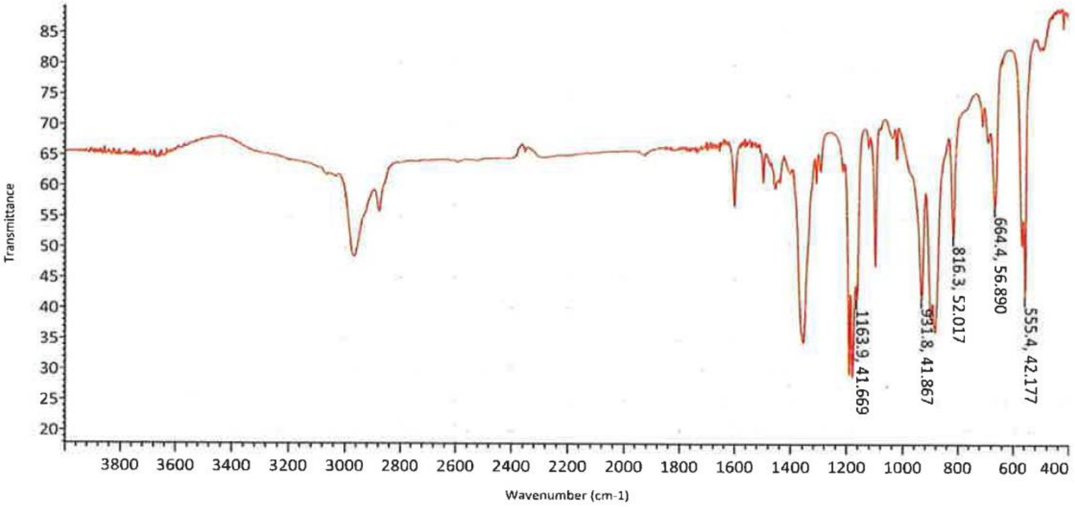

Peak Number	Wavenumber (cm^{-1})	Intensity
1	555.4	42.177
2	664.4	56.890
3	816.3	52.017
4	931.8	41.867
5	1163.9	41.669

E-SIGNATURES

Signature

bio cryst

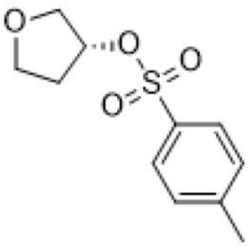

MW 21 July 2-

Sample ID:LCJ-007-147A

Sample Scans:32
Background Scans:32
Resolution:2
System Status:Good

Method Name:C:\Users\Public\Documents\Agilent\MicroLab\Methods\400-CM.a2m
User:admin
Date/Time:07/21/2023 11:29:15 AM
Range:4000 - 400
Apodization:Happ-Genzel

File Location:C:\Users\Public\Documents\Agilent\MicroLab\Results\\LCJ-007-147A_2023-07-21T11-29-15.a2r

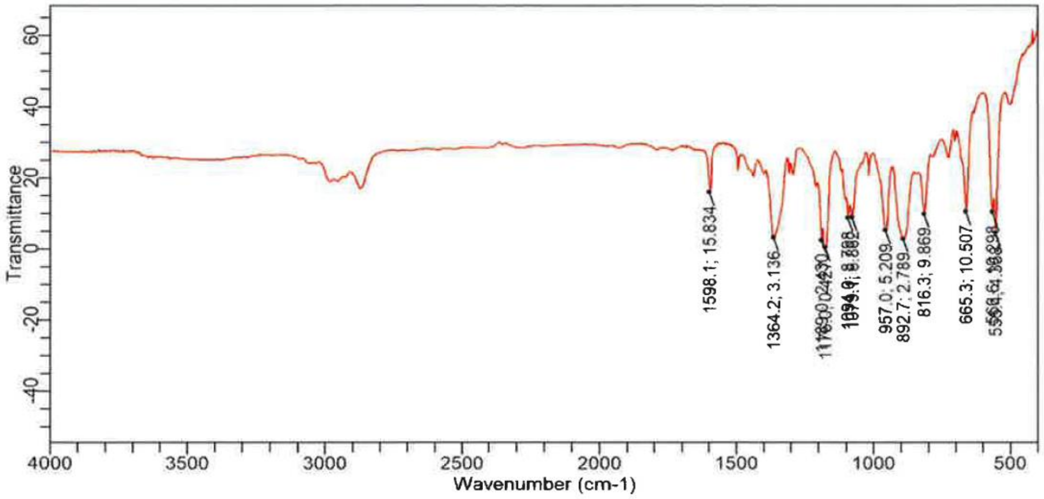

Peak Number	Wavenumber (cm-1)	Intensity
1	555.37334	4.35797
2	566.55535	10.29819
3	665.32980	10.50725
4	816.28699	9.86903
5	892.69741	2.78888

6	956.99399	5.20898
7	1079.06430	8.86190
8	1093.97366	8.79848
9	1175.97509	0.42671
10	1189.02077	2.43020
11	1364.20565	3.13606
12	1598.09610	15.83364

bio cryst

EB 21 July [illegible]

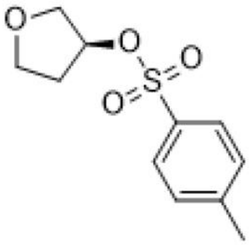

Sample ID:LCJ-7-148A

Sample Scans:32
Background Scans:32
Resolution:2
System Status:Good

Method Name:C:\Users\Public\Doc b\Methods\400-CM.a2m
User:admin
Date/Time:07/21/2023 12:4
Range:4000 - 400
Apodization:Happ-Genzel

File Location:C:\Users\Public\Documents\Agilent\MicroLab\Results\LCJ-7-148A_2023-07-21T12-40-48.a2r

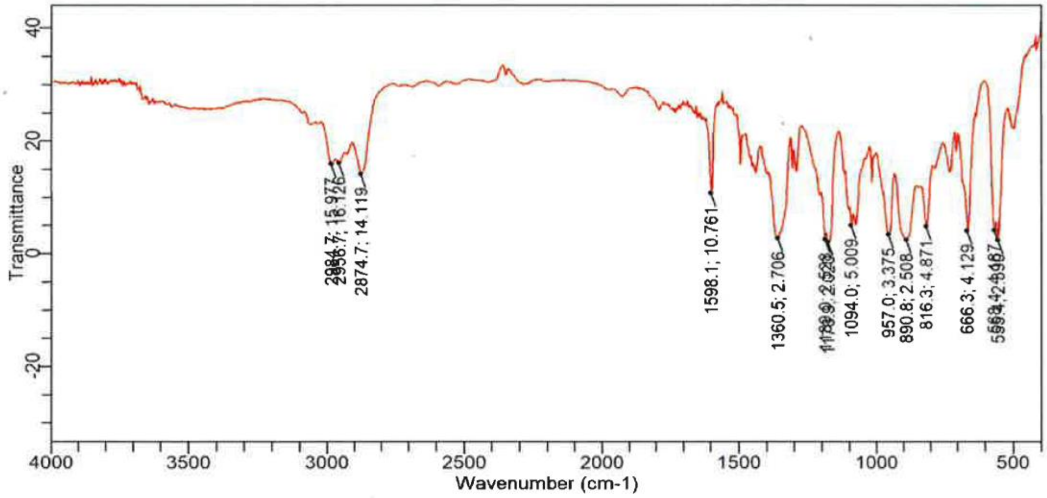

Peak Number	Wavenumber (cm^{-1})	Intensity
1	555.37334	2.39007
2	568.41902	4.18682
3	666.26164	4.12900
4	816.28699	4.87060
5	890.83374	2.50783

6	956.99399	3.37456
7	1093.97366	5.00907
8	1176.90692	2.02001
9	1189.02077	2.52788
10	1360.47831	2.70644
11	1598.09610	10.76052
12	2874.70931	14.11903
13	2956.71074	16.12599
14	2984.66577	15.97663

File Location:NAPLBBHPC2L810D\SQLEXPRESS|VAIMDB_Public(000)|Public Results\LJC-004-142A.a2r

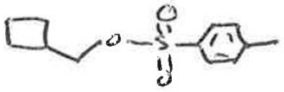

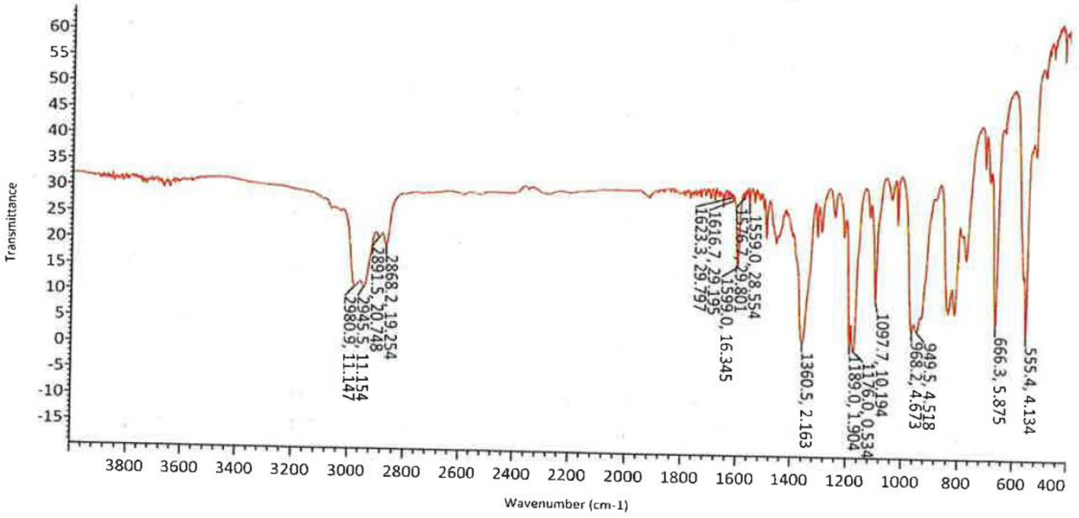

Peak Number	Wavenumber (cm^{-1})	Intensity
1	555.4	4.134
2	666.3	5.875
3	949.5	4.518
4	968.2	4.673
5	1097.7	10.194
6	1176.0	0.534
7	1189.0	1.904
8	1360.5	2.163
9	1559.0	28.554
10	1576.7	29.801
11	1599.0	16.345

12	1616.7	29.195
13	1623.3	29.797
14	2868.2	19.254
15	2891.5	20.748
16	2945.5	11.154
17	2980.9	11.147

E-SIGNATURES

Signature

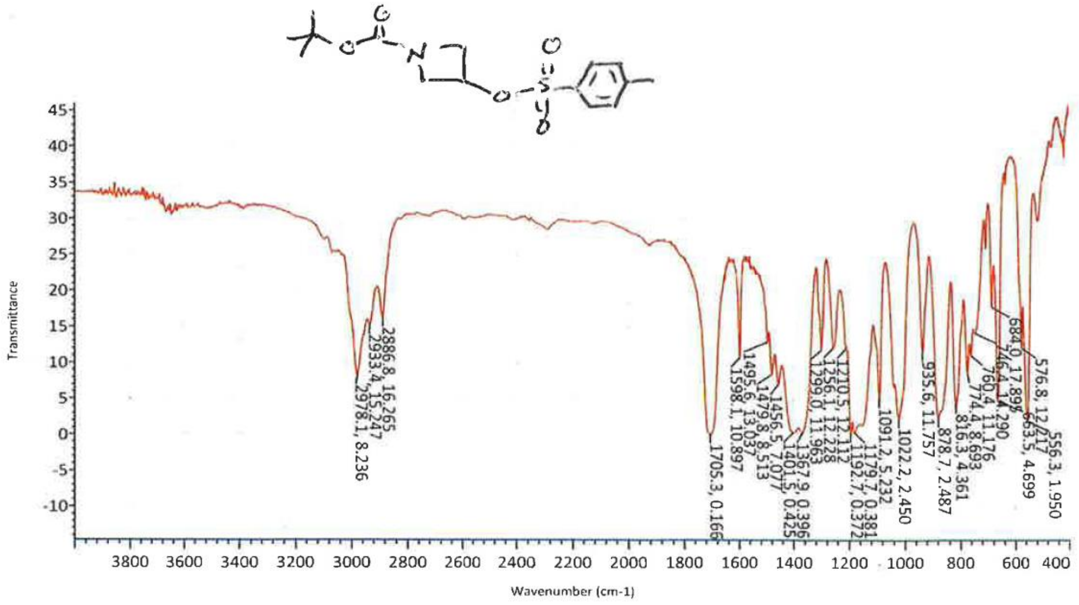

Peak Number	Wavenumber (cm^{-1})	Intensity
1	556.3	1.950
2	576.8	12.217
3	663.5	4.699
4	684.0	17.895
5	746.4	14.290
6	760.4	11.176
7	774.4	8.693
8	816.3	4.361
9	878.7	2.487
10	935.6	11.757
11	1022.2	2.450
12	1091.2	5.232

13	1179.7	0.381
14	1192.7	0.372
15	1210.5	12.112
16	1256.1	12.228
17	1299.0	11.963
18	1367.9	0.396
19	1401.5	0.425
20	1456.5	7.077
21	1479.8	8.513
22	1495.6	13.037
23	1598.1	10.897
24	1705.3	0.166
25	2886.8	16.265
26	2933.4	15.247
27	2978.1	8.236

E-SIGNATURES

Signature

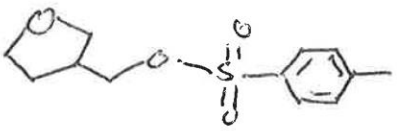

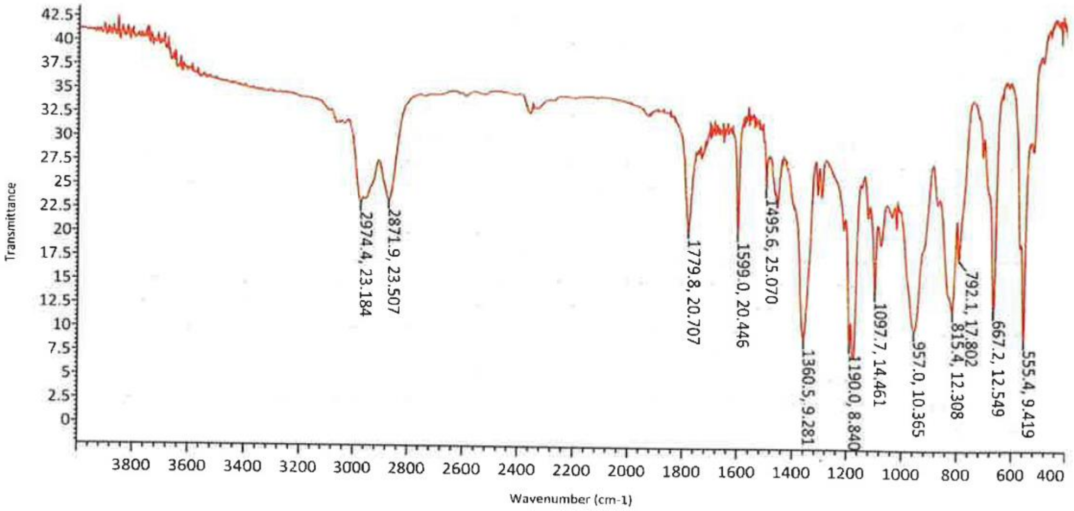

Peak Number	Wavenumber (cm^{-1})	Intensity
1	555.4	9.419
2	667.2	12.549
3	792.1	17.802
4	815.4	12.308
5	957.0	10.365
6	1097.7	14.461
7	1190.0	8.840
8	1360.5	9.281
9	1495.6	25.070
10	1599.0	20.446
11	1779.8	20.707
12	2871.9	23.507

13	2974.4	23.184

E-SIGNATURES

Signature

EB 21 July

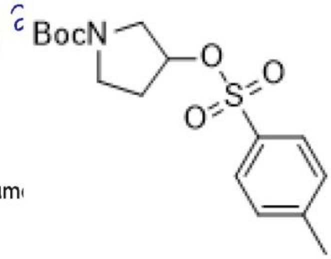

Sample ID:LCJ-7-151A

Method Name:C:\Users\Public\Docum[illegible] b\Methods\400-CM.a2m

Sample Scans:32

User:admin

Background Scans:32

Date/Time:07/21/2023 12:02:10 PM

Resolution:2

Range:4000 - 400

System Status:Good

Apodization:Happ-Genzel

File Location:C:\Users\Public\Documents\Agilent\MicroLab\Results\\LCJ-7-151A_2023-07-21T12-02-10.a2r

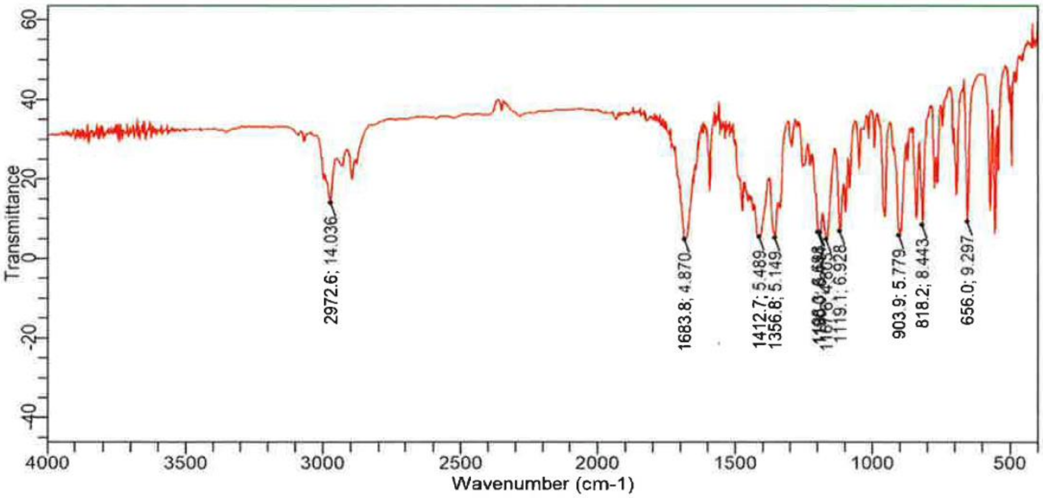

Peak Number	Wavenumber (cm^{-1})	Intensity
1	656.01146	9.29742
2	818.15066	8.44256
3	903.87943	5.77865
4	1119.13319	6.92768
5	1167.58858	4.80282

6	1189.95260	6.44371
7	1198.33911	6.69798
8	1356.75097	5.14927
9	1412.66104	5.48889
10	1683.82487	4.87042
11	2972.55193	14.03594

Sample ID:LCJ-012-145B

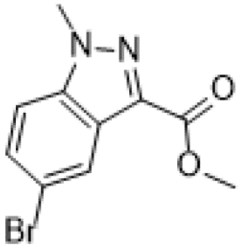

Sample Scans:128
Background Scans:32
Resolution:4
System Status:Good

Method Name:C:\Users\Public\Documents\Agilent\MicroLab\Methods\ATR 128 Scans.a2m
User:admin
Date/Time:03/13/2023 12:47:14 PM
Range:4000 - 650
Apodization:Happ-Genzel

File Location:C:\Users\Public\Documents\Agilent\MicroLab\Results\\LCJ-012-145B_2023-03-13T12-47-14.a2r

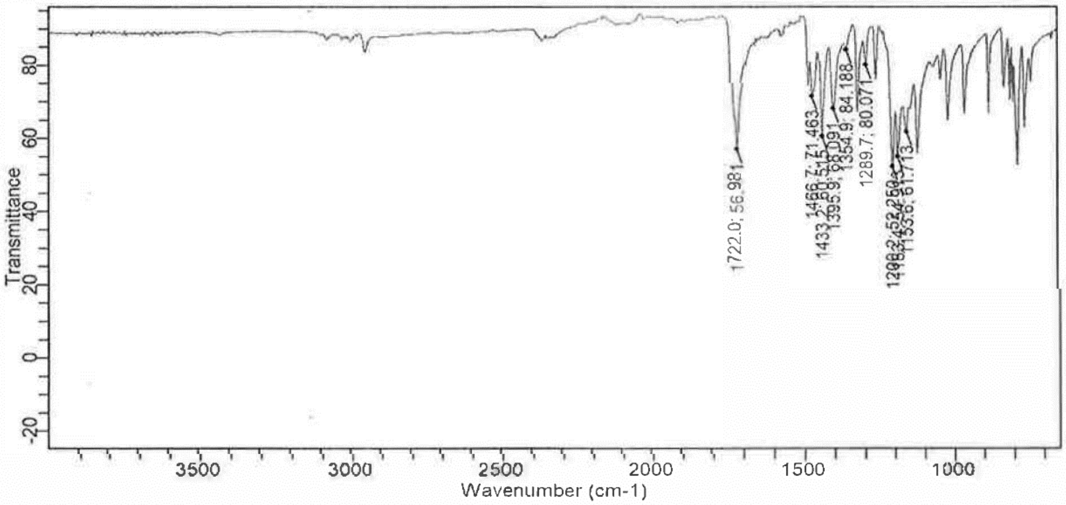

Peak Number	Wavenumber (cm^{-1})	Intensity
1	1153.61106	61.71313
2	1183.42976	54.91350
3	1200.20278	52.24999
4	1289.65889	80.07146
5	1354.88730	84.18800

6	1395.88802	68.09095
7	1433.16140	60.51481
8	1466.70744	71.46253
9	1722.03008	56.98058

Sample ID:LCJ-012-145C

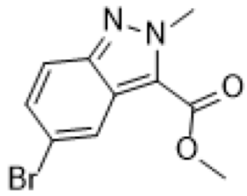

Method Name:C:\Users\Public\Documents\Agilent\MicroLab\Methods\ATR 128 Scans.a2m
User:admin

Sample Scans:128
Background Scans:32
Resolution:4
System Status:Good

Date/Time:03/13/2023 12:40:57 PM
Range:4000 - 650
Apodization:Happ-Genzel

File Location:C:\Users\Public\Documents\Agilent\MicroLab\Results\\LCJ-012-145C_2023-03-13T12-40-57.a2r

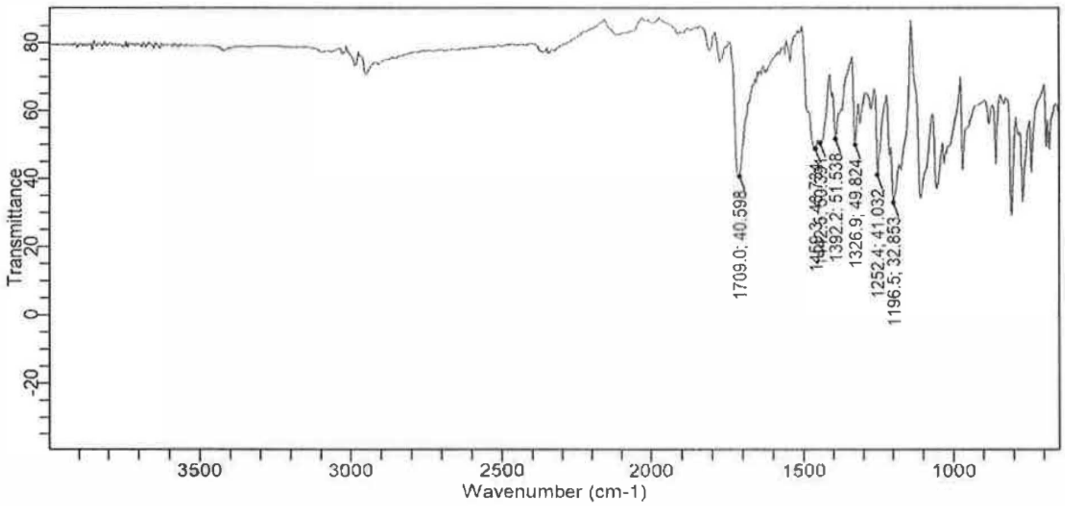

Peak Number	Wavenumber (cm^{-1})	Intensity
1	1196.47545	32.85279
2	1252.38551	41.03164
3	1326.93227	49.82378
4	1392.16068	51.53811
5	1442.47974	50.39129

6	1459.25276	48.73363
7	1708.98440	40.59753

Sample ID:LCJ-006-081B

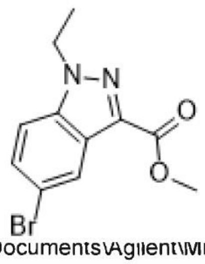

Sample Scans:128
Background Scans:32
Resolution:4
System Status:Good

Method Name:C:\Users\Public\Documents\Agilent\MicroLab\Methods\ATR 128 Scans.a2m
User:admin
Date/Time:03/13/2023 8:42:22 AM
Range:4000 - 650
Apodization:Happ-Genzel

File Location:C:\Users\Public\Documents\Agilent\MicroLab\Results\\LCJ-006-081B_2023-03-13T08-42-22.a2r

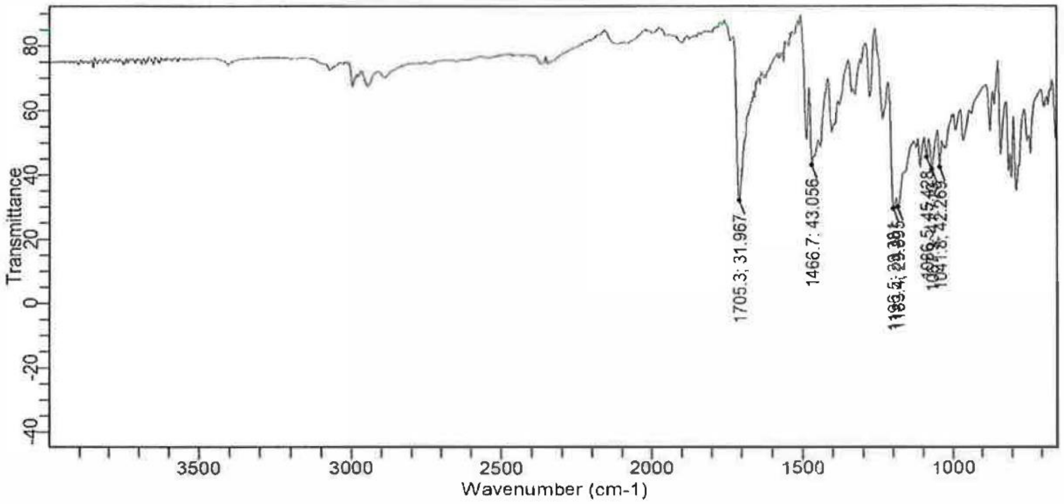

Peak Number	Wavenumber (cm^{-1})	Intensity
1	1041.79093	42.26946
2	1067.88229	41.70964
3	1086.51898	45.42796
4	1183.42976	29.89476
5	1196.47545	29.38142

6	1466.70744	43.05579
7	1705.25706	31.96682

bio cryst

Sample ID:LCJ-006-081C

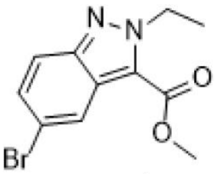

Sample Scans:128
Background Scans:32
Resolution:4
System Status:Good

Method Name:C:\Users\Public\Documents\Agilent\MicroLab\Methods\ATR 128 Scans.a2m
User:admin
Date/Time:03/13/2023 8:32:17 AM
Range:4000 - 650
Apodization:Happ-Genzel

File Location:C:\Users\Public\Documents\Agilent\MicroLab\Results\LCJ-006-081C_2023-03-13T08-32-17.a2r

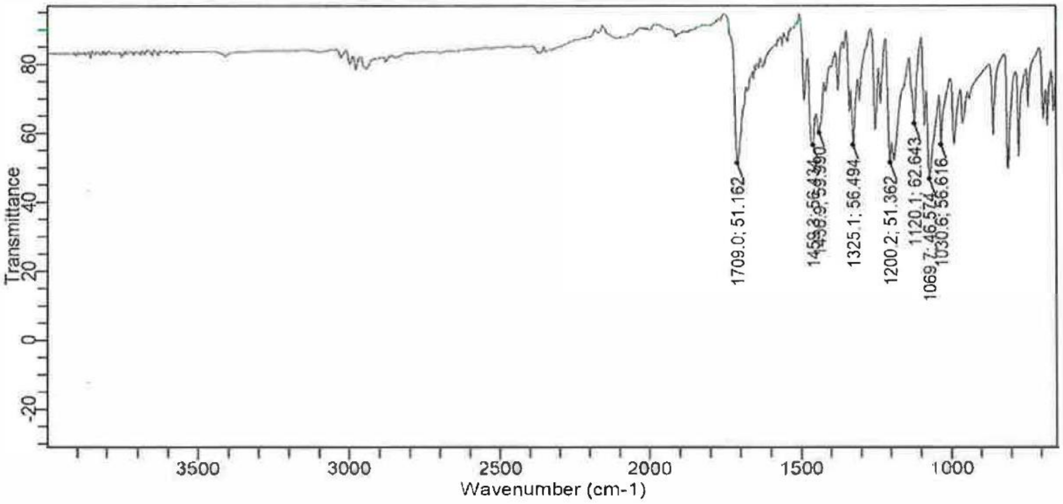

Peak Number	Wavenumber (cm⁻¹)	Intensity
1	1030.60891	56.61588
2	1069.74596	46.57442
3	1120.06502	62.64288
4	1200.20278	51.36187
5	1325.06860	56.49372

6	1436.88874	59.99048
7	1459.25276	56.43382
8	1708.98440	51.16249

bio cryst

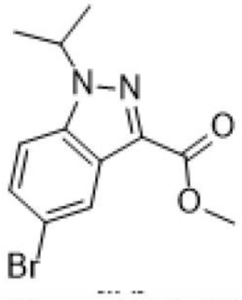

Sample ID:LCJ-011-008B

Sample Scans:128
Background Scans:32
Resolution:4
System Status:Good

Method
Name:C:\Users\Public\Documents\Agilent\MicroLab\Methods\ATR 128 Scans.a2m
User:admin
Date/Time:03/13/2023 12:33:41 PM
Range:4000 - 650
Apodization:Happ-Genzel

File Location:C:\Users\Public\Documents\Agilent\MicroLab\Results\\LCJ-011-008B_2023-03-13T12-33-41.a2r

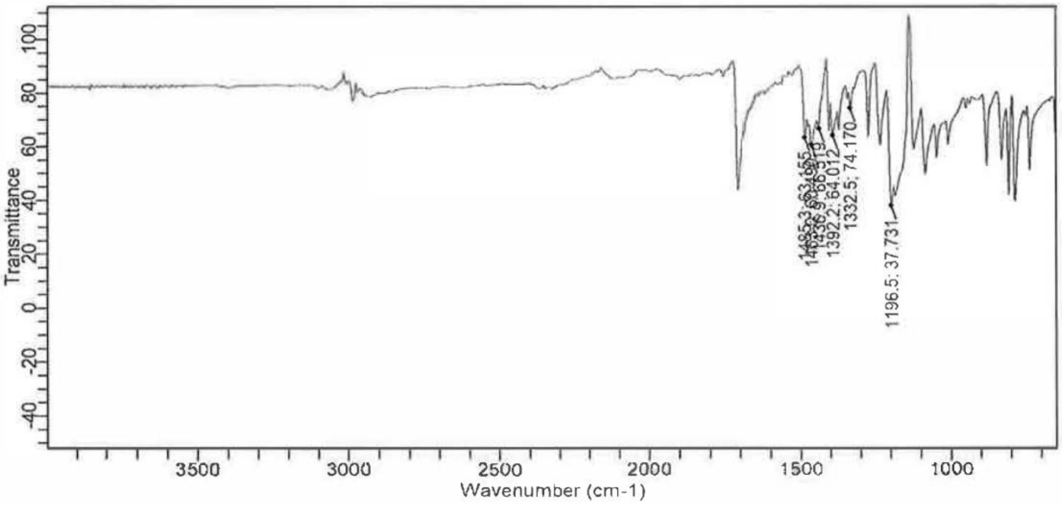

Peak Number	Wavenumber (cm⁻¹)	Intensity
1	1196.47545	37.73147
2	1332.52328	74.16989
3	1392.16068	64.01244
4	1436.88874	66.51938
5	1462.98010	60.49031

6	1485.34413	63.15505

Sample ID:LCJ-011-008C

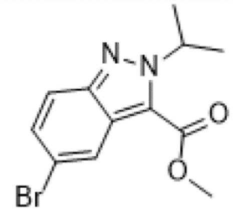

Method Name:C:\Users\Public\Documents\Agilent\MicroLab\Methods\ATR 128 Scans.a2m
User:admin

Sample Scans:128
Background Scans:32
Resolution:4
System Status:Good

Date/Time:03/13/2023 12:24:30 PM
Range:4000 - 650
Apodization:Happ-Genzel

File Location:C:\Users\Public\Documents\Agilent\MicroLab\Results\\LCJ-011-008C_2023-03-13T12-24-30.a2r

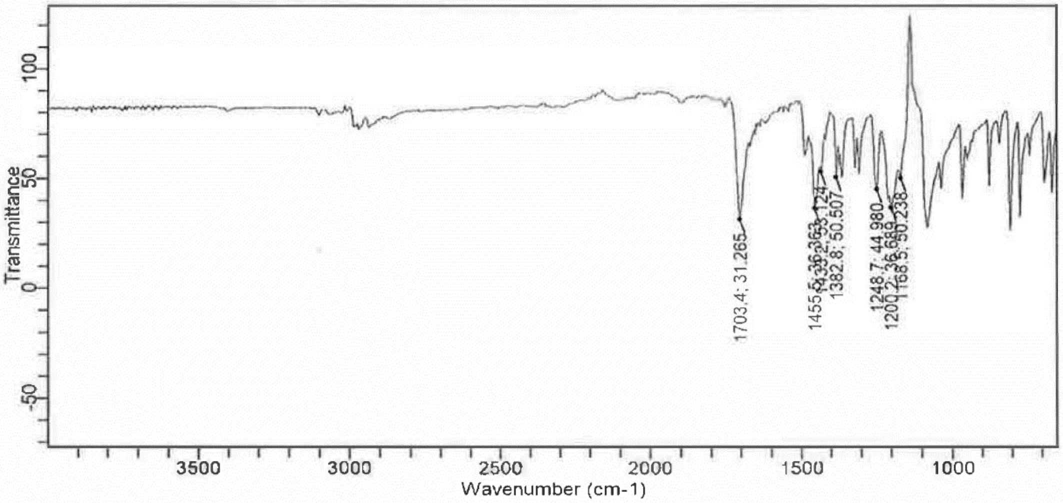

Peak Number	Wavenumber (cm^{-1})	Intensity
1	1168.52041	50.23774
2	1200.20278	36.68900
3	1248.65818	44.98000
4	1382.84234	50.50651
5	1433.16140	53.12354

6	1455.52543	36.36317
7	1703.39339	31.26469

bio cryst

Sample ID:LCJ-007-136B

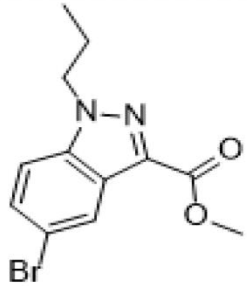

Method Name:C:\Users\Public\Documents\Agilent\MicroLab\Methods\ATR 128 Scans.a2m

Sample Scans:128

User:admin

Background Scans:32

Date/Time:03/13/2023 9:08:39 AM

Resolution:4

Range:4000 - 650

System Status:Good

Apodization:Happ-Genzel

File Location:C:\Users\Public\Documents\Agilent\MicroLab\Results\\LCJ-007-136B_2023-03-13T09-08-39.a2r

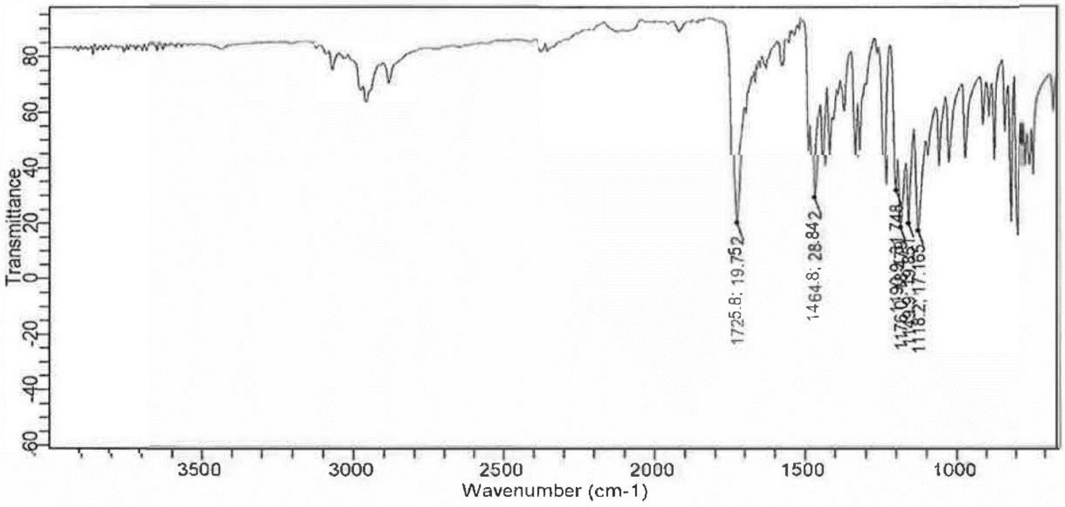

Peak Number	Wavenumber (cm^{-1})	Intensity
1	1118.20135	17.16486
2	1149.88372	19.85103
3	1175.97509	18.47925
4	1190.88444	31.74764
5	1464.84377	28.84161

6	1725.75742	19.75177

Sample ID:LCJ-007-136C

Sample Scans:128
Background Scans:32
Resolution:4
System Status:Good
File Location:C:\Users\Pul
42.a2r

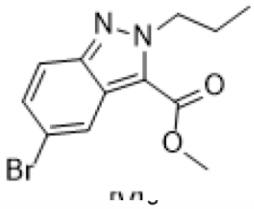

Method
Name:C:\Users\Public\Documents\Agilent\MicroLa
\Methods\ATR 128 Scans.a2m
User:admin
Date/Time:03/13/2023 8:57:42 AM
Range:4000 - 650
Apodization:Happ-Genzel
Lab\Results\\LCJ-007-136C_2023-03-13T08-57-

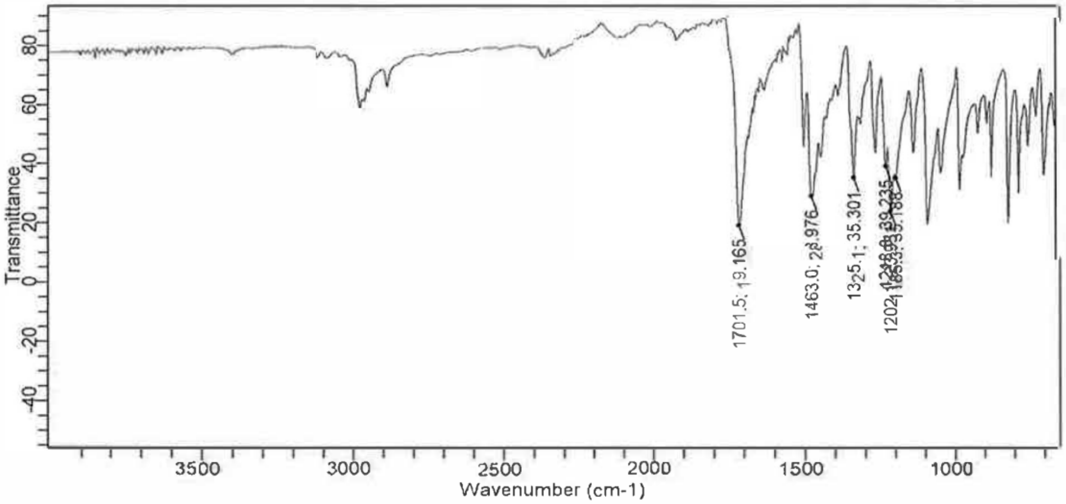

Peak Number	Wavenumber (cm-1)	Intensity
1	1185.29343	35.18787
2	1202.06645	23.93112
3	1218.83947	39.23543
4	1325.06860	35.30121
5	1462.98010	28.97583

6	1701.52972	19.16455

Sample ID:LCJ-007-152B

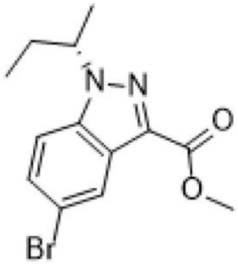

Method Name:C:\Users\Public\Documents\Agilent\MicroLab\Methods\ATR 128 Scans.a2m

Sample Scans:128
User:admin
Background Scans:32
Date/Time:03/13/2023 9:59:12 AM
Resolution:4
Range:4000 - 650
System Status:Good
Apodization:Happ-Genzel
File Location:C:\Users\Public\Documents\Agilent\MicroLab\Results\\LCJ-007-152B_2023-03-13T09-59-12.a2r

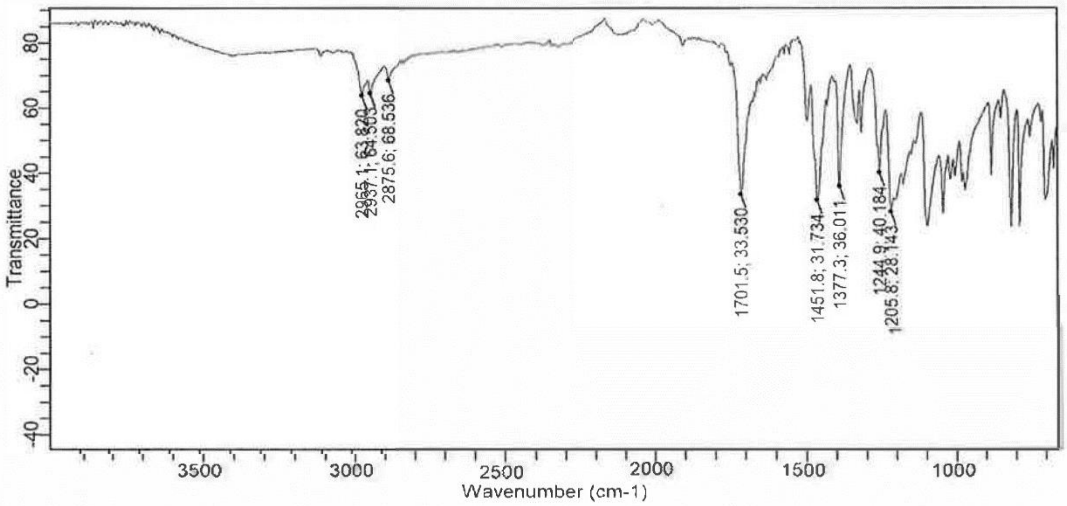

Peak Number	Wavenumber (cm^{-1})	Intensity
1	1205.79379	28.14339
2	1244.93084	40.18409
3	1377.25133	36.01087
4	1451.79809	31.73383
5	1701.52972	33.53023

6	2875.64114	68.53643
7	2937.14222	64.50303
8	2965.09725	63.81967

Sample ID:LCJ-007-152C

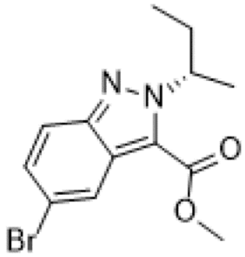

Sample Scans:32
Background Scans:32
Resolution:2
System Status:Good

Method Name:C:\Users\Public\Documents\Agilent\MicroLab\Methods\400-CM.a2m
User:admin
Date/Time:03/13/2023 1:29:08 PM
Range:4000 - 400
Apodization:Happ-Genzel

File Location:C:\Users\Public\Documents\Agilent\MicroLab\Results\\LCJ-007-152C_2023-03-13T13-29-08.a2r

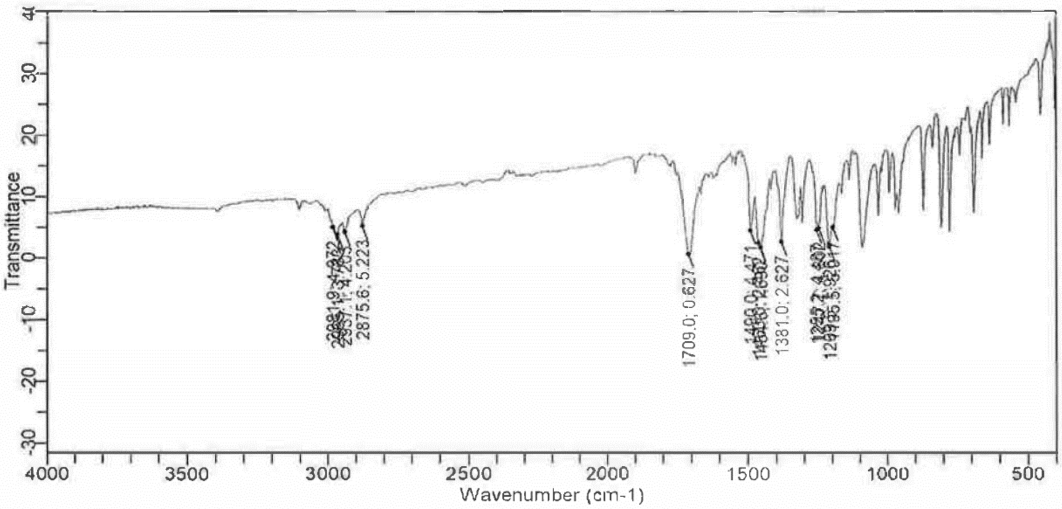

Peak Number	Wavenumber (cm-1)	Intensity
1	1195.54361	5.01697
2	1209.52113	1.92555
3	1247.72634	4.80237
4	1255.18102	4.48735
5	1380.97867	2.62699

6	1454.59359	1.68977
7	1462.98010	2.46233
8	1490.00330	4.47055
9	1708.98440	0.62685
10	2875.64114	5.22327
11	2937.14222	4.20273
12	2965.09725	3.78335
13	2981.87027	4.97185

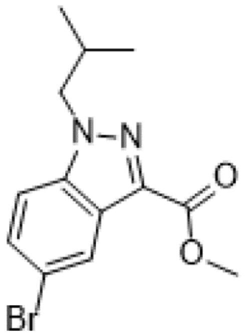

Sample ID:LCJ-007-154B

Sample Scans:32
Background Scans:32
Resolution:2
System Status:Good

Method Name:C:\Users\Public\Documents\Agilent\MicroLab\Methods\400-CM.a2m
User:admin
Date/Time:03/13/2023 1:38:35 PM
Range:4000 - 400
Apodization:Happ-Genzel

File Location:C:\Users\Public\Documents\Agilent\MicroLab\Results\\LCJ-007-154B_2023-03-13T13-38-35.a2r

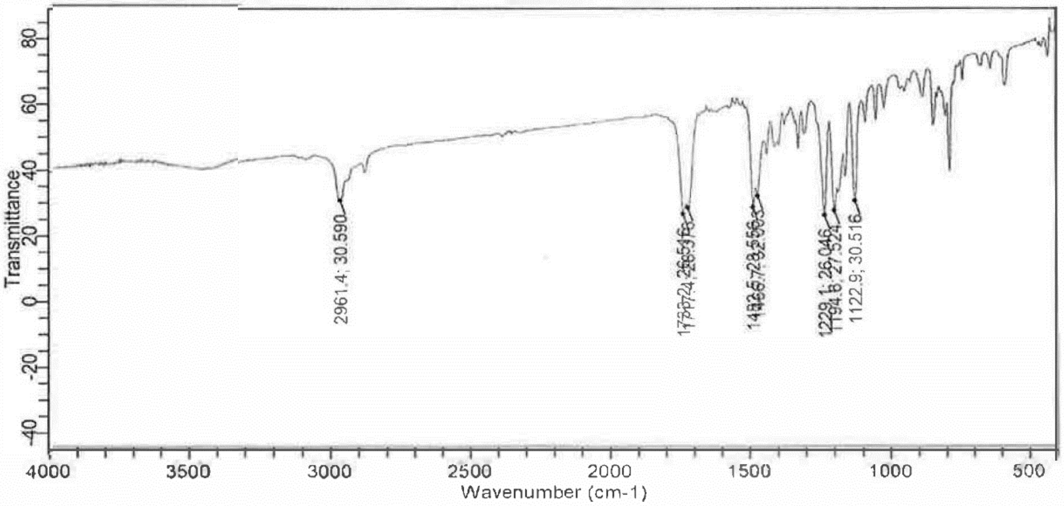

Peak Number	Wavenumber (cm^{-1})	Intensity
1	1122.86052	30.51611
2	1194.61178	27.52388
3	1229.08965	26.04618
4	1466.70744	32.00286
5	1482.54862	28.55584

6	1717.37091	28.37635
7	1733.21209	26.51607
8	2961.36991	30.58969

Sample ID:LCJ-007-154C

Sample Scans:128
Background Scans:32
Resolution:4
System Status:Good

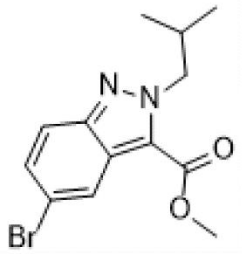

Method Name:C:\Users\Public\Documents\Agilent\MicroLab\Methods\ATR 128 Scans.a2m
User:admin
Date/Time:03/13/2023 10:12:54 AM
Range:4000 - 650
Apodization:Happ-Genzel

File Location:C:\Users\Public\Documents\Agilent\MicroLab\Results\\LCJ-007-154C_2023-03-13T10-12-54.a2r

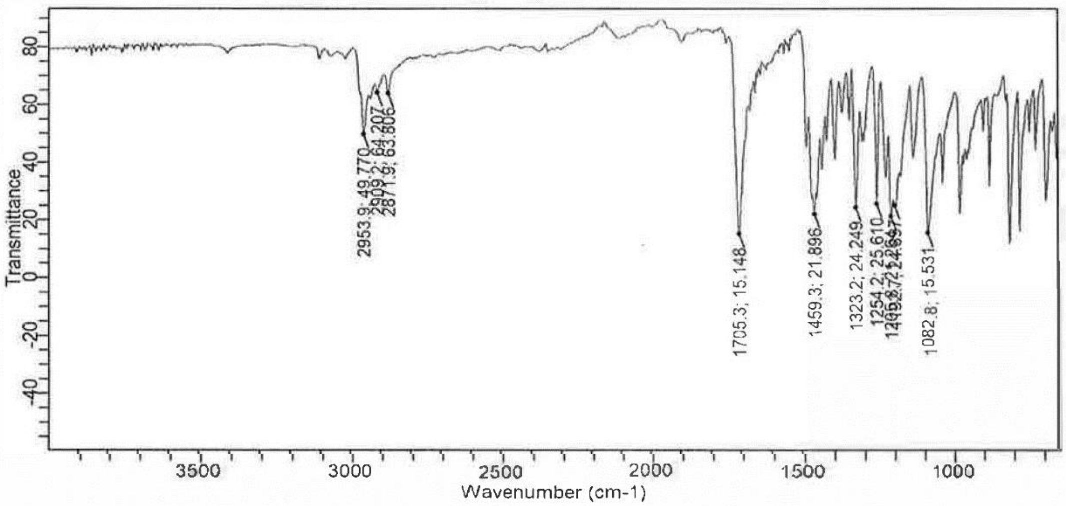

Peak Number	Wavenumber (cm⁻¹)	Intensity
1	1082.79164	15.53131
2	1192.74811	24.89689
3	1205.79379	21.26395
4	1254.24918	25.61045
5	1323.20493	24.24895

6	1459.25276	21.89565
7	1705.25706	15.14805
8	2871.91380	63.80600
9	2909.18718	64.20744
10	2953.91524	49.76992

Sample ID:LCJ-007-143B

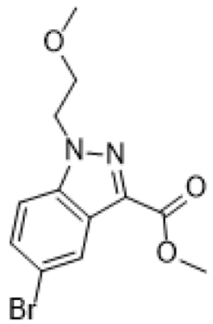

Sample Scans:128
Background Scans:32
Resolution:4
System Status:Good

Method Name:C:\Users\Public\Documents\Agilent\MicroLab\Methods\ATR 128 Scans.a2m
User:admin
Date/Time:03/13/2023 12:37:53 PM
Range:4000 - 650
Apodization:Happ-Genzel

File Location:C:\Users\Public\Documents\Agilent\MicroLab\Results\\LCJ-007-143B_2023-03-13T12-37-53.a2r

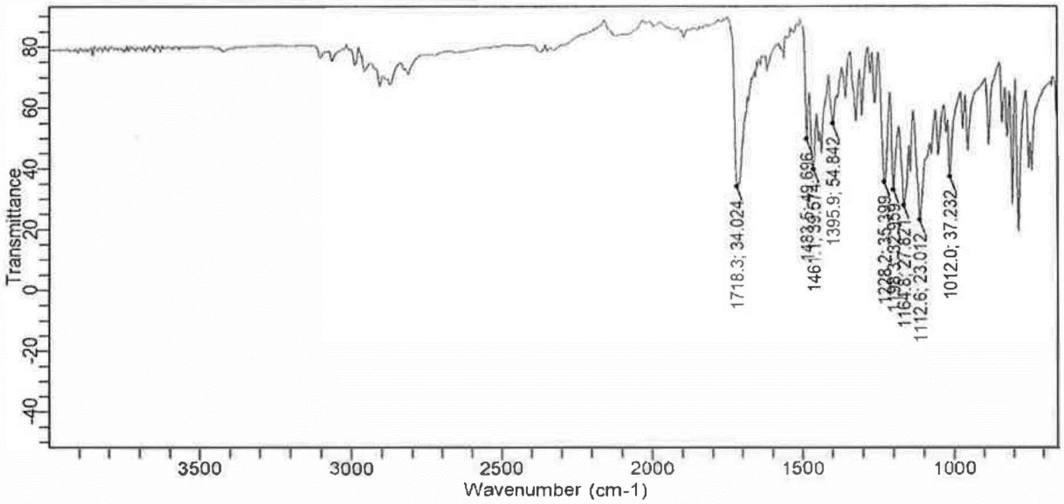

Peak Number	Wavenumber (cm^{-1})	Intensity
1	1011.97222	37.23186
2	1112.61034	23.01163
3	1164.79307	27.82095
4	1198.33911	32.95888
5	1228.15782	35.39860

6	1395.88802	54.84167
7	1461.11643	39.57371
8	1483.48046	49.69588
9	1718.30274	34.02357

Sample ID:LCJ-007-143C

Sample Scans:128
Background Scans:32
Resolution:4
System Status:Good

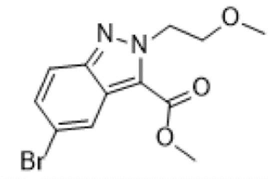

Method Name:C:\Users\Public\Documents\Agilent\MicroLab\Methods\ATR 128 Scans.a2m
User:admin
Date/Time:03/13/2023 12:27:27 PM
Range:4000 - 650
Apodization:Happ-Genzel

File Location:C:\Users\Public\Documents\Agilent\MicroLab\Results\\LCJ-007-143C_2023-03-13T12-27-27.a2r

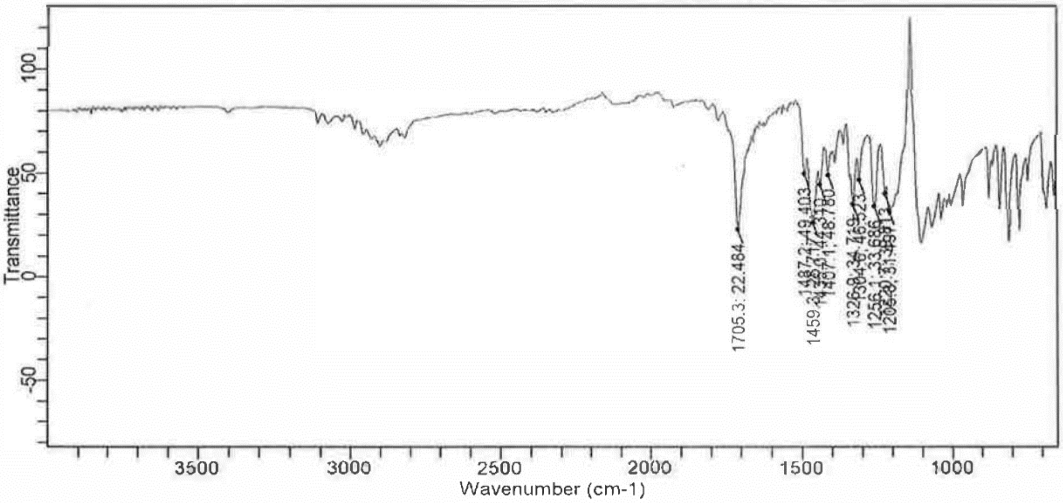

Peak Number	Wavenumber (cm^{-1})	Intensity
1	1205.79379	31.49663
2	1220.70314	39.81252
3	1256.11285	33.68586
4	1304.56824	46.52315
5	1326.93227	34.71922

6	1407.07003	48.77999
7	1435.02507	44.30999
8	1459.25276	25.71714
9	1487.20780	49.40263
10	1705.25706	22.48364

Sample ID:LCJ-007-140B

Sample Scans:128
Background Scans:32
Resolution:4
System Status:Good

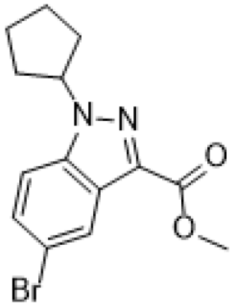

Method Name:C:\Users\Public\Documents\Agilent\MicroLab\Methods\ATR 128 Scans.a2m
User:admin
Date/Time:03/13/2023 10:07:46 AM
Range:4000 - 650
Apodization:Happ-Genzel

File Location:C:\Users\Public\Documents\Agilent\MicroLab\Results\\LCJ-007-140B_2023-03-13T10-07-46.a2r

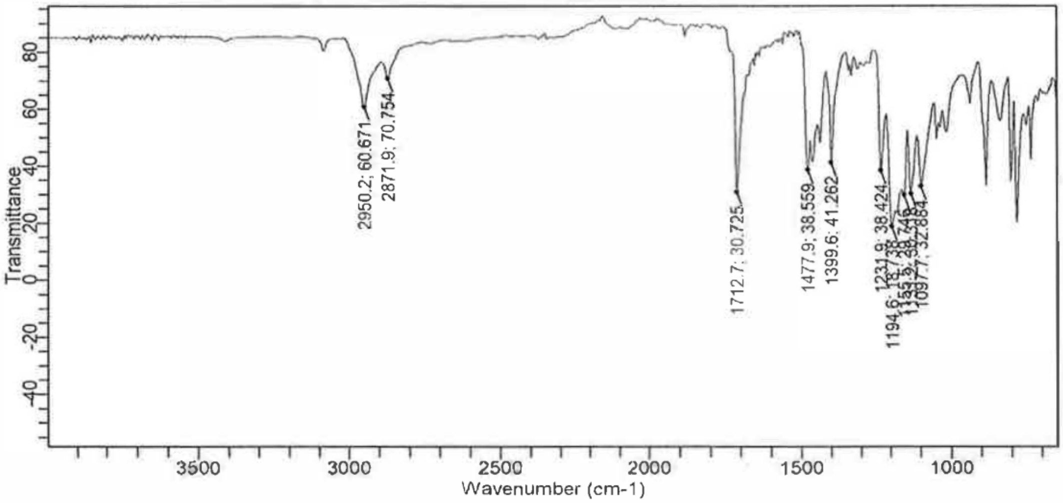

Peak Number	Wavenumber (cm^{-1})	Intensity
1	1097.70099	32.88424
2	1131.24703	30.31772
3	1155.47473	29.74621
4	1194.61178	18.73827
5	1231.88516	38.42359

6	1399.61536	41.26165
7	1477.88945	38.55933
8	1712.71174	30.72533
9	2871.91380	70.75439
10	2950.18790	60.67135

Sample ID:LCJ-007-140C

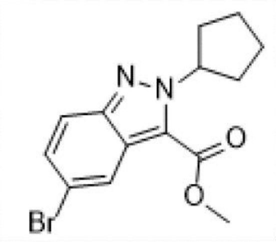

Method Name:C:\Users\Public\Documents\Agilent\MicroLab\Methods\400-CM.a2m

Sample Scans:32
Background Scans:32
Resolution:2
System Status:Good

User:admin
Date/Time:03/13/2023 1:34:08 PM
Range:4000 - 400
Apodization:Happ-Genzel

File Location:C:\Users\Public\Documents\Agilent\MicroLab\Results\\LCJ-007-140C_2023-03-13T13-34-08.a2r

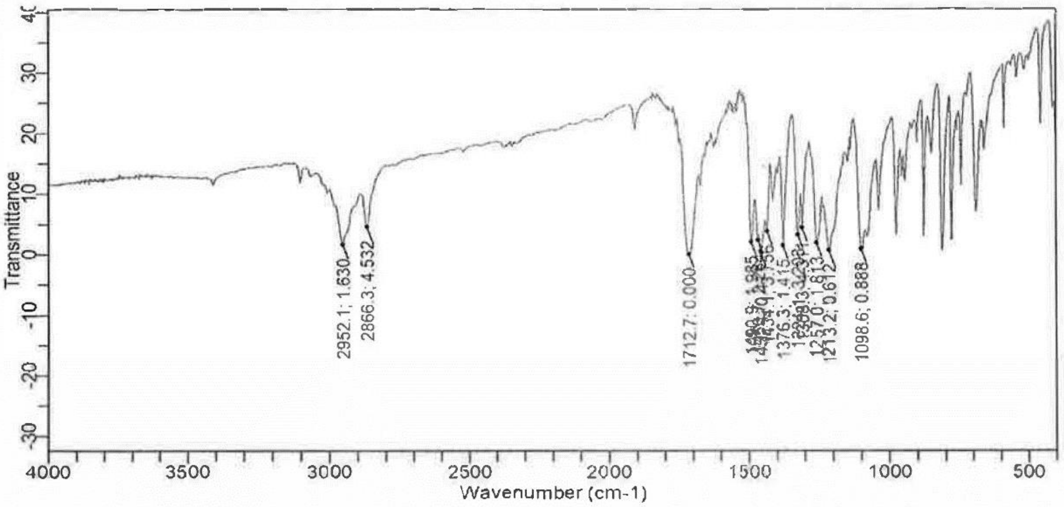

Peak Number	Wavenumber (cm^{-1})	Intensity
1	1098.63283	0.88798
2	1213.24847	0.61244
3	1257.04469	1.81301
4	1308.29558	4.31137
5	1324.13677	3.20328

6	1376.31950	1.41537
7	1434.09323	3.75614
8	1457.38909	0.41081
9	1466.70744	2.28432
10	1490.93513	1.98516
11	1712.71174	0.00001
12	2866.32280	4.53191
13	2952.05157	1.63012

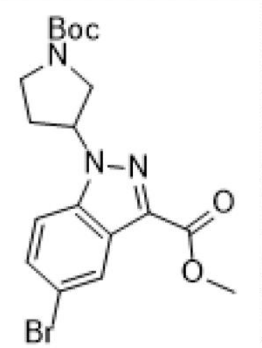

Sample ID:LCJ-007-151B

Method Name:C:\Users\Public\Documents\Agilent\MicroLab\Methods\400-CM.a2m

Sample Scans:32

User:admin

Background Scans:32

Date/Time:03/13/2023 1:13:03 PM

Resolution:2

Range:4000 - 400

System Status:Good

Apodization:Happ-Genzel

File Location:C:\Users\Public\Documents\Agilent\MicroLab\Results\\LCJ-007-151B_2023-03-13T13-13-03.a2r

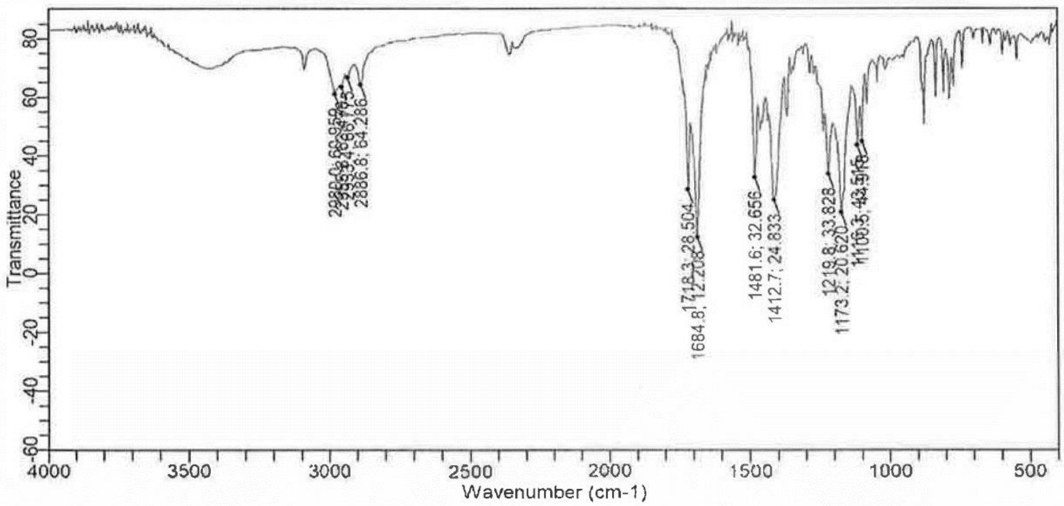

Peak Number	Wavenumber (cm^{-1})	Intensity
1	1100.49650	44.91764
2	1116.33768	43.51476
3	1173.17958	20.61996
4	1219.77131	33.82786
5	1412.66104	24.83296

6	1481.61679	32.65622
7	1684.75670	12.20782
8	1718.30274	28.50403
9	2886.82315	64.28613
10	2933.41488	66.77500
11	2955.77890	63.47835
12	2980.00660	60.95887

Sample ID:LCJ-007-151C

Sample Scans:128
Background Scans:32
Resolution:4
System Status:Good

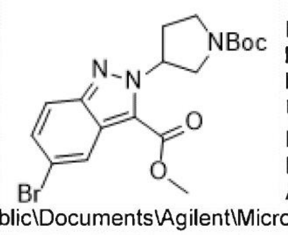

Method Name:C:\Users\Public\Documents\Agilent\MicroLab\Methods\ATR 128 Scans.a2m
User:admin
Date/Time:03/13/2023 9:13:52 AM
Range:4000 - 650
Apodization:Happ-Genzel

File Location:C:\Users\Public\Documents\Agilent\MicroLab\Results\\LCJ-007-151C_2023-03-13T09-13-52.a2r

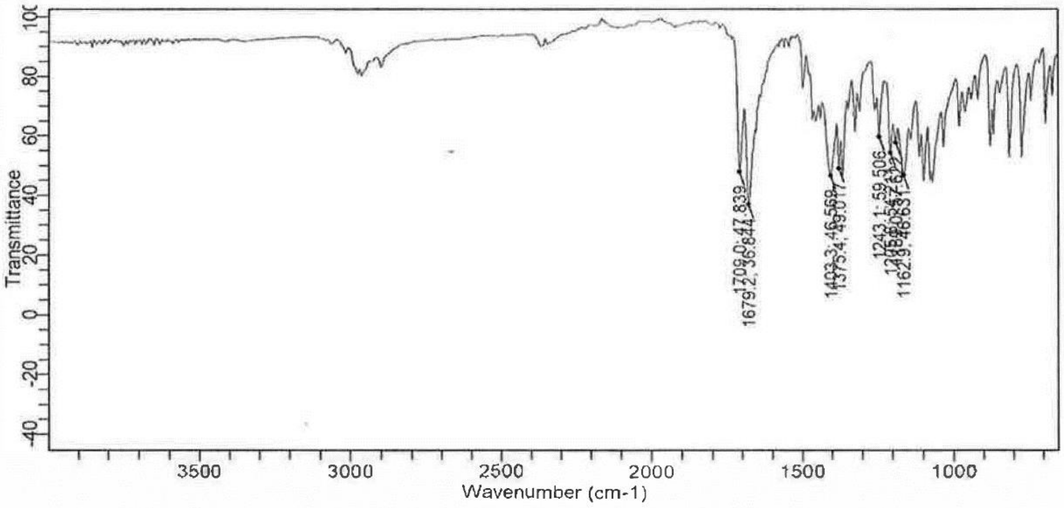

Peak Number	Wavenumber (cm^{-1})	Intensity
1	1162.92941	46.63055
2	1189.02077	57.62245
3	1205.79379	54.21115
4	1243.06717	59.50648
5	1375.38766	49.01651

6	1403.34270	46.56858
7	1679.16570	36.84422
8	1708.98440	47.83945

biocryst

Sample ID:LCJ-007-147C

Sample Scans:128
Background Scans:32
Resolution:4
System Status:Good

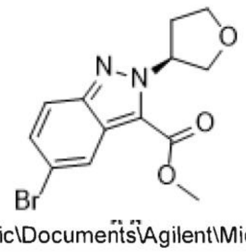

Method
Name:C:\Users\Public\Documents\Agilent\MicroLab\Methods\ATR 128 Scans.a2m
User:admin
Date/Time:03/13/2023 8:24:14 AM
Range:4000 - 650
Apodization:Happ-Genzel

File Location:C:\Users\Public\Documents\Agilent\MicroLab\Results\LCJ-007-147C_2023-03-13T08-24-14.a2r

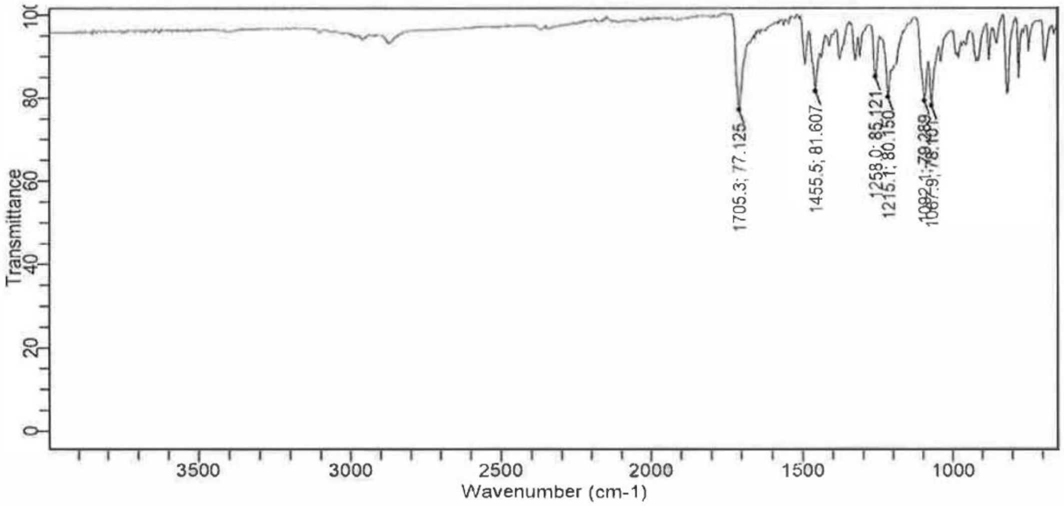

Peak Number	Wavenumber (cm^{-1})	Intensity
1	1067.88229	78.10095
2	1092.10999	79.28906
3	1215.11213	80.15043
4	1257.97652	85.12144
5	1455.52543	81.60687

6	1705.25706	77.12483

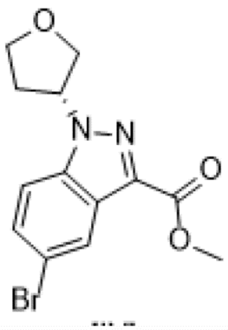

Sample ID:LCJ-007-148B

Sample Scans:128
Background Scans:32
Resolution:4
System Status:Good

Method
Name:C:\Users\Public\Documents\Agilent\MicroLab\Methods\ATR 128 Scans.a2m
User:admin
Date/Time:03/13/2023 9:04:38 AM
Range:4000 - 650
Apodization:Happ-Genzel

File Location:C:\Users\Public\Documents\Agilent\MicroLab\Results\\LCJ-007-148B_2023-03-13T09-04-38.a2r

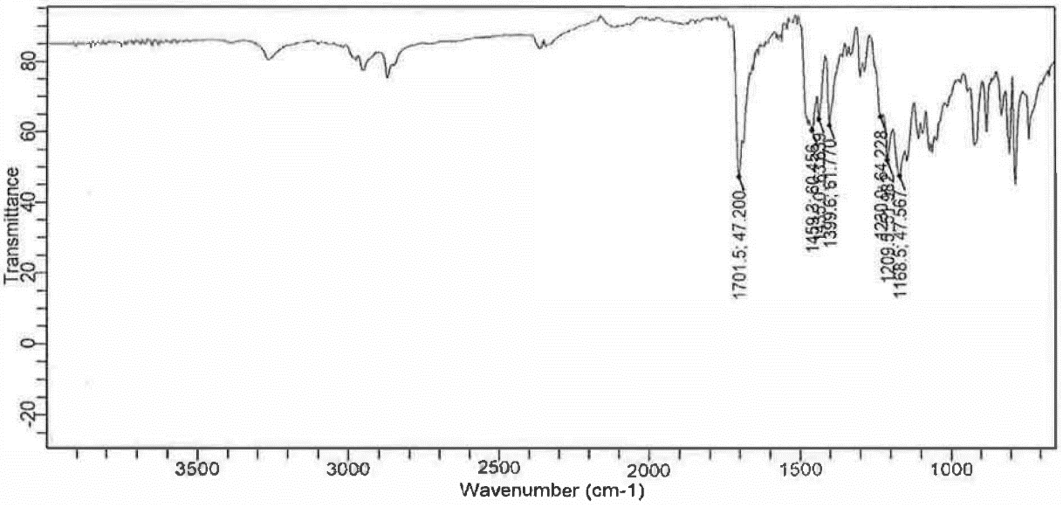

Peak Number	Wavenumber (cm⁻¹)	Intensity
1	1168.52041	47.56692
2	1209.52113	51.98202
3	1230.02149	64.22830
4	1399.61536	61.77011
5	1435.02507	63.63927

6	1459.25276	60.45590
7	1701.52972	47.20011

Sample ID:LCJ-007-148C

Sample Scans:128
Background Scans:32
Resolution:4
System Status:Good

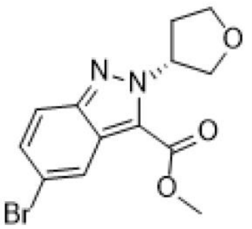

Method Name:C:\Users\Public\Documents\Agilent\MicroLab\Methods\ATR 128 Scans.a2m
User:admin
Date/Time:03/13/2023 8:54:01 AM
Range:4000 - 650
Apodization:Happ-Genzel

File Location:C:\Users\Public\Documents\Agilent\MicroLab\Results\LCJ-007-148C_2023-03-13T08-54-01.a2r

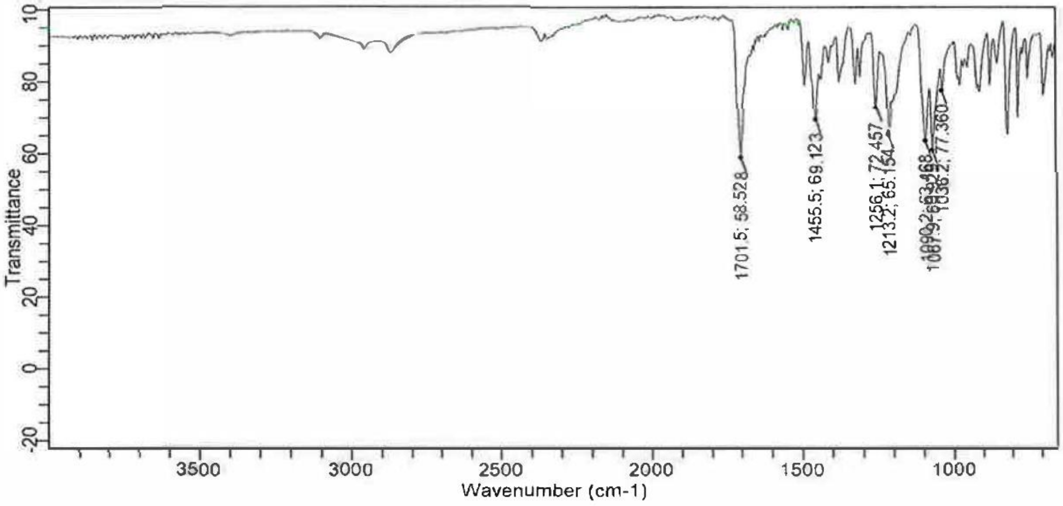

Peak Number	Wavenumber (cm^{-1})	Intensity
1	1036.19992	77.35991
2	1067.88229	60.92893
3	1090.24632	63.46799
4	1213.24847	65.15430
5	1256.11285	72.45704

6	1455.52543	69.12253
7	1701.52972	58.52757

Sample ID:LCJ-007-142B

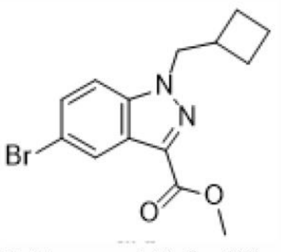

Sample Scans:128
Background Scans:32
Resolution:4
System Status:Good

Method Name:C:\Users\Public\Documents\Agilent\MicroLab\Methods\ATR 128 Scans.a2m
User:admin
Date/Time:03/13/2023 12:06:34 PM
Range:4000 - 650
Apodization:Happ-Genzel

File Location:C:\Users\Public\Documents\Agilent\MicroLab\Results\\LCJ-007-142B_2023-03-13T12-06-34.a2r

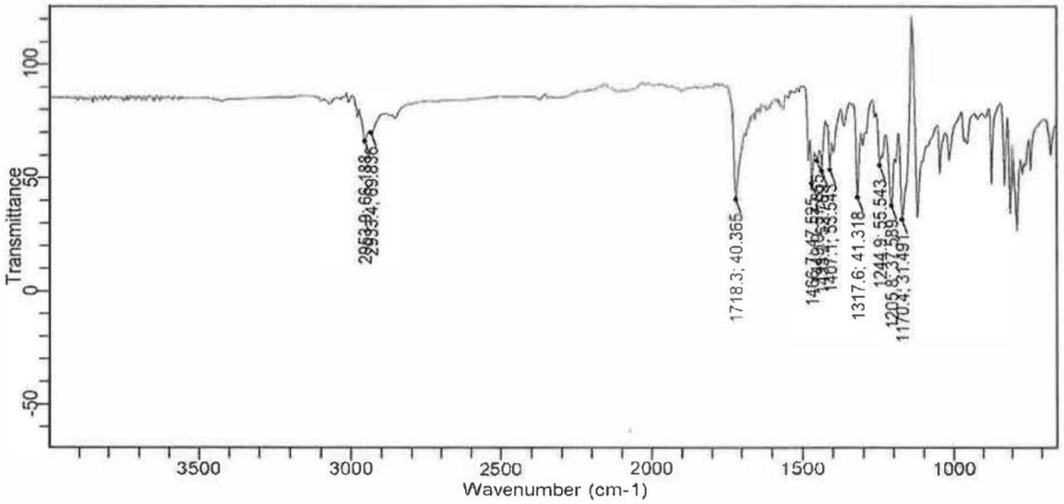

Peak Number	Wavenumber (cm^{-1})	Intensity
1	1170.38408	31.49126
2	1205.79379	37.58863
3	1244.93084	55.54266
4	1317.61393	41.31809
5	1407.07003	53.54253

6	1433.16140	52.76884
7	1449.93442	57.65510
8	1466.70744	47.52462
9	1718.30274	40.36483
10	2933.41488	69.83553
11	2953.91524	66.18779

Sample ID:LCJ-007-142C

Sample Scans:128
Background Scans:32
Resolution:4
System Status:Good

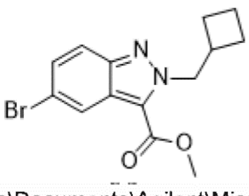

Method Name:C:\Users\Public\Documents\Agilent\MicroLab\Methods\ATR 128 Scans.a2m
User:admin
Date/Time:03/13/2023 12:00:08 PM
Range:4000 - 650
Apodization:Happ-Genzel

File Location:C:\Users\Public\Documents\Agilent\MicroLab\Results\\LCJ-007-142C_2023-03-13T12-00-08.a2r

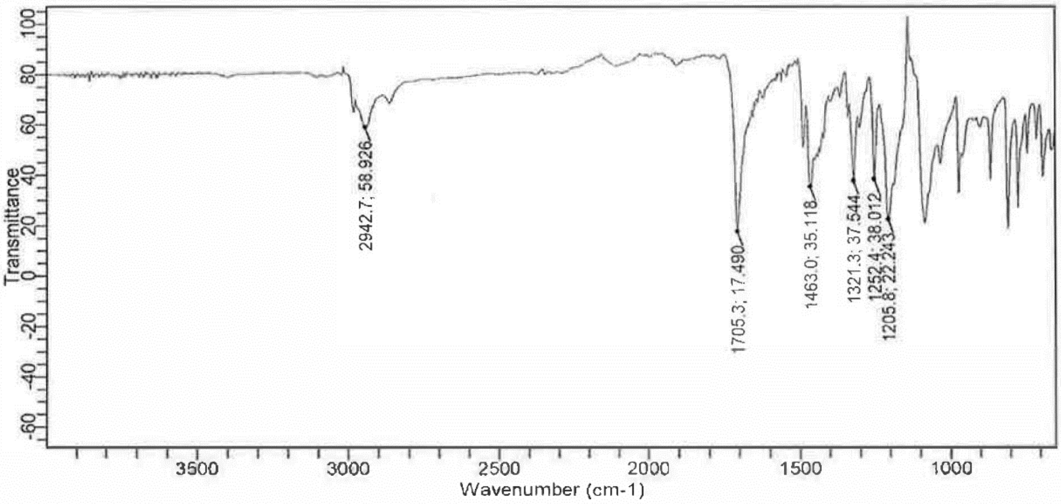

Peak Number	Wavenumber (cm^{-1})	Intensity
1	1205.79379	22.24316
2	1252.38551	38.01169
3	1321.34126	37.54367
4	1462.98010	35.11781
5	1705.25706	17.49019

6	2942.73322	58.92565

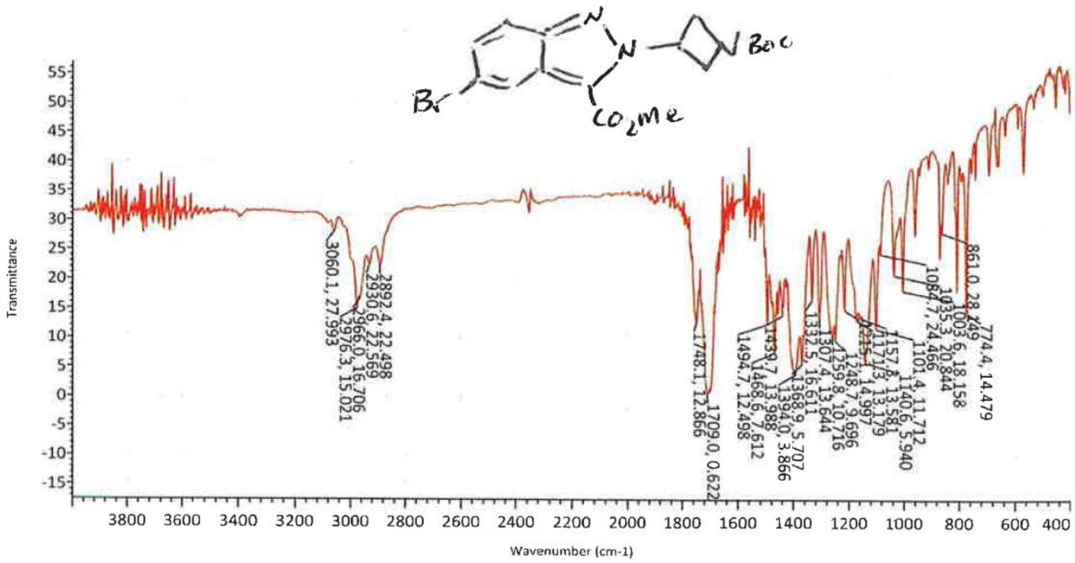

Peak Number	Wavenumber (cm^{-1})	Intensity
1	774.4	14.479
2	861.0	28.149
3	1003.6	18.158
4	1035.3	20.844
5	1084.7	24.466
6	1101.4	11.712
7	1140.6	5.940
8	1157.3	13.581
9	1171.3	13.179
10	1215.1	14.997
11	1248.7	9.696
12	1259.8	10.716

13	1307.4	13.644
14	1332.5	16.611
15	1368.9	5.707
16	1394.0	3.866
17	1439.7	13.988
18	1468.6	7.612
19	1494.7	12.498
20	1709.0	0.622
21	1748.1	12.866
22	2892.4	22.498
23	2930.6	22.569
24	2966.0	16.706
25	2976.3	15.021
26	3060.1	27.993

E-SIGNATURES

Signature

Sample ID:LCJ-007-141B

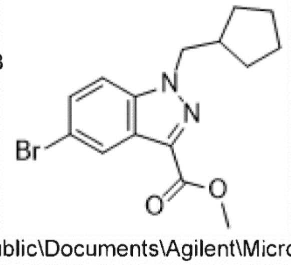

Sample Scans:128
Background Scans:32
Resolution:4
System Status:Good

Method Name:C:\Users\Public\Documents\Agilent\MicroLab\Methods\ATR 128 Scans.a2m
User:admin
Date/Time:03/13/2023 11:52:24 AM
Range:4000 - 650
Apodization:Happ-Genzel

File Location:C:\Users\Public\Documents\Agilent\MicroLab\Results\\LCJ-007-141B_2023-03-13T11-52-24.a2r

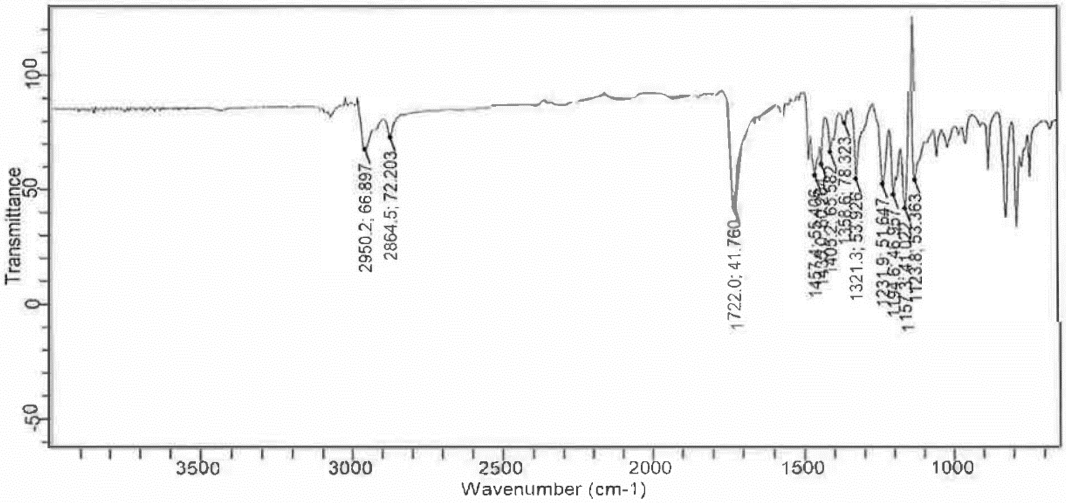

Peak Number	Wavenumber (cm^{-1})	Intensity
1	1123.79236	53.36288
2	1157.33840	41.02159
3	1194.61178	46.95675
4	1231.88516	51.64723
5	1321.34126	53.92642

6	1358.61464	78.32318
7	1405.20636	65.58237
8	1435.02507	60.26444
9	1457.38909	55.40582
10	1722.03008	41.75959
11	2864.45913	72.20255
12	2950.18790	66.89715

Sample ID:LCJ-007-141C

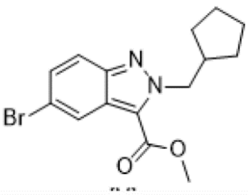

Sample Scans:128
Background Scans:32
Resolution:4
System Status:Good

Method Name:C:\Users\Public\Documents\Agilent\MicroLab\Methods\ATR 128 Scans.a2m
User:admin
Date/Time:03/13/2023 11:48:38 AM
Range:4000 - 650
Apodization:Happ-Genzel

File Location:C:\Users\Public\Documents\Agilent\MicroLab\Results\\LCJ-007-141C_2023-03-13T11-48-38.a2r

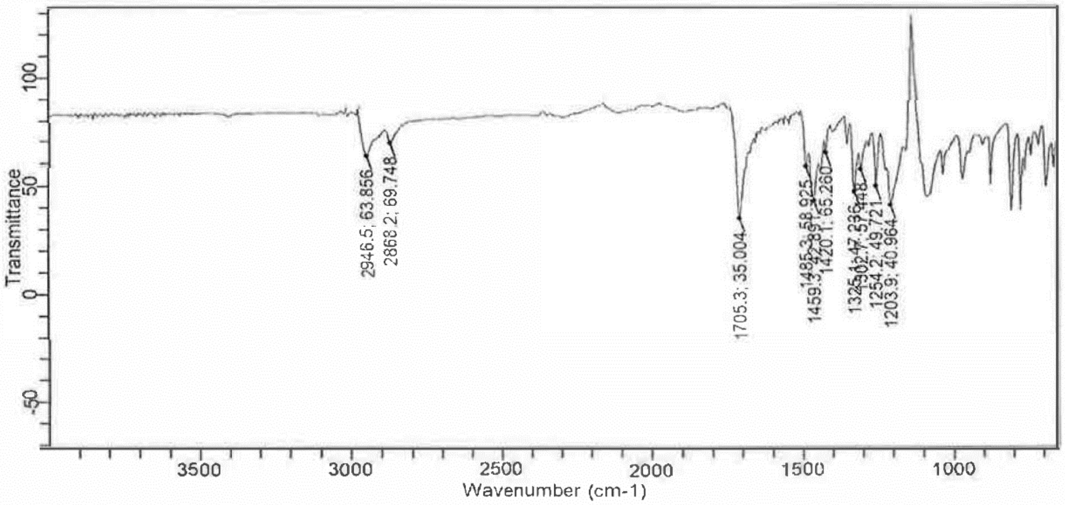

Peak Number	Wavenumber (cm^{-1})	Intensity
1	1203.93012	40.96397
2	1254.24918	49.72099
3	1302.70457	57.44787
4	1325.06860	47.23572
5	1420.11572	65.26002

6	1459.25276	42.89111
7	1485.34413	58.92480
8	1705.25706	35.00428
9	2868.18647	69.74761
10	2946.46056	63.85648

Sample ID:LCJ-007-146B

Sample Scans:32
Background Scans:32
Resolution:2
System Status:Good

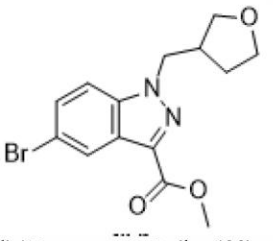

Method
Name:C:\Users\Public\Documents\Agilent\MicroLab\Methods\400-CM.a2m
User:admin
Date/Time:03/13/2023 1:43:02 PM
Range:4000 - 400
Apodization:Happ-Genzel

File Location:C:\Users\Public\Documents\Agilent\MicroLab\Results\\LCJ-007-146B_2023-03-13T13-43-02.a2r

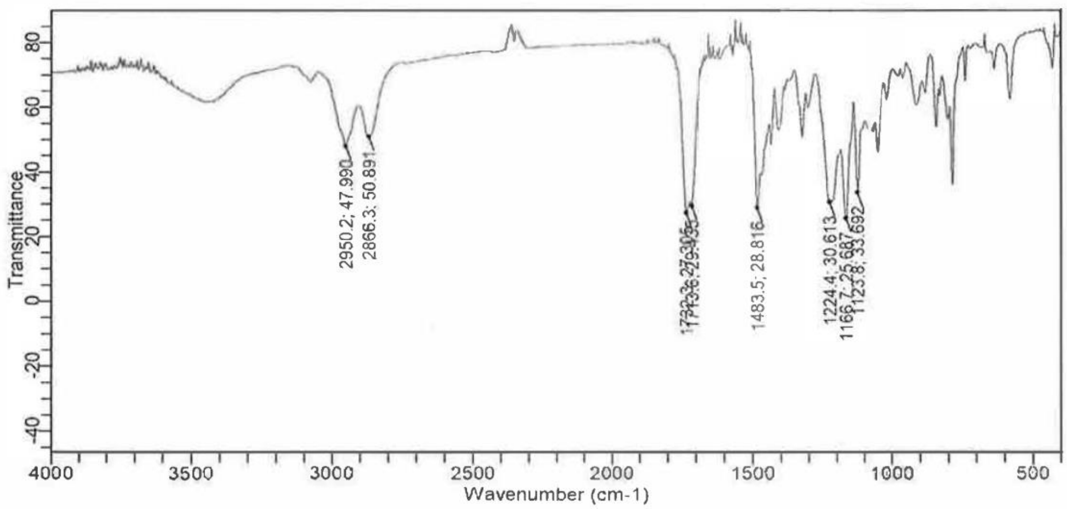

Peak Number	Wavenumber (cm^{-1})	Intensity
1	1123.79236	33.69202
2	1166.65674	25.68660
3	1224.43048	30.61295
4	1483.48046	28.81632
5	1713.64357	29.43483

6	1732.28026	27.30534
7	2866.32280	50.89122
8	2950.18790	47.99018

Sample ID:LCJ-007-146C

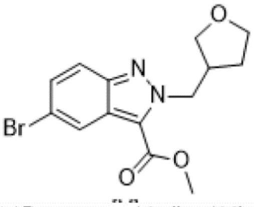

Sample Scans:128
Background Scans:32
Resolution:4
System Status:Good

Method Name:C:\Users\Public\Documents\Agilent\MicroLab\Methods\ATR 128 Scans.a2m
User:admin
Date/Time:03/13/2023 12:44:01 PM
Range:4000 - 650
Apodization:Happ-Genzel

File Location:C:\Users\Public\Documents\Agilent\MicroLab\Results\\LCJ-007-146C_2023-03-13T12-44-01.a2r

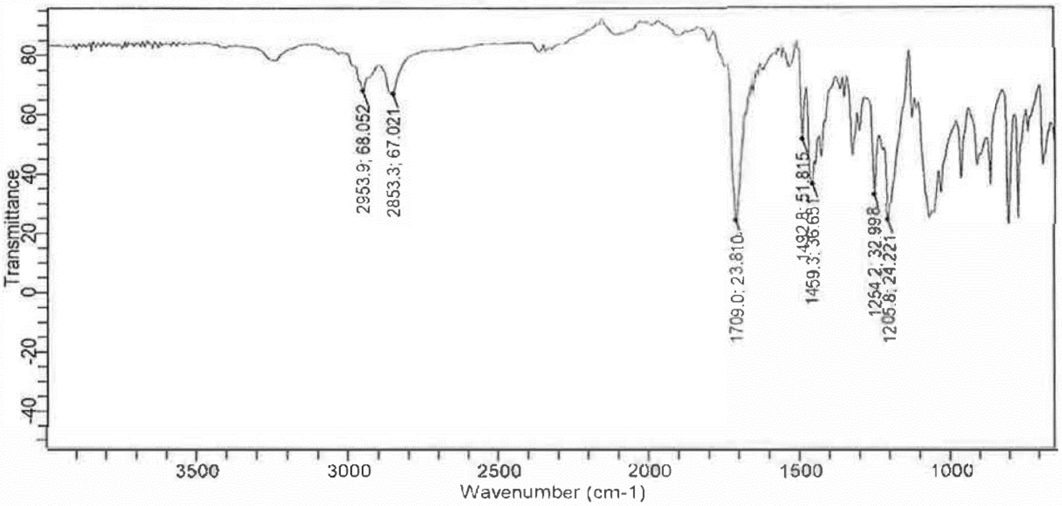

Peak Number	Wavenumber (cm^{-1})	Intensity
1	1205.79379	24.22135
2	1254.24918	32.99819
3	1459.25276	36.65103
4	1492.79880	51.81519
5	1708.98440	23.81042

6	2853.27711	67.02071
7	2953.91524	68.05167

Sample ID:LCJ-007-139B

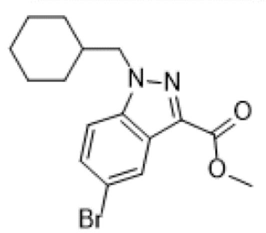

Sample Scans:32
Background Scans:32
Resolution:2
System Status:Good

Method Name:C:\Users\Public\Documents\Agilent\MicroLab\Methods\400-CM.a2m
User:admin
Date/Time:03/13/2023 1:19:20 PM
Range:4000 - 400
Apodization:Happ-Genzel

File Location:C:\Users\Public\Documents\Agilent\MicroLab\Results\\LCJ-007-139B_2023-03-13T13-19-20.a2r

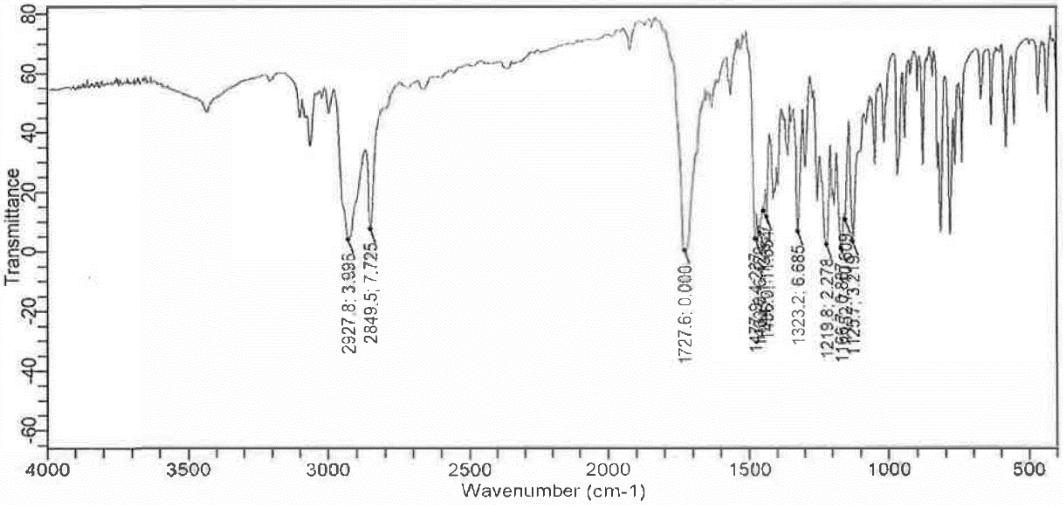

Peak Number	Wavenumber (cm^{-1})	Intensity
1	1125.65603	3.21879
2	1152.67923	10.60875
3	1166.65674	0.88655
4	1219.77131	2.27848
5	1323.20493	6.68505

6	1435.95690	11.65402
7	1447.13892	13.57684
8	1462.98010	6.41979
9	1477.88945	4.23725
10	1727.62109	0.00001
11	2849.54978	7.72533
12	2927.82387	3.99554

Sample ID:LCJ-007-139C

Sample Scans:128
Background Scans:32
Resolution:4
System Status:Good

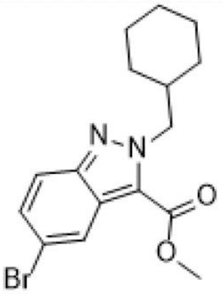

Method
Name:C:\Users\Public\Documents\Agilent\MicroLab\Methods\ATR 128 Scans.a2m
User:admin
Date/Time:03/13/2023 9:17:09 AM
Range:4000 - 650
Apodization:Happ-Genzel

File Location:C:\Users\Public\Documents\Agilent\MicroLab\Results\\LCJ-007-139C_2023-03-13T09-17-09.a2r

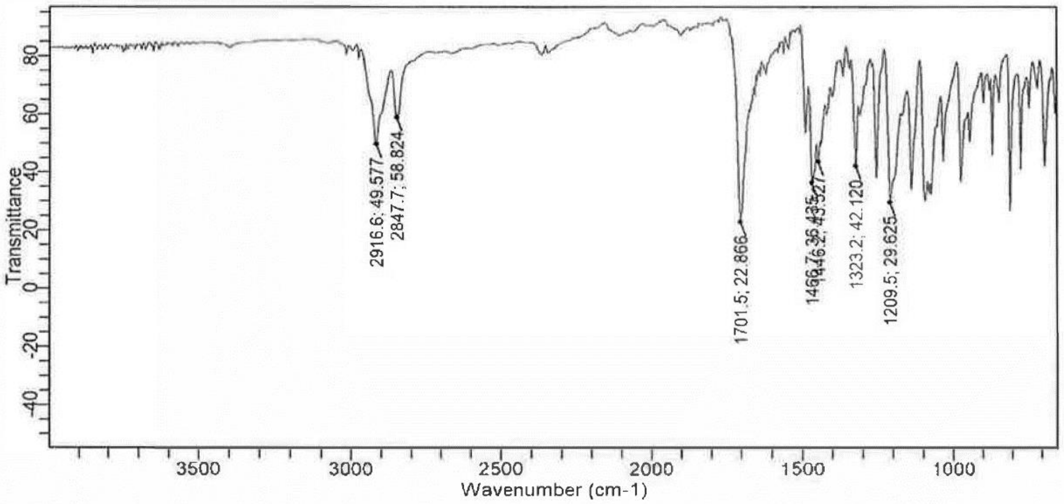

Peak Number	Wavenumber (cm^{-1})	Intensity
1	1209.52113	29.62499
2	1323.20493	42.11959
3	1446.20708	43.52681
4	1466.70744	36.43514
5	1701.52972	22.86613

6	2847.68611	58.82384
7	2916.64186	49.57708

bio cryst

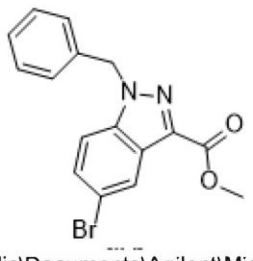

Sample ID:LCJ-009-186B

Sample Scans:128
Background Scans:32
Resolution:4
System Status:Good

Method Name:C:\Users\Public\Documents\Agilent\MicroLab\Methods\ATR 128 Scans.a2m
User:admin
Date/Time:03/13/2023 12:03:15 PM
Range:4000 - 650
Apodization:Happ-Genzel

File Location:C:\Users\Public\Documents\Agilent\MicroLab\Results\\LCJ-009-186B_2023-03-13T12-03-15.a2r

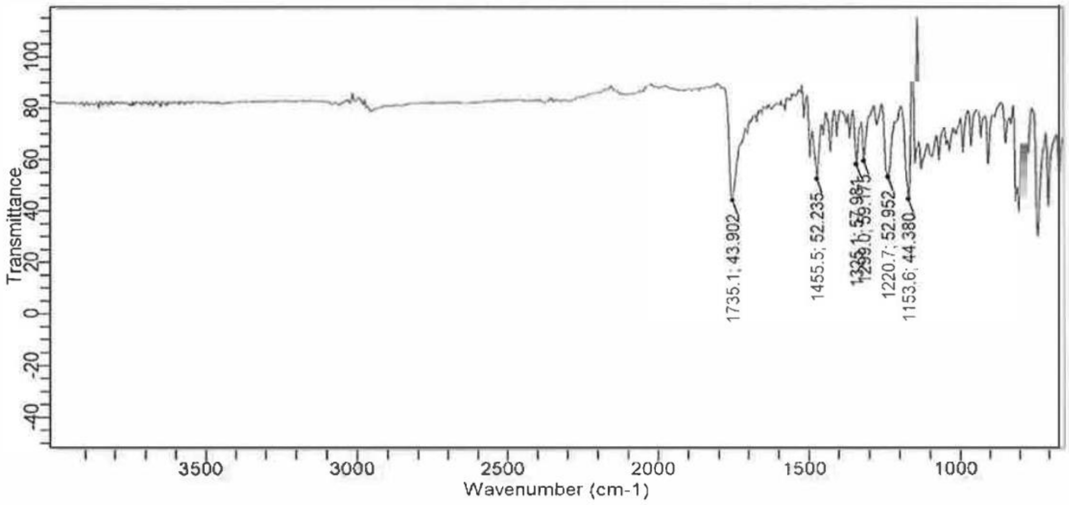

Peak Number	Wavenumber (cm^{-1})	Intensity
1	1153.61106	44.38023
2	1220.70314	52.95247
3	1298.97724	59.17542
4	1325.06860	57.98121
5	1455.52543	52.23538

6	1735.07576	43.90218

Sample ID:LCJ-009-186C

Sample Scans:128
Background Scans:32
Resolution:4
System Status:Good

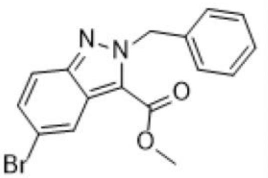

Method Name:C:\Users\Public\Documents\Agilent\MicroLab\Methods\ATR 128 Scans.a2m
User:admin
Date/Time:03/13/2023 11:56:41 AM
Range:4000 - 650
Apodization:Happ-Genzel

File Location:C:\Users\Public\Documents\Agilent\MicroLab\Results\\LCJ-009-186C_2023-03-13T11-56-41.a2r

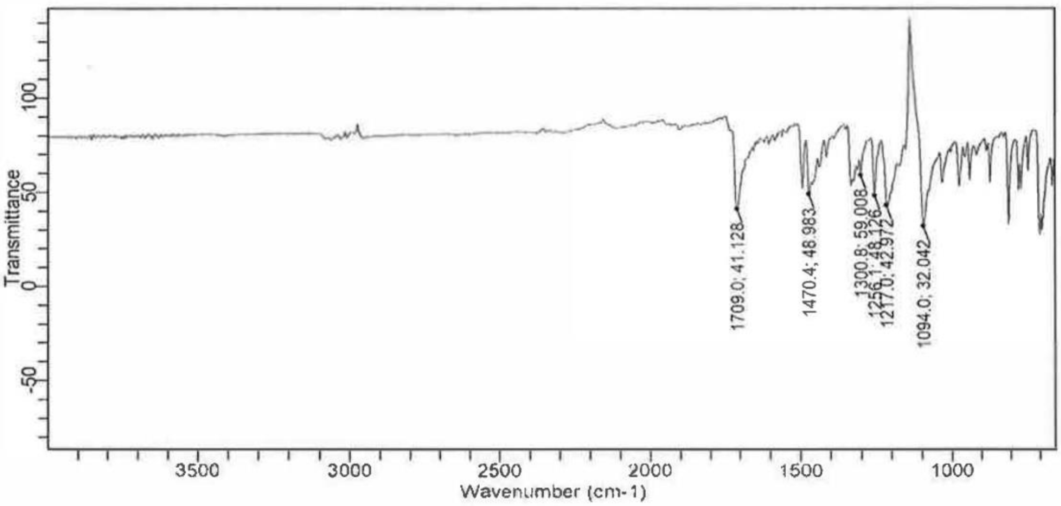

Peak Number	Wavenumber (cm^{-1})	Intensity
1	1093.97366	32.04191
2	1216.97580	42.97187
3	1256.11285	48.12629
4	1300.84091	59.00798
5	1470.43478	48.98330

6	1708.98440	41.12782

bio cryst

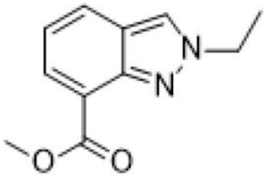

Mw 20 July 23

Sample ID:LCJ-013-121B

Sample Scans:32
Background Scans:32
Resolution:2
System Status:Good

Method Name:C:\Users\Public\Documents\Agilent\MicroLab\Methods\400-CM.a2m
User:admin
Date/Time:07/20/2023 1:20:47 PM
Range:4000 - 400
Apodization:Happ-Genzel

File Location:C:\Users\Public\Documents\Agilent\MicroLab\Results\LCJ-013-121B_2023-07-20T13-20-47.a2r

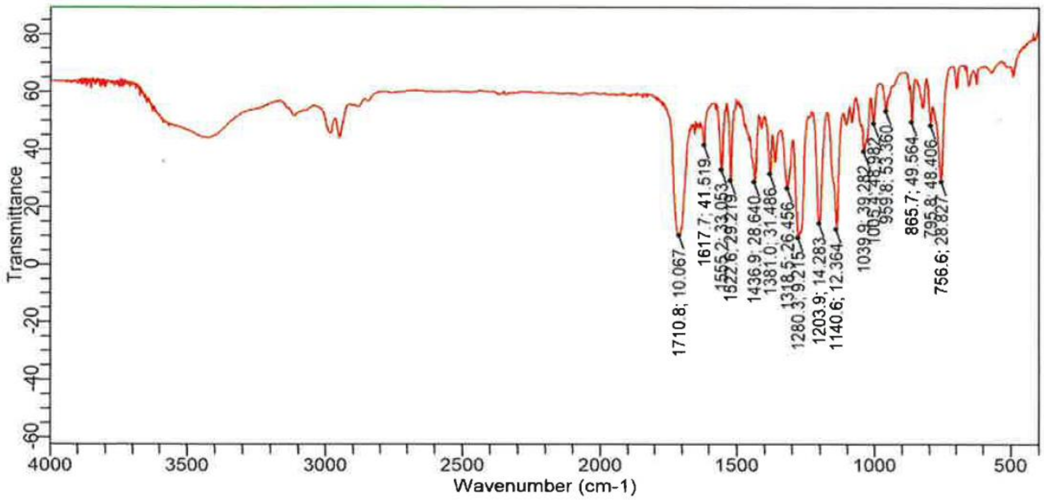

Peak Number	Wavenumber (cm⁻¹)	Intensity
1	756.64958	28.82692
2	795.78663	48.40602
3	865.67421	49.56427
4	959.78949	53.35960
5	1005.44938	48.98203

6	1039.92726	39.28233
7	1140.56538	12.36356
8	1203.93012	14.28277
9	1280.34055	9.21451
10	1318.54576	26.45588
11	1380.97867	31.48569
12	1436.88874	28.63951
13	1522.61751	29.21877
14	1555.23171	33.05342
15	1617.66462	41.51938
16	1710.84807	10.06669

biocryst

MW 27 July 23

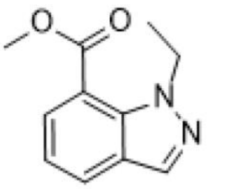

[IV]
Exact Mass: 204.09

Sample ID:LCJ-013-121C

Sample Scans:32
Background Scans:32
Resolution:2
System Status:Good

Method Name:C:\Users\Public\Documents\Agilent\MicroLab\Methods\400-CM.a2m
User:admin
Date/Time:07/27/2023 10:26:50 AM
Range:4000 - 400
Apodization:Happ-Genzel

File Location:C:\Users\Public\Documents\Agilent\MicroLab\Results\\LCJ-013-121C_2023-07-27T10-26-50.a2r

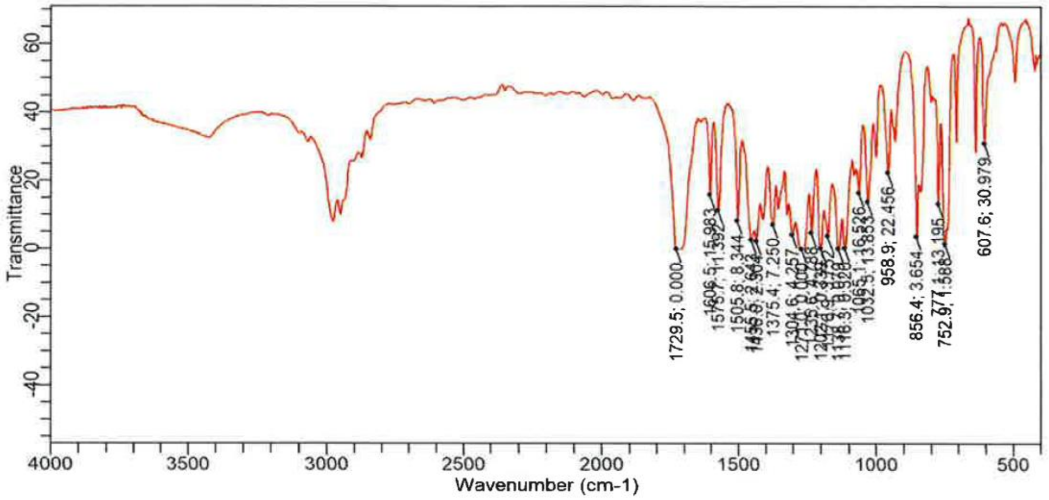

Peak Number	Wavenumber (cm-1)	Intensity
1	607.55607	30.97905
2	752.92224	1.58788
3	777.14994	13.19529
4	856.35587	3.65414
5	958.85766	22.45559

6	1032.47258	13.85300
7	1065.08679	16.52627
8	1116.33768	0.31963
9	1138.70171	0.07853
10	1176.90692	3.75183
11	1202.06645	0.33912
12	1235.61249	4.78812
13	1271.02220	0.00001
14	1304.56824	4.25745
15	1375.38766	7.24957
16	1435.95690	2.30385
17	1455.52543	2.64266
18	1505.84449	8.34396
19	1575.73207	11.39162
20	1606.48261	15.98287
21	1729.48476	0.00001

MW 21 July 23

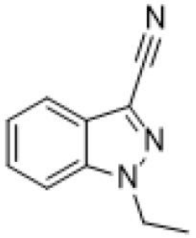

Sample ID:LCJ-013-123B

Sample Scans:32
Background Scans:32
Resolution:2
System Status:Good

Method Name:C:\Users\Public\Documents\Agilent\MicroLab\Methods\400-CM.a2m
User:admin
Date/Time:07/21/2023 7:59:50 AM
Range:4000 - 400
Apodization:Happ-Genzel

File Location:C:\Users\Public\Documents\Agilent\MicroLab\Results\LCJ-013-123B_2023-07-21T07-59-50.a2r

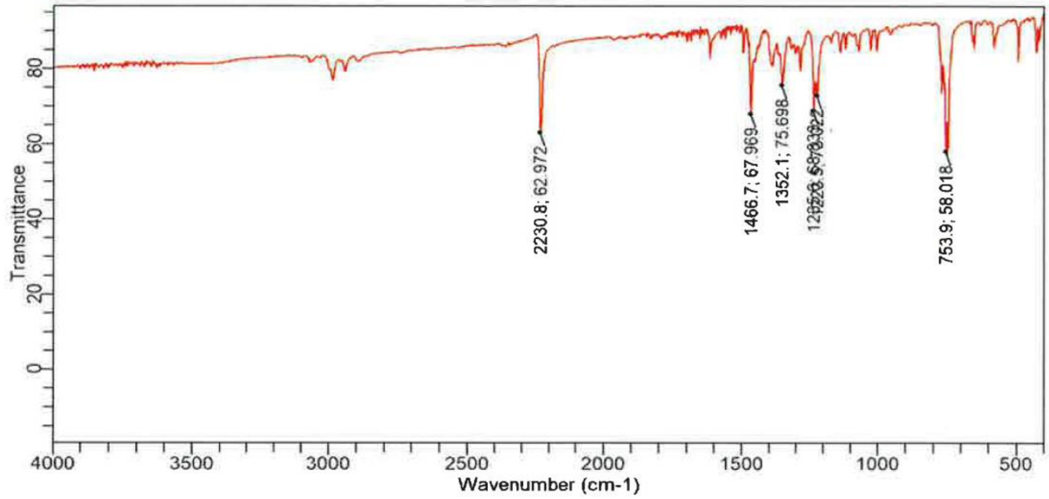

Peak Number	Wavenumber (cm-1)	Intensity
1	753.85408	58.01833
2	1223.49865	73.02160
3	1235.61249	68.83340
4	1352.09180	75.69780
5	1466.70744	67.96871

6	2230.81170	62.97228

(1) a, C. W.; a, C. N. V.; b, P. G. C.; b, S. K.-G.; b, M. A. O.; b, G. T. Elucidation of the Presence and Location of T-Boc Protecting Groups in Amines and Dipeptides Using on-Column H/D Exchange HPLC/ESI/MS. *J. Am. Soc. Mass Spectrom.* **2005**, *16* (4), 553–564. https://doi.org/10.1016/j.jasms.2005.01.008.
