# Supplementary material for: Regioselective alkylation of a versatile indazole: Electrophile scope and mechanistic insights from density functional theory calculations
Source: Beilstein J Org Chem. 2024 Aug 9;20:1940–54. doi: 10.3762/bjoc.20.170 (PMC11318628; doi:10.3762/bjoc.20.170)
Supplement: File 2 — DFT methods, relative energy comparisons, TS imaginary frequencies, and XYZ coordinates. [file Beilstein_J_Org_Chem-20-1940-s002.pdf]

BEILSTEIN JOURNAL OF ORGANIC CHEMISTRY

Supporting Information

for

Regioselective alkylation of a versatile indazole: Electrophile scope and mechanistic insights from density functional theory calculations

Pengcheng Lu, Luis Juarez, Paul A. Wiget, Weihe Zhang, Krishnan Raman
and Pravin L. Kotian

Beilstein J. Org. Chem. **2024,** *20,* 1940–1954. doi:10.3762/bjoc.20.170

DFT methods, relative energy comparisons, TS imaginary frequencies, and XYZ coordinates

License and Terms: This is a supporting information file under the terms of the Creative Commons Attribution License (https://creativecommons.org/licenses/by/4.0). Please note that the reuse, redistribution and reproduction in particular requires that the author(s) and source are credited and that individual graphics may be subject to special legal provisions.
The license is subject to the *Beilstein Journal of Organic Chemistry* terms and conditions: (https://www.beilstein-journals.org/bjoc/terms)

I. *Computational methods*

Geometry optimizations of ground-state reactants, products, and intermediates (INT) and transition-state (TS) structures were performed with Gaussian 16 rev. C.01[i] with density functional theory (DFT). The PBE0 functional[ii] was used with the def2-TZVP basis set for the cesium atom and def2SVP for all other atoms to optimize the geometries of all ground and transition states. Single-point electronic energies were evaluated at optimized stationary point geometries at the PBE0/def2-TZVP level of theory. The SMD[iii] implicit solvation mode (solvent = tetrahydrofuran (THF)) was used throughout. All DFT calculations used an "ultrafine" grid for numerical integration.

Quasi-harmonic corrections were applied to the computed vibrational entropies using the GoodVibe[iv] program. Standard state corrections were evaluated at 1 M concentration and a reaction temperature of 50 °C (for the Mitsunobu reaction) and 90 °C (for the S_N2 reaction with cesium). Reported Gibbs free energies (kcal/mol) are Boltzmann weighted over all the conformers found in each reaction step. All thermochemical data, including absolute and zero-point energies (ZPE) were generated and tabulated below.

Compound IDs are provided for Tables 1–6. A compound ID of "***n.a.***" is assigned for ancillary calculations whose data are not present in the manuscript.

Table S1: Summarized raw absolute energy values in Hartrees for the reaction pathways studied with the methyl-5-bromo-1*H*-indazole-3-carboxylate substrate for the Mitsunobu reaction calculated at 323.15 K (50 °C).

Compound ID	**Structure**	**E_SPC**	**E**	**ZPE**	**H_SPC**	**T.S**	**T.qh-S**	**G(T)_SPC**	**qh-G(T)_SPC**	**V_{imag}**
6(-N1H-Z)	indazole_deprot_neg_a	-3180.0788	-3179.0994	0.138882	-3179.9253	0.05587	0.0544	-3179.9811	-3179.9797	
6(-N1H-E)	indazole_deprot_neg_b	-3180.0784	-3179.0991	0.138831	-3179.925	0.056029	0.054482	-3179.981	-3179.9794	
15a	product_indazole_1Me_neut_a	-3219.8465	-3218.8345	0.180389	-3219.6492	0.060916	0.058909	-3219.7102	-3219.7082	
15a	product_indazole_1Me_neut_b	-3219.846	-3218.834	0.18038	-3219.6488	0.060705	0.058801	-3219.7095	-3219.7076	
15b	product_indazole_2Me_neut_a	-3219.8389	-3218.8275	0.180894	-3219.6415	0.059482	0.05795	-3219.701	-3219.6995	
15b	product_indazole_2Me_neut_b	-3219.8407	-3218.8292	0.180803	-3219.6433	0.059644	0.058085	-3219.703	-3219.7014	
6	rxt_indazole_1H_neut	-3180.5683	-3179.596	0.153056	-3180.4005	0.055911	0.05455	-3180.4564	-3180.4551	
n.a.	rxt_indazole_2H_neut	-3180.5635	-3179.591	0.153099	-3180.3956	0.056008	0.054599	-3180.4516	-3180.4502	
N1-P	INT_1PPh3OMe_b_neut	-4330.6699	-4328.721	0.46177	-4330.1702	0.11027	0.1011	-4330.2805	-4330.2713	
n.a.	INT_deprot-indazole_MeOPPh3_neut_a	-4330.6715	-4328.7132	0.460847	-4330.1719	0.11623	0.104356	-4330.2882	-4330.2763	
17	product_BaseH_neut	-567.28394	-566.64003	0.140761	-567.12947	0.052572	0.050897	-567.18204	-567.18037	

Ph_3PO	product_OPPh3_neut	-1110.8817	-1109.9326	0.280404	-1110.5808	0.069732	0.065215	-1110.6506	-1110.6461	
DMAD-H^-	rxt_Base_neg_b	-566.77668	-566.12226	0.126716	-566.63668	0.051735	0.050235	-566.68842	-566.68692	
$MeOPPh_3^+$	rxt_MeOPPh3_pos	-1150.5782	-1149.596	0.321178	-1150.2344	0.073461	0.069304	-1150.3079	-1150.3037	
N2-s-cis	TS1a_E_SN2_neut_c	-4330.6555	-4328.6989	0.46013	-4330.1575	0.113069	0.102112	-4330.2706	-4330.2597	-598.63
N2-s-cis	TS1a_Z_SN2_neut_a	-4330.6536	-4328.6958	0.460105	-4330.1556	0.114335	0.102684	-4330.2699	-4330.2583	-603.3
N2-s-cis	TS1a_Z_SN2_neut_d	-4330.6548	-4328.6973	0.46005	-4330.1569	0.112946	0.102056	-4330.2698	-4330.2589	-600.29
N1-s-trans	TS2a_E_SN2_neut_b	-4330.6517	-4328.6954	0.460065	-4330.1538	0.112124	0.101758	-4330.2659	-4330.2556	-614
N1-s-trans	TS2a_Z_SN2_neut_a	-4330.6523	-4328.6952	0.460006	-4330.1544	0.114555	0.102763	-4330.2689	-4330.2571	-616.83
N1-s-trans	TS2a_Z_SN2_neut_c	-4330.6532	-4328.6944	0.459768	-4330.1554	0.113729	0.102597	-4330.2692	-4330.258	-601.31
**n.a.**	ts_opt_TS1b_2p2_2M e-indazole_a	-4330.604	-4328.6516	0.459019	-4330.1073	0.109499	0.100353	-4330.2168	-4330.2077	-582.77
**n.a.**	ts_opt_TS1b_2p2_2M e-indazole_b	-4330.5993	-4328.6475	0.458376	-4330.1029	0.110961	0.101356	-4330.2139	-4330.2043	-558.26
N1-P-TS	ts_opt_TS1c_4p2_2M e-indazole	-4330.5936	-4328.6409	0.45794	-4330.0974	0.11577	0.10357	-4330.2132	-4330.201	-563.96
**n.a.**	ts_opt_TS2b_2p2_1M e-indazole	-4330.5989	-4328.6438	0.458526	-4330.1027	0.109046	0.100448	-4330.2117	-4330.2031	-572.95

Table S2: Summarized raw absolute energy values in Hartrees for the reaction pathways studied with the methyl-5-bromo-1*H*-indazole-3-carboxylate substrate for the S_N2 reaction with cesium calculated at 363.15 K (90 °C).

Compound ID	**Structure**	**E_SPC**	**E**	**ZPE**	**H_SPC**	**T.S**	**T.qh-S**	**G(T)_SPC**	**qh-G(T)_SPC**	**V_{imag}**
6(-N1H)	indazole_deprot_neg_a	3180.0788	3179.0994	0.138882	3179.9221	0.066024	0.064171	3179.9881	3179.9863	
6(-N1H)	indazole_deprot_neg_b	3180.0784	3179.0991	0.138831	3179.9218	0.066204	0.064253	-3179.988	-3179.986	
15a	product_indazole_1Me _neut_a	3219.8465	3218.8345	0.180389	3219.6456	0.072185	0.069652	3219.7178	3219.7153	
15a	product_indazole_1Me _neut_b	-3219.846	-3218.834	0.18038	3219.6451	0.07195	0.069544	3219.7171	3219.7147	
15b	product_indazole_2Me _neut_a	3219.8389	3218.8275	0.180894	3219.6379	0.070562	0.068623	3219.7084	3219.7065	
15b	product_indazole_2Me _neut_b	3219.8407	3218.8292	0.180803	3219.6397	0.070747	0.068769	3219.7104	3219.7085	
6	rxt_indazole_1H_neut	3180.5683	-3179.596	0.153056	3180.3973	0.066143	0.064418	3180.4634	3180.4617	
**n.a.**	rxt_indazole_2H_neut	3180.5635	-3179.591	0.153099	3180.3924	0.066244	0.064453	3180.4586	3180.4568	
**n.a.**	indazole_1Me_Cs_1_p os_a	3239.9421	3238.9339	0.181433	3239.7374	0.082669	0.077905	3239.8201	3239.8153	

**n.a.**	indazole_1Me_Cs_2_pos_b	3239.9557	3238.9498	0.181368	3239.7511	0.082401	0.077811	3239.8335	3239.8289	
**n.a.**	indazole_2Me_Cs_O_pos_b	3239.9437	3238.9356	0.181247	3239.7392	0.083492	0.078089	3239.8227	3239.8173	
6(N-H)NNCs-Z	indazole_Cs_1_neut_a	-3200.204	3199.2336	0.139726	3200.0435	0.077145	0.072887	3200.1207	3200.1164	
6(N-H)NNCs-E	indazole_Cs_1_neut_b	3200.2044	3199.2345	0.139753	-3200.044	0.077056	0.072797	-3200.121	3200.1168	
6(N-H)NOCs-E	indazole_Cs_2_neut_a	-3200.203	-3199.233	0.1396	3200.0426	0.077869	0.073161	3200.1205	3200.1158	
6(N-H)NOCs-Z	indazole_Cs_2_neut_b	-3200.206	3199.2367	0.139772	3200.0455	0.076615	0.072599	3200.1221	3200.1181	
6(N-H)OCs-E	indazole_Cs_O_neut_a	3200.1958	3199.2219	0.139582	3200.0355	0.076457	0.072477	3200.1119	-3200.108	
$CsHCO_3$	product_CO3HCs	284.50706	284.19375	0.028074	284.47051	0.045312	0.044472	284.51583	284.51498	
CsOTs	product_CsOTs	914.63938	913.88753	0.132022	914.48895	0.071956	0.066707	-914.5609	914.55565	
HCO_3	product_HCO3	264.37405	264.04542	0.026942	264.34131	0.034327	0.034328	264.37564	264.37564	
**n.a.**	product_Ots	894.51432	893.74534	0.131662	-894.3674	0.059422	0.057135	894.42682	894.42453	
Cs_2CO_3	rxt_CO3Cs2	304.11565	303.80566	0.016225	-304.0888	0.05436	0.052431	304.14316	304.14124	
Cs^+	rxt_Cs_pos	20.097396	20.097396	0	20.094521	0.020139	0.020139	-20.11466	-20.11466	
MeOTs	rxt_MeOTs	934.23552	933.44194	0.171708	934.04568	0.065818	0.063053	-934.1115	934.10873	
N1-s-cis	TS5A_E_1Me_OTs_b	4134.4228	4132.6658	0.311292	4134.0717	0.124271	0.111906	-4134.196	4134.1837	-626.63
N1-s-trans	TS5A_Z_1Me_OTs_b	4134.4205	4132.6631	0.31127	4134.0696	0.123555	0.111537	4134.1931	4134.1811	-625.71
N2-s-cis	TS5B_E_2Me_OTs_a	4134.4193	-4132.661	0.311135	4134.0685	0.124251	0.111917	4134.1927	4134.1804	-605.52
N2-s-trans	TS5B_Z_2Me_OTs_a	4134.4182	4132.6592	0.311306	4134.0671	0.124606	0.111863	4134.1917	-4134.179	-618.7

Table S3: Summarized raw absolute energy values in Hartrees for the reaction pathways studied with the methyl-1*H*-indazole-7-carboxylate substrate for the Mitsunobu reaction calculated at 323.15 K (50 °C).

Compound ID	**Structure**	**E_SPC**	**E**	**ZPE**	**H_SPC**	**T.S**	**T.qh-S**	**G(T)_SPC**	**qh-G(T)_SPC**	**V_{imag}**
18(N-H)	dep_indazole_E_7CO2Me_a	606.79948	-606.1318	0.149075	606.63759	0.050032	0.049174	606.68762	606.68676	
18(N-H)	dep_indazole_Z_7CO2Me_a	606.79796	-606.1307	0.149006	606.63612	0.050083	0.049237	-606.6862	606.68535	
18-N1	product_indazole_1Me_E_7CO2Me	646.57403	645.87444	0.191072	646.36814	0.05439	0.053126	646.42253	646.42126	
18-N1	product_indazole_1Me_Z_7CO2Me	-646.5745	645.87518	0.190845	646.36865	0.05508	0.053657	646.42373	646.42231	

18-N2	product_indazole_2Me_E_7CO2Me	646.57306	645.87253	0.190777	646.36731	0.055282	0.05386	646.42259	646.42117	
18-N2	product_indazole_2Me_Z_7CO2Me	646.57222	645.87195	0.190649	646.36652	0.05545	0.053915	646.42197	646.42044	
18	rxt_indazole_1H_E_7CO2Me_a	607.30549	606.64576	0.163307	607.12914	0.050372	0.049551	607.17951	607.17869	
18	rxt_indazole_1H_Z_7CO2Me_a	607.30674	-606.6468	0.163378	607.13036	0.050245	0.049499	-607.1806	607.17985	
**n.a.**	rxt_indazole_2H_E_7CO2Me_a	607.29454	606.63384	0.16332	607.11821	0.05067	0.049591	607.16888	-607.1678	
**n.a.**	rxt_indazole_2H_Z_7CO2Me_a	-607.2937	606.63322	0.163467	607.11727	0.050263	0.04942	607.16753	607.16669	
17	product_BaseH_neut	567.28394	566.64003	0.140761	567.12947	0.052572	0.050897	567.18204	567.18037	
Ph_3PO	product_OPPh3_neut	1110.8817	1109.9326	0.280404	1110.5808	0.069732	0.065215	1110.6506	1110.6461	
DMAD-H^-	rxt_Base_neg_b	566.77668	566.12226	0.126716	566.63668	0.051735	0.050235	566.68842	566.68692	
$MeOPPh_3$	rxt_MeOPPh3_pos	1150.5782	-1149.596	0.321178	1150.2344	0.073461	0.069304	1150.3079	1150.3037	
18-N2-OMe	TS1a_E_7CO2Me_opt_c	1757.3752	1755.7278	0.469919	1756.8691	0.108834	0.098289	1756.9779	1756.9674	-596.66
18-N2-OMe	TS1a_E_7CO2Me_opt_d	1757.3778	1755.7337	0.47044	1756.8715	0.105964	0.096573	1756.9774	1756.9681	-611.77
18-N2-OMe	TS1a_Z_7CO2Me_opt_a	1757.3758	1755.7321	0.470419	1756.8695	0.106596	0.09707	1756.9761	1756.9666	-609.87
18-N2-OMe	TS1a_Z_7CO2Me_opt_b	1757.3737	-1755.727	0.469863	1756.8677	0.110434	0.098986	1756.9781	1756.9667	-594.49
18-N1-OMe	TS2a_E_7CO2Me_opt_b	1757.3815	1755.7366	0.470327	1756.8753	0.104778	0.096142	1756.9801	1756.9715	-611.27
18-N1-OMe	TS2a_Z_7CO2Me_opt_c	1757.3804	1755.7367	0.470398	-1756.874	0.105697	0.096627	1756.9797	1756.9707	-593.02
18-N1-OMe	TS2a_Z_7CO2Me_opt_d	1757.3783	-1755.734	0.470228	-1756.872	0.107731	0.097621	1756.9797	1756.9696	-597.11

Table S4: Summarized raw absolute energy values in Hartrees for the reaction pathways studied with the methyl-1*H*-indazole-7-carboxylate substrate for the S_N2 reaction with cesium calculated at 363.15 K (90 °C).

Compound ID	**Structure**	**E_SPC**	**E**	**ZPE**	**H_SPC**	**T.S**	**T.qh-S**	**G(T)_SPC**	**qh-G(T)_SPC**	**V_{imag}**
18(N-H)	dep_indazole_E_7CO2Me_a	-606.79948	-606.1318	0.149075	606.63467	0.059182	0.058089	606.69385	-606.69276	
18(N-H)	dep_indazole_Z_7CO2Me_a	-606.79796	-606.1307	0.149006	-606.63319	0.059244	0.058166	606.69244	-606.69136	
18-N1	product_indazole_1Me_E_7CO2Me	-646.57403	645.87444	0.191072	646.36476	0.064558	0.062961	646.42932	-646.42773	
18-N1	product_indazole_1Me_Z_7CO2Me	-646.5745	645.87518	0.190845	-646.36528	0.06534	0.06354	646.43061	-646.42882	

18-N2	product_indazole_2Me _E_7CO2Me	-646.57306	645.87253	0.190777	-646.36392	0.065569	0.063783	646.42949	-646.42771	
18-N2	product_indazole_2Me _Z_7CO2Me	-646.57222	645.87195	0.190649	-646.36314	0.065763	0.063832	-646.4289	-646.42697	
18	rxt_indazole_1H_E_7C O2Me_a	-607.30549	606.64576	0.163307	-607.12615	0.05964	0.058589	607.18579	-607.18474	
18	rxt_indazole_1H_Z_7C O2Me_a	-607.30674	-606.6468	0.163378	-607.12737	0.059493	0.058531	607.18686	-607.1859	
**n.a.**	rxt_indazole_2H_E_7C O2Me_a	-607.29454	606.63384	0.16332	-607.11523	0.059965	0.058617	-607.1752	-607.17385	
**n.a.**	rxt_indazole_2H_Z_7C O2Me_a	-607.2937	606.63322	0.163467	-607.11429	0.059506	0.058432	607.17379	-607.17272	
**n.a.**	indazole_Cs_1_E_7CO 2Me	-626.92794	626.27002	0.149924	-626.75927	0.070907	0.067258	626.83018	-626.82653	
**n.a.**	indazole_Cs_1_Z_7CO 2Me	-626.93091	626.27472	0.149922	-626.76224	0.070272	0.067091	626.83251	-626.82933	
**n.a.**	indazole_Cs_2_E_7CO 2Me	-626.92795	626.27002	0.149928	-626.75927	0.070871	0.067241	626.83014	-626.82651	
**n.a.**	indazole_Cs_2_Z_7CO 2Me	-626.93091	626.27472	0.149921	-626.76224	0.070273	0.067092	626.83251	-626.82933	
**$CsHCO_3$**	product_CO3HCs	-284.50706	284.19375	0.028074	-284.47051	0.045312	0.044472	284.51583	-284.51498	
CsOTs	product_CsOTs	-914.63938	913.88753	0.132022	-914.48895	0.071956	0.066707	-914.5609	-914.55565	
**HCO_3**	product_HCO3	-264.37405	264.04542	0.026942	-264.34131	0.034327	0.034328	264.37564	-264.37564	
**n.a.**	product_OTs	-894.51432	893.74534	0.131662	-894.3674	0.059422	0.057135	894.42682	-894.42453	
**Cs_2CO_3**	rxt_CO3Cs2	-304.11565	303.80566	0.016225	-304.0888	0.05436	0.052431	304.14316	-304.14124	
**Cs^+**	rxt_Cs_pos	-20.097396	20.097396	0	-20.094521	0.020139	0.020139	-20.11466	-20.11466	
MeOTs	rxt_MeOTs	-934.23552	933.44194	0.171708	-934.04568	0.065818	0.063053	-934.1115	-934.10873	
18-N2-Cs	TS5A_Z_7CO2Me_OT s_a	-1561.1422	1559.6963	0.321473	-1560.7831	0.117054	0.106161	1560.9002	-1560.8893	-598.67
18-N2-Cs	TS5A_Z_7CO2Me_OT s_b	-1561.1436	1559.6976	0.321343	-1560.7845	0.12014	0.107563	1560.9047	-1560.8921	-608.54
18-N1-Cs	TS5B_E_7CO2Me_OT s_a	-1561.1452	1559.6996	0.321726	-1560.786	0.116434	0.105498	1560.9025	-1560.8915	-620.53
18-N1-Cs	TS5B_Z_7CO2Me_OT s_a	-1561.1329	1559.6828	0.320799	-1560.7741	0.122533	0.109004	1560.8967	-1560.8831	-601.08
18-N1-Cs	TS5B_Z_7CO2Me_OT s_b	-1561.1492	1559.7051	0.321549	-1560.7901	0.117884	0.106328	-1560.908	-1560.8964	-622.06

Table S5: Summarized raw absolute energy values in Hartrees for the reaction pathways studied with the 1*H*-indazole-3-carbonitrile substrate for the Mitsunobu reaction calculated at 323.15 K (50 °C).

Compoound ID	Structure	E_SPC	E	ZPE	H_SPC	T.S	T.qh-S	G(T)_SPC	qh-G(T)_SPC	V_{imag}
21(N-H)	dep_indazole_3CN_a	471.25823	470.74516	0.104951	471.14355	0.042642	0.042451	471.18619	-471.186	
22	product_indazole_1Me_3CN	511.02226	510.47642	0.146421	510.86393	0.047606	0.047089	510.91153	510.91102	
**n.a.**	product_indazole_2Me_3CN	511.01513	510.46874	0.146156	510.85695	0.048357	0.047328	510.90531	510.90428	
21	rxt_indazole_1H_3CN	471.74393	471.23783	0.118952	471.61507	0.042866	0.042711	471.65793	471.65778	
**n.a.**	rxt_indazole_2H_3CN	471.73584	471.22933	0.118966	471.60698	0.042868	0.042699	471.64985	471.64968	
17	product_BaseH_neut	567.28394	566.64003	0.140761	567.12947	0.052572	0.050897	567.18204	567.18037	
Ph_3PO	product_OPPh3_neut	1110.8817	1109.9326	0.280404	1110.5808	0.069732	0.065215	1110.6506	1110.6461	
$DMAD\text{-}H^-$	rxt_Base_neg_b	566.77668	566.12226	0.126716	566.63668	0.051735	0.050235	566.68842	566.68692	
$MeOPPh_3$	rxt_MeOPPh3_pos	1150.5782	-1149.596	0.321178	1150.2344	0.073461	0.069304	1150.3079	1150.3037	
N_{CN}-CH-TS	TS1a_3CN_opt_a	-1621.83	1620.3374	0.425807	1621.3711	0.102002	0.092027	1621.4731	1621.4631	-613.13
N_{CN}-CH-TS	TS1a_3CN_opt_c	1621.8317	1620.3396	0.425839	1621.3728	0.100025	0.091182	1621.4728	-1621.464	-610.28
N2-CH-TS	TS2a_3CN_opt_a	-1621.831	1620.3386	0.426096	-1621.372	0.101288	0.091705	1621.4732	1621.4637	-612.96
N2-CH-TS	TS2a_3CN_opt_c	1621.8313	1620.3386	0.42576	1621.3724	0.101209	0.091768	1621.4737	1621.4642	-606.28

Table S6: Summarized raw absolute energy values in Hartrees for the reaction pathways studied with the 1*H*-indazole-3-carbonitrile substrate for the S_N2 reaction with cesium calculated at 363.15 K (90 °C).

Compound ID	Structure	E_SPC	E	ZPE	H_SPC	T.S	T.qh-S	G(T)_SPC	qh-G(T)_SPC	V_{imag}
21(N-H)	dep_indazole_3CN_a	-471.25823	-470.74516	0.104951	-471.14129	0.050174	0.04991	-471.19146	-471.1912	
22	product_indazole_1Me_3CN	-511.02226	-510.47642	0.146421	-510.8612	0.056246	0.055563	-510.91745	-510.91677	
**n.a.**	product_indazole_2Me_3CN	-511.01513	-510.46874	0.146156	-510.85422	0.057098	0.055813	-510.91132	-510.91003	
21	rxt_indazole_1H_3CN	-471.74393	-471.23783	0.118952	-471.61274	0.050503	0.050285	-471.66324	-471.66302	
**n.a.**	rxt_indazole_2H_3CN	-471.73584	-471.22933	0.118966	-471.60466	0.050499	0.050263	-471.65516	-471.65492	
21(N-H)-Cs	indazole_Cs_1_3CN	-491.38241	-490.87781	0.105662	-491.26172	0.062129	0.059555	-491.32385	-491.32128	
21(N-H)-Cs	indazole_Cs_2_3CN	-491.3824	-490.87781	0.105663	-491.26172	0.062119	0.05955	-491.32384	-491.32127	
$CsHCO_3$	product_CO3HCs	-284.50706	-284.19375	0.028074	-284.47051	0.045312	0.044472	-284.51583	-284.51498	
CsOTs	product_CsOTs	-914.63938	-913.88753	0.132022	-914.48895	0.071956	0.066707	-914.5609	-914.55565	
HCO_3	product_HCO3	-264.37405	-264.04542	0.026942	-264.34131	0.034327	0.034328	-264.37564	-264.37564	
**n.a.**	product_OTs	-894.51432	-893.74534	0.131662	-894.3674	0.059422	0.057135	-894.42682	-894.42453	
Cs_2CO_3	rxt_CO3Cs2	-304.11565	-303.80566	0.016225	-304.0888	0.05436	0.052431	-304.14316	-304.14124	
Cs^+	rxt_Cs_pos	-20.097396	-20.097396	0	-20.094521	0.020139	0.020139	-20.11466	-20.11466	
MeOTs	rxt_MeOTs	-934.23552	-933.44194	0.171708	-934.04568	0.065818	0.063053	-934.1115	-934.10873	
N2-CH-TS	TS5A_3CN_OTs_a	-1425.5966	-1424.3038	0.277003	-1425.2855	0.111676	0.100062	-1425.3972	-1425.3856	-628.49
N2-CH-TS	TS5A_3CN_OTs_b	-1425.5899	-1424.2939	0.277071	-1425.2789	0.110721	0.099721	-1425.3896	-1425.3786	-611.78
N_{CN}-Cs-TS	TS5B_3CN_OTs_a	-1425.5945	-1424.3005	0.277163	-1425.2834	0.109978	0.099101	-1425.3934	-1425.3825	-627.14
N_{CN}-Cs-TS	TS5B_3CN_OTs_b	-1425.5956	-1424.3022	0.277081	-1425.2846	0.109971	0.099432	-1425.3946	-1425.384	-612.06

II. Reaction Pathways

Table S7: Summarized reaction pathways studied for the methyl-5-bromo-1*H*-indazole-3-carboxylate substrate. Gibbs free-energy values are reported in kcal/mol.

**isomerization (kcal/mol)**	**DE_SPC**	**DE**	**DZPE**	**DH_SPC**	**T.DS**	**T.qh-DS**	**DG(T)_SPC**	**qh-DG(T)_SPC**
2Hindazole	0	0	0	0	0	0	0	0
1Hindazole	-3	-3.1	0	-3.1	-0.1	0	-3	-3.1
**TS2_1Me-formation (kcal/mol)**	**DE_SPC**	**DE**	**DZPE**	**DH_SPC**	**T.DS**	**T.qh-DS**	**DG(T)_SPC**	**qh-DG(T)_SPC**
1Hindazole+Base+MeOPPh3	0	0	0	0	0	0	0	0
dep_indazole+BaseH+MeOPPh3	-11	-13.2	-0.1	-10.9	1	0.8	-11.9	-11.7
TS2a_1Me+BaseH	-8.5	-12.8	-0.2	-8.1	-8.7	-12.4	0.6	4.3
1Me_indazole+OPPh3+BaseH	-55.6	-58.1	0.4	-55.1	1.7	1	-56.8	-56
**TS1_2Me-formation (kcal/mol)**	**DE_SPC**	**DE**	**DZPE**	**DH_SPC**	**T.DS**	**T.qh-DS**	**DG(T)_SPC**	**qh-DG(T)_SPC**
1Hindazole+Base+MeOPPh3	0	0	0	0	0	0	0	0
dep_indazole+BaseH+MeOPPh3	-11	-13.2	-0.1	-10.9	1	0.8	-11.9	-11.7
TS1a_2Me+BaseH	-9.9	-14.9	-0.1	-9.3	-9	-12.6	-0.3	3.2
2Me_indazole+OPPh3+BaseH	-52	-54.8	0.6	-51.3	0.8	0.3	-52.1	-51.6
**TS5A_1Me-formation (kcal/mol)**	**DE_SPC**	**DE**	**DZPE**	**DH_SPC**	**T.DS**	**T.qh-DS**	**DG(T)_SPC**	**qh-DG(T)_SPC**
1Hindazole+Cs2CO3+MeOTs	0	0	0	0	0	0	0	0
dep_indazole+Cs+CsHCO3+MeOTs	0.6	7.1	-1.5	-0.6	7.4	8	-8	-8.6
indazole_Cs+MeOTs+CsHCO3	-17.7	-17.3	-0.9	-18.3	1.8	1	-20.1	-19.3
TS5A_1Me+CsHCO3	-6.3	-9.9	-1	-6.4	-10.3	-14.6	3.9	8.1
1Me_indazole+CsOTs+CsHCO3	-45.9	-45.2	-0.3	-45.9	2.4	1	-48.3	-46.9
**TS5B_2Me-formation (kcal/mol)**	**DE_SPC**	**DE**	**DZPE**	**DH_SPC**	**T.DS**	**T.qh-DS**	**DG(T)_SPC**	**qh-DG(T)_SPC**
1Hindazole+Cs2CO3+MeOTs	0	0	0	0	0	0	0	0

dep_indazole+Cs+CsHCO3+MeOTs	0.6	7.1	-1.5	-0.6	7.4	8	-8	-8.6
indazole_Cs+MeOTs+CsHCO3	-17.7	-17.3	-0.9	-18.3	1.8	1	-20.1	-19.3
TS5B_2Me+CsHCO3	-4.1	-6.8	-1.1	-4.3	-10.1	-14.4	5.7	10
2Me_indazole+CsOTs+CsHCO3	-42.3	-41.8	0	-42.1	1.4	0.3	-43.5	-42.4

Table S8: Summarized reaction pathways studied for the methyl-1*H*-indazole-7-carboxylate substrate. Gibbs free-energy values are reported in kcal/mol.

**isomerization (kcal/mol)**	**DE_SPC**	**DE**	**DZPE**	**DH_SPC**	**T.DS**	**T.qh-DS**	**DG(T)_SPC**	**qh-DG(T)_SPC**
2Hindazole	0	0	0	0	0	0	0	0
1Hindazole	-7.6	-8.1	0	-7.6	-0.2	0	-7.4	-7.6
**TS2_1Me-formation (kcal/mol)**	**DE_SPC**	**DE**	**DZPE**	**DH_SPC**	**T.DS**	**T.qh-DS**	**DG(T)_SPC**	**qh-DG(T)_SPC**
1Hindazole+Base+MeOPPh3	0	0	0	0	0	0	0	0
dep_indazole+MeOPPh3+BaseH	0	-1.8	-0.2	0	0.3	0.2	-0.4	-0.2
TS2a_1Me+BaseH	-2.2	-7.3	-0.1	-1.8	-10.8	-13.4	9	11.6
1Me_indazole+OPPh3+BaseH	-49.4	-52	0.5	-48.7	1.1	0.4	-49.8	-49.1
**TS1_2Me-formation (kcal/mol)**	**DE_SPC**	**DE**	**DZPE**	**DH_SPC**	**T.DS**	**T.qh-DS**	**DG(T)_SPC**	**qh-DG(T)_SPC**
1Hindazole+Base+MeOPPh3	0	0	0	0	0	0	0	0
dep_indazole+MeOPPh3+BaseH	0	-1.8	-0.2	0	0.3	0.2	-0.4	-0.2
TS1a_2Me+BaseH	0.6	-4	-0.2	1	-9.3	-12.6	10.3	13.6
2Me_indazole+OPPh3+BaseH	-48.4	-50.3	0.4	-47.8	1.4	0.6	-49.2	-48.4
**TS5A_1Me-formation**	**DE_SPC**	**DE**	**DZPE**	**DH_SPC**	**T.DS**	**T.qh-DS**	**DG(T)_SPC**	**qh-DG(T)_SPC**
1Hindazole+Cs2CO3+MeOTs	0	0	0	0	0	0	0	0
dep_indazole+Cs+CsHCO3+MeOTs	11.6	18.5	-1.5	10.3	6.7	7.3	3.6	3
indazole_Cs+MeOTs+CsHCO3	-9.8	-10	-1	-10.5	1.4	0.7	-11.8	-11.1

TS5A_1Me+CsHCO3	4.4	1.8	-1.2	4.1	-9.3	-14.1	13.5	18.2
1Me_indazole+CsOTs+CsHCO3	-39.7	-39	-0.2	-39.6	1.7	0.3	-41.3	-39.9
**TS5B_2Me-formation**	**DE_SPC**	**DE**	**DZPE**	**DH_SPC**	**T.DS**	**T.qh-DS**	**DG(T)_SPC**	**qh-DG(T)_SPC**
1Hindazole+Cs2CO3+MeOTs	0	0	0	0	0	0	0	0
dep_indazole+Cs+CsHCO3+MeOTs	11.6	18.5	-1.5	10.3	6.7	7.3	3.6	3
indazole_Cs+MeOTs+CsHCO3	-9.8	-10	-1	-10.5	1.4	0.7	-11.8	-11.1
TS5B_2Me+CsHCO3	0.8	-2.9	-1	0.6	-10.7	-15	11.4	15.6
2Me_indazole+CsOTs+CsHCO3	-38.7	-37.3	-0.3	-38.6	2.1	0.6	-40.7	-39.3

Table S9: Summarized reaction pathways studied for the 1*H*-indazole-3-carbonitrile substrate. Gibbs free-energy values are reported in kcal/mol.

**isomerization (kcal/mol)**	**DE_SPC**	**DE**	**DZPE**	**DH_SPC**	**T.DS**	**T.qh-DS**	**DG(T)_SPC**	**qh-DG(T)_SPC**
2Hindazole	0	0	0	0	0	0	0	0
1Hindazole	-5.1	-5.3	0	-5.1	0	0	-5.1	-5.1
**TS2_1Me-formation (kcal/mol)**	**DE_SPC**	**DE**	**DZPE**	**DH_SPC**	**T.DS**	**T.qh-DS**	**DG(T)_SPC**	**qh-DG(T)_SPC**
1Hindazole+Base+MeOPPh3	0	0	0	0	0	0	0	0
dep_indazole+BaseH+MeOPPh3	-13.5	-15.8	0	-13.3	0.4	0.3	-13.7	-13.6
TS2a_1Me+BaseH	-10.2	-14.1	-0.1	-9.8	-8.5	-11.9	-1.2	2.1
1Me_indazole+OPPh3+BaseH	-55.9	-58.3	0.5	-55.3	1.2	0.6	-56.4	-55.8
**TS1_2Me-formation (kcal/mol)**	**DE_SPC**	**DE**	**DZPE**	**DH_SPC**	**T.DS**	**T.qh-DS**	**DG(T)_SPC**	**qh-DG(T)_SPC**
**1Hindazole+Base+MeOPPh3**	0	0	0	0	0	0	0	0
dep_indazole+BaseH+MeOPPh3	-13.5	-15.8	0	-13.3	0.4	0.3	-13.7	-13.6
TS1a_2Me+BaseH	-10.2	-14.3	-0.2	-9.8	-8.9	-12.1	-0.8	2.3
2Me_indazole+OPPh3+BaseH	-51.4	-53.5	0.3	-50.9	1.6	0.7	-52.5	-51.6
**TS5A_1Me-formation**	**DE_SPC**	**DE**	**DZPE**	**DH_SPC**	**T.DS**	**T.qh-DS**	**DG(T)_SPC**	**qh-DG(T)_SPC**

1Hindazole+Cs2CO3+MeOTs	0	0	0	0	0	0	0	0
dep_indazole+Cs+CsHCO3+MeOTs	-2	4.5	-1.4	-3	6.8	7.4	-9.8	-10.4
indazole_Cs+MeOTs+CsHCO3	-18.8	-17.6	-0.9	-19.3	2.1	1.3	-21.4	-20.6
TS5A_1Me+CsHCO3	-5.4	-7.6	-1.1	-5.5	-8.6	-13.3	3.1	7.8
1Me_indazole+CsOTs+CsHCO3	-46.2	-45.3	-0.2	-46.1	1.8	0.6	-47.9	-46.7
**TS5B_2Me-formation**	**DE_SPC**	**DE**	**DZPE**	**DH_SPC**	**T.DS**	**T.qh-DS**	**DG(T)_SPC**	**qh-DG(T)_SPC**
1Hindazole+Cs2CO3+MeOTs	0	0	0	0	0	0	0	0
dep_indazole+Cs+CsHCO3+MeOTs	-2	4.5	-1.4	-3	6.8	7.4	-9.8	-10.4
indazole_Cs+MeOTs+CsHCO3	-18.8	-17.6	-0.9	-19.3	2.1	1.3	-21.4	-20.6
TS5B_2Me+CsHCO3	-4.6	-6.4	-1.1	-4.8	-9.3	-13.4	4.5	8.6
2Me_indazole+CsOTs+CsHCO3	-41.7	-40.5	-0.4	-41.7	2.3	0.8	-44	-42.5

III. *XYZ Coordinate*

15
dep_indazole_3CN_a
Eopt -470.745160
C 1.972741 -1.608028 0.000580
C 2.762343 -0.442158 0.003781
C 2.208674 0.839206 0.000254
C 0.783169 0.950978 -0.002820
C -0.011581 -0.254118 -0.002867
C -1.332266 0.253815 -0.000597
N -1.296489 1.585927 0.001340
N -0.025027 2.029824 -0.000329
C 0.586909 -1.522544 -0.003004
H 3.849361 -0.535609 0.006162
H -0.024272 -2.430937 -0.005074
H 2.463573 -2.585004 0.000436
H 2.830897 1.709688 -0.000138
C -2.665916 -0.516234 0.000899
N -3.670484 -1.096271 0.002026
20
dep_indazole_E_7CO2Me_a
Eopt -606.131804
C 1.467742 2.264879 -0.019808
C 0.115369 1.872822 -0.009066
C -0.277648 0.533455 0.000329
C 0.747347 -0.463513 0.002873
C 2.128757 -0.044549 -0.005038
C 2.819977 -1.279176 0.005945
N 1.941171 -2.280838 0.019322
N 0.679540 -1.809725 0.017359
C 2.479021 1.313479 -0.018132
H -0.660988 2.639382 -0.011041
H 3.529621 1.621538 -0.026091
H 1.714558 3.329951 -0.030029
C -1.714144 0.182691 0.001178
O -2.219140 1.277974 0.075823
O -2.469822 -0.935207 -0.057963
C -3.866780 -0.736770 -0.013332
H -4.212561 -0.120087 -0.857661
H -4.324955 -1.732538 -0.071905
H -4.173575 -0.242723 0.922033
H 3.896781 -1.463628 0.005351
20
dep_indazole_Z_7CO2Me_a
Eopt -606.130699
C -1.130971 2.363120 0.000002
C 0.157864 1.779504 0.000121
C 0.312898 0.405776 0.000261
C -0.856811 -0.367148 0.000172
C -2.145535 0.213977 -0.000022
C -3.029063 -0.912818 -0.000013
N -2.340698 -2.044944 -0.000113
N -1.060594 -1.715326 -0.000073
C -2.291874 1.611874 -0.000045
H 1.038782 2.424205 0.000013
H -3.281640 2.068133 0.000008
H -1.210692 3.430146 -0.000054
C 1.711994 -0.237752 -0.000046
O 1.833184 -1.490303 0.000087
O 2.877440 0.590882 -0.000483
C 4.047580 -0.231111 0.000155
H 4.049685 -0.849274 -0.873213
H 4.919315 0.389355 0.000064
H 4.049297 -0.848472 0.874090
H -4.097180 -0.849366 0.000064
25
indazole_1Me_Cs_1_pos_a
Eopt -3238.933905
Br -4.249350 -2.191993 0.002467
C -2.533899 -1.395216 -0.000047
C -2.428642 -0.022634 0.001159
C -1.121743 0.512117 -0.002586
C -0.578580 1.811107 -0.005253
C -1.338277 3.067415 0.001194
O -0.573897 4.155902 0.008157
C -1.258177 5.405007 0.013786
O -2.546559 3.089027 0.003322
N 0.772407 1.651755 -0.013854
N 1.153725 0.387002 -0.014996
C 0.018734 -0.339517 -0.008525
C -0.126719 -1.746022 -0.007540
C -1.402024 -2.259033 -0.003766
H -3.300047 0.631571 0.004564
H -1.889761 5.497962 0.909807
H -0.480741 6.177409 0.019437
H -1.887700 5.507940 -0.881857
H 0.743486 -2.405711 -0.010916
H -1.558296 -3.339511 -0.003457
Cs 3.932326 -1.139169 0.004208
C 1.793220 2.680353 -0.019065
H 1.667980 3.335025 -0.891000
H 1.728366 3.284899 0.895348

H 2.763370 2.172281 -0.065655
25
indazole_1Me_Cs_2_pos_b
Eopt -3238.949788
Br 5.239315 0.387147 0.022466
C 3.511394 -0.383928 0.002010
C 2.404689 0.444068 -0.003641
C 1.142338 -0.176126 -0.016003
C -0.220191 0.254310 -0.021737
C -0.790225 1.609364 -0.021951
O 0.152111 2.539517 -0.020131
C -0.277952 3.897229 -0.021090
O -1.979809 1.853884 -0.023417
N -1.035580 -0.794774 -0.033485
N -0.289107 -1.884679 -0.042587
C 1.045367 -1.586538 -0.026184
C 2.176463 -2.416509 -0.018817
C 3.410509 -1.795692 -0.005334
H 2.498221 1.529476 0.002387
H -0.871237 4.117999 0.878102
H 0.634546 4.504059 -0.026378
H -0.879063 4.114040 -0.915978
H 2.094767 -3.504497 -0.020769
H 4.320302 -2.398703 0.000865
Cs -4.241498 -0.138094
0.018760
C -0.882946 -3.197787 -0.007037
H -0.891219 -3.596108 1.019263
H -1.910378 -3.125707 -0.382105
H -0.311848 -3.876920 -0.653920
25
indazole_2Me_Cs_O_pos_b
Eopt -3238.935598
Br -5.412401 -1.021753 -0.052752
C -3.939175 0.165547 -0.022994
C -2.662908 -0.351965 0.011471
C -1.600253 0.577666 0.031954
C -0.191814 0.500227 0.064979
C 0.698258 -0.653105 0.092584
O 0.016577 -1.789496 0.097453
C 0.767379 -2.998025 0.119821
O 1.915560 -0.602363 0.107964
N 0.249799 1.792379 0.066217
N -0.714050 2.682789 0.038016
C -1.862827 1.979307 0.016599
C -3.185393 2.474856 -0.018168
C -4.212769 1.561744 -0.037596
H -2.477097 -1.425111 0.022487
H 1.390719 -3.054078 1.024267
H 0.031733 -3.810244 0.123406
H 1.406484 -3.076991 -0.771987
H -3.378154 3.549191 -0.029542
H -5.249510 1.901916 -0.064670
Cs 4.855536 -0.195509 -
0.059317
C 1.619926 2.258021 0.095419
H 2.122396 1.899753 1.003251
H 2.161022 1.900573 -0.790515
H 1.582059 3.352368 0.094678
16
indazole_Cs_1_3CN
Eopt -490.877805
C -4.320771 -0.951433 0.002645
C -3.707581 0.287375 0.002644
C -2.299709 0.303291 0.000283
C -1.280846 1.294226 -0.001097
N -0.079671 0.682532 -0.003276
N -0.222773 -0.632886 -0.003578
C -1.556604 -0.912089 -0.001726
C -2.214648 -2.157448 -0.001863
C -3.596951 -2.169348 0.000167
H -4.278536 1.216510 0.004656
H -1.648583 -3.092507 -0.003439
H -4.140680 -3.116164 0.000205
Cs 2.758416 -0.241108 0.000732
H -5.389984 -0.992405 0.004604
C -1.479193 2.821399 -0.000570
N -1.628596 3.971738 -0.000172
21
indazole_Cs_1_E_7CO2Me
Eopt -626.270018
C -4.252146 0.393286 -0.008720
C -4.046718 -0.976804 0.006941
C -2.727760 -1.457933 0.012136
C -2.121401 -2.735366 0.025739
N -0.805345 -2.566649 0.022685
N -0.455905 -1.274353 0.008729
C -1.602706 -0.562148 0.002153
C -1.836446 0.847620 -0.013878
C -3.160898 1.283079 -0.019587
H -4.893938 -1.668484 0.015206
H -3.352929 2.356902 -0.031362

Cs 2.597132 -0.473996 -
0.013208
H -5.267591 0.796511 -0.012715
C -0.733433 1.826863 -0.021670
O 0.452390 1.557581 0.012131
O -1.159087 3.092326 -0.070440
H -2.574990 -3.726834 0.037225
C 1.124006 2.816231 0.110281
H 0.985036 3.366349 -0.796889
H 0.720566 3.372785 0.930271
H 2.168952 2.651344 0.270902
21
indazole_Cs_1_neut_a
Eopt -3199.233608
Br -5.083898 -0.465321 0.002095
C -3.193285 -0.663504 -0.000868
C -2.398145 0.467158 0.000268
C -1.004348 0.267936 -0.001271
C 0.153865 1.091695 -0.001437
C 0.189014 2.548493 -0.000133
O 1.431233 3.056346 -0.004095
C 1.516583 4.469010 -0.001374
O -0.801612 3.251544 0.003843
N 1.247566 0.303780 -0.003309
N 0.905306 -0.974367 -0.004524
C -0.455516 -1.046650 -0.003618
C -1.295985 -2.176938 -0.004922
C -2.663899 -1.977651 -0.003697
H -2.820548 1.472572 0.002537
H 1.041585 4.897385 0.894764
H 2.585932 4.715431 -0.005650
H 1.033280 4.901618 -0.890986
H -0.879319 -3.187460 -0.006762
H -3.345812 -2.830350 -0.004571
Cs 3.911356 -1.042350 0.001754
21
indazole_Cs_1_neut_b
Eopt -3199.234453
Br 4.928793 -0.784969 0.033380
C 3.029794 -0.875184 -0.005904
C 2.302288 0.299946 -0.010594
C 0.898119 0.187632 -0.036082
C -0.214939 1.073828 -0.038671
C -0.269262 2.532016 -0.004374
O 0.965254 3.068246 -0.043524
C 1.031092 4.481600 -0.006968
O -1.272222 3.212208 0.055639
N -1.348257 0.344875 -0.061748
N -1.079024 -0.948836 -0.072618
C 0.275718 -1.095499 -0.054842
C 1.048431 -2.273064 -0.050159
C 2.425599 -2.156115 -0.024709
H 2.789104 1.275433 0.004486
H 0.584935 4.879321 0.917318
H 2.096836 4.742173 -0.045928
H 0.507125 4.929420 -0.869341
H 0.571722 -3.256676 -0.065268
H 3.056116 -3.047448 -0.018246
Cs -4.105962 -0.747549
0.020839
21
indazole_Cs_1_Z_7CO2Me
Eopt -626.274719
C -4.186117 -0.039458 -0.001128
C -3.865469 -1.387336 -0.003152
C -2.510525 -1.755148 -0.001581
C -1.798226 -2.976752 -0.003608
N -0.501172 -2.697242 -0.001702
N -0.262360 -1.379940 0.002149
C -1.465324 -0.767314 0.002609
C -1.817541 0.617704 0.004728
C -3.174093 0.939556 0.002029
H -4.651109 -2.148280 -0.005787
H -3.456318 1.993337 0.004051
Cs 2.711992 -0.323868 -0.002876
H -5.232045 0.276405 -0.001898
C -0.801369 1.686794 0.012168
O 0.402960 1.518402 0.044865
O -1.332565 2.912234 -0.020754
C -0.425350 4.000595 -0.011745
H 0.240404 3.969482 -0.887595
H -1.037283 4.910233 -0.043989
H 0.188736 3.997997 0.901646
H -2.166275 -4.003114 -0.006168
16
indazole_Cs_2_3CN
Eopt -490.877805
C -4.532165 0.834708 -0.012736
C -3.256139 1.366932 -0.007905
C -2.181378 0.457026 0.001591
C -0.762126 0.522620 0.009361
N -0.267386 -0.738807 0.016800

N -1.237487 -1.627086 0.014639
C -2.421566 -0.948821 0.005656
C -3.735590 -1.454926 0.000608
C -4.787487 -0.558039 -0.008609
H -3.086275 2.444064 -0.011285
H -3.919421 -2.532269 0.004147
H -5.819202 -0.915176 -0.012544
Cs 2.748828 -0.256095 -
0.004777
H -5.365173 1.506233 -0.019924
C 0.195858 1.728383 0.011155
N 0.917455 2.636620 0.012507
21
indazole_Cs_2_E_7CO2Me
Eopt -626.270019
C 3.957949 -1.639616 0.003179
C 2.969491 -2.605843 0.002425
C 1.635613 -2.155147 0.000064
C 0.346315 -2.753261 -0.001914
N -0.584893 -1.778662 -0.003710
N -0.014716 -0.584635 -0.003207
C 1.336382 -0.762374 -0.001194
C 2.369338 0.195198 -0.000574
C 3.677757 -0.250829 0.001453
H 3.200950 -3.671541 0.003863
H 4.504076 0.462821 0.002067
Cs -2.957672 0.031799 0.000886
C 2.052497 1.702252 -0.001981
O 3.165788 2.172167 -0.000933
O 0.957658 2.493169 -0.006497
C 1.202734 3.883381 0.003731
H 1.778212 4.196821 -0.881332
H 0.220317 4.372999 -0.002715
H 1.761636 4.187072 0.902905
H 4.980522 -1.954641 0.005155
H 0.147492 -3.804627 -0.001970
21
indazole_Cs_2_neut_a
Eopt -3199.232980
Br 5.267400 0.326420 -0.011846
C 3.508370 -0.394636 0.000002
C 2.428374 0.468276 0.001612
C 1.146672 -0.113875 0.009846
C -0.195611 0.347304 0.013965
C -0.655796 1.723645 0.010217
O -2.007324 1.837231 0.014097
C -2.510362 3.163077 0.009647
O 0.064594 2.699102 0.004000
N -1.023285 -0.723436 0.021427
N -0.339508 -1.850297 0.022731
C 0.986526 -1.530353 0.015973
C 2.111205 -2.378553 0.014520
C 3.368742 -1.804383 0.006588
H 2.554885 1.551586 -0.003442
H -2.179963 3.711972 -0.884972
H -3.605318 3.082499 0.008137
H -2.182927 3.717076 0.902266
H 1.993476 -3.465162 0.019436
H 4.262414 -2.431655 0.005041
Cs -4.022707 -0.595429 -
0.010545
21
indazole_Cs_2_neut_b
Eopt -3199.236661
Br -5.149465 0.232877 -0.007854
C -3.400300 -0.512144 -0.000794
C -2.309751 0.337695 0.001541
C -1.033086 -0.256633 0.006972
C 0.318665 0.180851 0.011205
C 0.889618 1.514307 0.012229
O -0.046009 2.471194 0.002367
C 0.414060 3.811421 0.000116
O 2.083264 1.776131 0.020389
N 1.128516 -0.905493 0.015554
N 0.426953 -2.018125 0.014590
C -0.894083 -1.676064 0.009618
C -2.028173 -2.510727 0.007200
C -3.279350 -1.922928 0.001972
H -2.429915 1.421501 -0.000715
H 1.029924 4.019178 -0.887982
H -0.482146 4.443618 -0.015266
H 1.006686 4.030214 0.901319
H -1.921425 -3.598419 0.009651
H -4.180396 -2.539459 0.000160
Cs 4.144666 -0.422460 -
0.007740
21
indazole_Cs_2_Z_7CO2Me
Eopt -626.274719
C -3.640469 -2.064319 -0.003331
C -2.548792 -2.912185 -0.000225
C -1.274647 -2.313091 0.001867

C 0.074173 -2.761095 0.005615
N 0.888821 -1.687156 0.005928
N 0.186886 -0.565514 0.002820
C -1.135330 -0.895365 0.000532
C -2.270235 -0.061146 -0.002443
C -3.519615 -0.652713 -0.004199
H -2.657875 -3.997260 0.000320
H -4.421550 -0.037400 -0.006594
Cs 3.040928 0.380754 -0.001123
C -2.126385 1.472121 -0.003845
O -1.091392 2.095833 -0.000601
O -3.322730 2.099014 -0.006103
C -3.278201 3.509915 0.009144
H -2.753264 3.905286 -0.874529
H -4.320348 3.854606 0.003948
H -2.768243 3.886927 0.909654
H -4.620707 -2.493305 -0.005161
H 0.390968 -3.783120 0.007825
21
indazole_Cs_O_neut_a
Eopt -3199.221863
Br -2.948892 -2.200632 0.218028
C -2.614863 -0.334883 0.033152
C -1.314059 0.125969 0.141728
C -1.103877 1.508708 -0.004919
C -0.002164 2.409798 0.022350
C 1.391432 2.084714 0.220463
O 2.208674 3.139751 0.233253
C 3.585660 2.874849 0.414783
O 1.818113 0.941475 0.365054
N -0.459174 3.671397 -0.185306
N -1.761369 3.672392 -0.347243
C -2.204021 2.382567 -0.246474
C -3.513692 1.880771 -0.348339
C -3.715816 0.517897 -0.209510
H -0.489402 -0.558134 0.340626
H 3.987185 2.256798 -0.403856
H 4.089070 3.849656 0.415231
H 3.774259 2.362748 1.371138
H -4.354199 2.554684 -0.532965
H -4.719978 0.095856 -0.283718
Cs 2.567312 -1.787239 -
0.177008
20
indazole_deprot_neg_a
Eopt -3179.099369
Br -3.337878 -1.019683 0.001084
C -1.843906 0.161718 0.000361
C -0.566613 -0.370187 -0.001957
C 0.505921 0.540873 -0.002526
C 1.925580 0.472115 -0.002726
C 2.728484 -0.739207 -0.003200
O 4.053164 -0.511128 0.005356
C 4.873277 -1.660260 0.006410
O 2.269341 -1.866843 -0.009999
N 2.429302 1.729191 -0.000892
N 1.457094 2.619525 -0.000773
C 0.269168 1.947289 -0.001216
C -1.045341 2.451160 0.000866
C -2.099516 1.553863 0.001956
H -0.388443 -1.446333 -0.003263
H 4.701462 -2.280958 -0.887217
H 5.910859 -1.301639 0.010740
H 4.695116 -2.283576 0.897032
H -1.229004 3.528952 0.002036
H -3.131421 1.911026 0.003737
20
indazole_deprot_neg_b
Eopt -3179.099111
Br 3.170751 -0.966404 0.000167
C 1.696454 0.239745 -0.000896
C 0.411294 -0.272518 -0.002241
C -0.651351 0.651306 -0.001429
C -2.073995 0.612227 -0.001422
C -2.978294 -0.528195 -0.000008
O -2.292868 -1.695411 0.001425
C -3.074314 -2.870972 0.000491
O -4.189996 -0.500017 0.000465
N -2.549720 1.880011 -0.000809
N -1.561360 2.750352 0.000759
C -0.387976 2.054344 0.000151
C 0.933624 2.538479 0.001159
C 1.974933 1.627003 0.000588
H 0.224363 -1.346826 -0.003647
H -3.718115 -2.926823 -0.891460
H -2.370421 -3.713400 -0.001017
H -3.717076 -2.928761 0.893052
H 1.131142 3.613780 0.002512
H 3.012037 1.968554 0.001584
59
INT_1PPh3OMe_b_neut
Eopt -4328.720962

Br 2.053387 12.136147 4.739893
C 0.468946 11.253689 4.181785
C 0.343499 10.906455 2.817298
C -0.791291 10.265183 2.354247
C -1.806223 9.970692 3.282395
C -1.662852 10.313731 4.650544
C -2.867576 9.825051 5.238390
C -3.246962 9.905363 6.651237
O -4.433387 9.353912 6.910803
C -4.854629 9.396071 8.262963
O -2.551682 10.419738 7.501432
N -3.626293 9.255206 4.303526
N -3.021070 9.348106 3.133054
C -0.511605 10.972636 5.114953
H 1.152929 11.146483 2.125303
H -0.880827 9.995008 1.302090
H -5.834903 8.904926 8.295461
H -4.148434 8.861987 8.916861
H -4.945943 10.433476 8.619329
H -0.408211 11.238325 6.167138
C -3.574052 10.620034 0.620724
O -3.160091 9.263860 0.578734
P -3.525769 8.031941 1.573036
C -3.894284 6.896279 0.127783
C -4.588922 7.401409 -0.980658
C -4.976319 6.565269 -2.025027
C -4.676089 5.203102 -1.980358
C -3.988275 4.686901 -0.884303
C -3.600423 5.526919 0.160790
C -2.142875 7.082464 2.276754
C -2.201701 6.528705 3.560161
C -1.109844 5.818516 4.052636
C 0.045909 5.681144 3.282176
C 0.105147 6.242869 2.007751
C -0.992729 6.932463 1.495794
C -5.175061 7.900188 2.328634
C -5.975873 9.032114 2.509849
C -7.274165 8.892793 2.994005
C -7.768209 7.632725 3.328944
C -6.964214 6.505374 3.165167
C -5.676611 6.633889 2.650002
H -2.851721 11.191483 0.022630
H -4.571246 10.720252 0.165207
H -3.592798 11.022368 1.642029
H -4.830855 8.465328 -1.032157
H -5.513699 6.980837 -2.881520
H -4.976473 4.546398 -2.800917
H -3.744779 3.622179 -0.840083
H -3.058576 5.099598 1.007428
H -3.090465 6.666065 4.180124
H -1.158989 5.379667 5.052115
H 0.905245 5.135063 3.679170
H 1.009272 6.140286 1.402776
H -0.951270 7.348952 0.486030
H -5.590582 10.028225 2.291884
H -7.898026 9.780280 3.124337
H -8.783401 7.528982 3.720071
H -7.343052 5.515100 3.429101
H -5.066708 5.740595 2.499678
59
INT_deprot-indazole_MeOPPh3_neut_a
Eopt -4328.713164
Br -0.071532 14.855789
5.449653
C -0.960826 13.175776 5.352064
C -1.403412 12.579690 6.519576
C -2.055383 11.338459 6.398244
C -2.654421 10.391145 7.272903
C -2.748585 10.490952 8.721168
O -3.369007 9.446717 9.293821
C -3.479689 9.495181 10.701418
O -2.313312 11.422043 9.372394
N -3.132893 9.356881 6.545333
N -2.896245 9.546454 5.261772
C -2.243630 10.735763 5.119907
C -1.777682 11.371120 3.953302
C -1.137223 12.591916 4.074540
H -1.257446 13.043262 7.496070
H -4.052306 10.376252 11.031295
H -4.005639 8.580216 11.003288
H -2.489706 9.527672 11.183023
H -1.914565 10.909008 2.972582
H -0.763221 13.111726 3.190030
C -4.116894 7.786371 3.191326
O -4.675004 6.995662 2.119676
P -4.576561 7.485802 0.602002
C -5.103232 6.056949 -0.332650
C -4.960649 4.778029 0.220592
C -5.328904 3.666407 -0.532694
C -5.829348 3.830116 -1.824378
C -5.968742 5.106525 -2.371242
C -5.608204 6.226794 -1.628568

C -5.674178 8.878764 0.370169
C -6.811034 8.964073 1.186428
C -7.708896 10.010128 0.998920
C -7.480526 10.956700 -0.000741
C -6.352963 10.864753 -0.816976
C -5.441972 9.827737 -0.634661
C -2.897730 7.942363 0.185041
C -2.065661 7.017962 -0.462282
C -0.737298 7.350178 -0.711968
C -0.240701 8.591950 -0.315085
C -1.068362 9.510821 0.331880
C -2.398786 9.192483 0.583982
H -4.425520 7.302152 4.123703
H -3.022043 7.797948 3.128332
H -4.506007 8.812252 3.168301
H -4.570925 4.656015 1.232895
H -5.224764 2.666266 -0.105948
H -6.117278 2.953830 -2.410397
H -6.366165 5.231578 -3.380915
H -5.726244 7.225595 -2.056510
H -6.988714 8.220920 1.967443
H -8.590760 10.087189 1.638698
H -8.188488 11.776815 -0.143148
H -6.177369 11.608286 -1.597555
H -4.555439 9.761449 -1.269537
H -2.455514 6.045494 -0.772249
H -0.086585 6.634543 -1.219284
H 0.803368 8.847477 -0.511813
H -0.674689 10.480460 0.644700
H -3.044894 9.913841 1.090030
18
product_BaseH_neut
Eopt -566.640028
O 1.252437 -0.620900 -1.127324
C 1.493576 0.153311 -0.234647
O 2.634166 0.207735 0.457155
C 3.629751 -0.729822 0.082054
N 0.645830 1.140774 0.199734
N -0.645244 1.139128 -0.213093
C -1.494575 0.159030 0.235485
O -2.632797 0.202697 -0.460971
C -3.627502 -0.733166 -0.079375
O -1.256795 -0.599978 1.141883
H 4.485228 -0.550330 0.744542
H 3.935117 -0.586615 -0.965414
H 3.270538 -1.761726 0.210714
H 0.862545 1.628931 1.063897
H -0.857883 1.605748 -1.090059
H -3.267289 -1.765514 -0.201818
H -3.932498 -0.583793 0.967242
H -4.483449 -0.558567 -0.742629
6
product_CO3HCs
Eopt -284.193749
C -3.570960 0.137012 0.026810
O -2.256261 0.138819 0.020001
O -4.187765 -0.982225 0.078178
O -4.191428 1.254491 -0.018147
Cs -1.994512 2.920340 0.056708
H -3.690938 -1.802801 0.115839
19
product_CsOTs
Eopt -913.887526
S -1.285239 1.398198 -1.285472
O 0.181027 1.394185 -1.488613
O -1.937886 2.688408 -1.603023
O -1.708382 0.815666 0.007613
C -1.919662 0.242680 -2.515786
C -1.194841 -0.908631 -2.830491
C -1.716643 -1.837765 -3.725144
C -2.968633 -1.640924 -4.328727
C -3.676580 -0.476759 -4.008298
C -3.159755 0.458468 -3.111125
H -0.213384 -1.057906 -2.373657
H -1.140591 -2.736551 -3.967553
H -4.650855 -0.297440 -4.473492
H -3.706682 1.373040 -2.869975
C -3.518026 -2.649774 -5.294542
H -4.514465 -2.359449 -5.657592
H -2.859189 -2.764327 -6.171073
H -3.603769 -3.644694 -4.827938
Cs -1.346689 5.124376 0.063305
5
product_HCO3
Eopt -264.045421
C -3.349328 0.137532 0.024095
O -2.122011 0.137532 0.024095
O -4.111868 -1.072192 0.024146
O -4.111868 1.347257 0.024095
H -3.516829 -1.825537 0.024177
19

product_indazole_1Me_3CN
Eopt -510.476415
C 1.781366 -2.113319 -0.000035
C 0.407166 -1.956619 0.000045
C -0.088121 -0.640679 -0.000063
C 0.811339 0.450561 -0.000448
C 2.203129 0.279459 -0.000389
C 2.677538 -1.019245 -0.000218
N 0.044850 1.582144 -0.000706
C 0.513877 2.944468 0.001010
N -1.247769 1.304718 -0.000051
C -1.376473 -0.015304 0.000120
H -0.270291 -2.810017 0.000251
H 2.888170 1.129242 -0.000335
H 3.752950 -1.206055 -0.000152
H -0.364320 3.599517 -0.011377
H 1.108469 3.146056 0.904359
H 1.129173 3.140200 -0.889529
H 2.183694 -3.104798 0.000045
C -2.782513 -0.643517 0.000138
N -3.841608 -1.116717 0.000151
24
product_indazole_1Me_E_7CO2Me
Eopt -645.874441
C -1.609550 2.465924 -0.000012
C -0.244311 2.145934 -0.000156
C 0.248667 0.840565 -0.000130
C -0.715539 -0.212667 -0.000048
C -2.103511 0.126847 0.000107
C -2.783712 -1.119392 0.000104
N -1.924813 -2.107809 -0.000074
N -0.684828 -1.584546 -0.000178
C -2.551494 1.454844 0.000146
H 0.498896 2.945538 -0.000254
H -3.622363 1.672209 0.000268
H -1.916877 3.513723 -0.000010
C 1.735732 0.755607 -0.000108
O 2.462011 1.722122 -0.000961
O 2.201954 -0.488423 0.000903
C 3.612332 -0.644807 0.000847
H 4.058166 -0.185513 0.895321
H 3.800873 -1.725385 0.001634
H 4.057946 -0.186852 -0.894425
H -3.857040 -1.309485 0.000194
C 0.402460 -2.538918 -0.000522
H 1.028907 -2.430923 -0.893908
H 1.028825 -2.431689 0.893011
H -0.068009 -3.528348 -0.000975
24
product_indazole_1Me_neut_a
Eopt -3218.834464
Br 3.547514 -0.960103 0.000730
C 1.961588 0.076110 -0.000423
C 0.736608 -0.566100 -0.001594
C -0.408540 0.248339 -0.001708
C -0.281713 1.655661 -0.001104
C 0.966284 2.295099 0.000213
C 2.087232 1.485136 0.000570
N -1.553971 2.156355 -0.001593
C -1.936065 3.545638 0.002197
N -2.465102 1.196153 -0.001472
C -1.822585 0.035831 -0.002015
C -2.505362 -1.266667 -0.001659
O -3.830584 -1.163246 0.000866
C -4.550629 -2.386729 0.003716
O -1.910054 -2.320873 -0.002917
H 0.647423 -1.652357 -0.002300
H 1.058184 3.382558 0.001419
H 3.081989 1.934567 0.001828
H -3.030687 3.591000 -0.013541
H -1.561550 4.045628 0.907802
H -1.535458 4.056822 -0.885644
H -4.314762 -2.981441 0.898985
H -4.316883 -2.984532 -0.889966
H -5.613521 -2.117147 0.004583
24
product_indazole_1Me_neut_b
Eopt -3218.834038
Br 3.418865 -0.653402 0.000434
C 1.770799 0.280825 -0.000589
C 0.588718 -0.437294 -0.000963
C -0.605471 0.303373 -0.001014
C -0.567570 1.715890 -0.001140
C 0.637658 2.432692 -0.000628
C 1.807413 1.694968 -0.000325
N -1.868848 2.135426 -0.001365
C -2.337718 3.497875 0.001907
N -2.717667 1.119723 -0.000418
C -2.003317 0.002190 -0.000657
C -2.602669 -1.340740 0.000599
O -1.700416 -2.316883 0.000736
C -2.214653 -3.640115 -0.000440

O -3.798994 -1.526616 0.000840
H 0.568153 -1.527012 -0.001109
H 0.660857 3.523781 0.000017
H 2.771876 2.206185 0.000320
H -3.433029 3.474169 -0.013418
H -1.995119 4.020928 0.907122
H -1.970441 4.032835 -0.886340
H -2.826605 -3.824962 -0.896079
H -2.829710 -3.825505 0.892873
H -1.344508 -4.307394 0.000795
24
product_indazole_1Me_Z_7CO2Me
Eopt -645.875177
C 0.657629 2.697334 0.000048
C -0.488924 1.887086 0.000013
C -0.447299 0.491619 -0.000086
C 0.845760 -0.112151 -0.000035
C 2.003727 0.722000 -0.000001
C 3.100026 -0.180772 0.000097
N 2.671096 -1.418978 0.000189
N 1.326428 -1.394559 0.000121
C 1.913440 2.120965 0.000013
H -1.469671 2.362570 -0.000019
H 2.820339 2.730490 0.000028
H 0.544889 3.783481 0.000077
C -1.730499 -0.257924 -0.000441
O -1.846569 -1.460980 -0.001826
O -2.789338 0.555383 0.000843
C -4.061943 -0.070932 0.000432
H -4.192796 -0.699316 0.894027
H -4.801484 0.738808 0.001660
H -4.193334 -0.697082 -0.894651
H 4.166817 0.042853 0.000146
C 0.659681 -2.678769 0.000431
H 0.031842 -2.800489 0.890480
H 0.032149 -2.801110 -0.889739
H 1.456254 -3.431412 0.000855
19
product_indazole_2Me_3CN
Eopt -510.468741
C -2.649022 1.201133 0.000231
C -1.311747 1.533394 0.000149
C -0.378348 0.475086 0.000100
C 1.026612 0.365787 0.000065
N 1.305387 -0.969304 -0.000261
C 2.624977 -1.561125 -0.000130
N 0.236168 -1.730031 -0.000063
C -0.814522 -0.884808 0.000109
C -2.192717 -1.201469 0.000195
C -3.085174 -0.153652 0.000255
H -0.982153 2.574531 0.000163
H 3.178835 -1.246770 -0.895687
H 3.176510 -1.251776 0.898631
H 2.496686 -2.648511 -0.003316
H -2.520266 -2.242901 0.000201
H -4.158391 -0.359762 0.000328
H -3.400155 1.994767 0.000302
C 2.029647 1.357131 0.000127
N 2.845688 2.183843 -0.000709
24
product_indazole_2Me_E_7CO2Me
Eopt -645.872528
C 0.372136 2.919550 -0.000024
C -0.811045 2.156016 -0.000074
C -0.784420 0.764676 -0.000048
C 0.487059 0.152903 0.000035
C 1.684957 0.909775 0.000075
C 2.713897 -0.081867 0.000157
N 2.195649 -1.291681 0.000182
N 0.865196 -1.145709 0.000138
C 1.620061 2.312954 0.000046
H -1.779371 2.659777 -0.000136
H 2.532939 2.913471 0.000081
H 0.298514 4.009260 -0.000044
C -1.987143 -0.088921 -0.000108
O -2.907124 0.703505 0.000263
O -2.156722 -1.406573 -0.000443
C -3.498647 -1.872529 -0.000396
H -4.035327 -1.523992 -0.895200
H -3.442793 -2.967493 -0.000606
H -4.035146 -1.524323 0.894645
H 3.794737 0.059067 0.000207
C 2.936750 -2.561196 0.000246
H 2.682315 -3.122674 0.874835
H 2.679591 -3.124353 -0.872465
H 3.987782 -2.360631 -0.001585
24
product_indazole_2Me_neut_a
Eopt -3218.827485
Br 3.648806 -0.854624 0.000482
C 2.069533 0.190831 0.001028
C 0.846136 -0.441132 -0.003406

C -0.294878 0.390621 -0.003154
C -1.686363 0.173163 -0.004934
N -2.253136 1.414075 -0.003329
C -3.661336 1.755069 -0.006139
N -1.377478 2.398196 0.000181
C -0.166687 1.809884 0.000819
C 1.106697 2.423556 0.005365
C 2.213160 1.607091 0.005621
C -2.362605 -1.125746 -0.004904
O -3.692119 -1.038457 0.009926
C -4.403571 -2.269679 0.012568
O -1.758558 -2.173785 -0.015341
H 0.747160 -1.526267 -0.007075
H -4.150390 1.345960 -0.898822
H -4.152779 1.351057 0.887511
H -3.719977 2.848399 -0.009360
H 1.202981 3.510984 0.008892
H 3.215157 2.040204 0.009697
H -4.170490 -2.858072 -0.886825
H -5.467711 -2.006431 0.023864
H -4.152974 -2.863896 0.903421
24
product_indazole_2Me_neut_b
Eopt -3218.829195
Br -3.501845 0.621625 -0.000731
C -1.881384 -0.358775 0.000248
C -0.684682 0.322398 -0.001976
C 0.489197 -0.462290 -0.001511
C 1.870694 -0.188451 -0.001429
N 2.487437 -1.405301 -0.000795
C 3.908336 -1.688771 -0.002450
N 1.652501 -2.424205 0.000442
C 0.418798 -1.885594 0.000471
C -0.828594 -2.550523 0.002774
C -1.967329 -1.779707 0.002836
C 2.493579 1.136870 0.001057
O 1.617963 2.141232 0.001085
C 2.165651 3.453526 0.001598
O 3.693294 1.291958 0.000959
H -0.629892 1.410663 -0.004122
H 4.381321 -1.258922 -0.894045
H 4.381984 -1.266320 0.892281
H 4.011373 -2.778809 -0.007026
H -0.880601 -3.640970 0.004778
H -2.950897 -2.253195 0.005212
H 2.783492 3.619188 0.896176
H 1.311040 4.140154 0.002920
H 2.781539 3.620275 -0.894164
24
product_indazole_2Me_Z_7CO2Me
Eopt -645.871954
C -0.065102 2.801865 -0.000087
C -1.089640 1.835831 -0.000137
C -0.810349 0.472550 -0.000111
C 0.551207 0.102290 -0.000028
C 1.591427 1.064452 0.000012
C 2.783596 0.276540 0.000094
N 2.494082 -1.007365 0.000119
N 1.159275 -1.105862 0.000075
C 1.272350 2.432412 -0.000017
H -2.133451 2.155029 -0.000199
H 2.060751 3.188977 0.000018
H -0.335734 3.859999 -0.000107
C -1.837718 -0.585600 -0.000171
O -1.570891 -1.770129 -0.000513
O -3.080377 -0.115746 0.000207
C -4.122551 -1.081017 0.000244
H -4.067230 -1.718518 0.895068
H -5.062678 -0.516901 0.000480
H -4.067523 -1.718266 -0.894777
H 3.820762 0.611746 0.000144
C 3.453765 -2.120877 0.000183
H 3.305719 -2.719273 0.874772
H 3.303346 -2.721418 -0.872528
H 4.450773 -1.732457 -0.001648
35
product_OPPh3_neut
Eopt -1109.932596
O -0.047379 -0.132019 2.417611
P -0.015867 -0.033217 0.914181
C -1.628779 -0.443911 0.179907
C -2.699352 -0.606389 1.065308
C -3.964283 -0.925955 0.577538
C -4.162994 -1.086862 -0.793171
C -3.093866 -0.931309 -1.679373
C -1.827273 -0.612000 -1.196340
C 1.192948 -1.172518 0.175158
C 1.596145 -2.257699 0.960813
C 2.502743 -3.187414 0.450691
C 3.007746 -3.032677 -0.839296
C 2.613904 -1.945905 -1.621315
C 1.710732 -1.012450 -1.114807

C 0.432089 1.613036 0.282429
C 1.745249 2.059849 0.490135
C 2.114650 3.344239 0.097297
C 1.177046 4.190192 -0.500671
C -0.131556 3.752048 -0.702098
C -0.506248 2.468114 -0.309120
H -2.519929 -0.482274 2.136420
H -4.800732 -1.050290 1.272655
H -5.153942 -1.337868 -1.177295
H -3.247960 -1.062547 -2.752884
H -0.995114 -0.494348 -1.895820
H 1.198191 -2.356396 1.974727
H 2.816330 -4.033314 1.067362
H 3.719293 -3.762070 -1.237378
H 3.017940 -1.819609 -2.627971
H 1.418711 -0.152133 -1.723665
H 2.481082 1.401862 0.958953
H 3.140683 3.685670 0.257395
H 1.470680 5.197618 -0.811442
H -0.866933 4.414832 -1.165771
H -1.534662 2.130945 -0.467403
18
product_OTs
Eopt -893.745339
S -1.277489 1.384725 -1.477901
O 0.101658 1.384725 -1.477901
O -1.997169 2.569545 -1.477901
O -1.697108 0.753425 0.000000
C -1.897552 0.273893 -2.528496
C -1.158030 -0.861616 -2.892330
C -1.672932 -1.799695 -3.779113
C -2.950197 -1.625138 -4.328016
C -3.695903 -0.491815 -3.975251
C -3.174098 0.440232 -3.087181
H -0.146871 -1.001810 -2.468435
H -1.075929 -2.682998 -4.052639
H -4.697874 -0.340879 -4.404967
H -3.764933 1.334993 -2.818732
C -3.509019 -2.631215 -5.258767
H -4.248309 -2.167173 -5.957193
H -2.699909 -3.110246 -5.863689
H -4.031889 -3.432522 -4.676374
17
rxt_Base_neg_b
Eopt -566.122257
O 1.793958 -1.320948 0.011226
C 1.702123 -0.079464 -0.003255
O 2.844043 0.691772 -0.006815
C 4.044633 -0.022022 0.011762
N 0.617201 0.670424 -0.019915
N -0.492491 -0.151905 -0.019830
C -1.747417 0.303165 0.000303
O -2.609600 -0.760904 -0.004780
C -3.973211 -0.426163 0.005918
O -2.133837 1.458600 0.017400
H 4.856725 0.720968 0.007014
H 4.147738 -0.657070 0.910019
H 4.159548 -0.680992 -0.868417
H -0.310775 -1.156598 -0.020275
H -4.254667 0.136340 0.912060
H -4.256088 0.180623 -0.870703
H -4.528728 -1.374161 -0.016113
6
rxt_CO3Cs2
Eopt -303.805659
C -3.349328 0.137532 0.024095
O -2.122011 0.137532 0.024095
O -4.111868 -1.072192 0.024146
O -4.111868 1.347257 0.024095
Cs -2.247360 3.710245 0.024095
Cs -2.246174 -3.434243
0.024244
1
rxt_Cs_pos
Eopt -20.097396
Cs 0.000000 0.000000 0.000000
16
rxt_indazole_1H_3CN
Eopt -471.237830
C -1.980946 -1.628007 0.000111
C -2.775267 -0.465271 0.000161
C -2.210977 0.806780 0.000135
C -0.801743 0.877391 0.000052
C 0.008726 -0.284914 0.000012
C 1.341779 0.229906 -0.000070
N 1.333446 1.546022 -0.000095
N 0.050558 1.927611 -0.000051
C -0.595431 -1.553031 0.000041
H -3.863187 -0.553933 0.000223
H -0.216728 2.906559 0.000012
H 0.012995 -2.460656 0.000006
H -2.471540 -2.603818 0.000131

H -2.816617 1.688880 0.000176
C 2.672017 -0.546025 -0.000140
N 3.674014 -1.130492 -0.000194
21
rxt_indazole_1H_E_7CO2Me_a
Eopt -606.645761
C -1.606217 2.245461 0.000000
C -0.224627 1.973229 0.000050
C 0.264809 0.670543 0.000024
C -0.690578 -0.367803 -0.000059
C -2.083381 -0.107199 -0.000099
C -2.673016 -1.408891 -0.000181
N -1.744363 -2.341540 -0.000206
N -0.562039 -1.714226 -0.000162
C -2.541630 1.220631 -0.000070
H 0.488983 2.799177 0.000112
H 0.314883 -2.224900 -0.000099
H -3.611808 1.441278 -0.000105
H -1.940514 3.285237 0.000020
C 1.697840 0.321845 0.000084
O 2.259851 1.398157 -0.000287
O 2.342359 -0.839861 0.000419
C 3.761484 -0.776917 0.000372
H 4.131368 -0.254721 0.895176
H 4.114232 -1.815008 0.000583
H 4.131322 -0.255095 -0.894669
H -3.729425 -1.677364 -0.000231
21
rxt_indazole_1H_neut
Eopt -3179.596002
Br 3.341603 -1.028470 -0.000044
C 1.856580 0.147692 -0.000006
C 2.109313 1.539752 0.000468
C 1.067413 2.448381 0.000504
C -0.233499 1.925640 0.000159
C -0.488219 0.535088 -0.000240
C -1.917510 0.448974 -0.000533
C -2.713787 -0.789540 -0.000882
O -4.023521 -0.567719 -0.002568
C -4.851248 -1.721539 0.001898
O -2.213531 -1.891453 0.000455
N -2.457415 1.658956 -0.000050
N -1.458404 2.524572 0.000426
C 0.578508 -0.380131 -0.000391
H 3.140541 1.897836 0.000897
H 1.256025 3.523341 0.001178
H -4.675272 -2.329288 0.902153
H -4.666352 -2.343134 -0.886876
H -5.885337 -1.356922 -0.005943
H -1.654935 3.521137 -0.000581
H 0.391058 -1.453756 -0.000900
21
rxt_indazole_1H_Z_7CO2Me_a
Eopt -606.646800
C -1.120186 2.392949 0.000029
C 0.166947 1.805587 0.000139
C 0.317986 0.431415 0.000255
C -0.853965 -0.338105 0.000151
C -2.140995 0.246765 -0.000034
C -3.027795 -0.877457 -0.000046
N -2.342725 -2.011579 -0.000166
N -1.061668 -1.685685 -0.000119
C -2.283268 1.645082 -0.000032
H 1.049736 2.447724 0.000044
H -0.355745 -2.416053 0.001235
H -3.271703 2.104217 0.000028
H -1.196804 3.460202 -0.000008
C 1.715205 -0.216179 -0.000062
O 1.832752 -1.469077 0.000049
O 2.883055 0.609063 -0.000484
C 4.050800 -0.216329 0.000141
H 4.051109 -0.834480 -0.873237
H 4.924335 0.401599 0.000061
H 4.050722 -0.833708 0.874065
H -4.095723 -0.810899 0.000032
16
rxt_indazole_2H_3CN
Eopt -471.229329
C -2.057831 -1.559925 0.000113
C -2.818811 -0.375101 0.000163
C -2.218624 0.880413 0.000137
C -0.807953 0.910975 0.000054
C -0.030819 -0.273878 0.000014
C 1.316317 0.202879 -0.000068
N 1.345363 1.518701 -0.000093
N 0.073828 1.936568 -0.000049
C -0.670745 -1.524325 0.000043
H -3.908810 -0.432831 0.000225
H -0.088339 -2.448863 0.000008
H -2.575938 -2.521411 0.000133
H -2.798970 1.779356 0.000178
C 2.623982 -0.610516 -0.000138

N 3.608977 -1.223202 -0.000192
H 2.161786 2.096154 -0.000136
21
rxt_indazole_2H_E_7CO2Me_a
Eopt -606.633836
C -1.510617 2.305998 0.000002
C -0.138247 1.990561 0.000052
C 0.310084 0.673163 0.000026
C -0.677405 -0.334702 -0.000057
C -2.061347 -0.030536 -0.000097
C -2.691525 -1.313090 -0.000179
N -1.792586 -2.274412 -0.000204
N -0.591165 -1.684495 -0.000160
C -2.477718 1.311016 -0.000068
H 0.600921 2.793717 0.000114
H -3.540448 1.565125 -0.000103
H -1.812133 3.355749 0.000022
C 1.731471 0.279683 0.000086
O 2.326969 1.337836 -0.000285
O 2.339231 -0.901669 0.000421
C 3.759632 -0.883273 0.000374
H 4.145716 -0.372937 0.895178
H 4.079643 -1.931919 0.000584
H 4.145658 -0.373309 -0.894667
H -3.755836 -1.548292 -0.000229
H -1.972837 -3.258032 -0.000248
21
rxt_indazole_2H_neut
Eopt -3179.591003
Br -3.388749 -0.991225 0.000866
C -1.875710 0.146562 0.000532
C -0.616395 -0.408247 -0.002009
C 0.470379 0.493427 -0.002318
C 1.869774 0.365521 -0.003765
C 2.719459 -0.824180 -0.003634
O 4.013280 -0.497767 0.004488
C 4.939168 -1.577549 0.008243
O 2.298145 -1.955859 -0.009387
N 2.343441 1.633086 -0.003162
N 1.429110 2.574554 -0.000990
C 0.257143 1.907749 -0.000268
C -1.054238 2.437837 0.002442
C -2.105431 1.552653 0.002657
H -0.452485 -1.485948 -0.003517
H 4.815289 -2.201322 -0.889108
H 5.937550 -1.124929 0.015143
H 4.804331 -2.204046 0.902172
H 3.330708 1.883733 -0.006389
H -1.220532 3.516761 0.004038
H -3.132794 1.921521 0.004316
21
rxt_indazole_2H_Z_7CO2Me_a
Eopt -606.633215
C 0.912758 2.451988 -0.000071
C -0.327124 1.784460 -0.000121
C -0.410663 0.395374 -0.000095
C 0.808428 -0.315079 -0.000012
C 2.062453 0.344645 0.000028
C 3.009711 -0.725296 0.000110
N 2.397369 -1.890315 0.000135
N 1.082635 -1.639536 0.000091
C 2.108778 1.748561 -0.000001
H -1.252558 2.363254 -0.000183
H 3.066306 2.274969 0.000034
H 0.925583 3.544108 -0.000091
C -1.677150 -0.360389 -0.000155
O -1.726405 -1.573599 -0.000497
O -2.755597 0.415445 0.000223
C -4.012315 -0.246775 0.000260
H -4.124089 -0.876834 0.895084
H -4.774137 0.541700 0.000496
H -4.124307 -0.876515 -0.894761
H 4.098313 -0.670319 0.000160
H 2.831611 -2.791111 0.000179
39
rxt_MeOPPh3_pos
Eopt -1149.596009
C -0.758433 0.787578 2.962781
O 0.029306 -0.089794 2.152401
P 0.007535 -0.023520 0.550558
C 1.296553 -1.165392 0.081490
C 2.363903 -1.413409 0.954818
C 3.385623 -2.268167 0.550623
C 3.341984 -2.864398 -0.711041
C 2.278415 -2.609699 -1.576999
C 1.246825 -1.759865 -1.186094
C -1.596391 -0.569673 -0.025244
C -2.276659 -1.528916 0.739885
C -3.496374 -2.019545 0.288655
C -4.029090 -1.566881 -0.920035
C -3.343733 -0.621680 -1.684492
C -2.125464 -0.115688 -1.241000

C 0.362916 1.627839 -0.036872
C 1.649774 1.933170 -0.503714
C 1.940347 3.229970 -0.912058
C 0.958071 4.219344 -0.851992
C -0.322222 3.915845 -0.385375
C -0.625907 2.620286 0.021027
H -0.654008 0.427010 3.994436
H -0.381379 1.818223 2.897567
H -1.819143 0.753556 2.670429
H 2.389403 -0.948385 1.941841
H 4.217967 -2.472962 1.229054
H 4.145960 -3.535204 -1.021105
H 2.246449 -3.082918 -2.563714
H 0.409872 -1.566388 -1.862376
H -1.854880 -1.885731 1.680707
H -4.034349 -2.760496 0.883996
H -4.987130 -1.958514 -1.269799
H -3.763525 -0.273755 -2.629731
H -1.593402 0.626065 -1.841330
H 2.419233 1.158145 -0.554324
H 2.939999 3.470114 -1.281218
H 1.191779 5.238086 -1.175364
H -1.087979 4.692025 -0.341121
H -1.633178 2.387948 0.378314
22
rxt_MeOTs
Eopt -933.441942
S -1.277489 1.384725 -1.477901
O 0.101658 1.384725 -1.477901
O -1.997169 2.569545 -1.477901
O -1.697108 0.753425 0.000000
C -1.897552 0.273893 -2.528496
C -1.158030 -0.861616 -2.892330
C -1.672932 -1.799695 -3.779113
C -2.950197 -1.625138 -4.328016
C -3.695903 -0.491815 -3.975251
C -3.174098 0.440232 -3.087181
H -0.146871 -1.001810 -2.468435
H -1.075929 -2.682998 -4.052639
H -4.697874 -0.340879 -4.404967
H -3.764933 1.334993 -2.818732
C -3.509019 -2.631215 -5.258767
H -4.248309 -2.167173 -5.957193
H -2.699909 -3.110246 -5.863689
H -4.031889 -3.432522 -4.676374
C -2.509602 1.570334 0.847002
H -2.665170 1.071705 1.780848
H -2.018017 2.504980 1.019282
H -3.453569 1.745569 0.374648
54
TS1a_3CN_opt_a
Eopt -1620.337385
C -7.108390 0.269958 0.330365
C -6.402628 -0.681511 -0.380668
C -5.007166 -0.510529 -0.461599
C -3.921975 -1.204509 -1.057836
N -2.792521 -0.521153 -0.774554
N -3.025668 0.547402 -0.051708
C -4.368718 0.598945 0.166419
C -5.121338 1.550149 0.883050
C -6.490019 1.378860 0.960789
H -6.893176 -1.529195 -0.860171
H -4.633686 2.401282 1.364388
H -7.105689 2.095836 1.507499
C -0.742239 -0.779142 -1.083744
O 1.039304 -0.898480 -1.276723
P 1.931586 -0.147923 -0.252291
C 3.636584 -0.621824 -0.579865
C 3.876697 -1.743614 -1.380360
C 5.188874 -2.142149 -1.628746
C 6.252295 -1.425459 -1.081042
C 6.009908 -0.304276 -0.284943
C 4.702967 0.102807 -0.032818
C 1.756637 1.635443 -0.446661
C 1.400771 2.116394 -1.713498
C 1.288274 3.488139 -1.923318
C 1.533563 4.377217 -0.876224
C 1.888551 3.898207 0.385066
C 1.998431 2.527180 0.605827
C 1.534333 -0.560260 1.459128
C 2.343555 -1.446248 2.182008
C 1.984254 -1.814638 3.476809
C 0.818416 -1.307511 4.049362
C 0.006577 -0.429919 3.329084
C 0.359734 -0.052361 2.036968
H -0.942125 -1.142497 -2.086712
H -0.799314 -1.486515 -0.261749
H -0.774479 0.289219 -0.884337
H 3.034317 -2.292610 -1.806553
H 5.380152 -3.016821 -2.255014
H 7.280203 -1.740072 -1.278282
H 6.844634 0.259099 0.138689

H 4.516683 0.984508 0.585933
H 1.204674 1.414004 -2.527437
H 1.003517 3.864644 -2.908613
H 1.441950 5.453331 -1.043504
H 2.073862 4.595844 1.205163
H 2.263854 2.154117 1.598116
H 3.256880 -1.847167 1.735969
H 2.619512 -2.502365 4.039879
H 0.538402 -1.598762 5.064848
H -0.910648 -0.036832 3.773603
H -0.288664 0.630312 1.482046
H -8.170339 0.164984 0.408749
C -3.977983 -2.504199 -1.882014
N -4.020171 -3.483186 -2.502824
54
TS1a_3CN_opt_c
Eopt -1620.339578
C -7.434805 0.020538 -0.064995
C -6.268325 0.282781 0.630334
C -5.044158 0.076349 -0.035103
C -3.662350 0.206091 0.267182
N -2.953984 -0.159414 -0.831283
N -3.732847 -0.514214 -1.816657
C -5.019319 -0.388152 -1.383664
C -6.219848 -0.649399 -2.075403
C -7.410864 -0.441151 -1.406133
H -6.296072 0.638543 1.663487
H -6.196220 -1.004926 -3.108397
H -8.358473 -0.634817 -1.915870
C -0.883366 -0.258199 -1.104969
O 0.875582 -0.389570 -1.455334
P 1.923203 -0.110467 -0.344994
C 1.852157 -1.390228 0.922646
C 1.358243 -2.645212 0.543461
C 1.318781 -3.681138 1.473172
C 1.774501 -3.468703 2.774545
C 2.268603 -2.219537 3.151648
C 2.307429 -1.176047 2.229825
C 3.545194 -0.131168 -1.125394
C 3.619122 -0.166619 -2.521385
C 4.866765 -0.171731 -3.143029
C 6.030088 -0.141369 -2.375196
C 5.953163 -0.107929 -0.981156
C 4.712236 -0.104357 -0.351147
C 1.686350 1.486995 0.455085
C 0.677501 1.630462 1.418914
C 0.411182 2.883912 1.962147
C 1.153083 3.992966 1.554038
C 2.158312 3.852785 0.596705
C 2.424745 2.603606 0.040969
H -0.885331 -0.914799 -0.238835
H -0.884325 0.819445 -0.966090
H -1.151941 -0.666194 -2.075849
H 1.002002 -2.803971 -0.477419
H 0.927851 -4.658341 1.180158
H 1.740491 -4.282810 3.502889
H 2.619849 -2.053658 4.172751
H 2.683112 -0.196062 2.533341
H 2.699215 -0.193083 -3.109355
H 4.928567 -0.200494 -4.233522
H 7.006764 -0.146100 -2.865560
H 6.866164 -0.087625 -0.381475
H 4.655245 -0.081709 0.740443
H 0.090828 0.770128 1.749555
H -0.385985 2.987102 2.701657
H 0.944782 4.975614 1.984769
H 2.737912 4.722197 0.277567
H 3.208112 2.497645 -0.713526
H -8.400115 0.171320 0.425640
C -3.025516 0.621469 1.455375
N -2.498676 0.961354 2.436567
59
TS1a_E_7CO2Me_opt_c
Eopt -1755.727817
C 6.461412 -2.235266 -0.140830
C 5.135533 -2.633695 -0.186281
C 4.136320 -1.645530 -0.131469
C 2.722415 -1.628834 -0.131232
N 2.328254 -0.357539 -0.045322
N 3.348536 0.491246 0.010055
C 4.474491 -0.248810 -0.043113
C 5.846369 0.137800 -0.004679
C 6.802528 -0.869894 -0.044199
H 4.872880 -3.693021 -0.258797
H 7.852645 -0.571188 -0.006711
C 0.348779 0.407718 0.004072
O -1.263595 1.153312 0.070324
P -2.548455 0.284229 0.035995
C -2.700769 -0.671365 1.556756
C -1.997404 -0.221762 2.681209
C -2.108081 -0.913453 3.885115
C -2.920833 -2.044008 3.969277

C -3.625212 -2.489099 2.849834
C -3.515660 -1.807370 1.640751
C -3.940631 1.413514 -0.121783
C -3.676856 2.774331 -0.308138
C -4.738266 3.669324 -0.431991
C -6.052162 3.207671 -0.370732
C -6.312391 1.848403 -0.183551
C -5.259591 0.947062 -0.056627
C -2.568796 -0.861644 -1.356232
C -1.865017 -2.073013 -1.280384
C -1.798822 -2.903868 -2.395499
C -2.431413 -2.533582 -3.583218
C -3.129663 -1.328876 -3.660870
C -3.198428 -0.489827 -2.551272
H 0.165006 -0.427337 0.674885
H 0.352790 0.243762 -1.069914
H 0.836952 1.294365 0.399371
H -1.360782 0.663100 2.606579
H -1.554666 -0.568332 4.761576
H -3.003413 -2.586219 4.914578
H -4.259252 -3.376240 2.917040
H -4.058425 -2.165533 0.762674
H -2.642270 3.121500 -0.350755
H -4.535574 4.733285 -0.575939
H -6.882075 3.912169 -0.467670
H -7.342745 1.488501 -0.134284
H -5.471743 -0.115190 0.090846
H -1.364644 -2.370837 -0.355306
H -1.248156 -3.845584 -2.336230
H -2.378729 -3.189557 -4.455723
H -3.623917 -1.038302 -4.590804
H -3.742896 0.455421 -2.616161
H 7.255975 -2.980974 -0.179927
H 2.017714 -2.456298 -0.187895
C 6.340782 1.528439 0.095970
O 7.502657 1.818321 0.307663
O 5.413773 2.428605 -0.100613
C 5.835536 3.779227 -0.033840
H 6.601473 3.988341 -0.790403
H 4.947989 4.389781 -0.221060
H 6.247436 4.011516 0.955713
59
TS1a_E_7CO2Me_opt_d
Eopt -1755.733685
C 6.484028 -1.186650 -0.356518
C 5.690876 -2.150570 -0.957917
C 4.302162 -1.939022 -1.014928
C 3.187780 -2.648342 -1.522018
N 2.097476 -1.922933 -1.283434
N 2.375189 -0.779702 -0.652934
C 3.707714 -0.747995 -0.465407
C 4.542090 0.227639 0.158369
C 5.910339 -0.020652 0.190109
H 6.137184 -3.056506 -1.378338
H 6.547678 0.728459 0.665568
C 0.005781 -1.960130 -1.286964
O -1.774424 -1.738749 -1.113822
P -2.203228 -0.488128 -0.297123
C -1.708680 1.022426 -1.149396
C -1.631757 0.984847 -2.547770
C -1.274011 2.132502 -3.249629
C -0.998102 3.314869 -2.561565
C -1.079323 3.353681 -1.169671
C -1.432194 2.208435 -0.458860
C -3.997628 -0.526909 -0.162113
C -4.667617 -1.721257 -0.447195
C -6.055610 -1.774348 -0.332919
C -6.768033 -0.643082 0.063648
C -6.097159 0.548701 0.345043
C -4.711342 0.612113 0.231456
C -1.495297 -0.430535 1.363558
C -0.123897 -0.169111 1.527048
C 0.424785 -0.169297 2.807119
C -0.377670 -0.423752 3.920039
C -1.737430 -0.687190 3.757010
C -2.298384 -0.693764 2.481626
H 0.229424 -0.911218 -1.451708
H 0.133967 -2.396609 -0.301782
H -0.072021 -2.625369 -2.144283
H -1.841086 0.053720 -3.080370
H -1.204918 2.102649 -4.339560
H -0.712859 4.212815 -3.115342
H -0.858198 4.278895 -0.632244
H -1.484159 2.237733 0.632125
H -4.097865 -2.598200 -0.761880
H -6.582725 -2.704877 -0.556934
H -7.856504 -0.687870 0.151268
H -6.657682 1.435395 0.650403
H -4.188000 1.547662 0.446831
H 0.543435 0.011590 0.676079
H 1.492518 0.028634 2.926802
H 0.060408 -0.419310 4.921422

H -2.367179 -0.889656 4.626579
H -3.364244 -0.900461 2.358769
H 7.566401 -1.325914 -0.298421
H 3.140385 -3.612366 -2.029073
C 4.075411 1.486810 0.784256
O 4.809098 2.330411 1.253330
O 2.745070 1.613523 0.798213
C 2.235087 2.797633 1.380220
H 2.611318 3.690914 0.858571
H 1.143938 2.744702 1.282041
H 2.511088 2.871760 2.443340
59
TS1a_E_SN2_neut_c
Eopt -4328.698859
Br 7.799449 -0.086475 0.071661
C 6.048538 -0.719929 -0.308452
C 4.966557 0.091857 -0.025887
C 3.687663 -0.418057 -0.324267
C 2.343025 0.030376 -0.217317
C 1.797234 1.284261 0.275705
O 2.758596 2.107311 0.715439
C 2.330212 3.363351 1.211978
O 0.619894 1.593618 0.302325
N 1.543755 -0.954334 -0.691169
N 2.222191 -1.995848 -1.095031
C 3.541056 -1.717855 -0.892777
C 4.666985 -2.518094 -1.166895
C 5.918613 -2.013035 -0.872457
H 5.092357 1.083751 0.408621
H 1.703308 3.242129 2.108812
H 3.240043 3.919287 1.469357
H 1.755475 3.915893 0.453757
H 4.550530 -3.513479 -1.602143
H 6.814440 -2.604378 -1.071713
C -0.547527 -1.085122 -0.877523
O -2.324703 -1.271638 -1.097317
P -3.276352 -0.370878 -0.266428
C -3.124614 1.350040 -0.781655
C -2.682822 1.595739 -2.087835
C -2.569148 2.906817 -2.542384
C -2.899013 3.968735 -1.699810
C -3.342086 3.723694 -0.399702
C -3.453897 2.415474 0.065220
C -4.956602 -0.940969 -0.568641
C -5.145155 -2.149134 -1.247159
C -6.439509 -2.612261 -1.477855
C -7.535444 -1.873626 -1.033568
C -7.344221 -0.666288 -0.358256
C -6.055794 -0.194882 -0.124865
C -2.947344 -0.450581 1.505116
C -1.839693 0.234025 2.029598
C -1.531538 0.107796 3.381528
C -2.319500 -0.692221 4.210242
C -3.417720 -1.375370 3.688250
C -3.733047 -1.259708 2.336195
H -0.516593 0.001031 -0.862420
H -0.503377 -1.628985 0.062130
H -0.326598 -1.622239 -1.795890
H -2.419554 0.758766 -2.739209
H -2.217064 3.100028 -3.558441
H -2.805893 4.996999 -2.058153
H -3.594878 4.556913 0.260191
H -3.786165 2.227573 1.089067
H -4.276919 -2.714700 -1.592117
H -6.591169 -3.555271 -2.008516
H -8.549044 -2.239347 -1.216175
H -8.204704 -0.087526 -0.014367
H -5.907365 0.751829 0.400988
H -1.207447 0.856429 1.389279
H -0.667205 0.638245 3.788396
H -2.074632 -0.784919 5.271383
H -4.033619 -2.003202 4.336424
H -4.592538 -1.798284 1.929684
59
TS1a_Z_7CO2Me_opt_a
Eopt -1755.732096
C 6.525262 -1.597511 -0.710821
C 5.592634 -2.509081 -1.182045
C 4.230254 -2.177994 -1.109124
C 3.015353 -2.813241 -1.458700
N 2.030511 -1.992667 -1.122063
N 2.450197 -0.834277 -0.609184
C 3.794484 -0.907005 -0.576931
C 4.776265 0.022066 -0.112221
C 6.115007 -0.360065 -0.177413
H 5.913994 -3.468587 -1.598142
H 6.868019 0.341102 0.185418
C -0.061572 -1.827317 -1.156143
O -1.809319 -1.552035 -1.091435
P -2.361816 -0.367560 -0.251918
C -4.143844 -0.343821 -0.497801
C -4.666780 -0.887256 -1.676620

C	-6.041058	-0.844610	-1.900953
C	-6.886135	-0.263535	-0.955129
C	-6.362218	0.274283	0.221434
C	-4.989947	0.235062	0.455042
C	-2.020999	-0.619489	1.500278
C	-2.066777	-1.934690	1.983965
C	-1.847578	-2.180088	3.336066
C	-1.588602	-1.119261	4.205790
C	-1.545165	0.189133	3.725619
C	-1.757392	0.443569	2.371851
C	-1.704243	1.247913	-0.718675
C	-2.550258	2.202430	-1.301740
C	-2.045334	3.447161	-1.671412
C	-0.700732	3.747017	-1.454958
C	0.145347	2.800336	-0.876091
C	-0.347138	1.549325	-0.512763
H	-0.165106	-2.856333	-1.490956
H	0.141451	-1.055711	-1.892605
H	0.149271	-1.589700	-0.127224
H	-3.995554	-1.346887	-2.405386
H	-6.454439	-1.269936	-2.818605
H	-7.963837	-0.232767	-1.134399
H	-7.026539	0.723183	0.963630
H	-4.579338	0.653102	1.377849
H	-2.268291	-2.762419	1.299260
H	-1.876471	-3.204972	3.713510
H	-1.415879	-1.315585	5.266955
H	-1.339995	1.017881	4.407291
H	-1.713548	1.468055	1.994775
H	-3.606830	1.976207	-1.463968
H	-2.709162	4.187079	-2.125056
H	-0.309257	4.727807	-1.737577
H	1.197939	3.012133	-0.677684
H	0.355595	0.835179	-0.074832
C	4.389669	1.353997	0.383621
O	3.369872	1.941730	0.098790
O	5.327908	1.894231	1.168196
C	5.102100	3.223688	1.591092
H	4.171901	3.303391	2.174604
H	5.958935	3.499760	2.218638
H	5.036340	3.910892	0.732690
H	7.591843	-1.831699	-0.754550
H	2.829947	-3.796661	-1.891861

59

TS1a_Z_7CO2Me_opt_b

Eopt -1755.727017

C	6.489136	2.197610	0.156448
C	5.192665	2.682008	0.179136
C	4.130919	1.771450	0.047998
C	2.720163	1.853930	0.025032
N	2.239499	0.622423	-0.130886
N	3.200844	-0.297137	-0.215219
C	4.374635	0.359902	-0.109428
C	5.719894	-0.120338	-0.129759
C	6.741150	0.817891	0.006886
H	5.000479	3.752962	0.297753
H	7.773642	0.462228	-0.008514
C	0.284452	-0.197152	-0.278488
O	-1.259796	-1.019354	-0.440624
P	-2.589513	-0.301913	-0.077933
C	-3.926950	-1.310562	-0.730407
C	-3.614921	-2.353351	-1.608351
C	-4.639111	-3.138122	-2.132645
C	-5.965174	-2.885103	-1.782554
C	-6.274463	-1.844134	-0.903357
C	-5.257037	-1.054131	-0.373249
C	-2.755315	-0.150161	1.710546
C	-2.028393	-1.046521	2.503856
C	-2.154711	-0.998621	3.889096
C	-3.005972	-0.063422	4.482296
C	-3.730913	0.829236	3.689532
C	-3.608216	0.790844	2.303956
C	-2.700005	1.350586	-0.793910
C	-3.364665	1.543818	-2.011492
C	-3.362672	2.801235	-2.610902
C	-2.693291	3.862253	-2.000919
C	-2.026310	3.672201	-0.790103
C	-2.027589	2.419886	-0.184777
H	0.865771	-1.099565	-0.447731
H	0.147041	0.502907	-1.097882
H	0.165473	0.155587	0.745226
H	-2.571286	-2.542960	-1.870067
H	-4.397393	-3.955283	-2.818344
H	-6.766177	-3.502572	-2.195678
H	-7.312426	-1.648337	-0.626931
H	-5.502439	-0.244819	0.320104
H	-1.365823	-1.776232	2.035106
H	-1.584137	-1.693380	4.510505
H	-3.102445	-0.026311	5.570499
H	-4.393595	1.563075	4.156770
H	-4.168464	1.499639	1.689143
H	-3.888353	0.711176	-2.493310

H -3.883517 2.951902 -3.559252
H -2.692366 4.847348 -2.475016
H -1.502530 4.504783 -0.314843
H -1.505485 2.276933 0.766661
C 6.033630 -1.558042 -0.291124
O 5.233569 -2.445224 -0.467937
O 7.357386 -1.798761 -0.220871
C 7.749525 -3.148150 -0.376135
H 7.447729 -3.542242 -1.360178
H 8.843280 -3.166753 -0.287491
H 7.302494 -3.787335 0.403612
H 7.334291 2.882157 0.257556
H 2.064496 2.723257 0.106366
59
TS1a_Z_SN2_neut_a
Eopt -4328.695804
Br 7.688970 -0.615649 0.270516
C 5.798293 -0.680288 0.090511
C 5.102886 0.495061 -0.119073
C 3.703999 0.393404 -0.245087
C 2.625779 1.291475 -0.458764
C 2.693144 2.736712 -0.619571
O 1.490090 3.303888 -0.805255
C 1.485577 4.711047 -0.964918
O 3.722070 3.379204 -0.589912
N 1.487180 0.566431 -0.486915
N 1.708132 -0.713737 -0.310779
C 3.051968 -0.871630 -0.158487
C 3.794278 -2.049750 0.055510
C 5.166367 -1.946101 0.179209
H 5.603814 1.461422 -0.184987
H 2.069452 5.014356 -1.847336
H 0.436279 5.003167 -1.096471
H 1.903316 5.213881 -0.079492
H 3.296202 -3.020050 0.121511
H 5.774315 -2.837559 0.345633
C -0.560975 0.958049 -0.619249
O -2.341819 1.170912 -0.712319
P -3.237874 0.056972 -0.108012
C -4.937220 0.646549 -0.167392
C -5.164902 2.008741 -0.389772
C -6.472108 2.491222 -0.417274
C -7.542918 1.619243 -0.223603
C -7.312991 0.259502 -0.004940
C -6.011135 -0.232239 0.022247
C -3.095684 -1.461370 -1.068845
C -2.759502 -1.340680 -2.423558
C -2.672735 -2.481803 -3.216447
C -2.924127 -3.737692 -2.662752
C -3.259514 -3.857698 -1.313888
C -3.343580 -2.722059 -0.511529
C -2.818322 -0.329572 1.604105
C -3.605566 0.157918 2.655281
C -3.228425 -0.084533 3.974485
C -2.066537 -0.805316 4.247724
C -1.276507 -1.286440 3.202533
C -1.647641 -1.053475 1.881617
H -0.363723 1.722897 -1.363578
H -0.484311 1.231409 0.428943
H -0.545539 -0.089522 -0.910033
H -4.316927 2.679286 -0.544528
H -6.653711 3.554315 -0.592501
H -8.566914 2.000542 -0.246478
H -8.153586 -0.422921 0.141079
H -5.834714 -1.298282 0.187712
H -2.558466 -0.354369 -2.849091
H -2.403332 -2.390221 -4.271285
H -2.852723 -4.631748 -3.287221
H -3.449629 -4.842572 -0.880905
H -3.593651 -2.820033 0.547777
H -4.515712 0.724747 2.445068
H -3.846605 0.293096 4.792299
H -1.772490 -0.993352 5.283400
H -0.362340 -1.845612 3.414761
H -1.016164 -1.430299 1.073136
59
TS1a_Z_SN2_neut_d
Eopt -4328.697288
Br 7.781252 0.141143 0.313173
C 6.008463 0.583446 -0.209648
C 5.025714 -0.385278 -0.139766
C 3.728975 -0.006186 -0.537593
C 2.460579 -0.635983 -0.632677
C 2.144633 -2.019051 -0.306072
O 0.861293 -2.345840 -0.541489
C 0.493053 -3.683861 -0.259217
O 2.946911 -2.816415 0.132871
N 1.581419 0.280168 -1.100722
N 2.144137 1.444490 -1.314868
C 3.460500 1.319083 -0.988712
C 4.488870 2.280443 -1.047578
C 5.759621 1.905581 -0.656173

H	5.233434	-1.398658	0.205304
H	0.702051	-3.943859	0.788433
H	-0.584228	-3.754940	-0.453555
H	1.035458	-4.386748	-0.910235
H	4.284867	3.296299	-1.394511
H	6.581607	2.623428	-0.688311
C	-0.506334	0.308308	-1.331188
O	-2.287819	0.382130	-1.533696
P	-3.196394	0.274584	-0.281393
C	-3.135991	1.803722	0.671308
C	-2.753512	2.969245	-0.004844
C	-2.726747	4.184127	0.675618
C	-3.084565	4.237453	2.022843
C	-3.465836	3.076121	2.696255
C	-3.490111	1.855634	2.025703
C	-4.877878	-0.007983	-0.860137
C	-5.079525	-0.308851	-2.211036
C	-6.372347	-0.540902	-2.677714
C	-7.454428	-0.473295	-1.801088
C	-7.250552	-0.171260	-0.453007
C	-5.963734	0.063701	0.021845
C	-2.732344	-1.080222	0.817046
C	-1.620259	-0.941453	1.661186
C	-1.217479	-2.009773	2.456613
C	-1.919739	-3.215046	2.416674
C	-3.022227	-3.357993	1.574542
C	-3.428980	-2.294875	0.770907
H	-0.427148	1.121430	-0.614183
H	-0.474216	-0.726073	-1.005079
H	-0.319768	0.541650	-2.375470
H	-2.472200	2.918139	-1.059559
H	-2.422031	5.092744	0.151016
H	-3.061216	5.191635	2.555115
H	-3.738795	3.119295	3.753250
H	-3.772883	0.946121	2.561316
H	-4.222806	-0.354041	-2.886758
H	-6.533604	-0.774350	-3.732864
H	-8.467005	-0.654647	-2.170223
H	-8.099971	-0.116139	0.231905
H	-5.807488	0.302058	1.077410
H	-1.065217	-0.000976	1.699951
H	-0.348826	-1.900191	3.109892
H	-1.602550	-4.050533	3.045763
H	-3.571162	-4.301998	1.542403
H	-4.293126	-2.409133	0.111933

54

TS2a_3CN_opt_a

Eopt -1620.338577

C	-6.696739	-1.448384	-1.059466
C	-5.827600	-2.066650	-1.990733
C	-4.485752	-1.733630	-2.022787
C	-4.021003	-0.771194	-1.108751
C	-4.904134	-0.157761	-0.173867
C	-4.044313	0.719775	0.545834
N	-2.797183	0.614522	0.059329
N	-2.771993	-0.260409	-0.922306
C	-6.268823	-0.499873	-0.147759
H	-6.227248	-2.809844	-2.684276
H	-3.811036	-2.207333	-2.740511
H	-6.949750	-0.032002	0.563642
C	-0.762107	-0.736488	-1.260517
O	1.018605	-1.028834	-1.346634
P	1.886650	-0.255174	-0.315549
C	3.555921	-0.919182	-0.411332
C	3.763346	-2.118530	-1.100221
C	5.046750	-2.657388	-1.170441
C	6.113260	-2.001689	-0.558152
C	5.905583	-0.803047	0.127316
C	4.627210	-0.256630	0.201853
C	1.915263	1.502157	-0.720245
C	1.724302	1.859417	-2.060288
C	1.769239	3.201432	-2.429508
C	2.007712	4.182211	-1.465712
C	2.196828	3.826952	-0.130581
C	2.148743	2.487666	0.247961
C	1.290318	-0.421570	1.380668
C	1.918096	-1.305640	2.269570
C	1.395823	-1.488515	3.546882
C	0.245489	-0.803575	3.939208
C	-0.386574	0.070950	3.053058
C	0.134032	0.269663	1.777372
H	-0.954406	-1.159354	-2.243138
H	-0.900668	-1.360551	-0.384409
H	-0.697879	0.343559	-1.163063
H	2.917752	-2.618898	-1.578473
H	5.212787	-3.592898	-1.708938
H	7.119125	-2.425855	-0.616709
H	6.742684	-0.289242	0.602384
H	4.466847	0.684158	0.734340
H	1.532086	1.085552	-2.807152
H	1.615441	3.482370	-3.473440
H	2.039485	5.234537	-1.758247

H 2.376233 4.597122 0.622907
H 2.283090 2.211148 1.296674
H 2.815011 -1.848611 1.964355
H 1.890118 -2.172975 4.239976
H -0.163871 -0.955466 4.939563
H -1.297980 0.599620 3.342930
H -0.387038 0.945035 1.096082
H -7.729827 -1.726998 -1.060923
C -4.424359 1.631324 1.727462
N -4.710627 2.317945 2.617519
54
TS2a_3CN_opt_c
Eopt -1620.338568
C 5.417489 -1.995743 0.999862
C 4.043104 -2.336688 1.005908
C 3.120894 -1.524002 0.372410
C 3.592248 -0.361947 -0.266209
C 4.975634 -0.029708 -0.266634
C 5.005923 1.189413 -1.002275
N 3.753727 1.514963 -1.379695
N 2.914471 0.600864 -0.949885
C 5.909485 -0.862238 0.378302
H 3.716896 -3.246154 1.514631
H 2.059853 -1.781824 0.376079
H 6.971418 -0.614252 0.382272
C 0.801165 0.706658 -1.178341
O -0.949919 0.786626 -1.408555
P -1.898205 0.229307 -0.311170
C -2.304286 1.533604 0.864941
C -1.816444 2.819659 0.610381
C -2.124953 3.859634 1.486015
C -2.918882 3.616779 2.605571
C -3.408679 2.333009 2.856659
C -3.104350 1.288401 1.989484
C -3.396488 -0.352720 -1.120919
C -3.600400 -0.021619 -2.464444
C -4.763851 -0.441977 -3.106237
C -5.715470 -1.186934 -2.410710
C -5.508141 -1.518648 -1.070317
C -4.348968 -1.104601 -0.420610
C -1.171516 -1.152212 0.592239
C -0.560722 -0.958759 1.837924
C 0.065349 -2.028165 2.474868
C 0.090955 -3.284094 1.869304
C -0.504300 -3.475560 0.621070
C -1.136424 -2.413865 -0.020136
H 1.016765 1.447830 -1.943590
H 0.836078 1.015644 -0.137586
H 0.891348 -0.345587 -1.429059
H -1.199132 3.000567 -0.272648
H -1.742250 4.864036 1.290252
H -3.159104 4.433760 3.290574
H -4.029973 2.145163 3.735476
H -3.484158 0.283446 2.191726
H -2.844225 0.557268 -2.999148
H -4.925480 -0.187755 -4.156482
H -6.626088 -1.516592 -2.917179
H -6.251991 -2.108116 -0.529255
H -4.183954 -1.374473 0.626023
H -0.572689 0.024833 2.313194
H 0.540755 -1.876547 3.446527
H 0.583432 -4.120526 2.371460
H -0.478432 -4.458530 0.145159
H -1.606028 -2.567580 -0.995457
H 6.106762 -2.644475 1.498810
C 6.257406 2.025525 -1.328323
N 7.200081 2.655324 -1.573917
59
TS2a_E_7CO2Me_opt_b
Eopt -1755.736565
C 5.785025 -0.716636 0.963656
C 4.786452 0.257814 1.144633
C 3.729792 0.424545 0.250815
C 3.653274 -0.469633 -0.856270
C 4.681589 -1.456116 -1.036362
C 4.258108 -2.146149 -2.201802
N 3.110774 -1.636705 -2.630246
N 2.737851 -0.618679 -1.839668
C 5.747620 -1.568527 -0.130638
H 4.839201 0.937898 1.997899
H 6.530193 -2.318317 -0.279679
C 0.615503 -0.569861 -1.704331
O -1.156148 -0.663113 -1.630368
P -1.879093 -0.262442 -0.314982
C -2.054618 1.529108 -0.225182
C -1.975671 2.240959 -1.428249
C -2.133574 3.624910 -1.422830
C -2.367202 4.295798 -0.222588
C -2.449563 3.585382 0.976396
C -2.296448 2.201264 0.980354
C -3.505617 -1.032251 -0.339097
C -3.821551 -1.905410 -1.384565

C -5.074431 -2.516048 -1.414011
C -6.002117 -2.256348 -0.406283
C -5.683808 -1.383233 0.636284
C -4.436581 -0.767455 0.673468
C -0.993822 -0.826758 1.149146
C 0.042794 -0.052175 1.687569
C 0.821845 -0.555760 2.724820
C 0.565624 -1.830509 3.230491
C -0.467782 -2.603523 2.699621
C -1.246919 -2.108183 1.656805
H 0.665800 -0.948986 -2.721438
H 0.724028 0.495626 -1.531009
H 0.799970 -1.264444 -0.890163
H -1.781026 1.707360 -2.361703
H -2.067276 4.182198 -2.360210
H -2.485127 5.382247 -0.219744
H -2.630454 4.113211 1.915657
H -2.353599 1.649922 1.922141
H -3.084579 -2.098463 -2.166993
H -5.325317 -3.198410 -2.229606
H -6.983348 -2.736886 -0.431976
H -6.412648 -1.180699 1.424535
H -4.190762 -0.083280 1.489831
H 0.247586 0.946516 1.299770
H 1.635160 0.052778 3.125770
H 1.178466 -2.226099 4.044240
H -0.666502 -3.601487 3.097236
H -2.050467 -2.718406 1.236517
H 4.735389 -2.983304 -2.714285
C 2.823712 1.563433 0.517287
O 2.720147 2.124102 1.588212
O 2.149965 1.973705 -0.563716
C 1.357687 3.140283 -0.407168
H 1.968095 3.982594 -0.049385
H 0.952297 3.369340 -1.399351
H 0.532914 2.979786 0.303477
H 6.600403 -0.787452 1.687749
59
TS2a_E_SN2_neut_b
Eopt -4328.695412
Br -7.362267 -1.598150 -0.305521
C -5.482993 -1.318700 -0.349108
C -4.653605 -2.428164 -0.640409
C -3.280057 -2.277844 -0.683923
C -2.744037 -1.001663 -0.431566
C -3.588257 0.109672 -0.138464
C -2.656696 1.173606 0.045726
C -2.868009 2.582594 0.367673
O -4.174642 2.859425 0.517489
C -4.497174 4.202018 0.829317
O -2.006772 3.426448 0.492579
N -1.411331 0.702136 -0.126866
N -1.449497 -0.578892 -0.410261
C -4.985949 -0.053310 -0.097252
H -5.107494 -3.402761 -0.830332
H -2.632755 -3.128801 -0.908579
H -5.589948 4.245856 0.915860
H -4.036355 4.512279 1.779626
H -4.159305 4.887264 0.036867
H -5.643715 0.787292 0.125172
C 0.534653 -1.226474 -0.622811
O 2.304891 -1.568204 -0.774817
P 3.278016 -0.510807 -0.184277
C 3.230803 -0.556145 1.617615
C 2.873367 -1.767227 2.224357
C 2.861105 -1.865732 3.613197
C 3.207404 -0.762554 4.394336
C 3.563329 0.443457 3.789950
C 3.574042 0.552424 2.401201
C 4.936214 -0.929960 -0.743228
C 5.086746 -1.903831 -1.735320
C 6.363859 -2.231963 -2.187319
C 7.480412 -1.590684 -1.652384
C 7.327105 -0.618950 -0.661157
C 6.056213 -0.286264 -0.201800
C 2.893978 1.171009 -0.708595
C 1.770457 1.813701 -0.167179
C 1.377916 3.057719 -0.652458
C 2.119560 3.674662 -1.660521
C 3.244661 3.044163 -2.194109
C 3.629553 1.788621 -1.729257
H 0.243846 -2.210704 -0.981922
H 0.546115 -1.046805 0.447589
H 0.548664 -0.394214 -1.317668
H 2.599726 -2.625165 1.605118
H 2.577080 -2.807663 4.088417
H 3.194973 -0.841954 5.484236
H 3.827330 1.308234 4.403210
H 3.839586 1.502352 1.930740
H 4.202904 -2.399524 -2.142368
H 6.485882 -2.993569 -2.961269
H 8.480517 -1.850569 -2.008404

H 8.203688 -0.119613 -0.241749
H 5.937971 0.473049 0.576188
H 1.171136 1.340653 0.612273
H 0.472650 3.520531 -0.250875
H 1.813908 4.653145 -2.039895
H 3.822204 3.528546 -2.985054
H 4.500894 1.290332 -2.160993

59
TS2a_Z_7CO2Me_opt_c
Eopt -1755.736728

C -6.474442 -0.248033 -0.023326
C -5.403600 0.623973 0.239616
C -4.072830 0.279908 -0.004806
C -3.810228 -1.016865 -0.546658
C -4.917941 -1.896720 -0.811019
C -4.291096 -3.058141 -1.326474
N -2.978498 -2.877168 -1.360017
N -2.672070 -1.656571 -0.895750
C -6.239635 -1.510263 -0.547694
H -5.611226 1.611390 0.654243
H -7.070090 -2.192100 -0.753332
C -0.566963 -1.420499 -1.005293
O 1.211293 -1.326308 -1.188971
P 2.016563 -0.353907 -0.288506
C 1.573582 1.357720 -0.638439
C 1.718464 2.372967 0.314355
C 1.385943 3.683696 -0.020092
C 0.906826 3.979223 -1.296872
C 0.761836 2.966229 -2.245595
C 1.097640 1.654770 -1.921285
C 1.752213 -0.640036 1.472885
C 0.526433 -0.265308 2.045662
C 0.282305 -0.542179 3.388007
C 1.251589 -1.184416 4.160268
C 2.469551 -1.555503 3.591447
C 2.722734 -1.287621 2.247627
C 3.761050 -0.603082 -0.655294
C 4.145417 -1.772372 -1.320488
C 5.492281 -1.990194 -1.603734
C 6.447410 -1.046283 -1.226853
C 6.060913 0.121385 -0.566418
C 4.717597 0.347891 -0.279369
H -0.698678 -1.923299 -1.958603
H -0.557165 -2.033695 -0.108528
H -0.762685 -0.358319 -0.911212
H 2.078214 2.141083 1.319813
H 1.495415 4.477569 0.722522
H 0.640974 5.007736 -1.553788
H 0.380736 3.197959 -3.242938
H 0.978487 0.855518 -2.656871
H -0.237237 0.241409 1.447641
H -0.673303 -0.254353 3.832864
H 1.055519 -1.397067 5.214269
H 3.228875 -2.056929 4.196163
H 3.677076 -1.581586 1.804266
H 3.386483 -2.498908 -1.618605
H 5.796028 -2.901568 -2.124261
H 7.502675 -1.219214 -1.452733
H 6.810081 0.861991 -0.276791
H 4.415877 1.263949 0.235275
H -7.494834 0.078433 0.191417
H -4.746357 -3.990056 -1.666069
C -3.001336 1.240372 0.314632
O -1.810015 1.039598 0.188135
O -3.460199 2.410079 0.773849
C -2.480364 3.378405 1.106655
H -1.885011 3.657648 0.224384
H -3.026775 4.252684 1.481332
H -1.798273 3.003410 1.884735

59
TS2a_Z_7CO2Me_opt_d
Eopt -1755.734006

C 6.832286 -0.664854 0.132920
C 6.151783 0.562139 0.043634
C 4.760496 0.649848 -0.032101
C 4.016062 -0.570626 -0.010129
C 4.727821 -1.817918 0.083678
C 3.689317 -2.781820 0.088057
N 2.515350 -2.170971 0.008479
N 2.693374 -0.843128 -0.050829
C 6.127550 -1.859136 0.152871
H 6.726891 1.488950 0.028166
H 6.653251 -2.815983 0.223061
C 0.777639 0.082855 -0.114273
O -0.769701 0.973078 -0.172079
P -2.102055 0.190653 -0.037278
C -2.335232 -0.920335 -1.438244
C -3.116302 -2.078796 -1.349278
C -3.284492 -2.884267 -2.473187
C -2.671848 -2.539476 -3.678467
C -1.891024 -1.386343 -3.765472
C -1.722393 -0.572976 -2.648291

C -2.191495 -0.794603 1.470058
C -1.458007 -1.988903 1.548259
C -1.454465 -2.715846 2.735747
C -2.176713 -2.260726 3.839917
C -2.902812 -1.072447 3.762737
C -2.910327 -0.334818 2.580819
C -3.432337 1.403062 -0.012789
C -3.108718 2.754224 0.146679
C -4.127521 3.704858 0.182813
C -5.458557 3.307717 0.061123
C -5.778668 1.958059 -0.100231
C -4.768176 1.001783 -0.139533
H 1.345182 0.926634 -0.495256
H 0.779666 -0.083121 0.959404
H 0.508779 -0.738470 -0.769391
H -3.585803 -2.358658 -0.403133
H -3.891762 -3.790021 -2.405025
H -2.800987 -3.177140 -4.556666
H -1.406868 -1.120418 -4.708091
H -1.106126 0.327435 -2.708578
H -0.886888 -2.349937 0.687478
H -0.881283 -3.643998 2.798200
H -2.172492 -2.836824 4.768716
H -3.467238 -0.715712 4.627525
H -3.478105 0.597017 2.523591
H -2.061495 3.050784 0.236846
H -3.878314 4.761579 0.305771
H -6.255006 4.055650 0.089153
H -6.821986 1.649325 -0.199639
H -5.020948 -0.053769 -0.271060
H 7.923648 -0.667998 0.186047
H 3.762803 -3.869080 0.147367
C 4.117848 1.972019 -0.142435
O 2.928559 2.171095 -0.290779
O 4.984494 2.988386 -0.068279
C 4.428996 4.288843 -0.160165
H 3.700085 4.464958 0.644779
H 5.268522 4.988538 -0.065728
H 3.925152 4.439560 -1.127115
59
TS2a_Z_SN2_neut_a
Eopt -4328.695161
Br 7.406476 -1.492982 -0.063603
C 5.539265 -1.247166 0.184093
C 4.761579 -2.374999 0.542019
C 3.397962 -2.251099 0.736139
C 2.818448 -0.980907 0.567284
C 3.610351 0.146507 0.203890
C 2.651970 1.197009 0.131224
C 2.894677 2.586198 -0.242671
O 1.774936 3.324398 -0.297723
C 1.946096 4.679960 -0.665889
O 3.991582 3.041814 -0.494511
N 1.437244 0.709019 0.430233
N 1.523867 -0.576454 0.694434
C 4.997714 0.013896 0.009627
H 5.249221 -3.344797 0.663488
H 2.793508 -3.118534 1.013547
H 0.947355 5.131183 -0.656344
H 2.598121 5.208047 0.047749
H 2.385884 4.770182 -1.670147
H 5.609088 0.873477 -0.266053
C -0.423462 -1.341409 0.635837
O -2.167767 -1.788563 0.489984
P -3.136676 -0.635952 0.105420
C -4.737435 -1.375090 -0.252059
C -4.814765 -2.761344 -0.420431
C -6.043161 -3.350185 -0.714941
C -7.184151 -2.559533 -0.838608
C -7.106384 -1.175859 -0.667909
C -5.883810 -0.578875 -0.372462
C -3.302877 0.521548 1.478512
C -3.094903 0.024740 2.770698
C -3.242614 0.870693 3.867006
C -3.600263 2.205857 3.674331
C -3.806395 2.701500 2.387320
C -3.656125 1.863570 1.285238
C -2.590397 0.297393 -1.340574
C -3.171094 0.065126 -2.595435
C -2.681842 0.728742 -3.717034
C -1.610344 1.614022 -3.596612
C -1.024654 1.840710 -2.349560
C -1.513679 1.190847 -1.219770
H -0.159502 -2.247085 1.175666
H -0.263314 -1.303954 -0.436156
H -0.584665 -0.427825 1.201082
H -3.912075 -3.368363 -0.316511
H -6.107661 -4.432559 -0.845445
H -8.146615 -3.024601 -1.067702
H -8.001669 -0.559247 -0.761061
H -5.825198 0.503606 -0.234116
H -2.809167 -1.020265 2.911910

H -3.075813 0.486216 4.875336
H -3.712531 2.867720 4.536485
H -4.079297 3.748678 2.238880
H -3.804120 2.256200 0.276154
H -4.005791 -0.631837 -2.695202
H -3.139828 0.551081 -4.692676
H -1.226095 2.126865 -4.480185
H -0.173730 2.518442 -2.245700
H -1.028679 1.370746 -0.258413
59
TS2a_Z_SN2_neut_c
Eopt -4328.694350
Br -6.129557 -2.498301 0.579557
C -4.621452 -1.446509 0.098649
C -3.386932 -2.109816 -0.103502
C -2.258459 -1.392503 -0.456110
C -2.379234 0.001655 -0.603382
C -3.626275 0.657443 -0.395053
C -3.308727 2.025184 -0.633227
C -4.216198 3.163796 -0.545727
O -3.621735 4.338814 -0.799501
C -4.453972 5.480592 -0.732290
O -5.394786 3.073952 -0.267535
N -2.005911 2.128570 -0.947878
N -1.448690 0.938475 -0.938770
C -4.771253 -0.078700 -0.038676
H -3.333701 -3.193206 0.020622
H -1.303115 -1.898280 -0.615165
H -3.820316 6.340767 -0.983450
H -5.286303 5.412562 -1.449544
H -4.874418 5.611940 0.276858
H -5.729070 0.418003 0.119875
C 0.641021 1.137677 -1.068605
O 2.431086 1.247219 -0.999670
P 3.190151 0.166218 -0.181232
C 3.213151 -1.397094 -1.078212
C 3.075338 -1.344994 -2.471156
C 3.125054 -2.521952 -3.213926
C 3.315627 -3.745403 -2.571099
C 3.453735 -3.797133 -1.183529
C 3.400744 -2.625643 -0.431918
C 4.875003 0.753048 0.052917
C 5.177941 2.077994 -0.276667
C 6.474517 2.553315 -0.087061
C 7.459019 1.711383 0.428559
C 7.153934 0.388385 0.755463
C 5.862947 -0.096050 0.567687
C 2.443374 -0.127178 1.432848
C 1.269681 -0.890757 1.517912
C 0.599082 -0.999902 2.732055
C 1.106238 -0.365698 3.866412
C 2.279281 0.385570 3.788138
C 2.946473 0.514588 2.572259
H 0.548384 1.993647 -1.731703
H 0.597033 0.142223 -1.501554
H 0.487747 1.276169 -0.002273
H 2.924047 -0.382689 -2.966410
H 3.012031 -2.483555 -4.299923
H 3.352612 -4.667839 -3.155973
H 3.596767 -4.756919 -0.681587
H 3.495855 -2.671630 0.655767
H 4.397429 2.725175 -0.682263
H 6.715465 3.587483 -0.344491
H 8.474698 2.087109 0.575764
H 7.927582 -0.270309 1.156935
H 5.626761 -1.132941 0.820960
H 0.862851 -1.385314 0.632788
H -0.332788 -1.566003 2.784407
H 0.578913 -0.452844 4.819840
H 2.674121 0.881595 4.677891
H 3.857389 1.115121 2.510242
38
TS5A_3CN_OTs_a
Eopt -1424.303761
C -0.684468 9.667609 8.483204
C -1.428753 8.498537 8.191317
C -2.218154 8.438689 7.059111
C -2.254948 9.566750 6.218411
C -1.502541 10.738780 6.520955
C -1.824690 11.607914 5.440048
N -2.677378 10.979407 4.611012
N -2.937441 9.775424 5.059363
C -0.700410 10.790139 7.677015
H -1.373771 7.644661 8.869267
H -2.795269 7.540879 6.826750
H -0.119080 11.679571 7.921442
C -4.259730 8.877102 3.733108
H -4.290274 7.936237 4.277470
H -3.450575 9.050839 3.032055
H -4.910325 9.686095 4.046967
Cs -3.750753 12.504588
2.034100

O -5.412874 8.285442 2.445548
S -5.641097 9.384838 1.400478
O -4.396600 9.687822 0.665771
O -6.287774 10.567212 2.003916
C -6.783961 8.631734 0.280686
C -6.300759 7.869280 -0.783206
C -8.151579 8.785129 0.492830
C -7.206620 7.253627 -1.638533
H -5.224732 7.768706 -0.939486
C -9.044814 8.161504 -0.374657
H -8.510768 9.393481 1.325344
C -8.591075 7.387111 -1.449743
H -6.833411 6.656694 -2.475400
H -10.119674 8.280676 -0.214183
C -9.551559 6.721634 -2.387556
H -10.593320 6.865590 -2.069070
H -9.447674 7.125455 -3.407869
H -9.356462 5.639354 -2.450838
C -1.372532 13.043915 5.115926
N -1.031946 14.125578 4.871782
H -0.082651 9.682477 9.367791
38
TS5A_3CN_OTs_b
Eopt -1424.293867
C -0.727742 9.894499 8.499490
C -1.480033 8.737260 8.177838
C -2.323494 8.706956 7.081450
C -2.413341 9.868482 6.289333
C -1.657792 11.031711 6.609417
C -2.041468 11.936147 5.577281
N -2.930161 11.340801 4.753167
N -3.150807 10.118423 5.171899
C -0.805006 11.041800 7.728827
H -1.386301 7.851872 8.812801
H -2.900300 7.813448 6.830893
H -0.220581 11.930050 7.982595
C -4.542422 8.951183 4.098559
H -4.575364 9.818581 3.444362
H -5.132445 8.962165 5.010592
H -3.738243 8.231004 3.978779
Cs -4.930644 5.099889 6.672986
O -5.739840 8.060524 3.107383
S -6.083184 6.667408 3.640552
O -6.811253 6.743996 4.924318
O -4.885515 5.802515 3.689717
C -7.182734 6.037615 2.409893
C -8.555481 6.234937 2.552009
C -6.659919 5.375455 1.300388
C -9.410947 5.761591 1.562903
H -8.946458 6.745963 3.434108
C -7.531106 4.908957 0.321207
H -5.582823 5.219901 1.211013
C -8.916085 5.096448 0.431795
H -10.488871 5.909039 1.671745
H -7.127526 4.383831 -0.548662
C -9.844912 4.620278 -0.642523
H -9.406239 3.793751 -1.219520
H -10.806567 4.287105 -0.225511
H -10.063733 5.436991 -1.351418
H -0.073181 9.874850 9.374727
C -1.611285 13.267987 5.369684
N -1.241154 14.360798 5.222089
43
TS5A_E_1Me_OTs_b
Eopt -4132.665838
Br 0.634647 9.473645 9.730555
C -0.462042 9.568317 8.183564
C -0.999104 8.360095 7.675116
C -1.804725 8.373283 6.552961
C -2.069640 9.614675 5.945271
C -1.525233 10.824191 6.463906
C -2.038071 11.807802 5.571546
C -1.881316 13.255251 5.542409
O -1.071957 13.696111 6.506896
C -0.850182 15.096366 6.565388
N -2.796562 11.201005 4.638579
N -2.820327 9.909473 4.849001
C -0.702531 10.800848 7.606295
H -0.770027 7.418134 8.177188
H -2.221755 7.447222 6.150828
H -0.165770 15.262971 7.405803
H -0.392634 15.462931 5.634353
H -1.791334 15.639342 6.738612
H -0.276495 11.718103 8.013596
C -4.032417 8.871099 3.501941
H -4.589137 8.459241 4.338258
H -3.138790 8.355213 3.158776
H -4.251590 9.868479 3.130908
O -2.418751 14.002468 4.741893
Cs -4.527655 12.975405
2.824962
O -5.187606 8.100884 2.306823

S -4.813407 8.469357 0.860816
O -3.779890 7.575301 0.328345
O -4.542404 9.917779 0.764927
C -6.323546 8.136647 -0.013040
C -6.609960 6.834990 -0.415604
C -7.229036 9.172205 -0.243052
C -7.822894 6.570293 -1.047089
H -5.879731 6.041773 -0.243594
C -8.435064 8.892553 -0.875370
H -6.971421 10.190620 0.055044
C -8.755375 7.588434 -1.283253
H -8.049596 5.550041 -1.368128
H -9.143949 9.703904 -1.064006
C -10.064057 7.304993 -1.956852
H -10.190479 7.928675 -2.856003
H -10.146000 6.250933 -2.256198
H -10.908442 7.535696 -1.287040
43
TS5A_Z_1Me_OTs_b
Eopt -4132.663055
Br 0.215569 9.591958 9.766200
C -0.790005 9.680522 8.159990
C -1.259765 8.462275 7.608383
C -1.994297 8.461410 6.439628
C -2.246825 9.704564 5.831647
C -1.788520 10.926196 6.395595
C -2.292539 11.909948 5.493421
C -2.126911 13.353779 5.599267
O -1.538534 13.906407 6.499933
N -2.968392 11.299684 4.499530
N -2.926060 10.011808 4.698682
C -1.036326 10.913770 7.586436
H -1.034688 7.521743 8.114855
H -2.357852 7.528710 6.003959
H -0.672441 11.839735 8.032211
C -3.863158 8.785487 3.624266
H -4.321792 8.401992 4.529527
H -2.977156 8.312788 3.218014
H -4.332739 9.601444 3.103677
O -2.694303 14.040508 4.581992
Cs -4.555371 12.920304
2.358237
C -2.522177 15.451961 4.637551
H -3.046062 15.880618 5.504077
H -1.457694 15.716656 4.703672
H -2.946378 15.858078 3.711311
O -4.967765 7.459820 2.635312
S -4.559123 7.186249 1.186251
O -4.306852 5.777401 1.016083
O -3.517792 8.122911 0.813081
C -5.958178 7.566838 0.227213
C -6.957544 6.606936 0.046799
C -6.067004 8.798287 -0.420938
C -8.038100 6.870612 -0.787784
H -6.863791 5.635962 0.543791
C -7.150188 9.057286 -1.256966
H -5.271271 9.540434 -0.296910
C -8.147453 8.095062 -1.461721
H -8.804151 6.102677 -0.938700
H -7.211810 10.017638 -1.780130
C -9.298752 8.361866 -2.377067
H -9.028364 9.092425 -3.158758
H -9.638394 7.434832 -2.870664
H -10.158310 8.773788 -1.815022
43
TS5A_Z_7CO2Me_OTs_a
Eopt -1559.696342
C -1.221839 9.872443 8.802014
C -2.099141 8.803266 8.538770
C -2.801361 8.678287 7.340993
C -2.605340 9.691919 6.352344
C -1.704268 10.776287 6.634574
C -1.759723 11.559932 5.458175
N -2.595645 10.996857 4.592763
N -3.112253 9.874159 5.114101
C -1.017837 10.862724 7.856132
H -2.241313 8.035131 9.299896
H -0.336521 11.695069 8.054774
C -4.437851 9.085975 3.679177
H -4.180222 9.892703 2.999307
H -5.175565 9.269899 4.453984
H -3.781796 8.223661 3.746782
Cs -6.535194 5.121169 5.439108
O -5.603489 8.305821 2.532785
S -6.564390 7.119624 2.410181
O -7.664164 7.219848 3.391325
O -5.837338 5.834725 2.458332
C -7.239060 7.323949 0.788866
C -8.381399 8.105394 0.616654
C -6.601487 6.731713 -0.298832
C -8.884058 8.293016 -0.665781
H -8.870994 8.555818 1.482507

C -7.119017 6.930218 -1.575823
H -5.711730 6.117989 -0.144098
C -8.264169 7.710797 -1.781738
H -9.779482 8.904796 -0.806244
H -6.622512 6.467911 -2.433188
C -8.832830 7.902696 -3.154575
H -8.107386 7.631210 -3.934354
H -9.727049 7.272541 -3.296272
H -9.145572 8.945434 -3.315774
H -0.702221 9.911664 9.762311
H -1.233094 12.486457 5.223041
C -3.705192 7.526137 7.136152
O -4.384099 7.320862 6.153972
O -3.711978 6.685495 8.178846
C -4.554922 5.553231 8.064593
H -4.265330 4.927465 7.206811
H -4.436100 4.985709 8.995777
H -5.607024 5.851695 7.941261
43
TS5A_Z_7CO2Me_OTs_b
Eopt -1559.697625
C -1.187496 9.856806 8.764793
C -2.087242 8.795825 8.495991
C -2.767669 8.742705 7.294657
C -2.536889 9.769211 6.359240
C -1.630691 10.830348 6.641137
C -1.695039 11.624975 5.463242
N -2.558610 11.061193 4.598328
N -3.060862 9.964647 5.118937
C -0.940695 10.878132 7.867039
H -2.238505 8.018685 9.247647
H -0.245166 11.688169 8.088674
C -4.389215 9.083581 3.776221
H -4.610442 8.256558 4.446628
H -3.502918 9.026792 3.153343
H -4.926152 10.017324 3.905586
Cs -3.219494 12.265513
1.732866
O -5.504315 8.465666 2.480347
S -5.471033 9.398062 1.262165
O -4.129928 9.416060 0.644229
O -5.992460 10.737002 1.603095
C -6.597361 8.622106 0.142887
C -6.125908 7.646612 -0.735572
C -7.945865 8.969382 0.177234
C -7.024753 7.013326 -1.586008
H -5.063853 7.393582 -0.752968
C -8.831443 8.325021 -0.682122
H -8.295343 9.740293 0.867011
C -8.389408 7.339753 -1.574770
H -6.660572 6.248045 -2.277012
H -9.891005 8.593559 -0.659848
C -9.340919 6.664451 -2.514562
H -10.386412 6.824546 -2.215784
H -9.221372 7.058380 -3.537894
H -9.153269 5.581125 -2.563395
H -0.676322 9.867802 9.704730
H -1.144970 12.526656 5.292082
C -3.747074 7.592706 6.994877
O -4.365491 7.518655 5.937341
O -3.888031 6.616840 8.030597
C -4.817986 5.612445 7.616743
H -4.459488 5.139594 6.726355
H -4.922068 4.881789 8.391475
H -5.768242 6.064412 7.422733
38
TS5B_3CN_OTs_a
Eopt -1424.300542
C -0.951836 11.640460 8.920316
C -1.786021 10.543105 8.830671
C -2.388825 10.301275 7.581162
C -3.277627 9.333094 7.043950
N -3.491394 9.642007 5.750233
N -2.831328 10.718150 5.383119
C -2.140370 11.158047 6.471341
C -1.283311 12.269597 6.598677
C -0.693495 12.503159 7.825528
H -1.975720 9.887237 9.680788
H -1.085913 12.931909 5.751892
H -0.022965 13.354009 7.961050
C -4.576044 8.866880 4.160134
H -4.906399 8.027914 4.765624
H -3.600691 8.821096 3.685389
H -5.118553 9.805150 4.224543
Cs -3.058464 12.007657
2.447406
O -5.473268 8.294044 2.680308
S -5.257194 9.256625 1.507505
O -3.844083 9.268355 1.078020
O -5.797796 10.596645 1.816058
C -6.233196 8.538793 0.220853
C -5.633631 7.681987 -0.697899

C -7.599365 8.818307 0.162323
C -6.418751 7.095883 -1.687995
H -4.561591 7.483378 -0.640215
C -8.367015 8.223724 -0.831227
H -8.050867 9.500965 0.885268
C -7.792639 7.352919 -1.770918
H -5.952888 6.424540 -2.414254
H -9.437580 8.440654 -0.883638
C -8.638545 6.722662 -2.834808
H -9.123960 7.490271 -3.458738
H -8.044637 6.072417 -3.491898
H -9.443975 6.116905 -2.389263
H -0.479712 11.848550 9.857704
C -3.900306 8.135284 7.784978
N -4.369337 7.233038 8.343154
38
TS5B_3CN_OTs_b
Eopt -1424.302189
C -2.300815 11.509913 9.548398
C -2.166569 10.549759 8.563160
C -2.991841 10.676850 7.428920
C -3.202220 9.967326 6.214404
N -4.164656 10.619466 5.518939
N -4.602694 11.674590 6.148573
C -3.916834 11.756680 7.324660
C -4.027034 12.717051 8.347956
C -3.215018 12.587927 9.458217
H -1.455081 9.729095 8.657042
H -4.737365 13.542832 8.263207
H -3.271826 13.313250
10.272160
C -5.090072 10.253390 3.674307
H -5.395614 9.297706 4.088917
H -4.103997 10.346339 3.229919
H -5.648954 11.150278 3.928769
Cs -2.974855 6.517559 2.243027
O -5.981753 10.073093 2.116360
S -5.552619 8.864338 1.282648
O -5.849998 7.594834 1.976980
O -4.149844 8.988227 0.836138
C -6.599104 8.982044 -0.138069
C -7.822126 8.316622 -0.146150
C -6.197697 9.766061 -1.220427
C -8.653044 8.442243 -1.256800
H -8.115941 7.701865 0.707016
C -7.039853 9.880491 -2.319713
H -5.231354 10.274146 -1.199664
C -8.279803 9.223564 -2.357450
H -9.613298 7.919855 -1.269327
H -6.729675 10.492058 -3.171611
C -9.170639 9.356284 -3.554772
H -9.396880 10.414216 -3.762525
H -8.681510 8.951223 -4.455535
H -10.121007 8.822977 -3.413848
H -1.685070 11.435244 10.420282
C -2.468341 8.688427 5.770063
N -1.915549 7.725101 5.435364
43
TS5B_E_2Me_OTs_a
Eopt -4132.661035
Br -1.663195 11.355648
11.128708
C -2.509952 11.518159 9.435651
C -2.335945 10.514470 8.501538
C -2.986294 10.672052 7.262560
C -3.086889 9.943791 6.043484
C -2.511440 8.654605 5.694232
O -2.488135 8.163550 4.573748
O -1.925091 8.064942 6.738960
N -3.899509 10.651042 5.215937
N -4.313696 11.762251 5.755768
C -3.775649 11.830144 7.006470
C -3.928610 12.835630 7.978550
C -3.293517 12.672732 9.194485
H -1.725519 9.636843 8.713533
H -4.537662 13.719428 7.774598
H -3.392167 13.428413 9.975970
C -4.826681 10.233482 3.688948
H -5.066975 9.324792 4.231511
H -3.940863 10.284116 3.066299
H -5.346735 11.156453 3.921635
Cs -2.433299 7.008082 1.937211
C -1.287623 6.822521 6.496667
H -0.838810 6.517520 7.449622
H -2.013198 6.061794 6.170103
H -0.500774 6.921385 5.732915
O -5.944135 9.887907 2.240409
S -5.403740 8.879977 1.228856
O -5.460354 7.502021 1.754968
O -4.066161 9.284469 0.747052
C -6.541269 9.013929 -0.120334
C -7.585955 8.098906 -0.231235

C -6.393072 10.052950 -1.039500
C -8.492453 8.231239 -1.280007
H -7.680968 7.288562 0.494257
C -7.309635 10.170642 -2.078973
H -5.566813 10.760368 -0.942271
C -8.372467 9.265454 -2.217970
H -9.313100 7.514564 -1.372616
H -7.199069 10.984770 -2.800641
C -9.346885 9.397397 -3.349317
H -10.189925 10.048338 -
3.061451
H -8.874502 9.851409 -4.232777
H -9.764009 8.422793 -3.641733
43
TS5B_E_7CO2Me_OTs_a
Eopt -1559.699605
C -6.111954 2.390336 0.356316
C -5.019620 3.209638 0.113012
C -3.762907 2.613627 -0.066218
C -2.451976 3.090193 -0.301903
N -1.635759 2.044364 -0.365594
N -2.281976 0.883344 -0.195802
C -3.586007 1.186343 -0.013528
C -4.726632 0.357603 0.225092
C -5.953773 0.990131 0.413989
H -5.137260 4.296041 0.064538
H -6.822503 0.356757 0.600426
C 0.450882 1.856203 -0.510685
H 0.190009 1.079905 -1.225678
H 0.382527 1.688043 0.557402
H 0.525279 2.882551 -0.866374
Cs -0.532933 -1.660209 -
0.101014
O 2.223633 1.583540 -0.601248
S 2.581779 0.135576 -0.224740
O 2.295545 -0.783769 -1.343868
O 1.944747 -0.252000 1.049821
C 4.327899 0.214152 0.017156
C 5.176165 0.054423 -1.076600
C 4.836349 0.465382 1.290673
C 6.553410 0.149286 -0.885483
H 4.764838 -0.151407 -2.067886
C 6.211410 0.557461 1.462644
H 4.152964 0.580288 2.137392
C 7.092448 0.404188 0.380872
H 7.223636 0.022057 -1.740221
H 6.613154 0.754861 2.461487
C 8.572543 0.497205 0.592111
H 8.842579 1.435623 1.100187
H 8.931330 -0.327104 1.227803
H 9.117664 0.451808 -0.362333
H -7.103227 2.822952 0.501977
C -4.759033 -1.121585 0.279286
O -3.569034 -1.713632 0.077785
O -5.764325 -1.774300 0.457888
H -2.087431 4.109837 -0.416617
C -3.591520 -3.134526 0.041958
H -3.834077 -3.555612 1.031549
H -4.333680 -3.499694 -0.686199
H -2.589431 -3.457647 -0.271260
43
TS5B_Z_2Me_OTs_a
Eopt -4132.659224
Br -0.766921 12.808223
10.295398
C -1.688716 12.235028 8.737509
C -2.087152 10.916204 8.637325
C -2.762278 10.542143 7.459424
C -3.328254 9.350476 6.937389
C -3.371513 8.040884 7.580420
O -4.003580 7.116117 6.846567
C -4.084063 5.817595 7.409241
O -2.891260 7.805700 8.668729
N -3.843320 9.625656 5.722615
N -3.670737 10.888738 5.398176
C -3.014701 11.490140 6.429470
C -2.599040 12.830283 6.566391
C -1.935656 13.193252 7.722188
H -1.890661 10.189076 9.425813
H -4.645356 5.829886 8.355928
H -4.609467 5.196607 6.673377
H -3.082328 5.403707 7.599165
H -2.792623 13.567246 5.781911
H -1.595906 14.221225 7.862784
C -4.569157 8.435867 4.186015
H -5.407999 8.165894 4.818067
H -3.651458 7.864185 4.275328
H -4.571240 9.375697 3.641106
Cs -4.362948 12.540256
2.859434
O -5.252761 7.470859 2.814008
S -4.476032 7.456814 1.474897

O -3.899492 6.144729 1.230594
O -3.581293 8.611756 1.408615
C -5.751226 7.695713 0.281356
C -6.496946 6.603952 -0.167472
C -6.020335 8.973987 -0.198670
C -7.521164 6.805741 -1.085313
H -6.263815 5.601815 0.200041
C -7.047044 9.164446 -1.120799
H -5.401856 9.809247 0.134300
C -7.818137 8.087766 -1.574160
H -8.103233 5.948950 -1.437466
H -7.249161 10.169028 -1.504048
C -8.939164 8.288208 -2.547189
H -8.941080 9.307213 -2.960149
H -8.873750 7.573899 -3.383413
H -9.914046 8.118662 -2.058199
43
TS5B_Z_7CO2Me_OTs_a
Eopt -1559.682792
C -2.350507 11.740510 9.367057
C -2.294713 10.715529 8.437738
C -3.179941 10.749789 7.346096
C -3.432356 9.934948 6.219737
N -4.416904 10.496272 5.517706
N -4.858292 11.625050 6.069366
C -4.131572 11.820124 7.189045
C -4.171472 12.866780 8.159989
C -3.275109 12.796048 9.222992
H -1.576007 9.898477 8.552291
H -3.295587 13.590095 9.970783
C -5.387205 10.014174 3.687871
H -5.602136 9.052392 4.145880
H -4.417776 10.188697 3.229665
H -5.992910 10.872209 3.969768
Cs -3.010620 6.451519 2.257660
O -6.282945 9.740436 2.186481
S -5.700222 8.644515 1.286955
O -5.935218 7.296492 1.841943
O -4.280847 8.918271 0.976520
C -6.646031 8.820960 -0.193867
C -7.612043 7.871830 -0.512228
C -6.413342 9.922173 -1.020617
C -8.356723 8.030435 -1.679327
H -7.772712 7.017076 0.147523
C -7.167371 10.065271 -2.177647
H -5.647000 10.656068 -0.762110

C -8.151274 9.125470 -2.526629
H -9.115117 7.286167 -1.936325
H -6.989383 10.924409 -2.830220
C -8.965032 9.307925 -3.770927
H -9.655936 10.160292 -3.661403
H -8.323192 9.526118 -4.638442
H -9.564926 8.415649 -3.997707
H -1.674330 11.740088 10.225764
C -5.127363 13.993377 8.057902
O -5.983020 14.125255 7.215524
O -4.950827 14.899300 9.037803
C -5.833729 16.003472 9.026396
H -6.881053 15.679387 9.128372
H -5.555858 16.632024 9.881943
H -5.738818 16.581755 8.094283
H -2.957385 9.004059 5.906207
43
TS5B_Z_7CO2Me_OTs_b
Eopt -1559.705113
C -2.300815 11.509913 9.548398
C -2.166569 10.549759 8.563160
C -2.991841 10.676850 7.428920
C -3.202220 9.967326 6.214404
N -4.164656 10.619466 5.518939
N -4.602694 11.674590 6.148573
C -3.916834 11.756680 7.324660
C -4.027034 12.717051 8.347956
C -3.215018 12.587927 9.458217
H -1.455081 9.729095 8.657042
H -3.271826 13.313250
10.272160
C -5.090072 10.253390 3.674307
H -5.395614 9.297706 4.088917
H -4.103997 10.346339 3.229919
H -5.648954 11.150278 3.928769
Cs -2.974855 6.517559 2.243027
O -5.981753 10.073093 2.116360
S -5.552619 8.864338 1.282648
O -5.849998 7.594834 1.976980
O -4.149844 8.988227 0.836138
C -6.599104 8.982044 -0.138069
C -7.822126 8.316622 -0.146150
C -6.197697 9.766061 -1.220427
C -8.653044 8.442243 -1.256800
H -8.115941 7.701865 0.707016
C -7.039853 9.880491 -2.319713

H -5.231354 10.274146 -1.199664
C -8.279803 9.223564 -2.357450
H -9.613298 7.919855 -1.269327
H -6.729675 10.492058 -3.171611
C -9.170639 9.356284 -3.554772
H -9.396880 10.414216 -3.762525
H -8.681510 8.951223 -4.455535
H -10.121007 8.822977 -3.413848
H -1.685070 11.435244 10.420282
C -5.028278 13.881027 8.228498
O -5.520504 13.621969 7.134465
O -5.436725 15.014060 8.999412
C -6.458320 15.726382 8.296673
H -7.297107 15.081602 8.136693
H -6.764554 16.573270 8.874515
H -6.077707 16.057271 7.352985
H -2.692317 9.078740 5.905673
59
ts_opt_TS1b_2p2_2Me-indazole_a
Eopt -4328.651585
Br 2.430400 10.015030 5.622195
C 0.580742 10.145617 5.206823
C -0.243187 9.068593 5.471426
C -1.596326 9.200505 5.109742
C -2.750258 8.374769 5.148214
C -2.845649 6.998238 5.613320
O -4.107385 6.573785 5.759267
C -4.270429 5.211042 6.104778
O -1.884881 6.298189 5.859688
N -3.764989 9.074484 4.604057
N -3.403283 10.269497 4.220420
C -2.077325 10.400001 4.509012
C -1.211540 11.482148 4.263733
C 0.118254 11.346313 4.614212
H 0.133191 8.146830 5.915821
H -3.804913 4.985570 7.076162
H -5.351610 5.033870 6.161348
H -3.827108 4.553492 5.341300
H -1.578891 12.400421 3.799679
H 0.822822 12.159941 4.431723
C -5.824747 8.620018 4.131184
O -4.775460 7.429042 2.887674
P -4.202942 7.902137 1.535944
C -4.291268 6.514970 0.380486
C -5.154014 5.453364 0.669901
C -5.249329 4.380501 -0.215061
C -4.487274 4.369400 -1.383063
C -3.623035 5.428260 -1.669176
C -3.519261 6.501174 -0.787891
C -5.128793 9.277325 0.808517
C -5.905279 9.089092 -0.342160
C -6.656036 10.145872 -0.854287
C -6.635619 11.388624 -0.222408
C -5.865114 11.576940 0.926039
C -5.111694 10.527601 1.446639
C -2.460126 8.358606 1.631564
C -1.614396 7.467111 2.306434
C -0.253775 7.741218 2.402703
C 0.266227 8.904378 1.834624
C -0.572434 9.788521 1.156813
C -1.935530 9.517177 1.049001
H -6.547735 8.287814 3.384159
H -5.828386 8.040265 5.052473
H -5.793876 9.705978 4.242760
H -5.737427 5.472441 1.593212
H -5.919603 3.547568 0.010956
H -4.562119 3.526496 -2.074844
H -3.021992 5.414744 -2.581582
H -2.835736 7.326003 -1.007205
H -5.925817 8.117978 -0.841815
H -7.259598 9.995168 -1.752568
H -7.224253 12.215985 -0.627060
H -5.849468 12.550092 1.423057
H -4.511105 10.676043 2.353231
H -2.020977 6.560098 2.760013
H 0.400711 7.052045 2.940979
H 1.332334 9.125858 1.927864
H -0.165185 10.699970 0.712983
H -2.587522 10.213714 0.517979
59
ts_opt_TS1b_2p2_2Me-indazole_b
Eopt -4328.647473
Br 1.164600 12.550600 4.645300
C -0.502500 11.670900 4.445300
C -0.659600 10.399400 4.960900
C -1.911400 9.787400 4.764800
C -2.476600 8.512400 5.032100
C -1.792300 7.382800 5.633200
O -2.555400 6.265400 5.720400
C -1.905600 5.136100 6.267400
O -0.645400 7.399000 6.017800
N -3.730600 8.512500 4.521000

N	-4.042000	9.649600	3.968400
C	-2.957600	10.465600	4.080800
C	-2.768800	11.765600	3.573500
C	-1.535600	12.358300	3.756900
H	0.144700	9.875200	5.477100
H	-1.560100	5.334600	7.292800
H	-2.642300	4.323400	6.266300
H	-1.029900	4.855300	5.663000
H	-3.570900	12.275500	3.036100
H	-1.338200	13.360900	3.374000
C	-5.368000	7.053300	4.689400
O	-5.485800	7.531200	2.746900
P	-4.520900	7.872300	1.599400
C	-4.906100	6.735400	0.233500
C	-6.212700	6.243000	0.141900
C	-6.566800	5.402800	-0.910300
C	-5.620600	5.051800	-1.873300
C	-4.316200	5.535600	-1.780500
C	-3.955600	6.374000	-0.727500
C	-4.802700	9.528500	0.931200
C	-4.171900	9.950700	-0.244300
C	-4.433800	11.220500	-0.753200
C	-5.339500	12.057600	-0.102200
C	-5.984400	11.625900	1.057100
C	-5.716200	10.363700	1.580100
C	-2.762400	7.617400	1.915300
C	-2.368400	6.338500	2.334800
C	-1.032400	6.083800	2.617800
C	-0.083900	7.100900	2.488900
C	-0.472300	8.368000	2.065300
C	-1.811500	8.631400	1.778400
H	-6.104500	6.400100	4.221200
H	-4.533000	6.537700	5.163600
H	-5.795400	7.888700	5.242300
H	-6.939200	6.518900	0.909500
H	-7.586700	5.016900	-0.978000
H	-5.900000	4.391300	-2.697700
H	-3.571700	5.251700	-2.527800
H	-2.926300	6.732600	-0.643200
H	-3.475600	9.291400	-0.768800
H	-3.933200	11.554800	-1.664600
H	-5.546600	13.052300	-0.504200
H	-6.695900	12.281800	1.564100
H	-6.175900	10.019400	2.506500
H	-3.113200	5.546900	2.451200
H	-0.727600	5.090100	2.953900
H	0.961700	6.904300	2.734700
H	0.262200	9.171700	1.984800
H	-2.114300	9.636100	1.479100

59

ts_opt_TS1c_4p2_2Me-indazole

Eopt -4328.640879

Br	5.768070	-1.786270	0.470295
C	4.016968	-1.185853	0.047257
C	2.994510	-2.157494	-0.079356
C	1.699385	-1.777332	-0.385220
C	1.443714	-0.406563	-0.563414
C	2.483328	0.562539	-0.439906
C	1.806328	1.791538	-0.678683
C	2.377249	3.133470	-0.650557
O	1.462466	4.096752	-0.847569
C	1.959332	5.429739	-0.843468
O	3.551530	3.369275	-0.465535
N	0.502547	1.502749	-0.897038
N	0.271328	0.223545	-0.848385
C	3.795648	0.168072	-0.127427
H	3.235048	-3.213773	0.070721
H	0.902640	-2.517476	-0.484985
H	1.093031	6.079097	-1.009290
H	2.428994	5.672233	0.118872
H	2.701297	5.577206	-1.644127
H	4.594774	0.903270	-0.028061
C	-0.939100	2.559721	-2.114180
O	-2.116703	1.534349	-0.850793
P	-2.283305	0.187856	-0.130177
C	-4.001789	0.223600	0.492898
C	-5.014984	0.541201	-0.424406
C	-6.343836	0.584607	-0.010920
C	-6.673786	0.317228	1.319959
C	-5.671232	0.009847	2.234400
C	-4.335761	-0.036624	1.825588
C	-2.290618	-1.287853	-1.184177
C	-2.898132	-2.464343	-0.726423
C	-2.933515	-3.591883	-1.546205
C	-2.404230	-3.536140	-2.834322
C	-1.830592	-2.352765	-3.303749
C	-1.772011	-1.231208	-2.481120
C	-1.283381	-0.018917	1.368399
C	-0.881233	1.152574	2.024367
C	-0.118370	1.064705	3.187554
C	0.231279	-0.184952	3.701245
C	-0.176193	-1.348077	3.048219

C	-0.933686	-1.266255	1.883094
H	-0.227489	2.156078	-2.836719
H	-1.955810	2.609740	-2.499535
H	-0.594551	3.436288	-1.563804
H	-4.760362	0.749688	-1.465324
H	-7.130658	0.828892	-0.732007
H	-7.718593	0.355242	1.640603
H	-5.922581	-0.194647	3.278874
H	-3.555818	-0.272690	2.554378
H	-3.347947	-2.505376	0.265556
H	-3.396771	-4.512139	-1.180867
H	-2.440394	-4.419867	-3.480053
H	-1.423992	-2.302992	-4.316226
H	-1.304128	-0.310787	-2.833430
H	-1.145121	2.126926	1.606768
H	0.206155	1.977076	3.693322
H	0.834068	-0.251415	4.612104
H	0.101407	-2.326429	3.446757
H	-1.233305	-2.185085	1.371268

59

ts_opt_TS2b_2p2_1Me-indazole

Eopt -4328.643833

Br	0.390096	13.083354	3.406012
C	-0.936532	11.781705	3.803194
C	-0.578337	10.650819	4.512686
C	-1.587612	9.701823	4.753048
C	-1.668551	8.423593	5.371544
C	-0.565448	7.674727	5.960790
O	-0.922143	6.465393	6.410741
C	0.114439	5.689327	6.980718
O	0.573530	8.091370	6.031699
N	-2.921473	7.949924	5.247483
N	-3.656026	8.816117	4.599058
C	-2.913023	9.912692	4.275884
C	-3.251145	11.083545	3.574016
C	-2.253585	12.012759	3.339737
H	0.440955	10.480541	4.860177
H	0.560903	6.193781	7.851551
H	-0.344463	4.743843	7.296195
H	0.912330	5.488253	6.249020
H	-4.263094	11.255641	3.203899
H	-2.476909	12.928769	2.789329
C	-5.734572	8.150771	4.601290
O	-5.112997	7.386056	2.867713
P	-4.354542	7.808685	1.597847
C	-4.592424	6.490419	0.375456
C	-5.791298	5.768457	0.411972
C	-6.027213	4.770887	-0.530517
C	-5.071155	4.492817	-1.508581
C	-3.874541	5.208714	-1.542504
C	-3.629230	6.205976	-0.600024
C	-5.043375	9.310813	0.859207
C	-4.658086	9.730190	-0.421553
C	-5.220005	10.877294	-0.975189
C	-6.177516	11.601635	-0.263417
C	-6.581394	11.172069	0.999691
C	-6.018527	10.026805	1.560960
C	-2.565192	7.978993	1.775238
C	-1.900603	6.934054	2.430923
C	-0.515071	6.968919	2.560341
C	0.209440	8.041578	2.039918
C	-0.451833	9.085069	1.392716
C	-1.838530	9.058899	1.260499
H	-6.613117	7.723727	4.114942
H	-5.284454	7.515975	5.365132
H	-5.851353	9.202311	4.864265
H	-6.529878	5.985729	1.186999
H	-6.961003	4.204091	-0.499382
H	-5.257667	3.707880	-2.245896
H	-3.122208	4.985182	-2.302737
H	-2.683854	6.754037	-0.619155
H	-3.924142	9.158840	-0.995001
H	-4.913212	11.203670	-1.971754
H	-6.618266	12.500473	-0.701781
H	-7.341700	11.728859	1.552642
H	-6.343131	9.688032	2.545512
H	-2.469127	6.103864	2.856875
H	0.000152	6.158910	3.082136
H	1.295951	8.070475	2.152819
H	0.112432	9.935097	1.002256
H	-2.349327	9.889362	0.770797

[i] M. J. Frisch, G. W. Trucks, H. B. Schlegel, G. E. Scuseria, M. a. Robb, J. R. Cheeseman, G. Scalmani, V. Barone, G. a. Petersson, H. Nakatsuji, X. Li, M. Caricato, a. V. Marenich, J. Bloino, B. G. Janesko, R. Gomperts, B. Mennucci, H. P. Hratchian, J. V. Ortiz, a. F. Izmaylov, J. L. Sonnenberg, Williams, F. Ding, F. Lipparini, F. Egidi, J. Goings, B. Peng, A. Petrone, T. Henderson, D. Ranasinghe, V. G. Zakrzewski, J. Gao, N. Rega, G. Zheng, W. Liang, M. Hada, M. Ehara, K.

Toyota, R. Fukuda, J. Hasegawa, M. Ishida, T. Nakajima, Y. Honda, O. Kitao, H. Nakai, T. Vreven, K. Throssell, J. a. Montgomery Jr., J. E. Peralta, F. Ogliaro, M. J. Bearpark, J. J. Heyd, E. N. Brothers, K. N. Kudin, V. N. Staroverov, T. a. Keith, R. Kobayashi, J. Normand, K. Raghavachari, a. P. Rendell, J. C. Burant, S. S. Iyengar, J. Tomasi, M. Cossi, J. M. Millam, M. Klene, C. Adamo, R. Cammi, J. W. Ochterski, R. L. Martin, K. Morokuma, O. Farkas, J. B. Foresman, D. J. Fox, 2016, Gaussian 16, Revision C.01, Gaussian, Inc., Wallin.

[ii] Adamo, C.; Barone, V. Toward Reliable Density Functional Methods Without Adjustable Parameters: The PBE0Model. *J. Chem.Phys.* **1999**, *110(13),* 6158−6170.

[iii] A. V. Marenich, C. J. Cramer, D. G. Truhlar, J. Phys. Chem. B 2009, 113, 6378–6396.

[iv] G. Luchini, J. Alegre-Requena, I. Funes-Ardoiz, R. Paton, GoodVibes: automated thermochemistry for heterogeneous computational chemistry data. *F1000Research* **2020**, 9 (291).
